# A Genome-Wide Comparison of Rice False Smut Fungus *Villosiclava virens* Albino Strain LN02 Reveals the Genetic Diversity of Secondary Metabolites and the Cause of Albinism

**DOI:** 10.3390/ijms242015196

**Published:** 2023-10-15

**Authors:** Mengyao Xue, Siji Zhao, Gan Gu, Dan Xu, Xuping Zhang, Xuwen Hou, Jiankun Miao, Hai Dong, Dongwei Hu, Daowan Lai, Ligang Zhou

**Affiliations:** 1 Department of Plant Pathology, College of Plant Protection, China Agricultural University, Beijing 100193, China; mengyaoxue@cau.edu.cn (M.X.); sijizhao@cau.edu.cn (S.Z.); gangu@cau.edu.cn (G.G.); cauxudan@cau.edu.cn (D.X.); zhangxuping5@cau.edu.cn (X.Z.); xwhou@cau.edu.cn (X.H.); dwlai@cau.edu.cn (D.L.); 2 Institute of Plant Protection, Liaoning Academy of Agricultural Science, Shenyang 110161, China; mjkkx@163.com (J.M.); lnsydh@163.com (H.D.); 3 Biotechnology Institute, Zhejiang University, Hangzhou 310058, China; hudw@zju.edu.cn

**Keywords:** rice false smut, *Villosiclava virens* (*Ustilaginoidea virens*), albino strain, genome-wide comparison, ustilaginoidins, sorbicillinoids

## Abstract

Rice false smut (RFS) caused by *Villosiclava virens* (anamorph: *Ustilaginoidea virens*) has become one of the most destructive fungal diseases to decrease the yield and quality of rice grains. An albino strain LN02 was isolated from the white RFS balls collected in the Liaoning Province of China in 2019. The strain LN02 was considered as a natural albino mutant of *V. virens* by analyzing its phenotypes, internal transcribed spacer (ITS) conserved sequence, and biosynthesis gene clusters (BGCs) for secondary metabolites. The total assembled genome of strain LN02 was 38.81 Mb, which was comprised of seven nuclear chromosomes and one mitochondrial genome with an N50 value of 6,326,845 bp and 9339 protein-encoding genes. In addition, the genome of strain LN02 encoded 19 gene clusters for biosynthesis of secondary metabolites mainly including polyketides, terpenoids and non-ribosomal peptides (NRPs). Four sorbicillinoid metabolites were isolated from the cultures of strain LN02. It was found that the polyketide synthase (PKS)-encoding gene *uspks1* for ustilaginoidin biosynthesis in strain LN02 was inactivated due to the deletion of four bases in the promoter sequence of *uvpks1*. The normal *uvpks1* complementary mutant of strain LN02 could restore the ability to synthesize ustilaginoidins. It demonstrated that deficiency of ustilaginoidin biosynthesis is the cause of albinism for RFS albino strain LN02, and *V. virens* should be a non-melanin-producing fungus. This study further confirmed strain LN02 as a white phenotype mutant of *V. virens*. The albino strain LN02 will have a great potential in the development and application of secondary metabolites. The physiological and ecological functions of ustilaginoidins in RFS fungus are needed for further investigation.

## 1. Introduction

Rice false smut (RFS) caused by *Villosiclava virens* (anamorph: *Ustilaginoidea virens*) is the most common disease to decrease the yield and quality of rice grains [1,2]. With global climate change, cultivation of high-yield varieties, dense planting, and widespread application of nitrogen fertilizers, RFS not only leads to a serious decrease in rice yield, but the toxic metabolites produced by *V. virens* also contaminate grains and straw to cause poisoning to humans and animals [3,4,5,6].

**Figure 1:**
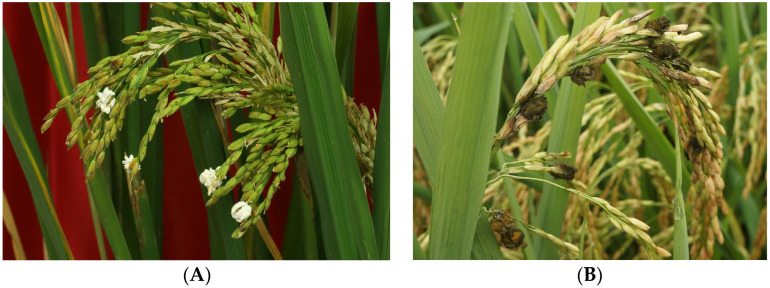
The albino balls (**A**) and normal balls (**B**) of RFS appeared in the rice field.

The typical feature of RFS is the formation of balls on rice panicles. The color of normal RFS balls appears from the initial white to yellow, and finally to greenish black [7,8,9]. Unlike normal RFS balls, the matured white RFS balls remained completely white (Figure 1). After white RFS was first reported in Japan in 1991 [10], it was later reported in China [11,12] and the United States [13]. As the RFS balls, chlamydospores, and conidia, as well as the rDNA-ITS sequences of the isolated white RFS fungus, were very similar to those of the normal RFS fungus, both fungal strains were commonly considered the same species [10,12,13]. However, Wang et al. classified the white RFS fungus as an independent species, named as *Ustilaginoidea albicans*, based on the sporulation, pigment accumulation, and pathogenicity [11]. Therefore, the taxonomic status of albino RFS fungus requires further confirmation.

The complete genome sequencing of normal RFS fungus has been reported by a few research groups, with some strains including UV8b [14], IPU010 [15], UV_Gvt [16], UV-FJ-1 [17], JS60-2 [18], and UV2_4G [19]. Unfortunately, there is no detailed report on the genome of albino RFS fungus. In this study, the phenotype characters and genome sequences of RFS fungus albino strain LN02 were comparatively analyzed with those of normal strains, and some genes and their functions specially related to secondary metabolism were annotated in order to provide more evidences to support taxonomic status of albino RFS fungus, to reveal the causes of albinism and to clarify the diversity of secondary metabolites. In addition, the main sorbicillinoids from strain LN02 and ustilaginoidins from the normal *uvpks1* complementary mutant of strain LN02 were also isolated and identified.

## 2. Results

### 2.1. Phenotype Characters of RFS Fungus Albino Strain LN02

**Figure 2:**
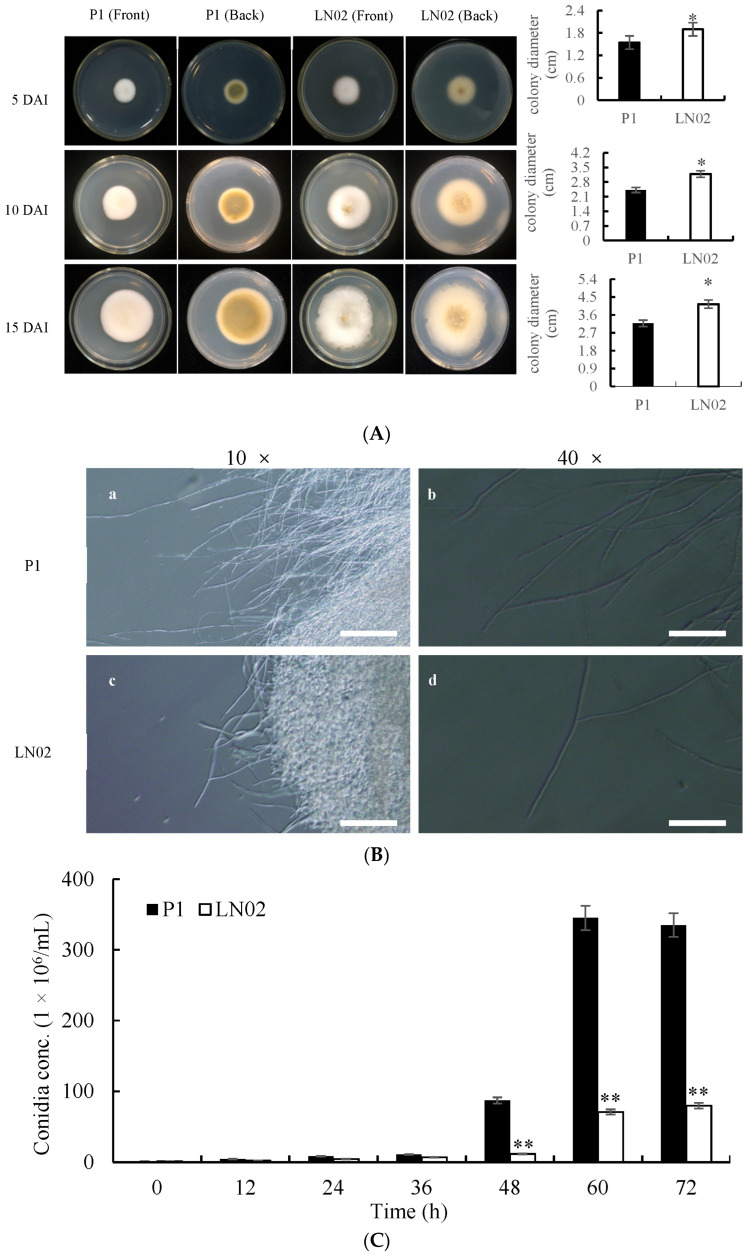
Phenotypic characterization of strain LN02. (**A**) The colonies of strains P1 and LN02 growing on PDA medium for 5, 10, and 15 days after inoculation (DAI), respectively; (**B**) the mycelia of strains P1 and LN02 observed under optical microscope. The mycelia marginal part of strain P1 cultured for 10 days after inoculation (**a**,**b**), and the mycelia marginal part of strain LN02 cultured for 10 days after inoculation (**c**,**d**); (**C**) the conidia concentrations of strains P1 and LN02 in different period of time. Data indicate means ± SD (from at least three independent samples) by Student’s *t*-test (* *p* < 0.05, ** *p* < 0.01). Scale bars: 500 μm in **B**(**a**,**c**), 100 μm in **B**(**b**,**d**).

An albino strain LN02 was isolated from the matured white RFS balls collected from a natural rice field in the Liaoning Province of China by using a single-chlamydospore isolation technique. The albino strain LN02 with a white colony was selected for further investigation of taxonomic status and genomic diversity. On potato dextrose agar (PDA) medium, both albino strain LN02 and normal strain P1 exhibited partial variability in colony color, growth pattern, and conidia morphology. The albino strain LN02 produced less pigment substances than the normal strain P1, per observations from the bottom of a Petri dish. In addition, the colony extension diameter of strain LN02 in Petri dishes was significantly bigger than that of strain P1 (Figure 2A). The mycelial morphology of both strains P1 and LN02 was very similar to each other (Figure 2B). Strain LN02 could produce white to milky white conidia, whereas strain P1 produced light-green to dark-green conidia. Though strain LN02 could produce conidia, the concentration of conidia in strain LN02 was significantly lower than that in strain P1 (Figure 2C).

### 2.2. Alignment of ITS Sequence of RFS Fungus Albino Strain LN02

In order to further confirm the taxonomic status of strain LN02, the total DNA was reacted by PCR with the primers of ITS1 and ITS4. The amplification sequences of about 500 bp were used to conduct BLASTn searches in GenBank (https://www.ncbi.nlm.nih.gov/ (accessed on 15 September 2023)). The ITS1-5.8S-ITS2 partial sequence (Figure S1) of strain LN02 was submitted to the GenBank with accession number OR554273. It had a 100% similarity to the sequences of small ribosomal RNA subunit of *V. virens* strains in GenBank (Table S1). Based on the phenotype characters (Figure 2), ITS sequence alignment (Table S1), and related literature [10,12,13], the albino strain LN02 was considered a white phenotype mutant of *V. virens*.

### 2.3. Genome Sequencing and Assembly of RFS Fungus Albino Strain LN02

The strain LN02 was sequenced to generate a large number of high quality reads after trimming, which corresponded to about 75× sequencing depth. The high-quality reads of strain LN02 were assembled multiple times with different k-mer sizes, the assembled genome length of about 38.81 Mb was considered for the downstream analysis as it yielded an N50 of 6326 Kb. The total assembled genomes of strain LN02 was comprised of seven nuclear chromosomes plus one mitochondrial genome. The whole genome constituted a G + C content of 49.87%. Complete genome assembly statistics of strain LN02 is shown in Table 1. The related genome information for albino strain LN02 was deposited in GenBank with Bio Project ID as PRJNA1002218, BioSample ID as SAMN36835811, and accession numbers as CP135105–CP135112 (accessed on 30 September 2023).

### 2.4. Gene Identification and Annotation of RFS Fungus Albino Strain LN02

The genome size and gene number of each chromosome in strain LN02 are shown in Table 2. About 9339 protein-encoding genes were identified in strain LN02 by gene prediction using GeneMark-ES. However, the genomes of strains UV8b, IPU010, UV_Gvt, UV-FJ-1, JS60-2, and UV2_4G harbored 8426, 6451, 6627, 7164, 8468, and 7444 protein-encoding genes, respectively [14,15,16,17,18,19]. The comparative genomics of different RFS fungal genomes are shown in Table S2.

**Figure 3:**
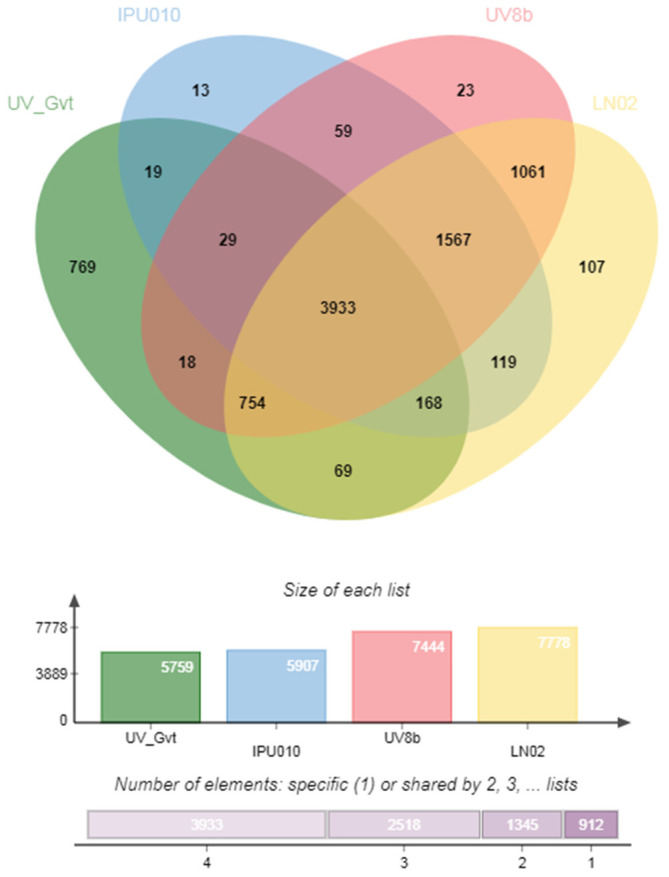
Venn diagram showing common orthologous and unique sets of genes across four RFS fungal strains.

There were 3933 relatively conservative core genes in the genome of strain LN02. Meanwhile, comparative genome analysis of four strains including LN02, UV8b, IPU010, and UV_Gvt revealed the presence of 107 unique genes in strain LN02 (Figure 3). The identified unique protein sequences predicted in the genome of strain LN02 are listed in Table S3. Gene ontology (GO) annotation showed that these unique genes were involved in several biological processes and different cellular components (Table S4). The phylogenetic tree and enrichment analysis of partial unique genes (e.g., cluster 3) are shown in Figure S2. These indicated that the genome in stain LN02 was different from that of other three RFS strains, which reflected the diversity of RFS strains collected from different areas of world. The differences provided a basis for further development of the LN02 strain’s genome.

### 2.5. Functional Annotation of Predicted Proteins in RFS Fungus Albino Strain LN02

By Kyoto Encyclopedia of Genes and Genomes (KEGG) pathway analysis, the predicted genes of strain LN02 genome displayed multiple protein families, which are associated with diverse pathways. In order to correct the analysis results, the genomes of *Fusarium graminearum* [20], *Fusarium oxysporum* [21], and *Trichoderma reesei* [22] were added as the model species, which belonged to Hypocreales. The presence or absence of homologous clusters in different fungal strains are shown in Table 3. A total of 707 proteins in strain LN02 were not in any cluster. It would bring more challenges to study protein functions in strain LN02. The function annotations of the predicted proteins by gene ontology (GO) and clusters of orthologous groups (COG) are shown in Tables S5 and S6, respectively. The pathways were categorized into three types, including biological processes, molecular functions, and cellular components. By GO annotation, about 581 predicted proteins were involved in the biological processes, 46 predicted proteins in the molecular functions, and 10 predicted proteins as the cellular components. It is worth noting that many predicted proteins were involved in the processes of secondary metabolism (Table S5). A total of 4414 predicted proteins were described by COG annotation (Table S6).

### 2.6. BGCs for Secondary Metabolites in RFS Fungus Albino Strain LN02

In most fungi, the genes involved in the biosynthesis of secondary metabolites with the same pathway are generally clustered together. The potential BGCs of secondary metabolites in strain LN02 were analyzed by antiSMASH (antibiotics and secondary metabolite analysis shell) [23,24]. A total of 19 different gene clusters, including five T1PKS, four NRPS, four NRPS-like, two NAPAA and four terpenoid types were predicted in the genome of strain LN02 (Table 4). Region 1.4 was the most similar known cluster for ustilaginoidin biosynthesis, with a similarity of 100% [25]. Region 2.3 was the most similar known cluster for sorbicillinoid biosynthesis, with a similarity of 57% [26].

#### 2.6.1. BGCs for Peptide Compounds

**Figure 4:**
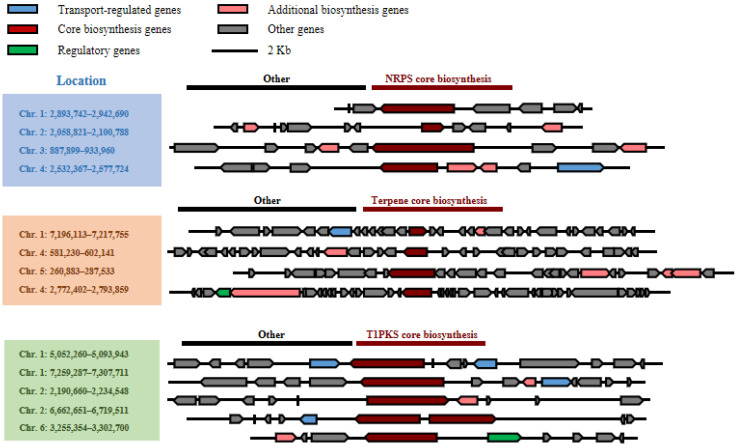
The putative genes and their annotations of the BGCs for biosynthesis of NRPs, terpenoids, and polyketides in albino strain LN02.

Four putative BGCs for non-ribosomal peptide biosynthesis and four putative BGCs for NRP-like fragment biosynthesis were predicted in albino strain LN02 (Table 4 and Figure 4). The NRPS and NRPS-like gene clusters might be silent, as peptide compounds have not been isolated from strain LN02 under laboratory culture conditions. Either the expression of NRPS- and NRPS-like-encoding genes or post-translational modification were unclear in strain LN02.

#### 2.6.2. BGCs for Terpenoid Compounds

Four BGCs for terpenoid biosynthesis were predicted in albino strain LN02 (Table 4 and Figure 4). The gene clusters were predicted to be involved in the biosynthesis of squalestatin S1, asperterpenoid A, gibberellin, and aphidicolin. Unfortunately, no terpenoid metabolites were found in the cultures of strain LN02 under current culture conditions.

#### 2.6.3. BGCs for Polyketide Compounds

Five type I PKS BGCs were deduced from the genome sequences of strain LN02 (Table 4 and Figure 4). Two groups of polyketides, including sorbicillinoids [27,28] and ustilaginoidins [25,29,30,31,32], have been isolated from the normal strains UV8b and P1. Their BGCs were also identified for sorbicillinoids [26] and ustilaginoidins [25,31] in the normal strains. However, there was no report on the previous isolation of sorbicillinoids and ustilaginodins from strain LN02.

### 2.7. Identification of BGCs of Sorbicillinoids and Ustilaginoidins in RFS Fungus Albino Strain LN02

#### 2.7.1. Identification of BGC for Sorbicillinoids

A putative gene cluster located on region 2.3 of chromosome 2 was predicted to be involved in the biosynthesis of sorbicillinoids [26]. By blastP searches for orthologous genes in the National Center for Biotechnology Information (NCBI), six genes *UvSorA*, *UvSorB*, *UvSorR1*, *UvSorR2*, *UvSorT*, and *UvSorC* were found to be conserved in *V. virens*, *Penicillium chrysogenum*, *Trichoderma reesei*, and *Acremonium chrysogenum*. Among these conserved genes, two PKS genes, *UvSorA* and *UvSorB*, showed high homology with its orthologues in other fungi [26].

**Figure 5:**
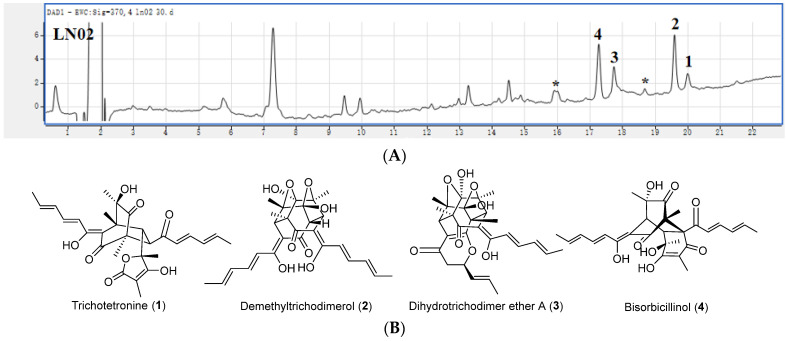
Isolation and identification of sorbicillinoids in albino strain LN02. (**A**) HPLC-DAD analysis (at 370 nm) of the extract from albino strain LN02. (**B**) Structures of four identified sorbicillinoids (**1**–**4**) in strain LN02. (*) indicated the unknown sorbicillinoid.

Four isolated sorbicillinoids were identified by comparing their spectroscopic data with those published in the literature [28], which included trichotetronine (**1**), demethyltrichodimerol (**2**), dihydrotrichodimer ether A (**3**), and bisorbicillinol (**4**) (Figure 5) with their *m*/*z* values of deprotonated peak [M–H]^-^ in HRESIMS spectra and suggested molecular formula shown in Table S7, ^1^H and ^13^C NMR data shown in Table S8, and structures of four identified sorbicillinoids shown in Figure S3. It indicated that the BGC for sorbicillinoid biosynthesis in strain LN02 was not in silent, and the strain LN02 fungus had the potential to produce sobicillinoids.

#### 2.7.2. Identification of BGC for Ustilaginoidins

**Figure 6:**
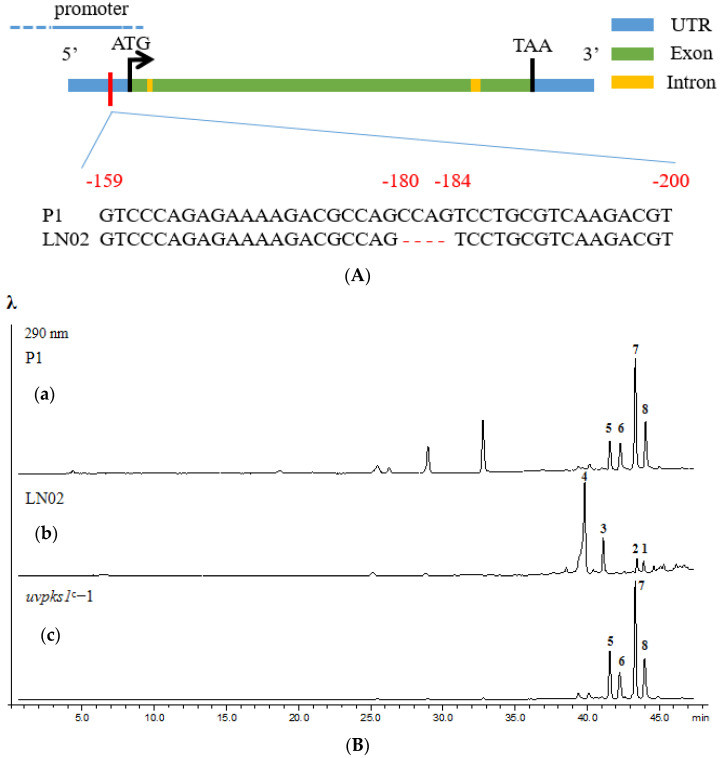

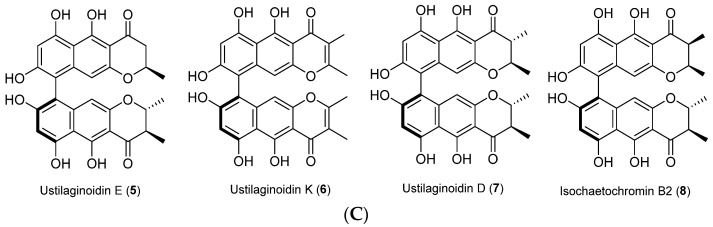
The deletion of four bases in the promoter sequence of PKS-encoding gene *uvpks1* led to ustilaginoidin BGC silencing in strain LN02. (**A**) The deletion of four bases in the promoter sequence of PKS-encoding gene *uvpks1*; (**B**) HPLC-DAD analysis (at 290 nm) of the crude extracts. (**a**) Four main ustilaginoidin peaks (**5**–**8**) were found in the extract of normal strain. (**b**) Ustilaginoidins were not found in the extract of albino strain LN02. However, four main sorbicillinoids (**1**–**4**) were found in the extracts. (**c**) Four main ustilaginoidin peaks (**5**–**8**) were found in the extract of complementary mutant *uvpks1^C^*-1; (**C**) structures of four identified ustilaginoidins (**5**–**8**) in the complementary mutant *uvpks1^C^*-1.

A putative gene cluster located on the region 1.4 of chromosome 1 was predicted to be involved in the biosynthesis of ustilaginoidins [25]. However, ustilaginoidins were not found in EtOAc extract of the cultures of albino strain LN02 (Figure 6B). By blastP searches for orthologous genes in the National Center for Biotechnology Information (NCBI), five genes including *uvpks1* (encoding a non-reducing polyketide synthase), *usgD* (encoding a putative dehydrase), *usgM* (encoding a C-methyltransferase), *usgL* (encoding a laccase), and *usgO* (encoding putative flavin-dependent oxidoreductase) were found in the BGC of ustilaginoidins in strain LN02 [25].

By blastN searches for the sequences of strain LN02 in the NCBI, a four-base (i.e., CCAG) deletion in the promoter sequence of the PKS-encoding gene *uvpks1* was found by comparing with normal strain P1 (Table S9; Figure 6A). The open reading frame (ORF) sequence of *uvpks1* containing a normal native promoter was introduced into the albino strain LN02 by complementation vector (pCBHT) to create the complemented strain, which restored the ability to normal phenotypes such as secretion of the pigment substances into the medium, and re-expression of PKS gene *uvpks1* for ustilaginoidin biosynthesis (Figure S4). Furthermore, it was found that three complementary strains restored the ability to synthesize ustilaginoidins. Four main ustilaginoidins, including ustilaginoidins E (**5**), K (**6**), D (**7**), and isochaetochromin B2 (**8**), were isolated and identified with their structures shown in Figure 6C and Figure S5. Their *m*/*z* values of deprotonated peak [M–H]^−^ in HRESIMS spectra and suggested molecular formula are shown in Table S10, and their ^1^H and ^13^C NMR data are shown in Table S11. It indicated that the BGC for ustilaginoidin biosynthesis in strain LN02 was silent due to the natural deletion of four bases in the promoter sequence of PKS-encoding gene *uvpks1*. The normal *uvpks1* complementary mutant of strain LN02 could restore the ability to synthesize ustilaginoidins.

## 3. Discussion

### 3.1. Taxonomic Status of RFS Fungus Albino Strain LN02

White RFS was first reported in Japan in 1991 [10]. Subsequently, it was also described in China [11,12], and in the United States [13]. Both white and normal RFS strains were commonly considered the same species [10,12,13]. In this study, the phenotype characters including colony and conidia of strain LN02 were observed. It was found that there was no significant difference for the phenotype characters between strains LN02 and P1 (Figure 2). In addition, the ITS1/ITS4 amplicons of strain LN02 had very high sequence similarities to the small subunit ribosomal RNA sequences from *V. virens* Uv-12 (with accession number as MN340266.1) and other conserved RNA sequences (Table S1). Furthermore, the BGCs for secondary metabolites of strain LN02 were very similar to those of other strains of *V. virens* (Table S12). The results in this study support that albino strain LN02 and normal strain P1 belong to the same species. The albino strain LN02 should be a white phenotype mutant of *V. virens*.

### 3.2. Genome Sequencing and Assembly Comparison of LN02 with Other Three V. virens Strains

The complete genome sequence of *V. virens* was first reported by Zhang et al. in 2014 [14], who provided basic analysis on different genomic components related to RFS symptoms. Subsequently, the sequencing efforts by Kumagai et al. focused more on the diverse pathways of mycotoxins produced by RFS fungus [15]. Recently, Indian scientists also provided new insights on the genetic diversity and phylogenetic divergence of RFS fungus [16]. Unfortunately, there was little information on the genome sequencing of albino strains, which limited the further research of white RFS. The albino strain LN02 was selected for whole genome sequencing in this study. The assembled genome length of strain LN02 was 38.81 Mb, a little larger than that of 33.6 Mb and 26.96 Mb of strains IPU010 and UV_Gvt, respectively. We also predicted 9339 protein-encoding genes in strain LN02 (Table S2), whereas only 8426, 6451, and 6627 protein-encoding genes have been reported in strains UV8b, IPU010, and UV_Gvt, respectively. There were more genes in the LN02 strain genome than those in the genomes of strains UV8b, IPU010, and UV_Gvt, and less genes than most of the other sequenced species in ascomycetes [14,15,16].

### 3.3. Functional Analysis of Predicted Genes and Proteins in Albino Strain LN02 Related to Secondary Metabolism

Comparative genomics of four *V. virens* strains revealed that about 107 unique genes were presented in the genome of strain LN02 (Figure 3). GO annotation suggested that these unique genes were involved in several biological processes and different cellular components (Table S5). We speculated that these unique genes might be involved in the phenotypic change processes of strain LN02 with their unclear specific mechanisms.

There were 19 BGCs of secondary metabolites in the genome of strain LN02 which contained five T1PKS, four NRPS, four NRPS-like, two NAPAA and four terpene synthases (Table 4). However, the BGCs of secondary metabolites in different RSF fungal strains were different. There were 49, 22, 24, 21, and 21 BGCs of secondary metabolism predicted in other RFS fungal strains UV8b, IPU010, UV_Gvt, UV-FJ-1, and JS60-2, respectively (Table S12). These enzymes were responsible for the fundamental steps for the backbone biosynthesis of secondary metabolites [33]. The reason for different number of BGCs of secondary metabolites predicted in RFS fungal strains was possibly that the fungal strains were derived from different areas of the world. Their secondary metabolism exhibited a diversity to adapt to different environments. Other reasons included the genome sequencing level and antiSMASH versions used at that time.

Secondary metabolites usually have functions in controlling morphological differentiation and biological fitness in fungi [34]. Three types of secondary metabolites have been reported from the RFS fungus, including the ustiloxins, ustilaginoidins, and sorbicillinoids. Ustiloxins are colorless cyclic peptides which have been reported to have antimitotic activity by inhibiting microtubule assembly and cell skeleton formation of plant and animal cells [35,36,37]. Both ustilaginoidins and sorbicillinoids are colored polyketides. Ustilaginoidins in RFS fungus are 9,9′-linked bis-naphtho-γ-pyrones with a *R* configuration [38], which exhibited a series of biological activities such as cytotoxic, phytotoxic, and antimicrobial activity [29,30]. Many ustilaginoidins were isolated from RFS balls [30], with more than 100 mg/g of ustilaginoidins in the mycelia and chlamydospores [8]. Sorbicillinoids in RFS fungus belong to hexaketide metabolites in which the cyclization has taken place on the carboxylate terminus [39], which also displayed a variety of biological activities [40]. Many sorbicillinoids were found in rice cultures of RFS fungus, but only trace amounts of sorbicillinoids were found in RFS balls [27,28].

Nineteen secondary metabolite BGCs were predicted in the genome of albino strain LN02. This fungal strain will have a great potential in the development and application of secondary metabolites. However, only four sorbicillinoids (**1**–**4**) were identified in the cultures of strain LN02 (Figure 5), and four ustilaginoidins (**5**–**8**) were identified from the normal *uvpks1* complemented mutant of strain LN02 (Figure 6) in this study. Other secondary metabolites should be revealed in detail. Some secondary metabolite BGCs might be in the silent state. In order to unlock these cryptic gene clusters, a variety of strategies could be used, such as one strain many compounds (OSMAC) method, by changing cultivation parameters (i.e., carbon source, nitrogen source, light intensity, ambient pH, shaking, aeration, incubation temperature, redox status, and metal ions) [41,42], global regulation [43,44], epigenetic manipulation [45,46], and gene cluster heterologous expression [47,48].

### 3.4. Deficiency of Ustilaginoidin Synthesis Should Be the Cause of Albinism for Albino Strain LN02

Melanin biosynthesis has been verified in many fungi. It protects fungi in the environment from a range of stresses [49,50,51,52,53]. 1,8-Dihydroxynaphthalene (1,8-DHN) was considered the precursor of melanin biosynthesis, which was mainly controlled by the genes of ALB1, RSY1, and BUF1 [54]. However, neither melanin and its precursors nor related biosynthetic genes were found in RFS fungus.

In this study, ustilaginoidins were not found in albino strain LN02 due to the four-base deletion in the promoter sequence of the PKS gene *uvpks1* (Figure 6A). The complementary mutant of strain LN02 could restore the ability to synthesize ustilaginoidins. The results indicated that deletion of the promoter of *uvpks1* led to BGC silencing and completely eliminating the production of ustilaginoidins. It demonstrates that deficiency of ustilaginoidin biosynthesis is the cause of albinism for RFS albino strain LN02. It also shows that RFS fungus does not belong to a melanotic fungus. Ustilaginoidins may provide RFS fungus protection from environmental stresses, and other physiological and ecological functions need to be studied in detail.

## 4. Materials and Methods

### 4.1. Fungal Strains

The white RFS balls were collected in the Liaoning Province of China in 2019. The white false smut balls were washed with deionized water three times. Then, the clean samples were sterilized with 70% ethanol for 2 min and immersed successively in 1% sodium hypochlorite for 20 min, then rinsed in sterile distilled water three times. Finally, the samples were dried on a sterile absorbent paper. The surface-dried balls were cut into small pieces, and 2–3 pieces were placed on each potato dextrose agar (PDA, potato 200 g/L, dextrose 20 g/L, and agar 20 g/L) plate containing 500 µg/mL of streptomycin sulfate. Inoculated plates were incubated at 28 °C for 7–10 days in darkness, until apparent mycelial growth. The pure cultures were isolated by hyphal tip isolation on PDA plates until the colony morphology was stable and consistent to afford the single colony LN02.

The chlamydospores isolated from the white RFS balls were prepared into chlamydospore suspension at 10^6^ CFU/mL. A little suspension was streaked onto acidified PDA medium at pH 6.0 containing ampicillin (50 μg/mL). The plates were incubated at 28 °C for 15 days to afford the single colony LN02. The normal strain P1 was kindly provided by Wenxian Sun from Department of Plant Pathology, China Agricultural University. Both strains LN02 and P1 were transferred to 50% glycerol-PDB (potato dextrose broth) and were stored at −80 °C at the China Agricultural University.

### 4.2. DNA Isolation

The pure culture of albino strain LN02 was grown in potato dextrose broth (PDB, potato 200 g/L and dextrose 20 g/L) in an incubator shaker at 150 rpm at 28 °C. After the fungus has been cultured for 10 days, the mycelia were harvested for DNA isolation. Genomic DNA was obtained by using cetyl trimethyl ammonium bromide (CTAB) and phenol–chloroform extraction method [55].

### 4.3. ITS Sequencing

The ribosomal DNA was amplified by nested-PCR with primers ITS-1 (5′-TCCGTAGGTGAACCTGCGG-3′) and ITS-4 (5′-TCCTCCGCTTATTGATATGC-3′) [56,57,58]. Replicated amplifications produced single DNA fragments as resolved on 1% agarose gels containing ethidium bromide. The amplicons were purified with a DNA sequencing clean-up kit. The pure PCR products were analyzed by Tsingke Biotech (Tiangen Biotech Co., Ltd., Beijing, China). Each ITS sequence was subjected to BLAST search against the GenBank database, and the strain with the highest similarity was found and downloaded.

### 4.4. Genome Sequencing, Data Processing and Genome Assembly

The genomic DNA was extracted from mycelia of strain LN02 after being grown in PSB for 5 days. The whole genome of strain LN02 was sequenced using the PacBio Sequel platform and Illumina NovaSeq PE150 at the Beijing Novogene Bioinformatics Technology Co., Ltd. (Beijing, China). We first used high-throughput Illumina sequencing technology to perform paired-end sequencing of the genome of strain LN02, and then sequenced the genome with PacBio. The genomic data from the two kinds of sequencing technology were assembled together using HGAP software (SMRT Analysis version 1.4) [59]. Polymerase reads were filtered for quality (eliminate length < 100, quality < 0.80), adapter, and other contamination.

High quality reads (Q > 30) were obtained after trimming the raw data using TrimGalore (v6.0) (https://www.bioinformatics.babraham.ac.uk/projects/trim_galore/ (accessed on 25 July 2020)); unpaired reads were also retained. KmerGenie was employed to estimate the best k-mer size for the assembly. Reads were assembled with assemblers such as SPAdes: St. Petersburg genome assembler (auto k-mer selection) [60], Velvet (*K* = 37) [61], ABySS: Assembly By Short Sequences (*K* within a range of 21–71) [62], MaSuRCA: Maryland Super-Read Celera Assembler (Auto) [63], IDBA-UD: Iterative De Bruijn Graph De Novo Assembler (*K* within a range of 21–71) [64]. Assembled genomes were compared among each other with metrics like N50, number of gaps and number of scaffolds and total length.

### 4.5. Gene Annotation

Protein-encoding genes in the genome of strain LN02 were predicted independently with GeneMark-ES [65]. The genomes among LN02, UV8b (GCA_000687475.1), IPU010 (GCA_000965225.2), and UV_Gvt (GCA_002939685.2) were compared to identify the unique genes in LN02 genome.

### 4.6. GO Annotation

All the predicted proteins of strain LN02 were mapped to reference canonical pathways in Gene Orthology (GO). All proteins were classified under three categories, including biological process, molecular function, and cellular component. The output of GO annotation includes GO enrichment results (https://orthovenn2.bioinfotoolkits.net/home (accessed on 13 March 2021)).

### 4.7. AntiSMASH

According to the method described by Zhu et al. [24], the antiSMASH database was used to search the gene clusters (https://fungismash.secondarymetabolites.org/#!/start (accessed on 18 January)). The potential biosynthetic gene clusters were identified by profile hidden Markov models (pHMMs), and 24 secondary metabolite classes of pHMMs describing the key biosynthetic enzymes were detected by HMMer3 (3.0) software. Further detailed analyses were performed using a pHMM-based approach. The putative product biosynthesis gene clusters between the genomes were obtained.

### 4.8. Complementation Test

The genomic fragment, including the native promoter from strain P1 and CDS sequence of *uvpks1* from strain LN02, was cloned into the pCBHT binary vector, which was then introduced into the protoplasts of strain LN02. The primers used were listed in Table S13. The transgenic strains were grown in the incubator at 28 °C. The vector pCBHT was kindly provided by Jin-Rong Xu (Department of Botany and Plant Pathology, Purdue University, West Lafayette, IN, USA).

### 4.9. Total RNA Extraction and RT-qPCR Analysis

Total RNA was extracted using an RNA prep pure kit (TIANGEN Biotech, Beijing, China, http://www.tiangen.com/en/ (accessed on 21 May 2023)). A 1 μg portion of total RNA was reverse-transcribed by priming with oligo (dT18) in a 20 μg reaction based on the Prime Script Reverse Transcriptase kit (TaKaRa, http://www.takara-bio.com (accessed on 20 March 2023)). The value of *β-actin* and *α-tubulin* mRNA were used as the internal control [66]. The primers in this study were listed in Table S13.

### 4.10. Isolation and Identification of Sorbicillinoids and Ustilaginoids from the Extracts of Fungal Strains

The strain LN02 and complementary strain *uvpks1*^C^-1 were separately grown on PDA medium in Petri dishes at 28 °C for 8 days. Then, three agar plugs (0.5 cm × 0.5 cm) containing mycelia were added into a 1000 mL Erlenmeyer flask containing 300 mL of PDB under aseptic condition and incubated at 28 °C in the dark for 7 days on a rotary shaker (150 rpm). This liquid culture was subsequently used to inoculate the solid rice medium, which was composed of rice and deionized water in a ratio of 1.0:1.1 (*w*/*w*) and autoclaved at 120 °C for 30 min before use. The fermentation was carried out in fifty 1000 mL Erlenmeyer flasks each containing 100 g of rice and 110 mL of water, and incubated at 28 °C for about 2 months before harvest.

The moldy rice cultures were collected and dried at room temperature. The dry cultures were pulverized and extracted with ethyl acetate (EtOAc) at room temperature five times (24 h for each time). The EtOAc extract was concentrated under vacuum at 38 °C on a rotatory evaporator to give a brownish residue. A total of 20 g of EtOAc extract was obtained from the LN02 strain. In total, 15 g of EtOAc extract was obtained from complementary strain *uvpks1*^C^-1. The extract was subjected to repeated vacuum liquid chromatograph over silica gel, column chromatography over Sephadex LH-20, and semi-preparative HPLC, to give pure compounds. High-resolution electrospray ionization mass spectrometry (HRESIMS) spectra were recorded on a LC1260/Q-TOF-MS 6520 instrument (Agilent Technologies, Santa Clara, CA, USA). ^1^H and ^13^C NMR spectra were measured on Bruker Avance 400 NMR spectrometers (Bruker BioSpin, Zürich, Switzerland). ^1^H and ^13^C NMR chemical shifts were expressed in δ (ppm) referring to the inner standard tetramethylsilane (TMS), and coupling constants in Hertz.

According to the method described by Meng et al. [28], four sorbicillinoids (**1**–**4**) were isolated and identified in the EtOAc extract from strain LN02. Similarly, four ustilaginoidins (**5**–**8**) were isolated and identified in the EtOAc extract from complementary strain *uvpks1c*-1 according to the method of Lu et al. [29].

### 4.11. HPLC-DAD Analysis of Sorbicillinoids and Ustilaginoidins from the Extracts of Fungal Strains

HPLC-DAD analysis of the EtOAc extracts was performed on a Shimadzu LC-20A instrument equipping with a SPD-M20A photodiode array detector (Shimadzu Corp., Tokyo, Japan) using an analytical C_18_ column (250 × 4.6 mm i.d., 5µm; Phenomenex Inc., Torrance, CA, USA) eluting with a mixture of MeOH and water (containing 0.02% TFA). A gradient elution program eluting from 10% to 100% MeOH over 45 min was used, and the flow rate was 1.0 mL/min. The column temperature was set at 30 °C. The wavelength was either set at 290 nm for detection of ustilaginoidins [25] or set at 370 nm for detection of sorbicillinoids [42].

### 4.12. Statistical Analysis

All statistical analyses were conducted using Excel 2013 (Microsoft Corp., Redmond, WA, USA).

## 5. Conclusions

In summary, an albino strain LN02 was isolated from the white RFS balls. It was considered a natural albino mutant of *V. virens* by analyzing its phenotypes, ITS conserved sequence, BGCs of secondary metabolism, and main isolated secondary metabolites. The total assembled genome data of the strain LN02 was provided as 38.81 Mb, which was comprised of seven nuclear chromosomes and one mitochondrial genome with 9339 protein-encoding genes. This is the first report of the whole-genome of white false smut fungus. In addition, the genome of strain LN02 encoded 19 gene clusters for biosynthesis of secondary metabolites, mainly including polyketides, terpenoids and NRPs to show its potential in the development and application of secondary metabolites. Four sorbicillinoids were identified from the cultures of albino strain LN02. It was found that the BGC for ustilaginoidin biosynthesis in strain LN02 was in silent due to the natural deletion of four bases in the promoter sequence of PKS-encoding *uvpks1*. The complementary mutant of strain LN02 could restore the ability to synthesize ustilaginoidins. The albino strain LN02 was further confirmed as a white phenotype mutant of *V. virens*, mainly due to the lack of ustilaginoidins. The results should be helpful in search of genetic diversity, secondary metabolites and their functions, fungal pathogenicity, and interactions between albino RFS fungus and rice plants. Furthermore, the physiological and ecological functions of ustilaginoidins such as sporulation, pathogenesis, and protection from environmental stresses in RFS fungus are required for investigation in detail.

## Figures and Tables

**Table 1:** Genome statistics of RFS fungus albino strain LN02.

Genomic Feature	Predicted Quantity/Value
Total sequence length (bp)	38,807,225
Chromosome count	8 (7 + 1)
G + C content (%)	49.87
Number of protein-encoding genes	9339
N50 length	6,326,845
N90 length	3,771,640
Longest length	7,470,426
Shortest length	126,472
Average length	4,850,903
Median length	5,972,438
Depth of sequencing	75×

**Table 2:** The genome size and gene number of each chromosome in strain LN02.

Region	Genome Size	Gene Number
Chr. 1	7,470,426	1937
Chr. 2	6,769,627	1686
Chr. 3	6,326,845	1335
Chr. 4	5,987,177	1628
Chr. 5	5,957,699	1406
Chr. 6	3,771,640	733
Chr. 7	2,397,339	614
Mitochondria	126,472	/
Total	38,807,225	9339

Note: The mitochondria genome was a circular DNA with no protein-encoding-gene to be identified.

**Table 3:** The presence or absence of homologous clusters in 7 fungal strains.

Species/Strain	Protein	Cluster	Singleton	Ref.
RFS fungus strain LN02	9339	7778	707	This study
RFS fungus strain UV8b	8426	7444	874	[14]
RFS fungus strain IPU010	6451	5907	479	[15]
RFS fungus strain UV_Gvt	6627	5759	462	[16]
*Fusarium graminearum*	14,145	10,233	3378	[20]
*Fusarium oxysporum*	16,884	10,903	3630	[21]
*Trichoderma reesei*	9113	7488	1353	[22]

**Table 4:** The putative BGCs predicted in the genome of RFS fungus albino strain LN02 by antiSMASH Fungal Version (v5.0.0).

Region	BGC	Type	Location
Chr. 1	Region 1.1	NPRS	2,893,742–2,942,690
	Region 1.2	T1PKS	5,052,260–5,093,943
	Region 1.3	Terpenoid	7,196,113–7,217,755
	Region 1.4	T1PKS	7,259,287–7,307,711
Chr. 2	Region 2.1	NRPS	2,058,821–2,100,788
	Region 2.2	T1PKS	2,190,660–2,234,548
	Region 2.3	T1PKS	6,662,651–6,719,511
Chr. 3	Region 3.1	NRPS-like	448,585–489,501
	Region 3.2	NRPS-like	3,797,799–3,841,987
Chr. 4	Region 4.1	Terpenoid	581,230–602,141
	Region 4.2	NRPS	887,899–933,960
	Region 4.3	NRPS	2,532,367–2,577,724
	Region 4.4	NAPAA	4,127,531–4,161,532
	Region 4.5	NRPS-like	5,446,369–5,489,759
Chr. 5	Region 5.1	Terpenoid	260,883–287,533
Chr. 6	Region 6.1	Terpenoid	2,772,402–2,793,859
	Region 6.2	NAPAA	3,191,042–3,224,944
	Region 6.3	T1PKS	3,255,354–3,302,700
Chr. 7	Region 7.1	NRPS-like	189,840–230,870

Note: NRPS: nonribosomal peptide synthetase; T1PKS: type I polyketide synthase; Terpenoid: biosynthesis of terpenoids; NRPS-like: NRPS-like fragment; NAPAA: non-alpha poly-amino acids like e-polylysin. No secondary metabolism region was found in mitochondrial genome.

## Data Availability

The data presented in this study are available on request from the corresponding author.

## Supplementary Materials

The supporting information can be downloaded at: https://www.mdpi.com/article/10.3390/ijms242015196/s1.
