# Supplementary material for: A Genome-Wide Comparison of Rice False Smut Fungus Villosiclava virens Albino Strain LN02 Reveals the Genetic Diversity of Secondary Metabolites and the Cause of Albinism
Source: Int J Mol Sci. 2023 Oct 15;24(20):15196. doi: 10.3390/ijms242015196 (PMC10607355; doi:10.3390/ijms242015196)
Supplement: Supplementary file 1 [file ijms-24-15196-s001.zip › ijms-2640943-supplementary.pdf]

Supplementary Materials:

A Genome-Wide Comparison of Rice False Smut Fungus *Villosiclava virens* Albino Strain LN02 Reveals the Genetic Diversity of Secondary Metabolites and the Cause of Albinism

Mengyao Xue [1], Siji Zhao [1], Gan Gu [1], Dan Xu [1], Xuping Zhang [1], Xuwen Hou [1], Jiankun Miao [2], Hai Dong [2], Dongwei Hu [3], Daowan Lai [1] and Ligang Zhou [1,*]

[1] Department of Plant Pathology, College of Plant Protection, China Agricultural University, Beijing 100193, China; mengyaoxue@cau.edu.cn (M.X.); sijizhao@cau.edu.cn (S.Z.) gangu@cau.edu.cn (G.G.); cauxudan@cau.edu.cn (D.X.); zhangxuping5@cau.edu.cn (X.Z.); xwhou@cau.edu.cn (X.H.); dwlai@cau.edu.cn (D.L.)

[2] Institute of Plant Protection, Liaoning Academy of Agricultural Science, Shenyang 110161, China; mjkkx@163.com (J.M.); lnsydh@163.com (H.D.)

[3] Biotechnology Institute of Zhejiang University, Hangzhou 310058, China; hudw@zju.edu.cn (D.H.)

* Correspondence: lgzhou@cau.edu.cn (L.Z.)

Contents

Figure S1. The ITS1-5.8S-ITS2 partial sequences (504 bp) of albino strain LN02. 3

Table S1. Alignment of the ITS sequence of albino strain LN02 with the ITS-sequences of other fungal strains in GenBank. 4

Table S2. Comparative genomics of RFS fungal strains. 6

Table S3. List of unique protein sequences predicted in the genome of strain LN02.. 7

Table S4. Gene ontology (GO) annotation of unique genes predicted in the genome of strain LN02.............. 23

Figure S2. The phylogenetic tree and enrichment analysis of partial unique genes (e.g., cluster 3) in strain LN02. 24

Table S5. Gene ontology (GO) annotation of predicted proteins in strain LN02 genome. 25

Table S6. Clusters of orthologous groups (COG) annotation of predicted proteins in strain LN02 genome..... 31

Table S7. The deprotonated peak [M-H]$^-$ (*m/z*) in HRESIMS spectra and molecular formula of the sorbicillinoids isolated from albino strain LN02. 252

Table S8. ^{1}H NMR and ^{13}C NMR data of the sorbicillinoids isolated from albino strain LN02......................... 253

Figure S3. Structures of four identified sorbicillinoids (**1**–**4**) with carbon atoms numbered in strain LN02... 255

Table S9. Sequences of the cloned gene *uvpks1* from *V. virens* (*U. virens*) P1, *uvpks1* promoter from *V. virens* (*U. virens*) P1, and *uvpks1* promoter from *V. virens* (*U. virens*) LN02. 256

Figure S4. Complementation of normal *uvpks1* in albino strain LN02 restored to normal phenotypes. 259

Table S10. The deprotonated peak [M-H]$^-$ (*m/z*) in HRESIMS spectra and molecular formula of the ustilaginoidins isolated from the in the complementary strain *uvpks1*^{C}-1 of albino strain LN02. 260

Table S11. ^{1}H and ^{13}C NMR data of the ustilaginoidins isolated from the complementary mutant *uvpks1*^{C}-1 of albino strain LN02. 261

Figure S5. Structures of four identified ustilaginoidins (**5**–**8**) with the carbon atoms numbered in the complementary mutant *uvpks1*^{C}-1 of strain LN02. 263

Table S12. Putative number of secondary metabolite BGCs in six RFS fungal strains by antiSMASH. 264

Table S13. The primers used in this study. 265

GGGCGTTTTACGGGCAGTGGCCGCGCCGCGCTCCAAGTGCGAGGATAACTGAATTACTACGCAGAGGAGGCTGCGACGAGACCGCCGATTCATTTCGG
GGGCGGCCCGCCCGGGCTGGCGGGCAGGGCCGATCCCCAACACCAAGCGCAAGACAGAGCTTGAGGGTTGAAATGACGCTCGAACAGGCATGCCCGC
CAGAATACTGGCGGGCGCAATGTGCGTTCAAAGATTCGATGATTCACTGAATTCTGCAATTCACATTACTTATCGCATTTCGCTGCGTTCTTCATCGATGC
CAGAACCAAGAGATCCGTTGTTGAAAGTTTTGATTCATTTTGATTTGCAAAAATCCACTCAGACATGCATTGGAAAACACAAGAGTTTTTTTGGTTGTAT
CCTCCGGCGGGCGCCTGGTTCCGGGTGCCCTCCCCCTCCCAGGCGCGAGGGCGTCGGGGGGGAGGCGGCTGCCCGGGGCTTGAAAGCCCGCCGAAGCA
ACGGCGTAGG

Figure S1. The ITS1-5.8S-ITS2 partial sequences (504 bp) of albino strain LN02.

Table S1. Alignment of the ITS sequence of albino strain LN02 with the ITS-sequences of other fungal strains in GenBank.

Description	Total Score	Query Cover (%)	E-value	Per. Ident (%)	Accession No.
Ustilaginoidea virens isolate Uv-12 small subunit ribosomal RNA gene, partial sequence; internal transcribed spacer 1, 5.8S ribosomal RNA gene, and internal transcribed spacer 2, complete sequence; and large subunit ribosomal RNA gene, partial sequence.	931	100	0.0	100	MN340266.1
Ustilaginoidea virens isolate Uv-9 small subunit ribosomal RNA gene, partial sequence; internal transcribed spacer 1 and 5.8S ribosomal RNA gene, complete sequence; and internal transcribed spacer 2, partial sequence.	931	100	0.0	100	MN340263.1
Ustilaginoidea virens isolate Uv_18 internal transcribed spacer 1, partial sequence; 5.8S ribosomal RNA gene and internal transcribed spacer 2, complete sequence; and large subunit ribosomal RNA gene, partial sequence.	931	100	0.0	100	MN218702.1
Ustilaginoidea virens isolate Uv_17 internal transcribed spacer 1, partial sequence; 5.8S ribosomal RNA gene and internal transcribed spacer 2, complete sequence; and large subunit ribosomal RNA gene, partial sequence.	931	100	0.0	100	MN218701.1
Ustilaginoidea virens isolate Uv_15 internal transcribed spacer 1, partial sequence; 5.8S ribosomal RNA gene, complete sequence; and internal transcribed spacer 2, partial sequence.	931	100	0.0	100	MN218700.1
Ustilaginoidea virens isolate Uv_14 internal transcribed spacer 1, partial sequence; 5.8S ribosomal RNA gene and internal transcribed spacer 2, complete sequence; and large subunit ribosomal RNA gene, partial sequence	931	100	0.0	100	MN218699.1
Ustilaginoidea virens isolate Uv-76-AYJ-TN-15 small subunit ribosomal RNA gene, partial sequence; internal transcribed spacer 1 and 5.8S ribosomal RNA gene, complete sequence; and internal transcribed spacer 2, partial sequence.	931	100	0.0	100	MN116617.1
Ustilaginoidea virens isolate Uv-75-MDV-TG-8 small subunit ribosomal RNA gene, partial sequence; internal transcribed spacer 1, 5.8S ribosomal RNA gene, and internal transcribed spacer 2, complete sequence; and large subunit ribosomal RNA gene, partial sequence.	931	100	0.0	100	MN116616.1

Ustilaginoidea virens isolate Uv-64-GVT-5-KA-18 small subunit ribosomal RNA gene, partial sequence; internal transcribed spacer 1, 5.8S ribosomal RNA gene, and internal transcribed spacer 2, complete sequence; and large subunit ribosomal RNA gene, partial sequence.	931	100	0.0	100	MN116605.1
Ustilaginoidea virens isolate Uv-61-KST-KA-15 small subunit ribosomal RNA gene, partial sequence; internal transcribed spacer 1, 5.8S ribosomal RNA gene, and internal transcribed spacer 2, complete sequence; and large subunit ribosomal RNA gene, partial sequence.	931	100	0.0	100	MN116602.1

Table S2. Comparative genomics of RFS fungal strains.

Genomic Feature	LN02	UV8b	IPU010	UV_Gvt	UV-FJ-1	JS60-2	UV2_4G
Genome size (Mb)	38.81	39.40	33.57	26.97	37.41	38.02	35.9
Coverage (X)	75	142	107	75	148	216	10
GC content (%)	49.9	49.9	51.3	54.9	49.71	/	49
Total number of genes	9339	8426	6451	6627	7164	8468	7444
N50 Length	6,326,845	4,262	52,998	15,534	65,280	6,315,893	700,296
Fungal specific transcription factor	99	116	23	26	/	/	/
Zinc finger transcription factor	14	25	3	2	/	/	/
Major facilitator family (MFS)	22	80	26	73	/	/	/
Protein kinase	229	229	221	227	/	/	/
Subtilisin	12	12	10	4	/	/	/
Aspartic protease	20	8	9	10	/	/	/
Lipase	24	39	39	42	/	/	/
Pectinesterase	0	1	1	1	/	/	/
Glycoside hydrolase	9	14	19	14	/	/	/
Dehydrogenase	154	146	163	161	/	/	/
Cutinase	0	8	6	7	/	/	/
Effector protein	2	2	1	2	/	/	/
Pectin lyase	0	0	2	1	/	/	/
Pectate lyase	0	0	0	0	/	/	/
Polyketide synthase	12	11	2	4	/	/	/
Geranylgeranyl diphsphate synthase	0	1	0	0	/	/	/
Ref.	This study	[14]	[15]	[16]	[17]	[18]	[19]

Note: “/” indicated that the data were not given in the article.

Table S3. List of unique protein sequences predicted in the genome of strain LN02.

>LN02|LN02_06842

MPAFCNLTALLLSLAITKAQGGSAGCGKTHDFVGHTREFSIQSSGGLRTYRIHLPLSYDSKTAKPL
LIAYHGHGNNPDKFELQTNFSNETVNPDMIVVYPAGLDKAWQGPSYARKGVSDKVFTTDLVN
HIKSDYCVKESRVYATGHSNGGGFVGTLACSPDHGGQFAAFAPISGAFYTDVKGNQDCHPARS
PLPMFEVHGTGDKTIPYAPTKDGSGGPLPSVADWVRRWSLRNKCDAPQEKDLGNGVHDVRYK
CQGVADGLEHIKVDGMGHPWPGPDSQLPDVSLRVVGFLNKHTKP*

>LN02|LN02_08665

MTEACALSSLVSRISSEKQWKFQTPHQRHKGPFKSVAKQPVLPSEKINFRFRTRLIYATATKASFD
FPNVLHLQICPSIIDRMIDRLLDLLLTSWRDRVGRYFPEWNLPPCLILKMCKKNWDKEFEVEKST
YKALEIIQGTRIPNFYGELKYNGKKAILISDIGGVCLGDPAGAVLEIPDFLDLMKEALTDLGRFHIQ
PDDIKLNNFRLVNGRIMVIDFEMVLDKFDTNEDCAREILGLTYWLGRQYEGRQSCHLADGLITQ
VK*

>LN02|LN02_08688

MAEACKLSSIVCRLASEKEWIDCLPSQRHEGPFESVAKQRLLPSETINFRFRTRLIYATATINYDYP
NVLRLQVQSSIIDRIMDRLLGHLPTSWRDRVSRFFPEWSLPSRLVLKVCKENWDEEFKIEKSTYKA
LESLQGSRIPNFYGELKYNGKKAILISDIGGVCLSTPEGALLKLSDVLNLVKDALIDLFRFGIEPCDI
KLDNFRLVDGRIMPIDFEMVDHNRQTDEKRAEAVLDSAYFLGDQYEKQRSCYLGDGLIKQVK*

>LN02|LN02_00001

MNKPEEKEGKAEKLKRLPAAYFKQFIDAIIRRLENAGVRRLIEAIARPIVTSIRLQPNGDNNNDN
DNNNNNKNNNEDNNNNNNISKDNGNNTDGGNRPDERLKGTTSDFDPLRSLGPVGPNQSYGK
RKQLLALPGHSATLEDVGLIGPNLARELRTSSNRLQATGNIEKPLNRNFSSQRAGNIITFDAKFKR
VLEYNLYKKMKKINKSKVITFGTNSYRDLTRSDLYYDLSTFFLMTNLKPG*

>LN02|LN02_00007

MPSGMLLNRGLNTRPSPLLVLLEDFNTPYEEFKVKEILAIKNA*

>LN02|LN02_00013

MRLIKTLTIEVTNKNNIPFYVLNSSIPFFYSIIDIDRIGIKLNNINNLLI*

>LN02|LN02_00026

MNQLRALNIAKFIENPSIASSFNDLDPGFSVVLIEAAIPNTA*

>LN02|LN02_00129

MLWSRLPSYLWSIELKKDFLEITYYHLPDLTSFRAIGAAVEVLIPPEKRVISYKLAPRTESGPISSGLE
GTFNFQPPSNLEGVNID*

>LN02|LN02_00136

MAFNFTHYIVEKLITSDATTVALYNRLAFLIYTYEKDYIIDDNLWELFHEDYSE*

>LN02|LN02_00142

MHYHLPDLTSFRAIGAAIEVLIPPEKRVISYKLAPQTESGRLLATLGNGTYLVYIPYRQVVTKTSFIT
FTYQNKEGVNID*

>LN02|LN02_00178

MELKKDFPEITHYYLPDLTSFRAIGAAVEVLIPLEKRVISYKLAPRTESGPILNGLEGKFDFLLSSILE
GVNADQITLEEPI*

>LN02|LN02_00179

MASQNVPTNNPGHNNPGHNNPGYQVPNTAEVEESNNNQEIAQLREDQ*

>LN02|LN02_00195

MKNRKNSEKPDENLPYGPEALYRSAVRAGSPIPVCRKGAV*

>LN02|LN02_00205

MAWGAIYRTGRSPIIIMERDEQSARKGFTARSYQKALSEGLLPIFNGIRLF*

>LN02|LN02_00227

MASQNVPTADNPSYTAEKQALIIQLRALNIAKFIENPSIASSFNDLEQALLLLLIKNNYTKRPAAA
INWCTNPESAFMLLTK*

>LN02|LN02_00622

MAESGSYKVPESDTEGLVSRQNNDTQATQATNKKPTKATKAGPSTRTTITKDNDDSEANIMEID
QPSTPIKYKGKEPIYITPPIITITNQISDRAIKELAKTANRFKESQKLKSPYNFDQ*

>LN02|LN02_00623

MDDFIIPKVISFKSKCRLLGLPNNTFARAFQIMLIGDTKTYYFNNIKRIRDTLTFNDIAPNRFKFTLK
DDR*

>LN02|LN02_01072

MPYGPVKFRLTVVKPYNYDLNSDPTTDDPEDIVYAEGPARPSRLEEGKITTLGDPFKQLDRIEIEV
L*

>LN02|LN02_01152

MVIQNTIGNILDTDSGLERGNFGHISTILKKWLKIMKNRKNSEKPDEKSVCPTMPSAV*

>LN02|LN02_01160

MHYYLPDLTSFRAISAAVEVLIPPEKRVISYKLAPQTESGPILNGLEGKFDFLSFSILEGVNAD*

>LN02|LN02_01327

MEVVKLLYGIAEVGTYWWAIYFRYHREKLGMTISTYDPYLLISAGNDCTNDTNVPDRTGDTICF
GIVGMQTNNTLGLSDNTFFQ*

>LN02|LN02_01328

MEVVKLLYGIAEVGTYWWAIYFRYHREKLGMTISTYDPYLLISAGNDCTNDTNVPDRTGDTICF
GIVGMQTNNTLGLSDNTFFQ*

>LN02|LN02_01538

MLGPILSSLEGTFNFQPPSNLEGVNIDQIALEEPIWKSPRLDMPISSKTDGIGLVEGTGLL*

>LN02|LN02_01539

MPYGLVKFRLIVVKPYNHDPNDAEDTVYTEGPARPGRLEVRIPAIAAPLAAITPAAATYLSLATG
PN*

>LN02|LN02_01585

MAESGSSTYKVPESDTEADTNAQLQLDMDDIKSQMNILTDPTEARPSTKTTTTKDNDNEADVIEI
DQPSTPIKYKGKEPIYTTPPTTITNQISDGAIKELAKTANRFKESQKLKSPYNFDQ*

>LN02|LN02_01663

MVVKPYNYDFNNNPTTDDPEDIVYTEGPARPGRLELGYRHYG*

>LN02|LN02_01940

MTNNWHFAANIESFAGSDILMRFCCLSYKLINRYPYLVKDNIVVDRPS*

>LN02|LN02_01974

MEITHYYLPDLTSFRAISAAIEVLIPPEKRVISHKLAPRTESGRLLATLGNGTYLVYIPYRRVVTKTSF
ITFTYQNEGIGPILSGLEGTFDFQPPSNLEGVNIDQIALEEPI*

>LN02|LN02_02038

MVELGSYKVPESDTKATNKKPTKATKAGLSTKTTTKNDNSEADVMEIDQPSTPIKYKGKEPIYITP
LITITN*

>LN02|LN02_02043

MVLNRSLLPFEELKKDFPEITHYHLPDLTSFRAISNGTYLVYIPHRRIVTKTSFITFTYQNKGIGPISS
GLEGTFDFQPPSNLEGVNIDQITLEEPIWKSPRLNIPISSKTDGIGLSESQSPIYTDFLRLSDTTDSAFS
PGFLVNTAFRYSKTLPILEYLKFESPDIASNKRS*

>LN02|LN02_02076

MPYSPIKFRLTVVKPYNHDLNSDPTANNAEDIVYTERPACPGRMEVRISAVAAPLAAITPAAVTY
LPLATGSD*

>LN02|LN02_02313

MPNSWKEVLYNPHKDSWVKALFSEFEQLIDLNIFQFIPKASIPTSRKILRNRPVFRIKKDANNNPV
KYKARLVVKGFMQVSGQDFTETYASTSIPPI*

>LN02|LN02_02314

MPSPNKTARIHGLSTDYPYSETYIMQMLYGPVKFRLIVVKPYNHDPDRNTDPEDIVYPEGLARPG
RLEVRIPAAAAPLAAMILVAAIYPSLVISPD*

>LN02|LN02_02315

MPNSWKEVLYNPYKDSWVKALFSEFEQLIDLNIFQFILKASIPTSRKILRNRPIFRVKKDANNNPV
KYKARLVVKGFMQISRQDFMETYASTSIPPI*

>LN02|LN02_02317

MVIQNTVGNLERGNFGHISTILKKWLKIMKNRKNSEKSDEKSVCPTVPSLCPISYPVHTQPSPVAY
SGVPDAL*

>LN02|LN02_02319

MRLMKTLTIEITNKKHIVNFSAGSATSSSILNILMIFDEAITNYTPNIQRIYANGHYRRHNNIIPERL
KHEDHDHRQEYAYSLA*

>LN02|LN02_02889

MVLNCSSLPFEELKKDFPEITHYHLPDLTSFRAIGAAVEVLIPLEKRVISHKLAPRTESGRLLATLG
NGTYLVYIPYRRVVTKTSFITFTYQNEGIGPILSGLKGTFDFQPPSNLEGVNIDQIALEEPI*

>LN02|LN02_03024

MGAKRPRNWSLRLHSGAQAARTCAPTAALGNAPPVLVAGDHQKTNSLQRRPPRRWQATPGE
R*

>LN02|LN02_03025

MGAKRPRNWSLRLHSGAQAARTCAPTAALGNAPPVLVAGDHQKTNSLQRRPPRRWQATPGE
R*

>LN02|LN02_03026

MGAKRPRNWSLRLHSGAQAARTCAPTAALGNAPPVLVAGDHQKTNSLQRRPPRRWQATPGE
R*

>LN02|LN02_03027

MGAKRPRNWSLRLHSGAQAARTCAPTAALGNAPPVLVAGDHQKTNSLQRRPPRRWQATPGE
R*

>LN02|LN02_03028

MVIQLIRIHARTRRYRPHVQMDKNRLRLSVLAGDLSAAQEARRGVSRITGGDDDDEYEDDEIAS
ASARAETVQEDASGEVPSEFLERDAIEGESPVWLDAEPL*

>LN02|LN02_03029

MNAAIPPHVQMDKNRLRLSVLAGDLSAAQEARRGVSRITGGDDDDEYEDDEIASASARAETVQ
GRPGRTNVRADGGPGER*

>LN02|LN02_03137

MASQNVPTNNPGYQVPDTAEVEESNNDQEIAQLREDQQEIRKEFTVSNRSSSPFEELKKDFPEIIH
YYLPDLTSFRAIGAAVEVLIPPEKRVISHKLAPRTESEGVNIDQIALEEPIWKSPRLDMPISSKTDAY
GSTTISGPTTTNGPATTSGPTTNWPAIALCNIS*

>LN02|LN02_03138

MPYGPVKFRLTVVKPYNHDLNSDLAADDPEDIVYDEGPACPGRLEIGYIIMLGNEEDNPMDNAF
LLTESADGNTLKIASDTLQATNINIPRYAVTSGIRQAPNIDKTAAPNTRYHES*

>LN02|LN02_03455

MASQNIPTADNLSYTAEKQALMIQLRALNIAKFIEEPSIATSPGFSVVLIEAAIPNTAQPP*

>LN02|LN02_03461

MTISTYDPCLLISTSDTDRTGDTDYTNVPDRTGDTSDTVCFSIVGMQTDNTLGLSDNAFF*

>LN02|LN02_03528

MLKAMREVEWISNLFEEIQVDIQRPIPLYCDNQGSISNANNPNQHARTKHTLLKFRY*

>LN02|LN02_03529

MVIQNTVGNVLDTDSGLERGNFGHISTILKKWLKIMKNRKNSEKPDEKSVCPTVPNILPNAYPTK
PSGL*

>LN02|LN02_03537

MATNTDLGNPANFQLLGSKDWFKWISIIEKFAVNENIWAYINPSVEEPNRPTL*

>LN02|LN02_03539

MASQNIPANNLGHNNPGHNNPGYQVPDTAEALMIQLRVLNIAKFIEDPSIASKFNDLDILTYQT
YSRPTYTLPSIN*

>LN02|LN02_03590

MATNTDLWNPANFQLLGNELYISHPSTTVIPVPTTQPYIPSPTLPSTSIPTSE*

>LN02|LN02_03591

MVIQNTIGNILDTDSGLERGNFGHISTILKKRLKTMKNRKNSEKPDEKSSCPTVPSLCLISYPVPTQ
PSSVAYSGVPDAL*

>LN02|LN02_03603

MAWGAIYRTGRSPIIIIERDEQSARNRFTARSYQKALSKGLLPIFNGIRLF*

>LN02|LN02_03605

MASQNVPTADNPSYTAEVEESDNNQEIAQLREDQQEMRKEFIDLLEEQRIKGSSVVRATTHLAK
VKGAATNSNSSKPTSNNTKKRVISYKLAPRTESGRLLAILGNGTYLVYILYRRVVTKTSFITFTYQN
EGIGPILSGLEGTFNFQPPSNLEGVNID*

>LN02|LN02_03607

MAYRHREEGAVEAAEIIDLDGGNRDYVLAATLGNIWNAIISLVDSIKRINRINPASNSNSNLIITN
NLEKVTDYPTYTAKNLPQNNN*

>LN02|LN02_03622

MDTIGHFWTYRLTNSLTHRLTNRTSIPTSRKILRNRPIFRVKKDANNNPVKYKARLVIKGFMQEIE
QIDFIGAFLNSELLETIYMEIPASFLEFTESLLSPNPALWSDLQR*

>LN02|LN02_03659

MVINRSLSPFEELKKDFPEITHYYLSDLTSFRAIGAAVEVLIPPEKRVISHKLAPRTESGRLLATLGN
GTYLIYIPHRRVVTKTSFITFTYQNEGIGPISSGLEGTFDFQPPSNLEGVNIDQIALEEPIWKSLRLN
MPISSKTDAYGSTTISGPTTTSGPTTTSGPTTALCNISQQFAVFRFQIASA*

>LN02|LN02_03664

MAESGSYKVPESDTEGLVSRQNNDSQATQAIQATNKKSTKATEAGPSTKTTTKDNDSKANVMEI
DQPSMPIKYKGKEPIYTTPPTTITNQISDGAIKELAKTANRFKESQKLKSPYNFD*

>LN02|LN02_03758

MVIQNTVGNVLDTDSGLERGNFGHISTILKKWLKIMKNRKNSEKPDENGL*

>LN02|LN02_03759

MEAPYITNIPKEPAIWRELRKHPKREEFIATAKEEYDKVTNREIKCQKFETFRRQLGLVVLKGSRD
TRQSHDIRRDIKEMPYKEDNKDTH*

>LN02|LN02_03760

MLYSPIKFRLTVIKPYNHDLNSNPTTDDPENIVYDEGPARPGRLKPGYRRYGQYHYH*

>LN02|LN02_04060

MYEDIDELYISHPSTTIIPIPTTQPSMPSLTLPSTFIPTSGI*

>LN02|LN02_04076

MWNAITSLVDSIKCINRTNPTPNPNLNFITINNLEKVTDHLTYTAKNLP*

>LN02|LN02_04115

MALTAPIGLISRQNNDTQPNQAINKKPTKATKAGPSTKTTTTKDNNNEANVIEIDQPSTPIKYKG
KEPIYITPLITITNQISDRAIKELAKTANRFKESQKLKSPYNFNQ*

>LN02|LN02_04123

MTISTYDPYLLISAGNNDTDYTDAPDSQDEAITNRTPNIQRIYTNGYYRRHNNVMPERSKHKDH
DYR*

>LN02|LN02_04177

MASQNIPTDNLSYTAEKQALMIQLRALNIAKFIEEPSITSKFNDSEQALLLLLIKNSPISNGLEGKF
DFLLSSILEGVNADQITLEEPIWKSPRLNMPISGLAEGTGLP*

>LN02|LN02_04179

MLERGNFGHISTILKKWLKIMKTRKNSEKPDEKSVCPTVPKMRRLLWALWPTVTGRCGRMWVA
VPVL*

>LN02|LN02_04180

MKNRKNSEKPDEKSVCPTVPKMRRLLWALWPTVTGRCGRLWGAVPVL*

>LN02|LN02_04185

MALYNRLAFLIYTYKKDCIIDDDLWELFHEDYRSSVARATTHLAKVKGAATNSNSSKPASNNTS
KGGKNTTTNANKKKQGSSTQ*

>LN02|LN02_04240

MANQLLNSTIMELKKDFPEITYYHLPDLTSFRAISVAVEVLIPPEKRVISHKLAPRTESGRLLATLG
NGTYLVYIPYRWVVIKTSFITFTYQNEGIGPISSSLEGKFDFLSSSILEGINID*

>LN02|LN02_04568

MVIQNTVGNVLDTDSGLKRGNFGHISTILKKWLEIMKNRKNSEKPDEKSVCPTVPNILPNAYPTK
PSGL*

>LN02|LN02_04806

MATNTDLGNPANFQLLGSKDWFKWISIIEKFAVNENIWAYINPSVEEPNRPTL*

>LN02|LN02_04836

MLIKLYARCKFEIFCRQFRLVTLKGSYDTGLQSHDINLSGKEEPQMDQKTP*

>LN02|LN02_04845

MKNRKNSEKPDEKSVCPTVPSPISSGLEEDFHKGQNIGSEGVSSSKEPAILLDDLESIPDPTNSTNL
TSITNTNAIPDDLESMPDSTNITNNNC*

>LN02|LN02_04848

MITYHQAYQYYNVPRYYGKPLMQLENALQSGKLMMSSLPVTDLIPGLFTNYHYIVKPYIYDLDK
DPIETDPIEIVPTEAPARHNSINTPARHNSINTPARYNSINTPARHNSINTPARPNIINTPARPNRT
NTPACSNRLEVRIPAIAALFNVIVNEE*

>LN02|LN02_04926

MAESGSSSYKVPESDTKADTNVQLQLDMDDMKSQMNILTDLIQGLVSRQNNDSQATQATQAT
NKKPTKATEAGPSTKTTTKDDDSEADVMEID*

>LN02|LN02_04939

MPYNYDLNSNPTTDNPEDIVYNEGPARPGRLELGYRYYG*

>LN02|LN02_04942

MDDFFILKMYYFNNIECIYDTFTFDDMVRKVKSYFENESIEQFYLYY*

>LN02|LN02_04961

MTNNWHFAANIESFASSDILIRFYYLSYKLIDKYPYLIKDDIVVDRPS*

>LN02|LN02_04965

MDSSRPLLPTPPFLLLPEPLQKYAKFINKNWIDANEHINPQNATDTDLYNCLIYRYNAYDIGEDM
DDDLWDIFCDDFSE*

>LN02|LN02_04981

MTISTYDPYLLISAGNNNTNRTGDTSDAIYFGIMGQSKKITTIDKNAPTA*

>LN02|LN02_04982

MEKRVISYKLAPRTESGPILNSLEGKFDFLPSSILEGVNAD*

>LN02|LN02_05200

MTSIPTSRKILRNQPVFRVKKDANNNPVKYKARLIVKGFMQVSRQDFTETYASTSIPPT*

>LN02|LN02_05203

MIQLRVLNIAKFIEDPSIASKFNDSDKPANNNTSKSGKNTTTNANKKK*

>LN02|LN02_05517

MASQNVPTNNPGHNNPGHNNPGYQVPNTAEVEESNNNQEIAQLREDQ*

>LN02|LN02_05532

MKNRKNSEKPDEKSVCLTVPKMRRLLWALWPTVTGRCGRMWVAVPVP*

>LN02|LN02_05725

MTISTYNPCLLISAGNNDTNRTSDTDRTNIPNRDTTTCFGIVGMQTNNTLGLSDNTFF*

>LN02|LN02_05936

MPNSWKETSIPTSRKILRNRPIFRVKKNANNNPVKYKARLVIKGFMQVSGQDFMETYASTSILLI*

>LN02|LN02_05942

MKNRKNSEKPDEKSVCPTMPIIHIGYNKGMRSFIIENGSIRK*

>LN02|LN02_06165

MAESGSYKVPESDTEATQATNKKPIKATNAGPSTKTTTTKDDDSEADVIEIDQPFTPIKYKEEAYL
LLVAQYCHSLEVQRAHLYPTFHTLTFTGYKGTLEAFNSEFNSYISRLRILGSKIDSFD*

>LN02|LN02_06171

MVIQNTVGNVPDTDSGLERGNFGHISTILKKWLKIMKNRKNSEKPDEKSVCPISYPVHTQPSPVA
YSGVPDAL*

>LN02|LN02_06280

MHLYTQTQHLYNISETVAIKSHQQTDVTAATAEPHYHTPATTHVRTTRTNLHIYTGESPQDSGL
KTAGLRNSQHSQRGGRISRSNRAQRASSDYDPHSERLPGNSDTGDRLAAKEKGRIVGILHNDFPL
DDSIKAVLRQYYDNGIRLWQV*

>LN02|LN02_06556

MDVFIILKVIFSRTFYFNNIERIRDTLTFDDMVCKIKSYFENESIK*

>LN02|LN02_06586

MKGKAANNKLFKAPSNNNNDNDDNNNNENNNEDNNNNNNISNNNRNDTDSSNRPDERLK
DNNNNSDSKLLDRKENKEDRDKEEEEVITFSTNSYWDLTRSDLYYGLNTFFLMMNLEPGCTNK
GSKKQAVLDAFFNLKIGLYVFITNISIIGISVNL*

>LN02|LN02_06599

MVIQNTVGNVLDTDSGLERGNFGHISTILKKWLKIMKNRKNSEKPDEKSVCPTVPKIRRLLWAL
WPAVTGRCGRLWGAVPVP*

>LN02|LN02_06653

MASQNVPTANNPGYQVPDTAEELKKDFPEITHYYLPDLTSFRAISAAVEVLIPLEKRVISYKLAPR
TESGPISSGLEGTFDFQPPSNLEGVNIDQIALEEPIWKSPRQNMPISSIAEGIGLPQATDLIQPTGPQ
MGLIQHTDPPQPARQSFFTG*

>LN02|LN02_06668

MELKKDFLEITHYYLPDLTSFRAIGAAVEVLIPLEKRVISHKLAPRTESGRLLATLGNGTYLVYIPY
RWVVTKTSFITFTYQNEGIGPISSGLEGTFDFQPPSNLEGVNIDQIALEEPIWKSPRLDMPISIPTNR
KILRNRPIFRVKKDANNNPVKYKARLVVKGFMQVSGQDFTETYASTSIPPIWRIILALAIANNWEI
EQIDFIGAFLNSELLEMIYIEIPAVLAAFSDSDFAGCRLTSKSTSGYLTTLNGGPISWRSKRSSTVVLL
TLEAESDVDIQRPIPLYCDNQGSISNANDPNQHARTKHTLLKFRYICEKAHGGLKQVEANKVDK
PTAIKVKGVIIQQSIG*

>LN02|LN02_06714

MASQNVPTNNPGYQVPDTAEALNIAKFIEEPSIASPILSGLEGTFDFQPPFNLEGVNIDQIALEEPI
WKSPRLNMPISSKTDD*

>LN02|LN02_07025

MHYYLPDLTSFRAISAAIEVLIPPEKRVISHKLAPQTESGPILSGLEGTFDFQPPSNLEG*

>LN02|LN02_07036

MVVINRSSSPFEELKKDFPEITHYHLPDLTSFRAIGAAIEVLIPLEKRVISHKLAPRTESGRLLATLG
NGTYLVYIPHRRVVTKTSFITFTYQNKGIGPISSGLEGKFDFLPSSILEGVNNDQIALEEPIWKSPRL
NMPISSKTTI*

>LN02|LN02_07041

MPKQSAILDKGVNIGTSAACYPYALIGKTYIPLKRTPKN*

>LN02|LN02_07047

MPNSWKEISIPISRKILRNQPIFRVKKDANNNPVKYKARLVVKGFI*

>LN02|LN02_07048

MASQNVPTDNPSYTAEKQALMIQLRALNIAKFIEEPFIARKFNDLEQALLLLLIKNSCIEGPAAAI
NWCTNPELLATLGNGTYLVYIPYRRVVTKTSFITFTYQNKGIGPILSGLEGTFDFQPPSNLEGVNID
QIALEEPI*

>LN02|LN02_07052

MAELGSSSYKVPESNTKADATNKEPTKATKAGPSTKTTTTKDDNSEADGKEPIYITPLITITNQISN
RAIKELAKTANLFKESPKLKSPYNFNQWKQALTIQLRAFKIANFVNDLSIRAHLYPTFHTLTFTGY
KSTLEAFNSEFNSYIFRLTILGSKIDSFD*

>LN02|LN02_07055

MPYGPVKFRSIVVKLYNYNPNDAEDIVYTEGPACPGRLEEGKITTPRDPFKQSDHIEIKAL*

>LN02|LN02_07184

MLDDGFKRKREWRLEDARKLYVRDGFRPRTPSTAAGGPNAWRGTRLDETGLDMD*

>LN02|LN02_07187

MGVVPISRLVHLIRARIDAVGTRCDPSLFPVCLPQDRRRGGTPLPLAKRVLLCLEPRAASRELRAA
SKAECRSISMFNHTHVLVSVWACNPLSQYSALSATFVRHGQPPSTAGFMIDAPLGAASAVSSQH
RVYVT*

>LN02|LN02_07204

MPPLFPPSISITSSVPAAPTPWTRCAIEIRRLLWALWPAVTGRYGRLWGAVPVP*

>LN02|LN02_07205

MPSGTLLNTRPGPLLVLLGDSNTPYKEFEVEEILTTKNA*

>LN02|LN02_07270

MTAQAYAAIRCSFLVFGMRLNVLCNSHRHRLTFDDDLKVASVVFRINPGAEANIFAPEIAPVTID
QIWSTEALA*

>LN02|LN02_07337

MAALTMGKGVARIARSGALSENMLSMKRFAVKLEAGWQFLEAGWQFLEAGWQFLEAGWQFL
EAGWQFLEAGWQFI*

>LN02|LN02_07368

MPYSPVKFRLTVVKLYNYDPDYNTNPKDIVYPKGLARPGRLEVRIPAVAAPPAAITLAAAIYPSP
ATGPDQVRPVLPARMISDVIINEDQEEALVNAIASDTIIDVSFLIAKE*

>LN02|LN02_07376

MAQSGPSTYKATQTIKKEHTNITKARPSTRMTTTKDTNKDDIIEIDQDQPSIPIRYKEAKNYNKEA
AKAMDWYNNPEEAYLLLIAQYCHLPEGTLEAFNSEFNSYISRLKILGSKIDSFDQINHAQGRSKG
SSKVAVNKPIAILKASKDPKNKGSANAASTNNSKNNSTKKKQ*

>LN02|LN02_07381

MLDDGFKRKREWRLVHNVAVINVTKAEAIRGANGTESEVCTGREDARKLYVRDGFRPRTPSTA
AGGPNAWRGTRLDETGLDMD*

>LN02|LN02_07384

MGVVPISRLVHLIRARIDAVGTRCDPSLFPVCLPQDRRRGGTPLPLAKRVLLCLEPRAASRELRAA
SKAECRSISMFNHTHVLVSVWACNPLSQYSALSATFVRHGQPPSTAGFMIDAPLGAASAVSSQH
RVYVT*

>LN02|LN02_07405

MPPLFPPSISITSSVPAAPTPWTRCAIEIRRLLWALWPAVTGRYGRLWGAVPVP*

>LN02|LN02_07470

MTAQAYAAIRCSFLVFGMRLNVLCNSHRHRLTFDDDLKVASVVFRINPGAEANIFAPEIAPVTID
QIWSTEALA*

>LN02|LN02_07541

MAALTMGKGVARIARSGALSENMLSMKRFAVKLEAGWQFLEAGWQFLEAGWQFLEAGWQFL
EAGWQFLEAGWQFI*

>LN02|LN02_07572

MPYSPVKFRLTVVKLYNYDPDYNTNPKDIVYPKGLARPGRLEVRIPAVAAPPAAITLAAAIYPSP
ATGPDQVRPVLPARMISDVIINEDQEEALVNAIASDTIIDVSFLIAKE*

>LN02|LN02_07578

MAQSGPSTYKATQTIKKEHTNITKARPSTRMTTTKDTNKDDIIEIDQDQPSIPIRYKEAKNYNKEA
AKAMDWYNNPEEAYLLLIAQYCHLPEGTLEAFNSEFNSYISRLKILGSKIDSFDQINHAQGRSKG
SSKVAVNKPIAILKASKDPKNKGSANAASTNNSKNNSTKKKQ*

>LN02|LN02_07581

MASQNVPANNLGHNNPGHNNPGYQVPNTAEVEESDNNQEIAQLREDQ*

>LN02|LN02_07608

MPYWPTNLLALIPPFGLTATNKKPTKATEAGPSTKTTTKDNDSEADVMEIDQLSTPIKYKGLIVP
KRSKGSNKVAANNNPKQLKASKDSKNKGSTNTASTNNSKNNSTNNRKKK*

>LN02|LN02_07620

MHYHLPDLTSFRAISAAVEVLIPLEKRVISYKLAPRTELGPILSGLEGTFDF*

>LN02|LN02_07621

MAVAAAAITAANAIIAAVANANTTTIITALLYGYKLAPRTESGRLLAILGNGTYLIYIPYRRVVTK
TSFITFTYQNEGIGPILSGLEGTFDFQSPSNLEGVNIDQIALKEPI*

>LN02|LN02_07626

MPLPNRTAWIPGLSTDYPYSETYIVQMLYGPIKFRSTVVKPYNYDLNNPAADNAEDIVYTESPAR
PGRLEVRILAPAAAPGHYHYHQ*

>LN02|LN02_07698

MAELGSYKVPESDTKADINATNKKPTKATEARPSIKITTTKDDDEADGKEPIYIMALITITNQISNR
AIKKLAKTANRFKESQKLKSPYNFDQ*

>LN02|LN02_07759

MVIQNTVRNVLDTDSGLERGNFGHISTILKKWLKIMKNRKNSEKPDEKASPS*

>LN02|LN02_07925

MSLMQSSQTPSGYKSSYRQYQPQAGHNDSYTGLLQDDENVTRQLAEQEGRAVGLLYNDFPIDD
PLKAAIRDYYAKGIRIWQGVTFPGIDLPLWMMHYKEMRHSDSAAKYFSRLKQKSGSSLSISYKTQ
EHLGKGTHITTHAYASNSTTKKLKEASLTADKADSTLKPSG*

>LN02|LN02_07992

MCQKFEIFCRQLRLVVLKGSYDAGPQSHDIILSGEEEPQKD*

>LN02|LN02_07995

MVRRLENAGMRQLMEAIIKDLRAPLFLLITDKLATRRDLLLLIKHHKATILVPPTKFPSYYNPDN
KPIISILALGEKQDKTAKRQIGNNNNDNNDNNNNNENNNKDNNDNNNDISNNNGNNSNSG
DRPNKRLKGITSDFDPLSALFSSSSTLIDYLIRANGKGLPKAYSGLIEAE*

>LN02|LN02_08001

MLRTAFWSTRCQKFEIFRRQLRLVVLKGSYNTRPQSHDVDLSGEAEPQKDQITP*

>LN02|LN02_08002

MDSSRPSSPTLPSLLLPKPLQKYAKFVDKNWIDANEHIDPQNATDTDLYNRLIYRYNAYNIGED
MDDDLWDIFCDDFSE*

>LN02|LN02_08003

MDLIPGLFTNYYYIVKPYIYDLDKDPIEIVPTEAPAHHDSINTPARYNSINTPARPNSIEIVPTEAPA
HYDSMNTPARPDIINTPARPDIINTPARPSRLRLACSSRLEVRIPAAAALFNVIVNEDQEEAFINTE
KLQHLETHLNNLTKPK*

>LN02|LN02_08010

MTYHQAYQYHNVPRYYGKLLMQLENALQSVVKPYIYDLDKDPIEIVPTEAPAHHDSINTPARH
DSINTPACPNSIEIVPTEAPAYYDRW*

>LN02|LN02_08014

MARSRSSISQPALPERLQRFAPLLDDNWATTIELITPDATTIALYNRLAFLIYTYEKDYIINDNL*

>LN02|LN02_08015

MAWGAIYRTGRSPIIIMDRDEHLIVVNEGLKGFIWRSFSYCVGEIIVPIDPADLIYLLALI*

>LN02|LN02_08020

MPSPNGTAWIPGLSTNYPYSETYIVQMPYGPIKFRLIVVKPYNHDLNNPAADDAEDIVYAKGLA
CPGRLEVRIPALADQEEALVNTIASNTVINAFFLTAKE*

>LN02|LN02_08043

MTTSTYDPCLLISAGNDRTSDTDRTNDTDVPNRTNDTDVPNRTNDTNIPDRRSDTTCFGIKGQS
MKITTIDKNAPTA*

>LN02|LN02_08044

MPNRGPYRKATDKPFRNTLRNTPKKIIPNIGLAKKIIPEPGETYIVQMPYGPVKFRSTVVKPYNYD
LDNPAANNAEDIVYAEGPARPSRLEVRDQEEALINAIASNTIIDASFLTAKEQADL*

>LN02|LN02_08051

MTSSHSLKSSQNISYKSSSISSPISGSGSNKDGGIGDDGRDESIVLKEKRVIIHKLAPRTESGRLLATL
GNGTYLVYIPHRRVVTKTSFITFTYQNEGIGPISSGLEGIFDFQPPSNLKGVNNDQIALEEPI*

>LN02|LN02_08052

MDEIPIEAHNSISKVERYYAPLRRAYTIVNNETQRSSAYIISLRGNISQGVRSIIPSTNGTAWIYSLST
DYPYSPIYGETCIIQMPYSPIKFWSTVVKPYNYDPNNDPAADNPKDIDYAEGPACPGRLEDQEEA
LVNTIASNTIINASFLTAKEQADLQLAS*

>LN02|LN02_08063

MPYSPIKFRSTVVKPYNYNLNNNPTADNPKDIVYTEGPARPGRLETTYSCNIIVVNP*

>LN02|LN02_08065

MPYSPIKFRSTVVKPYNHNLNNDPAANNPEDIVYTEGPARPGRLEGTPNKALETFIDRN*

>LN02|LN02_08073

MRLMKTLTIEIIDKKHMVNFGAGSATSSGILNILMIFGDTKVPIIRK*

>LN02|LN02_08074

MKNRKNSEKPDENLPYGPEALYRSAVRAGSPIPVCRKGAV*

>LN02|LN02_08075

MASQNVPTADNLSYTAEELKKDFLEITHYHLPDLTSFRAIGAAVEVLIPPEKRVISHKLAPQTESG
RLLATLGNGTYLVYIPHRRVVTKTSFITFTYQNEGIGPISSGLEGIFDF*

>LN02|LN02_08077

MGVKLDNLENPYIYNLDKDPIETDPIEIIPTETPARHDSINTPARPNSIEIIPTEAPARHDSINTPARP
DIINTPARPNNINPARPDIINTPARSSRLEVRIPAAAALFNSDQAEIEALQARNIFRFTTYNNIKHE
W*

>LN02|LN02_08080

MPEYKLRPARPPLEITIASIDLKAIEASQIAKLKDSDNSDS*

>LN02|LN02_08136

MPKRSATLDKGAGTGAACYPYTLTGKTYMPLERTPKNRAPVQELLDAQVGNYKAL*

>LN02|LN02_08156

MANQLLNSTIMVSYYALVLVTFLPLELKKDFLEITHYYLPDLISFRAISAAVEVLIPLEKRVISYKLA
PRTESGPILSGLEGKFDFLLSSILKGVNTD*

>LN02|LN02_08169

MVIQNTVGNVLDTDSGLERGNFGHISTILKKWLKIMKNRKNSEKPDEKSVCPTVPNILPNAYPTK
PSGL*

>LN02|LN02_08171

MPTITKAIIPNKRGNFGHISTILKKWLKIMKNRKNSEKPDENRRPF*

>LN02|LN02_08181

MANVTQWDIEDAVAPQITATQYFQAPLQTEASPTETLQALQDKSCRHAFLQIDPFRMPSPPDRN
SAFLEEERYGDHTFRGILPDTGASGPSTAGLGQAKALMKLMKSLTIETTDKKHTVNFDAGSATSS
GILNVLMIFGNIPFHVLDSGTPFLYSITDMDRIGVKLNNIDNLLIKGDIKVPIIRKWGYL*

>LN02|LN02_08300

MPNSWKEVLYSLYKDSWTNIPTSRKILHNRPVFRVKKDANNNLVKYKARLVIKGFMQVSRQDF
TETYASTSIPPI*

>LN02|LN02_08557

MASQNVPANNPGHNNPGHNNPGYQVPNTAEELKKDFLEITHYHLPDLTSFRAISPILSGLEGKF
DFLPSSILEGVNID*

>LN02|LN02_08667

MAESGSSTYKVPESDTEADVNAQLQLDMDDMKSQMNTLTNLIQCLTTQATNKEPTKAGPSTKT
TTTKDNDEDDVMEIDQPSTPIKYKGKEPLYTITAPTTTITNQISDGAIKELAKTANRFKESQKLKSP
YNFD*

>LN02|LN02_08668

MVIQNTVGNVLDTDSGLERGNFGHISTILKKWLKIMKNRKNSEKPDKKSVCPTVPNILPNAYPT
KPSGL*

>LN02|LN02_08673

MDVDTFQTPIEKRVISHKLAPRTESGRLLATLGNGTYLVYIPYRQIVTKTSFITFTYQNEGIGPISSG
LEGTFDFQPPSNLEGVNID*

>LN02|LN02_08676

MDVDTFQTPVISHKLAPRTESGRLLATLGNSTYLVYILYRRVITKTSFITFTYQNKGIGPISSGLEGK
FDFLPFSILEGVNINQIALEEPI*

>LN02|LN02_08679

MPNSWKEVLYNPQKDSWSSVPTSRKILRNRPIFCVKKDANNNPVKYKARLVVKGFIQVSGQDF
METYASTSILLI*

>LN02|LN02_08684

MAESGSYKVPESDTEVDTNAQLQLDMDDMKSQMNILTDLIQGLVSRQNNDTQATQATNKKPT
KATEAGPSTKTTC*

>LN02|LN02_08696

MPEYKLKPAQPLLEITIASIDLKAIEALRIAELRDSDNSDS*

>LN02|LN02_08699

MASQNIPTADNLSYTAEALMIQLRALNIAKFIEDPSIASKFNDLEQALLLLLIKNSCIEGPAAAIN
WFDLLEEQRIKGLLVVRAITHLAKVKGAATNTNNSSKPASNNTSKGGKNTTTNANKKK*

>LN02|LN02_08717

MAVSTKYTIIAIPSPLNIRLDIPNSIYPVFYVNLIEYAASNPLPSQILIDTRPGPLLVLLENSNTLYKEF
EVEEILTTKNT*

>LN02|LN02_08718

MDTRPSPLLVLLKDSNTPHEEFKIKEILAAKNARGYSSKYNILDTAVREAFKVK*

>LN02|LN02_08720

MYDICYAVKIKAAKRAPEGVPNSWKEVLYNPYKDSWVKALFSEFEQLIDLNIFQFIPKTSVPTSRK
ILRNRPIFRVKKDANNNPVKYKTRLIVKGFMQVSGQDFTETYASTSIPPI*

>LN02|LN02_08724

MELKKDFPEIMHYYLPDLTSFRAISAAVEVLIPITDSGYARVMD*

>LN02|LN02_08730

MDDFIILKVISFKSKYRLLGLPDNTFARAFQIILIGDAKTYYFNNIKCIRNTLTFDDIVRKVKSHFEN
ESTKQFYLYH*

>LN02|LN02_08731

MPLTNGTAQIRSLSTDYPYNPTYGPVKFRLTVVKPYNHNPDDTKDIVYAEGPARPGRLEVRIPAA
AAPLAAIIPIAETSSTIPTVATYPSPATSPNQVRPVSLLPPPDM*

>LN02|LN02_08733

MSSEKQDKTAKRPIGDNNNDNDNNNNNENNNEDNDNNNNIGNNNGNNTNSGDRPDERLK
GTTSDFNPFGPNQSYRKRKQLLALPGYSATLEDVGLIGPNLARELKTYRS*

>LN02|LN02_08735

MKQALIVKDSGYACIMLRASSKKRVISHKLAPRTESGRLLVTLGNGTYLVYIPYRRVVTKTSFITFT
YQNKGIGPISSGLEGKFDFLPSSILEGVNADQITLEEPI*

>LN02|LN02_08736

MLKAIREVEWISNLFKEIQVDIQRPIPLYCDNQGSISNANDPNQYACTKHTLLKFRYIREKAYSGL
PYC*

>LN02|LN02_08847

MLGNGTYLVYILYRQVVTKTSFITFTYQNEGIGPISSGLEGKFDFLPSSILEGVNAD*

>LN02|LN02_09334

MVRRLENAGVRRLMEAIMKDLRALLFLLITDELATGRDLPSLVFDFNLTVMGYAMQLLKFAPA
NIKEWMKAGRVLGLG*

>LN02|LN02_09336

MISILKNNFCIFRSAKAIINSPDGHSITFPRIDLLLQTVYYEEVNYGILHKKLVKTHSKESALSSFTPP
SQGIDNIITIAIASNRLQATGNIEKPPNRNFSSQRAETNEGRVITFGTNSYRDLMRSDLYYEEWSDA
*

>LN02|LN02_09337

MTNNWHFAANIESFAGSDVLVCFTAYPIRYPHLVKDDIVVDRPS*

>LN02|LN02_01903

MAVNAADAARLPGIWNARKHCGTFVSHIEVIYGGVRPGVTSQDANINRGFGGDFVTDVALWR
SSAKQESPPEGWRGMSDDINSGREGDYLYLMWKLQRYAGPSNDEHLLEKASEVPSPWELLAQD
LVDVRRKCNEREPTAQRAAPNE*

>LN02|LN02_04450

MVFSTPVRAAEFKSAYGPKYKYQPHINGWSKTTILRKSASFGGAAVVGLFFYVSGIPRVQQDVLQ
KLPLVGRYFVKEEINPQDNPF*

>LN02|LN02_05756

MPTLAQQESEKDPWDQKTKQKFESKSKSEFYDPCQEAAQRSYKCLYRNGGDKAMCGEYFQAY
RECKAAWVERRRKEKGGFFG*

>LN02|LN02_08487

MAPDRTGYENRGGTRRVKLQDKPSGSISEGVSIPALVELYSACRCLYYQHAIDRCASYGRRGHCI
QQRTIYVGYACSAHTARSGQHAGSRQYSESGHYNSRSNTKSHR*

>LN02|LN02_09245

MAAPTYIISRVGDPIFAVLIGLSAAAMRIGREERAKGYTARQTVENGLRRIGFSKK*

>LN02|LN02_00289

MSNQEYYGGYPSQPTYAQGQHAQPGQPYHHQQQQQQQQQQPYQPYQEQPQHQPYQQHQQ
HQQHQQHQQHQPYQQHQQGYPPQQPTYGHPQHQHQHQQQHQHYAGSAPHPGSAPPGGPD
GERGLGATLVGGGGAAWAAHKAGGGFLGTAGAAIAGAIGANVLEHALDKKKKKKKDKKKK
HGARGLGSSSSSSSDSD*

Table S4. Gene ontology (GO) annotation of unique genes predicted in the genome of strain LN02.

Gene Ontology	GO_ID	Description	Count
Biological process	GO: 0015074	DNA integration	1
Cellular component	GO: 0005575	Cellular component	1
	GO: 0005622	Intracellular	1
	GO: 0005739	Mitochondrion	1
	GO: 0043226	Organelle	1
	GO: 0043229	Intracellular organelle	1
	GO: 0044464	Cell part	1

(A)

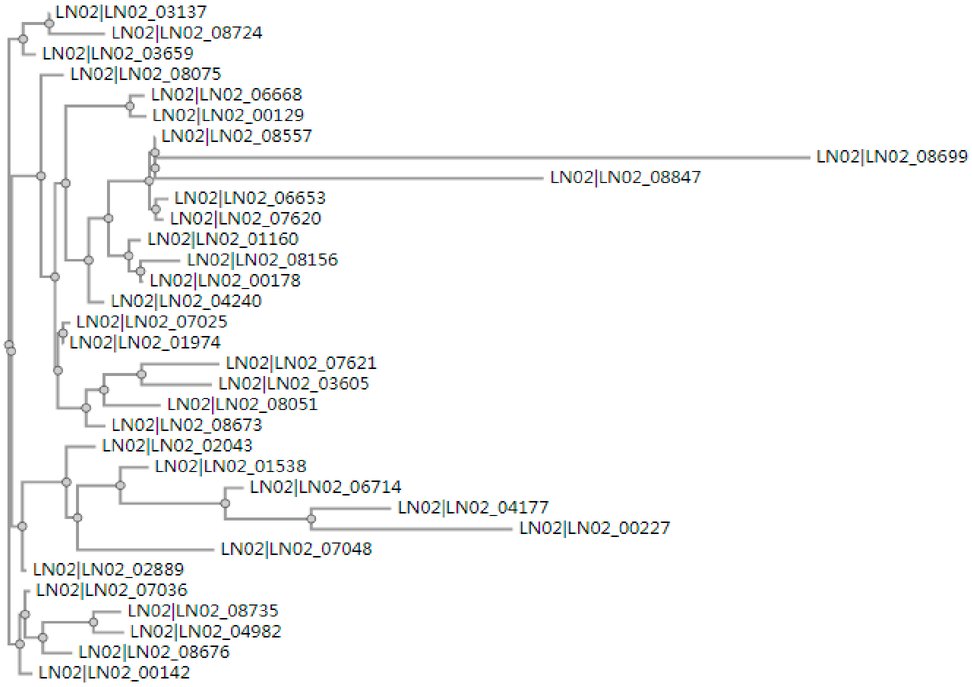

(B)

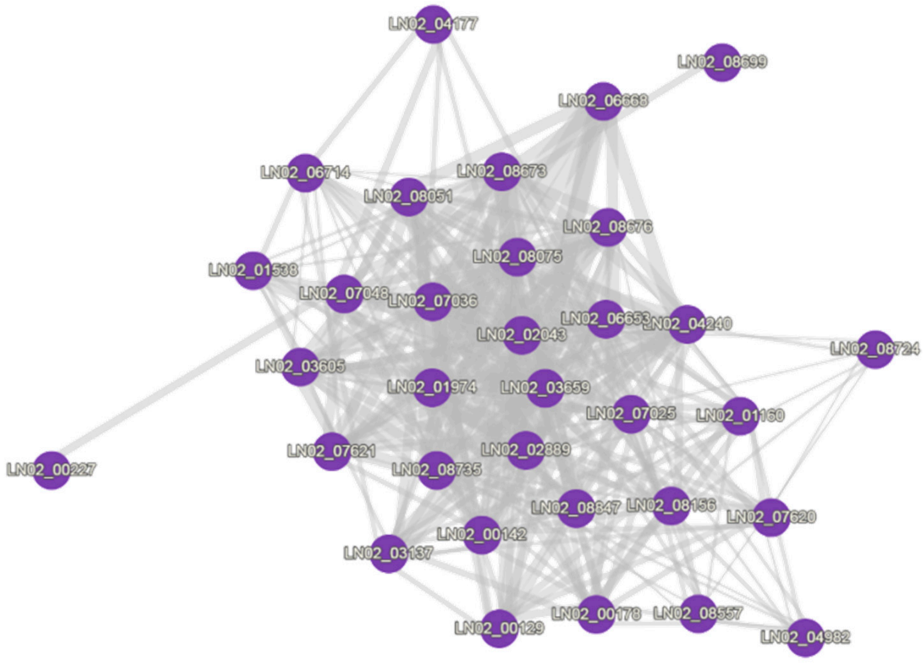

Figure S2. The phylogenetic tree and enrichment analysis of partial unique genes (e.g., cluster 3) in strain LN02.

(**A**) Phylogenetic tree of partial unique genes (e.g., cluster 3) of strain LN02; (**B**) Enrichment analysis of partial unique proteins (e.g., cluster 3) in strain LN02.

Table S5. Gene ontology (GO) annotation of predicted proteins in strain LN02 genome.

Gene Ontology	GO ID	Description	Count
Biological process	GO: 0015031	Protein transport	1
	GO: 0006081	Cellular aldehyde metabolic process	1
	GO: 0006725	Cellular aromatic compound metabolic process	1
	GO: 0006810	Transport	1
	GO: 0008152	Metabolic process	1
	GO: 0015031	Protein transport	1
	GO: 0044237	Cellular metabolic process	1
	GO: 0051234	Establishment of loc alization	1
	GO: 0005975	Carbohydrate metabolic process	4
	GO: 0005976	Polysaccharide metabolic process	3
	GO: 0006066	Alcohol metabolic process	2
	GO: 0006139	Nucleobase-containing compound metabolic process	1
	GO: 0006464	Cellular protein modification process	1
	GO: 0006629	Lipid metabolic process	3
	GO: 0006725	Cellular aromatic compound metabolic process	1
	GO: 0006807	Nitrogen compound metabolic process	3
	GO: 0006810	Transport	1
	GO: 0006865	Amino acid transport	2
	GO: 0006996	Organelle organization	1
	GO: 0007049	Cell cycle	1
	GO: 0007154	Cell communication	1
	GO: 0008150	Biological_process	10
	GO: 0008152	Metabolic process	8
	GO: 0009308	Amine metabolic process	1
	GO: 0009987	Cellular process	6
	GO: 0015833	Peptide transport	1
	GO: 0016070	RNA metabolic process	1
	GO: 0042710	Biofilm formation	1
	GO: 0043170	Macromolecule metabolic process	5
	GO: 0043412	Macromolecule modification	1
	GO: 0044237	Cellular metabolic process	7
	GO: 0044238	Primary metabolic process	1
	GO: 0044255	Cellular lipid metabolic process	2
	GO: 0046483	Heterocycle metabolic process	1
	GO: 0050896	Response to stimulus	3
	GO: 0051234	Establishment of localization	1
	GO: 0051703	Intraspecies interaction between organisms	1
	GO: 0051704	multi-organism process	1
	GO: 0065007	Biological regulation	3
	GO: 0071555	Cell wall organization	1
	GO: 0005975	Carbohydrate metabolic process	2
	GO: 0005976	Polysaccharide metabolic process	2
	GO: 0006139	Nucleobase-containing compound metabolic process	1

	GO: 0006725	Cellular aromatic compound metabolic process	1
	GO: 0006807	Nitrogen compound metabolic process	1
	GO: 0008150	Biological process	1
	GO: 0008152	metabolic process	3
	GO: 0009987	cellular process	1
	GO: 0016070	RNA metabo lic process	1
	GO: 0043170	macromolecule metabolic process	3
	GO: 0044237	cellular metabolic process	1
	GO: 0044238	Primary metabolic process	1
	GO: 0046483	Heterocycle metabolic process	1
	GO: 0065007	biological regulation	1
	GO: 0000003	Reproduction	2
	GO: 0005975	carbohydrate metabolic process	3
	GO: 0005976	polysaccharide metabolic process	1
	GO: 0006081	cellular aldehyde metabolic process	1
	GO: 0006082	Organic acid metabolic process	6
	GO: 0006139	Nucleobase-containing compound metabolic process	6
	GO: 0006412	translation	3
	GO: 0006464	Cellular protein modification process	3
	GO: 0006508	Proteolysis	5
	GO: 0006518	Peptide metabolic process	2
	GO: 0006629	Lipid metabolic process	10
	GO: 0006725	cellular aromatic compound metabolic process	7
	GO: 0006793	phosphorus metabolic process	6
	GO: 0006807	nitrogen compound metabolic process	14
	GO: 0006810	transport	5
	GO: 0006811	ion transport	1
	GO: 0006818	hydrogen transport	1
	GO: 0006865	amino acid transport	1
	GO: 0006996	organelle organization	1
	GO: 0007005	mitochondrion organization	1
	GO: 0007033	vacuole organization	1
	GO: 0007049	cell cycle	1
	GO: 0007154	cell communication	2
	GO: 0008150	biological_process	34
	GO: 0008152	metabolic process	30
	GO: 0009116	nucleoside metabolic process	1
	GO: 0009117	nucleotide metabolic process	3
	GO: 0009308	amine metabolic process	1
	GO: 0009987	cellular process	13
	GO: 0016032	viral process	1
	GO: 0016043	cellular component organization	1
	GO: 0016070	RNA metabolic process	4
	GO: 0017144	drug metabolic process	2
	GO: 0019538	protein metabolic process	2
	GO: 0019748	secondary metabolic process	1
	GO: 0032501	multicellular organismal process	2
	GO: 0032502	developmental process	2
	GO: 0042440	pigment metabolic process	1

	GO: 0043170	macromolecule metabolic process	8
	GO: 0043412	macromolecule modification	1
	GO: 0043603	cellular amide metabolic process	2
	GO: 0044237	cellular metabolic process	25
	GO: 0044238	primary metabolic process	9
	GO: 0044255	cellular lipid metabolic process	8
	GO: 0044419	interspecies interaction between organisms	1
	GO: 0046483	heterocycle metabolic process	8
	GO: 0048284	organelle fusion	1
	GO: 0050896	response to stimulus	5
	GO: 0051186	cofactor metabolic process	3
	GO: 0051189	prosthetic group metabolic process	1
	GO: 0051234	Establishment of localization	5
	GO: 0051604	Protein maturation	1
	GO: 0051704	multi-organism process	6
	GO: 0065007	Biological regulation	5
	GO: 0071555	Cell wall organization	1
	GO: 0006082	organic acid metabolic process	1
	GO: 0006139	nucleobase-containing compound metabolic process	1
	GO: 0006396	RNA processing	1
	GO: 0006464	Cellular protein modification process	1
	GO: 0006629	Lipid metabolic process	2
	GO: 0006725	Cellular aromatic compound metabolic process	1
	GO: 0006793	Phosphorus metabolic process	1
	GO: 0006807	Nitrogen compound metabolic process	1
	GO: 0008150	Biological process	3
	GO: 0008152	Metabolic process	4
	GO: 0016070	RNA metabolic process	2
	GO: 0042254	Ribosome biogenesis	1
	GO: 0043170	Macromolecule metabolic process	3
	GO: 0044237	Cellular metabolic process	3
	GO: 0044238	Primary metabolic process	1
	GO: 0044255	Cellular lipid metabolic process	2
	GO: 0046483	Heterocycle metabolic process	1
	GO: 0000280	Nuclear division	1
	GO: 0006139	Nucleobase-containing compound metabolic process	1
	GO: 0006508	Proteolysis	1
	GO: 0006725	Cellular aromatic compound metabolic process	1
	GO: 0006807	Nitrogen compound metabolic process	1
	GO: 0006996	Organelle organization	1
	GO: 0007049	Cell cycle	1
	GO: 0007059	Chromosome segregation	1
	GO: 0008150	Biological process	1
	GO: 0008152	Metabolic process	1
	GO: 0009987	Cellular process	1
	GO: 0016043	Cellular component organization	1
	GO: 0016070	RNA metabolic process	1

	GO: 0043170	Macromolecule metabolic process	1
	GO: 0044237	cellular metabolic process	1
	GO: 0044238	primary metabolic process	1
	GO: 0046483	heterocycle metabolic process	1
	GO: 0051276	chromosome organization	1
	GO: 0051604	Protein maturation	1
	GO: 0065007	Biological regulation	1
	GO: 0006725	Cellular aromatic compound metabolic process	1
	GO: 0006807	Nitrogen compound metabolic process	1
	GO: 0007154	Cell communication	1
	GO: 0008150	biological_process	2
	GO: 0008152	metabolic process	1
	GO: 0009308	amine metabolic process	1
	GO: 0009987	cellular process	1
	GO: 0042445	hormone metabolic process	1
	GO: 0044237	cellular metabolic process	1
	GO: 0046483	heterocycle metabolic process	1
	GO: 0050896	response to stimulus	1
	GO: 0051704	multi-organism process	1
	GO: 0065007	Biological regulation	2
	GO: 0006629	Lipid metabolic process	1
	GO: 0006793	phosphorus metabolic process	1
	GO: 0006810	Transport	1
	GO: 0006928	Movement of cell or subcellular component	1
	GO: 0008150	Biological process	4
	GO: 0008152	Metabolic process	4
	GO: 0009987	Cellular process	3
	GO: 0016043	Cellular component organization	1
	GO: 0017144	Drug metabolic process	2
	GO: 0032502	Developmental process	1
	GO: 0034622	Cellular macromolecular complex assembly	1
	GO: 0044237	Cellular metabolic process	3
	GO: 0044255	Cellular lipid metabolic process	2
	GO: 0051179	Localization	1
	GO: 0051234	Establishment of localization	1
	GO: 0051640	Organelle localization	1
	GO: 0051641	Cellular localization	1
	GO: 0065003	Macromolecular complex assembly	1
	GO: 0006810	Transport	1
	GO: 0006996	Organelle organization	1
	GO: 0007033	Vacuole organization	1
	GO: 0008150	Biological process	3
	GO: 0009987	Cellular process	2
	GO: 0016043	Cellular component organization	2
	GO: 0034622	Cellular macromolecular complex assembly	1
	GO: 0048284	Organelle fusion	1
	GO: 0051234	Establishment of localization	1
	GO: 0051704	Multi-organism process	1
	GO: 0065003	Macromolecular complex assembly	1
	GO: 0000003	Reproduction	1

	GO: 0005975	Carbohydrate metabolic process	1
	GO: 0005976	Polysaccharide metabolic process	1
	GO: 0006082	Organic acid metabolic process	3
	GO: 0006139	Nucleobase-containing compound metabolic process	2
	GO: 0006464	Cellular protein modification process	1
	GO: 0006508	Proteolysis	2
	GO: 0006725	Cellular aromatic compound metabolic process	2
	GO: 0006793	Phosphorus metabolic process	2
	GO: 0006807	Nitrogen compound metabolic process	3
	GO: 0006810	Transport	4
	GO: 0006869	lipid transport	1
	GO: 0007049	cell cycle	2
	GO: 0008150	Biological process	15
	GO: 0008152	metabolic process	8
	GO: 0009117	nucleotide metabolic process	1
	GO: 0009987	cellular process	4
	GO: 0015031	Protein transport	1
	GO: 0015833	Peptide transport	1
	GO: 0016070	RNA metabolic process	1
	GO: 0019538	Protein metabolic process	2
	GO: 0043170	Macromolecule metabolic process	4
	GO: 0043603	Cellular amide metabolic process	1
	GO: 0044237	Cellular metabolic process	6
	GO: 0044238	Primary metabolic process	5
	GO: 0044255	Cellular lipid metabolic process	1
	GO: 0045333	Cellular respiration	1
	GO: 0046483	Heterocycle metabolic process	2
	GO: 0050896	Response to stimulus	3
	GO: 0051179	Localization	1
	GO: 0051234	Establishment of localization	4
	GO: 0051704	Multi-organism process	5
	GO: 0065007	Biological regulation	2
Molecular function	GO: 0003674	Molecular function	1
	GO: 0003676	Nucleic acid binding	1
	GO: 0004497	Monooxygenase activity	2
	GO: 0008233	peptidase activity	1
	GO: 0016491	oxidoreductase activity	2
	GO: 0016740	transferase activity	1
	GO: 0016787	hydrolase activity	1
	GO: 0016740	transferase activity	2
	GO: 0000166	Nucleotide binding	1
	GO: 0001882	Nucleoside binding	1
	GO: 0003674	Molecular function	3
	GO: 0005215	Transporter activity	1
	GO: 0005488	Binding	2
	GO: 0008233	Peptidase activity	2
	GO: 0016491	Oxidoreductase activity	3
	GO: 0016740	Transferase activity	4
	GO: 0019842	Vitamin binding	1

	GO: 0033218	Amide binding	1
	GO: 0043167	Ion binding	1
	GO: 0004497	Monooxygenase activity	1
	GO: 0016787	Hydrolase activity	1
	GO: 0043167	Ion binding	1
	GO: 0043167	Ion binding	1
	GO: 0016787	Hydrolase activity	1
	GO: 0000166	Nucleotide binding	1
	GO: 0001882	Nucleoside binding	1
	GO: 0003676	Nucleic acid binding	1
	GO: 0004497	Monooxygenase activity	1
	GO: 0005488	Binding	1
	GO: 0008233	Peptidase activity	1
	GO: 0016491	Oxidoreductase activity	1
	GO: 0016740	Transferase activity	1
	GO: 0016787	Hydrolase activity	1
	GO: 0043167	Ion binding	1
Cellular component	GO: 0005739	Mitochondrion	1
	GO: 0005576	Extracellular region	1
	GO: 0005634	Nucleus	1
	GO: 0005773	Vacuole	1
	GO: 0005575	Cellular component	1
	GO: 0005622	Intracellular	1
	GO: 0005739	Mitochondrion	1
	GO: 0043226	Organelle	1
	GO: 0043229	Intracellular organelle	1
	GO: 0044464	Cell part	1
Go enrichment	GO: 0016712	Oxidoreductase activity, acting on paired donors, with incorporation or reduction of molecular oxygen, reduced flavin or flavoprotein as one donor, and incorporation of one atom of oxygen; Molecular function; 0.0002743762007201826	2
	GO: 0000272	Polysaccharide catabolic process; biological process; 0.00048372626639220134	2
	GO: 0006508	Proteolysis; Biological process; 0.00018568319398507856	4
	GO: 0017000	Antibiotic biosynthetic process; Biological process; 0.00034490961143298157	2
	GO: 0009405	Pathogenesis; Biological process; 0.00011775133821952776	5

Table S6. Clusters of orthologous groups (COG) annotation of predicted proteins in strain LN02 genome.

Query id	Subject id	Identity (%)	Align length	Mismatch	Gap openings	Query start	Query end	Subject start	Subject end	E-value	Score	Ortholog group	Predicted gene name	OG Description
LN02_00065|LN02 Chr01:372321-373457(-)|297	CDD:223729	41.958	286	151	5	9	291	5	278	1.14e-110	319	COG0656	ARA1	Aldo/keto reductases, related to diketogulonate reductase
LN02_00257|LN02 Chr01:1477273-1480590(-)|1001	CDD:224136	16.716	335	229	9	420	751	115	402	2.64e-29	119	COG1215	COG1215	Glycosyltransferases, probably involved in cell wall biogenesis
LN02_00321|LN02 Chr01:1772927-1773991(+)|354	CDD:224919	31.953	338	219	7	13	345	2	333	1.82e-91	274	COG2008	GLY1	Threonine aldolase
LN02_00449|LN02 Chr01:2151173-2152999(+)|584	CDD:224729	25.914	301	189	17	231	528	64	333	8.73e-20	88.1	COG1816	Add	Adenosine deaminase
LN02_00961|LN02 Chr01:3938574-3939752(+)|362	CDD:223745	32.865	356	218	3	7	361	5	340	2.03e-73	228	COG0673	MviM	Predicted dehydrogenases and related proteins
LN02_01025|LN02 Chr01:4141001-4144391(-)|1014	CDD:223496	17.722	237	163	7	576	804	254	466	3.34e-05	44.8	COG0419	SbcC	ATPase involved in DNA repair
LN02_01345|LN02 Chr01:5374058-5376622(-)|705	CDD:225201	18.220	236	154	8	218	425	117	341	2.79e-04	40.8	COG2319	COG2319	FOG: WD40 repeat
LN02_01729|LN02 Chr01:6869886-6870955(-)|345	CDD:227069	26.012	173	108	10	165	323	13	179	3.54e-06	44.2	COG4725	IME4	Transcriptional activator, adenine-specific DNA methyltransferase
LN02_01921|LN02 Chr01:7413713-7414490(-)|224	CDD:223376	35.071	211	111	7	10	219	2	187	9.01e-46	148	COG0299	PurN	Folate-dependent phosphoribosylglycinamide formyltransferase PurN
LN02_01985|LN02 Chr02:205788-208883(-)|1031	CDD:225805	22.500	160	104	5	314	456	62	218	1.57e-06	48.0	COG3266	DamX	Uncharacterized protein conserved in bacteria
LN02_02049|LN02 Chr02:462953-464482(+)|138	CDD:227479	54.264	129	57	1	8	136	6	132	6.56e-49	151	COG5150	COG5150	Class 2 transcription repressor NC2, beta subunit (Dr1)
LN02_02177|LN02 Chr02:825212-825836(+)|104	CDD:227835	30.337	89	62	0	5	93	4	92	1.25e-15	63.7	COG5548	COG5548	Small integral membrane protein

LN02_02241ǀLN02 Chr02:1043638-1046376(-)ǀ776	CDD:223671	26.380	326	201	8	448	771	30	318	3.62e-71	233	COG0598	CorA	Mg2+ and Co2+ transporters
LN02_02433ǀLN02 Chr02:1803391-1805723(+)ǀ714	CDD:226000	21.053	285	143	16	60	301	71	316	4.76e-10	58.7	COG3469	COG3469	Chitinase
LN02_02497ǀLN02 Chr02:2027243-2029338(-)ǀ658	CDD:227598	21.569	255	161	8	334	575	10	238	6.22e-35	131	COG5273	COG5273	Uncharacterized protein containing DHHC-type Zn finger
LN02_02753ǀLN02 Chr02:2855055-2857170(+)ǀ581	CDD:227863	27.184	103	65	1	18	110	9	111	8.23e-13	63.6	COG5576	COG5576	Homeodomain-containing transcription factor
LN02_02945ǀLN02 Chr02:3541443-3544104(-)ǀ820	CDD:225201	19.668	422	254	13	67	485	21	360	2.73e-26	110	COG2319	COG2319	FOG: WD40 repeat
LN02_02945ǀLN02 Chr02:3541443-3544104(-)ǀ820	CDD:225201	18.810	420	267	14	400	817	58	405	9.26e-15	74.7	COG2319	COG2319	FOG: WD40 repeat
LN02_03073ǀLN02 Chr02:4001947-4005313(-)ǀ1094	CDD:223605	12.707	181	132	5	32	192	50	224	7.18e-05	43.6	COG0531	PotE	Amino acid transporters
LN02_03201ǀLN02 Chr02:4475160-4479929(+)ǀ1531	CDD:225201	29.501	461	296	13	989	1437	20	463	6.54e-58	205	COG2319	COG2319	FOG: WD40 repeat
LN02_04097ǀLN02 Chr03:2324222-2328439(-)ǀ1383	CDD:227623	25.594	379	238	13	413	762	296	659	2.25e-41	162	COG5307	COG5307	SEC7 domain proteins
LN02_04417ǀLN02 Chr03:4106839-4109137(+)ǀ663	CDD:223624	28.702	655	362	15	2	647	1	559	1.50e-151	447	COG0550	TopA	Topoisomerase IA
LN02_04673ǀLN02 Chr03:5020456-5022449(+)ǀ562	CDD:223712	31.737	167	93	7	192	350	1	154	1.37e-22	91.6	COG0639	ApaH	Diadenosine tetraphosphatase and related serine/threonine protein phosphatases
LN02_05249ǀLN02 Chr04:1106564-1107847(+)ǀ387	CDD:224117	20.082	244	166	7	113	339	144	375	8.11e-05	41.6	COG1196	Smc	Chromosome segregation ATPases
LN02_05313ǀLN02 Chr04:1341647-1342660(-)ǀ309	CDD:223943	19.522	251	152	8	25	272	2	205	1.44e-15	71.9	COG1011	COG1011	Predicted hydrolase (HAD superfamily)
LN02_05377ǀLN02 Chr04:1537867-1540023(+)ǀ670	CDD:224729	27.557	352	234	7	158	505	1	335	2.47e-100	308	COG1816	Add	Adenosine deaminase
LN02_05441ǀLN02 Chr04:1769444-1770431(-)ǀ239	CDD:223477	27.700	213	124	7	28	235	20	207	1.16e-35	123	COG0400	COG0400	Predicted esterase

LN02_05633|LN02 Chr04:2695824-2699520(-)|1076	CDD:227361	33.372	869	509	20	198	1047	43	860	0.0	555	COG5028	COG5028	Vesicle coat complex COPII, subunit SEC24/subunit SFB2/subunit SFB3
LN02_05697|LN02 Chr04:2912460-2913346(-)|223	CDD:227410	30.303	165	74	4	1	141	4	151	2.41e-32	112	COG5078	COG5078	Ubiquitin-protein ligase
LN02_05889|LN02 Chr04:3523151-3526012(+)|929	CDD:224117	26.923	104	75	1	164	266	844	947	3.33e-06	48.2	COG1196	Smc	Chromosome segregation ATPases
LN02_06081|LN02 Chr04:4198418-4199764(+)|380	CDD:225136	35.897	117	73	2	119	234	41	156	3.83e-21	88.5	COG2226	UbiE	Methylase involved in ubiquinone/menaquinone biosynthesis
LN02_06273|LN02 Chr04:4857795-4860740(+)|963	CDD:224117	17.760	366	280	7	213	570	168	520	6.19e-13	70.1	COG1196	Smc	Chromosome segregation ATPases
LN02_06401|LN02 Chr04:5267305-5268864(+)|519	CDD:223589	18.617	188	140	3	204	391	32	206	1.29e-06	47.4	COG0515	SPS1	Serine/threonine protein kinase
LN02_06465|LN02 Chr04:5466369-5469759(-)|1063	CDD:225857	24.383	324	216	12	696	1003	2	312	7.09e-28	114	COG3320	COG3320	Putative dehydrogenase domain of multifunctional non-ribosomal peptide synthetases and related enzymes
LN02_06465|LN02 Chr04:5466369-5469759(-)|1063	CDD:223395	20.761	578	368	26	11	549	2	528	8.18e-28	116	COG0318	CaiC	Acyl-CoA synthetases (AMP-forming)/AMP-acid ligases II
LN02_06529|LN02 Chr04:5742222-5742977(-)|251	CDD:223589	21.693	189	125	6	38	204	38	225	9.64e-18	78.6	COG0515	SPS1	Serine/threonine protein kinase
LN02_06721|LN02 Chr05:593567-595622(+)|591	CDD:224117	18.841	69	56	0	165	233	357	425	1.63e-04	41.6	COG1196	Smc	Chromosome segregation ATPases
LN02_06785|LN02 Chr05:810343-812477(-)|339	CDD:223796	19.565	184	124	6	1	163	110	290	2.90e-11	60.7	COG0724	COG0724	RNA-binding proteins (RRM domain)
LN02_06849|LN02 Chr05:1023629-1030800(-)|2189	CDD:227505	56.623	2310	768	24	83	2178	62	2351	0.0	3053	COG5178	PRP8	U5 snRNP spliceosome subunit
LN02_07297|LN02 Chr05:3252122-3253486(+)|230	CDD:225003	34.091	88	57	1	143	230	2	88	3.02e-27	97.4	COG2092	EFB1	Translation elongation factor EF-1beta
LN02_07361|LN02 Chr05:3444639-3445725(-)|313	CDD:227458	49.600	250	104	5	3	251	2	230	1.80e-81	246	COG5129	MAK16	Nuclear protein with HMG-like acidic region

LN02_07425|LN02 Chr05:3756025-3756468(+)|112	CDD:224969	42.857	112	61	2	1	112	1	109	8.40e-12	54.3	COG2058	RPP1A	Ribosomal protein L12E/L44/L45/RPP1/RPP2
LN02_07553|LN02 Chr05:4140868-4141893(+)|341	CDD:226000	26.515	132	78	3	47	163	53	180	8.72e-07	47.2	COG3469	COG3469	Chitinase
LN02_07681|LN02 Chr05:4818347-4819943(+)|382	CDD:223712	29.048	210	88	8	94	297	1	155	5.41e-22	88.6	COG0639	ApaH	Diadenosine tetraphosphatase and related serine/threonine protein phosphatases
LN02_08449|LN02 Chr06:2647650-2650439(-)|929	CDD:227122	24.167	120	90	1	603	721	313	432	2.21e-06	48.2	COG4783	COG4783	Putative Zn-dependent protease, contains TPR repeats
LN02_08449|LN02 Chr06:2647650-2650439(-)|929	CDD:227122	25.000	108	81	0	677	784	320	427	1.49e-04	42.4	COG4783	COG4783	Putative Zn-dependent protease, contains TPR repeats
LN02_08513|LN02 Chr06:2838113-2839036(+)|307	CDD:223943	21.116	251	163	8	46	285	1	227	2.15e-25	98.4	COG1011	COG1011	Predicted hydrolase (HAD superfamily)
LN02_08769|LN02 Chr07:296814-297879(+)|223	CDD:224066	42.169	166	86	3	63	223	1	161	3.08e-66	199	COG1143	NuoI	Formate hydrogenlyase subunit 6/NADH:ubiquinone oxidoreductase 23 kD subunit (chain I)
LN02_09089|LN02 Chr07:1388312-1389183(-)|247	CDD:223649	37.500	200	116	2	47	246	2	192	8.06e-50	159	COG0576	GrpE	Molecular chaperone GrpE (heat shock protein)
LN02_09281|LN02 Chr07:2025995-2027388(-)|349	CDD:225201	26.336	262	175	7	46	303	195	442	1.46e-23	98.2	COG2319	COG2319	FOG: WD40 repeat
LN02_00066|LN02 Chr01:373940-375387(-)|376	CDD:223758	29.787	94	43	5	11	95	8	87	9.90e-09	53.5	COG0686	Ald	Alanine dehydrogenase
LN02_00258|LN02 Chr01:1483204-1485046(+)|572	CDD:224536	31.546	485	266	18	45	511	24	460	2.33e-101	312	COG1621	SacC	Beta-fructosidases (levanase/invertase)
LN02_00386|LN02 Chr01:1977891-1979519(+)|507	CDD:227388	33.911	404	214	14	15	402	8	374	4.70e-84	262	COG5055	RAD52	Recombination DNA repair protein (RAD52 pathway)
LN02_00450|LN02 Chr01:2154162-2155441(-)|318	CDD:223774	21.212	132	85	5	22	141	21	145	4.18e-04	38.3	COG0702	COG0702	Predicted nucleoside-diphosphate-sugar epimerases
LN02_00514|LN02 Chr01:2332662-2333390(-)|219	CDD:223393	43.636	110	60	1	112	219	1	110	2.31e-45	143	COG0316	sufA	Fe-S cluster assembly scaffold protein

LN02_00578|LN02 Chr01:2601268-2602431(-)|307	CDD:223084	37.549	253	151	5	9	258	14	262	3.23e-88	261	COG0005	Pnp	Purine nucleoside phosphorylase
LN02_00706|LN02 Chr01:3101511-3102377(+)|288	CDD:225136	33.043	115	71	2	47	161	50	158	5.47e-20	83.9	COG2226	UbiE	Methylase involved in ubiquinone/menaquinone biosynthesis
LN02_00770|LN02 Chr01:3304937-3305552(+)|154	CDD:227883	35.766	137	80	2	5	133	27	163	1.72e-38	126	COG5596	TIM22	Mitochondrial import inner membrane translocase, subunit TIM22
LN02_01026|LN02 Chr01:4146677-4149507(-)|852	CDD:224419	16.754	382	199	14	86	460	41	310	8.05E-16	78	COG1502	Cls	Phosphatidylserine/phosphatidyl glycerophosphate/cardiolipin synthases and related enzymes
LN02_01282|LN02 Chr01:5143835-5144879(+)|180	CDD:223725	50.365	137	57	6	24	157	3	131	1.70E-60	182	COG0652	PpiB	Peptidyl-prolyl cis-trans isomerase (rotamase) - cyclophilin family
LN02_01346|LN02 Chr01:5379761-5381881(+)|673	CDD:223520	58.375	603	216	6	49	649	7	576	0.0	746	COG0443	DnaK	Molecular chaperone
LN02_01410|LN02 Chr01:5637261-5639490(-)|605	CDD:223995	40.55	582	296	14	18	597	4	537	0.0	543	COG1069	AraB	Ribulose kinase
LN02_01602|LN02 Chr01:6435614-6436971(-)|381	CDD:223168	44.516	310	133	8	61	368	3	275	3.38E-99	292	COG0090	RplB	Ribosomal protein L2
LN02_01666|LN02 Chr01:6688394-6689499(+)|317	CDD:223798	27.064	218	135	8	103	313	66	266	1.42E-31	116	COG0726	CDA1	Predicted xylanase/chitin deacetylase
LN02_01858|LN02 Chr01:7233348-7235990(-)|880	CDD:227455	31.667	60	36	3	292	347	59	117	1.36E-04	40.4	COG5126	FRQ1	Ca2+-binding protein (EF-Hand superfamily)
LN02_01858|LN02 Chr01:7233348-7235990(-)|880	CDD:227445	26.316	38	28	0	152	189	8	45	2.90E-04	41.2	COG5114	COG5114	Histone acetyltransferase complex SAGA/ADA, subunit ADA2
LN02_01922|LN02 Chr01:7417139-7420887(+)|1070	CDD:227881	30.477	817	447	14	50	837	10	734	3.80E-156	481	COG5594	COG5594	Uncharacterized integral membrane protein
LN02_02050|LN02 Chr02:465117-466661(+)|466	CDD:223915	41.176	238	118	9	36	261	13	240	3.80E-67	212	COG0846	SIR2	NAD-dependent protein deacetylases, SIR2 family
LN02_02114|LN02 Chr02:655881-657603(+)|422	CDD:223299	27.723	202	115	4	162	363	1	171	2.24E-60	191	COG0221	Ppa	Inorganic pyrophosphatase
LN02_02434|LN02 Chr02:1806958-1811125(+)|1266	CDD:223738	40.476	84	44	2	152	230	105	187	7.23E-11	61	COG0666	Arp	FOG: Ankyrin repeat

LN02_02434|LN02 Chr02:1806958-1811125(+)|1266	CDD:223738	25.556	180	91	6	183	355	67	210	1.63E-05	44.8	COG0666	Arp	FOG: Ankyrin repeat
LN02_02626|LN02 Chr02:2463535-2465361(+)|562	CDD:224083	28.344	314	180	9	192	501	24	296	6.52E-51	174	COG1161	COG1161	Predicted GTPases
LN02_02690|LN02 Chr02:2692433-2695564(-)|739	CDD:223518	42.607	629	315	14	109	735	1	585	0.0	665	COG0441	ThrS	Threonyl-tRNA synthetase
LN02_02754|LN02 Chr02:2857985-2859430(-)|481	CDD:223139	31.847	314	180	6	99	411	1	281	1.39E-75	236	COG0061	nadF	NAD kinase
LN02_02882|LN02 Chr02:3308456-3309012(-)|109	CDD:223335	60.465	43	11	1	66	108	1	37	3.72E-12	53.5	COG0257	RpmJ	Ribosomal protein L36
LN02_02946|LN02 Chr02:3544316-3545188(+)|113	CDD:227460	40.206	97	53	2	17	113	5	96	2.89E-23	83.4	COG5131	URM1	Ubiquitin-like protein
LN02_03202|LN02 Chr02:4480325-4480822(-)|115	CDD:224417	28.571	84	53	3	11	94	10	86	3.80E-06	41.1	COG1500	COG1500	Predicted exosome subunit
LN02_03586|LN02 Chr02:6342100-6344052(-)|606	CDD:226042	34.36	422	230	14	3	404	70	464	1.79E-77	252	COG3511	PlcC	Phospholipase C
LN02_03650|LN02 Chr03:128396-130327(+)|627	CDD:226582	20.82	317	207	16	142	435	47	342	7.14E-12	64.8	COG4097	COG4097	Predicted ferric reductase
LN02_03970|LN02 Chr03:1284060-1286405(-)|730	CDD:224117	19.774	354	236	9	48	364	660	1002	8.88E-08	52.8	COG1196	Smc	Chromosome segregation ATPases
LN02_04226|LN02 Chr03:3432465-3435777(-)|959	CDD:227944	29.145	971	641	25	4	957	3	943	2.49E-149	464	COG5657	CSE1	CAS/CSE protein involved in chromosome segregation
LN02_04290|LN02 Chr03:3678680-3679739(-)|258	CDD:223295	28.163	245	142	8	23	242	2	237	9.57E-35	122	COG0217	COG0217	Uncharacterized conserved protein
LN02_04354|LN02 Chr03:3890311-3892365(-)|664	CDD:224117	20.482	166	123	2	465	630	354	510	3.79E-07	50.5	COG1196	Smc	Chromosome segregation ATPases
LN02_04482|LN02 Chr03:4296267-4298688(+)|578	CDD:223395	29.389	507	322	13	54	556	42	516	9.05E-104	320	COG0318	CaiC	Acyl-CoA synthetases (AMP-forming)/AMP-acid ligases II
LN02_04546|LN02 Chr03:4518372-4522788(-)|1048	CDD:227943	29.893	1027	657	14	2	1024	1	968	0	708	COG5656	SXM1	Importin, protein involved in nuclear import

LN02_04738∣LN02 Chr03:5224474-5226503(+)∣582	CDD:223587	38.025	405	218	9	98	496	31	408	2.90E-127	380	COG0513	SrmB	Superfamily II DNA and RNA helicases
LN02_04866∣LN02 Chr03:5818207-5819958(+)∣467	CDD:223944	37.607	468	283	3	2	467	12	472	8.86E-152	437	COG1012	PutA	NAD-dependent aldehyde dehydrogenases
LN02_04994∣LN02 Chr04:297646-299809(+)∣679	CDD:227547	40.548	365	188	8	1	357	1	344	3.87E-113	344	COG5222	COG5222	Uncharacterized conserved protein, contains RING Zn-finger
LN02_05058∣LN02 Chr04:484390-484997(+)∣98	CDD:227552	47.191	89	46	1	9	96	12	100	1.06E-30	101	COG5227	SMT3	Ubiquitin-like protein (sentrin)
LN02_05122∣LN02 Chr04:685306-686274(+)∣322	CDD:227609	18.644	295	155	6	7	295	13	228	4.24E-16	74.4	COG5285	COG5285	Protein involved in biosynthesis of mitomycin antibiotics/polyketide fumonisin
LN02_05314∣LN02 Chr04:1343509-1344730(+)∣302	CDD:223334	31.056	161	73	4	14	174	1	123	1.13E-30	109	COG0256	RplR	Ribosomal protein L18
LN02_05378∣LN02 Chr04:1541742-1546224(+)∣1448	CDD:224117	21.892	740	505	16	394	1083	290	1006	1.66E-23	105	COG1196	Smc	Chromosome segregation ATPases
LN02_05442∣LN02 Chr04:1771323-1772937(-)∣381	CDD:223549	43.966	348	178	8	48	381	1	345	8.02E-152	429	COG0473	LeuB	Isocitrate/isopropylmalate dehydrogenase
LN02_05570∣LN02 Chr04:2483607-2486150(-)∣703	CDD:223373	29.022	634	415	16	70	696	23	628	1.23E-140	423	COG0296	GlgB	1,4-alpha-glucan branching enzyme
LN02_05698∣LN02 Chr04:2914567-2916177(+)∣433	CDD:224143	50.594	421	168	4	37	433	2	406	0	545	COG1222	RPT1	ATP-dependent 26S proteasome regulatory subunit
LN02_05826∣LN02 Chr04:3334010-3335763(+)∣537	CDD:223176	31.613	155	106	0	376	530	27	181	5.07E-24	96.1	COG0098	RpsE	Ribosomal protein S5
LN02_05954∣LN02 Chr04:3809882-3811655(+)∣481	CDD:223190	48.252	429	198	4	14	441	2	407	0	610	COG0112	GlyA	Glycine/serine hydroxymethyltransferase
LN02_06146∣LN02 Chr04:4416158-4418078(-)∣551	CDD:223535	34.717	530	319	14	27	545	1	514	6.19E-176	504	COG0459	GroL	Chaperonin GroEL (HSP60 family)
LN02_06274∣LN02 Chr04:4861488-4864703(-)∣1071	CDD:224117	19.217	281	223	1	674	950	681	961	5.37E-07	50.9	COG1196	Smc	Chromosome segregation ATPases
LN02_06274∣LN02 Chr04:4861488-4864703(-)∣1071	CDD:224117	15.882	850	616	19	18	829	179	967	2.41E-05	45.5	COG1196	Smc	Chromosome segregation ATPases

LN02_06530|LN02 Chr04:5744813-5746508(+)|499	CDD:225177	20.408	294	201	6	122	383	136	428	4.60E-11	61.8	COG2268	COG2268	Uncharacterized protein conserved in bacteria
LN02_06658|LN02 Chr05:280081-282155(+)|536	CDD:225035	29.73	185	93	6	316	498	237	386	7.71E-13	67.5	COG2124	CypX	Cytochrome P450
LN02_06722|LN02 Chr05:597095-599410(-)|530	CDD:223589	28.013	307	190	9	19	299	1	302	6.47E-49	171	COG0515	SPS1	Serine/threonine protein kinase
LN02_06786|LN02 Chr05:813440-817190(-)|1223	CDD:227680	23.377	77	54	1	913	984	175	251	2.00E-04	42.5	COG5391	COG5391	Phox homology (PX) domain protein
LN02_06914|LN02 Chr05:1305639-1307263(+)|482	CDD:223591	43.478	46	25	1	279	324	8	52	6.82E-06	42.5	COG0517	COG0517	FOG: CBS domain
LN02_06914|LN02 Chr05:1305639-1307263(+)|482	CDD:223591	23.276	116	75	5	210	322	12	116	1.73E-05	41.3	COG0517	COG0517	FOG: CBS domain
LN02_06914|LN02 Chr05:1305639-1307263(+)|482	CDD:224173	16.981	106	67	3	279	382	217	303	3.02E-04	40	COG1253	TlyC	Hemolysins and related proteins containing CBS domains
LN02_07106|LN02 Chr05:2586908-2587573(+)|145	CDD:227890	27.273	143	82	1	3	145	16	136	1.79E-27	96.5	COG5603	TRS20	Subunit of TRAPP, an ER-Golgi tethering complex
LN02_07170|LN02 Chr05:2800158-2801804(+)|496	CDD:223238	26.971	482	291	14	38	496	3	446	3.24E-108	326	COG0160	GabT	4-aminobutyrate aminotransferase and related aminotransferases
LN02_07298|LN02 Chr05:3254469-3257553(-)|993	CDD:223627	27.187	640	325	14	390	991	328	864	1.78E-91	308	COG0553	HepA	Superfamily II DNA/RNA helicases, SNF2 family
LN02_07490|LN02 Chr05:3934853-3936718(+)|569	CDD:227598	28.804	184	119	3	58	240	63	235	8.32E-36	133	COG5273	COG5273	Uncharacterized protein containing DHHC-type Zn finger
LN02_07810|LN02 Chr05:5357929-5360216(-)|667	CDD:227539	26.917	665	389	18	13	666	2	580	6.31E-110	341	COG5214	POL12	DNA polymerase alpha-primase complex, polymerase-associated subunit B
LN02_07938|LN02 Chr05:5737741-5738698(-)|262	CDD:223959	37.255	255	152	6	10	261	1	250	7.02E-58	182	COG1028	FabG	Dehydrogenases with different specificities (related to short-chain alcohol dehydrogenases)
LN02_08450|LN02 Chr06:2650872-2652226(+)|397	CDD:223109	39.13	230	131	3	37	260	2	228	8.73E-90	269	COG0030	KsgA	Dimethyladenosine transferase (rRNA methylation)
LN02_08514|LN02 Chr06:2843491-2844387(+)|236	CDD:224025	30.244	205	107	5	5	191	3	189	3.58E-29	106	COG1100	COG1100	GTPase SAR1 and related small G proteins

LN02_08578 ǀ LN02 Chr06:3063856-3065219(-) ǀ 370	CDD:225779	17.925	318	231	11	62	356	26	336	2.32E-16	76.4	COG3239	DesA	Fatty acid desaturase
LN02_08706 ǀ LN02 Chr06:3643386-3644333(-) ǀ 136	CDD:224915	39.286	112	62	1	8	119	2	107	4.48E-43	135	COG2004	RPS24A	Ribosomal protein S24E
LN02_08770 ǀ LN02 Chr07:298453-299135(+) ǀ 134	CDD:223314	52.542	59	28	0	72	130	20	78	6.35E-19	72.7	COG0236	AcpP	Acyl carrier protein
LN02_08834 ǀ LN02 Chr07:485832-487852(-) ǀ 606	CDD:223671	25.385	260	158	14	334	593	98	321	6.75E-25	102	COG0598	CorA	Mg2+ and Co2+ transporters
LN02_08962 ǀ LN02 Chr07:973064-976086(-) ǀ 989	CDD:227928	24.837	153	95	6	845	978	161	312	1.56E-06	48.7	COG5641	GAT1	GATA Zn-finger-containing transcription factor
LN02_08962 ǀ LN02 Chr07:973064-976086(-) ǀ 989	CDD:225112	22.034	118	76	3	317	432	121	224	1.07E-04	41.8	COG2202	AtoS	FOG: PAS/PAC domain
LN02_09090 ǀ LN02 Chr07:1389755-1391200(+) ǀ 409	CDD:227488	44.498	418	215	4	7	408	5	421	5.25E-143	411	COG5159	RPN6	26S proteasome regulatory complex component
LN02_09154 ǀ LN02 Chr07:1584309-1585940(-) ǀ 474	CDD:224145	53.915	447	198	3	9	449	6	450	0	636	COG1224	TIP49	DNA helicase TIP49, TBP-interacting protein
LN02_09218 ǀ LN02 Chr07:1778361-1781204(+) ǀ 911	CDD:224117	16.749	203	150	3	498	689	231	425	2.28E-08	55.1	COG1196	Smc	Chromosome segregation ATPases
LN02_09282 ǀ LN02 Chr07:2027793-2028237(-) ǀ 128	CDD:223410	51.064	47	21	2	64	110	2	46	2.31E-11	52.3	COG0333	RpmF	Ribosomal protein L32
LN02_00075 ǀ LN02 Chr01:400487-403788(-) ǀ 1082	CDD:227881	25.886	734	467	20	46	731	22	726	3.28E-109	357	COG5594	COG5594	Uncharacterized integral membrane protein
LN02_00331 ǀ LN02 Chr01:1800490-1803021(-) ǀ 813	CDD:227278	19.186	172	113	6	194	354	180	336	8.57E-05	42.8	COG4942	COG4942	Membrane-bound metallopeptidase
LN02_00971 ǀ LN02 Chr01:3967048-3968088(+) ǀ 315	CDD:227412	38.182	220	106	2	81	299	37	227	3.14E-56	179	COG5080	YIP1	Rab GTPase interacting factor, Golgi membrane protein
LN02_01035 ǀ LN02 Chr01:4171005-4172518(+) ǀ 394	CDD:227520	29.358	218	130	9	30	227	30	243	6.46E-13	66.6	COG5193	LHP1	La protein, small RNA-binding pol III transcript stabilizing protein and related La-motif-containing proteins involved in translation

LN02_01227∣LN02 Chr01:4944313-4946127(+)∣604	CDD:223610	25.771	551	182	10	62	603	1	333	2.44E-60	202	COG0536	Obg	Predicted GTPase
LN02_01483∣LN02 Chr01:5960734-5962665(-)∣545	CDD:223515	20.803	274	190	7	273	542	129	379	1.15E-25	105	COG0438	RfaG	Glycosyltransferase
LN02_01611∣LN02 Chr01:6476430-6478822(+)∣727	CDD:223600	31.884	69	44	1	286	351	36	104	5.70E-09	52.2	COG0526	TrxA	Thiol-disulfide isomerase and thioredoxins
LN02_01611∣LN02 Chr01:6476430-6478822(+)∣727	CDD:223600	23.81	126	85	3	33	158	8	122	1.11E-04	39.9	COG0526	TrxA	Thiol-disulfide isomerase and thioredoxins
LN02_01675∣LN02 Chr01:6714128-6716364(-)∣692	CDD:223446	29.968	634	351	17	60	691	45	587	9.61E-153	452	COG0369	CysJ	Sulfite reductase, alpha subunit (flavoprotein)
LN02_01803∣LN02 Chr01:7078148-7079199(-)∣139	CDD:223171	42.4	125	64	5	18	138	1	121	5.40E-49	150	COG0093	RplN	Ribosomal protein L14
LN02_01867∣LN02 Chr01:7256459-7267254(-)∣2962	CDD:227889	28.158	1243	665	32	1687	2898	72	1117	0	656	COG5602	SIN3	Histone deacetylase complex, SIN3 component
LN02_02059∣LN02 Chr02:488022-489659(-)∣504	CDD:223232	41.935	465	186	12	54	504	79	473	2.07E-98	302	COG0154	GatA	Asp-tRNAAsn/Glu-tRNAGln amidotransferase A subunit and related amidases
LN02_02251∣LN02 Chr02:1122976-1125117(+)∣713	CDD:225043	22.2	509	295	15	165	673	39	446	6.65E-52	183	COG2132	SufI	Putative multicopper oxidases
LN02_02443∣LN02 Chr02:1830867-1832680(+)∣496	CDD:227377	27.525	396	232	14	7	392	5	355	3.49E-53	182	COG5044	MRS6	RAB proteins geranylgeranyltransferase component A (RAB escort protein)
LN02_02507∣LN02 Chr02:2053988-2054825(-)∣224	CDD:223600	33.824	68	40	3	22	86	34	99	2.87E-14	64.5	COG0526	TrxA	Thiol-disulfide isomerase and thioredoxins
LN02_02507∣LN02 Chr02:2053988-2054825(-)∣224	CDD:226685	30.952	42	27	1	2	41	454	495	5.54E-04	37.4	COG4232	COG4232	Thiol:disulfide interchange protein
LN02_02699∣LN02 Chr02:2718928-2720210(-)∣274	CDD:227455	29.808	104	62	5	177	273	22	121	7.67E-13	61.9	COG5126	FRQ1	Ca2+-binding protein (EF-Hand superfamily)
LN02_02699∣LN02 Chr02:2718928-2720210(-)∣274	CDD:227455	24.812	133	91	3	114	239	26	156	1.69E-11	58.5	COG5126	FRQ1	Ca2+-binding protein (EF-Hand superfamily)

LN02_02763∣LN02 Chr02:2882875-2883828(+)∣205	CDD:224869	30.864	81	41	1	7	87	14	79	1.05E-12	58.5	COG1958	LSM1	Small nuclear ribonucleoprotein (snRNP) homolog
LN02_02827∣LN02 Chr02:3141245-3143131(+)∣607	CDD:224391	21.981	414	231	10	142	552	16	340	2.73E-41	150	COG1474	CDC6	Cdc6-related protein, AAA superfamily ATPase
LN02_03083∣LN02 Chr02:4037538-4039441(-)∣572	CDD:223354	34.81	474	282	10	107	572	2	456	1.18E-115	348	COG0277	GlcD	FAD/FMN-containing dehydrogenases
LN02_03275∣LN02 Chr02:4711891-4713076(-)∣315	CDD:224503	22.222	288	165	15	19	301	4	237	1.42E-19	83.2	COG1587	HemD	Uroporphyrinogen-III synthase
LN02_03403∣LN02 Chr02:5105896-5107051(+)∣322	CDD:227122	27.044	159	87	6	77	217	313	460	5.04E-05	41.6	COG4783	COG4783	Putative Zn-dependent protease, contains TPR repeats
LN02_03467∣LN02 Chr02:5693284-5698022(-)∣1334	CDD:223550	21.336	1003	618	28	334	1299	42	910	1.33E-160	503	COG0474	MgtA	Cation transport ATPase
LN02_03851∣LN02 Chr03:931832-933477(-)∣523	CDD:227381	19.685	127	95	3	85	205	317	442	5.33E-07	48.9	COG5048	COG5048	FOG: Zn-finger
LN02_03979∣LN02 Chr03:1317021-1318110(+)∣281	CDD:227533	43.32	247	99	9	7	229	37	266	8.67E-63	196	COG5208	HAP5	CCAAT-binding factor, subunit C
LN02_04043∣LN02 Chr03:1580596-1581103(-)∣145	CDD:223126	36.765	136	71	5	10	144	2	123	1.86E-46	144	COG0048	RpsL	Ribosomal protein S12
LN02_04171∣LN02 Chr03:2960408-2961853(-)∣355	CDD:223258	40.061	327	172	9	15	337	8	314	5.32E-108	316	COG0180	TrpS	Tryptophanyl-tRNA synthetase
LN02_04235∣LN02 Chr03:3460807-3462895(+)∣520	CDD:225371	22.717	449	288	7	69	515	3	394	4.97E-32	124	COG2814	AraJ	Arabinose efflux permease
LN02_04299∣LN02 Chr03:3711836-3712839(-)∣253	CDD:227311	31.298	131	88	2	92	222	63	191	2.58E-20	85.4	COG4977	COG4977	Transcriptional regulator containing an amidase domain and an AraC-type DNA-binding HTH domain
LN02_04363∣LN02 Chr03:3918424-3920100(+)∣540	CDD:223669	24.103	195	117	3	137	331	18	181	2.29E-07	49.6	COG0596	MhpC	Predicted hydrolases or acyltransferases (alpha/beta hydrolase superfamily)
LN02_04555∣LN02 Chr03:4559633-4559842(-)∣69	CDD:226090	43.333	60	31	1	4	63	12	68	2.10E-12	55.9	COG3560	FMR2	Predicted oxidoreductase related to nitroreductase

LN02_04619∣LN02 Chr03:4823730-4825749(+)∣628	CDD:223240	33.118	465	236	14	63	524	7	399	8.97E-98	302	COG0162	TyrS	Tyrosyl-tRNA synthetase
LN02_04683∣LN02 Chr03:5068208-5069894(-)∣476	CDD:223279	25.314	478	275	14	12	464	1	421	2.48E-89	276	COG0201	SecY	Preprotein translocase subunit SecY
LN02_04811∣LN02 Chr03:5487615-5489066(+)∣483	CDD:224513	16.914	337	179	16	105	428	5	253	3.30E-04	39.6	COG1597	LCB5	Sphingosine kinase and enzymes related to eukaryotic diacylglycerol kinase
LN02_05003∣LN02 Chr04:316490-317272(+)∣260	CDD:226168	42.795	229	104	3	32	260	3	204	2.40E-77	230	COG3642	COG3642	Mn2+-dependent serine/threonine protein kinase
LN02_05067∣LN02 Chr04:505015-506815(+)∣577	CDD:224250	23.077	195	104	10	115	280	391	568	3.21E-05	43.9	COG1331	COG1331	Highly conserved protein containing a thioredoxin domain
LN02_05131∣LN02 Chr04:705327-707139(-)∣516	CDD:225133	24.773	331	208	18	176	483	67	379	4.96E-11	61.5	COG2223	NarK	Nitrate/nitrite transporter
LN02_05195∣LN02 Chr04:928206-929482(+)∣404	CDD:225546	28.75	160	94	5	214	370	91	233	1.54E-17	79	COG3000	ERG3	Sterol desaturase
LN02_05259∣LN02 Chr04:1142666-1144475(+)∣578	CDD:226196	25.946	555	308	27	38	578	23	488	1.22E-59	203	COG3670	COG3670	Lignostilbene-alpha,beta-dioxygenase and related enzymes
LN02_05323∣LN02 Chr04:1374739-1378566(-)∣592	CDD:223796	30.645	124	84	1	181	302	89	212	1.49E-18	83.8	COG0724	COG0724	RNA-binding proteins (RRM domain)
LN02_05387∣LN02 Chr04:1580469-1581500(-)∣291	CDD:227311	20	265	134	5	12	276	9	195	2.71E-12	63.1	COG4977	COG4977	Transcriptional regulator containing an amidase domain and an AraC-type DNA-binding HTH domain
LN02_05643∣LN02 Chr04:2729636-2732620(+)∣937	CDD:227400	17.727	220	147	8	603	819	189	377	7.49E-04	40	COG5068	ARG80	Regulator of arginine metabolism and related MADS box-containing transcription factors
LN02_05707∣LN02 Chr04:2941691-2948677(+)∣2021	CDD:223627	20.821	999	535	22	922	1901	90	851	1.96E-74	265	COG0553	HepA	Superfamily II DNA/RNA helicases, SNF2 family
LN02_05707∣LN02 Chr04:2941691-2948677(+)∣2021	CDD:225499	20.635	126	94	2	483	605	44	166	7.10E-05	44.4	COG2948	VirB10	Type IV secretory pathway, VirB10 components
LN02_05963∣LN02 Chr04:3839105-3839892(-)∣194	CDD:225582	30.065	153	90	4	45	190	31	173	8.23E-24	89.8	COG3040	Blc	Bacterial lipocalin

LN02_06027|LN02 Chr04:4040885-4042110(-)|346	CDD:225201	26.032	315	204	8	16	328	153	440	1.32E-30	118	COG2319	COG2319	FOG: WD40 repeat
LN02_06219|LN02Chr04:4705479-4707303(+)|448	CDD:227356	54.147	434	196	1	1	431	1	434	0	726	COG5023	COG5023	Tubulin
LN02_06795|LN02Chr05:839611-841209(+)|532	CDD:223529	32.468	77	49	2	44	120	75	148	3.65E-10	58.9	COG0452	Dfp	Phosphopantothenoylcysteine synthetase/decarboxylase
LN02_06859|LN02Chr05:1058355-1059376(+)|266	CDD:223698	23.482	247	146	11	11	257	4	207	1.20E-24	95.3	COG0625	Gst	Glutathione S-transferase
LN02_07115|LN02Chr05:2612318-2613202(-)|232	CDD:224025	35.938	192	115	4	10	197	3	190	9.84E-45	146	COG1100	COG1100	GTPase SAR1 and related small G proteins
LN02_07179|LN02Chr05:2830235-2831650(-)|421	CDD:226580	20.513	78	62	0	71	148	7	84	3.59E-05	39.3	COG4095	COG4095	Uncharacterized conserved protein
LN02_07435|LN02Chr05:3778372-3780025(-)|339	CDD:223980	35.714	336	196	7	3	330	1	324	1.71E-91	273	COG1052	LdhA	Lactate dehydrogenase and related dehydrogenases
LN02_07499|LN02Chr05:3981477-3984924(-)|1099	CDD:223627	27.969	640	331	13	485	1097	328	864	2.23E-98	329	COG0553	HepA	Superfamily II DNA/RNA helicases, SNF2 family
LN02_07563|LN02Chr05:4167738-4170602(+)|830	CDD:227535	25.61	328	203	6	421	745	186	475	1.62E-52	188	COG5210	COG5210	GTPase-activating protein
LN02_07819|LN02Chr05:5387119-5390380(+)|1013	CDD:224706	21.392	388	234	18	262	643	122	444	2.74E-18	85.9	COG1793	CDC9	ATP-dependent DNA ligase
LN02_07883|LN02Chr05:5589600-5591338(+)|550	CDD:227419	24	325	159	13	19	332	5	252	1.09E-33	128	COG5087	RTT109	Uncharacterized conserved protein
LN02_07947|LN02Chr05:5766649-5768670(+)|550	CDD:223553	16.667	336	265	5	76	408	1	324	2.90E-05	43.1	COG0477	ProP	Permeases of the major facilitator superfamily
LN02_08075|LN02Chr06:1077756-1080752(-)|115	CDD:226325	32.143	84	47	3	38	113	95	176	2.82E-04	35.4	COG3803	COG3803	Uncharacterized protein conserved in bacteria
LN02_08203|LN02Chr06:1763932-1765739(+)|526	CDD:225371	23.35	197	137	5	36	229	4	189	1.29E-08	53.8	COG2814	AraJ	Arabinose efflux permease
LN02_08331|LN02Chr06:2140797-2141786(-)|189	CDD:225284	32.402	179	107	7	7	182	4	171	2.91E-40	133	COG2428	COG2428	Uncharacterized conserved protein

LN02_08395|LN02Chr06:2447120-2453880(-)|2213	CDD:223494	21.826	953	467	29	230	1174	101	783	2.84E-125	412	COG0417	PolB	DNA polymerase elongation subunit (family B)
LN02_08715|LN02Chr06:3667229-3668740(-)|503	CDD:223589	26.23	244	150	8	51	286	2	223	2.22E-43	155	COG0515	SPS1	Serine/threonine protein kinase
LN02_08779|LN02Chr07:323995-325528(-)|483	CDD:223589	20.26	385	187	9	95	464	1	280	4.41E-38	140	COG0515	SPS1	Serine/threonine protein kinase
LN02_08843|LN02Chr07:519473-521555(+)|565	CDD:225371	17.181	454	282	10	68	515	17	382	4.20E-14	71.1	COG2814	AraJ	Arabinose efflux permease
LN02_08907|LN02Chr07:763634-767759(-)|1193	CDD:227381	41.333	75	42	1	39	111	14	88	4.97E-09	57	COG5048	COG5048	FOG: Zn-finger
LN02_08971|LN02Chr07:1001382-1002573(+)|320	CDD:223103	36.646	161	99	2	130	289	2	160	6.05E-62	195	COG0024	Map	Methionine aminopeptidase
LN02_09227|LN02Chr07:1819874-1820496(-)|113	CDD:223619	46.847	111	51	2	2	112	102	204	4.68E-38	124	COG0545	FkpA	FKBP-type peptidyl-prolyl cis-trans isomerases 1
LN02_09291|LN02Chr07:2049124-2050179(-)|262	CDD:223402	41.633	245	115	12	17	253	3	227	9.80E-60	186	COG0325	COG0325	Predicted enzyme with a TIM-barrel fold
LN02_00716|LN02Chr01:3143442-3145012(+)|415	CDD:223261	34.815	405	222	12	27	408	1	386	1.90E-89	273	COG0183	PaaJ	Acetyl-CoA acetyltransferase
LN02_01036|LN02Chr01:4173144-4174284(-)|297	CDD:223559	33.929	280	157	6	11	288	6	259	2.91E-55	177	COG0483	SuhB	Archaeal fructose-1,6-bisphosphatase and related enzymes of inositol monophosphatase family
LN02_01100|LN02Chr01:4428122-4428981(-)|258	CDD:223592	35.099	151	93	4	73	220	41	189	1.67E-34	120	COG0518	GuaA	GMP synthase - Glutamine amidotransferase domain
LN02_01228|LN02Chr01:4946506-4949181(-)|835	CDD:223222	24.764	424	185	8	53	476	65	354	2.51E-64	216	COG0144	Sun	tRNA and rRNA cytosine-C5-methylases
LN02_01484|LN02Chr01:5963317-5964339(+)|340	CDD:223778	25.714	105	64	4	217	321	210	300	1.51E-04	40.1	COG0706	YidC	Preprotein translocase subunit YidC
LN02_01548|LN02Chr01:6225958-6228893(+)|764	CDD:224322	28.479	309	165	10	138	430	136	404	5.28E-28	115	COG1404	AprE	Subtilisin-like serine proteases

LN02_01612│LN02Chr01:6480107-6480746(+)│185	CDD:224168	24.793	121	74	5	52	167	40	148	2.56E-06	42.7	COG1247	COG1247	Sortase and related acyltransferases
LN02_02124│LN02Chr02:681076-682307(-)│378	CDD:223377	28.571	238	147	6	89	311	5	234	8.50E-42	144	COG0300	DltE	Short-chain dehydrogenases of various substrate specificities
LN02_02316│LN02Chr02:1375509-1378325(-)│854	CDD:226406	23.171	164	107	7	38	195	702	852	2.63E-05	44.9	COG3889	COG3889	Predicted solute binding protein
LN02_02380│LN02Chr02:1642036-1643977(-)│539	CDD:223590	35.683	227	81	12	295	521	1	162	7.18E-28	106	COG0516	GuaB	IMP dehydrogenase/GMP reductase
LN02_02380│LN02Chr02:1642036-1643977(-)│539	CDD:223590	33.684	95	63	0	36	130	6	100	1.78E-24	97.3	COG0516	GuaB	IMP dehydrogenase/GMP reductase
LN02_02380│LN02Chr02:1642036-1643977(-)│539	CDD:223591	32.743	113	66	4	132	238	8	116	5.75E-17	74	COG0517	COG0517	FOG: CBS domain
LN02_02380│LN02Chr02:1642036-1643977(-)│539	CDD:225979	25.472	106	71	3	189	286	248	353	1.24E-05	44.4	COG3448	COG3448	CBS-domain-containing membrane protein
LN02_02508│LN02Chr02:2055122-2056297(+)│343	CDD:225017	31.333	300	159	10	40	334	5	262	6.15E-56	181	COG2106	COG2106	Uncharacterized conserved protein
LN02_02636│LN02Chr02:2509240-2511520(-)│556	CDD:227596	30.508	59	36	1	72	125	4009	4067	3.53E-04	40.8	COG5271	MDN1	AAA ATPase containing von Willebrand factor type A (vWA) domain
LN02_02700│LN02Chr02:2721035-2722077(+)│213	CDD:226226	32.02	203	115	6	13	211	7	190	2.43E-42	138	COG3703	ChaC	Uncharacterized protein involved in cation transport
LN02_02764│LN02Chr02:2884377-2885395(-)│230	CDD:223560	26.316	114	65	4	8	119	5	101	8.86E-14	66.5	COG0484	DnaJ	DnaJ-class molecular chaperone with C-terminal Zn finger domain
LN02_02828│LN02Chr02:3143930-3145672(-)│580	CDD:223587	33.816	414	247	11	80	491	28	416	4.31E-91	287	COG0513	SrmB	Superfamily II DNA and RNA helicases
LN02_02892│LN02Chr02:3374801-3376613(+)│503	CDD:223944	44.28	472	253	5	24	493	3	466	0	568	COG1012	PutA	NAD-dependent aldehyde dehydrogenases
LN02_03020│LN02Chr02:3776230-3779904(-)│1174	CDD:227392	34.05	558	315	15	104	652	6	519	5.67E-110	353	COG5059	KIP1	Kinesin-like protein
LN02_03020│LN02Chr02:3776230-3779904(-)│1174	CDD:223496	16.117	515	372	14	454	936	157	643	8.41E-06	47.1	COG0419	SbcC	ATPase involved in DNA repair

LN02_03084∣LN02Chr02:4040004-4042284(-)∣661	CDD:227401	31.27	630	409	11	4	627	1	612	1.61E-142	426	COG5069	SAC6	Ca2+-binding actin-bundling protein fimbrin/plastin (EF-Hand superfamily)
LN02_03148∣LN02Chr02:4305225-4307122(-)∣562	CDD:227436	33.7	273	158	5	267	530	159	417	7.04E-57	194	COG5105	MIH1	Mitotic inducer, protein phosphatase
LN02_03212∣LN02Chr02:4510685-4512572(-)∣562	CDD:223444	20.979	572	331	22	1	553	1	470	8.63E-51	180	COG0367	AsnB	Asparagine synthase (glutamine-hydrolyzing)
LN02_03276∣LN02Chr02:4713502-4714202(+)∣197	CDD:227387	52.83	106	50	0	88	193	76	181	5.54E-49	155	COG5054	ERV1	Mitochondrial sulfhydryl oxidase involved in the biogenesis of cytosolic Fe/S proteins
LN02_03340∣LN02Chr02:4915923-4917947(+)∣674	CDD:223757	36.634	303	162	8	18	318	17	291	3.59E-80	253	COG0685	MetF	5,10-methylenetetrahydrofolate reductase
LN02_03724∣LN02Chr03:441754-445245(-)∣1077	CDD:227187	32.192	292	176	6	504	777	30	317	1.13E-60	209	COG4850	COG4850	Uncharacterized conserved protein
LN02_03852∣LN02Chr03:933793-934491(+)∣152	CDD:224672	32.394	71	47	1	71	140	1	71	1.43E-15	64.6	COG1758	RpoZ	DNA-directed RNA polymerase, subunit K/omega
LN02_03916∣LN02Chr03:1125335-1126199(-)∣165	CDD:223432	30.894	123	82	3	35	156	4	124	6.23E-26	93.5	COG0355	AtpC	F0F1-type ATP synthase, epsilon subunit (mitochondrial delta subunit)
LN02_04108∣LN02Chr03:2486299-2487211(+)∣265	CDD:227611	35	200	126	3	38	235	19	216	1.28E-34	123	COG5291	COG5291	Predicted membrane protein
LN02_04172∣LN02Chr03:2962279-2963529(+)∣343	CDD:223465	24.823	282	170	10	1	279	3	245	6.72E-29	109	COG0388	COG0388	Predicted amidohydrolase
LN02_04300∣LN02Chr03:3713708-3715770(-)∣494	CDD:223258	27.64	322	195	10	97	403	1	299	2.93E-82	255	COG0180	TrpS	Tryptophanyl-tRNA synthetase
LN02_04364∣LN02Chr03:3920333-3923950(-)∣929	CDD:224125	26.127	754	445	24	143	873	42	706	2.16E-76	263	COG1204	COG1204	Superfamily II helicase
LN02_04556∣LN02Chr03:4561202-4563064(-)∣620	CDD:223446	27.211	441	254	19	190	620	204	587	9.77E-66	223	COG0369	CysJ	Sulfite reductase, alpha subunit (flavoprotein)
LN02_04684∣LN02Chr03:5070444-5071910(+)∣464	CDD:223401	33.876	307	172	5	10	316	4	279	5.78E-85	260	COG0324	MiaA	tRNA delta(2)-isopentenylpyrophosphate transferase

LN02_04876∣LN02Chr03:5847750-5849741(-)∣511	CDD:227887	28.814	236	131	5	141	345	117	346	4.19E-26	107	COG5600	COG5600	Transcription-associated recombination protein
LN02_05004∣LN02Chr04:317716-318833(-)∣340	CDD:223729	28.226	124	69	3	143	265	82	186	2.97E-12	63	COG0656	ARA1	Aldo/keto reductases, related to diketogulonate reductase
LN02_05068∣LN02Chr04:507669-508548(+)∣255	CDD:223560	44.776	67	31	2	48	113	7	68	7.94E-15	70.3	COG0484	DnaJ	DnaJ-class molecular chaperone with C-terminal Zn finger domain
LN02_05132∣LN02Chr04:708996-711216(+)∣592	CDD:223727	21.41	383	261	12	7	384	2	349	5.76E-29	115	COG0654	UbiH	2-polyprenyl-6-methoxyphenol hydroxylase and related FAD-dependent oxidoreductases
LN02_05196∣LN02Chr04:930082-931130(+)∣265	CDD:223528	23.316	193	119	7	1	184	1	173	2.03E-08	51.1	COG0451	WcaG	Nucleoside-diphosphate-sugar epimerases
LN02_05324∣LN02Chr04:1379342-1381761(+)∣592	CDD:227535	21.667	420	239	11	193	572	125	494	2.74E-50	178	COG5210	COG5210	GTPase-activating protein
LN02_05388∣LN02Chr04:1582937-1586105(+)∣1019	CDD:224117	20.588	272	186	8	628	872	172	440	2.75E-12	68.2	COG1196	Smc	Chromosome segregation ATPases
LN02_05644∣LN02Chr04:2733548-2734867(-)∣439	CDD:224637	26.855	283	185	8	135	412	60	325	1.35E-39	142	COG1723	COG1723	Uncharacterized conserved protein
LN02_05900∣LN02Chr04:3555937-3559381(-)∣772	CDD:223903	39.279	499	284	6	293	772	34	532	3.42E-157	465	COG0833	LysP	Amino acid transporters
LN02_06220∣LN02Chr04:4707775-4708778(+)∣252	CDD:223175	34.536	194	99	3	48	241	1	166	1.88E-49	158	COG0097	RplF	Ribosomal protein L6P/L9E
LN02_06348∣LN02Chr04:5106530-5108839(-)∣658	CDD:225201	28.15	373	202	16	314	656	61	397	7.98E-30	120	COG2319	COG2319	FOG: WD40 repeat
LN02_06348∣LN02Chr04:5106530-5108839(-)∣658	CDD:224117	21.687	83	65	0	163	245	269	351	4.94E-04	40.5	COG1196	Smc	Chromosome segregation ATPases
LN02_06412∣LN02Chr04:5325219-5326811(+)∣455	CDD:223663	26.286	175	70	3	137	310	8	124	4.25E-27	103	COG0590	CumB	Cytosine/adenosine deaminases
LN02_06540∣LN02Chr04:5790475-5791268(-)∣226	CDD:223532	27.119	59	37	3	126	179	99	156	1.72E-04	38.1	COG0456	RimI	Acetyltransferases
LN02_06924∣LN02Chr05:1392517-1393028(+)∣135	CDD:227587	83.193	119	18	1	17	135	16	132	1.03E-60	180	COG5262	HTA1	Histone H2A

LN02_06988∣LN02Chr05:1883061-1884330(-)∣370	CDD:224037	22.034	295	166	10	1	259	472	738	9.38E-20	88.1	COG1112	COG1112	Superfamily I DNA and RNA helicases and helicase subunits
LN02_07180∣LN02Chr05:2832886-2834356(+)∣413	CDD:225373	34.375	256	130	7	152	404	59	279	1.22E-62	200	COG2816	NPY1	NTP pyrophosphohydrolases containing a Zn-finger, probably nucleic-acid-binding
LN02_07308∣LN02Chr05:3296026-3297197(+)∣298	CDD:227370	33.505	194	116	5	16	204	3	188	1.58E-31	115	COG5037	TOS9	Gluconate transport-inducing protein
LN02_07692∣LN02Chr05:4852241-4852910(-)∣152	CDD:227410	34.503	171	68	6	3	149	1	151	2.65E-35	117	COG5078	COG5078	Ubiquitin-protein ligase
LN02_07820∣LN02Chr05:5390558-5391955(-)∣349	CDD:226022	32.911	316	153	13	7	301	5	282	1.53E-53	176	COG3491	PcbC	Isopenicillin N synthase and related dioxygenases
LN02_07948∣LN02Chr05:5769135-5770643(+)∣428	CDD:223081	37.356	348	176	9	112	425	4	343	1.42E-106	316	COG0002	ArgC	Acetylglutamate semialdehyde dehydrogenase
LN02_08140∣LN02Chr06:1395322-1397928(+)∣868	CDD:223247	29.167	312	165	9	521	827	5	265	1.93E-58	198	COG0169	AroE	Shikimate 5-dehydrogenase
LN02_08268∣LN02Chr06:1943248-1944152(-)∣184	CDD:224867	44.444	171	76	4	14	184	11	162	1.85E-63	190	COG1956	COG1956	GAF domain-containing protein
LN02_08332∣LN02Chr06:2142996-2145038(+)∣623	CDD:225490	27.789	493	305	21	11	463	8	489	1.29E-93	294	COG2939	COG2939	Carboxypeptidase C (cathepsin A)
LN02_08460∣LN02Chr06:2683720-2685525(+)∣551	CDD:223185	39.683	315	130	6	231	545	1	255	1.78E-109	324	COG0107	HisF	Imidazoleglycerol-phosphate synthase
LN02_08460∣LN02Chr06:2683720-2685525(+)∣551	CDD:223196	42.381	210	113	4	1	208	1	204	2.93E-75	234	COG0118	HisH	Glutamine amidotransferase
LN02_08524∣LN02Chr06:2869078-2870582(+)∣446	CDD:223093	49.02	408	197	4	19	424	16	414	0	517	COG0014	ProA	Gamma-glutamyl phosphate reductase
LN02_08652∣LN02Chr06:3302262-3304314(+)∣598	CDD:223482	33.946	598	324	15	6	595	2	536	1.03E-141	419	COG0405	Ggt	Gamma-glutamyltransferase
LN02_08716∣LN02Chr06:3670568-3671946(-)∣390	CDD:227602	37.136	447	200	13	5	384	7	439	2.55E-122	358	COG5277	COG5277	Actin and related proteins
LN02_08780∣LN02Chr07:326162-330026(+)∣1172	CDD:227429	35.462	1159	677	15	33	1167	17	1128	0	999	COG5098	COG5098	Chromosome condensation complex Condensin, subunit D2

LN02_09100|LN02Chr07:1416148-1419037(-)|716	CDD:223249	25.545	321	156	9	342	662	15	252	9.91E-44	155	COG0171	NadE	NAD synthase
LN02_09100|LN02Chr07:1416148-1419037(-)|716	CDD:223465	26.667	300	187	9	5	301	3	272	8.95E-36	133	COG0388	COG0388	Predicted amidohydrolase
LN02_09228|LN02Chr07:1822595-1824743(+)|541	CDD:223535	35.227	528	320	13	12	529	3	518	2.06E-173	497	COG0459	GroL	Chaperonin GroEL (HSP60 family)
LN02_00333|LN02Chr01:1804230-1805923(+)|459	CDD:223648	19.578	332	194	5	69	399	1	260	8.49E-36	130	COG0575	CdsA	CDP-diglyceride synthetase
LN02_00461|LN02Chr01:2196975-2198726(-)|418	CDD:223589	27.217	327	205	8	23	340	2	304	3.30E-61	200	COG0515	SPS1	Serine/threonine protein kinase
LN02_00525|LN02Chr01:2379463-2381678(+)|583	CDD:223809	15.233	407	274	12	147	536	69	421	2.17E-05	43.8	COG0738	FucP	Fucose permease
LN02_00717|LN02Chr01:3145439-3147021(-)|277	CDD:225443	45.455	33	18	0	46	78	112	144	7.35E-05	40.4	COG2890	HemK	Methylase of polypeptide chain release factors
LN02_00717|LN02Chr01:3145439-3147021(-)|277	CDD:226608	17.073	82	55	3	40	109	40	120	1.33E-04	39.5	COG4123	COG4123	Predicted O-methyltransferase
LN02_00845|LN02Chr01:3567831-3569770(-)|516	CDD:225371	26.619	139	93	3	86	220	54	187	9.99E-14	70	COG2814	AraJ	Arabinose efflux permease
LN02_00845|LN02Chr01:3567831-3569770(-)|516	CDD:223553	17.708	288	234	2	45	329	4	291	4.64E-08	52	COG0477	ProP	Permeases of the major facilitator superfamily
LN02_00909|LN02Chr01:3739061-3740818(+)|484	CDD:225343	37.107	477	247	17	7	467	4	443	3.95E-130	382	COG2723	BglB	Beta-glucosidase/6-phospho-beta-glucosidase/beta-galactosidase
LN02_00973|LN02Chr01:3970439-3972240(+)|530	CDD:225180	20.657	426	319	9	72	492	24	435	4.54E-13	68.1	COG2271	UhpC	Sugar phosphate permease
LN02_01101|LN02Chr01:4430820-4431948(+)|334	CDD:224148	20.261	306	197	12	25	327	3	264	2.30E-23	95.5	COG1227	PPX1	Inorganic pyrophosphatase/exopolyphosphatase
LN02_01229|LN02Chr01:4949667-4953003(-)|1047	CDD:225201	19.57	465	276	13	292	747	74	449	4.18E-19	88.6	COG2319	COG2319	FOG: WD40 repeat
LN02_01229|LN02Chr01:4949667-4953003(-)|1047	CDD:225201	21.993	291	184	11	147	435	122	371	1.43E-11	65.1	COG2319	COG2319	FOG: WD40 repeat

LN02_01485|LN02Chr01:5964678-5965591(-)|269	CDD:227635	26.368	201	143	2	73	268	80	280	9.51E-15	69.5	COG5325	COG5325	t-SNARE complex subunit, syntaxin
LN02_01741|LN02Chr01:6898267-6898797(-)|85	CDD:224869	40.909	66	35	2	8	69	12	77	5.24E-14	58.5	COG1958	LSM1	Small nuclear ribonucleoprotein (snRNP) homolog
LN02_01997|LN02Chr02:260881-264206(+)|903	CDD:223550	29.348	920	580	20	24	902	27	917	0	585	COG0474	MgtA	Cation transport ATPase
LN02_02125|LN02Chr02:683100-685816(+)|809	CDD:225195	47.04	321	149	4	67	380	4	310	1.82E-100	310	COG2313	IndA	Uncharacterized enzyme involved in pigment biosynthesis
LN02_02125|LN02Chr02:683100-685816(+)|809	CDD:223598	19.647	397	228	11	413	804	1	311	7.32E-23	97.2	COG0524	RbsK	Sugar kinases, ribokinase family
LN02_02381|LN02Chr02:1644750-1647625(-)|853	CDD:225882	22.5	80	56	3	320	399	294	367	4.99E-04	40.6	COG3345	GalA	Alpha-galactosidase
LN02_02445|LN02Chr02:1836298-1837224(-)|308	CDD:223230	31.034	290	155	8	10	298	2	247	1.32E-82	247	COG0152	PurC	Phosphoribosylaminoimidazolesuccinocarboxamide (SAICAR) synthase
LN02_02509|LN02Chr02:2056678-2058000(-)|414	CDD:223397	53.716	296	127	5	93	388	21	306	2.33E-162	455	COG0320	LipA	Lipoate synthase
LN02_02573|LN02Chr02:2286723-2287473(+)|230	CDD:223384	46.919	211	103	2	1	211	1	202	3.34E-89	259	COG0307	RibC	Riboflavin synthase alpha chain
LN02_02701|LN02Chr02:2722980-2724063(-)|174	CDD:223796	42.5	80	46	0	2	81	116	195	4.48E-23	90.4	COG0724	COG0724	RNA-binding proteins (RRM domain)
LN02_02893|LN02Chr02:3377776-3378663(+)|261	CDD:223311	26.25	160	108	4	103	261	32	182	7.77E-10	54.1	COG0233	Frr	Ribosome recycling factor
LN02_03021|LN02Chr02:3782347-3784173(+)|608	CDD:225371	25.625	160	118	1	86	244	13	172	2.39E-12	66.1	COG2814	AraJ	Arabinose efflux permease
LN02_03021|LN02Chr02:3782347-3784173(+)|608	CDD:223553	22.961	331	244	6	87	406	3	333	8.62E-11	60.9	COG0477	ProP	Permeases of the major facilitator superfamily
LN02_03085|LN02Chr02:4043435-4044158(-)|217	CDD:223412	28.358	67	41	1	105	164	22	88	3.88E-07	44.5	COG0335	RplS	Ribosomal protein L19
LN02_03213|LN02Chr02:4513311-4514166(+)|250	CDD:225353	19.816	217	139	9	43	239	14	215	3.04E-10	55.7	COG2755	TesA	Lysophospholipase L1 and related esterases

LN02_03277|LN02Chr02:4715091-4717142(+)|575	CDD:226470	45.15	567	299	7	10	575	1	556	0	681	COG3961	COG3961	Pyruvate decarboxylase and related thiamine pyrophosphate-requiring enzymes
LN02_03405|LN02Chr02:5113412-5115703(+)|712	CDD:225606	28.369	141	92	2	255	386	117	257	1.09E-08	54.6	COG3064	TolA	Membrane protein involved in colicin uptake
LN02_03469|LN02Chr02:5701649-5702344(+)|187	CDD:224025	28	200	123	6	6	186	4	201	3.19E-30	107	COG1100	COG1100	GTPase SAR1 and related small G proteins
LN02_03725|LN02Chr03:445627-448036(-)|781	CDD:223587	32.017	481	318	6	264	741	39	513	4.87E-128	389	COG0513	SrmB	Superfamily II DNA and RNA helicases
LN02_03917|LN02Chr03:1134120-1136165(+)|681	CDD:225805	18.182	99	68	3	130	228	66	151	1.18E-04	41.5	COG3266	DamX	Uncharacterized protein conserved in bacteria
LN02_03981|LN02Chr03:1322412-1326152(+)|615	CDD:224802	43.981	216	105	2	380	595	11	210	1.14E-86	266	COG1890	RPS1A	Ribosomal protein S3AE
LN02_03981|LN02Chr03:1322412-1326152(+)|615	CDD:223878	31.765	170	96	6	180	343	37	192	9.15E-38	135	COG0807	RibA	GTP cyclohydrolase II
LN02_04109|LN02Chr03:2488420-2491118(-)|771	CDD:227380	56.824	762	321	4	9	770	2	755	0	1062	COG5047	SEC23	Vesicle coat complex COPII, subunit SEC23
LN02_04301|LN02Chr03:3716254-3717177(+)|307	CDD:224417	27.119	295	150	8	11	303	1	232	1.61E-55	177	COG1500	COG1500	Predicted exosome subunit
LN02_04365|LN02Chr03:3928698-3935436(+)|1648	CDD:227470	20.988	243	104	9	942	1169	196	365	1.20E-15	79.3	COG5141	COG5141	PHD zinc finger-containing protein
LN02_04493|LN02Chr03:4334183-4335310(+)|326	CDD:223796	19.487	195	146	5	133	325	73	258	4.60E-08	50.7	COG0724	COG0724	RNA-binding proteins (RRM domain)
LN02_04557|LN02Chr03:4566647-4567847(-)|325	CDD:224801	49.18	244	108	4	81	322	2	231	1.32E-118	338	COG1889	NOP1	Fibrillarin-like rRNA methylase
LN02_04621|LN02Chr03:4828884-4830266(+)|428	CDD:319244	32	75	48	2	12	86	1	72	7.16E-15	66.3	COG5272	UBI4	UBI4; linked to 3D-structure.
LN02_04877|LN02Chr03:5850276-5851680(-)|430	CDD:223261	41.22	410	204	16	36	427	2	392	7.20E-102	306	COG0183	PaaJ	Acetyl-CoA acetyltransferase
LN02_04941|LN02Chr03:6195980-6199045(-)|1021	CDD:226406	24.038	104	74	3	410	512	763	862	6.84E-05	43.7	COG3889	COG3889	Predicted solute binding protein

LN02_04941	LN02Chr03:6195980-6199045(-)	1021	CDD:226406	24.272	103	66	3	64	166	753	843	8.61E-05	43.3	COG3889	COG3889	Predicted solute binding protein
LN02_05005	LN02Chr04:320526-321384(+)	155	CDD:227363	48.611	144	74	0	2	145	1	144	2.78E-62	186	COG5030	APS2	Clathrin adaptor complex, small subunit
LN02_05133	LN02Chr04:711858-713749(+)	555	CDD:223699	27.92	351	206	14	209	546	80	396	4.58E-44	158	COG0626	MetC	Cystathionine beta-lyases/cystathionine gamma-synthases
LN02_05197	LN02Chr04:931327-933709(-)	600	CDD:223443	41.035	541	259	15	20	546	1	495	1.11E-146	430	COG0366	AmyA	Glycosidases
LN02_05581	LN02Chr04:2522378-2524842(-)	737	CDD:223201	35.754	358	177	11	64	416	7	316	1.38E-91	286	COG0123	AcuC	Deacetylases, including yeast histone deacetylase and acetoin utilization protein
LN02_05837	LN02Chr04:3365763-3370943(+)	1382	CDD:227721	19.005	442	246	22	254	619	84	489	6.86E-05	44	COG5434	PGU1	Endopygalactorunase
LN02_05965	LN02Chr04:3843222-3845831(-)	799	CDD:226809	22.917	144	92	6	10	139	147	285	6.52E-04	40	COG4372	COG4372	Uncharacterized protein conserved in bacteria with the myosin-like domain
LN02_06029	LN02Chr04:4043243-4045737(+)	771	CDD:225201	27.987	318	200	13	8	312	58	359	2.87E-30	122	COG2319	COG2319	FOG: WD40 repeat
LN02_06093	LN02Chr04:4244784-4246041(+)	310	CDD:223992	27.697	343	183	9	15	308	9	335	6.96E-71	220	COG1064	AdhP	Zn-dependent alcohol dehydrogenases
LN02_06157	LN02Chr04:4452296-4453601(+)	320	CDD:223423	25	144	96	4	170	309	2	137	4.59E-20	82.1	COG0346	GloA	Lactoylglutathione lyase and related lyases
LN02_06157	LN02Chr04:4452296-4453601(+)	320	CDD:223423	21.528	144	101	4	11	150	2	137	1.11E-16	72.9	COG0346	GloA	Lactoylglutathione lyase and related lyases
LN02_06221	LN02Chr04:4709018-4710106(-)	362	CDD:224139	23.496	349	181	9	9	348	3	274	9.49E-56	180	COG1218	CysQ	3'-Phosphoadenosine 5'-phosphosulfate (PAPS) 3'-phosphatase
LN02_06285	LN02Chr04:4909687-4913428(-)	1155	CDD:227371	18.978	685	428	29	230	895	205	781	6.85E-49	187	COG5038	COG5038	Ca2+-dependent lipid-binding protein, contains C2 domain
LN02_06349	LN02Chr04:5109486-5110484(+)	279	CDD:224521	26.389	72	37	1	152	223	21	76	2.34E-05	39.6	COG1605	PheA	Chorismate mutase
LN02_06605	LN02Chr05:99545-100056(-)	98	CDD:223143	38.235	34	20	1	4	36	95	128	6.58E-05	37.2	COG0065	LeuC	3-isopropylmalate dehydratase large subunit

LN02_06733∣LN02Chr05:626241-627679(+)∣399	CDD:223712	42.038	157	80	7	208	355	1	155	8.09E-39	134	COG0639	ApaH	Diadenosine tetraphosphatase and related serine/threonine protein phosphatases
LN02_06797∣LN02Chr05:848374-849157(-)∣140	CDD:227410	30.657	137	80	6	6	138	5	130	4.07E-33	111	COG5078	COG5078	Ubiquitin-protein ligase
LN02_06989∣LN02Chr05:1884465-1887720(-)∣883	CDD:223989	32.258	62	33	2	699	756	39	95	3.72E-04	40.9	COG1061	SSL2	DNA or RNA helicases of superfamily II
LN02_07437∣LN02Chr05:3782238-3782833(+)∣166	CDD:227410	46.296	162	74	1	1	162	1	149	5.04E-72	210	COG5078	COG5078	Ubiquitin-protein ligase
LN02_07501∣LN02Chr05:3988788-3989956(-)∣330	CDD:226200	20.53	151	83	7	92	213	82	224	1.40E-04	39.9	COG3675	COG3675	Predicted lipase
LN02_07565∣LN02Chr05:4171812-4172898(-)∣313	CDD:227458	49.6	250	104	5	3	251	2	230	1.80E-81	246	COG5129	MAK16	Nuclear protein with HMG-like acidic region
LN02_07693∣LN02Chr05:4854796-4856216(-)∣336	CDD:223677	36.826	334	197	8	1	328	1	326	3.80E-69	216	COG0604	Qor	NADPH:quinone reductase and related Zn-dependent oxidoreductases
LN02_07757∣LN02Chr05:5121211-5123499(-)∣687	CDD:226406	30.986	71	41	1	479	549	791	853	0.001	39.5	COG3889	COG3889	Predicted solute binding protein
LN02_07949∣LN02Chr05:5771630-5773154(-)∣483	CDD:223884	20.079	254	180	6	74	315	15	257	5.64E-04	39.2	COG0814	SdaC	Amino acid permeases
LN02_08141∣LN02Chr06:1398104-1399519(-)∣471	CDD:223131	26.255	259	174	9	183	434	19	267	4.09E-27	107	COG0053	MMT1	Predicted Co/Zn/Cd cation transporters
LN02_08205∣LN02Chr06:1767754-1769782(+)∣631	CDD:225371	23.113	212	151	3	139	346	10	213	7.20E-10	58.4	COG2814	AraJ	Arabinose efflux permease
LN02_08333∣LN02Chr06:2145766-2147122(+)∣337	CDD:227414	35.714	84	39	3	236	319	61	129	2.85E-14	67.6	COG5082	AIR1	Arginine methyltransferase-interacting protein, contains RING Zn-finger
LN02_08333∣LN02Chr06:2145766-2147122(+)∣337	CDD:227414	32.394	71	43	2	38	108	54	119	2.70E-12	61.8	COG5082	AIR1	Arginine methyltransferase-interacting protein, contains RING Zn-finger
LN02_08333∣LN02Chr06:2145766-2147122(+)∣337	CDD:227506	19.697	132	101	3	125	251	824	955	1.51E-04	40.4	COG5179	TAF1	Transcription initiation factor TFIID, subunit TAF1
LN02_08525∣LN02Chr06:2871051-2873150(-)∣600	CDD:224515	21.292	418	262	16	47	436	27	405	2.93E-29	117	COG1599	RFA1	Single-stranded DNA-binding replication protein A (RPA), large

														(70 kD) subunit and related ssDNA-binding proteins
LN02_08653|LN02Chr06:3304529-3305026(-)|143	CDD:223355	55.14	107	46	1	30	136	1	105	1.62E-58	174	COG0278	COG0278	Glutaredoxin-related protein
LN02_08781|LN02Chr07:331767-334331(+)|854	CDD:227517	46.774	186	72	4	150	335	18	176	1.73E-38	145	COG5190	FCP1	TFIIF-interacting CTD phosphatases, including NLI-interacting factor
LN02_08781|LN02Chr07:331767-334331(+)|854	CDD:223582	26.667	75	42	2	17	91	18	79	6.81E-05	43.2	COG0508	AceF	Pyruvate/2-oxoglutarate dehydrogenase complex, dihydrolipoamide acyltransferase (E2) component, and related enzymes
LN02_08845|LN02Chr07:525651-528632(-)|948	CDD:224149	22	400	266	16	151	535	29	397	1.92E-31	125	COG1228	HutI	Imidazolonepropionase and related amidohydrolases
LN02_08909|LN02Chr07:778535-779173(+)|212	CDD:225218	19.753	162	113	8	18	175	55	203	8.40E-09	50.6	COG2340	COG2340	Uncharacterized protein with SCP/PR1 domains
LN02_00014|LN02Chr01:191303-194177(+)|919	CDD:223669	30.894	123	78	4	528	648	9	126	1.47E-11	63.1	COG0596	MhpC	Predicted hydrolases or acyltransferases (alpha/beta hydrolase superfamily)
LN02_00014|LN02Chr01:191303-194177(+)|919	CDD:224423	27.358	106	64	5	775	875	499	596	7.46E-04	40.2	COG1506	DAP2	Dipeptidyl aminopeptidases/acylaminoacyl-peptidases
LN02_00078|LN02Chr01:411844-412799(+)|243	CDD:223539	21.545	246	172	5	7	243	1	234	5.10E-28	105	COG0463	WcaA	Glycosyltransferases involved in cell wall biogenesis
LN02_00462|LN02Chr01:2199791-2200545(-)|161	CDD:223471	34.395	157	85	5	3	159	1	139	3.16E-40	129	COG0394	Wzb	Protein-tyrosine-phosphatase
LN02_00590|LN02Chr01:2633552-2634977(-)|390	CDD:225201	21.488	121	92	2	267	386	152	270	3.20E-05	42.8	COG2319	COG2319	FOG: WD40 repeat
LN02_00654|LN02Chr01:2885391-2886780(-)|417	CDD:225297	23.529	85	34	3	137	221	108	161	8.31E-06	43.2	COG2453	CDC14	Predicted protein-tyrosine phosphatase
LN02_00718|LN02Chr01:3148914-3150939(+)|493	CDD:225035	23.058	412	254	15	72	472	33	392	3.01E-31	122	COG2124	CypX	Cytochrome P450
LN02_00846|LN02Chr01:3570842-3571474(-)|138	CDD:224300	22.222	117	91	0	22	138	3	119	1.97E-08	46.5	COG1382	GimC	Prefoldin, chaperonin cofactor

LN02_00974∣LN02Chr01:3973055-3974473(+)∣391	CDD:223737	16.827	416	254	16	1	376	1	364	2.18E-05	43	COG0665	DadA	Glycine/D-amino acid oxidases (deaminating)
LN02_01358∣LN02Chr01:5433039-5433743(-)∣234	CDD:223537	36.123	227	114	5	8	230	2	201	6.72E-55	172	COG0461	PyrE	Orotate phosphoribosyltransferase
LN02_01422∣LN02Chr01:5725770-5728234(-)∣769	CDD:227369	42.166	517	260	15	1	506	1	489	4.96E-162	476	COG5036	COG5036	SPX domain-containing protein involved in vacuolar polyphosphate accumulation
LN02_01422∣LN02Chr01:5725770-5728234(-)∣769	CDD:227589	41.176	119	68	1	626	744	4	120	3.40E-34	124	COG5264	VTC1	Vacuolar transporter chaperone
LN02_01550∣LN02Chr01:6232725-6235640(+)∣922	CDD:223327	31.101	881	527	27	15	880	9	824	0	599	COG0249	MutS	Mismatch repair ATPase (MutS family)
LN02_01614∣LN02Chr01:6483418-6484935(-)∣391	CDD:227557	31.674	221	136	4	112	325	28	240	3.20E-44	151	COG5232	SEC62	Preprotein translocase subunit Sec62
LN02_01806∣LN02Chr01:7089704-7091936(+)∣635	CDD:227358	42.188	64	37	0	271	334	337	400	3.54E-08	53.3	COG5025	COG5025	Transcription factor of the Forkhead/HNF3 family
LN02_01870∣LN02Chr01:7274222-7275030(-)∣197	CDD:227680	30.38	79	48	2	123	196	175	251	1.46E-09	53.6	COG5391	COG5391	Phox homology (PX) domain protein
LN02_01998∣LN02Chr02:264911-266455(-)∣514	CDD:224932	41.439	403	176	7	111	513	23	365	6.94E-130	379	COG2021	MET2	Homoserine acetyltransferase
LN02_02062∣LN02Chr02:494604-494944(-)∣83	CDD:224962	42.647	68	36	1	16	83	3	67	2.51E-24	84	COG2051	RPS27A	Ribosomal protein S27E
LN02_02126∣LN02Chr02:686241-688022(+)∣593	CDD:225201	18.283	361	250	11	233	592	126	442	6.19E-09	55.5	COG2319	COG2319	FOG: WD40 repeat
LN02_02382∣LN02Chr02:1648195-1649909(+)∣474	CDD:223685	32.488	434	275	4	38	464	16	438	1.98E-106	320	COG0612	PqqL	Predicted Zn-dependent peptidases
LN02_02446∣LN02Chr02:1838600-1840244(+)∣482	CDD:227403	44.632	475	224	3	4	477	1	437	6.76E-152	437	COG5071	RPN5	26S proteasome regulatory complex component
LN02_02510∣LN02Chr02:2058619-2060133(-)∣400	CDD:223240	21.552	348	248	10	8	331	5	351	5.26E-66	212	COG0162	TyrS	Tyrosyl-tRNA synthetase
LN02_02574∣LN02Chr02:2287652-2289421(-)∣589	CDD:224859	27.586	261	157	7	292	552	24	252	7.14E-38	137	COG1948	MUS81	ERCC4-type nuclease

LN02_02830|LN02Chr02:3151260-3152000(-)|246	CDD:224002	27.742	155	105	2	97	244	11	165	1.48E-19	80.1	COG1076	DjlA	DnaJ-domain-containing proteins 1
LN02_03150|LN02Chr02:4317640-4318747(-)|294	CDD:223711	35.075	268	138	6	6	271	1	234	8.80E-69	211	COG0638	PRE1	20S proteasome, alpha and beta subunits
LN02_03278|LN02Chr02:4719694-4721100(+)|329	CDD:224731	30.263	76	51	2	230	303	95	170	3.28E-05	40.8	COG1818	COG1818	Predicted RNA-binding protein, contains THUMP domain
LN02_03406|LN02Chr02:5116534-5117160(-)|208	CDD:225422	30.675	163	87	6	41	200	2	141	4.67E-31	108	COG2867	COG2867	Oligoketide cyclase/lipid transport protein
LN02_03470|LN02Chr02:5703241-5705043(+)|460	CDD:223250	39.683	441	252	8	1	439	1	429	3.66E-160	457	COG0172	SerS	Seryl-tRNA synthetase
LN02_03726|LN02Chr03:448585-449651(+)|245	CDD:227542	32.491	277	147	3	1	237	5	281	5.65E-65	202	COG5217	BIM1	Microtubule-binding protein involved in cell cycle control
LN02_03790|LN02Chr03:757725-759287(+)|489	CDD:223468	18.548	372	174	13	34	401	10	256	6.41E-14	69.6	COG0391	COG0391	Uncharacterized conserved protein
LN02_03918|LN02Chr03:1137505-1138571(+)|189	CDD:224025	26.056	142	98	2	16	151	1	141	5.19E-26	96.6	COG1100	COG1100	GTPase SAR1 and related small G proteins
LN02_03982|LN02Chr03:1326630-1327998(-)|311	CDD:223669	26.923	260	176	7	59	306	23	280	3.52E-24	96.6	COG0596	MhpC	Predicted hydrolases or acyltransferases (alpha/beta hydrolase superfamily)
LN02_04046|LN02Chr03:1587548-1588869(-)|265	CDD:227611	27.67	206	138	8	3	205	11	208	1.30E-18	80.8	COG5291	COG5291	Predicted membrane protein
LN02_04238|LN02Chr03:3468352-3469404(+)|350	CDD:223377	27.099	262	130	11	48	294	6	221	2.61E-33	121	COG0300	DltE	Short-chain dehydrogenases of various substrate specificities
LN02_04302|LN02Chr03:3717500-3718301(-)|156	CDD:223177	39.161	143	63	3	14	154	1	121	2.11E-43	136	COG0099	RpsM	Ribosomal protein S13
LN02_04366|LN02Chr03:3937162-3938598(+)|445	CDD:223589	19.95	401	192	8	31	429	1	274	1.45E-29	115	COG0515	SPS1	Serine/threonine protein kinase
LN02_04494|LN02Chr03:4336274-4338534(-)|209	CDD:224025	31.579	190	112	5	33	204	8	197	5.95E-39	130	COG1100	COG1100	GTPase SAR1 and related small G proteins
LN02_04558|LN02Chr03:4568565-4569657(+)|187	CDD:223169	29.53	149	72	2	8	155	4	120	4.67E-33	111	COG0091	RplV	Ribosomal protein L22

LN02_04814|LN02 Chr03:5493082-5494797(-)|417	CDD:223562	29.794	339	155	10	64	385	18	290	4.37E-54	185	COG0488	Uup	ATPase components of ABC transporters with duplicated ATPase domains
LN02_04878|LN02 Chr03:5852451-5853156(+)|151	CDD:227410	50	152	75	1	1	151	2	153	3.35E-75	218	COG5078	COG5078	Ubiquitin-protein ligase
LN02_05006|LN02 Chr04:323736-324837(+)|341	CDD:227379	30.846	201	109	6	35	234	81	252	4.38E-28	107	COG5046	MAF1	Protein involved in Mod5 protein sorting
LN02_05134|LN02Chr04:714250-716209(-)|565	CDD:225490	28.512	242	142	8	25	246	25	255	1.39E-31	125	COG2939	COG2939	Carboxypeptidase C (cathepsin A)
LN02_05326|LN02Chr04:1386060-1386881(+)|225	CDD:223454	58.48	171	71	0	55	225	2	172	8.96E-103	293	COG0377	NuoB	NADH:ubiquinone oxidoreductase 20 kD subunit and related Fe-S oxidoreductases
LN02_05582|LN02Chr04:2528795-2529844(-)|309	CDD:227483	48.718	312	128	5	1	309	1	283	8.95E-119	340	COG5154	BRX1	RNA-binding protein required for 60S ribosomal subunit biogenesis
LN02_05774|LN02Chr04:3143508-3145118(-)|536	CDD:223669	22.261	283	198	5	269	536	4	279	4.46E-23	96.2	COG0596	MhpC	Predicted hydrolases or acyltransferases (alpha/beta hydrolase superfamily)
LN02_05902|LN02 Chr04:3565285-3566220(-)|311	CDD:223729	48.12	266	132	3	38	301	6	267	2.17E-128	364	COG0656	ARA1	Aldo/keto reductases, related to diketogulonate reductase
LN02_06094|LN02 Chr04:4249343-4251277(+)|644	CDD:227507	24.476	143	87	6	481	610	473	607	5.52E-04	40.1	COG5180	PBP1	Protein interacting with poly(A)-binding protein
LN02_06158|LN02 Chr04:4454777-4456498(+)|573	CDD:223189	43.252	326	177	4	19	342	5	324	4.19E-104	314	COG0111	SerA	Phosphoglycerate dehydrogenase and related dehydrogenases
LN02_06286|LN02 Chr04:4914283-4915836(-)|517	CDD:225035	19.869	458	284	15	52	497	16	402	5.57E-24	100	COG2124	CypX	Cytochrome P450
LN02_06542|LN02Chr04:5794516-5795952(+)|433	CDD:225714	20.183	109	70	3	255	363	181	272	9.14E-06	44.3	COG3173	COG3173	Predicted aminoglycoside phosphotransferase
LN02_06606|LN02Chr05:101952-103701(+)|520	CDD:223608	26.733	404	288	4	77	473	11	413	6.77E-61	204	COG0534	NorM	Na+-driven multidrug efflux pump
LN02_06670|LN02Chr05:344449-347132(+)|870	CDD:224117	25.287	174	105	7	25	187	170	329	2.01E-07	52	COG1196	Smc	Chromosome segregation ATPases
LN02_06670|LN02Chr05:344449-347132(+)|870	CDD:223589	17.262	168	116	4	704	857	10	168	1.15E-04	42.4	COG0515	SPS1	Serine/threonine protein kinase

LN02_06734∣LN02Chr05:628225-629460(+)∣411	CDD:225318	19.634	382	184	17	33	409	78	341	2.01E-27	108	COG2520	COG2520	Predicted methyltransferase
LN02_06798∣LN02Chr05:849716-851110(+)∣400	CDD:223407	35.688	269	150	6	68	330	21	272	1.31E-48	164	COG0330	HflC	Membrane protease subunits, stomatin/prohibitin homologs
LN02_06926∣LN02Chr05:1394608-1395284(+)∣201	CDD:223582	27.586	116	83	1	42	157	3	117	8.46E-25	97.1	COG0508	AceF	Pyruvate/2-oxoglutarate dehydrogenase complex, dihydrolipoamide acyltransferase (E2) component, and related enzymes
LN02_06990∣LN02Chr05:1888906-1891614(+)∣801	CDD:223272	35.979	189	105	6	614	797	7	184	3.98E-35	129	COG0194	Gmk	Guanylate kinase
LN02_07118∣LN02Chr05:2626815-2633274(-)∣2033	CDD:227484	27.157	707	406	18	1341	2029	1005	1620	2.21E-111	389	COG5155	ESP1	Separase, a protease involved in sister chromatid separation
LN02_07182∣LN02Chr05:2838901-2840025(+)∣295	CDD:223669	21.306	291	182	9	27	293	15	282	3.25E-10	56.9	COG0596	MhpC	Predicted hydrolases or acyltransferases (alpha/beta hydrolase superfamily)
LN02_07246∣LN02Chr05:3080028-3082210(+)∣612	CDD:227270	20.07	573	348	25	50	597	58	545	6.69E-20	91.4	COG4934	COG4934	Predicted protease
LN02_07822∣LN02Chr05:5397645-5401991(-)∣1360	CDD:223631	30.793	643	367	17	542	1177	101	672	5.29E-119	385	COG0557	VacB	Exoribonuclease R
LN02_08078∣LN02Chr06:1117817-1121596(+)∣1259	CDD:226406	22.652	181	115	7	480	647	694	862	5.74E-04	41	COG3889	COG3889	Predicted solute binding protein
LN02_08142∣LN02Chr06:1401183-1402315(-)∣294	CDD:223959	37.247	247	149	5	48	289	1	246	2.06E-71	218	COG1028	FabG	Dehydrogenases with different specificities (related to short-chain alcohol dehydrogenases)
LN02_08206∣LN02Chr06:1771225-1775001(+)∣1211	CDD:227371	22.825	885	555	36	9	849	2	802	4.71E-107	362	COG5038	COG5038	Ca2+-dependent lipid-binding protein, contains C2 domain
LN02_08270∣LN02Chr06:1946730-1947243(+)∣126	CDD:225794	32.692	52	35	0	68	119	69	120	1.33E-06	41.6	COG3255	COG3255	Putative sterol carrier protein
LN02_08334∣LN02Chr06:2147914-2148975(+)∣332	CDD:223698	22.609	230	147	9	8	233	3	205	2.16E-09	53.7	COG0625	Gst	Glutathione S-transferase
LN02_08398∣LN02Chr06:2457825-2458844(+)∣339	CDD:224928	27.576	330	185	13	7	334	26	303	5.43E-53	174	COG2017	GalM	Galactose mutarotase and related enzymes

LN02_08462 I LN02Chr06:2688685-2691265(-) I 722	CDD:227651	41.228	114	66	1	12	124	5	118	1.68E-36	136	COG5347	COG5347	GTPase-activating protein that regulates ARFs (ADP-ribosylation factors), involved in ARF-mediated vesicular transport
LN02_08526 I LN02Chr06:2873642-2874898(+) I 319	CDD:223489	21.778	225	135	6	98	319	47	233	1.37E-17	77.4	COG0412	COG0412	Dienelactone hydrolase and related enzymes
LN02_08654 I LN02Chr06:3305350-3306099(+) I 249	CDD:223529	30.622	209	113	9	31	239	4	180	4.53E-30	112	COG0452	Dfp	Phosphopantothenoylcysteine synthetase/decarboxylase
LN02_09038 I LN02Chr07:1214266-1215619(-) I 372	CDD:225136	21	200	105	6	115	282	42	220	8.46E-09	52.7	COG2226	UbiE	Methylase involved in ubiquinone/menaquinone biosynthesis
LN02_09166 I LN02Chr07:1617396-1621611(+) I 706	CDD:223535	42.991	535	267	13	29	543	2	518	1.77E-171	498	COG0459	GroL	Chaperonin GroEL (HSP60 family)
LN02_00143 I LN02Chr01:682757-687706(+) I 1637	CDD:226406	22.652	181	112	7	467	631	694	862	1.05E-04	44.1	COG3889	COG3889	Predicted solute binding protein
LN02_00335 I LN02Chr01:1813439-1814410(+) I 323	CDD:223574	26.271	118	75	3	68	174	40	156	1.48E-05	43	COG0500	SmtA	SAM-dependent methyltransferases
LN02_00399 I LN02Chr01:2022397-2023373(+) I 287	CDD:223198	34.599	237	123	7	9	245	4	208	2.93E-63	196	COG0120	RpiA	Ribose 5-phosphate isomerase
LN02_00527 I LN02Chr01:2382763-2384728(+) I 426	CDD:224365	48.354	395	199	4	33	424	3	395	0	541	COG1448	TyrB	Aspartate/tyrosine/aromatic aminotransferase
LN02_00591 I LN02Chr01:2636452-2638440(+) I 627	CDD:224025	23.383	201	137	6	1	185	3	202	4.16E-21	89.2	COG1100	COG1100	GTPase SAR1 and related small G proteins
LN02_00591 I LN02Chr01:2636452-2638440(+) I 627	CDD:224025	21.488	121	81	4	425	537	8	122	1.14E-04	41.1	COG1100	COG1100	GTPase SAR1 and related small G proteins
LN02_00719 I LN02Chr01:3151689-3153252(+) I 417	CDD:223091	26.442	416	254	16	3	413	1	369	4.66E-96	289	COG0012	COG0012	Predicted GTPase, probable translation factor
LN02_00783 I LN02Chr01:3402274-3404748(+) I 580	CDD:224729	22.506	431	225	20	138	562	9	336	3.72E-16	77	COG1816	Add	Adenosine deaminase
LN02_00911 I LN02Chr01:3747635-3749518(+) I 582	CDD:223808	30.233	516	307	13	35	531	22	503	5.15E-91	287	COG0737	UshA	5'-nucleotidase/2',3'-cyclic phosphodiesterase and related esterases

LN02_01103∣LN02 Chr01:4435094-4439434(+)∣1411	CDD:227439	32.168	1144	674	23	136	1254	28	1094	0	757	COG5108	RPO41	Mitochondrial DNA-directed RNA polymerase
LN02_01295∣LN02 Chr01:5174883-5177197(+)∣692	CDD:223251	33.07	632	341	23	70	687	19	582	5.59E-139	416	COG0173	AspS	Aspartyl-tRNA synthetase
LN02_01423∣LN02 Chr01:5730690-5731523(+)∣200	CDD:223695	28.902	173	109	6	4	172	3	165	1.32E-26	97.4	COG0622	COG0622	Predicted phosphoesterase
LN02_01743∣LN02 Chr01:6900638-6902116(-)∣492	CDD:224983	33.58	405	260	5	5	406	6	404	2.07E-84	264	COG2072	TrkA	Predicted flavoprotein involved in K+ transport
LN02_01871∣LN02 Chr01:7276133-7277602(+)∣426	CDD:224843	36.493	422	206	11	3	424	2	361	7.38E-117	343	COG1932	SerC	Phosphoserine aminotransferase
LN02_02127∣LN02 Chr02:688519-689459(-)∣221	CDD:227418	33.333	207	131	5	2	207	4	204	1.02E-39	133	COG5086	COG5086	Uncharacterized conserved protein
LN02_02383∣LN02 Chr02:1650476-1651234(-)∣252	CDD:227425	37.931	145	67	4	48	192	10	131	1.60E-31	110	COG5094	TAF9	Transcription initiation factor TFIID, subunit TAF9 (also component of histone acetyltransferase SAGA)
LN02_02447∣LN02 Chr02:1840576-1841480(-)∣269	CDD:223477	23.03	165	83	10	93	242	56	191	9.63E-05	39.6	COG0400	COG0400	Predicted esterase
LN02_02511∣LN02 Chr02:2061611-2062646(+)∣293	CDD:223395	24.848	165	119	3	132	292	16	179	5.58E-20	86.7	COG0318	CaiC	Acyl-CoA synthetases (AMP-forming)/AMP-acid ligases II
LN02_02575∣LN02 Chr02:2291368-2293123(+)∣538	CDD:224034	23.185	496	250	19	50	538	91	462	9.97E-40	147	COG1109	{ManB	Phosphomannomutase
LN02_02703∣LN02 Chr02:2728858-2729923(-)∣326	CDD:225184	42.373	295	152	8	18	310	1	279	7.60E-78	236	COG2301	CitE	Citrate lyase beta subunit
LN02_02767∣LN02 Chr02:2903658-2906942(+)∣970	CDD:226406	26.415	106	66	4	428	523	739	842	2.73E-04	41.8	COG3889	COG3889	Predicted solute binding protein
LN02_02831∣LN02 Chr02:3152992-3155261(+)∣504	CDD:224223	33.425	365	213	8	116	474	5	345	8.17E-82	255	COG1304	idi	Isopentenyl diphosphate isomerase (BS_ypgA, MTH48 and related proteins)
LN02_02831∣LN02 Chr02:3152992-3155261(+)∣504	CDD:227599	47.143	70	37	0	1	70	48	117	1.27E-17	77.6	COG5274	CYB5	Cytochrome b involved in lipid metabolism

LN02_02895|LN02 Chr02:3381145-3382954(-)|540	CDD:223712	37.179	156	88	7	336	482	1	155	3.47E-32	118	COG0639	ApaH	Diadenosine tetraphosphatase and related serine/threonine protein phosphatases
LN02_03087|LN02 Chr02:4045933-4047171(-)|370	CDD:223110	47.604	313	144	5	34	342	3	299	4.49E-136	387	COG0031	CysK	Cysteine synthase
LN02_03407|LN02 Chr02:5117673-5121557(+)|1082	CDD:227427	18.957	211	164	4	67	273	30	237	7.58E-04	40.5	COG5096	COG5096	Vesicle coat complex, various subunits
LN02_03727|LN02 Chr03:450825-451922(+)|365	CDD:227680	26.891	119	72	4	5	110	133	249	4.03E-10	57.9	COG5391	COG5391	Phox homology (PX) domain protein
LN02_03855|LN02 Chr03:937338-938542(+)|290	CDD:223700	37.255	306	161	10	13	289	11	314	1.71E-74	228	COG0627	COG0627	Predicted esterase
LN02_04239|LN02 Chr03:3471587-3472863(+)|349	CDD:223774	17.155	239	157	10	9	236	4	212	9.96E-05	40.3	COG0702	COG0702	Predicted nucleoside-diphosphate-sugar epimerases
LN02_04367|LN02 Chr03:3939119-3942500(-)|1075	CDD:223460	28.406	1035	599	42	68	1075	24	943	0	551	COG0383	AMS1	Alpha-mannosidase
LN02_04495|LN02 Chr03:4340821-4342354(+)|458	CDD:224947	29.213	89	55	2	309	397	7	87	2.40E-10	54.3	COG2036	HHT1	Histones H3 and H4
LN02_04687|LN02 Chr03:5078429-5079175(+)|181	CDD:224920	29.771	131	76	3	56	179	2	123	2.16E-09	50.4	COG2009	SdhC	Succinate dehydrogenase/fumarate reductase, cytochrome b subunit
LN02_04751|LN02 Chr03:5275973-5278053(-)|543	CDD:225135	47.901	524	250	12	23	531	26	541	0	628	COG2225	AceB	Malate synthase
LN02_04879|LN02 Chr03:5854265-5857331(+)|936	CDD:227392	45.697	337	177	3	1	336	13	344	2.35E-99	320	COG5059	KIP1	Kinesin-like protein
LN02_04879|LN02 Chr03:5854265-5857331(+)|936	CDD:224117	21.306	291	214	5	430	716	658	937	1.54E-10	62	COG1196	Smc	Chromosome segregation ATPases
LN02_04879|LN02 Chr03:5854265-5857331(+)|936	CDD:224117	18.685	289	207	7	601	874	681	956	6.07E-08	53.6	COG1196	Smc	Chromosome segregation ATPases
LN02_05071|LN02 Chr04:514338-519437(+)|1670	CDD:224419	17.735	468	251	17	648	1110	57	395	3.80E-30	122	COG1502	Cls	Phosphatidylserine/phosphatidyl glycerophosphate/cardiolipin synthases and related enzymes
LN02_05263|LN02 Chr04:1154735-1155573(-)|223	CDD:227414	28.025	157	84	5	7	161	61	190	1.57E-18	77.2	COG5082	AIR1	Arginine methyltransferase-interacting protein, contains RING Zn-finger

LN02_05263|LN02 Chr04:1154735-1155573(-)|223	CDD:227414	41.538	65	27	2	130	194	63	116	8.20E-09	50.6	COG5082	AIR1	Arginine methyltransferase-interacting protein, contains RING Zn-finger
LN02_05327|LN02 Chr04:1388069-1391083(-)|837	CDD:227354	39.437	852	453	13	11	837	59	872	0	795	COG5021	HUL4	Ubiquitin-protein ligase
LN02_05583|LN02 Chr04:2530674-2531636(-)|212	CDD:224025	34.171	199	113	4	10	190	6	204	8.90E-45	145	COG1100	COG1100	GTPase SAR1 and related small G proteins
LN02_06095|LN02 Chr04:4253611-4255913(+)|730	CDD:223738	29.293	99	64	2	592	687	94	189	1.92E-13	67.5	COG0666	Arp	FOG: Ankyrin repeat
LN02_06159|LN02 Chr04:4457898-4459764(+)|527	CDD:224173	28.07	342	210	13	1	322	11	336	6.01E-55	187	COG1253	TlyC	Hemolysins and related proteins containing CBS domains
LN02_06287|LN02 Chr04:4917859-4918576(+)|203	CDD:224490	31.852	135	82	3	75	203	401	531	2.49E-19	82.4	COG1574	COG1574	Predicted metal-dependent hydrolase with the TIM-barrel fold
LN02_06351|LN02 Chr04:5112213-5114165(+)|595	CDD:227680	27.586	87	57	3	157	237	159	245	6.00E-05	42.9	COG5391	COG5391	Phox homology (PX) domain protein
LN02_06479|LN02Chr04:5531083-5535270(-)|710	CDD:223497	25.641	117	65	4	339	448	2	103	8.83E-08	51.8	COG0420	SbcD	DNA repair exonuclease
LN02_06607|LN02Chr05:104651-105735(+)|342	CDD:223528	26.486	185	103	6	10	189	3	159	8.60E-23	93.9	COG0451	WcaG	Nucleoside-diphosphate-sugar epimerases
LN02_06927|LN02Chr05:1395538-1395926(+)|113	CDD:223582	56.25	112	47	1	1	112	244	353	2.54E-44	145	COG0508	AceF	Pyruvate/2-oxoglutarate dehydrogenase complex, dihydrolipoamide acyltransferase (E2) component, and related enzymes
LN02_06991|LN02Chr05:1892291-1893667(-)|423	CDD:224517	25.21	119	84	2	16	134	29	142	2.72E-08	50.1	COG1601	GCD7	Translation initiation factor 2, beta subunit (eIF-2beta)/eIF-5 N-terminal domain
LN02_07183|LN02Chr05:2841095-2846135(+)|1485	CDD:224055	31.638	531	343	11	959	1483	51	567	2.85E-114	369	COG1132	MdlB	ABC-type multidrug transport system, ATPase and permease components
LN02_07183|LN02Chr05:2841095-2846135(+)|1485	CDD:224055	21.098	583	401	15	251	792	3	567	1.42E-59	213	COG1132	MdlB	ABC-type multidrug transport system, ATPase and permease components
LN02_07247|LN02Chr05:3083341-3085330(-)|582	CDD:223903	49.805	512	242	6	70	579	41	539	0	606	COG0833	LysP	Amino acid transporters
LN02_07503|LN02Chr05:3997906-4000895(+)|917	CDD:224389	27.926	376	218	10	127	491	55	388	8.86E-58	201	COG1472	BglX	Beta-glucosidase-related glycosidases

LN02_07567|LN02Chr05:4174327-4177092(-)|921	CDD:223225	26.327	452	238	9	460	910	90	447	4.23E-92	296	COG0147	TrpE	Anthranilate/para-aminobenzoate synthases component I
LN02_07567|LN02Chr05:4174327-4177092(-)|921	CDD:223586	30.739	257	111	13	39	294	1	191	3.16E-47	163	COG0512	PabA	Anthranilate/para-aminobenzoate synthases component II
LN02_07695|LN02Chr05:4879682-4882291(+)|792	CDD:226406	28.169	71	43	1	190	260	791	853	8.92E-04	39.8	COG3889	COG3889	Predicted solute binding protein
LN02_08143|LN02Chr06:1403154-1405454(+)|742	CDD:223824	51.073	466	222	2	65	530	37	496	0	664	COG0753	KatE	Catalase
LN02_08143|LN02Chr06:1403154-1405454(+)|742	CDD:223765	24.528	106	78	1	555	660	1	104	1.16E-05	43.7	COG0693	ThiJ	Putative intracellular protease/amidase
LN02_08271|LN02Chr06:1947493-1949067(-)|392	CDD:224981	26.722	363	199	14	53	391	16	335	1.62E-30	116	COG2070	COG2070	Dioxygenases related to 2-nitropropane dioxygenase
LN02_08527|LN02Chr06:2874972-2876051(-)|334	CDD:223677	37.126	334	195	7	1	330	1	323	1.88E-83	253	COG0604	Qor	NADPH:quinone reductase and related Zn-dependent oxidoreductases
LN02_08591|LN02Chr06:3102287-3103819(-)|380	CDD:227602	41.309	443	194	14	1	378	1	442	7.41E-123	359	COG5277	COG5277	Actin and related proteins
LN02_08655|LN02Chr06:3306345-3307198(+)|245	CDD:224117	15.707	191	151	5	47	230	709	896	7.16E-04	37.4	COG1196	Smc	Chromosome segregation ATPases
LN02_08975|LN02Chr07:1009474-1011002(+)|444	CDD:227598	25.738	237	137	7	62	275	59	279	5.91E-30	115	COG5273	COG5273	Uncharacterized protein containing DHHC-type Zn finger
LN02_09039|LN02Chr07:1216827-1218245(-)|400	CDD:223727	22.812	377	238	15	12	373	5	343	8.11E-27	107	COG0654	UbiH	2-polyprenyl-6-methoxyphenol hydroxylase and related FAD-dependent oxidoreductases
LN02_09103|LN02Chr07:1426405-1427844(-)|286	CDD:227569	39.437	71	35	3	196	266	4	66	9.31E-12	62.4	COG5244	NIP100	Dynactin complex subunit involved in mitotic spindle partitioning in anaphase B
LN02_09295|LN02Chr07:2059302-2061477(+)|610	CDD:225371	21.635	208	153	7	38	240	1	203	2.12E-10	59.9	COG2814	AraJ	Arabinose efflux permease
LN02_00336|LN02Chr01:1815400-1819356(-)|1290	CDD:223407	35.593	59	37	1	465	523	180	237	5.90E-04	40.5	COG0330	HflC	Membrane protease subunits, stomatin/prohibitin homologs
LN02_00464|LN02Chr01:2203131-2204524(+)|357	CDD:223161	36.975	357	164	10	1	356	1	297	1.99E-100	296	COG0083	ThrB	Homoserine kinase
LN02_00912|LN02Chr01:3750725-3751810(-)|361	CDD:223255	32.432	185	106	5	158	340	33	200	8.98E-40	137	COG0177	Nth	Predicted EndoIII-related endonuclease
LN02_00976|LN02Chr01:3978847-3979595(-)|236	CDD:227502	26.882	93	58	5	95	180	4	93	1.22E-07	48.9	COG5175	MOT2	Transcriptional repressor
LN02_01104|LN02Chr01:4439928-4441184(-)|418	CDD:223151	31.707	123	64	4	227	346	18	123	1.78E-19	80.8	COG0073	ARC1	EMAP domain
LN02_01104|LN02Chr01:4439928-4441184(-)|418	CDD:223698	19.802	101	70	3	1	101	109	198	6.66E-04	37.9	COG0625	Gst	Glutathione S-transferase
LN02_01168|LN02Chr01:4755450-4758796(-)|893	CDD:225837	25.194	258	167	7	18	274	2	234	4.20E-20	87.4	COG3300	COG3300	MHYT domain (predicted integral membrane sensor domain)
LN02_01296|LN02Chr01:5179226-5182666(+)|976	CDD:227495	26.946	167	114	3	475	639	367	527	1.34E-21	97.4	COG5166	COG5166	Uncharacterized conserved protein

LN02_01616|LN02Chr01:6486318-6487234(-)|237	CDD:223943	24.779	226	153	9	11	224	2	222	6.86E-29	106	COG1011	COG1011	Predicted hydrolase (HAD superfamily)
LN02_01680|LN02Chr01:6731542-6734987(+)|1116	CDD:223542	38.529	911	415	10	176	1085	7	773	0	1003	COG0466	Lon	ATP-dependent Lon protease, bacterial type
LN02_01744|LN02Chr01:6903368-6905379(+)|578	CDD:223533	21.757	239	174	4	239	468	47	281	1.92E-05	43.7	COG0457	NrfG	FOG: TPR repeat
LN02_02064|LN02Chr02:497168-498655(+)|495	CDD:227223	32.143	84	54	2	206	288	139	220	4.81E-07	48.8	COG4886	COG4886	Leucine-rich repeat (LRR) protein
LN02_02192|LN02Chr02:879696-880714(-)|285	CDD:223678	25.911	247	123	11	32	272	4	196	6.04E-29	106	COG0605	SodA	Superoxide dismutase
LN02_02256|LN02Chr02:1134356-1136278(-)|640	CDD:227820	19.869	458	222	14	25	467	54	381	5.18E-11	62	COG5533	UBP5	Ubiquitin C-terminal hydrolase
LN02_02256|LN02Chr02:1134356-1136278(-)|640	CDD:225689	27.692	65	46	1	483	546	86	150	1.81E-04	40.3	COG3147	DedD	Uncharacterized protein conserved in bacteria
LN02_02320|LN02Chr02:1416536-1419841(-)|546	CDD:223593	54.79	334	132	4	213	546	1	315	9.06E-179	503	COG0519	GuaA	GMP synthase, PP-ATPase domain/subunit
LN02_02320|LN02Chr02:1416536-1419841(-)|546	CDD:223592	38.424	203	112	9	17	212	2	198	3.03E-58	189	COG0518	GuaA	GMP synthase - Glutamine amidotransferase domain
LN02_02384|LN02Chr02:1651804-1654508(+)|858	CDD:223219	49.425	435	200	9	427	854	1	422	0	551	COG0141	HisD	Histidinol dehydrogenase
LN02_02384|LN02Chr02:1651804-1654508(+)|858	CDD:223217	47.312	93	46	3	203	292	16	108	6.01E-30	111	COG0139	HisI	Phosphoribosyl-AMP cyclohydrolase
LN02_02384|LN02Chr02:1651804-1654508(+)|858	CDD:223218	34.483	87	53	2	299	381	4	90	6.04E-20	82.3	COG0140	HisI	Phosphoribosyl-ATP pyrophosphohydrolase
LN02_02512|LN02Chr02:2062906-2064233(+)|352	CDD:223395	27.628	333	185	16	4	315	237	534	5.03E-36	134	COG0318	CaiC	Acyl-CoA synthetases (AMP-forming)/AMP-acid ligases II
LN02_02640|LN02Chr02:2522535-2523919(+)|391	CDD:224143	53.608	388	177	2	5	389	19	406	0	569	COG1222	RPT1	ATP-dependent 26S proteasome regulatory subunit
LN02_03152|LN02Chr02:4321960-4323314(-)|421	CDD:227563	36.145	415	232	6	10	421	4	388	5.95E-98	295	COG5238	RNA1	Ran GTPase-activating protein (RanGAP) involved in mRNA processing and transport
LN02_03216|LN02Chr02:4523367-4525790(-)|765	CDD:224839	37.006	708	403	10	53	758	25	691	0	612	COG1928	PMT1	Dolichyl-phosphate-mannose--protein O-mannosyl transferase
LN02_03920|LN02Chr03:1139703-1142215(+)|788	CDD:224666	21.708	281	173	10	369	639	8	251	8.92E-34	129	COG1752	RssA	Predicted esterase of the alpha-beta hydrolase superfamily
LN02_04048|LN02Chr03:1589947-1591271(-)|356	CDD:225369	28.467	274	168	5	39	289	9	277	7.42E-27	108	COG2812	DnaX	DNA polymerase III, gamma/tau subunits
LN02_04368|LN02Chr03:3945518-3948628(+)|1001	CDD:223327	30.942	892	497	14	92	978	17	794	0	608	COG0249	MutS	Mismatch repair ATPase (MutS family)
LN02_04496|LN02Chr03:4342489-4343836(-)|325	CDD:225883	36.965	257	145	7	74	323	6	252	4.44E-65	202	COG3346	COG3346	Uncharacterized conserved protein
LN02_04560|LN02Chr03:4573838-4575612(-)|556	CDD:223519	29.883	512	325	13	53	556	14	499	6.58E-153	445	COG0442	ProS	Prolyl-tRNA synthetase

LN02_04624∣LN02Chr03:4833745-4834691(+)∣281	CDD:225139	25.301	166	80	8	3	162	48	175	5.24E-09	53	COG2230	Cfa	Cyclopropane fatty acid synthase and related methyltransferases
LN02_04688∣LN02Chr03:5079626-5081157(-)∣469	CDD:227426	41.247	417	228	4	11	418	5	413	6.78E-131	383	COG5095	TAF6	Transcription initiation factor TFIID, subunit TAF6 (also component of histone acetyltransferase SAGA)
LN02_04752∣LN02Chr03:5279678-5283567(-)∣1183	CDD:223589	28.296	311	171	8	107	391	2	286	1.76E-51	183	COG0515	SPS1	Serine/threonine protein kinase
LN02_05072∣LN02Chr04:519874-520794(-)∣257	CDD:225492	34.872	195	109	3	63	257	28	204	3.41E-50	161	COG2941	CAT5	Ubiquinone biosynthesis protein COQ7
LN02_05328∣LN02Chr04:1393604-1394482(-)∣195	CDD:223796	25.197	127	89	2	35	155	74	200	2.72E-17	75.4	COG0724	COG0724	RNA-binding proteins (RRM domain)
LN02_05392∣LN02Chr04:1592568-1593767(+)∣399	CDD:227625	23.846	260	161	12	18	273	45	271	8.19E-24	97.6	COG5309	COG5309	Exo-beta-1,3-glucanase
LN02_05712∣LN02Chr04:2957974-2960772(+)∣768	CDD:225201	28.289	304	203	11	363	654	142	442	1.35E-39	149	COG2319	COG2319	FOG: WD40 repeat
LN02_05840∣LN02Chr04:3376831-3378702(+)∣449	CDD:227356	60.714	448	169	2	1	448	1	441	0	730	COG5023	COG5023	Tubulin
LN02_05904∣LN02Chr04:3575090-3576012(-)∣294	CDD:224630	21.277	141	82	5	145	285	49	160	6.26E-05	39.9	COG1716	COG1716	FOG: FHA domain
LN02_06032∣LN02Chr04:4049346-4051632(-)∣720	CDD:227625	25.993	277	161	8	441	704	60	305	9.31E-37	137	COG5309	COG5309	Exo-beta-1,3-glucanase
LN02_06160∣LN02Chr04:4460184-4461549(-)∣367	CDD:225333	18.77	309	192	17	8	295	7	277	1.21E-10	59.3	COG2706	COG2706	3-carboxymuconate cyclase
LN02_06288∣LN02Chr04:4919050-4919904(+)∣96	CDD:224869	37.662	77	42	3	15	91	7	77	2.94E-10	49.2	COG1958	LSM1	Small nuclear ribonucleoprotein (snRNP) homolog
LN02_06608∣LN02Chr05:106334-107384(-)∣279	CDD:223959	25.887	282	142	9	21	272	7	251	1.40E-23	93.7	COG1028	FabG	Dehydrogenases with different specificities (related to short-chain alcohol dehydrogenases)
LN02_06992∣LN02Chr05:1895145-1896168(-)∣210	CDD:225035	18.497	173	90	5	33	165	220	381	2.86E-08	50.1	COG2124	CypX	Cytochrome P450
LN02_07120∣LN02Chr05:2634533-2635717(-)∣394	CDD:223915	25	332	145	13	43	367	13	247	6.51E-33	121	COG0846	SIR2	NAD-dependent protein deacetylases, SIR2 family
LN02_07312∣LN02Chr05:3308939-3309745(+)∣238	CDD:224527	28.774	212	123	8	17	224	8	195	6.62E-36	123	COG1611	COG1611	Predicted Rossmann fold nucleotide-binding protein
LN02_07888∣LN02Chr05:5606783-5609185(+)∣412	CDD:223197	32.552	384	248	4	36	410	1	382	4.63E-126	367	COG0119	LeuA	Isopropylmalate/homocitrate/citramalate synthases
LN02_08272∣LN02Chr06:1949456-1950717(-)∣346	CDD:223739	40	335	175	10	10	340	1	313	6.57E-98	289	COG0667	Tas	Predicted oxidoreductases (related to aryl-alcohol dehydrogenases)
LN02_08528∣LN02Chr06:2876985-2880322(-)∣914	CDD:224136	19.308	347	228	10	357	694	115	418	4.38E-29	118	COG1215	COG1215	Glycosyltransferases, probably involved in cell wall biogenesis
LN02_08592∣LN02Chr06:3104557-3106987(+)∣678	CDD:223738	21.341	164	102	4	257	414	69	211	3.24E-07	49.1	COG0666	Arp	FOG: Ankyrin repeat
LN02_08592∣LN02Chr06:3104557-3106987(+)∣678	CDD:227061	26.277	137	91	4	487	613	256	392	4.34E-04	40.6	COG4717	COG4717	Uncharacterized conserved protein

LN02_08656 | LN02Chr06 :3307932-3309277(-) | 415	CDD:2231 93	32.803	314	166	12	91	400	12	284	7.90E-52	172	COG01 15	IlvE	Branched-chain amino acid aminotransferase/4-amino-4-deoxychorismate lyase
LN02_08976 | LN02Chr07 :1011307-1015125(-) | 1111	CDD:2273 55	45.158	919	488	10	31	939	51	963	0	1151	COG50 22	COG502 2	Myosin heavy chain
LN02_09040 | LN02Chr07 :1220039-1220991(-) | 266	CDD:2274 10	40.833	120	65	3	1	117	1	117	4.51E-44	143	COG50 78	COG507 8	Ubiquitin-protein ligase
LN02_09168 | LN02Chr07 :1623378-1625450(+) | 690	CDD:2232 22	40.96	354	187	8	258	607	18	353	6.09E-110	333	COG01 44	Sun	tRNA and rRNA cytosine-C5-methylases
LN02_09232 | LN02Chr07 :1861224-1863344(+) | 516	CDD:2234 83	25	232	146	9	3	230	1	208	6.03E-23	94.1	COG04 06	phoE	Broad specificity phosphatase PhoE and related phosphatases
LN02_09296 | LN02Chr07 :2063380-2065420(+) | 632	CDD:2235 20	50.081	615	245	10	1	590	1	578	0	670	COG04 43	DnaK	Molecular chaperone
LN02_00081 | LN02Chr01 :419778-422472(+) | 770	CDD:2257 00	27.648	727	406	16	46	765	9	622	8.23E-111	347	COG31 58	Kup	K+ transporter
LN02_00273 | LN02Chr01 :1517162-1519967(-) | 795	CDD:2235 40	23.954	263	155	15	526	766	236	475	6.25E-14	72.2	COG04 64	SpoVK	ATPases of the AAA+ class
LN02_00337 | LN02Chr01 :1820566-1822205(-) | 503	CDD:2241 80	20.941	425	243	12	82	498	5	344	4.74E-59	196	COG12 60	INO1	Myo-inositol-1-phosphate synthase
LN02_00465 | LN02Chr01 :2205106-2206193(+) | 326	CDD:2234 06	29.26	311	191	10	11	319	7	290	3.45E-47	158	COG03 29	DapA	Dihydrodipicolinate synthase/N-acetylneuraminate lyase
LN02_00529 | LN02Chr01 :2387026-2387955(+) | 309	CDD:2233 05	29.333	75	52	1	101	175	1	74	8.69E-17	70.8	COG02 27	RpmB	Ribosomal protein L28
LN02_00593 | LN02Chr01 :2641343-2643021(+) | 490	CDD:2275 14	32.568	479	235	6	11	489	21	411	8.67E-114	339	COG51 87	RPN7	26S proteasome regulatory complex component, contains PCI domain
LN02_00721 | LN02Chr01 :3156775-3158343(+) | 239	CDD:2250 36	47.458	118	59	3	1	118	4	118	1.00E-44	143	COG21 25	RPS6A	Ribosomal protein S6E (S10)
LN02_00849 | LN02Chr01 :3576321-3579707(+) | 1128	CDD:2235 20	25.527	427	250	9	28	454	5	363	2.53E-49	182	COG04 43	DnaK	Molecular chaperone
LN02_00977 | LN02Chr01 :3980550-3988142(+) | 2436	CDD:2273 65	26.904	2245	1430	50	236	2436	28	2105	0	1230	COG50 32	TEL1	Phosphatidylinositol kinase and protein kinases of the PI-3 kinase family
LN02_01041 | LN02Chr01 :4189784-4195544(-) | 1863	CDD:2273 55	22.695	705	406	22	85	766	139	727	9.78E-45	175	COG50 22	COG502 2	Myosin heavy chain
LN02_01041 | LN02Chr01 :4189784-4195544(-) | 1863	CDD:2241 36	18.238	488	286	13	1198	1676	17	400	3.45E-28	116	COG12 15	COG121 5	Glycosyltransferases, probably involved in cell wall biogenesis
LN02_01105 | LN02Chr01 :4441852-4444884(+) | 916	CDD:2236 27	29.3	686	298	16	152	827	335	843	2.81E-86	292	COG05 53	HepA	Superfamily II DNA/RNA helicases, SNF2 family
LN02_01169 | LN02Chr01 :4762153-4764450(+) | 765	CDD:2233 01	18.343	338	160	14	35	369	66	290	1.31E-08	54.2	COG02 23	Fmt	Methionyl-tRNA formyltransferase
LN02_01169 | LN02Chr01 :4762153-4764450(+) | 765	CDD:2239 55	25	232	154	6	444	672	28	242	1.68E-07	50.1	COG10 24	CaiD	Enoyl-CoA hydratase/carnithine racemase
LN02_01297 | LN02Chr01 :5184417-5186464(-) | 652	CDD:2239 89	29.919	371	221	10	39	388	35	387	8.78E-84	267	COG10 61	SSL2	DNA or RNA helicases of superfamily II

LN02_01489∣LN02Chr01:5972177-5974355(-)∣596	CDD:227497	41.322	121	61	5	72	189	1	114	5.92E-31	119	COG5169	HSF1	Heat shock transcription factor
LN02_01553∣LN02Chr01:6239344-6241139(-)∣505	CDD:224384	27.011	348	221	9	94	438	9	326	3.32E-66	214	COG1467	PRI1	Eukaryotic-type DNA primase, catalytic (small) subunit
LN02_01745∣LN02Chr01:6906309-6907903(+)∣463	CDD:223505	26.797	153	94	6	309	458	122	259	6.91E-09	53.5	COG0428	COG0428	Predicted divalent heavy-metal cations transporter
LN02_01809∣LN02Chr01:7101697-7103661(+)∣633	CDD:223492	40.9	489	258	8	138	626	1	458	9.28E-170	489	COG0415	PhrB	Deoxyribodipyrimidine photolyase
LN02_01873∣LN02Chr01:7279287-7287711(-)∣2569	CDD:225858	32.225	1083	571	26	1	1056	1	947	1.26E-170	553	COG3321	COG3321	Polyketide synthase modules and related proteins
LN02_01873∣LN02Chr01:7279287-7287711(-)∣2569	CDD:223677	32.903	310	190	8	1884	2183	24	325	1.11E-53	189	COG0604	Qor	NADPH:quinone reductase and related Zn-dependent oxidoreductases
LN02_01873∣LN02Chr01:7279287-7287711(-)∣2569	CDD:223959	26.111	180	123	7	2208	2378	7	185	4.14E-10	59.8	COG1028	FabG	Dehydrogenases with different specificities (related to short-chain alcohol dehydrogenases)
LN02_01873∣LN02Chr01:7279287-7287711(-)∣2569	CDD:225136	24.413	213	121	14	1452	1640	25	221	1.36E-06	49.2	COG2226	UbiE	Methylase involved in ubiquinone/menaquinone biosynthesis
LN02_02129∣LN02Chr02:692347-694353(+)∣622	CDD:223725	51.592	157	64	5	474	621	3	156	7.95E-69	217	COG0652	PpiB	Peptidyl-prolyl cis-trans isomerase (rotamase) - cyclophilin family
LN02_02129∣LN02Chr02:692347-694353(+)∣622	CDD:225201	25	228	151	6	77	304	248	455	8.63E-13	67.8	COG2319	COG2319	FOG: WD40 repeat
LN02_02257∣LN02Chr02:1138375-1142773(-)∣1397	CDD:224117	20.161	372	271	5	620	987	166	515	2.00E-19	92.1	COG1196	Smc	Chromosome segregation ATPases
LN02_02321∣LN02Chr02:1421134-1422552(-)∣472	CDD:223245	31.7	347	180	11	129	472	2	294	2.08E-72	228	COG0167	PyrD	Dihydroorotate dehydrogenase
LN02_02385∣LN02Chr02:1654977-1657511(-)∣755	CDD:227415	38.88	625	290	11	1	601	1	557	6.38E-172	504	COG5083	SMP2	Uncharacterized protein involved in plasmid maintenance
LN02_02577∣LN02Chr02:2296706-2298597(-)∣345	CDD:226562	26.277	137	91	6	35	170	11	138	1.22E-10	58	COG4076	COG4076	Predicted RNA methylase
LN02_02641∣LN02Chr02:2526341-2528892(+)∣702	CDD:223526	40.142	705	311	15	1	702	1	597	0	736	COG0449	GlmS	Glucosamine 6-phosphate synthetase, contains amidotransferase and phosphosugar isomerase domains
LN02_02705∣LN02Chr02:2732995-2734328(-)∣395	CDD:226406	28.571	70	41	2	214	283	811	871	9.11E-05	41.4	COG3889	COG3889	Predicted solute binding protein
LN02_02769∣LN02Chr02:2915282-2921115(-)∣914	CDD:226406	20.339	118	83	3	322	434	751	862	1.23E-04	42.9	COG3889	COG3889	Predicted solute binding protein
LN02_03089∣LN02Chr02:4052832-4053796(-)∣290	CDD:223955	40.161	249	144	3	40	285	10	256	2.19E-83	248	COG1024	CaiD	Enoyl-CoA hydratase/carnithine racemase
LN02_03153∣LN02Chr02:4324284-4325679(+)∣322	CDD:225413	39.357	249	147	4	57	303	1	247	2.11E-80	242	COG2857	CYT1	Cytochrome c1
LN02_03217∣LN02Chr02:4527579-4528220(+)∣125	CDD:223329	50.413	121	57	2	6	123	6	126	1.31E-47	146	COG0251	TdcF	Putative translation initiation inhibitor, yjgF family
LN02_03281∣LN02Chr02:4727983-4731743(+)∣1171	CDD:224117	18.15	1157	778	31	128	1144	3	1130	2.25E-57	213	COG1196	Smc	Chromosome segregation ATPases

LN02_03409 | LN02Chr02 :5127402-5129032(+) | 470	CDD:225230	33.52	358	186	10	91	445	2	310	4.60E-87	266	COG2355	COG2355	Zn-dependent dipeptidase, microsomal dipeptidase homolog
LN02_03857 | LN02Chr03 :943659-944631(+) | 272	CDD:227455	28.788	132	87	3	97	226	12	138	2.16E-16	72	COG5126	FRQ1	Ca2+-binding protein (EF-Hand superfamily)
LN02_03921 | LN02Chr03 :1143188-1145706(+) | 698	CDD:227694	22.124	678	416	18	1	653	1	591	9.28E-86	279	COG5407	SEC63	Preprotein translocase subunit Sec63
LN02_03985 | LN02Chr03 :1332803-1334341(-) | 418	CDD:223589	35.587	281	150	8	148	401	2	278	1.20E-58	193	COG0515	SPS1	Serine/threonine protein kinase
LN02_04049 | LN02Chr03 :1591681-1592354(+) | 199	CDD:224330	46.715	137	70	3	61	195	1	136	2.36E-45	144	COG1412	COG1412	Uncharacterized proteins of PilT N-term./Vapc superfamily
LN02_04369 | LN02Chr03 :3949140-3950375(-) | 315	CDD:223322	27.919	197	119	6	1	196	1	175	3.40E-34	120	COG0244	RplJ	Ribosomal protein L10
LN02_04561 | LN02Chr03 :4576627-4578548(+) | 566	CDD:223535	35.009	557	313	15	16	561	3	521	3.35E-158	459	COG0459	GroL	Chaperonin GroEL (HSP60 family)
LN02_04625 | LN02Chr03 :4835107-4837543(-) | 589	CDD:224111	43.495	515	263	9	79	589	11	501	0	628	COG1190	LysU	Lysyl-tRNA synthetase (class II)
LN02_04753 | LN02Chr03 :5285514-5289272(+) | 1119	CDD:224117	17.541	1163	796	29	76	1093	3	1147	1.63E-64	235	COG1196	Smc	Chromosome segregation ATPases
LN02_04817 | LN02Chr03 :5512991-5514621(-) | 473	CDD:223371	44.398	241	114	7	224	459	5	230	1.07E-71	225	COG0294	FolP	Dihydropteroate synthase and related enzymes
LN02_04817 | LN02Chr03 :5512991-5514621(-) | 473	CDD:223872	47.328	131	68	1	48	177	1	131	1.04E-51	169	COG0801	FolK	7,8-dihydro-6-hydroxymethylpterin-pyrophosphokinase
LN02_04881 | LN02Chr03 :5859952-5861128(+) | 356	CDD:225852	27.363	201	98	11	60	246	34	200	1.83E-09	55	COG3315	COG3315	O-Methyltransferase involved in polyketide biosynthesis
LN02_05009 | LN02Chr04 :335739-336370(-) | 185	CDD:225777	30.159	63	44	0	73	135	3	65	3.58E-05	37.3	COG3237	COG3237	Uncharacterized protein conserved in bacteria
LN02_05073 | LN02Chr04 :521550-523704(+) | 597	CDD:227615	22.795	601	380	15	1	597	1	521	3.15E-35	136	COG5296	COG5296	Transcription factor involved in TATA site selection and in elongation by RNA polymerase II
LN02_05201 | LN02Chr04 :961838-964734(+) | 896	CDD:226406	24.545	110	72	4	242	341	739	847	2.17E-04	41.8	COG3889	COG3889	Predicted solute binding protein
LN02_05265 | LN02Chr04 :1159326-1161912(+) | 782	CDD:227532	30.556	792	495	22	1	781	1	748	0	597	COG5207	UBP14	Isopeptidase T
LN02_05393 | LN02Chr04 :1594713-1596262(+) | 412	CDD:223505	22.951	183	120	3	225	406	102	264	5.33E-14	68.5	COG0428	COG0428	Predicted divalent heavy-metal cations transporter
LN02_05713 | LN02Chr04 :2961463-2963567(+) | 614	CDD:227680	18.078	437	308	8	179	576	97	522	3.58E-25	106	COG5391	COG5391	Phox homology (PX) domain protein
LN02_05841 | LN02Chr04 :3379269-3380421(+) | 353	CDD:223197	21.701	341	214	9	17	353	1	292	7.17E-55	182	COG0119	LeuA	Isopropylmalate/homocitrate/citramalate synthases
LN02_05905 | LN02Chr04 :3578667-3580925(+) | 573	CDD:223232	25.331	529	299	21	81	573	1	469	2.07E-69	229	COG0154	GatA	Asp-tRNAAsn/Glu-tRNAGln amidotransferase A subunit and related amidases
LN02_06353 | LN02Chr04 :5116682-5117522(-) | 259	CDD:225491	22.034	177	101	6	25	200	332	472	1.53E-06	45.6	COG2940	COG2940	Proteins containing SET domain

LN02_06545∣LN02Chr04:5807443-5808539(+)∣328	CDD:227022	27.143	280	160	10	34	276	94	366	1.65E-24	100	COG4677	PemB	Pectin methylesterase
LN02_06609∣LN02Chr05:107864-108908(+)∣294	CDD:223959	31.317	281	157	8	14	288	1	251	2.36E-47	156	COG1028	FabG	Dehydrogenases with different specificities (related to short-chain alcohol dehydrogenases)
LN02_06737∣LN02Chr05:634367-636013(+)∣548	CDD:223725	33.511	188	88	5	14	194	1	158	1.37E-47	160	COG0652	PpiB	Peptidyl-prolyl cis-trans isomerase (rotamase) - cyclophilin family
LN02_06801∣LN02Chr05:855441-856913(+)∣417	CDD:225768	38.176	296	156	9	123	413	233	506	7.34E-95	291	COG3227	LasB	Zinc metalloprotease (elastase)
LN02_06865∣LN02Chr05:1069578-1070851(+)∣368	CDD:225546	31.333	150	97	5	200	347	88	233	2.08E-27	106	COG3000	ERG3	Sterol desaturase
LN02_07121∣LN02Chr05:2637103-2639046(+)∣597	CDD:223131	28.425	292	177	5	242	533	11	270	3.56E-63	207	COG0053	MMT1	Predicted Co/Zn/Cd cation transporters
LN02_07249∣LN02Chr05:3088693-3089964(+)∣423	CDD:226651	40.625	32	19	0	2	33	75	106	3.36E-04	39.4	COG4188	COG4188	Predicted dienelactone hydrolase
LN02_07441∣LN02Chr05:3796366-3798338(-)∣577	CDD:225181	30.04	506	299	17	27	529	8	461	1.81E-69	229	COG2272	PnbA	Carboxylesterase type B
LN02_07505∣LN02Chr05:4002001-4002865(-)∣242	CDD:224584	15.347	202	119	6	9	204	5	160	4.51E-06	43	COG1670	RimL	Acetyltransferases, including N-acetylases of ribosomal proteins
LN02_07825∣LN02Chr05:5409151-5410179(+)∣342	CDD:224092	37.463	339	202	7	1	337	1	331	1.81E-64	205	COG1171	IlvA	Threonine dehydratase
LN02_07889∣LN02Chr05:5609786-5612443(+)∣647	CDD:223232	23.529	459	251	24	203	615	67	471	3.57E-11	62.7	COG0154	GatA	Asp-tRNAAsn/Glu-tRNAGln amidotransferase A subunit and related amidases
LN02_07953∣LN02Chr05:5782699-5784074(+)∣440	CDD:223589	19.487	390	205	10	39	426	2	284	3.14E-15	74	COG0515	SPS1	Serine/threonine protein kinase
LN02_08145∣LN02Chr06:1407304-1410699(+)∣1131	CDD:224732	22.87	446	288	16	292	729	4	401	1.76E-38	146	COG1819	COG1819	Glycosyl transferases, related to UDP-glucuronosyltransferase
LN02_08145∣LN02Chr06:1407304-1410699(+)∣1131	CDD:227376	32.5	80	49	1	913	992	2325	2399	2.34E-04	42.6	COG5043	MRS6	Vacuolar protein sorting-associated protein
LN02_08209∣LN02Chr06:1781550-1783798(+)∣645	CDD:223589	31.319	364	211	9	259	607	1	340	9.92E-55	188	COG0515	SPS1	Serine/threonine protein kinase
LN02_08465∣LN02Chr06:2701272-2702282(+)∣285	CDD:223955	31.518	257	160	4	22	278	14	254	1.21E-49	162	COG1024	CaiD	Enoyl-CoA hydratase/carnithine racemase
LN02_08913∣LN02Chr07:785411-788600(+)∣1013	CDD:223104	25.113	442	289	10	9	441	1	409	3.68E-92	297	COG0025	NhaP	NhaP-type Na+/H+ and K+/H+ antiporters
LN02_09041∣LN02Chr07:1221578-1223391(+)∣526	CDD:227116	26.794	418	242	16	128	525	393	766	8.07E-44	162	COG4775	COG4775	Outer membrane protein/protective antigen OMA87
LN02_09233∣LN02Chr07:1864682-1870936(-)∣1981	CDD:223715	37.209	258	138	6	1309	1565	102	336	5.24E-57	198	COG0642	BaeS	Signal transduction histidine kinase
LN02_09233∣LN02Chr07:1864682-1870936(-)∣1981	CDD:225112	28.729	181	121	3	971	1151	3	175	1.45E-17	81	COG2202	AtoS	FOG: PAS/PAC domain

LN02_09233|LN02Chr07:1864682-1870936(-)|1981	CDD:223855	30.172	116	63	3	1875	1973	1	115	9.48E-17	75.7	COG0784	CheY	FOG: CheY-like receiver
LN02_09233|LN02Chr07:1864682-1870936(-)|1981	CDD:226229	21.053	228	128	7	1774	1973	39	242	1.56E-05	46.5	COG3706	PleD	Response regulator containing a CheY-like receiver domain and a GGDEF domain
LN02_00530|LN02Chr01:2388199-2390784(-)|720	CDD:223211	67.095	389	126	2	325	712	8	395	0	745	COG0133	TrpB	Tryptophan synthase beta chain
LN02_00530|LN02Chr01:2388199-2390784(-)|720	CDD:223237	44.358	257	142	1	1	257	1	256	3.74E-105	319	COG0159	TrpA	Tryptophan synthase alpha chain
LN02_00594|LN02Chr01:2643850-2645731(-)|534	CDD:223513	29.6	375	216	17	166	534	61	393	1.65E-64	212	COG0436	COG0436	Aspartate/tyrosine/aromatic aminotransferase
LN02_00722|LN02Chr01:3159252-3171727(-)|4004	CDD:227354	37.277	617	328	11	3390	4001	308	870	2.03E-180	577	COG5021	HUL4	Ubiquitin-protein ligase
LN02_00786|LN02Chr01:3413137-3415711(+)|812	CDD:223282	26.531	147	93	6	268	409	117	253	5.21E-10	58	COG0204	PlsC	1-acyl-sn-glycerol-3-phosphate acyltransferase
LN02_00914|LN02Chr01:3754093-3757532(-)|804	CDD:227392	38.947	285	157	6	508	785	58	332	7.98E-70	237	COG5059	KIP1	Kinesin-like protein
LN02_00914|LN02Chr01:3754093-3757532(-)|804	CDD:224117	26.217	267	177	6	192	456	652	900	3.50E-16	80.1	COG1196	Smc	Chromosome segregation ATPases
LN02_00978|LN02Chr01:3988864-3990896(-)|566	CDD:224117	19.068	236	157	5	155	363	292	520	2.07E-06	47.8	COG1196	Smc	Chromosome segregation ATPases
LN02_01042|LN02Chr01:4200051-4205596(+)|1782	CDD:224136	16.189	488	288	13	1041	1517	19	396	1.24E-21	96.9	COG1215	COG1215	Glycosyltransferases, probably involved in cell wall biogenesis
LN02_01042|LN02Chr01:4200051-4205596(+)|1782	CDD:227355	24.294	177	91	9	186	331	255	419	1.94E-05	46.6	COG5022	COG5022	Myosin heavy chain
LN02_01106|LN02Chr01:4445342-4451090(-)|1852	CDD:223738	25.191	131	85	3	1332	1459	68	188	3.87E-07	50.2	COG0666	Arp	FOG: Ankyrin repeat
LN02_01106|LN02Chr01:4445342-4451090(-)|1852	CDD:223738	32.258	124	71	3	1415	1527	70	191	8.59E-05	43.3	COG0666	Arp	FOG: Ankyrin repeat
LN02_01106|LN02Chr01:4445342-4451090(-)|1852	CDD:224117	18.519	189	142	5	1663	1845	280	462	1.02E-05	47.4	COG1196	Smc	Chromosome segregation ATPases
LN02_01362|LN02Chr01:5438126-5440412(+)|655	CDD:224009	43.064	346	191	5	1	343	1	343	8.91E-150	434	COG1084	COG1084	Predicted GTPase
LN02_01490|LN02Chr01:5978682-5981472(-)|896	CDD:225201	22.43	321	225	9	7	312	56	367	8.38E-25	105	COG2319	COG2319	FOG: WD40 repeat
LN02_01554|LN02Chr01:6243757-6246025(-)|570	CDD:227497	41.026	117	65	3	11	125	3	117	7.50E-37	135	COG5169	HSF1	Heat shock transcription factor
LN02_01554|LN02Chr01:6243757-6246025(-)|570	CDD:223855	25.758	132	92	3	346	476	2	128	1.71E-24	96.1	COG0784	CheY	FOG: CheY-like receiver
LN02_01682|LN02Chr01:6740016-6743031(+)|972	CDD:223589	20.308	325	215	7	334	618	14	334	4.27E-14	72.1	COG0515	SPS1	Serine/threonine protein kinase

LN02_02386∣LN02Chr02:1660119-1663748(+)∣1130	CDD:224117	25.238	210	155	2	296	503	727	936	3.73E-13	70.9	COG1196	Smc	Chromosome segregation ATPases
LN02_02578∣LN02Chr02:2299701-2300553(-)∣260	CDD:224918	33.884	121	54	2	140	260	33	127	3.45E-28	101	COG2007	RPS8A	Ribosomal protein S8E
LN02_02642∣LN02Chr02:2529755-2530725(-)∣261	CDD:223711	27.803	223	148	6	34	253	24	236	3.13E-42	141	COG0638	PRE1	20S proteasome, alpha and beta subunits
LN02_02834∣LN02Chr02:3162543-3166332(+)∣1218	CDD:223587	34.93	501	310	7	576	1072	15	503	1.95E-131	409	COG0513	SrmB	Superfamily II DNA and RNA helicases
LN02_02898∣LN02Chr02:3392993-3396672(+)∣1202	CDD:225201	30.337	89	42	3	167	255	161	229	2.64E-05	45.1	COG2319	COG2319	FOG: WD40 repeat
LN02_02898∣LN02Chr02:3392993-3396672(+)∣1202	CDD:225201	19.149	282	192	10	661	932	155	410	4.46E-04	41.2	COG2319	COG2319	FOG: WD40 repeat
LN02_02962∣LN02Chr02:3610678-3613213(-)∣704	CDD:223515	15.909	220	145	5	122	341	51	230	9.88E-05	42.2	COG0438	RfaG	Glycosyltransferase
LN02_02962∣LN02Chr02:3610678-3613213(-)∣704	CDD:223515	13.939	165	117	5	441	605	241	380	5.43E-04	39.9	COG0438	RfaG	Glycosyltransferase
LN02_03090∣LN02Chr02:4054435-4055965(-)∣470	CDD:224172	31.808	437	253	11	44	468	2	405	8.05E-101	305	COG1252	Ndh	NADH dehydrogenase, FAD-containing subunit
LN02_03218∣LN02Chr02:4528472-4529703(-)∣362	CDD:225201	24.107	224	143	6	10	231	240	438	1.12E-12	65.9	COG2319	COG2319	FOG: WD40 repeat
LN02_03282∣LN02Chr02:4732137-4733044(-)∣255	CDD:227877	24.878	205	142	4	51	250	32	229	7.76E-33	116	COG5590	COG5590	Uncharacterized conserved protein
LN02_03346∣LN02Chr02:4943015-4944151(+)∣352	CDD:225201	23.39	295	192	7	8	296	102	368	3.22E-27	109	COG2319	COG2319	FOG: WD40 repeat
LN02_03410∣LN02Chr02:5129325-5129859(-)∣85	CDD:223177	32.5	40	27	0	1	40	1	40	2.99E-04	34	COG0099	RpsM	Ribosomal protein S13
LN02_03474∣LN02Chr02:5716189-5717383(-)∣327	CDD:227410	26.271	118	81	3	12	126	1	115	6.44E-27	100	COG5078	COG5078	Ubiquitin-protein ligase
LN02_03730∣LN02Chr03:461482-462408(+)∣308	CDD:225136	25.641	195	111	8	74	245	46	229	2.23E-09	53.8	COG2226	UbiE	Methylase involved in ubiquinone/menaquinone biosynthesis
LN02_03794∣LN02Chr03:766877-768661(+)∣567	CDD:225442	34.694	49	22	4	43	88	20	61	6.71E-05	38.2	COG2888	COG2888	Predicted Zn-ribbon RNA-binding protein with a function in translation
LN02_04050∣LN02Chr03:1594625-1596007(+)∣460	CDD:223774	22.053	263	171	10	64	326	1	229	3.16E-06	45.7	COG0702	COG0702	Predicted nucleoside-diphosphate-sugar epimerases
LN02_04242∣LN02Chr03:3501442-3506376(+)∣1468	CDD:226406	23.204	181	101	7	428	582	694	862	4.66E-04	41.8	COG3889	COG3889	Predicted solute binding protein
LN02_04306∣LN02Chr03:3726383-3728317(+)∣644	CDD:225121	17.021	235	165	8	143	371	66	276	3.44E-07	49.9	COG2211	MelB	Na+/melibiose symporter and related transporters
LN02_04434∣LN02Chr03:4164267-4165272(+)∣247	CDD:223322	20.219	183	122	6	18	187	3	174	1.06E-11	58.8	COG0244	RplJ	Ribosomal protein L10

LN02_04562 | LN02Chr03 :4579130-4580130(-) | 234	CDD:2237 95	32.164	171	106	3	66	234	4	166	1.10E-49	158	COG07 23	QcrA	Rieske Fe-S protein
LN02_04626 | LN02Chr03 :4838307-4839025(-) | 137	CDD:2263 14	32.353	136	82	4	1	136	2	127	2.29E-26	93.3	COG37 91	COG379 1	Uncharacterized conserved protein
LN02_04690 | LN02Chr03 :5084905-5085679(+) | 232	CDD:2237 65	31.915	235	106	9	1	232	1	184	3.12E-29	105	COG06 93	ThiJ	Putative intracellular protease/amidase
LN02_04754 | LN02Chr03 :5290543-5295109(-) | 1449	CDD:2241 17	17.17	629	409	11	797	1326	224	839	7.62E-22	99.8	COG11 96	Smc	Chromosome segregation ATPases
LN02_04754 | LN02Chr03 :5290543-5295109(-) | 1449	CDD:2255 97	23.563	174	87	11	277	408	79	248	2.01E-06	48.9	COG30 55	COG305 5	Uncharacterized protein conserved in bacteria
LN02_04818 | LN02Chr03 :5515661-5517336(+) | 381	CDD:2249 81	35.507	276	160	6	7	277	3	265	4.65E-52	174	COG20 70	COG207 0	Dioxygenases related to 2-nitropropane dioxygenase
LN02_04882 | LN02Chr03 :5861867-5863029(+) | 319	CDD:2275 73	23.75	80	59	1	8	87	7	84	5.05E-06	42.3	COG52 48	TAF19	Transcription initiation factor TFIID, subunit TAF13
LN02_05202 | LN02Chr04 :965553-970481(-) | 1375	CDD:2264 06	24.074	162	99	7	412	562	694	842	2.81E-04	42.2	COG38 89	COG388 9	Predicted solute binding protein
LN02_05458 | LN02Chr04 :1901770-1903557(+) | 439	CDD:2252 01	27.055	292	173	11	138	428	197	449	1.53E-27	111	COG23 19	COG231 9	FOG: WD40 repeat
LN02_05650 | LN02Chr04 :2748484-2750030(+) | 421	CDD:2241 43	54.167	384	175	1	38	421	24	406	0	588	COG12 22	RPT1	ATP-dependent 26S proteasome regulatory subunit
LN02_05842 | LN02Chr04 :3381127-3383062(+) | 511	CDD:2240 78	64.059	473	158	1	14	486	3	463	0	835	COG11 56	NtpB	Archaeal/vacuolar-type H+-ATPase subunit B
LN02_05970 | LN02Chr04 :3860411-3861210(-) | 214	CDD:2274 55	25.325	154	100	2	58	211	17	155	1.39E-19	78.9	COG51 26	FRQ1	Ca2+-binding protein (EF-Hand superfamily)
LN02_06034 | LN02Chr04 :4058049-4061256(+) | 934	CDD:2240 37	23.482	494	305	17	281	755	296	735	1.90E-37	148	COG11 12	COG111 2	Superfamily I DNA and RNA helicases and helicase subunits
LN02_06226 | LN02Chr04 :4726461-4726882(-) | 79	CDD:2245 58	56.25	64	27	1	1	64	1	63	1.87E-31	101	COG16 44	RPB10	DNA-directed RNA polymerase, subunit N (RpoN/RPB10)
LN02_06290 | LN02Chr04 :4924748-4925875(+) | 375	CDD:2235 05	21.212	165	114	4	210	371	109	260	8.39E-05	40.8	COG04 28	COG042 8	Predicted divalent heavy-metal cations transporter
LN02_06354 | LN02Chr04 :5119444-5121315(-) | 489	CDD:2251 80	17.906	363	260	13	42	386	24	366	1.13E-04	41.5	COG22 71	UhpC	Sugar phosphate permease
LN02_06610 | LN02Chr05 :109039-110251(-) | 155	CDD:2255 46	32.812	64	40	2	66	127	172	234	9.35E-10	52.5	COG30 00	ERG3	Sterol desaturase
LN02_06674 | LN02Chr05 :359236-360966(+) | 576	CDD:2251 81	28.125	512	279	21	48	535	9	455	4.12E-67	223	COG22 72	PnbA	Carboxylesterase type B
LN02_06738 | LN02Chr05 :637365-639689(+) | 741	CDD:2274 97	38.71	124	72	2	147	270	9	128	1.86E-28	112	COG51 69	HSF1	Heat shock transcription factor
LN02_06802 | LN02Chr05 :858053-859405(-) | 415	CDD:2251 82	28.689	122	81	3	119	238	151	268	9.02E-11	59.8	COG22 73	SKN1	Beta-glucanase/Beta-glucan synthetase
LN02_06930 | LN02Chr05 :1455619-1460609(-) | 1125	CDD:2264 06	24	125	90	2	283	403	739	862	1.87E-04	42.5	COG38 89	COG388 9	Predicted solute binding protein
LN02_07122 | LN02Chr05 :2641444-2642142(+) | 232	CDD:2239 96	23.571	140	96	6	90	227	372	502	8.54E-09	52	COG10 70	XylB	Sugar (pentulose and hexulose) kinases

LN02_07250 | LN02Chr05:3091101-3091776(-) | 184	CDD:223738	27.82	133	85	2	45	172	99	225	4.33E-08	48.3	COG0666	Arp	FOG: Ankyrin repeat
LN02_07314 | LN02Chr05:3312347-3315213(+) | 914	CDD:225201	23.4	500	286	15	225	717	31	440	1.01E-30	124	COG2319	COG2319	FOG: WD40 repeat
LN02_07314 | LN02Chr05:3312347-3315213(+) | 914	CDD:225201	29.804	255	160	5	25	267	203	450	2.40E-20	92.5	COG2319	COG2319	FOG: WD40 repeat
LN02_07506 | LN02Chr05:4003280-4005542(-) | 670	CDD:223221	36.833	562	297	9	50	583	2	533	1.32E-170	496	COG0143	MetG	Methionyl-tRNA synthetase
LN02_07826 | LN02Chr05:5412614-5414711(-) | 447	CDD:223197	32.723	382	229	6	47	420	15	376	6.66E-116	343	COG0119	LeuA	Isopropylmalate/homocitrate/citramalate synthases
LN02_07954 | LN02Chr05:5784520-5785899(+) | 459	CDD:224356	40.476	84	48	2	274	356	95	177	6.01E-19	81.3	COG1439	COG1439	Predicted nucleic acid-binding protein, consists of a PIN domain and a Zn-ribbon module
LN02_08082 | LN02Chr06:1189635-1190812(+) | 355	CDD:225687	29.24	171	92	6	158	321	42	190	2.50E-17	76.3	COG3145	AlkB	Alkylated DNA repair protein
LN02_08210 | LN02Chr06:1785158-1786627(+) | 489	CDD:223671	18.519	54	43	1	348	400	268	321	4.35E-04	39.2	COG0598	CorA	Mg2+ and Co2+ transporters
LN02_08274 | LN02Chr06:1952809-1954413(-) | 467	CDD:223589	22.715	383	272	12	12	379	1	374	2.15E-41	149	COG0515	SPS1	Serine/threonine protein kinase
LN02_08338 | LN02Chr06:2158970-2159658(-) | 173	CDD:225489	34.524	84	54	1	36	119	1	83	2.79E-21	80.4	COG2938	COG2938	Uncharacterized conserved protein
LN02_08658 | LN02Chr06:3311158-3312743(-) | 450	CDD:223688	35.156	128	80	3	21	147	3	128	3.77E-37	130	COG0615	TagD	Cytidylyltransferase
LN02_08658 | LN02Chr06:3311158-3312743(-) | 450	CDD:223688	22.727	176	85	7	242	406	4	139	2.52E-14	66.9	COG0615	TagD	Cytidylyltransferase
LN02_08850 | LN02Chr07:583580-586003(-) | 730	CDD:226406	23.353	167	109	7	253	413	694	847	1.42E-04	42.2	COG3889	COG3889	Predicted solute binding protein
LN02_09042 | LN02Chr07:1225906-1230946(+) | 1523	CDD:224054	30.126	239	152	7	894	1128	18	245	3.65E-50	177	COG1131	CcmA	ABC-type multidrug transport system, ATPase component
LN02_09042 | LN02Chr07:1225906-1230946(+) | 1523	CDD:224054	25.911	247	165	5	171	417	7	235	1.31E-41	152	COG1131	CcmA	ABC-type multidrug transport system, ATPase component
LN02_00019 | LN02Chr01:204381-207346(-) | 967	CDD:227371	18.019	838	548	33	3	788	31	781	1.26E-51	194	COG5038	COG5038	Ca2+-dependent lipid-binding protein, contains C2 domain
LN02_00083 | LN02Chr01:426097-427782(-) | 541	CDD:223589	25.298	336	221	8	59	373	3	329	1.52E-45	161	COG0515	SPS1	Serine/threonine protein kinase
LN02_00211 | LN02Chr01:1159573-1161304(+) | 509	CDD:224513	24.129	373	201	11	128	492	3	301	9.22E-35	129	COG1597	LCB5	Sphingosine kinase and enzymes related to eukaryotic diacylglycerol kinase
LN02_00275 | LN02Chr01:1524623-1525335(-) | 216	CDD:225021	47.977	173	81	3	41	207	3	172	1.15E-56	175	COG2110	COG2110	Predicted phosphatase homologous to the C-terminal domain of histone macroH2A1
LN02_00595 | LN02Chr01:2646833-2647219(+) | 58	CDD:223345	34.694	49	30	2	8	54	2	50	4.19E-06	36.5	COG0267	RpmG	Ribosomal protein L33

LN02_00723|LN02Chr01:3175291-3178337(+)|904	CDD:227535	33.184	223	136	3	537	758	282	492	7.19E-44	163	COG5210	COG5210	GTPase-activating protein
LN02_00723|LN02Chr01:3175291-3178337(+)|904	CDD:223496	22.699	163	120	3	746	902	277	439	2.92E-06	48.2	COG0419	SbcC	ATPase involved in DNA repair
LN02_00915|LN02Chr01:3759015-3760120(+)|328	CDD:223282	21.622	185	133	7	42	217	1	182	1.93E-05	42.2	COG0204	PlsC	1-acyl-sn-glycerol-3-phosphate acyltransferase
LN02_01043|LN02Chr01:4206584-4208173(-)|463	CDD:223715	25.234	107	48	1	350	456	253	327	9.23E-06	44.4	COG0642	BaeS	Signal transduction histidine kinase
LN02_01107|LN02Chr01:4452752-4454026(-)|424	CDD:223959	27.111	225	134	7	52	275	2	197	6.38E-22	91.4	COG1028	FabG	Dehydrogenases with different specificities (related to short-chain alcohol dehydrogenases)
LN02_01171|LN02Chr01:4766857-4767813(+)|318	CDD:224001	20.69	174	114	6	1	172	45	196	6.78E-07	47.1	COG1075	LipA	Predicted acetyltransferases and hydrolases with the alpha/beta hydrolase fold
LN02_01363|LN02Chr01:5441338-5443448(-)|464	CDD:227377	43.612	454	225	12	1	450	1	427	1.05E-152	438	COG5044	MRS6	RAB proteins geranylgeranyltransferase component A (RAB escort protein)
LN02_01427|LN02Chr01:5739775-5741654(+)|588	CDD:225181	24.765	533	291	22	20	522	9	461	4.54E-35	135	COG2272	PnbA	Carboxylesterase type B
LN02_01491|LN02Chr01:5981897-5983283(+)|394	CDD:225201	21.587	315	213	9	61	370	134	419	3.08E-21	92.1	COG2319	COG2319	FOG: WD40 repeat
LN02_01555|LN02Chr01:6250685-6253223(+)|783	CDD:224129	24.638	414	231	15	77	477	3	348	4.86E-45	162	COG1208	GCD1	Nucleoside-diphosphate-sugar pyrophosphorylase involved in lipopolysaccharide biosynthesis/translation initiation factor 2B, gamma/epsilon subunits (eIF-2Bgamma/eIF-2Bepsilon)
LN02_01683|LN02Chr01:6746234-6747750(+)|472	CDD:224316	38.832	291	171	6	32	320	3	288	2.68E-104	310	COG1398	OLE1	Fatty-acid desaturase
LN02_01683|LN02Chr01:6746234-6747750(+)|472	CDD:227599	21.29	155	106	5	316	461	11	158	2.11E-16	73.7	COG5274	CYB5	Cytochrome b involved in lipid metabolism
LN02_02003|LN02Chr02:277805-280845(-)|958	CDD:226406	24.561	171	102	6	283	443	694	847	2.95E-05	44.9	COG3889	COG3889	Predicted solute binding protein
LN02_02259|LN02Chr02:1151183-1151621(+)|95	CDD:227935	36.842	95	60	0	1	95	51	145	9.62E-21	79.1	COG5648	NHP6B	Chromatin-associated proteins containing the HMG domain
LN02_02387|LN02Chr02:1664319-1666854(+)|815	CDD:227278	19.892	186	120	7	36	199	29	207	8.37E-06	45.9	COG4942	COG4942	Membrane-bound metallopeptidase
LN02_02643|LN02Chr02:2532190-2533377(+)|326	CDD:227935	23.333	90	54	3	214	300	55	132	4.51E-04	37.9	COG5648	NHP6B	Chromatin-associated proteins containing the HMG domain
LN02_02707|LN02Chr02:2741097-2742097(-)|295	CDD:224330	34.667	150	75	7	19	162	4	136	4.43E-21	84.3	COG1412	COG1412	Uncharacterized proteins of PilT N-term./Vapc superfamily
LN02_02963|LN02Chr02:3616245-3617774(+)|472	CDD:225201	24.275	276	160	9	131	402	213	443	9.38E-20	88.6	COG2319	COG2319	FOG: WD40 repeat
LN02_03155|LN02Chr02:4327941-4329446(-)|440	CDD:224143	51.707	410	191	1	28	437	4	406	0	617	COG1222	RPT1	ATP-dependent 26S proteasome regulatory subunit

LN02_03219∣LN02Chr02:4530181-4530902(+)∣201	CDD:223796	25.926	135	92	3	7	138	96	225	1.45E-13	65	COG0724	COG0724	RNA-binding proteins (RRM domain)
LN02_03283∣LN02Chr02:4735738-4737853(+)∣471	CDD:223796	24.091	220	147	4	58	275	94	295	6.15E-17	78.5	COG0724	COG0724	RNA-binding proteins (RRM domain)
LN02_03283∣LN02Chr02:4735738-4737853(+)∣471	CDD:223796	19.838	247	138	5	206	450	118	306	1.56E-15	74.2	COG0724	COG0724	RNA-binding proteins (RRM domain)
LN02_03347∣LN02Chr02:4944522-4945911(-)∣394	CDD:227353	48.454	291	142	1	68	358	74	356	2.04E-138	397	COG5020	KTR1	Mannosyltransferase
LN02_03411∣LN02Chr02:5130376-5131499(+)∣269	CDD:223725	46.914	162	73	5	44	198	3	158	1.17E-61	188	COG0652	PpiB	Peptidyl-prolyl cis-trans isomerase (rotamase) - cyclophilin family
LN02_03475∣LN02Chr02:5718350-5720298(-)∣471	CDD:223189	38.438	333	188	4	63	392	5	323	9.86E-117	343	COG0111	SerA	Phosphoglycerate dehydrogenase and related dehydrogenases
LN02_03731∣LN02Chr03:464212-465783(+)∣523	CDD:223903	46.573	496	249	7	36	523	33	520	4.25E-173	496	COG0833	LysP	Amino acid transporters
LN02_03859∣LN02Chr03:948343-950730(+)∣701	CDD:223560	62.5	64	24	0	558	621	4	67	4.35E-32	125	COG0484	DnaJ	DnaJ-class molecular chaperone with C-terminal Zn finger domain
LN02_03859∣LN02Chr03:948343-950730(+)∣701	CDD:223533	26.23	183	119	4	356	537	101	268	2.62E-13	68.3	COG0457	NrfG	FOG: TPR repeat
LN02_03859∣LN02Chr03:948343-950730(+)∣701	CDD:223533	32.353	102	68	1	196	296	161	262	1.43E-08	53.7	COG0457	NrfG	FOG: TPR repeat
LN02_03859∣LN02Chr03:948343-950730(+)∣701	CDD:223533	22.857	245	136	6	200	439	93	289	1.73E-07	50.6	COG0457	NrfG	FOG: TPR repeat
LN02_03923∣LN02Chr03:1149715-1153121(+)∣1033	CDD:223552	28.78	205	136	5	25	221	4	206	2.83E-34	129	COG0476	ThiF	Dinucleotide-utilizing enzymes involved in molybdopterin and thiamine biosynthesis family 2
LN02_03923∣LN02Chr03:1149715-1153121(+)∣1033	CDD:223552	31.361	169	103	4	423	589	9	166	2.72E-31	120	COG0476	ThiF	Dinucleotide-utilizing enzymes involved in molybdopterin and thiamine biosynthesis family 2
LN02_04371∣LN02Chr03:3953324-3954776(-)∣415	CDD:223560	36.103	349	202	8	2	343	1	335	1.45E-105	314	COG0484	DnaJ	DnaJ-class molecular chaperone with C-terminal Zn finger domain
LN02_04435∣LN02Chr03:4166127-4167748(-)∣398	CDD:223796	24.476	143	97	2	50	189	61	195	6.60E-14	68.8	COG0724	COG0724	RNA-binding proteins (RRM domain)
LN02_04563∣LN02Chr03:4580539-4584180(-)∣1163	CDD:227490	19.792	384	263	10	811	1162	946	1316	4.39E-08	54.6	COG5161	SFT1	Pre-mRNA cleavage and polyadenylation specificity factor
LN02_04627∣LN02Chr03:4840180-4841937(-)∣378	CDD:223549	43.305	351	184	8	40	378	1	348	4.84E-141	402	COG0473	LeuB	Isocitrate/isopropylmalate dehydrogenase
LN02_04755∣LN02Chr03:5298513-5303081(+)∣1403	CDD:225201	18.9	291	196	8	1096	1354	127	409	5.14E-16	79.4	COG2319	COG2319	FOG: WD40 repeat
LN02_04819∣LN02Chr03:5517968-5519722(-)∣479	CDD:227651	35.583	163	100	2	5	167	2	159	2.89E-42	149	COG5347	COG5347	GTPase-activating protein that regulates ARFs (ADP-ribosylation factors), involved in ARF-mediated vesicular transport
LN02_04883∣LN02Chr03:5863409-5867090(-)∣1185	CDD:226614	15.71	331	230	14	670	992	13	302	1.96E-05	45	COG4129	COG4129	Predicted membrane protein

LN02_04947	LN02Chr03:6273571-6277899(+)	1442	CDD:226406	23.626	182	108	7	264	427	694	862	5.41E-04	41.4	COG3889	COG3889	Predicted solute binding protein
LN02_05011	LN02Chr04:339908-341732(+)	500	CDD:224029	56.812	389	162	4	99	484	1	386	0	613	COG1104	NifS	Cysteine sulfinate desulfinase/cysteine desulfurase and related enzymes
LN02_05075	LN02Chr04:529214-530659(-)	481	CDD:223737	21.09	422	269	14	3	415	1	367	2.55E-23	98.4	COG0665	DadA	Glycine/D-amino acid oxidases (deaminating)
LN02_05331	LN02Chr04:1399192-1402771(+)	1111	CDD:223627	39.652	517	257	16	174	637	331	845	1.18E-123	397	COG0553	HepA	Superfamily II DNA/RNA helicases, SNF2 family
LN02_05395	LN02Chr04:1605586-1607368(+)	506	CDD:223154	21.687	498	339	13	14	499	1	459	1.37E-60	203	COG0076	GadB	Glutamate decarboxylase and related PLP-dependent proteins
LN02_05715	LN02Chr04:2964961-2965835(+)	261	CDD:224766	33.333	117	69	5	80	195	6	114	1.46E-17	75	COG1853	COG1853	Conserved protein/domain typically associated with flavoprotein oxygenases, DIM6/NTAB family
LN02_05779	LN02Chr04:3166172-3172683(-)	2124	CDD:224117	18.971	991	632	18	82	1038	176	1029	3.47E-22	101	COG1196	Smc	Chromosome segregation ATPases
LN02_05779	LN02Chr04:3166172-3172683(-)	2124	CDD:224117	18.288	771	571	12	853	1587	189	936	1.37E-21	99.8	COG1196	Smc	Chromosome segregation ATPases
LN02_05843	LN02Chr04:3384074-3385980(+)	520	CDD:224928	25	372	194	14	141	510	20	308	4.94E-41	147	COG2017	GalM	Galactose mutarotase and related enzymes
LN02_06035	LN02Chr04:4062171-4064581(+)	768	CDD:225201	26.316	190	119	6	132	306	155	338	2.68E-10	60.5	COG2319	COG2319	FOG: WD40 repeat
LN02_06035	LN02Chr04:4062171-4064581(+)	768	CDD:225201	24.841	157	103	5	591	740	207	355	1.88E-09	57.8	COG2319	COG2319	FOG: WD40 repeat
LN02_06099	LN02Chr04:4262557-4264266(-)	489	CDD:223809	32.338	402	254	5	31	420	12	407	4.14E-80	253	COG0738	FucP	Fucose permease
LN02_06227	LN02Chr04:4727210-4730147(+)	957	CDD:225201	24.167	120	88	1	356	472	276	395	1.25E-04	42.4	COG2319	COG2319	FOG: WD40 repeat
LN02_06291	LN02Chr04:4926747-4927734(+)	151	CDD:225147	48.507	134	67	1	4	137	1	132	1.92E-63	188	COG2238	RPS19A	Ribosomal protein S19E (S16A)
LN02_06547	LN02Chr04:5811559-5812724(-)	357	CDD:223560	40.23	87	46	3	49	134	6	87	2.66E-19	85	COG0484	DnaJ	DnaJ-class molecular chaperone with C-terminal Zn finger domain
LN02_06611	LN02Chr05:111593-111994(+)	133	CDD:227772	33.058	121	77	3	9	128	11	128	2.24E-19	75.2	COG5485	COG5485	Predicted ester cyclase
LN02_06675	LN02Chr05:361632-364561(-)	890	CDD:223385	28.986	897	571	22	1	877	7	857	3.04E-158	482	COG0308	PepN	Aminopeptidase N
LN02_06739	LN02Chr05:640199-640813(-)	169	CDD:223725	39.521	167	84	6	2	162	1	156	1.04E-56	172	COG0652	PpiB	Peptidyl-prolyl cis-trans isomerase (rotamase) - cyclophilin family
LN02_06995	LN02Chr05:1903180-1904200(+)	246	CDD:224887	48.889	225	111	2	1	225	2	222	4.01E-101	290	COG1976	TIF6	Translation initiation factor 6 (eIF-6)
LN02_07251	LN02Chr05:3094458-3096850(+)	745	CDD:224571	29.663	563	346	12	177	738	1	514	3.25E-109	339	COG1657	SqhC	Squalene cyclase
LN02_07315	LN02Chr05:3315751-3317507(+)	576	CDD:227847	21.311	122	81	3	436	557	716	822	6.92E-05	42.6	COG5560	UBP12	Ubiquitin C-terminal hydrolase

LN02_07571 ǀ LN02Chr05 :4189492-4191967(-) ǀ 665	CDD:2267 39	35.484	465	267	14	17	480	25	457	1.36E-91	289	COG42 89	COG428 9	Uncharacterized protein conserved in bacteria
LN02_07827 ǀ LN02Chr05 :5422935-5424826(+) ǀ 527	CDD:2250 35	20.773	414	252	13	115	519	65	411	2.70E-23	99	COG21 24	CypX	Cytochrome P450
LN02_07955 ǀ LN02Chr05 :5786277-5786465(-) ǀ 62	CDD:2251 69	42.373	59	31	3	1	57	1	58	4.91E-18	67.1	COG22 60	COG226 0	Predicted Zn-ribbon RNA-binding protein
LN02_08083 ǀ LN02Chr06 :1191168-1195323(-) ǀ 1330	CDD:2273 54	27.387	398	234	11	949	1330	514	872	1.89E-60	221	COG50 21	HUL4	Ubiquitin-protein ligase
LN02_08147 ǀ LN02Chr06 :1416918-1420205(+) ǀ 1095	CDD:2235 50	30.361	998	575	30	105	1083	19	915	0	553	COG04 74	MgtA	Cation transport ATPase
LN02_08211 ǀ LN02Chr06 :1786994-1788607(-) ǀ 537	CDD:2232 50	35	480	250	13	55	526	1	426	1.58E-109	330	COG01 72	SerS	Seryl-tRNA synthetase
LN02_08403 ǀ LN02Chr06 :2468279-2470239(+) ǀ 530	CDD:2237 68	48.184	523	255	9	3	523	1	509	0	620	COG06 96	GpmI	Phosphoglyceromutase
LN02_08659 ǀ LN02Chr06 :3313455-3314814(+) ǀ 367	CDD:2234 83	22.857	210	119	6	108	313	9	179	1.84E-15	71.7	COG04 06	phoE	Broad specificity phosphatase PhoE and related phosphatases
LN02_08787 ǀ LN02Chr07 :352266-353848(+) ǀ 412	CDD:2235 89	19.663	356	199	13	63	405	1	282	9.33E-16	75.5	COG05 15	SPS1	Serine/threonine protein kinase
LN02_08915 ǀ LN02Chr07 :790529-791661(+) ǀ 299	CDD:2250 31	22.794	272	153	12	31	290	6	232	1.79E-16	74.4	COG21 20	COG212 0	Uncharacterized proteins, LmbE homologs
LN02_09043 ǀ LN02Chr07 :1231648-1233096(-) ǀ 404	CDD:2237 27	25.513	341	216	14	1	333	55	365	1.85E-28	112	COG06 54	UbiH	2-polyprenyl-6-methoxyphenol hydroxylase and related FAD-dependent oxidoreductases
LN02_09107 ǀ LN02Chr07 :1442317-1442982(-) ǀ 221	CDD:2251 36	22.727	154	93	6	8	141	7	154	1.34E-09	53.4	COG22 26	UbiE	Methylase involved in ubiquinone/menaquinone biosynthesis
LN02_09171 ǀ LN02Chr07 :1634438-1635570(-) ǀ 300	CDD:2237 29	43.369	279	141	7	12	286	3	268	1.50E-97	286	COG06 56	ARA1	Aldo/keto reductases, related to diketogulonate reductase
LN02_00020 ǀ LN02Chr01 :207845-211596(+) ǀ 1181	CDD:2273 71	19.835	1089	717	40	3	1034	13	1002	1.85E-85	300	COG50 38	COG503 8	Ca2+-dependent lipid-binding protein, contains C2 domain
LN02_00276 ǀ LN02Chr01 :1526267-1528306(+) ǀ 629	CDD:2230 87	27.925	530	308	15	87	605	6	472	5.22E-111	338	COG00 08	GlnS	Glutamyl- and glutaminyl-tRNA synthetases
LN02_00340 ǀ LN02Chr01 :1826835-1828448(-) ǀ 418	CDD:2232 04	53.976	415	164	10	6	418	4	393	0	524	COG01 26	Pgk	3-phosphoglycerate kinase
LN02_00596 ǀ LN02Chr01 :2647811-2650470(-) ǀ 835	CDD:2241 17	18.395	299	226	5	37	335	690	970	2.27E-09	58.2	COG11 96	Smc	Chromosome segregation ATPases
LN02_00660 ǀ LN02Chr01 :2913742-2922402(-) ǀ 2855	CDD:2239 51	20.777	669	468	16	1203	1842	6	641	3.35E-37	148	COG10 20	EntF	Non-ribosomal peptide synthetase modules and related proteins
LN02_00660 ǀ LN02Chr01 :2913742-2922402(-) ǀ 2855	CDD:2239 51	18.557	194	142	5	2334	2524	15	195	1.37E-04	44.2	COG10 20	EntF	Non-ribosomal peptide synthetase modules and related proteins
LN02_00724 ǀ LN02Chr01 :3179402-3180247(-) ǀ 206	CDD:2249 18	29.897	194	68	3	1	193	1	127	1.28E-38	127	COG20 07	RPS8A	Ribosomal protein S8E
LN02_00916 ǀ LN02Chr01 :3760733-3762672(-) ǀ 603	CDD:2232 07	49.288	562	273	7	47	599	13	571	0	761	COG01 29	IlvD	Dihydroxyacid dehydratase/phosphogluconate dehydratase

LN02_01108|LN02Chr01:4454500-4456740(-)|694	CDD:226199	30.566	265	141	9	55	315	23	248	1.59E-48	173	COG3673	COG3673	Uncharacterized conserved protein
LN02_01172|LN02Chr01:4768190-4769428(-)|385	CDD:223159	19.697	198	107	9	105	282	51	216	5.26E-04	38	COG0081	RplA	Ribosomal protein L1
LN02_02132|LN02Chr02:699315-700754(+)|375	CDD:223794	62.254	355	130	2	13	367	1	351	0	574	COG0722	AroG	3-deoxy-D-arabino-heptulosonate 7-phosphate (DAHP) synthase
LN02_02388|LN02Chr02:1669253-1670892(-)|433	CDD:226734	29.744	390	243	14	21	404	9	373	2.31E-80	253	COG4284	COG4284	UDP-glucose pyrophosphorylase
LN02_02516|LN02Chr02:2072063-2073377(+)|362	CDD:223951	19.786	374	209	8	55	346	161	525	3.60E-18	83.1	COG1020	EntF	Non-ribosomal peptide synthetase modules and related proteins
LN02_02580|LN02Chr02:2302373-2305040(-)|860	CDD:223589	23.256	344	234	9	516	846	2	328	1.07E-35	136	COG0515	SPS1	Serine/threonine protein kinase
LN02_02836|LN02Chr02:3168411-3170829(+)|740	CDD:227516	26.316	76	36	3	588	644	346	420	1.45E-04	41.6	COG5189	SFP1	Putative transcriptional repressor regulating G2/M transition
LN02_02836|LN02Chr02:3168411-3170829(+)|740	CDD:227381	18.577	253	183	8	430	668	221	464	7.00E-04	39.7	COG5048	COG5048	FOG: Zn-finger
LN02_02900|LN02Chr02:3401351-3402629(+)|364	CDD:223991	35.522	335	196	9	36	360	24	348	8.96E-81	248	COG1063	Tdh	Threonine dehydrogenase and related Zn-dependent dehydrogenases
LN02_02964|LN02Chr02:3618057-3620016(-)|615	CDD:223426	23.936	188	115	10	246	429	2	165	1.08E-10	60.8	COG0349	Rnd	Ribonuclease D
LN02_03156|LN02Chr02:4329999-4331780(+)|533	CDD:223535	36.452	513	304	12	25	529	12	510	1.75E-159	461	COG0459	GroL	Chaperonin GroEL (HSP60 family)
LN02_03476|LN02Chr02:5721462-5723181(-)|527	CDD:223545	46.407	487	244	8	32	516	5	476	0	611	COG0469	PykF	Pyruvate kinase
LN02_03668|LN02Chr03:227346-232336(+)|1635	CDD:227510	17.529	696	476	21	930	1615	558	1165	1.40E-53	203	COG5183	SSM4	Protein involved in mRNA turnover and stability
LN02_03668|LN02Chr03:227346-232336(+)|1635	CDD:227510	36.585	123	65	3	27	142	1	117	1.03E-31	132	COG5183	SSM4	Protein involved in mRNA turnover and stability
LN02_03732|LN02Chr03:467453-471310(+)|1285	CDD:223395	29.434	530	332	14	9	517	13	521	5.21E-73	250	COG0318	CaiC	Acyl-CoA synthetases (AMP-forming)/AMP-acid ligases II
LN02_03732|LN02Chr03:467453-471310(+)|1285	CDD:226674	43.265	245	136	2	1038	1281	3	245	1.41E-72	239	COG4221	COG4221	Short-chain alcohol dehydrogenase of unknown specificity
LN02_03732|LN02Chr03:467453-471310(+)|1285	CDD:225857	38.384	297	169	6	651	943	2	288	1.18E-65	224	COG3320	COG3320	Putative dehydrogenase domain of multifunctional non-ribosomal peptide synthetases and related enzymes
LN02_03924|LN02Chr03:1153577-1155125(+)|400	CDD:223512	53.271	321	131	8	72	389	13	317	1.94E-163	459	COG0435	ECM4	Predicted glutathione S-transferase
LN02_04308|LN02Chr03:3729723-3731156(-)|477	CDD:227361	23.636	110	77	3	318	425	4	108	2.05E-04	40.9	COG5028	COG5028	Vesicle coat complex COPII, subunit SEC24/subunit SFB2/subunit SFB3
LN02_04372|LN02Chr03:3956114-3958605(-)|806	CDD:224117	20.741	270	183	6	345	612	242	482	1.91E-09	58.2	COG1196	Smc	Chromosome segregation ATPases

LN02_04372|LN02Chr03:3956114-3958605(-)|806	CDD:227569	38.356	73	40	3	101	173	4	71	1.18E-08	55.5	COG5244	NIP100	Dynactin complex subunit involved in mitotic spindle partitioning in anaphase B
LN02_04628|LN02Chr03:4842892-4845226(-)|678	CDD:223589	30.323	310	184	8	26	306	8	314	1.13E-54	189	COG0515	SPS1	Serine/threonine protein kinase
LN02_04628|LN02Chr03:4842892-4845226(-)|678	CDD:227226	34.483	58	36	1	576	631	925	982	1.55E-05	45.2	COG4889	COG4889	Predicted helicase
LN02_04628|LN02Chr03:4842892-4845226(-)|678	CDD:225805	22.843	197	126	10	386	572	31	211	6.01E-05	42.6	COG3266	DamX	Uncharacterized protein conserved in bacteria
LN02_04692|LN02Chr03:5086798-5089705(-)|938	CDD:227822	22.922	746	422	23	115	854	52	650	3.37E-67	234	COG5535	RAD4	DNA repair protein RAD4
LN02_04948|LN02Chr03:6279512-6283840(+)|1442	CDD:226406	23.626	182	108	7	264	427	694	862	5.41E-04	41.4	COG3889	COG3889	Predicted solute binding protein
LN02_05076|LN02Chr04:531946-532899(-)|270	CDD:225012	46.328	177	93	2	74	248	4	180	8.19E-94	271	COG2101	SPT15	TATA-box binding protein (TBP), component of TFIID and TFIIIB
LN02_05140|LN02Chr04:732099-733436(+)|445	CDD:223523	20.769	390	258	12	33	405	1	356	6.02E-22	94.6	COG0446	HcaD	Uncharacterized NAD(FAD)-dependent dehydrogenases
LN02_05332|LN02Chr04:1403740-1405075(-)|337	CDD:225201	20.058	344	238	13	4	337	103	419	3.37E-18	82.1	COG2319	COG2319	FOG: WD40 repeat
LN02_05396|LN02Chr04:1610559-1612412(-)|586	CDD:225922	19.059	425	224	20	61	452	261	598	1.42E-10	60.9	COG3387	SGA1	Glucoamylase and related glycosyl hydrolases
LN02_05460|LN02Chr04:1908095-1910509(-)|641	CDD:227351	20.952	210	133	8	10	218	2	179	1.08E-06	46.8	COG5018	KapD	Inhibitor of the KinA pathway to sporulation, predicted exonuclease
LN02_05460|LN02Chr04:1908095-1910509(-)|641	CDD:223796	21.008	119	92	2	221	338	84	201	3.35E-05	43.4	COG0724	COG0724	RNA-binding proteins (RRM domain)
LN02_05524|LN02Chr04:2258951-2262087(+)|963	CDD:223400	29.762	420	262	9	37	451	2	393	7.62E-85	283	COG0323	MutL	DNA mismatch repair enzyme (predicted ATPase)
LN02_05524|LN02Chr04:2258951-2262087(+)|963	CDD:223400	25	248	158	6	691	929	398	626	5.00E-39	151	COG0323	MutL	DNA mismatch repair enzyme (predicted ATPase)
LN02_05716|LN02Chr04:2977268-2979385(+)|705	CDD:223403	44.772	679	311	10	1	673	3	623	0	726	COG0326	HtpG	Molecular chaperone, HSP90 family
LN02_05844|LN02Chr04:3386723-3388253(+)|463	CDD:224650	37.183	355	211	9	104	455	1	346	1.21E-112	333	COG1736	DPH2	Diphthamide synthase subunit DPH2
LN02_05908|LN02Chr04:3586230-3587003(+)|257	CDD:224989	36.424	151	82	6	92	236	43	185	9.45E-37	126	COG2078	AMMECR1	Uncharacterized conserved protein
LN02_06228|LN02Chr04:4731180-4732426(+)|377	CDD:226022	32.768	354	204	8	1	352	1	322	5.25E-63	201	COG3491	PcbC	Isopenicillin N synthase and related dioxygenases
LN02_06484|LN02Chr04:5562058-5562977(+)|292	CDD:226098	28.723	94	57	4	181	273	162	246	5.31E-06	43.9	COG3568	ElsH	Metal-dependent hydrolase
LN02_06548|LN02Chr04:5813469-5813947(+)|92	CDD:223263	49.383	81	35	3	9	85	2	80	9.03E-30	98.8	COG0185	RpsS	Ribosomal protein S19
LN02_06612|LN02Chr05:113372-114797(+)|403	CDD:225546	25.258	194	125	6	188	373	53	234	5.04E-30	114	COG3000	ERG3	Sterol desaturase

LN02_06804 ǀ LN02Chr05:862472-863883(-) ǀ 407	CDD:224390	30.11	362	225	9	21	360	12	367	5.67E-63	204	COG1473	AbgB	Metal-dependent amidase/aminoacylase/carboxypeptidase
LN02_06868 ǀ LN02Chr05:1080046-1082051(-) ǀ 623	CDD:227482	40.5	400	209	7	70	462	42	419	1.31E-126	377	COG5153	CVT17	Putative lipase essential for disintegration of autophagic bodies inside the vacuole
LN02_06996 ǀ LN02Chr05:1905199-1906166(+) ǀ 278	CDD:223574	22.652	181	112	6	21	198	3	158	5.03E-07	46.8	COG0500	SmtA	SAM-dependent methyltransferases
LN02_07124 ǀ LN02Chr05:2644186-2645219(+) ǀ 283	CDD:225034	35.018	277	149	4	1	257	1	266	8.02E-80	239	COG2123	COG2123	RNase PH-related exoribonuclease
LN02_07252 ǀ LN02Chr05:3097719-3099312(+) ǀ 488	CDD:227422	22.656	256	130	6	238	488	176	368	8.78E-22	94	COG5091	SGT1	Suppressor of G2 allele of skp1 and related proteins
LN02_07316 ǀ LN02Chr05:3318265-3319511(-) ǀ 345	CDD:227386	34.01	197	110	5	132	311	12	205	1.72E-42	144	COG5053	CDC33	Translation initiation factor 4E (eIF-4E)
LN02_07380 ǀ LN02Chr05:3568035-3572783(+) ǀ 1405	CDD:224055	31.638	531	343	11	879	1403	51	567	2.49E-114	368	COG1132	MdlB	ABC-type multidrug transport system, ATPase and permease components
LN02_07380 ǀ LN02Chr05:3568035-3572783(+) ǀ 1405	CDD:224055	21.599	588	418	14	145	712	3	567	1.66E-66	233	COG1132	MdlB	ABC-type multidrug transport system, ATPase and permease components
LN02_07508 ǀ LN02Chr05:4007209-4008925(+) ǀ 514	CDD:227823	29.231	325	175	12	206	500	20	319	3.36E-45	158	COG5536	BET4	Protein prenyltransferase, alpha subunit
LN02_07892 ǀ LN02Chr05:5615029-5615659(+) ǀ 169	CDD:223753	20.472	127	93	4	10	132	11	133	1.71E-05	40	COG0681	LepB	Signal peptidase I
LN02_08340 ǀ LN02Chr06:2161404-2162320(+) ǀ 200	CDD:227465	37.288	59	34	1	1	56	1	59	4.93E-17	72.8	COG5136	COG5136	U1 snRNP-specific protein C
LN02_09108 ǀ LN02Chr07:1444566-1446049(-) ǀ 411	CDD:223745	30.526	285	164	9	9	290	4	257	5.50E-43	151	COG0673	MviM	Predicted dehydrogenases and related proteins
LN02_09172 ǀ LN02Chr07:1636151-1638378(+) ǀ 715	CDD:223474	30.969	578	291	12	138	715	19	488	2.51E-152	448	COG0397	COG0397	Uncharacterized conserved protein
LN02_00003 ǀ LN02Chr01:63162-64040(-) ǀ 292	CDD:225714	20.968	186	112	7	69	244	67	227	4.58E-08	50.5	COG3173	COG3173	Predicted aminoglycoside phosphotransferase
LN02_00835 ǀ LN02Chr01:3543813-3545698(-) ǀ 247	CDD:223796	27.941	136	96	2	14	148	63	197	5.29E-10	55.7	COG0724	COG0724	RNA-binding proteins (RRM domain)
LN02_01219 ǀ LN02Chr01:4910321-4911090(-) ǀ 243	CDD:223232	25.373	134	73	8	13	136	136	252	3.58E-11	59.2	COG0154	GatA	Asp-tRNAAsn/Glu-tRNAGln amidotransferase A subunit and related amidases
LN02_01283 ǀ LN02Chr01:5145558-5146898(-) ǀ 397	CDD:227442	24.39	369	196	11	24	392	15	300	8.15E-49	164	COG5111	RPC34	DNA-directed RNA polymerase III, subunit C34
LN02_01603 ǀ LN02Chr01:6438654-6440342(+) ǀ 480	CDD:227357	36.935	398	219	6	98	463	35	432	1.53E-115	344	COG5024	COG5024	Cyclin
LN02_01795 ǀ LN02Chr01:7051234-7051611(+) ǀ 125	CDD:223600	45.07	71	34	3	24	91	35	103	3.43E-16	66.8	COG0526	TrxA	Thiol-disulfide isomerase and thioredoxins
LN02_01923 ǀ LN02Chr01:7421761-7422783(+) ǀ 340	CDD:223992	37.941	340	203	5	4	338	1	337	1.73E-107	314	COG1064	AdhP	Zn-dependent alcohol dehydrogenases

LN02_02051 | LN02Chr02 :467304-469336(-) | 592	CDD:225043	27.983	461	261	18	99	555	57	450	1.96E-60	205	COG2132	SufI	Putative multicopper oxidases
LN02_02179 | LN02Chr02 :826955-827678(+) | 121	CDD:224300	22.449	98	76	0	16	113	9	106	1.11E-04	36.1	COG1382	GimC	Prefoldin, chaperonin cofactor
LN02_02307 | LN02Chr02 :1309297-1310624(+) | 414	CDD:223697	24.138	406	262	15	46	413	11	408	2.71E-50	172	COG0624	ArgE	Acetylornithine deacetylase/Succinyl-diaminopimelate desuccinylase and related deacylases
LN02_02371 | LN02Chr02 :1584594-1587842(+) | 1082	CDD:224036	40.153	523	295	10	146	666	1	507	0	565	COG1111	MPH1	ERCC4-like helicases
LN02_02435 | LN02Chr02 :1812358-1813896(+) | 358	CDD:224857	29.73	333	178	10	19	346	2	283	2.09E-54	177	COG1946	TesB	Acyl-CoA thioesterase
LN02_02499 | LN02Chr02 :2032058-2033491(-) | 477	CDD:223634	45.116	215	113	2	253	466	2	212	1.09E-70	221	COG0560	SerB	Phosphoserine phosphatase
LN02_02627 | LN02Chr02 :2465792-2468821(-) | 984	CDD:223631	31.69	710	396	17	270	972	71	698	2.00E-167	504	COG0557	VacB	Exoribonuclease R
LN02_02755 | LN02Chr02 :2861465-2862392(-) | 249	CDD:223761	21.028	214	137	10	9	208	23	218	2.54E-05	41.1	COG0689	Rph	RNase PH
LN02_02819 | LN02Chr02 :3123485-3125119(-) | 383	CDD:224868	36.288	361	173	13	14	372	4	309	3.12E-83	253	COG1957	URH1	Inosine-uridine nucleoside N-ribohydrolase
LN02_02947 | LN02Chr02 :3548391-3549864(-) | 443	CDD:227381	26.446	121	80	5	203	316	264	382	2.64E-08	52.8	COG5048	COG5048	FOG: Zn-finger
LN02_03395 | LN02Chr02 :5080036-5080848(-) | 169	CDD:227385	39.416	137	80	2	14	147	15	151	2.40E-37	124	COG5052	YOP1	Protein involved in membrane traffic
LN02_03587 | LN02Chr02 :6346009-6348108(+) | 600	CDD:225043	23.827	533	277	17	55	579	38	449	4.44E-52	182	COG2132	SufI	Putative multicopper oxidases
LN02_03843 | LN02Chr03 :915248-916066(+) | 272	CDD:224931	31.579	190	125	3	83	272	2	186	6.86E-30	108	COG2020	STE14	Putative protein-S-isoprenylcysteine methyltransferase
LN02_03907 | LN02Chr03 :1105377-1107060(-) | 530	CDD:223944	45.851	482	247	7	50	529	2	471	0	532	COG1012	PutA	NAD-dependent aldehyde dehydrogenases
LN02_04035 | LN02Chr03 :1544313-1546433(-) | 547	CDD:223535	31.002	529	340	11	16	532	1	516	3.47E-131	390	COG0459	GroL	Chaperonin GroEL (HSP60 family)
LN02_04483 | LN02Chr03 :4299674-4302490(-) | 938	CDD:226406	23.636	165	105	4	447	611	709	852	2.33E-04	41.8	COG3889	COG3889	Predicted solute binding protein
LN02_04547 | LN02Chr03 :4526757-4528782(+) | 649	CDD:223466	23.82	445	227	13	41	471	1	347	7.62E-38	141	COG0389	DinP	Nucleotidyltransferase/DNA polymerase involved in DNA repair
LN02_04611 | LN02Chr03 :4796001-4798342(-) | 615	CDD:227827	38.776	49	29	1	371	419	326	373	1.84E-06	47.3	COG5540	COG5540	RING-finger-containing ubiquitin ligase
LN02_04739 | LN02Chr03 :5226965-5228921(-) | 609	CDD:223944	36.495	485	281	11	77	556	7	469	1.53E-110	337	COG1012	PutA	NAD-dependent aldehyde dehydrogenases
LN02_04803 | LN02Chr03 :5457589-5458611(-) | 298	CDD:223796	37.778	90	56	0	209	298	107	196	8.51E-15	70.4	COG0724	COG0724	RNA-binding proteins (RRM domain)
LN02_04995 | LN02Chr04 :300717-301877(-) | 341	CDD:223082	35.484	310	179	6	39	341	15	310	2.64E-75	232	COG0003	ArsA	Predicted ATPase involved in chromosome partitioning

LN02_05443 lLN02Chr04 :1776257-1779373(+) l955	CDD:2264 06	23.353	167	109	7	264	424	694	847	1.49E-04	42.5	COG38 89	COG388 9	Predicted solute binding protein
LN02_05571 lLN02Chr04 :2487337-2488743(+) l448	CDD:2236 99	40.806	397	219	7	39	421	1	395	2.15E-148	425	COG06 26	MetC	Cystathionine beta-lyases/cystathionine gamma-synthases
LN02_05763 lLN02Chr04 :3113091-3115088(+) l532	CDD:2250 35	21.041	442	293	13	102	532	15	411	5.30E-36	136	COG21 24	CypX	Cytochrome P450
LN02_05955 lLN02Chr04 :3813281-3816945(-) l1177	CDD:2274 08	34.667	150	93	2	312	457	120	268	3.40E-26	109	COG50 76	COG507 6	Transcription factor involved in chromatin remodeling, contains bromodomain
LN02_06147 lLN02Chr04 :4418768-4421374(+) l833	CDD:2275 81	40.865	416	229	6	428	830	10	421	1.35E-144	430	COG52 56	TEF1	Translation elongation factor EF-1alpha (GTPase)
LN02_06339 lLN02Chr04 :5083152-5083598(-) l148	CDD:2261 58	27.857	140	88	5	8	147	7	133	1.79E-12	57.8	COG36 31	COG363 1	Ketosteroid isomerase-related protein
LN02_06403 lLN02Chr04 :5275038-5275409(-) l123	CDD:2236 00	23.077	104	76	3	1	101	1	103	1.56E-04	36	COG05 26	TrxA	Thiol-disulfide isomerase and thioredoxins
LN02_06531 lLN02Chr04 :5747772-5749316(+) l514	CDD:2249 83	32.87	432	267	5	5	432	8	420	2.64E-104	317	COG20 72	TrkA	Predicted flavoprotein involved in K+ transport
LN02_06595 lLN02Chr05 :50843-51700(-) l285	CDD:2235 89	25.185	135	91	5	110	240	27	155	1.31E-10	58.2	COG05 15	SPS1	Serine/threonine protein kinase
LN02_06659 lLN02Chr05 :283912-285721(-) l507	CDD:2250 35	25.606	289	157	10	217	501	176	410	1.52E-22	96.7	COG21 24	CypX	Cytochrome P450
LN02_06723 lLN02Chr05 :602512-603735(-) l360	CDD:2233 77	28.7	223	144	5	95	305	9	228	8.99E-28	106	COG03 00	DltE	Short-chain dehydrogenases of various substrate specificities
LN02_06787 lLN02Chr05 :817884-818827(-) l271	CDD:2232 98	24.797	246	158	5	23	265	4	225	1.42E-53	170	COG02 20	COG022 0	Predicted S-adenosylmethionine-dependent methyltransferase
LN02_07107 lLN02Chr05 :2588194-2588802(-) l84	CDD:2249 47	28.571	84	41	2	18	82	8	91	2.21E-10	49.2	COG20 36	HHT1	Histones H3 and H4
LN02_07235 lLN02Chr05 :3049470-3050818(+) l413	CDD:2235 73	52.62	439	152	4	4	405	1	420	0	595	COG04 99	SAM1	S-adenosylhomocysteine hydrolase
LN02_07363 lLN02Chr05 :3447156-3449919(-) l850	CDD:2232 25	25.785	446	224	12	413	839	90	447	1.52E-74	248	COG01 47	TrpE	Anthranilate/para-aminobenzoate synthases component I
LN02_07363 lLN02Chr05 :3447156-3449919(-) l850	CDD:2235 86	30.041	243	103	13	39	266	1	191	7.26E-40	142	COG05 12	PabA	Anthranilate/para-aminobenzoate synthases component II
LN02_07683 lLN02Chr05 :4829966-4831949(+) l548	CDD:2244 02	37.33	442	196	6	79	520	4	364	6.89E-139	403	COG14 85	COG148 5	Predicted ATPase
LN02_07747 lLN02Chr05 :5068557-5070341(-) l568	CDD:2253 71	20.925	411	253	12	80	486	20	362	1.11E-14	73	COG28 14	AraJ	Arabinose efflux permease
LN02_07939 lLN02Chr05 :5739719-5744294(-) l1449	CDD:2231 64	29.061	905	522	23	24	924	7	795	0	718	COG00 86	RpoC	DNA-directed RNA polymerase, beta' subunit/160 kD subunit
LN02_07939 lLN02Chr05 :5739719-5744294(-) l1449	CDD:2231 64	25.634	355	225	12	1068	1410	411	738	1.24E-25	111	COG00 86	RpoC	DNA-directed RNA polymerase, beta' subunit/160 kD subunit
LN02_08195 lLN02Chr06 :1739205-1740807(-) l471	CDD:2241 17	18.919	111	89	1	92	202	379	488	0.001	38.9	COG11 96	Smc	Chromosome segregation ATPases
LN02_08259 lLN02Chr06 :1909911-1912304(-) l640	CDD:2234 42	32.87	575	290	16	61	591	1	523	6.11E-143	423	COG03 65	Acs	Acyl-coenzyme A synthetases/AMP-(fatty) acid ligases

LN02_08323 l LN02Chr06 :2105850-2106320(-) l 130	CDD:227589	53.719	121	54	1	1	119	1	121	1.34E-49	151	COG5264	VTC1	Vacuolar transporter chaperone
LN02_08643 l LN02Chr06 :3272553-3273581(+) l 342	CDD:223406	28.939	311	198	7	12	320	7	296	5.54E-48	160	COG0329	DapA	Dihydrodipicolinate synthase/N-acetylneuraminate lyase
LN02_08707 l LN02Chr06 :3644777-3645950(+) l 355	CDD:226413	25	128	80	3	161	288	59	170	8.33E-08	49.3	COG3897	COG3897	Predicted methyltransferase
LN02_08771 l LN02Chr07 :300505-302775(-) l 590	CDD:227416	33.333	84	50	3	11	91	82	162	7.75E-09	54.1	COG5084	YTH1	Cleavage and polyadenylation specificity factor (CPSF) Clipper subunit and related makorin family Zn-finger proteins
LN02_09091 l LN02Chr07 :1391679-1397127(-) l 1761	CDD:227596	20.398	402	239	18	461	833	3915	4264	1.84E-05	46.9	COG5271	MDN1	AAA ATPase containing von Willebrand factor type A (vWA) domain
LN02_09155 l LN02Chr07 :1586964-1588850(+) l 598	CDD:224117	19.583	240	176	6	162	390	675	908	8.83E-05	42.4	COG1196	Smc	Chromosome segregation ATPases
LN02_09283 l LN02Chr07 :2029008-2030162(+) l 338	CDD:227352	45.302	298	161	2	12	307	2	299	2.30E-140	399	COG5019	CDC3	Septin family protein
LN02_00021 l LN02Chr01 :213425-215079(-) l 525	CDD:225035	22.777	461	275	15	57	504	8	400	5.48E-38	141	COG2124	CypX	Cytochrome P450
LN02_00085 l LN02Chr01 :439033-441919(-) l 664	CDD:223589	24.34	341	183	8	332	657	2	282	1.48E-42	155	COG0515	SPS1	Serine/threonine protein kinase
LN02_00277 l LN02Chr01 :1529407-1532980(+) l 1054	CDD:223562	23.873	599	350	14	435	997	2	530	3.10E-120	376	COG0488	Uup	ATPase components of ABC transporters with duplicated ATPase domains
LN02_00469 l LN02Chr01 :2219495-2220791(+) l 358	CDD:225201	25.152	330	224	12	25	346	103	417	1.16E-19	87.1	COG2319	COG2319	FOG: WD40 repeat
LN02_00533 l LN02Chr01 :2399459-2400649(+) l 373	CDD:223485	54.662	311	127	7	65	373	5	303	8.10E-162	452	COG0408	HemF	Coproporphyrinogen III oxidase
LN02_00917 l LN02Chr01 :3763349-3764380(-) l 218	CDD:223811	48.02	202	91	3	8	204	1	193	1.61E-77	229	COG0740	ClpP	Protease subunit of ATP-dependent Clp proteases
LN02_00981 l LN02Chr01 :3998626-4000902(-) l 758	CDD:227469	23.504	234	111	9	7	236	26	195	6.07E-14	70.7	COG5140	UFD1	Ubiquitin fusion-degradation protein
LN02_01045 l LN02Chr01 :4212510-4214626(+) l 537	CDD:225035	21.586	454	278	12	74	526	25	401	2.85E-60	201	COG2124	CypX	Cytochrome P450
LN02_01237 l LN02Chr01 :5006026-5006947(-) l 167	CDD:224947	35.714	84	54	0	74	157	8	91	2.29E-17	70	COG2036	HHT1	Histones H3 and H4
LN02_01365 l LN02Chr01 :5446677-5448270(+) l 335	CDD:225201	26.367	311	206	7	13	318	94	386	2.32E-28	111	COG2319	COG2319	FOG: WD40 repeat
LN02_01557 l LN02Chr01 :6256364-6256747(+) l 127	CDD:224784	34.177	79	52	0	23	101	13	91	8.73E-09	46.9	COG1872	COG1872	Uncharacterized conserved protein
LN02_01685 l LN02Chr01 :6748367-6749886(-) l 465	CDD:225201	29.114	79	50	3	23	101	239	311	3.26E-05	43.2	COG2319	COG2319	FOG: WD40 repeat
LN02_01749 l LN02Chr01 :6914942-6917881(-) l 870	CDD:223551	30.097	412	256	8	45	453	2	384	1.09E-62	214	COG0475	KefB	Kef-type K+ transport systems, membrane components
LN02_01877 l LN02Chr01 :7294048-7297065(-) l 1005	CDD:223951	22.762	659	434	22	232	870	9	612	1.78E-42	162	COG1020	EntF	Non-ribosomal peptide synthetase modules and related proteins

LN02_02133|LN02Chr02:701620-702962(+)|373	CDD:227381	33.333	81	49	1	142	222	36	111	4.35E-06	45.5	COG5048	COG5048	FOG: Zn-finger
LN02_02197|LN02Chr02:893820-898378(-)|1099	CDD:223968	55.938	1103	425	10	44	1092	48	1143	0	1676	COG1038	PycA	Pyruvate carboxylase
LN02_02261|LN02Chr02:1156855-1158952(-)|553	CDD:223111	35.018	554	313	21	1	539	3	524	0	595	COG0033	Pgm	Phosphoglucomutase
LN02_02325|LN02Chr02:1432631-1433302(-)|223	CDD:225687	27.612	134	82	5	40	164	63	190	9.79E-12	59	COG3145	AlkB	Alkylated DNA repair protein
LN02_02389|LN02Chr02:1671968-1672924(+)|318	CDD:226189	20	185	131	2	117	301	2	169	2.71E-21	86.3	COG3663	Mug	G:T/U mismatch-specific DNA glycosylase
LN02_02453|LN02Chr02:1855816-1858065(-)|370	CDD:225218	21.477	149	96	5	143	283	33	168	7.50E-05	40.6	COG2340	COG2340	Uncharacterized protein with SCP/PR1 domains
LN02_02517|LN02Chr02:2073931-2080788(+)|1879	CDD:223951	29.52	542	316	17	814	1307	4	527	1.30E-62	224	COG1020	EntF	Non-ribosomal peptide synthetase modules and related proteins
LN02_02517|LN02Chr02:2073931-2080788(+)|1879	CDD:223951	28.462	260	153	9	347	583	42	291	6.39E-23	103	COG1020	EntF	Non-ribosomal peptide synthetase modules and related proteins
LN02_02581|LN02Chr02:2305815-2307180(+)|332	CDD:227562	36.12	299	185	5	25	319	13	309	1.55E-73	227	COG5237	PER1	Predicted membrane protein
LN02_02645|LN02Chr02:2536037-2537332(+)|371	CDD:223528	26.87	361	204	13	11	362	2	311	1.67E-39	140	COG0451	WcaG	Nucleoside-diphosphate-sugar epimerases
LN02_02773|LN02Chr02:2958924-2960137(-)|321	CDD:223600	30.682	88	60	1	19	105	30	117	7.57E-15	67.6	COG0526	TrxA	Thiol-disulfide isomerase and thioredoxins
LN02_02837|LN02Chr02:3171789-3172831(-)|124	CDD:223333	35.821	67	43	0	5	71	2	68	2.10E-09	47.7	COG0255	RpmC	Ribosomal protein L29
LN02_02965|LN02Chr02:3620454-3621010(+)|80	CDD:224869	34.615	78	47	1	1	74	2	79	2.43E-18	69.3	COG1958	LSM1	Small nuclear ribonucleoprotein (snRNP) homolog
LN02_03093|LN02Chr02:4067918-4069180(-)|311	CDD:224452	26.012	173	96	8	127	269	35	205	6.18E-08	49.4	COG1535	EntB	Isochorismate hydrolase
LN02_03285|LN02Chr02:4740214-4742110(-)|598	CDD:225779	21.236	259	169	7	307	564	80	304	2.33E-18	83.7	COG3239	DesA	Fatty acid desaturase
LN02_03285|LN02Chr02:4740214-4742110(-)|598	CDD:227599	24.286	70	48	2	64	128	68	137	2.47E-04	39	COG5274	CYB5	Cytochrome b involved in lipid metabolism
LN02_03349|LN02Chr02:4949493-4950218(-)|241	CDD:223477	22.222	162	98	6	17	175	5	141	4.43E-11	57.7	COG0400	COG0400	Predicted esterase
LN02_03477|LN02Chr02:5724705-5726765(-)|632	CDD:225201	21.068	337	232	6	257	593	31	333	3.03E-20	91.3	COG2319	COG2319	FOG: WD40 repeat
LN02_03669|LN02Chr03:233656-235495(+)|588	CDD:224117	25.275	182	130	2	22	197	679	860	3.71E-15	75.9	COG1196	Smc	Chromosome segregation ATPases
LN02_03989|LN02Chr03:1342015-1343838(-)|607	CDD:223587	24.651	430	215	16	146	566	30	359	1.02E-48	174	COG0513	SrmB	Superfamily II DNA and RNA helicases
LN02_04245|LN02Chr03:3535388-3537706(+)|527	CDD:225689	16.471	85	67	2	329	413	71	151	2.80E-05	42.6	COG3147	DedD	Uncharacterized protein conserved in bacteria

LN02_04437|LN02Chr03:4168955-4170072(-)|247	CDD:223227	48.571	245	121	4	3	243	2	245	2.52E-105	302	COG0149	TpiA	Triosephosphate isomerase
LN02_04693|LN02Chr03:5090493-5091419(-)|308	CDD:226110	18.75	128	74	5	40	140	12	136	2.50E-05	41	COG3582	COG3582	Predicted nucleic acid binding protein containing the AN1-type Zn-finger
LN02_04757|LN02Chr03:5308362-5309848(-)|411	CDD:227383	37.965	403	223	7	1	398	1	381	4.10E-94	285	COG5050	EPT1	sn-1,2-diacylglycerol ethanolamine- and cholinephosphotranferases
LN02_04949|LN02Chr03:6289088-6291131(+)|427	CDD:227928	38.235	136	66	5	12	129	83	218	3.47E-20	89.5	COG5641	GAT1	GATA Zn-finger-containing transcription factor
LN02_04949|LN02Chr03:6289088-6291131(+)|427	CDD:225288	26.05	119	72	5	238	354	432	536	1.84E-05	43.9	COG2433	COG2433	Uncharacterized conserved protein
LN02_05013|LN02Chr04:342629-344725(-)|358	CDD:223252	25.843	356	227	11	14	344	4	347	2.78E-83	257	COG0174	GlnA	Glutamine synthetase
LN02_05077|LN02Chr04:536839-538870(-)|633	CDD:223717	30.087	462	256	11	84	544	1	396	1.32E-94	293	COG0644	FixC	Dehydrogenases (flavoproteins)
LN02_05077|LN02Chr04:536839-538870(-)|633	CDD:225289	35.714	56	29	3	576	630	47	96	2.15E-05	40.8	COG2440	FixX	Ferredoxin-like protein
LN02_05205|LN02Chr04:981459-982655(+)|398	CDD:226678	28.405	257	162	10	145	395	116	356	6.19E-34	126	COG4225	COG4225	Predicted unsaturated glucuronyl hydrolase involved in regulation of bacterial surface properties, and related proteins
LN02_05269|LN02Chr04:1168983-1171002(+)|613	CDD:223587	40.863	394	213	7	164	554	2	378	5.55E-127	381	COG0513	SrmB	Superfamily II DNA and RNA helicases
LN02_05461|LN02Chr04:1914360-1916264(+)|548	CDD:225371	21.978	182	133	3	68	243	46	224	2.09E-10	59.5	COG2814	AraJ	Arabinose efflux permease
LN02_05589|LN02Chr04:2552367-2557724(-)|1785	CDD:223951	28.238	471	289	13	38	488	199	640	5.50E-73	254	COG1020	EntF	Non-ribosomal peptide synthetase modules and related proteins
LN02_05589|LN02Chr04:2552367-2557724(-)|1785	CDD:223951	19.178	219	165	4	934	1151	15	222	3.37E-12	68.4	COG1020	EntF	Non-ribosomal peptide synthetase modules and related proteins
LN02_05589|LN02Chr04:2552367-2557724(-)|1785	CDD:223951	19.271	192	148	2	1594	1779	14	204	1.81E-04	43	COG1020	EntF	Non-ribosomal peptide synthetase modules and related proteins
LN02_05589|LN02Chr04:2552367-2557724(-)|1785	CDD:225967	29.825	57	40	0	1269	1325	3	59	4.69E-05	40.5	COG3433	COG3433	Aryl carrier domain
LN02_05717|LN02Chr04:2980221-2982359(-)|591	CDD:227389	32.924	489	250	7	116	590	87	511	1.68E-99	309	COG5056	ARE1	Acyl-CoA cholesterol acyltransferase
LN02_05909|LN02Chr04:3588286-3589101(-)|271	CDD:224025	26.368	201	100	6	13	194	6	177	2.29E-25	97.3	COG1100	COG1100	GTPase SAR1 and related small G proteins
LN02_05973|LN02Chr04:3871398-3875107(-)|1183	CDD:227360	44.186	344	168	5	494	822	61	395	1.36E-118	370	COG5027	SAS2	Histone acetyltransferase (MYST family)
LN02_06421|LN02Chr04:5339100-5340902(-)|600	CDD:223443	38.163	566	273	18	14	566	1	502	9.81E-132	392	COG0366	AmyA	Glycosidases
LN02_06549|LN02Chr04:5814318-5814987(-)|201	CDD:227491	34.826	201	101	6	2	201	26	197	1.02E-36	124	COG5162	COG5162	Transcription initiation factor TFIID, subunit TAF10 (also component of histone acetyltransferase SAGA)
LN02_06613|LN02Chr05:115107-115705(-)|181	CDD:223532	20.513	78	61	1	102	178	93	170	1.02E-04	38.1	COG0456	RimI	Acetyltransferases

LN02_06613|LN02Chr05:115107-115705(-)|181	CDD:224168	20.755	159	99	6	3	156	1	137	3.73E-04	36.5	COG1247	COG1247	Sortase and related acyltransferases
LN02_06677|LN02Chr05:371025-373947(-)|944	CDD:227886	22.464	414	176	13	507	917	21	292	9.58E-39	143	COG5599	PTP2	Protein tyrosine phosphatase
LN02_06741|LN02Chr05:643097-644975(-)|431	CDD:223959	35.249	261	154	5	162	420	4	251	5.23E-67	211	COG1028	FabG	Dehydrogenases with different specificities (related to short-chain alcohol dehydrogenases)
LN02_06805|LN02Chr05:867794-869011(+)|300	CDD:223915	36.17	282	140	6	6	286	1	243	1.34E-67	208	COG0846	SIR2	NAD-dependent protein deacetylases, SIR2 family
LN02_06869|LN02Chr05:1083355-1085511(+)|576	CDD:227494	40.152	528	274	7	7	533	5	491	4.94E-169	487	COG5165	POB3	Nucleosome-binding factor SPN, POB3 subunit
LN02_06997|LN02Chr05:1906770-1909309(-)|545	CDD:224331	27.222	180	103	6	196	367	51	210	1.03E-04	41.4	COG1413	COG1413	FOG: HEAT repeat
LN02_07061|LN02Chr05:2435164-2436143(+)|237	CDD:225605	37.975	79	48	1	31	108	110	188	4.42E-07	46.3	COG3063	PilF	Tfp pilus assembly protein PilF
LN02_07125|LN02Chr05:2645762-2646661(-)|245	CDD:224025	24.352	193	117	8	49	228	3	179	5.22E-20	82.3	COG1100	COG1100	GTPase SAR1 and related small G proteins
LN02_07253|LN02Chr05:3099704-3101344(-)|546	CDD:225086	19.697	198	140	4	317	499	93	286	1.61E-15	74.3	COG2175	TauD	Probable taurine catabolism dioxygenase
LN02_07445|LN02Chr05:3806917-3809101(+)|612	CDD:227270	20.07	573	348	25	50	597	58	545	6.69E-20	91.4	COG4934	COG4934	Predicted protease
LN02_07637|LN02Chr05:4693750-4697151(-)|1028	CDD:227944	18.501	854	577	27	2	813	3	779	2.01E-27	117	COG5657	CSE1	CAS/CSE protein involved in chromosome segregation
LN02_07829|LN02Chr05:5432764-5434274(+)|387	CDD:226131	28.829	111	64	2	63	173	33	128	1.24E-10	55.9	COG3603	COG3603	Uncharacterized conserved protein
LN02_07893|LN02Chr05:5616596-5618817(-)|559	CDD:227396	26.171	363	227	14	22	360	51	396	3.99E-17	81.5	COG5064	SRP1	Karyopherin (importin) alpha
LN02_07893|LN02Chr05:5616596-5618817(-)|559	CDD:227396	24.862	181	123	5	278	446	49	228	2.34E-06	47.2	COG5064	SRP1	Karyopherin (importin) alpha
LN02_07957|LN02Chr05:5791281-5792285(-)|307	CDD:223563	38.462	234	114	7	4	234	54	260	1.45E-69	214	COG0489	Mrp	ATPases involved in chromosome partitioning
LN02_08085|LN02Chr06:1199959-1200915(+)|318	CDD:226406	26.19	126	79	3	64	189	760	871	5.35E-06	44.9	COG3889	COG3889	Predicted solute binding protein
LN02_08149|LN02Chr06:1422140-1424404(-)|754	CDD:223715	34.156	243	147	6	299	540	96	326	2.83E-45	162	COG0642	BaeS	Signal transduction histidine kinase
LN02_08149|LN02Chr06:1422140-1424404(-)|754	CDD:223855	35.772	123	77	2	611	732	4	125	2.90E-31	116	COG0784	CheY	FOG: CheY-like receiver
LN02_08149|LN02Chr06:1422140-1424404(-)|754	CDD:225112	30.894	123	82	3	184	304	111	232	1.77E-08	52.9	COG2202	AtoS	FOG: PAS/PAC domain
LN02_08213|LN02Chr06:1791313-1793795(+)|780	CDD:223336	22.172	221	127	16	25	213	4	211	7.89E-15	73.1	COG0258	Exo	5'-3' exonuclease (including N-terminal domain of PolI)
LN02_08661|LN02Chr06:3317509-3318822(-)|437	CDD:223294	33.249	394	216	11	51	432	3	361	4.30E-101	303	COG0216	PrfA	Protein chain release factor A
LN02_08789|LN02Chr07:358403-359804(-)|384	CDD:225779	24.157	356	210	10	18	365	12	315	9.08E-28	109	COG3239	DesA	Fatty acid desaturase

LN02_08853 | LN02Chr07 :603899-605944(+) | 563	CDD:2235 53	17.288	295	242	1	79	371	43	337	6.18E-04	39.3	COG04 77	ProP	Permeases of the major facilitator superfamily
LN02_08981 | LN02Chr07 :1024202-1025110(-) | 302	CDD:2245 19	23.077	234	164	3	1	232	1	220	6.04E-22	89	COG16 03	RPP1	RNase P/RNase MRP subunit p30
LN02_09045 | LN02Chr07 :1247775-1250048(+) | 708	CDD:2241 61	23.529	187	115	8	52	228	80	248	2.30E-09	55.8	COG12 40	ChlD	Mg-chelatase subunit ChlD
LN02_09173 | LN02Chr07 :1638994-1641577(+) | 742	CDD:2239 65	42.939	694	372	11	34	716	2	682	0	663	COG10 34	NuoG	NADH dehydrogenase/NADH:ubiquinone oxidoreductase 75 kD subunit (chain G)
LN02_09237 | LN02Chr07 :1879768-1882590(+) | 851	CDD:2237 38	32.374	139	82	3	106	236	52	186	7.65E-12	63.3	COG06 66	Arp	FOG: Ankyrin repeat
LN02_09301 | LN02Chr07 :2080498-2083252(+) | 891	CDD:2237 40	26.95	141	97	4	503	642	115	250	1.57E-13	69.6	COG06 68	MscS	Small-conductance mechanosensitive channel
LN02_09301 | LN02Chr07 :2080498-2083252(+) | 891	CDD:2274 55	26.263	99	61	3	370	457	27	124	2.65E-04	39.6	COG51 26	FRQ1	Ca2+-binding protein (EF-Hand superfamily)
LN02_00214 | LN02Chr01 :1166268-1167551(-) | 360	CDD:2232 69	33.724	341	161	6	21	359	8	285	2.46E-113	328	COG01 91	Fba	Fructose/tagatose bisphosphate aldolase
LN02_00278 | LN02Chr01 :1533980-1535815(-) | 560	CDD:2239 03	35.446	505	305	5	28	529	38	524	9.11E-154	448	COG08 33	LysP	Amino acid transporters
LN02_00342 | LN02Chr01 :1833378-1835468(+) | 510	CDD:2250 35	24.793	363	205	12	127	480	81	384	4.67E-42	152	COG21 24	CypX	Cytochrome P450
LN02_00406 | LN02Chr01 :2038657-2044043(-) | 1619	CDD:2236 27	32.847	548	323	12	713	1216	319	865	8.58E-95	324	COG05 53	HepA	Superfamily II DNA/RNA helicases, SNF2 family
LN02_00406 | LN02Chr01 :2038657-2044043(-) | 1619	CDD:2274 70	27.778	90	55	5	348	429	179	266	5.34E-04	41.5	COG51 41	COG514 1	PHD zinc finger-containing protein
LN02_00598 | LN02Chr01 :2652607-2654541(-) | 644	CDD:2252 01	31.387	137	91	3	214	348	253	388	4.05E-11	62.4	COG23 19	COG231 9	FOG: WD40 repeat
LN02_00598 | LN02Chr01 :2652607-2654541(-) | 644	CDD:2252 01	20.285	281	185	11	58	329	202	452	7.56E-06	45.8	COG23 19	COG231 9	FOG: WD40 repeat
LN02_00854 | LN02Chr01 :3593285-3594940(+) | 453	CDD:2274 85	36.111	144	89	2	243	386	22	162	1.23E-36	130	COG51 56	DOC1	Anaphase-promoting complex (APC), subunit 10
LN02_01046 | LN02Chr01 :4215308-4216063(+) | 210	CDD:2254 50	24.194	124	77	5	72	183	159	277	1.06E-05	41.9	COG28 97	SseA	Rhodanese-related sulfurtransferase
LN02_01238 | LN02Chr01 :5007430-5007873(+) | 62	CDD:2272 56	46.512	43	22	1	5	47	4	45	1.41E-05	35.4	COG49 19	COG491 9	Ribosomal protein S30
LN02_01494 | LN02Chr01 :5993397-5994570(-) | 203	CDD:2240 25	33.641	217	126	4	5	203	1	217	1.12E-48	155	COG11 00	COG110 0	GTPase SAR1 and related small G proteins
LN02_01558 | LN02Chr01 :6257033-6257836(+) | 267	CDD:2235 32	31.667	60	36	1	167	221	94	153	1.36E-05	41.5	COG04 56	RimI	Acetyltransferases
LN02_01622 | LN02Chr01 :6502186-6504347(+) | 630	CDD:2273 93	32.258	465	216	12	10	473	5	371	8.14E-115	347	COG50 61	ERO1	Oxidoreductin, endoplasmic reticulum membrane-associated protein involved in disulfide bond formation
LN02_01686 | LN02Chr01 :6750285-6751340(+) | 313	CDD:2276 40	24.378	201	123	7	40	240	53	224	6.89E-11	59	COG53 33	CCL1	Cdk activating kinase (CAK)/RNA polymerase II transcription

														initiation/nucleotide excision repair factor TFIIH/TFIIK, cyclin H subunit
LN02_01750∣LN02Chr01:6919925-6921735(+)∣535	CDD:223560	47.475	99	44	1	7	97	6	104	5.86E-37	137	COG0484	DnaJ	DnaJ-class molecular chaperone with C-terminal Zn finger domain
LN02_01814∣LN02Chr01:7111992-7113872(-)∣626	CDD:223589	23.292	322	188	12	320	623	1	281	5.00E-26	107	COG0515	SPS1	Serine/threonine protein kinase
LN02_01878∣LN02Chr01:7297921-7302264(+)∣1263	CDD:224055	33.616	589	362	8	35	621	3	564	3.37E-116	371	COG1132	MdlB	ABC-type multidrug transport system, ATPase and permease components
LN02_01878∣LN02Chr01:7297921-7302264(+)∣1263	CDD:224055	32.209	593	373	9	669	1260	3	567	2.60E-108	350	COG1132	MdlB	ABC-type multidrug transport system, ATPase and permease components
LN02_02134∣LN02Chr02:703842-705665(+)∣607	CDD:226972	29.195	435	198	11	56	489	27	352	2.96E-62	208	COG4624	COG4624	Iron only hydrogenase large subunit, C-terminal domain
LN02_02262∣LN02Chr02:1159543-1160279(-)∣205	CDD:224860	39.344	183	106	4	22	204	5	182	7.89E-70	208	COG1949	Orn	Oligoribonuclease (3'->5' exoribonuclease)
LN02_02390∣LN02Chr02:1673378-1674890(-)∣468	CDD:227602	26.22	492	276	13	16	468	1	444	3.06E-73	235	COG5277	COG5277	Actin and related proteins
LN02_02518∣LN02Chr02:2081569-2082765(+)∣331	CDD:225875	27.719	285	132	10	40	321	27	240	7.53E-34	122	COG3338	Cah	Carbonic anhydrase
LN02_02710∣LN02Chr02:2746764-2748710(+)∣648	CDD:226582	21.17	359	217	17	172	505	45	362	4.61E-10	59	COG4097	COG4097	Predicted ferric reductase
LN02_02838∣LN02Chr02:3173381-3174236(+)∣228	CDD:224241	18.182	121	92	2	9	124	49	167	8.14E-04	37	COG1322	COG1322	Predicted nuclease of restriction endonuclease-like fold, RmuC family
LN02_02966∣LN02Chr02:3621492-3622442(-)∣128	CDD:224675	30	100	69	1	15	114	1	99	6.62E-18	70.4	COG1761	RPB11	DNA-directed RNA polymerase, subunit L
LN02_03030∣LN02Chr02:3857922-3862289(-)∣1455	CDD:226406	27.928	111	66	5	322	425	739	842	4.42E-05	44.9	COG3889	COG3889	Predicted solute binding protein
LN02_03094∣LN02Chr02:4069912-4084752(-)∣4887	CDD:227596	31.056	4917	2963	125	79	4887	2	4599	0	2814	COG5271	MDN1	AAA ATPase containing von Willebrand factor type A (vWA) domain
LN02_03222∣LN02Chr02:4536587-4538741(+)∣604	CDD:223903	51.773	564	236	7	42	603	12	541	0	677	COG0833	LysP	Amino acid transporters
LN02_03286∣LN02Chr02:4743029-4744905(-)∣508	CDD:224169	48.366	459	230	4	41	498	2	454	0	526	COG1249	Lpd	Pyruvate/2-oxoglutarate dehydrogenase complex, dihydrolipoamide dehydrogenase (E3) component, and related enzymes
LN02_03478∣LN02Chr02:5728145-5730097(+)∣650	CDD:224117	18.543	302	198	7	256	538	367	639	9.79E-05	42.4	COG1196	Smc	Chromosome segregation ATPases
LN02_03670∣LN02Chr03:239252-246346(+)∣2292	CDD:223516	34.247	511	265	12	60	566	2	445	5.00E-147	462	COG0439	AccC	Biotin carboxylase
LN02_03670∣LN02Chr03:239252-246346(+)∣2292	CDD:227136	24.476	572	371	16	1602	2159	1	525	7.85E-136	433	COG4799	COG4799	Acetyl-CoA carboxylase, carboxyltransferase component (subunits alpha and beta)
LN02_03670∣LN02Chr03:239252-246346(+)∣2292	CDD:223968	32.836	67	44	1	699	764	1078	1144	4.33E-09	58.9	COG1038	PycA	Pyruvate carboxylase

LN02_03926∣LN02Chr03:1159879-1162524(-)∣748	CDD:227487	25.49	612	395	17	13	608	12	578	2.78E-97	310	COG5158	SEC1	Proteins involved in synaptic transmission and general secretion, Sec1 family
LN02_04502∣LN02Chr03:4365909-4370234(+)∣1318	CDD:226406	20.339	118	83	3	305	417	751	862	1.05E-04	43.7	COG3889	COG3889	Predicted solute binding protein
LN02_05014∣LN02Chr04:348471-349758(-)∣380	CDD:224749	29.02	255	134	7	1	255	11	218	1.88E-27	105	COG1836	COG1836	Predicted membrane protein
LN02_05078∣LN02Chr04:540544-542616(+)∣523	CDD:226068	36.325	468	256	11	54	518	2	430	3.62E-140	408	COG3538	COG3538	Uncharacterized conserved protein
LN02_05206∣LN02Chr04:982824-984144(-)∣394	CDD:225046	29.643	280	140	8	1	279	1	224	1.07E-47	159	COG2135	COG2135	Uncharacterized conserved protein
LN02_05334∣LN02Chr04:1410738-1414003(+)∣943	CDD:225354	50.794	630	234	7	314	943	1	554	0	804	COG2759	MIS1	Formyltetrahydrofolate synthetase
LN02_05334∣LN02Chr04:1410738-1414003(+)∣943	CDD:223268	51.93	285	128	3	5	289	1	276	2.97E-124	375	COG0190	FolD	5,10-methylene-tetrahydrofolate dehydrogenase/Methenyl tetrahydrofolate cyclohydrolase
LN02_05462∣LN02Chr04:1916388-1917186(-)∣175	CDD:226327	29.268	123	73	4	51	173	4	112	1.12E-19	77.2	COG3805	DodA	Aromatic ring-cleaving dioxygenase
LN02_05526∣LN02Chr04:2264451-2266120(+)∣512	CDD:223567	23.168	505	306	26	36	512	6	456	1.77E-45	162	COG0493	GltD	NADPH-dependent glutamate synthase beta chain and related oxidoreductases
LN02_05590∣LN02Chr04:2559617-2561523(+)∣576	CDD:223395	33.333	555	342	10	25	572	1	534	4.60E-140	414	COG0318	CaiC	Acyl-CoA synthetases (AMP-forming)/AMP-acid ligases II
LN02_05654∣LN02Chr04:2759308-2760666(-)∣452	CDD:223348	24.545	330	179	10	39	367	68	328	4.46E-28	110	COG0270	Dcm	Site-specific DNA methylase
LN02_05846∣LN02Chr04:3389685-3390739(+)∣313	CDD:223959	31.561	301	150	7	10	308	4	250	1.72E-36	128	COG1028	FabG	Dehydrogenases with different specificities (related to short-chain alcohol dehydrogenases)
LN02_06358∣LN02Chr04:5136163-5138949(+)∣764	CDD:223589	23.978	367	180	9	391	739	1	286	5.48E-34	131	COG0515	SPS1	Serine/threonine protein kinase
LN02_06422∣LN02Chr04:5343655-5345313(+)∣552	CDD:225371	24.088	137	87	5	51	185	15	136	2.99E-06	46.5	COG2814	AraJ	Arabinose efflux permease
LN02_06550∣LN02Chr04:5815757-5816656(-)∣215	CDD:227523	55.14	214	93	2	3	214	1	213	3.97E-80	235	COG5196	ERD2	ER lumen protein retaining receptor
LN02_06678∣LN02Chr05:378602-379967(+)∣309	CDD:226191	34.137	249	143	6	47	292	32	262	3.15E-74	226	COG3665	COG3665	Uncharacterized conserved protein
LN02_06998∣LN02Chr05:1911007-1913019(+)∣569	CDD:223150	24.877	611	351	20	1	568	1	546	1.47E-103	323	COG0072	PheT	Phenylalanyl-tRNA synthetase beta subunit
LN02_07126∣LN02Chr05:2647883-2650945(+)∣809	CDD:223104	27.543	403	260	8	179	576	1	376	7.61E-67	225	COG0025	NhaP	NhaP-type Na+/H+ and K+/H+ antiporters
LN02_07254∣LN02Chr05:3101983-3103659(-)∣558	CDD:224590	26.036	169	81	8	363	520	30	165	5.71E-16	73.2	COG1676	SEN2	tRNA splicing endonuclease
LN02_07318∣LN02Chr05:3322460-3324112(-)∣423	CDD:223506	30.986	355	214	6	38	388	17	344	4.11E-69	219	COG0429	COG0429	Predicted hydrolase of the alpha/beta-hydrolase fold

LN02_07446丨LN02Chr05:3810235-3812225(-)丨582	CDD:223903	49.805	512	242	6	70	579	41	539	0	606	COG0833	LysP	Amino acid transporters
LN02_07510丨LN02Chr05:4023015-4024156(+)丨289	CDD:227370	33.505	194	116	5	27	215	3	188	2.42E-31	114	COG5037	TOS9	Gluconate transport-inducing protein
LN02_07638丨LN02Chr05:4699150-4701030(+)丨555	CDD:224864	41.903	494	270	6	17	508	14	492	1.15E-174	500	COG1953	FUI1	Cytosine/uracil/thiamine/allantoin permeases
LN02_07830丨LN02Chr05:5434623-5435429(-)丨268	CDD:224653	31.429	105	64	4	150	252	17	115	1.33E-14	67.7	COG1739	COG1739	Uncharacterized conserved protein
LN02_07894丨LN02Chr05:5622263-5624018(+)丨450	CDD:223591	26.05	119	77	5	323	439	8	117	6.44E-10	53.6	COG0517	COG0517	FOG: CBS domain
LN02_07894丨LN02Chr05:5622263-5624018(+)丨450	CDD:223591	21.053	114	75	4	246	358	8	107	5.91E-08	48.2	COG0517	COG0517	FOG: CBS domain
LN02_08150丨LN02Chr06:1427417-1428956(+)丨418	CDD:225421	17.69	277	194	13	139	404	121	374	4.65E-12	64	COG2866	COG2866	Predicted carboxypeptidase
LN02_08214丨LN02Chr06:1794224-1795948(-)丨574	CDD:225165	51.99	402	186	6	158	555	10	408	5.43E-174	497	COG2256	MGS1	ATPase related to the helicase subunit of the Holliday junction resolvase
LN02_08662丨LN02Chr06:3319490-3320215(+)丨241	CDD:223698	25	208	145	7	3	204	1	203	2.90E-27	101	COG0625	Gst	Glutathione S-transferase
LN02_08854丨LN02Chr07:607786-610083(+)丨765	CDD:225616	32.432	37	25	0	27	63	28	64	4.91E-04	36.9	COG3074	COG3074	Uncharacterized protein conserved in bacteria
LN02_09110丨LN02Chr07:1448246-1449486(-)丨337	CDD:224811	38.824	340	166	9	12	335	1	314	7.98E-126	360	COG1899	DYS1	Deoxyhypusine synthase
LN02_09174丨LN02Chr07:1641871-1644606(-)丨477	CDD:226731	31.111	90	48	4	9	98	6	81	5.42E-11	56	COG4281	ACB	Acyl-CoA-binding protein
LN02_09238丨LN02Chr07:1887314-1890496(+)丨1026	CDD:227535	27.692	260	179	7	667	920	153	409	1.25E-38	148	COG5210	COG5210	GTPase-activating protein
LN02_09302丨LN02Chr07:2084159-2085372(-)丨255	CDD:223489	24.096	249	135	6	20	239	13	236	2.03E-31	113	COG0412	COG0412	Dienelactone hydrolase and related enzymes
LN02_00087丨LN02Chr01:444626-445737(+)丨314	CDD:224191	25.397	252	154	8	46	297	2	219	3.19E-43	145	COG1272	COG1272	Predicted membrane protein, hemolysin III homolog
LN02_00151丨LN02Chr01:731152-734370(+)丨1072	CDD:223627	30.864	567	316	19	159	699	323	839	1.71E-82	284	COG0553	HepA	Superfamily II DNA/RNA helicases, SNF2 family
LN02_00215丨LN02Chr01:1168523-1170078(-)丨394	CDD:223091	42.593	378	206	7	19	391	1	372	8.69E-151	428	COG0012	COG0012	Predicted GTPase, probable translation factor
LN02_00407丨LN02Chr01:2044646-2046436(+)丨596	CDD:223640	27.703	296	149	9	269	563	24	255	3.74E-45	158	COG0566	SpoU	rRNA methylases
LN02_00535丨LN02Chr01:2402187-2403353(-)丨344	CDD:224229	18.939	132	94	6	39	159	5	134	6.08E-13	62.5	COG1310	COG1310	Predicted metal-dependent protease of the PAD1/JAB1 superfamily
LN02_00663丨LN02Chr01:2934926-2937814(+)丨897	CDD:224117	26.882	93	68	0	663	755	396	488	4.55E-06	47.4	COG1196	Smc	Chromosome segregation ATPases
LN02_00855丨LN02Chr01:3596242-3597490(+)丨349	CDD:223407	30.566	265	144	7	84	308	24	288	2.03E-38	136	COG0330	HflC	Membrane protease subunits, stomatin/prohibitin homologs
LN02_00919丨LN02Chr01:3766753-3770174(-)丨1035	CDD:225143	24.503	151	100	4	190	334	211	353	8.98E-10	59.1	COG2234	Iap	Predicted aminopeptidases

LN02_01239|LN02Chr01:5008507-5009062(+)|150	CDD:223796	20.354	113	90	0	1	113	81	193	7.23E-15	67.3	COG0724	COG0724	RNA-binding proteins (RRM domain)
LN02_01303|LN02Chr01:5197850-5201604(+)|1191	CDD:227519	36.936	1175	631	22	1	1168	1	1072	0	961	COG5192	BMS1	GTP-binding protein required for 40S ribosome biogenesis
LN02_01367|LN02Chr01:5454726-5457116(-)|632	CDD:223553	21.831	142	106	1	46	187	2	138	3.49E-08	52.8	COG0477	ProP	Permeases of the major facilitator superfamily
LN02_01559|LN02Chr01:6259093-6260204(-)|303	CDD:225780	21.672	323	182	13	20	291	30	332	1.75E-11	61	COG3240	COG3240	Phospholipase/lecithinase/hemolysin
LN02_01623|LN02Chr01:6505384-6507156(+)|541	CDD:223737	20.248	484	276	15	49	522	1	384	7.13E-25	103	COG0665	DadA	Glycine/D-amino acid oxidases (deaminating)
LN02_02263|LN02Chr02:1161482-1163056(+)|524	CDD:223745	21.778	225	144	9	28	249	5	200	5.75E-26	106	COG0673	MviM	Predicted dehydrogenases and related proteins
LN02_02327|LN02Chr02:1438889-1444303(+)|1767	CDD:227709	21.594	866	537	30	872	1713	402	1149	4.13E-52	198	COG5422	ROM1	RhoGEF, Guanine nucleotide exchange factor for Rho/Rac/Cdc42-like GTPases
LN02_02327|LN02Chr02:1438889-1444303(+)|1767	CDD:227400	20.419	191	136	7	124	306	132	314	1.03E-04	43.9	COG5068	ARG80	Regulator of arginine metabolism and related MADS box-containing transcription factors
LN02_02391|LN02Chr02:1676000-1677495(+)|476	CDD:224111	37.419	465	233	14	1	460	91	502	2.91E-126	373	COG1190	LysU	Lysyl-tRNA synthetase (class II)
LN02_02455|LN02Chr02:1864556-1865708(+)|336	CDD:224117	22.807	171	114	3	160	318	322	486	1.50E-04	40.5	COG1196	Smc	Chromosome segregation ATPases
LN02_02519|LN02Chr02:2083129-2084361(-)|410	CDD:223727	24.181	397	240	17	1	393	1	340	7.97E-30	116	COG0654	UbiH	2-polyprenyl-6-methoxyphenol hydroxylase and related FAD-dependent oxidoreductases
LN02_02583|LN02Chr02:2312058-2314044(-)|576	CDD:223944	32.305	486	303	13	75	559	5	465	5.24E-110	334	COG1012	PutA	NAD-dependent aldehyde dehydrogenases
LN02_02711|LN02Chr02:2750853-2751905(-)|350	CDD:223574	20.091	219	169	3	129	345	17	231	3.26E-05	41.8	COG0500	SmtA	SAM-dependent methyltransferases
LN02_02967|LN02Chr02:3624665-3627514(+)|906	CDD:223541	49.495	594	271	13	272	856	23	596	0	657	COG0465	HflB	ATP-dependent Zn proteases
LN02_03031|LN02Chr02:3864417-3868784(+)|1455	CDD:226406	27.928	111	66	5	322	425	739	842	4.42E-05	44.9	COG3889	COG3889	Predicted solute binding protein
LN02_03095|LN02Chr02:4085103-4085722(+)|179	CDD:223300	35.338	133	73	3	48	179	4	124	4.30E-24	88.9	COG0222	RplL	Ribosomal protein L7/L12
LN02_03159|LN02Chr02:4334289-4335641(-)|411	CDD:223621	35.878	393	192	10	17	407	3	337	4.18E-92	278	COG0547	TrpD	Anthranilate phosphoribosyltransferase
LN02_03287|LN02Chr02:4749667-4751349(+)|517	CDD:223560	42.453	106	54	3	400	502	2	103	1.54E-29	116	COG0484	DnaJ	DnaJ-class molecular chaperone with C-terminal Zn finger domain
LN02_03287|LN02Chr02:4749667-4751349(+)|517	CDD:223533	24.859	177	123	3	34	206	97	267	2.47E-04	40.2	COG0457	NrfG	FOG: TPR repeat
LN02_03351|LN02Chr02:4953805-4955284(+)|461	CDD:223420	25.679	405	233	19	30	412	9	367	1.29E-24	101	COG0343	Tgt	Queuine/archaeosine tRNA-ribosyltransferase

LN02_03415 | LN02Chr02 :5135259-5135974(-) | 161	CDD:2241 17	23.602	161	119	1	2	158	733	893	8.02E-07	44.7	COG11 96	Smc	Chromosome segregation ATPases
LN02_03479 | LN02Chr02 :5731678-5734079(+) | 742	CDD:2235 89	21.951	410	253	8	152	543	2	362	1.47E-29	118	COG05 15	SPS1	Serine/threonine protein kinase
LN02_03671 | LN02Chr03 :247467-247781(-) | 104	CDD:2233 49	34.118	85	48	2	28	104	1	85	5.65E-20	74.7	COG02 71	BolA	Stress-induced morphogen (activity unknown)
LN02_03735 | LN02Chr03 :479452-481110(-) | 552	CDD:2246 32	43.816	283	142	7	129	410	2	268	1.85E-101	304	COG17 18	RIO1	Serine/threonine protein kinase involved in cell cycle control
LN02_03799 | LN02Chr03 :777112-779351(-) | 723	CDD:2252 01	27.913	369	199	10	364	717	131	447	3.89E-40	151	COG23 19	COG231 9	FOG: WD40 repeat
LN02_03927 | LN02Chr03 :1163166-1164209(-) | 347	CDD:2239 80	44.643	336	161	6	1	328	1	319	2.63E-127	365	COG10 52	LdhA	Lactate dehydrogenase and related dehydrogenases
LN02_04631 | LN02Chr03 :4861130-4862767(+) | 418	CDD:2237 96	17.891	313	224	5	27	334	18	302	1.16E-27	108	COG07 24	COG072 4	RNA-binding proteins (RRM domain)
LN02_05015 | LN02Chr04 :350677-351753(-) | 358	CDD:2260 00	20.904	177	116	7	96	260	90	254	2.24E-04	39.5	COG34 69	COG346 9	Chitinase
LN02_05079 | LN02Chr04 :542885-546435(-) | 1142	CDD:2273 54	34.5	400	210	9	749	1142	519	872	1.85E-83	288	COG50 21	HUL4	Ubiquitin-protein ligase
LN02_05271 | LN02Chr04 :1175519-1177225(+) | 526	CDD:2250 35	22.198	455	287	14	45	490	15	411	1.19E-28	114	COG21 24	CypX	Cytochrome P450
LN02_05399 | LN02Chr04 :1626578-1628122(+) | 445	CDD:2248 14	35.93	398	207	13	11	404	4	357	3.54E-97	293	COG19 02	NemA	NADH:flavin oxidoreductases, Old Yellow Enzyme family
LN02_05463 | LN02Chr04 :1918156-1918824(-) | 222	CDD:2236 98	28.922	204	140	4	2	201	1	203	3.17E-32	113	COG06 25	Gst	Glutathione S-transferase
LN02_05527 | LN02Chr04 :2266175-2267409(-) | 393	CDD:2249 36	49.226	323	150	6	74	393	2	313	9.97E-125	359	COG20 25	FixB	Electron transfer flavoprotein, alpha subunit
LN02_05847 | LN02Chr04 :3390854-3391858(-) | 334	CDD:2237 30	26.522	230	123	15	71	292	82	273	2.74E-09	54.6	COG06 57	Aes	Esterase/lipase
LN02_05911 | LN02Chr04 :3592062-3594048(+) | 520	CDD:2231 54	23.57	437	295	13	90	518	32	437	1.72E-50	176	COG00 76	GadB	Glutamate decarboxylase and related PLP-dependent proteins
LN02_05975 | LN02Chr04 :3876532-3877710(-) | 392	CDD:2231 15	29.252	294	193	5	28	321	1	279	9.88E-61	195	COG00 37	MesJ	tRNA(Ile)-lysidine synthase MesJ
LN02_06231 | LN02Chr04 :4735078-4736035(-) | 204	CDD:2276 66	36.139	202	118	3	1	202	1	191	1.44E-43	141	COG53 74	COG537 4	Uncharacterized conserved protein
LN02_06295 | LN02Chr04 :4939257-4940320(-) | 334	CDD:2242 29	31.933	119	67	4	53	170	4	109	8.08E-17	73.3	COG13 10	COG131 0	Predicted metal-dependent protease of the PAD1/JAB1 superfamily
LN02_06423 | LN02Chr04 :5345694-5348269(-) | 590	CDD:2250 35	22.864	398	230	13	189	567	52	391	1.80E-26	109	COG21 24	CypX	Cytochrome P450
LN02_06615 | LN02Chr05 :117786-119121(+) | 337	CDD:2232 54	31.013	158	92	4	114	268	69	212	5.51E-21	87.3	COG01 76	MipB	Transaldolase
LN02_06679 | LN02Chr05 :380377-381668(-) | 389	CDD:2241 43	57.474	388	160	1	5	387	19	406	0	574	COG12 22	RPT1	ATP-dependent 26S proteasome regulatory subunit
LN02_06807 | LN02Chr05 :870559-873158(+) | 679	CDD:2260 67	25.196	766	419	26	23	678	38	759	9.82E-88	288	COG35 37	COG353 7	Putative alpha-1,2-mannosidase

LN02_07255∣LN02Chr05:3105174-3106853(+)∣504	CDD:224136	15.506	445	279	14	51	469	54	427	1.86E-11	63	COG1215	COG1215	Glycosyltransferases, probably involved in cell wall biogenesis
LN02_07319∣LN02Chr05:3327820-3330272(+)∣746	CDD:223589	27.019	322	196	10	384	690	6	303	2.31E-46	167	COG0515	SPS1	Serine/threonine protein kinase
LN02_07959∣LN02Chr05:5797060-5799187(-)∣619	CDD:223597	33.119	311	141	7	28	337	1	245	1.36E-71	231	COG0523	COG0523	Putative GTPases (G3E family)
LN02_08151∣LN02Chr06:1429781-1431782(-)∣616	CDD:227765	24.837	153	103	2	284	436	1	141	1.83E-18	79.4	COG5478	COG5478	Predicted small integral membrane protein
LN02_08151∣LN02Chr06:1429781-1431782(-)∣616	CDD:227765	23.81	147	99	4	398	540	4	141	5.85E-08	49.3	COG5478	COG5478	Predicted small integral membrane protein
LN02_08151∣LN02Chr06:1429781-1431782(-)∣616	CDD:227765	31.667	60	41	0	161	220	1	60	9.46E-05	40.1	COG5478	COG5478	Predicted small integral membrane protein
LN02_08279∣LN02Chr06:1968853-1971124(+)∣701	CDD:223188	48	125	63	2	572	695	53	176	4.99E-41	145	COG0110	WbbJ	Acetyltransferase (isoleucine patch superfamily)
LN02_08407∣LN02Chr06:2482399-2485166(+)∣824	CDD:227496	31.164	799	502	14	5	788	1	766	0	659	COG5167	VID27	Protein involved in vacuole import and degradation
LN02_08471∣LN02Chr06:2719757-2721660(+)∣509	CDD:223354	26.404	178	124	4	42	213	35	211	1.61E-22	97.3	COG0277	GlcD	FAD/FMN-containing dehydrogenases
LN02_08535∣LN02Chr06:2893480-2895234(-)∣543	CDD:224117	21.407	327	229	4	121	443	664	966	1.11E-13	70.9	COG1196	Smc	Chromosome segregation ATPases
LN02_08919∣LN02Chr07:800711-802825(-)∣704	CDD:223234	37.44	414	231	6	227	640	2	387	2.60E-133	395	COG0156	BioF	7-keto-8-aminopelargonate synthetase and related enzymes
LN02_09047∣LN02Chr07:1253908-1257406(-)∣1147	CDD:227223	27.586	232	159	7	570	800	141	364	6.09E-18	84.6	COG4886	COG4886	Leucine-rich repeat (LRR) protein
LN02_09047∣LN02Chr07:1253908-1257406(-)∣1147	CDD:227223	32.479	117	72	4	986	1100	109	220	1.37E-06	48.8	COG4886	COG4886	Leucine-rich repeat (LRR) protein
LN02_09175∣LN02Chr07:1646042-1647087(-)∣321	CDD:223644	24.8	250	136	14	69	302	21	234	1.63E-07	48.4	COG0571	Rnc	dsRNA-specific ribonuclease
LN02_09303∣LN02Chr07:2086026-2088920(+)∣858	CDD:224081	38.889	54	23	3	46	95	6	53	5.62E-04	39.9	COG1159	Era	GTPase
LN02_00088∣LN02Chr01:445785-448042(-)∣727	CDD:225201	19.167	240	175	5	369	604	64	288	7.41E-07	49.3	COG2319	COG2319	FOG: WD40 repeat
LN02_00344∣LN02Chr01:1842749-1845525(+)∣758	CDD:227392	23.585	424	193	11	45	466	42	336	2.60E-29	120	COG5059	KIP1	Kinesin-like protein
LN02_00344∣LN02Chr01:1842749-1845525(+)∣758	CDD:224117	22.368	152	111	3	540	685	293	443	1.15E-05	45.9	COG1196	Smc	Chromosome segregation ATPases
LN02_00536∣LN02Chr01:2404702-2405749(+)∣269	CDD:223637	38.208	212	95	3	40	251	2	177	1.13E-63	194	COG0563	Adk	Adenylate kinase and related kinases
LN02_00664∣LN02Chr01:2939584-2949253(+)∣3200	CDD:227376	23.994	2734	1742	73	1	2621	1	2511	0	958	COG5043	MRS6	Vacuolar protein sorting-associated protein
LN02_00664∣LN02Chr01:2939584-2949253(+)∣3200	CDD:227376	35.742	775	453	15	2435	3194	1802	2546	9.11E-166	571	COG5043	MRS6	Vacuolar protein sorting-associated protein

LN02_00792∣LN02Chr01:3429899-3431472(-)∣480	CDD:224528	32.011	378	200	5	88	465	2	322	2.07E-70	224	COG1612	CtaA	Uncharacterized protein required for cytochrome oxidase assembly
LN02_00856∣LN02Chr01:3598592-3600597(-)∣604	CDD:224415	44.974	378	186	7	43	415	1	361	9.42E-123	365	COG1498	SIK1	Protein implicated in ribosomal biogenesis, Nop56p homolog
LN02_00920∣LN02Chr01:3771771-3774089(-)∣574	CDD:227503	48.551	276	133	5	16	289	1	269	2.12E-103	310	COG5176	MSL5	Splicing factor (branch point binding protein)
LN02_00920∣LN02Chr01:3771771-3774089(-)∣574	CDD:227414	29.524	105	51	4	252	338	20	119	2.20E-12	63.3	COG5082	AIR1	Arginine methyltransferase-interacting protein, contains RING Zn-finger
LN02_00984∣LN02Chr01:4006457-4007950(-)∣448	CDD:227602	28.778	483	242	12	15	440	5	442	6.14E-70	225	COG5277	COG5277	Actin and related proteins
LN02_01048∣LN02Chr01:4218661-4221896(-)∣578	CDD:319244	70.27	74	21	1	452	524	1	74	8.00E-26	97.9	COG5272	UBI4	UBI4; linked to 3D-structure.
LN02_01048∣LN02Chr01:4218661-4221896(-)∣578	CDD:224469	55.102	49	19	2	530	578	1	46	6.34E-18	74.8	COG1552	RPL40A	Ribosomal protein L40E
LN02_01240∣LN02Chr01:5010179-5014123(+)∣1314	CDD:225201	16.709	395	279	16	38	420	72	428	2.28E-16	80.5	COG2319	COG2319	FOG: WD40 repeat
LN02_01432∣LN02Chr01:5753012-5755126(-)∣677	CDD:224557	39.971	683	348	15	22	666	49	707	0	656	COG1643	HrpA	HrpA-like helicases
LN02_01496∣LN02Chr01:5997129-5997827(-)∣175	CDD:224869	29.333	75	44	1	43	117	14	79	2.18E-12	56.9	COG1958	LSM1	Small nuclear ribonucleoprotein (snRNP) homolog
LN02_01752∣LN02Chr01:6927085-6928218(+)∣377	CDD:227599	29.412	102	67	2	242	343	11	107	1.59E-11	59.5	COG5274	CYB5	Cytochrome b involved in lipid metabolism
LN02_01880∣LN02Chr01:7306791-7308682(+)∣438	CDD:227383	35.354	396	231	9	37	424	4	382	1.91E-74	235	COG5050	EPT1	sn-1,2-diacylglycerol ethanolamine- and cholinephosphotranferases
LN02_02008∣LN02Chr02:294738-302573(-)∣2477	CDD:225201	21.852	270	178	9	2211	2475	161	402	3.84E-09	58.6	COG2319	COG2319	FOG: WD40 repeat
LN02_02072∣LN02Chr02:517327-519779(+)∣746	CDD:224250	34.438	694	344	18	36	728	8	591	1.01E-163	485	COG1331	COG1331	Highly conserved protein containing a thioredoxin domain
LN02_02712∣LN02Chr02:2757997-2759777(+)∣545	CDD:225035	22.947	475	248	14	75	544	50	411	4.10E-27	110	COG2124	CypX	Cytochrome P450
LN02_02840∣LN02Chr02:3176792-3178487(+)∣493	CDD:223582	30.531	452	264	6	40	490	1	403	3.93E-97	296	COG0508	AceF	Pyruvate/2-oxoglutarate dehydrogenase complex, dihydrolipoamide acyltransferase (E2) component, and related enzymes
LN02_02904∣LN02Chr02:3418330-3420738(-)∣412	CDD:227478	22.353	425	184	11	1	412	1	292	2.88E-21	90.5	COG5149	TOA1	Transcription initiation factor IIA, large chain
LN02_03160∣LN02Chr02:4337781-4338824(+)∣347	CDD:224323	28.763	299	185	5	23	320	4	275	3.24E-71	220	COG1405	SUA7	Transcription initiation factor TFIIIB, Brf1 subunit/Transcription initiation factor TFIIB
LN02_03224∣LN02Chr02:4543154-4547863(+)∣1522	CDD:226855	24.675	77	53	3	60	134	105	178	5.35E-06	47.2	COG4447	COG4447	Uncharacterized protein related to plant photosystem II stability/assembly factor
LN02_03352∣LN02Chr02:4956784-4959103(+)∣655	CDD:224699	27.236	492	277	16	168	649	59	479	1.83E-76	250	COG1785	PhoA	Alkaline phosphatase

LN02_03480 | LN02Chr02 :5734433-5735829(-) | 415	CDD:227375	40.882	340	184	5	11	350	17	339	6.47E-110	324	COG5042	NUP	Purine nucleoside permease
LN02_03672 | LN02Chr03 :248677-251322(+) | 607	CDD:223903	37.817	513	304	5	55	565	42	541	5.78E-175	504	COG0833	LysP	Amino acid transporters
LN02_04312 | LN02Chr03 :3744763-3746121(+) | 452	CDD:223607	28.117	409	213	14	29	431	1	334	1.85E-81	252	COG0533	QRI7	Metal-dependent proteases with possible chaperone activity
LN02_04376 | LN02Chr03 :3967375-3968807(+) | 405	CDD:223560	31.313	99	37	4	46	143	6	74	7.27E-08	50.7	COG0484	DnaJ	DnaJ-class molecular chaperone with C-terminal Zn finger domain
LN02_04504 | LN02Chr03 :4379940-4382213(-) | 677	CDD:226406	25.225	111	78	4	242	349	739	847	2.28E-04	41.4	COG3889	COG3889	Predicted solute binding protein
LN02_04632 | LN02Chr03 :4864328-4865266(-) | 312	CDD:223292	68.121	298	91	1	15	312	3	296	0	504	COG0214	SNZ1	Pyridoxine biosynthesis enzyme
LN02_04760 | LN02Chr03 :5321619-5324456(+) | 815	CDD:224216	20.619	194	124	8	106	295	3	170	3.54E-04	40.8	COG1297	COG1297	Predicted membrane protein
LN02_04824 | LN02Chr03 :5537458-5538717(-) | 419	CDD:227381	52.703	74	35	0	54	127	24	97	2.74E-11	62	COG5048	COG5048	FOG: Zn-finger
LN02_04888 | LN02Chr03 :5879790-5881006(+) | 334	CDD:224995	39.474	304	164	8	26	329	2	285	1.71E-68	213	COG2084	MmsB	3-hydroxyisobutyrate dehydrogenase and related beta-hydroxyacid dehydrogenases
LN02_04952 | LN02Chr03 :6298400-6299461(+) | 353	CDD:225546	25.907	193	127	6	139	330	56	233	3.46E-15	71.7	COG3000	ERG3	Sterol desaturase
LN02_05016 | LN02Chr04 :354156-354987(+) | 254	CDD:226755	31.776	107	65	5	106	211	86	185	3.23E-04	38	COG4305	COG4305	Endoglucanase C-terminal domain/subunit and related proteins
LN02_05080 | LN02Chr04 :548467-550806(+) | 749	CDD:223796	19.828	348	225	5	129	464	1	306	3.54E-27	109	COG0724	COG0724	RNA-binding proteins (RRM domain)
LN02_05080 | LN02Chr04 :548467-550806(+) | 749	CDD:223796	28.571	189	112	2	43	208	101	289	1.83E-15	75	COG0724	COG0724	RNA-binding proteins (RRM domain)
LN02_05144 | LN02Chr04 :740434-741742(+) | 351	CDD:225041	46.667	345	169	7	1	341	5	338	1.20E-134	384	COG2130	COG2130	Putative NADP-dependent oxidoreductases
LN02_05208 | LN02Chr04 :986993-987877(+) | 246	CDD:223483	22.917	192	113	7	1	190	1	159	1.16E-08	50.9	COG0406	phoE	Broad specificity phosphatase PhoE and related phosphatases
LN02_05528 | LN02Chr04 :2270928-2273855(-) | 607	CDD:226406	19.231	78	63	0	23	100	753	830	3.53E-04	40.6	COG3889	COG3889	Predicted solute binding protein
LN02_05592 | LN02Chr04 :2565166-2566778(-) | 423	CDD:224172	19.432	458	253	22	15	416	5	402	1.91E-10	59.2	COG1252	Ndh	NADH dehydrogenase, FAD-containing subunit
LN02_05976 | LN02Chr04 :3878623-3879437(+) | 238	CDD:224277	31.897	116	78	1	84	198	1	116	1.12E-21	83.9	COG1358	RPL8A	Ribosomal protein HS6-type (S12/L30/L7a)
LN02_06232 | LN02Chr04 :4736711-4740298(-) | 1160	CDD:225491	37.5	136	73	3	723	856	340	465	1.30E-17	84.1	COG2940	COG2940	Proteins containing SET domain
LN02_06552 | LN02Chr04 :5819257-5820626(-) | 370	CDD:223465	20.69	348	169	11	3	336	2	256	1.32E-14	70.2	COG0388	COG0388	Predicted amidohydrolase
LN02_06616 | LN02Chr05 :119270-120314(-) | 326	CDD:223959	23.556	225	138	6	27	246	2	197	5.07E-20	84.9	COG1028	FabG	Dehydrogenases with different specificities (related to short-chain alcohol dehydrogenases)

LN02_06680|LN02Chr05:383498-387143(+)|1134	CDD:223464	20.608	592	243	7	505	1095	1	366	5.26E-85	277	COG0387	ChaA	Ca2+/H+ antiporter
LN02_06744|LN02Chr05:649961-652489(-)|792	CDD:223541	48.81	504	237	6	259	744	96	596	0	564	COG0465	HflB	ATP-dependent Zn proteases
LN02_06808|LN02Chr05:874937-876005(-)|309	CDD:227463	25.217	230	122	8	6	190	1	225	2.82E-09	53.9	COG5134	COG5134	Uncharacterized conserved protein
LN02_06872|LN02Chr05:1095072-1097127(-)|553	CDD:223769	18.367	147	111	4	183	328	154	292	6.47E-04	38.7	COG0697	RhaT	Permeases of the drug/metabolite transporter (DMT) superfamily
LN02_07000|LN02Chr05:1917795-1918818(+)|257	CDD:223170	29.204	226	139	5	12	230	10	221	2.86E-65	200	COG0092	RpsC	Ribosomal protein S3
LN02_07448|LN02Chr05:3815070-3816866(+)|553	CDD:226651	40	35	21	0	129	163	72	106	1.78E-04	40.9	COG4188	COG4188	Predicted dienelactone hydrolase
LN02_07640|LN02Chr05:4706395-4708374(-)|576	CDD:225371	27.778	144	87	6	147	286	61	191	6.68E-05	42.2	COG2814	AraJ	Arabinose efflux permease
LN02_07704|LN02Chr05:4948491-4961744(+)|4344	CDD:227570	17.652	3280	2378	90	1073	4314	159	3153	0	1237	COG5245	DYN1	Dynein, heavy chain
LN02_07768|LN02Chr05:5183944-5184801(+)|237	CDD:227410	31.677	161	92	5	1	158	5	150	2.71E-36	122	COG5078	COG5078	Ubiquitin-protein ligase
LN02_07896|LN02Chr05:5629836-5631494(+)|414	CDD:225421	19.608	255	172	11	156	399	141	373	2.65E-12	64.8	COG2866	COG2866	Predicted carboxypeptidase
LN02_07960|LN02Chr05:5801484-5803152(-)|462	CDD:223884	17.15	379	263	8	43	388	14	374	1.90E-07	50	COG0814	SdaC	Amino acid permeases
LN02_08920|LN02Chr07:806066-808371(+)|689	CDD:223442	32.598	589	329	22	88	669	1	528	7.80E-120	365	COG0365	Acs	Acyl-coenzyme A synthetases/AMP-(fatty) acid ligases
LN02_08984|LN02Chr07:1027418-1030607(-)|952	CDD:224732	22.098	448	274	15	150	587	10	392	2.32E-42	157	COG1819	COG1819	Glycosyl transferases, related to UDP-glucuronosyltransferase
LN02_09304|LN02Chr07:2089436-2090437(-)|333	CDD:223242	24.473	237	125	9	49	282	4	189	1.12E-39	135	COG0164	RnhB	Ribonuclease HII
LN02_00089|LN02Chr01:449505-451389(+)|505	CDD:225371	18.325	191	131	7	261	447	215	384	6.58E-05	42.2	COG2814	AraJ	Arabinose efflux permease
LN02_00089|LN02Chr01:449505-451389(+)|505	CDD:223553	15.502	329	269	4	41	369	4	323	1.06E-04	41.2	COG0477	ProP	Permeases of the major facilitator superfamily
LN02_00153|LN02Chr01:735420-736077(-)|183	CDD:223596	23.729	177	120	5	1	177	1	162	1.56E-29	105	COG0522	RpsD	Ribosomal protein S4 and related proteins
LN02_00217|LN02Chr01:1172002-1172484(-)|160	CDD:223178	30.508	118	73	3	43	160	21	129	4.71E-21	80.4	COG0100	RpsK	Ribosomal protein S11
LN02_00281|LN02Chr01:1548931-1549611(-)|226	CDD:224504	38.889	54	32	1	129	182	14	66	1.98E-04	36.5	COG1588	POP4	RNase P/RNase MRP subunit p29
LN02_00537|LN02Chr01:2406431-2409481(-)|782	CDD:224117	23.125	320	223	5	166	480	661	962	1.10E-11	65.5	COG1196	Smc	Chromosome segregation ATPases
LN02_00537|LN02Chr01:2406431-2409481(-)|782	CDD:224117	18.546	399	255	9	285	677	181	515	3.36E-09	57.4	COG1196	Smc	Chromosome segregation ATPases
LN02_00601|LN02Chr01:2663649-2664806(-)|330	CDD:224981	38.209	335	189	8	5	324	5	336	6.27E-65	205	COG2070	COG2070	Dioxygenases related to 2-nitropropane dioxygenase

LN02_00665∣LN02Chr01:2951895-2954136(+)∣713	CDD:224883	34.878	410	258	5	292	700	2	403	7.43E-110	336	COG1972	NupC	Nucleoside permease
LN02_00729∣LN02Chr01:3188569-3191598(-)∣963	CDD:227223	30.469	128	88	1	109	236	122	248	3.25E-16	78.9	COG4886	COG4886	Leucine-rich repeat (LRR) protein
LN02_00793∣LN02Chr01:3431799-3434705(+)∣911	CDD:227931	24.334	863	531	22	123	909	52	868	6.37E-62	223	COG5644	COG5644	Uncharacterized conserved protein
LN02_00857∣LN02Chr01:3601195-3602480(+)∣363	CDD:224444	25.068	367	245	11	1	362	4	345	1.08E-69	219	COG1527	NtpC	Archaeal/vacuolar-type H+-ATPase subunit C
LN02_00985∣LN02Chr01:4008682-4009791(-)∣369	CDD:223739	28.986	276	155	12	5	260	37	291	9.29E-32	119	COG0667	Tas	Predicted oxidoreductases (related to aryl-alcohol dehydrogenases)
LN02_01049∣LN02Chr01:4222771-4223816(-)∣159	CDD:223309	28.906	128	83	4	19	146	1	120	7.69E-33	110	COG0231	Efp	Translation elongation factor P (EF-P)/translation initiation factor 5A (eIF-5A)
LN02_01113∣LN02Chr01:4466435-4467768(-)∣395	CDD:223324	24.558	452	263	17	5	389	15	455	2.36E-71	228	COG0246	MtlD	Mannitol-1-phosphate/altronate dehydrogenases
LN02_01305∣LN02Chr01:5206082-5209419(-)∣1061	CDD:227535	35.193	233	139	6	309	530	209	440	2.75E-45	168	COG5210	COG5210	GTPase-activating protein
LN02_01305∣LN02Chr01:5206082-5209419(-)∣1061	CDD:224117	16.143	223	153	3	675	895	684	874	5.46E-04	40.9	COG1196	Smc	Chromosome segregation ATPases
LN02_01369∣LN02Chr01:5462914-5464253(+)∣420	CDD:223359	36.232	414	227	10	9	412	3	389	5.99E-122	356	COG0282	ackA	Acetate kinase
LN02_01433∣LN02Chr01:5755745-5762710(+)∣1328	CDD:227369	28.571	140	84	5	409	541	40	170	9.00E-10	59.8	COG5036	COG5036	SPX domain-containing protein involved in vacuolar polyphosphate accumulation
LN02_01433∣LN02Chr01:5755745-5762710(+)∣1328	CDD:223738	29.771	131	83	3	712	834	70	199	1.47E-08	54.1	COG0666	Arp	FOG: Ankyrin repeat
LN02_01433∣LN02Chr01:5755745-5762710(+)∣1328	CDD:223738	25.664	113	67	3	638	736	92	201	1.51E-06	47.9	COG0666	Arp	FOG: Ankyrin repeat
LN02_01497∣LN02Chr01:5999173-5999807(-)∣162	CDD:223568	26.852	108	74	4	33	135	13	120	3.24E-12	58.3	COG0494	MutT	NTP pyrophosphohydrolases including oxidative damage repair enzymes
LN02_01625∣LN02Chr01:6516741-6517456(+)∣179	CDD:223979	25.191	131	90	4	1	131	6	128	8.01E-23	85.9	COG1051	COG1051	ADP-ribose pyrophosphatase
LN02_01817∣LN02Chr01:7120002-7122469(-)∣682	CDD:223732	22.627	632	391	12	45	664	2	547	1.61E-91	292	COG0659	SUL1	Sulfate permease and related transporters (MFS superfamily)
LN02_02009∣LN02Chr02:305771-307445(+)∣531	CDD:224322	37.282	287	136	11	165	421	119	391	4.77E-37	140	COG1404	AprE	Subtilisin-like serine proteases
LN02_02201∣LN02Chr02:912905-916395(+)∣1020	CDD:224760	47.222	36	18	1	777	811	170	205	1.49E-05	44.2	COG1847	Jag	Predicted RNA-binding protein
LN02_02457∣LN02Chr02:1867901-1871301(+)∣1077	CDD:223935	55.115	479	182	8	591	1047	29	496	0	673	COG1003	GcvP	Glycine cleavage system protein P (pyridoxal-binding), C-terminal domain

LN02_02457|LN02Chr02:1867901-1871301(+)|1077	CDD:223935	28.571	182	107	10	289	463	191	356	8.31E-07	49.6	COG1003	GcvP	Glycine cleavage system protein P (pyridoxal-binding), C-terminal domain
LN02_02457|LN02Chr02:1867901-1871301(+)|1077	CDD:223480	48.592	426	198	9	96	519	7	413	7.34E-176	519	COG0403	GcvP	Glycine cleavage system protein P (pyridoxal-binding), N-terminal domain
LN02_02521|LN02Chr02:2089697-2091337(-)|546	CDD:223903	46.449	521	259	9	29	545	37	541	0	543	COG0833	LysP	Amino acid transporters
LN02_02649|LN02Chr02:2550230-2552034(-)|390	CDD:227410	28.03	132	80	5	264	390	31	152	6.56E-14	65.7	COG5078	COG5078	Ubiquitin-protein ligase
LN02_02841|LN02Chr02:3180562-3181336(-)|212	CDD:225804	43.03	165	87	3	43	207	2	159	4.99E-66	197	COG3265	GntK	Gluconate kinase
LN02_02905|LN02Chr02:3422134-3423900(-)|588	CDD:225201	26.46	291	202	8	277	560	157	442	8.34E-38	142	COG2319	COG2319	FOG: WD40 repeat
LN02_03033|LN02Chr02:3880197-3882800(+)|716	CDD:223824	51.025	488	227	6	56	541	16	493	0	651	COG0753	KatE	Catalase
LN02_03097|LN02Chr02:4087594-4088642(+)|330	CDD:223422	34.304	309	155	8	10	316	3	265	7.51E-71	218	COG0345	ProC	Pyrroline-5-carboxylate reductase
LN02_03161|LN02Chr02:4339745-4344522(+)|1493	CDD:223550	18.415	1249	579	32	116	1343	35	864	1.06E-108	364	COG0474	MgtA	Cation transport ATPase
LN02_03353|LN02Chr02:4959640-4960607(-)|285	CDD:225684	31.78	236	130	7	10	245	4	208	9.64E-45	148	COG3142	CutC	Uncharacterized protein involved in copper resistance
LN02_03417|LN02Chr02:5138973-5140206(+)|238	CDD:224843	22.099	362	131	7	17	231	6	363	2.20E-24	96.6	COG1932	SerC	Phosphoserine aminotransferase
LN02_03801|LN02Chr03:787578-789574(-)|510	CDD:223354	15.848	448	310	13	87	503	39	450	1.43E-14	72.6	COG0277	GlcD	FAD/FMN-containing dehydrogenases
LN02_03865|LN02Chr03:961898-964265(+)|747	CDD:227602	16.229	647	321	23	108	744	9	444	3.67E-19	87.9	COG5277	COG5277	Actin and related proteins
LN02_03929|LN02Chr03:1165799-1168177(-)|744	CDD:224983	24.46	278	158	12	6	276	3	235	8.98E-28	114	COG2072	TrkA	Predicted flavoprotein involved in K+ transport
LN02_04249|LN02Chr03:3546568-3549198(+)|815	CDD:223540	40.604	298	140	7	515	808	230	494	3.34E-87	282	COG0464	SpoVK	ATPases of the AAA+ class
LN02_04313|LN02Chr03:3746623-3747585(-)|283	CDD:223711	30.085	236	162	2	39	272	1	235	1.34E-60	189	COG0638	PRE1	20S proteasome, alpha and beta subunits
LN02_04441|LN02Chr03:4176150-4177030(+)|181	CDD:224025	23.308	133	95	2	10	136	1	132	9.74E-18	74.6	COG1100	COG1100	GTPase SAR1 and related small G proteins
LN02_04505|LN02Chr03:4390680-4391999(+)|439	CDD:224805	19.844	257	170	10	160	411	78	303	2.40E-16	76.6	COG1893	ApbA	Ketopantoate reductase
LN02_04633|LN02Chr03:4866651-4867401(+)|227	CDD:223388	47.273	220	84	6	4	223	1	188	2.33E-84	246	COG0311	PDX2	Predicted glutamine amidotransferase involved in pyridoxine biosynthesis
LN02_04825|LN02Chr03:5545908-5547759(+)|460	CDD:223544	22.549	204	103	6	28	226	41	194	1.44E-12	65	COG0468	RecA	RecA/RadA recombinase
LN02_04889|LN02Chr03:5881218-5883720(-)|775	CDD:227427	25.865	607	358	21	44	615	28	577	5.18E-56	202	COG5096	COG5096	Vesicle coat complex, various subunits

LN02_05145 ǀ LN02Chr04 :741992-744460(-) ǀ 822	CDD:227590	41.717	501	280	5	315	808	2	497	1.26E-146	437	COG5265	ATM1	ABC-type transport system involved in Fe-S cluster assembly, permease and ATPase components
LN02_05209 ǀ LN02Chr04 :987981-990214(-) ǀ 652	CDD:223557	52.073	603	281	5	50	651	4	599	0	889	COG0481	LepA	Membrane GTPase LepA
LN02_05273 ǀ LN02Chr04 :1178661-1180538(-) ǀ 577	CDD:223523	28.882	322	214	9	169	485	1	312	3.17E-49	173	COG0446	HcaD	Uncharacterized NAD(FAD)-dependent dehydrogenases
LN02_05273 ǀ LN02Chr04 :1178661-1180538(-) ǀ 577	CDD:225057	37.895	95	57	1	50	144	14	106	2.62E-25	97.4	COG2146	{NirD	Ferredoxin subunits of nitrite reductase and ring-hydroxylating dioxygenases
LN02_05401 ǀ LN02Chr04 :1631535-1632911(-) ǀ 458	CDD:223579	45.503	378	187	5	57	425	1	368	0	524	COG0505	CarA	Carbamoylphosphate synthase small subunit
LN02_05593 ǀ LN02Chr04 :2568549-2572736(+) ǀ 1351	CDD:224055	30.658	623	355	9	58	677	16	564	1.27E-115	371	COG1132	MdlB	ABC-type multidrug transport system, ATPase and permease components
LN02_05593 ǀ LN02Chr04 :2568549-2572736(+) ǀ 1351	CDD:224055	28.522	582	378	8	761	1341	14	558	2.27E-113	365	COG1132	MdlB	ABC-type multidrug transport system, ATPase and permease components
LN02_05657 ǀ LN02Chr04 :2768320-2769438(+) ǀ 372	CDD:227719	35.417	48	29	2	34	81	23	68	2.34E-07	49.3	COG5432	RAD18	RING-finger-containing E3 ubiquitin ligase
LN02_05785 ǀ LN02Chr04 :3191008-3191815(+) ǀ 244	CDD:223393	37.405	131	59	3	112	242	3	110	3.13E-39	129	COG0316	sufA	Fe-S cluster assembly scaffold protein
LN02_05849 ǀ LN02Chr04 :3394958-3395637(-) ǀ 198	CDD:227521	31.148	61	29	2	124	173	21	79	1.65E-07	44.8	COG5194	APC11	Component of SCF ubiquitin ligase and anaphase-promoting complex
LN02_05977 ǀ LN02Chr04 :3880423-3881683(+) ǀ 320	CDD:226275	25.091	275	174	5	14	280	18	268	7.48E-33	119	COG3752	COG3752	Steroid 5-alpha reductase family enzyme
LN02_06361 ǀ LN02Chr04 :5144580-5146871(-) ǀ 684	CDD:224152	22.763	514	308	17	161	660	5	443	4.50E-37	141	COG1231	COG1231	Monoamine oxidase
LN02_06361 ǀ LN02Chr04 :5144580-5146871(-) ǀ 684	CDD:223523	30.233	86	48	3	119	203	101	175	2.16E-04	41.1	COG0446	HcaD	Uncharacterized NAD(FAD)-dependent dehydrogenases
LN02_06425 ǀ LN02Chr04 :5355172-5358555(-) ǀ 969	CDD:225127	34.026	770	414	18	177	930	1	692	7.89E-173	517	COG2217	ZntA	Cation transport ATPase
LN02_06617 ǀ LN02Chr05 :120920-122044(+) ǀ 316	CDD:223528	19.504	282	191	13	16	266	12	288	1.27E-10	58.4	COG0451	WcaG	Nucleoside-diphosphate-sugar epimerases
LN02_06745 ǀ LN02Chr05 :663250-664684(-) ǀ 395	CDD:227381	25.203	123	87	1	14	131	320	442	3.10E-09	55.5	COG5048	COG5048	FOG: Zn-finger
LN02_06809 ǀ LN02Chr05 :877095-878330(-) ǀ 188	CDD:227410	40	155	82	6	1	146	1	153	3.43E-51	159	COG5078	COG5078	Ubiquitin-protein ligase
LN02_06937 ǀ LN02Chr05 :1606691-1607837(+) ǀ 356	CDD:223549	45.833	360	170	8	5	356	3	345	2.45E-139	396	COG0473	LeuB	Isocitrate/isopropylmalate dehydrogenase
LN02_07001 ǀ LN02Chr05 :1920129-1921721(-) ǀ 393	CDD:223769	28.767	73	52	0	43	115	217	289	3.37E-05	42.2	COG0697	RhaT	Permeases of the drug/metabolite transporter (DMT) superfamily
LN02_07001 ǀ LN02Chr05 :1920129-1921721(-) ǀ 393	CDD:223769	17.814	247	172	6	43	286	72	290	4.99E-05	41.8	COG0697	RhaT	Permeases of the drug/metabolite transporter (DMT) superfamily
LN02_07065 ǀ LN02Chr05 :2445234-2446572(-) ǀ 359	CDD:227635	28.521	284	172	6	69	347	25	282	1.09E-49	165	COG5325	COG5325	t-SNARE complex subunit, syntaxin

LN02_07193|LN02Chr05:2874815-2875622(+)|243	CDD:227883	35.329	167	103	3	65	230	28	190	3.61E-39	131	COG5596	TIM22	Mitochondrial import inner membrane translocase, subunit TIM22
LN02_07257|LN02Chr05:3109898-3121861(+)|3687	CDD:227365	22.462	2502	1451	87	1233	3687	46	2105	0	614	COG5032	TEL1	Phosphatidylinositol kinase and protein kinases of the PI-3 kinase family
LN02_07321|LN02Chr05:3333923-3334492(-)|80	CDD:223478	46.429	56	29	1	1	55	1	56	1.79E-11	51.1	COG0401	COG0401	Uncharacterized homolog of Blt101
LN02_07449|LN02Chr05:3818004-3818679(-)|184	CDD:223738	27.82	133	85	2	45	172	99	225	4.33E-08	48.3	COG0666	Arp	FOG: Ankyrin repeat
LN02_07641|LN02Chr05:4710480-4712926(+)|786	CDD:227476	26.282	468	300	12	4	462	18	449	4.40E-61	211	COG5147	REB1	Myb superfamily proteins, including transcription factors and mRNA splicing factors
LN02_07769|LN02Chr05:5185451-5190589(-)|1607	CDD:223589	30.476	210	123	8	768	961	73	275	3.77E-28	115	COG0515	SPS1	Serine/threonine protein kinase
LN02_07769|LN02Chr05:5185451-5190589(-)|1607	CDD:223589	20.29	345	230	10	284	599	2	330	3.69E-19	88.6	COG0515	SPS1	Serine/threonine protein kinase
LN02_07769|LN02Chr05:5185451-5190589(-)|1607	CDD:223202	21.381	449	281	24	1032	1457	30	429	5.00E-26	110	COG0124	HisS	Histidyl-tRNA synthetase
LN02_07897|LN02Chr05:5632292-5632809(-)|108	CDD:224032	30.137	73	40	3	8	69	201	273	1.17E-04	36.6	COG1107	COG1107	Archaea-specific RecJ-like exonuclease, contains DnaJ-type Zn finger domain
LN02_07961|LN02Chr05:5804654-5808777(-)|1268	CDD:225186	19.649	626	345	25	39	599	7	539	4.22E-20	92.9	COG2303	BetA	Choline dehydrogenase and related flavoproteins
LN02_08345|LN02Chr06:2175048-2175680(-)|210	CDD:224336	23.611	216	136	6	11	208	15	219	4.79E-20	81.7	COG1418	COG1418	Predicted HD superfamily hydrolase
LN02_08409|LN02Chr06:2489584-2491235(-)|514	CDD:223457	50.321	467	222	5	17	478	15	476	0	642	COG0380	OtsA	Trehalose-6-phosphate synthase
LN02_08793|LN02Chr07:366868-371768(-)|1471	CDD:224732	21.789	436	287	12	968	1394	1	391	1.57E-50	181	COG1819	COG1819	Glycosyl transferases, related to UDP-glucuronosyltransferase
LN02_08921|LN02Chr07:811435-812924(+)|359	CDD:225201	29.667	300	197	8	64	357	148	439	3.36E-43	153	COG2319	COG2319	FOG: WD40 repeat
LN02_08985|LN02Chr07:1033766-1035334(-)|484	CDD:227517	53.552	183	85	0	302	484	200	382	9.73E-74	235	COG5190	FCP1	TFIIF-interacting CTD phosphatases, including NLI-interacting factor
LN02_09049|LN02Chr07:1263347-1264159(+)|208	CDD:225546	35.135	74	47	1	93	166	101	173	2.35E-11	58.6	COG3000	ERG3	Sterol desaturase
LN02_09177|LN02Chr07:1649145-1651861(+)|770	CDD:227430	28.533	375	256	8	370	737	387	756	3.28E-80	270	COG5099	COG5099	RNA-binding protein of the Puf family, translational repressor
LN02_09241|LN02Chr07:1903959-1913271(-)|2419	CDD:223715	37.091	275	154	8	1896	2169	77	333	8.43E-60	207	COG0642	BaeS	Signal transduction histidine kinase
LN02_09241|LN02Chr07:1903959-1913271(-)|2419	CDD:226415	21.131	937	572	37	589	1485	19	828	6.59E-39	155	COG3899	COG3899	Predicted ATPase
LN02_09241|LN02Chr07:1903959-1913271(-)|2419	CDD:223855	31.852	135	83	3	2215	2348	1	127	3.62E-26	103	COG0784	CheY	FOG: CheY-like receiver
LN02_09241|LN02Chr07:1903959-1913271(-)|2419	CDD:223589	22.186	311	223	7	180	475	48	354	6.41E-17	82.5	COG0515	SPS1	Serine/threonine protein kinase

LN02_09241∣LN02Chr07:1903959-1913271(-)∣2419	CDD:225113	18.889	90	68	3	1802	1889	89	175	2.04E-06	47.3	COG2203	FhlA	FOG: GAF domain
LN02_00154∣LN02Chr01:736594-737414(+)∣222	CDD:223316	26.761	71	49	2	129	199	7	74	1.78E-07	44.6	COG0238	RpsR	Ribosomal protein S18
LN02_00282∣LN02Chr01:1550596-1553353(+)∣834	CDD:225488	30.435	414	252	8	181	585	269	655	1.92E-72	251	COG2937	PlsB	Glycerol-3-phosphate O-acyltransferase
LN02_00346∣LN02Chr01:1848555-1855550(+)∣2271	CDD:224037	29.706	340	194	10	728	1063	454	752	9.46E-40	157	COG1112	COG1112	Superfamily I DNA and RNA helicases and helicase subunits
LN02_00346∣LN02Chr01:1848555-1855550(+)∣2271	CDD:223540	20.465	430	278	17	1598	2004	25	413	2.03E-12	69.1	COG0464	SpoVK	ATPases of the AAA+ class
LN02_00346∣LN02Chr01:1848555-1855550(+)∣2271	CDD:223540	22.143	420	256	17	1308	1705	12	382	3.16E-10	62.1	COG0464	SpoVK	ATPases of the AAA+ class
LN02_00410∣LN02Chr01:2051516-2052124(-)∣136	CDD:226314	32.8	125	76	4	1	124	2	119	1.69E-25	91	COG3791	COG3791	Uncharacterized conserved protein
LN02_00730∣LN02Chr01:3192972-3194600(+)∣479	CDD:223588	38.876	427	221	11	16	440	8	396	1.82E-132	392	COG0514	RecQ	Superfamily II DNA helicase
LN02_00794∣LN02Chr01:3435292-3437874(-)∣472	CDD:223464	37.466	371	212	5	97	461	12	368	4.85E-93	283	COG0387	ChaA	Ca2+/H+ antiporter
LN02_00858∣LN02Chr01:3602831-3603863(-)∣318	CDD:223262	35.849	106	40	3	202	307	10	87	5.46E-21	83	COG0184	RpsO	Ribosomal protein S15P/S13E
LN02_00986∣LN02Chr01:4011034-4012855(-)∣532	CDD:223553	16.294	313	236	3	61	372	41	328	7.08E-06	45.1	COG0477	ProP	Permeases of the major facilitator superfamily
LN02_00986∣LN02Chr01:4011034-4012855(-)∣532	CDD:224208	20.325	123	65	5	375	491	338	433	6.59E-04	39.3	COG1289	COG1289	Predicted membrane protein
LN02_01370∣LN02Chr01:5466204-5467742(+)∣309	CDD:226735	22.222	207	149	7	27	226	4	205	1.11E-12	63.6	COG4285	COG4285	Uncharacterized conserved protein
LN02_01498∣LN02Chr01:6001934-6003985(+)∣467	CDD:225486	24.832	298	146	8	15	303	9	237	9.54E-37	132	COG2935	COG2935	Putative arginyl-tRNA:protein arginylyltransferase
LN02_01562∣LN02Chr01:6263162-6265106(-)∣567	CDD:225490	28.866	194	110	7	87	261	66	250	7.67E-32	126	COG2939	COG2939	Carboxypeptidase C (cathepsin A)
LN02_01754∣LN02Chr01:6932329-6933332(-)∣277	CDD:224500	44.103	195	99	3	63	256	18	203	5.40E-50	161	COG1584	COG1584	Predicted membrane protein
LN02_01818∣LN02Chr01:7125365-7127042(-)∣466	CDD:225051	34.536	194	119	5	240	430	1	189	2.13E-48	162	COG2140	COG2140	Thermophilic glucose-6-phosphate isomerase and related metalloenzymes
LN02_01818∣LN02Chr01:7125365-7127042(-)∣466	CDD:225051	27.206	136	90	3	123	255	63	192	8.02E-11	58.6	COG2140	COG2140	Thermophilic glucose-6-phosphate isomerase and related metalloenzymes
LN02_01882∣LN02Chr01:7309515-7310975(-)∣396	CDD:223261	40.842	404	211	13	6	395	3	392	4.15E-105	313	COG0183	PaaJ	Acetyl-CoA acetyltransferase
LN02_01946∣LN02Chr02:69944-71324(-)∣372	CDD:225143	23.596	267	168	11	129	370	144	399	5.77E-28	111	COG2234	Iap	Predicted aminopeptidases
LN02_02074∣LN02Chr02:522932-524039(+)∣339	CDD:223796	26.786	168	118	3	1	166	83	247	4.21E-14	68.8	COG0724	COG0724	RNA-binding proteins (RRM domain)

LN02_02138∣LN02Chr02:714766-717555(+)∣699	CDD:223589	28.647	377	228	10	290	630	1	372	6.65E-59	201	COG0515	SPS1	Serine/threonine protein kinase
LN02_02202∣LN02Chr02:917479-918877(-)∣329	CDD:223999	22.143	280	205	7	46	317	18	292	1.11E-23	95.9	COG1073	COG1073	Hydrolases of the alpha/beta superfamily
LN02_02266∣LN02Chr02:1168382-1169051(-)∣129	CDD:223263	43.21	81	41	3	48	128	1	76	3.90E-28	96.1	COG0185	RpsS	Ribosomal protein S19
LN02_02394∣LN02Chr02:1686125-1688296(-)∣723	CDD:223587	24.727	550	303	14	154	694	25	472	4.72E-67	227	COG0513	SrmB	Superfamily II DNA and RNA helicases
LN02_02458∣LN02Chr02:1872319-1875823(-)∣997	CDD:225201	22.006	309	187	8	631	939	189	443	6.12E-15	75.5	COG2319	COG2319	FOG: WD40 repeat
LN02_02458∣LN02Chr02:1872319-1875823(-)∣997	CDD:227367	23.03	165	96	9	19	161	112	267	5.92E-06	46.1	COG5034	TNG2	Chromatin remodeling protein, contains PhD zinc finger
LN02_02522∣LN02Chr02:2098748-2100094(-)∣175	CDD:223140	33.684	95	58	3	47	140	110	200	9.68E-17	71.5	COG0062	COG0062	Uncharacterized conserved protein
LN02_02650∣LN02Chr02:2552795-2554163(+)∣382	CDD:225369	26.124	356	203	13	38	371	9	326	1.11E-28	114	COG2812	DnaX	DNA polymerase III, gamma/tau subunits
LN02_02714∣LN02Chr02:2761611-2762585(+)∣291	CDD:223959	32.857	280	153	8	13	286	1	251	1.01E-52	170	COG1028	FabG	Dehydrogenases with different specificities (related to short-chain alcohol dehydrogenases)
LN02_02906∣LN02Chr02:3424777-3427389(+)∣787	CDD:225597	21.384	159	83	6	110	231	83	236	3.11E-04	40.8	COG3055	COG3055	Uncharacterized protein conserved in bacteria
LN02_02970∣LN02Chr02:3635483-3636717(+)∣319	CDD:224170	47.241	290	144	3	31	319	2	283	6.73E-111	321	COG1250	FadB	3-hydroxyacyl-CoA dehydrogenase
LN02_03034∣LN02Chr02:3883205-3883922(+)∣123	CDD:223831	36.29	124	68	3	6	122	140	259	3.71E-10	52.8	COG0760	SurA	Parvulin-like peptidyl-prolyl isomerase
LN02_03418∣LN02Chr02:5141258-5145380(+)∣1168	CDD:225491	28.723	188	101	4	1012	1168	295	480	7.10E-21	94.5	COG2940	COG2940	Proteins containing SET domain
LN02_03482∣LN02Chr02:5738435-5742276(+)∣1254	CDD:223550	29.86	931	557	16	135	1046	51	904	0	626	COG0474	MgtA	Cation transport ATPase
LN02_03546∣LN02Chr02:6096645-6098951(+)∣626	CDD:225789	24.545	440	274	17	67	471	13	429	9.22E-42	159	COG3250	LacZ	Beta-galactosidase/beta-glucuronidase
LN02_03610∣LN02Chr02:6660661-6662151(-)∣496	CDD:225490	23.517	489	295	18	31	480	32	480	9.03E-38	141	COG2939	COG2939	Carboxypeptidase C (cathepsin A)
LN02_03674∣LN02Chr03:267617-269895(-)∣523	CDD:223605	18.605	473	364	7	44	516	9	460	1.81E-29	117	COG0531	PotE	Amino acid transporters
LN02_03802∣LN02Chr03:791745-793839(+)∣589	CDD:223179	14.754	427	177	8	105	506	2	266	2.16E-33	125	COG0101	TruA	Pseudouridylate synthase
LN02_03866∣LN02Chr03:965060-966667(-)∣368	CDD:223549	47.802	364	167	11	1	360	1	345	8.31E-153	431	COG0473	LeuB	Isocitrate/isopropylmalate dehydrogenase
LN02_03994∣LN02Chr03:1359317-1360699(-)∣317	CDD:223483	24.413	213	110	8	5	216	4	166	9.67E-23	91	COG0406	phoE	Broad specificity phosphatase PhoE and related phosphatases
LN02_04058∣LN02Chr03:1706646-1708659(-)∣563	CDD:227513	43.894	565	298	8	1	563	1	548	0	602	COG5186	PAP1	Poly(A) polymerase

LN02_04250 ǀ LN02Chr03 :3549743-3550195(-) ǀ 128	CDD:223264	39.726	73	43	1	16	88	11	82	1.75E-11	53.4	COG0186	RpsQ	Ribosomal protein S17
LN02_04314 ǀ LN02Chr03 :3748976-3753803(-) ǀ 1486	CDD:227371	33.412	1266	737	27	10	1249	7	1192	0	864	COG5038	COG5038	Ca2+-dependent lipid-binding protein, contains C2 domain
LN02_04378 ǀ LN02Chr03 :3974617-3975691(-) ǀ 270	CDD:223635	31.696	224	133	6	23	240	8	217	1.04E-25	99.4	COG0561	Cof	Predicted hydrolases of the HAD superfamily
LN02_04506 ǀ LN02Chr03 :4392421-4393007(+) ǀ 135	CDD:225029	38.525	122	69	1	1	122	1	116	2.64E-17	69.7	COG2118	COG2118	DNA-binding protein
LN02_04570 ǀ LN02Chr03 :4630263-4631522(+) ǀ 419	CDD:225201	20.084	239	135	7	174	375	84	303	1.45E-04	40.8	COG2319	COG2319	FOG: WD40 repeat
LN02_04634 ǀ LN02Chr03 :4868224-4869114(+) ǀ 296	CDD:223532	33.929	56	33	2	178	229	98	153	7.47E-07	45.8	COG0456	RimI	Acetyltransferases
LN02_04698 ǀ LN02Chr03 :5099288-5101000(+) ǀ 521	CDD:227411	22.222	243	135	11	182	372	7	247	7.01E-06	45.7	COG5079	SAC3	Nuclear protein export factor
LN02_04826 ǀ LN02Chr03 :5552664-5554231(+) ǀ 438	CDD:227408	26.667	90	63	1	343	432	166	252	1.33E-11	62.5	COG5076	COG5076	Transcription factor involved in chromatin remodeling, contains bromodomain
LN02_05146 ǀ LN02Chr04 :746195-750628(+) ǀ 1357	CDD:223124	32.099	810	421	24	179	985	13	696	0	648	COG0046	PurL	Phosphoribosylformylglycinamidine (FGAM) synthase, synthetase domain
LN02_05146 ǀ LN02Chr04 :746195-750628(+) ǀ 1357	CDD:223125	39.245	265	122	12	1096	1357	3	231	1.66E-77	252	COG0047	PurL	Phosphoribosylformylglycinamidine (FGAM) synthase, glutamine amidotransferase domain
LN02_05274 ǀ LN02Chr04 :1182821-1185473(+) ǀ 737	CDD:224117	23.661	224	150	3	39	241	703	926	1.04E-09	59	COG1196	Smc	Chromosome segregation ATPases
LN02_05402 ǀ LN02Chr04 :1634945-1635973(-) ǀ 259	CDD:224997	44.318	264	134	4	4	259	1	259	4.58E-87	256	COG2086	FixA	Electron transfer flavoprotein, beta subunit
LN02_05594 ǀ LN02Chr04 :2574455-2577268(+) ǀ 837	CDD:223547	25.212	472	326	9	372	830	4	461	1.15E-81	267	COG0471	CitT	Di- and tricarboxylate transporters
LN02_05594 ǀ LN02Chr04 :2574455-2577268(+) ǀ 837	CDD:227695	26.403	303	172	13	1	259	1	296	3.19E-33	127	COG5408	COG5408	SPX domain-containing protein
LN02_05722 ǀ LN02Chr04 :2994280-2996610(+) ǀ 776	CDD:224117	21.429	252	175	5	433	668	227	471	8.43E-08	52.8	COG1196	Smc	Chromosome segregation ATPases
LN02_05786 ǀ LN02Chr04 :3192286-3193335(-) ǀ 274	CDD:224754	40	55	33	0	115	169	1	55	2.03E-15	66	COG1841	RpmD	Ribosomal protein L30/L7E
LN02_06106 ǀ LN02Chr04 :4290072-4291392(+) ǀ 414	CDD:223173	33.935	277	134	10	49	313	9	248	2.66E-45	154	COG0095	LplA	Lipoate-protein ligase A
LN02_06170 ǀ LN02Chr04 :4540039-4543134(+) ǀ 799	CDD:226406	22.892	166	111	6	217	377	694	847	1.13E-05	46	COG3889	COG3889	Predicted solute binding protein
LN02_06298 ǀ LN02Chr04 :4944767-4950392(-) ǀ 1811	CDD:223613	21.622	555	362	21	346	887	15	509	3.05E-49	182	COG0539	RpsA	Ribosomal protein S1
LN02_06298 ǀ LN02Chr04 :4944767-4950392(-) ǀ 1811	CDD:223613	23.186	565	365	20	810	1367	11	513	1.33E-41	159	COG0539	RpsA	Ribosomal protein S1
LN02_06554 ǀ LN02Chr04 :5823698-5825458(+) ǀ 562	CDD:225482	31.455	213	94	4	260	470	44	206	3.20E-36	132	COG2930	COG2930	Uncharacterized conserved protein

LN02_06618	LN02Chr05 :122320-124138(-)	538	CDD:2250 35	21.834	229	133	6	277	505	210	392	4.84E-12	64.8	COG21 24	CypX	Cytochrome P450
LN02_06874	LN02Chr05 :1099571-1101735(-)	707	CDD:2237 96	29.293	198	117	4	32	207	103	299	5.08E-12	64.6	COG07 24	COG072 4	RNA-binding proteins (RRM domain)
LN02_06874	LN02Chr05 :1099571-1101735(-)	707	CDD:2237 96	27.451	102	74	0	265	366	74	175	4.26E-11	61.5	COG07 24	COG072 4	RNA-binding proteins (RRM domain)
LN02_06938	LN02Chr05 :1608119-1610363(-)	625	CDD:2235 56	41.547	698	304	8	9	615	7	691	0	679	COG04 80	FusA	Translation elongation factors (GTPases)
LN02_07066	LN02Chr05 :2447468-2449432(-)	517	CDD:2232 34	27.685	419	264	8	91	507	7	388	2.99E-84	263	COG01 56	BioF	7-keto-8-aminopelargonate synthetase and related enzymes
LN02_07130	LN02Chr05 :2662599-2666155(-)	1144	CDD:2238 20	19.886	352	259	14	675	1015	2	341	1.78E-44	168	COG07 49	PolA	DNA polymerase I - 3'-5' exonuclease and polymerase domains
LN02_07258	LN02Chr05 :3123447-3125930(+)	615	CDD:2234 42	24.399	582	295	22	79	578	1	519	1.27E-61	210	COG03 65	Acs	Acyl-coenzyme A synthetases/AMP-(fatty) acid ligases
LN02_07386	LN02Chr05 :3593010-3594606(-)	489	CDD:2275 53	41.801	311	138	3	107	417	9	276	1.35E-95	288	COG52 28	POP2	mRNA deadenylase subunit
LN02_07450	LN02Chr05 :3818887-3820272(-)	323	CDD:2246 11	36.598	194	107	8	21	205	92	278	8.81E-37	132	COG16 97	COG169 7	DNA topoisomerase VI, subunit A
LN02_07514	LN02Chr05 :4035985-4036791(+)	238	CDD:2245 27	28.774	212	123	8	17	224	8	195	6.62E-36	123	COG16 11	COG161 1	Predicted Rossmann fold nucleotide-binding protein
LN02_07642	LN02Chr05 :4713344-4715732(-)	631	CDD:2253 71	31.915	141	96	0	164	304	49	189	5.73E-19	86.5	COG28 14	AraJ	Arabinose efflux permease
LN02_07642	LN02Chr05 :4713344-4715732(-)	631	CDD:2235 53	19.939	326	252	4	134	452	7	330	8.99E-10	57.8	COG04 77	ProP	Permeases of the major facilitator superfamily
LN02_07770	LN02Chr05 :5191201-5193239(+)	654	CDD:2241 17	19.557	271	193	8	183	439	209	468	1.99E-07	51.3	COG11 96	Smc	Chromosome segregation ATPases
LN02_08090	LN02Chr06 :1212256-1215989(+)	1081	CDD:2275 40	18.027	527	346	22	7	489	27	511	5.41E-05	44.2	COG52 15	KAP95	Karyopherin (importin) beta
LN02_08346	LN02Chr06 :2177544-2179649(+)	567	CDD:2231 54	28.645	391	260	6	115	494	32	414	5.09E-89	279	COG00 76	GadB	Glutamate decarboxylase and related PLP-dependent proteins
LN02_08410	LN02Chr06 :2492813-2495522(+)	819	CDD:2234 26	31.559	263	160	6	239	500	14	257	1.54E-58	200	COG03 49	Rnd	Ribonuclease D
LN02_08474	LN02Chr06 :2725397-2737363(-)	2686	CDD:2275 96	20.851	470	328	15	480	933	3834	4275	3.59E-17	86.2	COG52 71	MDN1	AAA ATPase containing von Willebrand factor type A (vWA) domain
LN02_08474	LN02Chr06 :2725397-2737363(-)	2686	CDD:2275 96	20.913	526	356	18	168	673	3833	4318	5.25E-13	72.3	COG52 71	MDN1	AAA ATPase containing von Willebrand factor type A (vWA) domain
LN02_08474	LN02Chr06 :2725397-2737363(-)	2686	CDD:2275 96	19.312	523	327	19	1625	2085	3816	4305	1.15E-10	64.6	COG52 71	MDN1	AAA ATPase containing von Willebrand factor type A (vWA) domain
LN02_08474	LN02Chr06 :2725397-2737363(-)	2686	CDD:2275 96	18.484	541	372	17	966	1459	3800	4318	2.53E-10	63.5	COG52 71	MDN1	AAA ATPase containing von Willebrand factor type A (vWA) domain
LN02_08538	LN02Chr06 :2904129-2906432(-)	628	CDD:2279 52	29.28	625	353	13	11	626	1	545	3.95E-122	370	COG56 65	NOT5	CCR4-NOT transcriptional regulation complex, NOT5 subunit
LN02_08794	LN02Chr07 :373474-374586(+)	304	CDD:2245 84	21.477	149	101	7	112	253	46	185	1.23E-04	39.2	COG16 70	RimL	Acetyltransferases, including N-acetylases of ribosomal proteins

LN02_08986|LN02Chr07:1038619-1041260(+)|812	CDD:226646	26.183	634	388	17	145	752	25	604	7.17E-92	298	COG4178	COG4178	ABC-type uncharacterized transport system, permease and ATPase components
LN02_09114|LN02Chr07:1461699-1465153(-)|993	CDD:227600	33.953	215	132	5	184	396	64	270	7.91E-46	163	COG5275	COG5275	BRCT domain type II
LN02_09114|LN02Chr07:1461699-1465153(-)|993	CDD:225165	27.273	77	41	2	481	557	52	113	1.54E-04	42.2	COG2256	MGS1	ATPase related to the helicase subunit of the Holliday junction resolvase
LN02_09242|LN02Chr07:1916065-1918143(-)|530	CDD:224153	22.547	479	311	22	10	469	3	440	1.34E-35	135	COG1232	HemY	Protoporphyrinogen oxidase
LN02_00219|LN02Chr01:1175933-1177423(+)|475	CDD:223540	22.967	209	144	6	212	409	247	449	4.57E-16	77.6	COG0464	SpoVK	ATPases of the AAA+ class
LN02_00283|LN02Chr01:1553686-1554858(-)|390	CDD:223491	43.072	332	137	8	50	379	2	283	1.02E-106	313	COG0414	PanC	Panthothenate synthetase
LN02_00411|LN02Chr01:2052861-2054311(-)|463	CDD:226027	25.532	141	88	3	4	144	54	177	1.57E-14	70.6	COG3496	COG3496	Uncharacterized conserved protein
LN02_00475|LN02Chr01:2230880-2231772(-)|237	CDD:223998	27.536	207	134	5	17	223	75	265	2.05E-43	145	COG1072	CoaA	Panthothenate kinase
LN02_00603|LN02Chr01:2667161-2668057(-)|298	CDD:226413	24.051	237	117	8	50	286	45	218	6.44E-19	80.5	COG3897	COG3897	Predicted methyltransferase
LN02_00795|LN02Chr01:3439239-3441215(-)|520	CDD:227785	39.502	562	289	10	1	518	201	755	2.00E-165	484	COG5498	ACF2	Predicted glycosyl hydrolase
LN02_00859|LN02Chr01:3604312-3605446(+)|279	CDD:223130	27.273	253	131	7	16	220	5	252	3.04E-63	196	COG0052	RpsB	Ribosomal protein S2
LN02_01051|LN02Chr01:4228229-4229777(-)|409	CDD:227535	20.408	196	132	7	101	283	256	440	8.81E-04	38.2	COG5210	COG5210	GTPase-activating protein
LN02_01115|LN02Chr01:4470461-4472384(-)|553	CDD:223244	47.347	490	210	9	52	540	3	445	0	513	COG0166	Pgi	Glucose-6-phosphate isomerase
LN02_01179|LN02Chr01:4790925-4794629(-)|1142	CDD:225606	23.846	130	91	3	146	275	133	254	3.04E-04	41.5	COG3064	TolA	Membrane protein involved in colicin uptake
LN02_01371|LN02Chr01:5469489-5470538(-)|349	CDD:227381	32.857	70	47	0	190	259	32	101	2.66E-05	42.8	COG5048	COG5048	FOG: Zn-finger
LN02_01499|LN02Chr01:6004788-6006041(+)|376	CDD:225034	26.715	277	137	11	7	267	4	230	8.80E-25	99.4	COG2123	COG2123	RNase PH-related exoribonuclease
LN02_01627|LN02Chr01:6520660-6521641(+)|279	CDD:227548	36.434	129	72	1	2	130	3	121	1.26E-23	93.6	COG5223	COG5223	Uncharacterized conserved protein
LN02_01691|LN02Chr01:6765357-6769313(-)|1134	CDD:224117	26.797	153	102	3	612	760	693	839	2.01E-06	48.9	COG1196	Smc	Chromosome segregation ATPases
LN02_01883|LN02Chr01:7311959-7315264(+)|984	CDD:223556	23.341	844	492	26	122	957	1	697	3.40E-143	440	COG0480	FusA	Translation elongation factors (GTPases)
LN02_01947|LN02Chr02:74365-76232(-)|534	CDD:225371	20.782	409	254	15	53	457	5	347	1.40E-10	59.9	COG2814	AraJ	Arabinose efflux permease
LN02_02075|LN02Chr02:524349-525732(-)|377	CDD:225201	25.806	155	110	3	119	270	134	286	3.02E-10	58.6	COG2319	COG2319	FOG: WD40 repeat
LN02_02459|LN02Chr02:1876475-1877519(+)|248	CDD:224910	28.205	195	102	4	73	239	16	200	2.80E-42	140	COG1999	COG1999	Uncharacterized protein SCO1/SenC/PrrC, involved in biogenesis

														of respiratory and photosynthetic systems
LN02_02523ǀLN02Chr02:2100542-2101035(+)ǀ114	CDD:223306	45.055	91	41	1	3	93	2	83	9.60E-32	104	COG0228	RpsP	Ribosomal protein S16
LN02_02779ǀLN02Chr02:2985297-2986803(-)ǀ453	CDD:224368	48.148	27	14	0	91	117	170	196	7.31E-06	44	COG1451	COG1451	Predicted metal-dependent hydrolase
LN02_02843ǀLN02Chr02:3191454-3192587(-)ǀ200	CDD:225074	28.125	128	88	2	49	176	2	125	1.34E-19	77.8	COG2163	RPL14A	Ribosomal protein L14E/L6E/L27E
LN02_02907ǀLN02Chr02:3429485-3432758(-)ǀ899	CDD:227516	24.346	497	256	13	387	871	33	421	1.27E-37	143	COG5189	SFP1	Putative transcriptional repressor regulating G2/M transition
LN02_03035ǀLN02Chr02:3884366-3885693(-)ǀ179	CDD:224277	26.667	120	78	2	48	167	1	110	1.77E-21	81.6	COG1358	RPL8A	Ribosomal protein HS6-type (S12/L30/L7a)

LN02_03227ǀLN02Chr02:4558527-4560593(-)ǀ558	CDD:223354	35.546	467	277	12	103	556	1	456	1.98E-113	342	COG0277	GlcD	FAD/FMN-containing dehydrogenases
LN02_03291ǀLN02Chr02:4760316-4762007(-)ǀ472	CDD:227361	23	100	58	4	338	437	4	84	0.001	38.6	COG5028	COG5028	Vesicle coat complex COPII, subunit SEC24/subunit SFB2/subunit SFB3
LN02_03355ǀLN02Chr02:4966393-4969434(+)ǀ616	CDD:225646	20.992	524	331	18	46	549	4	464	3.97E-16	78.5	COG3104	PTR2	Dipeptide/tripeptide permease
LN02_03483ǀLN02Chr02:5743066-5747097(-)ǀ944	CDD:224055	28.148	540	360	9	407	944	52	565	1.09E-77	261	COG1132	MdlB	ABC-type multidrug transport system, ATPase and permease components
LN02_03483ǀLN02Chr02:5743066-5747097(-)ǀ944	CDD:224055	18.639	338	237	6	5	312	37	366	3.51E-23	102	COG1132	MdlB	ABC-type multidrug transport system, ATPase and permease components
LN02_03739ǀLN02Chr03:487400-489501(-)ǀ520	CDD:225035	18.985	453	295	13	56	501	24	411	9.09E-26	106	COG2124	CypX	Cytochrome P450
LN02_04251ǀLN02Chr03:3551100-3552252(+)ǀ282	CDD:224670	39.13	230	125	7	55	282	7	223	1.35E-77	232	COG1756	Mra1	Uncharacterized conserved protein
LN02_04315ǀLN02Chr03:3756738-3758133(-)ǀ411	CDD:227827	35.714	42	27	0	362	403	325	366	1.66E-04	40.4	COG5540	COG5540	RING-finger-containing ubiquitin ligase
LN02_04379ǀLN02Chr03:3976453-3979616(-)ǀ1018	CDD:223604	28.169	142	102	0	108	249	7	148	9.60E-28	112	COG0530	ECM27	Ca2+/Na+ antiporter
LN02_04379ǀLN02Chr03:3976453-3979616(-)ǀ1018	CDD:223604	24.427	131	87	1	817	935	137	267	3.21E-17	81.1	COG0530	ECM27	Ca2+/Na+ antiporter
LN02_04443ǀLN02Chr03:4183984-4185017(-)ǀ297	CDD:223282	32.911	237	139	8	45	270	1	228	4.66E-39	135	COG0204	PlsC	1-acyl-sn-glycerol-3-phosphate acyltransferase
LN02_04507ǀLN02Chr03:4393323-4394296(-)ǀ285	CDD:227364	37.021	235	139	5	39	268	5	235	7.76E-78	233	COG5031	COQ4	Uncharacterized protein involved in ubiquinone biosynthesis
LN02_04763ǀLN02Chr03:5328556-5329532(+)ǀ160	CDD:224986	48.485	66	34	0	1	66	1	66	1.69E-23	84.7	COG2075	RPL24A	Ribosomal protein L24E
LN02_04891ǀLN02Chr03:5885099-5886563(-)ǀ462	CDD:224117	18.085	282	217	3	165	446	675	942	2.23E-11	63.2	COG1196	Smc	Chromosome segregation ATPases
LN02_05083ǀLN02Chr04:556339-558751(-)ǀ722	CDD:223662	20.29	207	102	4	484	688	6	151	4.99E-11	58.8	COG0589	UspA	Universal stress protein UspA and related nucleotide-binding proteins

LN02_05403∣LN02Chr04:1636398-1637784(-)∣386	CDD:227651	24.159	327	212	8	7	320	1	304	2.00E-44	153	COG5347	COG5347	GTPase-activating protein that regulates ARFs (ADP-ribosylation factors), involved in ARF-mediated vesicular transport
LN02_05467∣LN02Chr04:1936056-1938681(+)∣826	CDD:223587	33.902	469	292	8	49	513	28	482	3.56E-124	380	COG0513	SrmB	Superfamily II DNA and RNA helicases
LN02_05595∣LN02Chr04:2577864-2581417(+)∣1100	CDD:227709	19.653	346	208	13	14	336	836	1134	2.37E-04	42.2	COG5422	ROM1	RhoGEF, Guanine nucleotide exchange factor for Rho/Rac/Cdc42-like GTPases
LN02_05787∣LN02Chr04:3194157-3194853(+)∣149	CDD:223178	50	112	47	2	27	138	19	121	1.19E-43	137	COG0100	RpsK	Ribosomal protein S11
LN02_05915∣LN02Chr04:3607382-3610578(-)∣986	CDD:227438	26.887	636	367	18	127	756	2	545	3.90E-100	326	COG5107	RNA14	Pre-mRNA 3'-end processing (cleavage and polyadenylation) factor
LN02_06043∣LN02Chr04:4098335-4099871(-)∣466	CDD:227417	20.588	136	104	4	246	378	85	219	2.55E-14	69.2	COG5085	COG5085	Predicted membrane protein
LN02_06235∣LN02Chr04:4745239-4748847(+)∣1202	CDD:223336	26.389	144	98	6	893	1033	80	218	1.96E-21	93.5	COG0258	Exo	5'-3' exonuclease (including N-terminal domain of PolI)
LN02_06235∣LN02Chr04:4745239-4748847(+)∣1202	CDD:223336	29.268	82	52	2	14	90	1	81	5.93E-08	52.7	COG0258	Exo	5'-3' exonuclease (including N-terminal domain of PolI)
LN02_06363∣LN02Chr04:5154130-5156256(+)∣708	CDD:227604	22.581	155	103	5	403	550	165	309	1.70E-15	76.9	COG5279	CYK3	Uncharacterized protein involved in cytokinesis, contains TGc (transglutaminase/protease-like) domain
LN02_06427∣LN02Chr04:5360013-5361578(-)∣471	CDD:223443	20.588	374	218	12	27	331	3	366	4.15E-35	133	COG0366	AmyA	Glycosidases
LN02_06619∣LN02Chr05:124688-128753(+)∣1312	CDD:223951	29.401	551	340	17	137	667	118	639	1.91E-84	286	COG1020	EntF	Non-ribosomal peptide synthetase modules and related proteins
LN02_06619∣LN02Chr05:124688-128753(+)∣1312	CDD:225857	32.576	264	149	10	920	1172	2	247	7.55E-46	167	COG3320	COG3320	Putative dehydrogenase domain of multifunctional non-ribosomal peptide synthetases and related enzymes
LN02_06811∣LN02Chr05:880018-881235(+)∣356	CDD:223490	51.685	267	127	1	75	341	1	265	1.44E-120	346	COG0413	PanB	Ketopantoate hydroxymethyltransferase
LN02_06875∣LN02Chr05:1102184-1105403(+)∣1042	CDD:227543	23.869	884	554	17	53	926	11	785	7.22E-135	426	COG5218	YCG1	Chromosome condensation complex Condensin, subunit G
LN02_07067∣LN02Chr05:2450408-2452570(+)∣402	CDD:227409	25.926	324	174	17	94	383	187	478	2.20E-23	99	COG5077	COG5077	Ubiquitin carboxyl-terminal hydrolase
LN02_07195∣LN02Chr05:2879733-2881778(-)∣634	CDD:224250	27.329	161	95	8	151	305	444	588	3.78E-06	47	COG1331	COG1331	Highly conserved protein containing a thioredoxin domain
LN02_07451∣LN02Chr05:3821363-3823755(+)∣745	CDD:224571	29.663	563	346	12	177	738	1	514	3.25E-109	339	COG1657	SqhC	Squalene cyclase
LN02_07771∣LN02Chr05:5193622-5194587(+)∣296	CDD:223900	27.016	248	150	5	55	296	7	229	3.01E-30	111	COG0830	UreF	Urease accessory protein UreF

LN02_07899∣LN02Chr05:5638080-5639804(+)∣533	CDD:224117	21.081	185	141	2	49	232	200	380	1.15E-10	61.3	COG1196	Smc	Chromosome segregation ATPases
LN02_07963∣LN02Chr05:5811493-5813896(+)∣686	CDD:226582	18.826	494	294	19	198	663	3	417	1.73E-12	66.7	COG4097	COG4097	Predicted ferric reductase
LN02_08347∣LN02Chr06:2184405-2185674(-)∣375	CDD:224117	23.636	110	77	2	220	322	301	410	2.70E-04	40.1	COG1196	Smc	Chromosome segregation ATPases
LN02_08411∣LN02Chr06:2497531-2498139(-)∣156	CDD:223709	40.323	62	37	0	89	150	16	77	9.14E-10	49.6	COG0636	AtpE	F0F1-type ATP synthase, subunit c/Archaeal/vacuolar-type H+-ATPase, subunit K
LN02_08539∣LN02Chr06:2907333-2908167(+)∣159	CDD:224407	50.34	147	70	2	1	147	1	144	1.72E-65	193	COG1490	Dtd	D-Tyr-tRNAtyr deacylase
LN02_08795∣LN02Chr07:374923-376552(-)∣520	CDD:225201	20	325	215	12	174	491	149	435	1.07E-14	73.2	COG2319	COG2319	FOG: WD40 repeat
LN02_08987∣LN02Chr07:1042613-1043773(+)∣323	CDD:223729	36.156	307	155	4	1	306	2	268	1.79E-106	309	COG0656	ARA1	Aldo/keto reductases, related to diketogulonate reductase
LN02_09051∣LN02Chr07:1265579-1266696(-)∣164	CDD:224961	24.823	141	99	5	5	143	1	136	1.65E-21	82	COG2050	PaaI	HGG motif-containing thioesterase, possibly involved in aromatic compounds catabolism
LN02_09115∣LN02Chr07:1465850-1466519(+)∣173	CDD:223275	34.375	128	80	4	1	128	13	136	5.72E-27	96.5	COG0197	RplP	Ribosomal protein L16/L10E
LN02_09179∣LN02Chr07:1653820-1656033(+)∣587	CDD:223704	20.325	246	132	8	198	443	37	218	4.28E-24	99	COG0631	PTC1	Serine/threonine protein phosphatase
LN02_09243∣LN02Chr07:1923599-1925012(+)∣398	CDD:223769	15.702	242	192	4	92	329	55	288	7.57E-04	37.9	COG0697	RhaT	Permeases of the drug/metabolite transporter (DMT) superfamily
LN02_00092∣LN02Chr01:460097-462429(+)∣746	CDD:223540	21.154	156	112	6	490	635	272	426	6.59E-07	49.4	COG0464	SpoVK	ATPases of the AAA+ class
LN02_00156∣LN02Chr01:741003-741897(+)∣251	CDD:226450	35.821	67	29	2	185	251	531	583	8.06E-04	37.2	COG3941	COG3941	Mu-like prophage protein
LN02_00220∣LN02Chr01:1178185-1180037(+)∣562	CDD:223903	37.59	556	327	9	12	562	1	541	1.53E-180	517	COG0833	LysP	Amino acid transporters
LN02_00284∣LN02Chr01:1556191-1558003(-)∣559	CDD:225371	24.837	153	97	2	138	272	75	227	8.80E-12	63.8	COG2814	AraJ	Arabinose efflux permease
LN02_00348∣LN02Chr01:1859498-1862250(+)∣824	CDD:225201	32.251	462	274	16	234	680	9	446	2.21E-52	187	COG2319	COG2319	FOG: WD40 repeat
LN02_00412∣LN02Chr01:2057121-2057647(+)∣154	CDD:224726	31.818	66	43	1	82	147	80	143	2.80E-08	47.4	COG1813	COG1813	Predicted transcription factor, homolog of eukaryotic MBF1
LN02_00476∣LN02Chr01:2232139-2233730(+)∣444	CDD:223103	25.076	327	161	9	123	444	8	255	1.48E-59	192	COG0024	Map	Methionine aminopeptidase
LN02_00540∣LN02Chr01:2413619-2418892(+)∣1591	CDD:227623	27.734	768	497	25	381	1107	2	752	4.23E-99	340	COG5307	COG5307	SEC7 domain proteins
LN02_00668∣LN02Chr01:2961452-2964184(-)∣798	CDD:223732	23.96	601	360	9	58	658	1	504	1.10E-104	330	COG0659	SUL1	Sulfate permease and related transporters (MFS superfamily)

LN02_00988丨LN02Chr01:4027784-4029094(+)丨342	CDD:223787	24.172	302	207	9	2	294	27	315	4.32E-48	162	COG0715	TauA	ABC-type nitrate/sulfonate/bicarbonate transport systems, periplasmic components
LN02_01116丨LN02Chr01:4473677-4475960(-)丨505	CDD:223589	33.75	80	49	2	294	372	1	77	9.45E-11	60.5	COG0515	SPS1	Serine/threonine protein kinase
LN02_01244丨LN02Chr01:5020338-5024886(-)丨1452	CDD:227490	21.766	1461	948	43	1	1429	2	1299	3.96E-131	436	COG5161	SFT1	Pre-mRNA cleavage and polyadenylation specificity factor
LN02_01372丨LN02Chr01:5479959-5481929(-)丨598	CDD:224415	30.195	308	197	5	89	395	82	372	2.45E-54	187	COG1498	SIK1	Protein implicated in ribosomal biogenesis, Nop56p homolog
LN02_01564丨LN02Chr01:6270979-6271338(+)丨119	CDD:226180	35.106	94	48	4	7	91	4	93	3.29E-15	63.9	COG3654	Doc	Prophage maintenance system killer protein
LN02_01820丨LN02Chr01:7131198-7133377(-)丨660	CDD:224423	22.552	674	426	21	15	651	3	617	1.65E-61	213	COG1506	DAP2	Dipeptidyl aminopeptidases/acylaminoacyl-peptidases
LN02_01948丨LN02Chr02:77197-78514(-)丨385	CDD:223089	37.048	332	173	12	43	369	5	305	1.71E-74	231	COG0010	SpeB	Arginase/agmatinase/formimionoglutamate hydrolase, arginase family
LN02_02204丨LN02Chr02:925837-926975(-)丨350	CDD:227861	47.273	55	27	2	296	348	216	270	1.36E-15	73	COG5574	PEX10	RING-finger-containing E3 ubiquitin ligase
LN02_02268丨LN02Chr02:1173026-1174817(+)丨453	CDD:227356	56.889	450	183	3	3	452	2	440	0	649	COG5023	COG5023	Tubulin
LN02_02460丨LN02Chr02:1878086-1879696(+)丨461	CDD:223720	20.809	346	165	11	78	417	9	251	1.70E-31	118	COG0647	NagD	Predicted sugar phosphatases of the HAD superfamily
LN02_02524丨LN02Chr02:2101671-2103525(-)丨520	CDD:223884	21.594	389	286	4	37	422	9	381	3.51E-57	193	COG0814	SdaC	Amino acid permeases
LN02_02652丨LN02Chr02:2556372-2563094(+)丨2208	CDD:224125	32.472	813	492	19	490	1292	1	766	0	645	COG1204	COG1204	Superfamily II helicase
LN02_02652丨LN02Chr02:2556372-2563094(+)丨2208	CDD:224125	25.641	624	420	20	1343	1952	1	594	4.00E-67	241	COG1204	COG1204	Superfamily II helicase
LN02_02716丨LN02Chr02:2767334-2769297(+)丨561	CDD:223395	22.581	217	144	7	134	347	41	236	1.23E-07	51.3	COG0318	CaiC	Acyl-CoA synthetases (AMP-forming)/AMP-acid ligases II
LN02_02780丨LN02Chr02:2987748-2989483(-)丨558	CDD:223732	27.171	449	297	6	60	506	2	422	1.76E-79	257	COG0659	SUL1	Sulfate permease and related transporters (MFS superfamily)
LN02_02844丨LN02Chr02:3193297-3195456(+)丨719	CDD:223616	52.958	710	255	8	2	709	145	777	0	897	COG0542	clpA	ATP-binding subunits of Clp protease and DnaK/DnaJ chaperones
LN02_02972丨LN02Chr02:3646119-3648313(+)丨615	CDD:227863	43.103	58	32	1	185	242	52	108	2.44E-17	76.7	COG5576	COG5576	Homeodomain-containing transcription factor
LN02_03036丨LN02Chr02:3885764-3886495(+)丨130	CDD:223174	40.602	133	75	3	1	130	1	132	4.04E-51	155	COG0096	RpsH	Ribosomal protein S8
LN02_03100丨LN02Chr02:4093638-4095997(-)丨493	CDD:223671	14.894	188	132	6	270	447	151	320	1.73E-06	46.9	COG0598	CorA	Mg2+ and Co2+ transporters
LN02_03164丨LN02Chr02:4352916-4354997(-)丨660	CDD:226274	30.488	246	148	5	38	279	25	251	1.78E-48	167	COG3751	EGL-9	Predicted proline hydroxylase

LN02_03292∣LN02Chr02:4763206-4764355(-)∣356	CDD:227721	29.665	209	114	8	101	285	209	408	1.82E-21	92.6	COG5434	PGU1	Endopygalactorunase
LN02_03548∣LN02Chr02:6121112-6122446(+)∣422	CDD:223730	30.233	301	190	7	22	318	12	296	1.47E-33	125	COG0657	Aes	Esterase/lipase
LN02_03612∣LN02Chr02:6667731-6669109(+)∣437	CDD:223727	25.45	389	245	11	12	394	2	351	7.73E-28	110	COG0654	UbiH	2-polyprenyl-6-methoxyphenol hydroxylase and related FAD-dependent oxidoreductases
LN02_03676∣LN02Chr03:273582-275396(-)∣604	CDD:224115	29.841	439	164	14	124	561	12	307	1.88E-83	262	COG1194	MutY	A/G-specific DNA glycosylase
LN02_03804∣LN02Chr03:796486-797928(+)∣480	CDD:226304	27.914	326	182	7	82	405	12	286	3.74E-49	168	COG3781	COG3781	Predicted membrane protein
LN02_04188∣LN02Chr03:3270765-3273396(+)∣512	CDD:227430	20.896	134	95	3	164	286	109	242	7.43E-04	39	COG5099	COG5099	RNA-binding protein of the Puf family, translational repressor
LN02_04316∣LN02Chr03:3763356-3766747(+)∣998	CDD:227381	19.231	260	179	7	1	241	193	440	8.41E-05	43.1	COG5048	COG5048	FOG: Zn-finger
LN02_04380∣LN02Chr03:3980961-3983875(+)∣859	CDD:227554	27.237	760	420	16	112	848	1	650	2.29E-116	366	COG5229	LOC7	Chromosome condensation complex Condensin, subunit H
LN02_04764∣LN02Chr03:5330259-5330877(-)∣163	CDD:223750	43.662	142	68	5	33	163	25	165	2.59E-45	143	COG0678	AHP1	Peroxiredoxin
LN02_04828∣LN02Chr03:5558717-5559243(-)∣99	CDD:226731	39.759	83	43	4	14	94	6	83	3.93E-10	49.1	COG4281	ACB	Acyl-CoA-binding protein
LN02_04892∣LN02Chr03:5887262-5887985(-)∣145	CDD:223600	28.947	114	72	4	32	138	4	115	1.83E-15	65.7	COG0526	TrxA	Thiol-disulfide isomerase and thioredoxins
LN02_05084∣LN02Chr04:560289-561584(-)∣362	CDD:223127	22.013	159	92	4	192	348	20	148	1.04E-10	56.4	COG0049	RpsG	Ribosomal protein S7
LN02_05148∣LN02Chr04:759689-761815(-)∣626	CDD:227270	20.098	612	385	30	40	624	41	575	7.40E-21	94.4	COG4934	COG4934	Predicted protease
LN02_05404∣LN02Chr04:1638774-1640291(-)∣364	CDD:223796	30.909	110	75	1	15	123	84	193	5.13E-11	60	COG0724	COG0724	RNA-binding proteins (RRM domain)
LN02_05404∣LN02Chr04:1638774-1640291(-)∣364	CDD:223796	28.846	156	99	2	194	349	64	207	1.32E-08	52.6	COG0724	COG0724	RNA-binding proteins (RRM domain)
LN02_05788∣LN02Chr04:3195276-3198794(-)∣1172	CDD:223627	26.404	890	557	20	88	910	8	866	4.05E-109	359	COG0553	HepA	Superfamily II DNA/RNA helicases, SNF2 family
LN02_05916∣LN02Chr04:3612008-3613129(+)∣295	CDD:227466	61.491	161	57	1	1	156	1	161	1.39E-69	214	COG5137	COG5137	Histone chaperone involved in gene silencing
LN02_06044∣LN02Chr04:4100383-4101841(+)∣428	CDD:227468	23.786	412	255	9	34	427	26	396	2.74E-46	161	COG5139	COG5139	Uncharacterized conserved protein
LN02_06108∣LN02Chr04:4299286-4303268(+)∣1231	CDD:223589	22.305	269	169	5	398	665	88	317	1.66E-25	107	COG0515	SPS1	Serine/threonine protein kinase
LN02_06172∣LN02Chr04:4556108-4557279(+)∣304	CDD:226016	27.895	190	116	5	111	296	45	217	4.65E-25	97.5	COG3485	PcaH	Protocatechuate 3,4-dioxygenase beta subunit
LN02_06236∣LN02Chr04:4749505-4750482(+)∣294	CDD:224510	42.857	49	28	0	246	294	65	113	1.76E-18	76.3	COG1594	RPB9	DNA-directed RNA polymerase, subunit M/Transcription elongation factor TFIIS

LN02_06300∣LN02Chr04:4954560-4956386(-)∣574	CDD:223685	22.619	504	284	6	24	525	18	417	5.32E-61	205	COG0612	PqqL	Predicted Zn-dependent peptidases
LN02_06364∣LN02Chr04:5156881-5158347(+)∣488	CDD:223548	15.837	221	159	5	160	378	68	263	3.42E-25	102	COG0472	Rfe	UDP-N-acetylmuramyl pentapeptide phosphotransferase/UDP-N-acetylglucosamine-1-phosphate transferase
LN02_06684∣LN02Chr05:400370-401831(+)∣456	CDD:227325	45.854	410	207	5	46	453	1	397	3.07E-157	448	COG4992	ArgD	Ornithine/acetylornithine aminotransferase
LN02_07196∣LN02Chr05:2883096-2883664(+)∣164	CDD:224430	41.135	141	82	1	24	164	6	145	3.48E-41	132	COG1513	CynS	Cyanate lyase
LN02_07452∣LN02Chr05:3824626-3826219(+)∣488	CDD:227422	22.656	256	130	6	238	488	176	368	8.78E-22	94	COG5091	SGT1	Suppressor of G2 allele of skp1 and related proteins
LN02_07516∣LN02Chr05:4039390-4042255(+)∣914	CDD:225201	23.4	500	286	15	225	717	31	440	1.01E-30	124	COG2319	COG2319	FOG: WD40 repeat
LN02_07516∣LN02Chr05:4039390-4042255(+)∣914	CDD:225201	29.804	255	160	5	25	267	203	450	2.40E-20	92.5	COG2319	COG2319	FOG: WD40 repeat
LN02_07580∣LN02Chr05:4291731-4296146(-)∣1411	CDD:226406	21.93	114	77	2	325	438	728	829	3.63E-05	45.2	COG3889	COG3889	Predicted solute binding protein
LN02_07580∣LN02Chr05:4291731-4296146(-)∣1411	CDD:226406	23.353	167	109	7	245	405	694	847	9.22E-05	44.1	COG3889	COG3889	Predicted solute binding protein
LN02_07644∣LN02Chr05:4716863-4718840(-)∣564	CDD:227359	22.581	434	249	17	10	436	24	377	2.60E-28	115	COG5026	COG5026	Hexokinase
LN02_07708∣LN02Chr05:4972977-4974929(+)∣610	CDD:223605	25.767	489	311	8	115	594	9	454	4.20E-41	152	COG0531	PotE	Amino acid transporters
LN02_07772∣LN02Chr05:5194885-5196220(-)∣397	CDD:223796	20	165	123	4	161	318	113	275	1.49E-10	58.8	COG0724	COG0724	RNA-binding proteins (RRM domain)
LN02_07836∣LN02Chr05:5456587-5458424(+)∣500	CDD:226732	37.611	226	94	5	99	324	11	189	9.25E-58	187	COG4282	SMI1	Protein involved in beta-1,3-glucan synthesis
LN02_08028∣LN02Chr06:537515-538646(-)∣314	CDD:223669	17.532	308	198	7	1	281	2	280	5.25E-20	85.1	COG0596	MhpC	Predicted hydrolases or acyltransferases (alpha/beta hydrolase superfamily)
LN02_08092∣LN02Chr06:1220534-1221583(-)∣252	CDD:223489	21.121	232	166	8	28	250	11	234	7.10E-28	103	COG0412	COG0412	Dienelactone hydrolase and related enzymes
LN02_08220∣LN02Chr06:1808914-1810227(+)∣400	CDD:223796	19.008	242	178	3	57	290	3	234	2.61E-10	58	COG0724	COG0724	RNA-binding proteins (RRM domain)
LN02_08220∣LN02Chr06:1808914-1810227(+)∣400	CDD:223880	27.083	96	69	1	9	104	40	134	2.35E-05	42.5	COG0810	TonB	Periplasmic protein TonB, links inner and outer membranes
LN02_08348∣LN02Chr06:2187829-2189422(+)∣439	CDD:223715	17.083	240	155	7	200	437	139	336	1.23E-09	56.3	COG0642	BaeS	Signal transduction histidine kinase
LN02_08540∣LN02Chr06:2909758-2911041(+)∣370	CDD:223725	48.98	147	59	4	17	160	3	136	8.03E-62	192	COG0652	PpiB	Peptidyl-prolyl cis-trans isomerase (rotamase) - cyclophilin family
LN02_08540∣LN02Chr06:2909758-2911041(+)∣370	CDD:223533	22.963	135	92	3	206	340	78	200	2.31E-05	42.5	COG0457	NrfG	FOG: TPR repeat
LN02_08668∣LN02Chr06:3360288-3360613(+)∣69	CDD:227887	25.397	63	46	1	1	62	125	187	6.86E-04	32.8	COG5600	COG5600	Transcription-associated recombination protein

LN02_08860 | LN02Chr07 :619603-620955(+) | 450	CDD:2241 94	20.166	362	252	6	88	447	3	329	3.68E-41	146	COG12 75	TehA	Tellurite resistance protein and related permeases
LN02_08988 | LN02Chr07 :1044198-1045384(-) | 375	CDD:2272 23	29.455	275	184	8	103	370	107	378	3.16E-17	79.6	COG48 86	COG488 6	Leucine-rich repeat (LRR) protein
LN02_09052 | LN02Chr07 :1267050-1268861(+) | 511	CDD:2274 73	35.614	497	254	9	13	507	12	444	7.77E-121	359	COG51 44	TFB2	RNA polymerase II transcription initiation/nucleotide excision repair factor TFIIH, subunit TFB2
LN02_09116 | LN02Chr07 :1466939-1468160(+) | 352	CDD:2235 46	19.307	202	145	4	155	350	41	230	5.66E-10	55.8	COG04 70	HolB	ATPase involved in DNA replication
LN02_09180 | LN02Chr07 :1656813-1658759(-) | 409	CDD:2241 94	20.796	226	142	3	151	376	106	294	5.17E-12	63.5	COG12 75	TehA	Tellurite resistance protein and related permeases
LN02_09244 | LN02Chr07 :1925945-1926837(-) | 135	CDD:2250 74	27.559	127	89	2	1	127	2	125	2.73E-28	97.9	COG21 63	RPL14A	Ribosomal protein L14E/L6E/L27E
LN02_00157 | LN02Chr01 :744612-745456(+) | 100	CDD:2234 78	69.231	52	15	1	50	100	5	56	2.33E-13	56.5	COG04 01	COG040 1	Uncharacterized homolog of Blt101
LN02_00349 | LN02Chr01 :1863025-1863393(+) | 122	CDD:2251 27	38.462	65	17	2	58	118	577	622	1.35E-09	51.4	COG22 17	ZntA	Cation transport ATPase
LN02_00669 | LN02Chr01 :2969468-2970817(+) | 449	CDD:2276 25	25.581	258	156	11	19	272	42	267	5.32E-23	96.1	COG53 09	COG530 9	Exo-beta-1,3-glucanase
LN02_00861 | LN02Chr01 :3616443-3619155(-) | 837	CDD:2235 40	36.879	282	148	8	298	579	234	485	2.40E-61	213	COG04 64	SpoVK	ATPases of the AAA+ class
LN02_00861 | LN02Chr01 :3616443-3619155(-) | 837	CDD:2241 44	28.571	84	52	4	620	698	142	222	4.01E-07	50.2	COG12 23	COG122 3	Predicted ATPase (AAA+ superfamily)
LN02_01053 | LN02Chr01 :4237088-4240619(+) | 1068	CDD:2236 06	32.81	573	306	13	471	1036	1	501	2.72E-155	468	COG05 32	InfB	Translation initiation factor 2 (IF-2; GTPase)
LN02_01309 | LN02Chr01 :5218429-5223830(-) | 1669	CDD:2232 65	27.18	688	430	22	112	781	1	635	5.79E-148	466	COG01 87	GyrB	Type IIA topoisomerase (DNA gyrase/topo II, topoisomerase IV), B subunit
LN02_01309 | LN02Chr01 :5218429-5223830(-) | 1669	CDD:2232 66	23.301	515	331	19	774	1246	4	496	1.42E-80	281	COG01 88	GyrA	Type IIA topoisomerase (DNA gyrase/topo II, topoisomerase IV), A subunit
LN02_01373 | LN02Chr01 :5482284-5484302(+) | 672	CDD:2275 18	26.415	106	75	2	50	152	83	188	1.68E-08	54.2	COG51 91	COG519 1	Uncharacterized conserved protein, contains HAT (Half-A-TPR) repeat
LN02_01373 | LN02Chr01 :5482284-5484302(+) | 672	CDD:2274 38	18.496	492	309	16	63	537	117	533	2.40E-04	41.2	COG51 07	RNA14	Pre-mRNA 3'-end processing (cleavage and polyadenylation) factor
LN02_01437 | LN02Chr01 :5771904-5775657(-) | 924	CDD:2235 50	29.279	888	551	18	77	889	30	915	0	622	COG04 74	MgtA	Cation transport ATPase
LN02_01501 | LN02Chr01 :6007804-6008436(+) | 118	CDD:2260 05	56.436	101	41	2	20	118	33	132	1.64E-53	161	COG34 74	COG347 4	Cytochrome c2
LN02_01757 | LN02Chr01 :6939921-6941434(-) | 464	CDD:2244 05	30.115	435	228	14	17	445	15	379	5.73E-85	264	COG14 88	PncB	Nicotinic acid phosphoribosyltransferase
LN02_01821 | LN02Chr01 :7134535-7137068(-) | 556	CDD:2235 89	26.667	300	135	10	135	371	2	279	2.21E-30	119	COG05 15	SPS1	Serine/threonine protein kinase

LN02_01885∣LN02Chr01:7316217-7316991(-)∣206	CDD:227462	42.718	206	89	4	4	206	2	181	7.82E-58	177	COG5133	COG5133	Uncharacterized conserved protein
LN02_01949∣LN02Chr02:80609-82290(-)∣537	CDD:224594	20.823	389	244	12	17	386	42	385	3.04E-23	98.9	COG1680	AmpC	Beta-lactamase class C and other penicillin binding proteins
LN02_02077∣LN02Chr02:542370-544105(+)∣379	CDD:227352	45.33	364	189	5	10	367	13	372	1.37E-142	407	COG5019	CDC3	Septin family protein
LN02_02205∣LN02Chr02:927802-929102(+)∣405	CDD:227696	24.601	313	200	8	73	382	89	368	2.71E-35	131	COG5409	COG5409	EXS domain-containing protein
LN02_02269∣LN02Chr02:1175322-1177217(-)∣466	CDD:223796	26.496	117	60	2	234	350	104	194	5.44E-06	44.9	COG0724	COG0724	RNA-binding proteins (RRM domain)
LN02_02461∣LN02Chr02:1896660-1899230(+)∣745	CDD:223584	20	305	155	12	272	574	2	219	1.38E-21	92.5	COG0510	ycfN	Thiamine kinase and related kinases
LN02_02781∣LN02Chr02:2990394-2992056(+)∣443	CDD:227535	26.74	273	153	5	162	428	225	456	8.25E-38	141	COG5210	COG5210	GTPase-activating protein
LN02_02845∣LN02Chr02:3196795-3197689(-)∣183	CDD:224025	29.861	144	93	3	15	153	3	143	1.44E-33	116	COG1100	COG1100	GTPase SAR1 and related small G proteins
LN02_02973∣LN02Chr02:3650191-3651747(-)∣481	CDD:223727	22.933	375	248	11	1	366	1	343	1.90E-27	110	COG0654	UbiH	2-polyprenyl-6-methoxyphenol hydroxylase and related FAD-dependent oxidoreductases
LN02_03101∣LN02Chr02:4097140-4099095(-)∣568	CDD:223589	38.284	303	155	8	255	528	1	300	1.78E-60	202	COG0515	SPS1	Serine/threonine protein kinase
LN02_03229∣LN02Chr02:4567370-4571132(+)∣1155	CDD:224117	16.976	754	483	19	444	1139	279	947	1.17E-16	82.5	COG1196	Smc	Chromosome segregation ATPases
LN02_03293∣LN02Chr02:4767509-4768822(+)∣363	CDD:225344	20.056	354	225	15	7	313	49	391	9.48E-24	98.3	COG2730	BglC	Endoglucanase
LN02_03357∣LN02Chr02:4973330-4976197(-)∣906	CDD:225201	24.145	497	322	12	137	624	10	460	1.48E-47	173	COG2319	COG2319	FOG: WD40 repeat
LN02_03421∣LN02Chr02:5153253-5154600(-)∣423	CDD:224223	35.054	368	205	9	44	398	2	348	3.00E-95	287	COG1304	idi	Isopentenyl diphosphate isomerase (BS_ypgA, MTH48 and related proteins)
LN02_03485∣LN02Chr02:5751435-5753499(+)∣630	CDD:223395	25.224	559	362	18	47	592	7	522	8.54E-85	272	COG0318	CaiC	Acyl-CoA synthetases (AMP-forming)/AMP-acid ligases II
LN02_03613∣LN02Chr02:6670116-6671008(+)∣176	CDD:223959	31.447	159	104	3	11	164	2	160	8.45E-39	130	COG1028	FabG	Dehydrogenases with different specificities (related to short-chain alcohol dehydrogenases)
LN02_03677∣LN02Chr03:277260-279199(+)∣527	CDD:225371	22.059	136	104	1	39	174	11	144	1.35E-05	44.5	COG2814	AraJ	Arabinose efflux permease
LN02_03677∣LN02Chr03:277260-279199(+)∣527	CDD:223553	16.399	311	240	4	39	346	2	295	1.44E-04	41.2	COG0477	ProP	Permeases of the major facilitator superfamily
LN02_03805∣LN02Chr03:798282-799577(-)∣431	CDD:225646	31.034	116	71	4	38	144	65	180	3.95E-04	39.6	COG3104	PTR2	Dipeptide/tripeptide permease
LN02_03933∣LN02Chr03:1175668-1176372(-)∣234	CDD:223894	24.194	62	46	1	172	233	56	116	1.71E-09	51.9	COG0824	FcbC	Predicted thioesterase

LN02_03933	LN02Chr03:1175668-1176372(-)	234	CDD:224961	29.412	102	56	5	120	217	34	123	1.38E-04	37.7	COG2050	PaaI	HGG motif-containing thioesterase, possibly involved in aromatic compounds catabolism
LN02_03997	LN02Chr03:1369480-1373429(+)	1208	CDD:223589	29.11	292	159	10	945	1203	1	277	2.04E-44	162	COG0515	SPS1	Serine/threonine protein kinase
LN02_04253	LN02Chr03:3572234-3574147(+)	637	CDD:225633	25.714	105	63	7	446	543	58	154	3.24E-07	47.3	COG3091	SprT	Zn-dependent metalloprotease, SprT family
LN02_04317	LN02Chr03:3767334-3768831(+)	413	CDD:223669	26.027	146	98	3	87	230	14	151	1.05E-07	50	COG0596	MhpC	Predicted hydrolases or acyltransferases (alpha/beta hydrolase superfamily)
LN02_04381	LN02Chr03:3985333-3987465(-)	533	CDD:227935	38.71	93	55	1	101	191	56	148	3.07E-16	74.9	COG5648	NHP6B	Chromatin-associated proteins containing the HMG domain
LN02_04445	LN02Chr03:4191393-4193339(+)	440	CDD:223232	30.316	475	287	13	6	440	2	472	1.83E-79	251	COG0154	GatA	Asp-tRNAAsn/Glu-tRNAGln amidotransferase A subunit and related amidases
LN02_04637	LN02Chr03:4875099-4876434(+)	411	CDD:227486	29.777	403	228	10	11	403	5	362	4.34E-68	217	COG5157	CDC73	RNA polymerase II assessory factor
LN02_05341	LN02Chr04:1438107-1439373(+)	388	CDD:223736	38.182	110	65	1	254	360	1	110	6.57E-21	87.2	COG0664	Crp	cAMP-binding proteins - catabolite gene activator and regulatory subunit of cAMP-dependent protein kinases
LN02_05341	LN02Chr04:1438107-1439373(+)	388	CDD:223736	22.628	137	97	3	122	258	1	128	1.08E-16	75.3	COG0664	Crp	cAMP-binding proteins - catabolite gene activator and regulatory subunit of cAMP-dependent protein kinases
LN02_05469	LN02Chr04:1944548-1946038(+)	372	CDD:224012	40.51	353	183	8	5	355	2	329	5.79E-163	456	COG1087	GalE	UDP-glucose 4-epimerase
LN02_05789	LN02Chr04:3201155-3204316(+)	1034	CDD:225201	21.554	399	235	14	1	389	47	377	9.54E-29	118	COG2319	COG2319	FOG: WD40 repeat
LN02_05917	LN02Chr04:3614055-3616754(-)	779	CDD:227586	33.95	757	443	16	72	777	303	1053	5.43E-94	313	COG5261	IQG1	Protein involved in regulation of cellular morphogenesis/cytokinesis
LN02_06109	LN02Chr04:4304738-4306395(-)	471	CDD:223515	24.122	427	269	9	6	429	5	379	5.31E-34	128	COG0438	RfaG	Glycosyltransferase
LN02_06237	LN02Chr04:4750976-4752367(-)	463	CDD:223110	43.465	329	154	6	96	424	4	300	3.91E-107	317	COG0031	CysK	Cysteine synthase
LN02_06301	LN02Chr04:4957348-4959339(-)	589	CDD:224630	19.58	143	101	4	218	357	53	184	3.38E-15	71.5	COG1716	COG1716	FOG: FHA domain
LN02_06365	LN02Chr04:5159533-5162268(-)	911	CDD:225491	26	100	71	3	323	422	358	454	4.77E-06	47.1	COG2940	COG2940	Proteins containing SET domain
LN02_06621	LN02Chr05:133801-134329(+)	133	CDD:224869	45.588	68	37	0	50	117	12	79	2.45E-18	71.2	COG1958	LSM1	Small nuclear ribonucleoprotein (snRNP) homolog
LN02_06749	LN02Chr05:684252-687998(-)	1147	CDD:223657	21.701	341	171	9	801	1136	6	255	7.32E-32	122	COG0584	UgpQ	Glycerophosphoryl diester phosphodiesterase
LN02_06749	LN02Chr05:684252-687998(-)	1147	CDD:227369	34.586	133	80	3	1	133	1	126	9.54E-17	81.8	COG5036	COG5036	SPX domain-containing protein involved in vacuolar polyphosphate accumulation

LN02_06749 | LN02Chr05 :684252-687998(-) | 1147	CDD:2237 38	28.571	140	91	2	454	587	73	209	7.02E-14	69.5	COG06 66	Arp	FOG: Ankyrin repeat
LN02_06813 | LN02Chr05 :886356-888039(+) | 499	CDD:2230 85	29.138	429	242	17	80	495	5	384	3.18E-68	221	COG00 06	PepP	Xaa-Pro aminopeptidase
LN02_07069 | LN02Chr05 :2455323-2456441(-) | 282	CDD:2252 01	31.76	233	145	8	7	225	127	359	9.24E-36	130	COG23 19	COG231 9	FOG: WD40 repeat
LN02_07133 | LN02Chr05 :2675798-2686254(+) | 2444	CDD:2273 65	24.133	692	425	16	1774	2423	1465	2098	8.80E-78	285	COG50 32	TEL1	Phosphatidylinositol kinase and protein kinases of the PI-3 kinase family
LN02_07197 | LN02Chr05 :2884363-2887751(+) | 885	CDD:2233 15	31.746	189	92	8	16	192	33	196	4.69E-25	100	COG02 37	CoaE	Dephospho-CoA kinase
LN02_07261 | LN02Chr05 :3130060-3131922(-) | 564	CDD:2235 33	23.348	227	162	4	312	532	62	282	2.42E-06	46.4	COG04 57	NrfG	FOG: TPR repeat
LN02_07453 | LN02Chr05 :3826611-3828251(-) | 546	CDD:2250 86	19.697	198	140	4	317	499	93	286	1.61E-15	74.3	COG21 75	TauD	Probable taurine catabolism dioxygenase
LN02_07517 | LN02Chr05 :4042796-4044553(+) | 585	CDD:2278 47	21.311	122	81	3	445	566	716	822	7.13E-05	42.6	COG55 60	UBP12	Ubiquitin C-terminal hydrolase
LN02_07709 | LN02Chr05 :4976137-4978453(-) | 390	CDD:2239 80	36.755	302	180	7	67	366	23	315	6.03E-99	294	COG10 52	LdhA	Lactate dehydrogenase and related dehydrogenases
LN02_07773 | LN02Chr05 :5196565-5198202(+) | 371	CDD:2240 85	52.055	365	172	3	1	365	1	362	1.71E-167	469	COG11 63	DRG	Predicted GTPase
LN02_07965 | LN02Chr05 :5818485-5820096(+) | 508	CDD:2235 53	17.895	285	233	1	45	328	4	288	1.73E-07	50.1	COG04 77	ProP	Permeases of the major facilitator superfamily
LN02_08029 | LN02Chr06 :541274-542420(+) | 299	CDD:2260 16	28.402	169	98	6	109	271	45	196	4.01E-29	108	COG34 85	PcaH	Protocatechuate 3,4-dioxygenase beta subunit
LN02_08221 | LN02Chr06 :1811528-1813528(+) | 550	CDD:2241 17	23.695	249	181	3	87	326	654	902	3.69E-15	75.9	COG11 96	Smc	Chromosome segregation ATPases
LN02_08477 | LN02Chr06 :2741015-2742683(-) | 485	CDD:2235 87	34.951	412	231	9	64	460	27	416	7.79E-108	327	COG05 13	SrmB	Superfamily II DNA and RNA helicases
LN02_08861 | LN02Chr07 :622063-623871(+) | 549	CDD:2236 05	21.51	437	326	5	45	481	17	436	3.81E-32	126	COG05 31	PotE	Amino acid transporters
LN02_08925 | LN02Chr07 :823767-825185(+) | 248	CDD:2274 99	47.442	215	103	3	17	228	3	210	4.02E-61	188	COG51 71	YRB1	Ran GTPase-activating protein (Ran-binding protein)
LN02_09117 | LN02Chr07 :1470545-1473180(+) | 666	CDD:2234 42	42.549	557	287	12	84	639	1	525	0.0	642	COG03 65	Acs	Acyl-coenzyme A synthetases/AMP-(fatty) acid ligases
LN02_00030 | LN02Chr01 :283293-283976(-) | 172	CDD:2237 53	20	165	102	4	9	144	1	164	4.25E-07	44.6	COG06 81	LepB	Signal peptidase I
LN02_00478 | LN02Chr01 :2236680-2238534(-) | 574	CDD:2239 03	43.866	538	276	7	31	567	27	539	0.0	572	COG08 33	LysP	Amino acid transporters
LN02_00670 | LN02Chr01 :2971395-2973261(-) | 588	CDD:2233 95	28.975	566	351	12	25	586	16	534	1.54E-119	361	COG03 18	CaiC	Acyl-CoA synthetases (AMP-forming)/AMP-acid ligases II
LN02_00734 | LN02Chr01 :3201528-3203253(-) | 498	CDD:2237 30	30.625	320	162	10	124	441	51	312	2.98E-40	144	COG06 57	Aes	Esterase/lipase

LN02_00798丨LN02Chr01:3448640-3449704(+)丨354	CDD:227567	31.024	332	186	10	1	328	1	293	5.88E-67	210	COG5242	TFB4	RNA polymerase II transcription initiation/nucleotide excision repair factor TFIIH, subunit TFB4
LN02_00926丨LN02Chr01:3799410-3802346(-)丨978	CDD:227698	25.5	400	175	12	34	432	20	297	6.49E-32	127	COG5411	COG5411	Phosphatidylinositol 5-phosphate phosphatase
LN02_00990丨LN02Chr01:4033945-4035272(+)丨361	CDD:224025	22.798	193	126	7	3	182	6	188	4.87E-17	76.2	COG1100	COG1100	GTPase SAR1 and related small G proteins
LN02_01502丨LN02Chr01:6008905-6011175(-)丨756	CDD:223540	34.32	507	280	11	243	744	30	488	6.53E-100	314	COG0464	SpoVK	ATPases of the AAA+ class
LN02_01630丨LN02Chr01:6525577-6526793(-)丨364	CDD:223991	39.143	350	197	9	17	356	5	348	1.17E-93	281	COG1063	Tdh	Threonine dehydrogenase and related Zn-dependent dehydrogenases
LN02_01758丨LN02Chr01:6942073-6944790(+)丨884	CDD:225201	18.458	428	289	15	150	562	76	458	2.09E-13	70.5	COG2319	COG2319	FOG: WD40 repeat
LN02_02078丨LN02Chr02:545455-546487(-)丨278	CDD:223742	31.481	216	142	3	68	278	18	232	1.83E-35	124	COG0670	COG0670	Integral membrane protein, interacts with FtsH
LN02_02142丨LN02Chr02:723310-724854(-)丨514	CDD:223589	34.783	322	179	9	168	464	1	316	1.41E-55	188	COG0515	SPS1	Serine/threonine protein kinase
LN02_02270丨LN02Chr02:1178055-1180219(+)丨649	CDD:227596	18.545	426	267	20	21	400	3868	4259	4.14E-05	43.8	COG5271	MDN1	AAA ATPase containing von Willebrand factor type A (vWA) domain
LN02_02334丨LN02Chr02:1459747-1460266(+)丨147	CDD:225074	37.405	131	76	4	10	140	1	125	7.81E-16	66.3	COG2163	RPL14A	Ribosomal protein L14E/L6E/L27E
LN02_02398丨LN02Chr02:1697864-1699375(-)丨453	CDD:227827	44.068	59	29	1	259	317	320	374	2.01E-08	53.1	COG5540	COG5540	RING-finger-containing ubiquitin ligase
LN02_02462丨LN02Chr02:1899473-1901398(-)丨596	CDD:223738	28.125	96	64	1	16	106	54	149	7.35E-06	44.8	COG0666	Arp	FOG: Ankyrin repeat
LN02_02782丨LN02Chr02:2992956-2994500(-)丨514	CDD:224191	19.844	257	175	7	248	504	1	226	3.33E-35	128	COG1272	COG1272	Predicted membrane protein, hemolysin III homolog
LN02_03166丨LN02Chr02:4359562-4361021(-)丨399	CDD:223669	19.931	291	207	6	97	377	7	281	4.94E-14	68.9	COG0596	MhpC	Predicted hydrolases or acyltransferases (alpha/beta hydrolase superfamily)
LN02_03230丨LN02Chr02:4572668-4575169(+)丨833	CDD:224709	23.181	371	208	14	484	831	1	317	3.45E-30	119	COG1796	POL4	DNA polymerase IV (family X)
LN02_03294丨LN02Chr02:4769944-4771775(+)丨518	CDD:224230	21.373	510	328	21	24	514	20	475	7.04E-50	175	COG1311	HYS2	Archaeal DNA polymerase II, small subunit/DNA polymerase delta, subunit B
LN02_03358丨LN02Chr02:4976950-4978390(+)丨384	CDD:227352	49.598	373	184	4	9	378	2	373	2.98E-164	462	COG5019	CDC3	Septin family protein
LN02_03486丨LN02Chr02:5757407-5759930(+)丨818	CDD:224666	21.023	352	212	13	217	564	8	297	7.95E-35	132	COG1752	RssA	Predicted esterase of the alpha-beta hydrolase superfamily
LN02_03678丨LN02Chr03:279785-280918(-)丨377	CDD:224829	21.918	73	49	2	75	146	58	123	3.62E-04	37.5	COG1917	COG1917	Uncharacterized conserved protein, contains double-stranded beta-helix domain
LN02_03870丨LN02Chr03:979792-980838(-)丨348	CDD:223669	19.608	306	204	6	34	335	15	282	1.68E-24	98.2	COG0596	MhpC	Predicted hydrolases or acyltransferases (alpha/beta hydrolase superfamily)
LN02_03998丨LN02Chr03:1374316-1377914(-)丨1077	CDD:225201	20.958	334	218	11	7	325	28	330	4.49E-06	47.4	COG2319	COG2319	FOG: WD40 repeat

LN02_04318 | LN02Chr03 :3769321-3774192(-) | 1332	CDD:224055	32.513	569	365	8	763	1331	18	567	2.29E-126	400	COG1132	MdlB	ABC-type multidrug transport system, ATPase and permease components
LN02_04318 | LN02Chr03 :3769321-3774192(-) | 1332	CDD:224055	30.394	635	368	8	62	696	6	566	1.58E-114	368	COG1132	MdlB	ABC-type multidrug transport system, ATPase and permease components
LN02_04446 | LN02Chr03 :4195600-4197541(+) | 601	CDD:223769	20.161	248	171	7	72	318	71	292	3.54E-07	49.1	COG0697	RhaT	Permeases of the drug/metabolite transporter (DMT) superfamily
LN02_04510 | LN02Chr03 :4400056-4402367(-) | 663	CDD:225201	23.145	337	224	13	235	562	49	359	5.03E-19	87.5	COG2319	COG2319	FOG: WD40 repeat
LN02_04574 | LN02Chr03 :4639412-4640870(-) | 464	CDD:227454	23.37	184	100	3	263	446	116	258	3.92E-18	81.2	COG5125	COG5125	Uncharacterized conserved protein
LN02_04702 | LN02Chr03 :5112209-5115233(+) | 925	CDD:227365	20.652	644	410	19	345	905	1456	2081	1.34E-66	241	COG5032	TEL1	Phosphatidylinositol kinase and protein kinases of the PI-3 kinase family
LN02_04830 | LN02Chr03 :5563469-5564454(+) | 257	CDD:224360	37.097	186	100	3	45	230	1	169	3.58E-77	229	COG1443	Idi	Isopentenyldiphosphate isomerase
LN02_04958 | LN02Chr03 :6309715-6310840(-) | 339	CDD:225714	27.174	92	52	5	209	289	202	289	1.13E-04	40.5	COG3173	COG3173	Predicted aminoglycoside phosphotransferase
LN02_05214 | LN02Chr04 :1000563-1004573(-) | 1261	CDD:223163	29.037	1257	654	34	21	1251	10	1054	0	1067	COG0085	RpoB	DNA-directed RNA polymerase, beta subunit/140 kD subunit
LN02_05278 | LN02Chr04 :1193858-1194394(+) | 178	CDD:224167	23.077	143	87	6	9	150	4	124	6.80E-07	43.8	COG1246	ArgA	N-acetylglutamate synthase and related acetyltransferases
LN02_05406 | LN02Chr04 :1643375-1645577(+) | 633	CDD:223446	29.272	632	350	17	1	631	43	578	2.67E-115	354	COG0369	CysJ	Sulfite reductase, alpha subunit (flavoprotein)
LN02_05470 | LN02Chr04 :1946273-1947337(-) | 329	CDD:223677	41.337	329	180	8	8	327	1	325	6.49E-90	269	COG0604	Qor	NADPH:quinone reductase and related Zn-dependent oxidoreductases
LN02_05662 | LN02Chr04 :2779886-2781353(-) | 430	CDD:223540	42.238	277	150	6	100	367	213	488	1.16E-87	272	COG0464	SpoVK	ATPases of the AAA+ class
LN02_05726 | LN02Chr04 :3008758-3010117(+) | 395	CDD:225862	33.838	396	211	10	4	353	36	426	3.45E-104	312	COG3325	ChiA	Chitinase
LN02_05790 | LN02Chr04 :3205708-3206424(+) | 216	CDD:227353	26.519	181	107	4	1	172	101	264	2.50E-27	104	COG5020	KTR1	Mannosyltransferase
LN02_05854 | LN02Chr04 :3413983-3415748(-) | 533	CDD:227359	25.549	501	313	12	35	525	5	455	5.64E-54	186	COG5026	COG5026	Hexokinase
LN02_05982 | LN02Chr04 :3904950-3907035(-) | 585	CDD:225661	30.909	495	283	14	21	482	3	471	1.56E-88	279	COG3119	AslA	Arylsulfatase A and related enzymes
LN02_06046 | LN02Chr04 :4104187-4108549(+) | 585	CDD:223589	32.258	155	84	7	119	267	1	140	1.97E-22	96.7	COG0515	SPS1	Serine/threonine protein kinase
LN02_06110 | LN02Chr04 :4307086-4308094(+) | 275	CDD:223955	25.806	248	156	9	25	263	10	238	6.39E-28	105	COG1024	CaiD	Enoyl-CoA hydratase/carnithine racemase
LN02_06174 | LN02Chr04 :4561755-4564367(-) | 253	CDD:223756	24.8	125	84	2	137	252	83	206	1.45E-10	56.5	COG0684	MenG	Demethylmenaquinone methyltransferase
LN02_06238 | LN02Chr04 :4752593-4754371(+) | 549	CDD:224481	27.632	152	66	5	142	292	21	129	7.86E-12	63.9	COG1565	COG1565	Uncharacterized conserved protein
LN02_06302 | LN02Chr04 :4961674-4969635(-) | 2454	CDD:227365	30.989	526	291	12	1949	2454	1632	2105	4.41E-93	335	COG5032	TEL1	Phosphatidylinositol kinase and protein kinases of the PI-3 kinase family

LN02_06686∣LN02Chr05:402512-404503(-)∣527	CDD:223154	35.252	417	250	8	55	466	33	434	2.37E-108	328	COG0076	GadB	Glutamate decarboxylase and related PLP-dependent proteins
LN02_06942∣LN02Chr05:1699125-1702886(+)∣1186	CDD:224117	24.077	1192	822	16	1	1186	41	1155	4.93E-143	459	COG1196	Smc	Chromosome segregation ATPases
LN02_07070∣LN02Chr05:2456579-2457773(-)∣343	CDD:225201	29.333	75	49	2	273	343	242	316	2.69E-06	45.8	COG2319	COG2319	FOG: WD40 repeat
LN02_07134∣LN02Chr05:2686459-2688911(+)∣766	CDD:227934	24.682	786	466	23	48	766	47	773	9.30E-104	333	COG5647	COG5647	Cullin, a subunit of E3 ubiquitin ligase
LN02_07198∣LN02Chr05:2888477-2890217(-)∣444	CDD:225362	35.616	73	41	3	222	289	51	122	3.22E-09	54	COG2802	COG2802	Uncharacterized protein, similar to the N-terminal domain of Lon protease
LN02_07198∣LN02Chr05:2888477-2890217(-)∣444	CDD:227861	33.333	60	35	2	110	167	203	259	1.61E-07	49.5	COG5574	PEX10	RING-finger-containing E3 ubiquitin ligase
LN02_07262∣LN02Chr05:3132402-3133226(+)∣229	CDD:223209	44.954	218	94	3	10	227	4	195	3.60E-96	276	COG0131	HisB	Imidazoleglycerol-phosphate dehydratase
LN02_07326∣LN02Chr05:3348873-3349751(+)∣236	CDD:227387	47.748	111	57	1	88	198	72	181	2.20E-49	157	COG5054	ERV1	Mitochondrial sulfhydryl oxidase involved in the biogenesis of cytosolic Fe/S proteins
LN02_07454∣LN02Chr05:3828889-3830564(-)∣450	CDD:224590	26.036	169	81	8	255	412	30	165	4.05E-16	73.2	COG1676	SEN2	tRNA splicing endonuclease
LN02_07518∣LN02Chr05:4045311-4046557(-)∣345	CDD:227386	34.01	197	110	5	132	311	12	205	1.72E-42	144	COG5053	CDC33	Translation initiation factor 4E (eIF-4E)
LN02_07646∣LN02Chr05:4721152-4722418(-)∣322	CDD:227407	35.312	320	188	7	1	319	1	302	2.48E-78	238	COG5075	COG5075	Uncharacterized conserved protein
LN02_07966∣LN02Chr05:5820341-5821349(-)∣286	CDD:223859	54.007	287	131	1	1	286	1	287	2.71E-154	429	COG0788	PurU	Formyltetrahydrofolate hydrolase
LN02_08286∣LN02Chr06:1986895-1987679(-)∣202	CDD:223281	45.614	114	60	2	6	119	5	116	2.26E-36	120	COG0203	RplQ	Ribosomal protein L17
LN02_08350∣LN02Chr06:2194180-2196385(-)∣564	CDD:223738	27.607	163	94	2	243	381	40	202	3.40E-13	66.4	COG0666	Arp	FOG: Ankyrin repeat
LN02_08350∣LN02Chr06:2194180-2196385(-)∣564	CDD:223738	23.913	138	97	4	164	299	72	203	0.001	38.3	COG0666	Arp	FOG: Ankyrin repeat
LN02_08414∣LN02Chr06:2503525-2505958(+)∣785	CDD:227634	36.414	725	401	15	50	753	44	729	0	570	COG5324	COG5324	Uncharacterized conserved protein
LN02_08862∣LN02Chr07:624654-626261(-)∣494	CDD:225201	29.032	186	113	6	251	435	146	313	2.05E-22	97.1	COG2319	COG2319	FOG: WD40 repeat
LN02_08926∣LN02Chr07:827580-828881(+)∣240	CDD:227366	30.493	223	140	4	14	230	6	219	1.41E-37	128	COG5033	TFG3	Transcription initiation factor IIF, auxiliary subunit
LN02_08990∣LN02Chr07:1054125-1054802(+)∣225	CDD:224584	27.551	196	116	7	28	218	3	177	3.37E-18	76.5	COG1670	RimL	Acetyltransferases, including N-acetylases of ribosomal proteins
LN02_09182∣LN02Chr07:1663341-1666349(+)∣928	CDD:224117	20.95	358	250	3	493	850	690	1014	1.22E-16	82.1	COG1196	Smc	Chromosome segregation ATPases
LN02_09310∣LN02Chr07:2137329-2140199(+)∣843	CDD:226406	21.739	115	85	2	222	334	751	862	3.17E-05	44.5	COG3889	COG3889	Predicted solute binding protein

LN02_00260∣LN02Chr01:1487439-1488657(-)∣331	CDD:227538	43.056	72	36	2	136	207	105	171	2.27E-13	66.2	COG5213	FIP1	Polyadenylation factor I complex, subunit FIP1
LN02_00388∣LN02Chr01:1982627-1983370(+)∣121	CDD:227599	40.984	61	32	2	13	71	44	102	3.65E-10	51.7	COG5274	CYB5	Cytochrome b involved in lipid metabolism
LN02_00452∣LN02Chr01:2157929-2162214(-)∣1346	CDD:223224	50.276	543	229	9	775	1306	1	513	0	641	COG0146	HyuB	N-methylhydantoinase B/acetone carboxylase, alpha subunit
LN02_00452∣LN02Chr01:2157929-2162214(-)∣1346	CDD:223223	33.95	757	402	20	9	755	2	670	0	548	COG0145	HyuA	N-methylhydantoinase A/acetone carboxylase, beta subunit
LN02_00580∣LN02Chr01:2607343-2608162(-)∣231	CDD:223186	49.767	215	93	3	10	223	2	202	6.64E-88	255	COG0108	RibB	3,4-dihydroxy-2-butanone 4-phosphate synthase
LN02_00900∣LN02Chr01:3718240-3719227(+)∣234	CDD:225145	28.641	206	117	7	31	226	1	186	1.38E-46	150	COG2236	COG2236	Predicted phosphoribosyltransferases
LN02_00964∣LN02Chr01:3946005-3947480(-)∣491	CDD:223139	24.855	346	165	9	93	437	1	252	1.27E-51	174	COG0061	nadF	NAD kinase
LN02_01092∣LN02Chr01:4400677-4401244(-)∣141	CDD:223478	47.273	55	29	0	1	55	1	55	5.08E-19	72.3	COG0401	COG0401	Uncharacterized homolog of Blt101
LN02_01156∣LN02Chr01:4640766-4647234(+)∣1027	CDD:226406	19.872	156	86	6	320	455	694	830	0.001	39.8	COG3889	COG3889	Predicted solute binding protein
LN02_01284∣LN02Chr01:5148614-5150795(-)∣652	CDD:223201	30.523	344	220	7	20	353	2	336	3.63E-118	353	COG0123	AcuC	Deacetylases, including yeast histone deacetylase and acetoin utilization protein
LN02_01284∣LN02Chr01:5148614-5150795(-)∣652	CDD:227596	21.293	263	178	11	404	642	3881	4138	4.67E-06	46.9	COG5271	MDN1	AAA ATPase containing von Willebrand factor type A (vWA) domain
LN02_01348∣LN02Chr01:5387677-5391291(+)∣1160	CDD:223627	24.962	665	300	15	509	1145	360	853	5.25E-62	225	COG0553	HepA	Superfamily II DNA/RNA helicases, SNF2 family
LN02_01668∣LN02Chr01:6691734-6694139(-)∣768	CDD:226947	47.826	46	24	0	445	490	476	521	4.95E-08	53.5	COG4581	COG4581	Superfamily II RNA helicase
LN02_01668∣LN02Chr01:6691734-6694139(-)∣768	CDD:224125	21.159	345	181	13	230	490	55	392	5.15E-08	53.5	COG1204	COG1204	Superfamily II helicase
LN02_01732∣LN02Chr01:6877339-6880582(-)∣1042	CDD:227944	21.332	736	481	26	22	736	9	667	2.29E-52	196	COG5657	CSE1	CAS/CSE protein involved in chromosome segregation
LN02_01796∣LN02Chr01:7051807-7054351(-)∣728	CDD:226582	19.624	479	271	21	254	703	24	417	3.21E-09	56.7	COG4097	COG4097	Predicted ferric reductase
LN02_01924∣LN02Chr01:7423261-7424137(+)∣270	CDD:223761	28.509	228	146	4	13	237	8	221	4.31E-43	143	COG0689	Rph	RNase PH
LN02_02180∣LN02Chr02:832110-833195(+)∣202	CDD:224947	36.25	80	49	1	45	124	14	91	1.00E-28	100	COG2036	HHT1	Histones H3 and H4
LN02_02308∣LN02Chr02:1310889-1312052(-)∣387	CDD:223260	44.598	361	170	6	12	372	12	342	1.64E-143	408	COG0182	COG0182	Predicted translation initiation factor 2B subunit, eIF-2B alpha/beta/delta family
LN02_02500∣LN02Chr02:2036410-2037324(+)∣304	CDD:223253	35.849	265	154	9	39	297	5	259	1.97E-68	211	COG0175	CysH	3'-phosphoadenosine 5'-phosphosulfate sulfotransferase (PAPS reductase)/FAD synthetase and related enzymes

LN02_02564 | LN02Chr02 :2252557-2254289(-) | 520	CDD:227394	26.087	529	277	16	6	520	1	429	6.95E-62	206	COG5062	COG5062	Uncharacterized membrane protein
LN02_02756 | LN02Chr02 :2862952-2864271(+) | 439	CDD:227390	38.36	378	197	8	65	439	9	353	7.36E-110	325	COG5057	LAG1	Phosphotyrosyl phosphatase activator
LN02_03268 | LN02Chr02 :4688927-4693569(+) | 1290	CDD:227432	25.325	154	104	5	33	179	18	167	3.29E-09	58.4	COG5101	CRM1	Importin beta-related nuclear transport receptor
LN02_03332 | LN02Chr02 :4897607-4899976(+) | 665	CDD:227357	46.831	284	148	2	341	623	145	426	1.36E-110	337	COG5024	COG5024	Cyclin
LN02_03396 | LN02Chr02 :5083531-5089468(+) | 1136	CDD:223601	31.954	435	248	11	709	1132	48	445	4.51E-92	299	COG0527	LysC	Aspartokinases
LN02_03652 | LN02Chr03 :134520-136206(-) | 462	CDD:223085	25.472	424	243	12	46	456	21	384	2.26E-58	194	COG0006	PepP	Xaa-Pro aminopeptidase
LN02_03844 | LN02Chr03 :916332-918157(-) | 513	CDD:227389	33.878	245	150	4	255	492	270	509	8.15E-47	167	COG5056	ARE1	Acyl-CoA cholesterol acyltransferase
LN02_03972 | LN02Chr03 :1290832-1291944(-) | 320	CDD:226413	25.581	129	62	6	47	164	21	126	1.46E-09	54.4	COG3897	COG3897	Predicted methyltransferase
LN02_04164 | LN02Chr03 :2923879-2925618(+) | 419	CDD:223103	25.882	255	151	10	12	265	4	221	3.54E-18	80.7	COG0024	Map	Methionine aminopeptidase
LN02_04356 | LN02Chr03 :3895144-3896877(-) | 487	CDD:227582	49.176	425	178	6	77	479	6	414	0	589	COG5257	GCD11	Translation initiation factor 2, gamma subunit (eIF-2gamma; GTPase)
LN02_04676 | LN02Chr03 :5032751-5037581(+) | 1202	CDD:224983	27.004	237	157	7	1	233	9	233	2.12E-25	107	COG2072	TrkA	Predicted flavoprotein involved in K+ transport
LN02_04740 | LN02Chr03 :5230280-5235124(-) | 1537	CDD:223550	23.674	1018	496	29	362	1376	149	888	1.41E-162	513	COG0474	MgtA	Cation transport ATPase
LN02_04804 | LN02Chr03 :5459544-5465480(+) | 1259	CDD:227881	31.674	442	277	8	600	1039	304	722	2.82E-87	299	COG5594	COG5594	Uncharacterized integral membrane protein
LN02_04804 | LN02Chr03 :5459544-5465480(+) | 1259	CDD:227881	29.699	266	158	6	91	341	38	289	1.19E-32	134	COG5594	COG5594	Uncharacterized integral membrane protein
LN02_04996 | LN02Chr04 :302531-302917(+) | 128	CDD:225226	37.681	138	58	4	1	128	5	124	5.79E-32	107	COG2351	COG2351	Transthyretin-like protein
LN02_05124 | LN02Chr04 :689164-690229(+) | 253	CDD:223711	39.044	251	135	7	4	252	1	235	5.95E-67	204	COG0638	PRE1	20S proteasome, alpha and beta subunits
LN02_05188 | LN02Chr04 :901989-903651(-) | 527	CDD:225035	21.247	433	282	13	30	459	5	381	5.32E-20	89.4	COG2124	CypX	Cytochrome P450
LN02_05572 | LN02Chr04 :2490418-2492269(+) | 573	CDD:224140	33.406	461	179	7	80	531	52	393	7.84E-119	354	COG1219	ClpX	ATP-dependent protease Clp, ATPase subunit
LN02_05700 | LN02Chr04 :2919141-2923823(-) | 1512	CDD:224054	31.034	232	143	7	872	1100	4	221	5.49E-46	165	COG1131	CcmA	ABC-type multidrug transport system, ATPase component
LN02_05700 | LN02Chr04 :2919141-2923823(-) | 1512	CDD:224054	21.739	299	201	7	183	480	18	284	3.28E-40	148	COG1131	CcmA	ABC-type multidrug transport system, ATPase component

LN02_05764 | LN02Chr04 :3115260-3116634(-) | 386	CDD:2257 14	23.622	127	78	5	149	274	128	236	6.15E-04	38.2	COG31 73	COG317 3	Predicted aminoglycoside phosphotransferase
LN02_05828 | LN02Chr04 :3336885-3343005(-) | 1966	CDD:2241 17	18.342	736	490	16	1047	1742	238	902	2.77E-08	56.3	COG11 96	Smc	Chromosome segregation ATPases
LN02_05828 | LN02Chr04 :3336885-3343005(-) | 1966	CDD:2234 28	30	90	43	4	669	744	81	164	8.98E-05	43.3	COG03 51	ThiD	Hydroxymethylpyrimidine/phosphomet hylpyrimidine kinase
LN02_05892 | LN02Chr04 :3535386-3536869(+) | 389	CDD:2251 80	17.61	159	121	3	1	150	55	212	2.21E-04	39.9	COG22 71	UhpC	Sugar phosphate permease
LN02_05956 | LN02Chr04 :3818507-3819197(+) | 207	CDD:2279 39	20.339	118	90	3	9	123	32	148	1.18E-05	40.8	COG56 52	COG565 2	Predicted integral membrane protein
LN02_06084 | LN02Chr04 :4204336-4205737(+) | 377	CDD:2237 30	25.573	262	171	8	80	338	40	280	5.47E-13	66.1	COG06 57	Aes	Esterase/lipase
LN02_06148 | LN02Chr04 :4422723-4425053(+) | 665	CDD:2235 20	52.941	612	250	9	39	648	4	579	0	709	COG04 43	DnaK	Molecular chaperone
LN02_06212 | LN02Chr04 :4679697-4681633(+) | 580	CDD:2234 98	28.947	152	93	6	342	483	80	226	7.12E-21	90.1	COG04 21	SpeE	Spermidine synthase
LN02_06660 | LN02Chr05 :286532-289181(-) | 714	CDD:2239 77	19.617	836	474	26	50	712	51	861	8.19E-101	326	COG10 48	AcnA	Aconitase A
LN02_06980 | LN02Chr05 :1852242-1853126(+) | 213	CDD:2237 96	21.212	165	99	2	79	212	111	275	4.78E-12	61.1	COG07 24	COG072 4	RNA-binding proteins (RRM domain)
LN02_07044 | LN02Chr05 :2255366-2258689(-) | 869	CDD:2264 28	25.917	436	242	10	426	858	260	617	1.55E-53	194	COG39 14	Spy	Predicted O-linked N-acetylglucosamine transferase, SPINDLY family
LN02_07044 | LN02Chr05 :2255366-2258689(-) | 869	CDD:2235 33	29.07	86	60	1	130	214	186	271	7.78E-08	51.8	COG04 57	NrfG	FOG: TPR repeat
LN02_07108 | LN02Chr05 :2590128-2592768(-) | 787	CDD:2235 33	20.641	281	202	8	450	721	20	288	6.16E-14	70.3	COG04 57	NrfG	FOG: TPR repeat
LN02_07236 | LN02Chr05 :3051505-3053158(-) | 339	CDD:2239 80	35.714	336	196	7	3	330	1	324	1.71E-91	273	COG10 52	LdhA	Lactate dehydrogenase and related dehydrogenases
LN02_07300 | LN02Chr05 :3261778-3262946(-) | 330	CDD:2262 00	20.53	151	83	7	92	213	82	224	1.40E-04	39.9	COG36 75	COG367 5	Predicted lipase
LN02_07556 | LN02Chr05 :4144972-4145834(-) | 154	CDD:2231 83	56.818	132	57	0	5	136	3	134	5.16E-80	230	COG01 05	Ndk	Nucleoside diphosphate kinase
LN02_07684 | LN02Chr05 :4834394-4836232(+) | 551	CDD:2264 06	21.818	165	94	10	53	202	728	872	5.83E-05	42.9	COG38 89	COG388 9	Predicted solute binding protein
LN02_07812 | LN02Chr05 :5367091-5368778(-) | 514	CDD:2239 55	24.85	334	167	8	61	393	7	257	1.28E-42	149	COG10 24	CaiD	Enoyl-CoA hydratase/carnithine racemase
LN02_07940 | LN02Chr05 :5745093-5745809(-) | 238	CDD:2239 43	25.532	235	167	5	4	236	1	229	1.07E-32	116	COG10 11	COG101 1	Predicted hydrolase (HAD superfamily)
LN02_08132 | LN02Chr06 :1364589-1366324(-) | 515	CDD:2252 01	32.275	378	201	13	145	513	108	439	9.73E-54	185	COG23 19	COG231 9	FOG: WD40 repeat
LN02_08836 | LN02Chr07 :493482-495095(-) | 503	CDD:2234 01	26.744	258	108	5	7	190	1	251	3.38E-38	138	COG03 24	MiaA	tRNA delta(2)-isopentenylpyrophosphate transferase
LN02_08836 | LN02Chr07 :493482-495095(-) | 503	CDD:2245 27	31.746	189	110	7	302	486	17	190	5.54E-28	107	COG16 11	COG161 1	Predicted Rossmann fold nucleotide-binding protein

<table>
<tr><th></th><th></th><th></th><th></th><th></th><th></th><th></th><th></th><th></th><th></th><th></th><th></th><th></th><th></th><th></th></tr>
<tr><td>LN02_09028|LN02Chr07:1185328-1186302(+)|289</td><td>CDD:223796</td><td>21.429</td><td>84</td><td>61</td><td>2</td><td>4</td><td>82</td><td>115</td><td>198</td><td>6.91E-04</td><td>37.6</td><td>COG0724</td><td>COG0724</td><td>RNA-binding proteins (RRM domain)</td></tr>
<tr><td>LN02_09220|LN02Chr07:1784400-1786844(+)|814</td><td>CDD:227500</td><td>19.37</td><td>826</td><td>559</td><td>19</td><td>3</td><td>813</td><td>2</td><td>735</td><td>5.72E-74</td><td>253</td><td>COG5173</td><td>SEC6</td><td>Exocyst complex subunit SEC6</td></tr>
<tr><td>LN02_09284|LN02Chr07:2030836-2031955(-)|238</td><td>CDD:223272</td><td>49.171</td><td>181</td><td>87</td><td>2</td><td>6</td><td>186</td><td>2</td><td>177</td><td>8.44E-84</td><td>245</td><td>COG0194</td><td>Gmk</td><td>Guanylate kinase</td></tr>
<tr><td>LN02_00159|LN02Chr01:750940-752344(+)|387</td><td>CDD:225042</td><td>42.667</td><td>150</td><td>80</td><td>4</td><td>230</td><td>375</td><td>8</td><td>155</td><td>1.76E-54</td><td>174</td><td>COG2131</td><td>ComEB</td><td>Deoxycytidylate deaminase</td></tr>
<tr><td>LN02_00223|LN02Chr01:1193518-1197542(-)|1282</td><td>CDD:223738</td><td>20.276</td><td>217</td><td>151</td><td>5</td><td>691</td><td>902</td><td>31</td><td>230</td><td>7.68E-06</td><td>46</td><td>COG0666</td><td>Arp</td><td>FOG: Ankyrin repeat</td></tr>
<tr><td>LN02_00287|LN02Chr01:1564064-1564840(+)|258</td><td>CDD:223710</td><td>29.648</td><td>199</td><td>118</td><td>7</td><td>1</td><td>196</td><td>9</td><td>188</td><td>7.40E-32</td><td>114</td><td>COG0637</td><td>COG0637</td><td>Predicted phosphatase/phosphohexomutase</td></tr>
<tr><td>LN02_00351|LN02Chr01:1869831-1871694(+)|567</td><td>CDD:225181</td><td>28.625</td><td>538</td><td>297</td><td>21</td><td>36</td><td>553</td><td>10</td><td>480</td><td>5.44E-77</td><td>249</td><td>COG2272</td><td>PnbA</td><td>Carboxylesterase type B</td></tr>
<tr><td>LN02_00415|LN02Chr01:2061014-2061829(+)|106</td><td>CDD:224546</td><td>55.789</td><td>95</td><td>39</td><td>2</td><td>2</td><td>95</td><td>1</td><td>93</td><td>4.03E-29</td><td>97.9</td><td>COG1631</td><td>RPL42A</td><td>Ribosomal protein L44E</td></tr>
<tr><td>LN02_00671|LN02Chr01:2974221-2975960(-)|529</td><td>CDD:225035</td><td>19.076</td><td>498</td><td>309</td><td>12</td><td>29</td><td>520</td><td>1</td><td>410</td><td>9.47E-40</td><td>146</td><td>COG2124</td><td>CypX</td><td>Cytochrome P450</td></tr>
<tr><td>LN02_00735|LN02Chr01:3204181-3205460(-)|342</td><td>CDD:224117</td><td>24.167</td><td>120</td><td>85</td><td>2</td><td>127</td><td>240</td><td>298</td><td>417</td><td>4.40E-06</td><td>45.5</td><td>COG1196</td><td>Smc</td><td>Chromosome segregation ATPases</td></tr>
<tr><td>LN02_00799|LN02Chr01:3450664-3452624(-)|592</td><td>CDD:224363</td><td>34.463</td><td>177</td><td>97</td><td>5</td><td>26</td><td>199</td><td>4</td><td>164</td><td>7.51E-25</td><td>102</td><td>COG1446</td><td>COG1446</td><td>Asparaginase</td></tr>
<tr><td>LN02_00799|LN02Chr01:3450664-3452624(-)|592</td><td>CDD:224363</td><td>24.088</td><td>137</td><td>79</td><td>4</td><td>405</td><td>532</td><td>170</td><td>290</td><td>1.09E-12</td><td>66.2</td><td>COG1446</td><td>COG1446</td><td>Asparaginase</td></tr>
<tr><td>LN02_00991|LN02Chr01:4035336-4036039(-)|185</td><td>CDD:224288</td><td>25</td><td>132</td><td>72</td><td>3</td><td>2</td><td>133</td><td>12</td><td>116</td><td>1.26E-16</td><td>69.7</td><td>COG1369</td><td>POP5</td><td>RNase P/RNase MRP subunit POP5</td></tr>
<tr><td>LN02_01055|LN02Chr01:4245988-4248246(-)|692</td><td>CDD:224136</td><td>19.239</td><td>447</td><td>289</td><td>13</td><td>95</td><td>519</td><td>8</td><td>404</td><td>1.42E-20</td><td>91.9</td><td>COG1215</td><td>COG1215</td><td>Glycosyltransferases, probably involved in cell wall biogenesis</td></tr>
<tr><td>LN02_01439|LN02Chr01:5785133-5788106(-)|718</td><td>CDD:223796</td><td>21.101</td><td>109</td><td>77</td><td>3</td><td>456</td><td>555</td><td>70</td><td>178</td><td>2.14E-06</td><td>47.2</td><td>COG0724</td><td>COG0724</td><td>RNA-binding proteins (RRM domain)</td></tr>
<tr><td>LN02_01567|LN02Chr01:6285722-6287839(-)|679</td><td>CDD:227657</td><td>25.959</td><td>678</td><td>371</td><td>15</td><td>6</td><td>675</td><td>4</td><td>558</td><td>2.28E-126</td><td>383</td><td>COG5354</td><td>COG5354</td><td>Uncharacterized protein, contains Trp-Asp (WD) repeat</td></tr>
<tr><td>LN02_02015|LN02Chr02:327578-331895(+)|1346</td><td>CDD:227585</td><td>31.549</td><td>355</td><td>211</td><td>10</td><td>250</td><td>598</td><td>15</td><td>343</td><td>1.05E-73</td><td>251</td><td>COG5260</td><td>TRF4</td><td>DNA polymerase sigma</td></tr>
<tr><td>LN02_02079|LN02Chr02:549032-550776(+)|469</td><td>CDD:227391</td><td>31.23</td><td>317</td><td>188</td><td>6</td><td>86</td><td>399</td><td>88</td><td>377</td><td>6.06E-65</td><td>212</td><td>COG5058</td><td>LAG1</td><td>Protein transporter of the TRAM (translocating chain-associating membrane) superfamily, longevity assurance factor</td></tr>
<tr><td>LN02_02143|LN02Chr02:726324-727886(+)|520</td><td>CDD:225201</td><td>27.078</td><td>373</td><td>228</td><td>10</td><td>171</td><td>516</td><td>88</td><td>443</td><td>5.01E-41</td><td>151</td><td>COG2319</td><td>COG2319</td><td>FOG: WD40 repeat</td></tr>
<tr><td>LN02_02207|LN02Chr02:935213-936823(+)|407</td><td>CDD:223568</td><td>35.443</td><td>79</td><td>45</td><td>2</td><td>116</td><td>194</td><td>9</td><td>81</td><td>1.17E-07</td><td>48.3</td><td>COG0494</td><td>MutT</td><td>NTP pyrophosphohydrolases including oxidative damage repair enzymes</td></tr>
<tr><td>LN02_02335|LN02Chr02:1460957-1461758(+)|206</td><td>CDD:224227</td><td>22.36</td><td>161</td><td>75</td><td>4</td><td>49</td><td>205</td><td>7</td><td>121</td><td>7.80E-14</td><td>62.8</td><td>COG1308</td><td>EGD2</td><td>Transcription factor homologous to NACalpha-BTF3</td></tr>
</table>

LN02_02399	LN02Chr02:1703947-1704929(+)	182	CDD:223725	49.686	159	62	5	23	176	6	151	2.24E-67	200	COG0652	PpiB	Peptidyl-prolyl cis-trans isomerase (rotamase) - cyclophilin family
LN02_02463	LN02Chr02:1902192-1904715(+)	766	CDD:223589	25.445	393	233	11	6	373	1	358	1.98E-42	156	COG0515	SPS1	Serine/threonine protein kinase
LN02_02591	LN02Chr02:2340121-2341461(-)	446	CDD:223481	37.845	399	220	11	51	444	1	376	1.95E-111	330	COG0404	GcvT	Glycine cleavage system T protein (aminomethyltransferase)
LN02_02975	LN02Chr02:3655255-3655869(+)	185	CDD:225057	32.051	78	43	3	81	156	30	99	1.59E-12	58.1	COG2146	{NirD	Ferredoxin subunits of nitrite reductase and ring-hydroxylating dioxygenases
LN02_03359	LN02Chr02:4979478-4981183(-)	460	CDD:226654	22.951	61	39	1	286	346	499	551	2.41E-04	40.4	COG4191	COG4191	Signal transduction histidine kinase regulating C4-dicarboxylate transport system
LN02_03359	LN02Chr02:4979478-4981183(-)	460	CDD:223715	15.789	285	204	7	64	346	29	279	4.71E-04	39	COG0642	BaeS	Signal transduction histidine kinase
LN02_03487	LN02Chr02:5761536-5762344(+)	218	CDD:223527	36.946	203	112	5	3	199	1	193	8.78E-73	216	COG0450	AhpC	Peroxiredoxin
LN02_03551	LN02Chr02:6131590-6135664(+)	977	CDD:223385	29.105	883	570	22	111	967	7	859	0	602	COG0308	PepN	Aminopeptidase N
LN02_03615	LN02Chr02:6672947-6674148(+)	379	CDD:224325	20	90	58	5	45	125	24	108	1.71E-05	42.7	COG1407	COG1407	Predicted ICC-like phosphoesterases
LN02_03679	LN02Chr03:281931-282434(-)	167	CDD:226636	24.107	112	63	6	7	105	13	115	2.83E-04	37.4	COG4166	OppA	ABC-type oligopeptide transport system, periplasmic component
LN02_04255	LN02Chr03:3576061-3576836(+)	132	CDD:223611	41.176	136	75	2	1	131	1	136	7.23E-44	137	COG0537	Hit	Diadenosine tetraphosphate (Ap4A) hydrolase and other HIT family hydrolases
LN02_04447	LN02Chr03:4198399-4199472(-)	271	CDD:227444	33.333	105	65	2	167	266	818	922	3.22E-11	60.4	COG5113	UFD2	Ubiquitin fusion degradation protein 2
LN02_04447	LN02Chr03:4198399-4199472(-)	271	CDD:223533	30.392	102	70	1	1	101	164	265	7.05E-04	37.5	COG0457	NrfG	FOG: TPR repeat
LN02_04511	LN02Chr03:4403866-4405660(+)	533	CDD:225030	25.769	260	112	3	272	530	6	185	1.00E-54	180	COG2119	COG2119	Predicted membrane protein
LN02_04703	LN02Chr03:5116855-5118570(-)	503	CDD:227511	30.162	431	265	10	67	491	63	463	4.46E-86	270	COG5184	ATS1	Alpha-tubulin suppressor and related RCC1 domain-containing proteins
LN02_04767	LN02Chr03:5342615-5344373(-)	515	CDD:227352	21.024	371	202	9	149	511	10	297	7.66E-28	111	COG5019	CDC3	Septin family protein
LN02_04831	LN02Chr03:5567368-5568384(+)	255	CDD:223532	26.471	68	40	3	161	221	92	156	1.50E-06	44.2	COG0456	RimI	Acetyltransferases
LN02_04895	LN02Chr03:5894889-5896296(+)	363	CDD:223737	17.553	376	262	15	6	350	7	365	1.70E-13	68	COG0665	DadA	Glycine/D-amino acid oxidases (deaminating)
LN02_05023	LN02Chr04:374584-376773(+)	729	CDD:224557	33.333	720	394	20	41	726	42	709	2.79E-170	508	COG1643	HrpA	HrpA-like helicases
LN02_05087	LN02Chr04:574329-575108(+)	259	CDD:223774	27.419	186	124	5	10	193	4	180	2.06E-11	59.5	COG0702	COG0702	Predicted nucleoside-diphosphate-sugar epimerases
LN02_05215	LN02Chr04:1005806-1007516(+)	507	CDD:225180	18.75	192	145	5	50	237	28	212	1.65E-04	41.1	COG2271	UhpC	Sugar phosphate permease

LN02_05279|LN02Chr04<br>:1194608-1195219(-)|203	CDD:2263<br>14	20.492	122	74	4	9	127	2	103	4.09E-<br>09	50.1	COG37<br>91	COG379<br>1	Uncharacterized conserved protein
LN02_05663|LN02Chr04<br>:2782099-2782661(+)|127	CDD:2243<br>00	32.456	114	77	0	6	119	6	119	6.99E-<br>15	63.1	COG13<br>82	GimC	Prefoldin, chaperonin cofactor
LN02_05727|LN02Chr04<br>:3010604-3012285(+)|506	CDD:2244<br>15	42.105	380	199	7	49	425	2	363	2.30E-<br>121	358	COG14<br>98	SIK1	Protein implicated in ribosomal<br>biogenesis, Nop56p homolog
LN02_05855|LN02Chr04<br>:3418989-3420339(+)|396	CDD:2234<br>40	37.849	251	141	7	1	249	1	238	8.00E-<br>78	237	COG03<br>63	NagB	6-<br>phosphogluconolactonase/Glucosamine-<br>6-phosphate isomerase/deaminase
LN02_05919|LN02Chr04<br>:3621818-3622604(+)|181	CDD:2237<br>50	38.037	163	89	5	27	180	3	162	1.44E-<br>45	145	COG06<br>78	AHP1	Peroxiredoxin
LN02_06047|LN02Chr04<br>:4109388-4110710(-)|351	CDD:2277<br>02	18.77	309	189	7	7	314	3	250	2.40E-<br>18	80.4	COG54<br>15	COG541<br>5	Predicted integral membrane metal-<br>binding protein
LN02_06111|LN02Chr04<br>:4308651-4311881(-)|1076	CDD:2275<br>35	33.333	252	159	6	745	989	183	432	6.12E-<br>44	164	COG52<br>10	COG521<br>0	GTPase-activating protein
LN02_06239|LN02Chr04<br>:4754621-4758227(-)|1096	CDD:2230<br>92	40.291	963	486	23	139	1096	1	879	0	915	COG00<br>13	AlaS	Alanyl-tRNA synthetase
LN02_06303|LN02Chr04<br>:4970050-4977346(-)|2390	CDD:2273<br>55	39.123	1117	599	25	120	1218	19	1072	0	968	COG50<br>22	COG502<br>2	Myosin heavy chain
LN02_06303|LN02Chr04<br>:4970050-4977346(-)|2390	CDD:2241<br>17	17.845	863	600	15	980	1806	213	1002	1.52E-<br>21	99.8	COG11<br>96	Smc	Chromosome segregation ATPases
LN02_06303|LN02Chr04<br>:4970050-4977346(-)|2390	CDD:2241<br>17	17.492	646	487	10	1715	2317	257	899	2.10E-<br>20	96.3	COG11<br>96	Smc	Chromosome segregation ATPases
LN02_06367|LN02Chr04<br>:5167885-5168463(-)|123	CDD:2243<br>53	32.456	114	60	5	13	123	5	104	1.07E-<br>20	77.3	COG14<br>36	NtpG	Archaeal/vacuolar-type H+-ATPase<br>subunit F
LN02_06431|LN02Chr04<br>:5371787-5372972(+)|349	CDD:2240<br>92	31.511	311	172	9	17	316	25	305	3.87E-<br>49	165	COG11<br>71	IlvA	Threonine dehydratase
LN02_06623|LN02Chr05<br>:142133-143684(+)|246	CDD:2234<br>83	27.966	236	134	4	2	237	1	200	3.17E-<br>34	119	COG04<br>06	phoE	Broad specificity phosphatase PhoE and<br>related phosphatases
LN02_06687|LN02Chr05<br>:405766-407957(-)|704	CDD:2237<br>30	19.104	424	198	10	163	583	30	311	3.06E-<br>23	98.1	COG06<br>57	Aes	Esterase/lipase
LN02_06943|LN02Chr05<br>:1703810-<br>1712691(+)|1833	CDD:2264<br>06	26	100	63	3	1096	1191	767	859	3.27E-<br>04	42.5	COG38<br>89	COG388<br>9	Predicted solute binding protein
LN02_07071|LN02Chr05<br>:2460883-2462449(-)|402	CDD:2235<br>05	21.302	169	102	3	224	392	119	256	1.42E-<br>09	55.4	COG04<br>28	COG042<br>8	Predicted divalent heavy-metal cations<br>transporter
LN02_07135|LN02Chr05<br>:2690298-2692980(+)|858	CDD:2235<br>81	14.768	474	362	18	350	802	229	681	4.09E-<br>26	111	COG05<br>07	RecD	ATP-dependent exoDNAse (exonuclease<br>V), alpha subunit - helicase superfamily<br>I member
LN02_07135|LN02Chr05<br>:2690298-2692980(+)|858	CDD:2266<br>46	17.949	78	59	2	309	382	363	439	0.001	39.6	COG41<br>78	COG417<br>8	ABC-type uncharacterized transport<br>system, permease and ATPase<br>components
LN02_07199|LN02Chr05<br>:2890692-2898149(-)|919	CDD:2240<br>54	32.258	248	145	7	25	269	2	229	2.09E-<br>52	182	COG11<br>31	CcmA	ABC-type multidrug transport system,<br>ATPase component

LN02_07263ǀLN02Chr05:3133611-3135047(-)ǀ446	CDD:224283	47.454	432	198	9	16	446	1	404	0	511	COG1364	ArgJ	N-acetylglutamate synthase (N-acetylornithine aminotransferase)
LN02_07327ǀLN02Chr05:3350313-3351342(+)ǀ230	CDD:223711	30.457	197	131	3	33	226	33	226	1.23E-51	164	COG0638	PRE1	20S proteasome, alpha and beta subunits
LN02_07391ǀLN02Chr05:3601478-3602158(+)ǀ226	CDD:227883	36.25	160	97	3	65	223	28	183	3.19E-38	128	COG5596	TIM22	Mitochondrial import inner membrane translocase, subunit TIM22
LN02_07455ǀLN02Chr05:3832082-3833760(+)ǀ536	CDD:224136	21.088	147	98	4	51	196	54	183	1.64E-09	56.9	COG1215	COG1215	Glycosyltransferases, probably involved in cell wall biogenesis
LN02_07775ǀLN02Chr05:5200782-5202509(-)ǀ452	CDD:224871	29.95	404	259	14	14	413	6	389	3.58E-74	235	COG1960	CaiA	Acyl-CoA dehydrogenases
LN02_07967ǀLN02Chr05:5822803-5824019(+)ǀ371	CDD:223743	19.34	212	160	2	88	295	16	220	2.77E-08	50.9	COG0671	PgpB	Membrane-associated phospholipid phosphatase
LN02_08031ǀLN02Chr06:548528-549668(+)ǀ316	CDD:224790	24.615	130	77	5	187	315	106	215	1.09E-04	39.6	COG1878	COG1878	Kynurenine formamidase
LN02_08351ǀLN02Chr06:2197426-2200978(+)ǀ959	CDD:223657	21.359	309	168	9	637	941	20	257	1.35E-15	74.9	COG0584	UgpQ	Glycerophosphoryl diester phosphodiesterase
LN02_08351ǀLN02Chr06:2197426-2200978(+)ǀ959	CDD:223738	22.072	222	166	2	237	453	5	224	7.62E-11	60.2	COG0666	Arp	FOG: Ankyrin repeat
LN02_08415ǀLN02Chr06:2506255-2507148(-)ǀ222	CDD:227155	31	100	60	3	124	219	6	100	1.20E-12	59.3	COG4818	COG4818	Predicted membrane protein
LN02_08543ǀLN02Chr06:2915440-2919404(-)ǀ1301	CDD:227586	22.5	200	133	7	888	1083	512	693	5.65E-06	48	COG5261	IQG1	Protein involved in regulation of cellular morphogenesis/cytokinesis
LN02_08863ǀLN02Chr07:627051-627520(+)ǀ121	CDD:225660	38.028	71	42	1	28	98	52	120	2.10E-09	50.8	COG3118	COG3118	Thioredoxin domain-containing protein
LN02_09055ǀLN02Chr07:1271879-1272933(-)ǀ334	CDD:225869	28.07	228	111	13	1	205	1	198	8.75E-22	90.3	COG3332	COG3332	Uncharacterized conserved protein
LN02_09247ǀLN02Chr07:1929786-1931030(+)ǀ414	CDD:225726	32.506	403	216	11	12	412	9	357	1.38E-75	236	COG3185	COG3185	4-hydroxyphenylpyruvate dioxygenase and related hemolysins
LN02_00160ǀLN02Chr01:755545-758933(+)ǀ1085	CDD:223589	26.432	227	129	5	719	931	2	204	1.53E-30	122	COG0515	SPS1	Serine/threonine protein kinase
LN02_00224ǀLN02Chr01:1199032-1201343(-)ǀ727	CDD:227122	23.171	164	105	4	1	164	302	444	5.57E-09	55.9	COG4783	COG4783	Putative Zn-dependent protease, contains TPR repeats
LN02_00288ǀLN02Chr01:1567154-1568548(+)ǀ401	CDD:223566	24.852	338	211	15	30	359	1	303	1.07E-26	105	COG0492	TrxB	Thioredoxin reductase
LN02_00608ǀLN02Chr01:2681091-2683700(+)ǀ783	CDD:223732	27.063	606	378	16	163	766	1	544	1.39E-109	342	COG0659	SUL1	Sulfate permease and related transporters (MFS superfamily)
LN02_00672ǀLN02Chr01:2987751-2989149(+)ǀ428	CDD:223257	21.495	321	154	13	104	421	37	262	3.17E-28	109	COG0179	MhpD	2-keto-4-pentenoate hydratase/2-oxohepta-3-ene-1,7-dioic acid hydratase (catechol pathway)
LN02_00736ǀLN02Chr01:3206071-3208416(+)ǀ728	CDD:227512	24.919	618	414	8	93	710	48	615	3.25E-77	257	COG5185	HEC1	Protein involved in chromosome segregation, interacts with SMC proteins
LN02_00800ǀLN02Chr01:3453643-3455435(+)ǀ439	CDD:227602	31.557	488	223	9	6	432	7	444	2.36E-108	324	COG5277	COG5277	Actin and related proteins
LN02_00992ǀLN02Chr01:4036471-4038747(+)ǀ684	CDD:223380	39.906	426	229	8	256	681	6	404	8.24E-119	358	COG0303	MoeA	Molybdopterin biosynthesis enzyme

LN02_00992|LN02Chr01:4036471-4038747(+)|684	CDD:223595	34.783	161	99	4	4	162	6	162	1.06E-31	118	COG0521	MoaB	Molybdopterin biosynthesis enzymes
LN02_01056|LN02Chr01:4254787-4258333(-)|1161	CDD:224126	33.291	790	496	15	317	1093	19	790	0	582	COG1205	COG1205	Distinct helicase family with a unique C-terminal domain including a metal-binding cysteine cluster
LN02_01248|LN02Chr01:5040126-5044165(+)|1284	CDD:227506	30.01	993	533	30	18	991	29	878	4.72E-163	509	COG5179	TAF1	Transcription initiation factor TFIID, subunit TAF1
LN02_01248|LN02Chr01:5040126-5044165(+)|1284	CDD:225606	19.828	116	87	2	1161	1276	125	234	2.70E-04	41.9	COG3064	TolA	Membrane protein involved in colicin uptake
LN02_01440|LN02Chr01:5790898-5792682(+)|472	CDD:223449	28.846	416	261	11	61	472	3	387	2.67E-115	341	COG0372	GltA	Citrate synthase
LN02_01504|LN02Chr01:6012978-6014156(-)|327	CDD:225547	26.978	278	174	9	41	318	37	285	4.59E-38	134	COG3001	COG3001	Uncharacterized protein conserved in bacteria
LN02_01696|LN02Chr01:6788086-6788796(-)|236	CDD:224584	30.357	112	75	1	121	232	78	186	7.55E-19	78.5	COG1670	RimL	Acetyltransferases, including N-acetylases of ribosomal proteins
LN02_01760|LN02Chr01:6947844-6949330(+)|472	CDD:224224	26.582	79	40	2	247	308	181	258	3.96E-05	42.3	COG1305	COG1305	Transglutaminase-like enzymes, putative cysteine proteases
LN02_01824|LN02Chr01:7144374-7145957(-)|435	CDD:223553	16.071	336	279	2	43	376	4	338	1.40E-04	40.8	COG0477	ProP	Permeases of the major facilitator superfamily
LN02_02016|LN02Chr02:339856-341346(-)|403	CDD:223589	20.765	366	199	12	35	396	1	279	2.54E-14	70.9	COG0515	SPS1	Serine/threonine protein kinase
LN02_02080|LN02Chr02:551502-553590(-)|649	CDD:223395	31.703	593	332	12	35	623	1	524	5.52E-134	401	COG0318	CaiC	Acyl-CoA synthetases (AMP-forming)/AMP-acid ligases II
LN02_02144|LN02Chr02:728594-730037(+)|433	CDD:227640	28.638	213	132	5	87	298	22	215	1.57E-29	114	COG5333	CCL1	Cdk activating kinase (CAK)/RNA polymerase II transcription initiation/nucleotide excision repair factor TFIIH/TFIIK, cyclin H subunit
LN02_02592|LN02Chr02:2342048-2343755(-)|480	CDD:227556	29.091	220	149	5	260	477	217	431	4.44E-39	143	COG5231	VMA13	Vacuolar H+-ATPase V1 sector, subunit H
LN02_02656|LN02Chr02:2576983-2577899(-)|221	CDD:227825	32.353	170	106	2	12	180	7	168	1.13E-37	126	COG5538	SEC66	Endoplasmic reticulum translocation complex, subunit SEC66
LN02_02912|LN02Chr02:3442067-3446944(-)|1561	CDD:224055	28.096	541	361	6	1014	1550	51	567	1.16E-97	323	COG1132	MdlB	ABC-type multidrug transport system, ATPase and permease components
LN02_02912|LN02Chr02:3442067-3446944(-)|1561	CDD:224055	22.989	435	310	7	401	827	111	528	2.47E-42	161	COG1132	MdlB	ABC-type multidrug transport system, ATPase and permease components
LN02_03040|LN02Chr02:3892080-3892959(-)|190	CDD:227574	46.995	183	92	2	5	187	2	179	1.07E-62	189	COG5249	RER1	Golgi protein involved in Golgi-to-ER retrieval
LN02_03232|LN02Chr02:4577639-4579006(+)|420	CDD:227403	21.543	311	218	8	103	402	128	423	5.55E-14	70.4	COG5071	RPN5	26S proteasome regulatory complex component
LN02_03296|LN02Chr02:4777461-4778621(+)|339	CDD:224092	31.475	305	193	4	13	316	24	313	2.28E-72	225	COG1171	IlvA	Threonine dehydratase

LN02_03360∣LN02Chr02:4984303-4988583(+)∣1187	CDD:227409	38.795	1129	628	21	60	1176	1	1078	0	916	COG5077	COG5077	Ubiquitin carboxyl-terminal hydrolase
LN02_03424∣LN02Chr02:5162543-5166118(-)∣1122	CDD:225201	26.38	163	104	4	660	814	239	393	7.66E-05	43.5	COG2319	COG2319	FOG: WD40 repeat
LN02_03488∣LN02Chr02:5764989-5768348(+)∣1119	CDD:225597	33.784	74	43	2	282	353	85	154	8.99E-05	43.1	COG3055	COG3055	Uncharacterized protein conserved in bacteria
LN02_03552∣LN02Chr02:6136088-6137123(-)∣308	CDD:223774	22.093	258	181	6	2	258	1	239	8.96E-16	73	COG0702	COG0702	Predicted nucleoside-diphosphate-sugar epimerases
LN02_03616∣LN02Chr02:6676285-6678056(-)∣527	CDD:225371	22.404	183	123	5	61	233	13	186	6.06E-09	54.9	COG2814	AraJ	Arabinose efflux permease
LN02_03744∣LN02Chr03:519283-522048(+)∣656	CDD:224961	25.532	141	100	3	506	646	1	136	1.16E-13	65.9	COG2050	PaaI	HGG motif-containing thioesterase, possibly involved in aromatic compounds catabolism
LN02_04064∣LN02Chr03:1844871-1846016(+)∣303	CDD:223796	28.261	138	98	1	23	159	64	201	9.23E-12	61.5	COG0724	COG0724	RNA-binding proteins (RRM domain)
LN02_04128∣LN02Chr03:2752816-2753720(-)∣217	CDD:223159	25.561	223	153	7	1	217	11	226	1.39E-53	168	COG0081	RplA	Ribosomal protein L1
LN02_04320∣LN02Chr03:3782173-3783846(-)∣379	CDD:223589	28.866	291	180	8	20	286	3	290	3.89E-55	183	COG0515	SPS1	Serine/threonine protein kinase
LN02_04384∣LN02Chr03:3996220-4000506(-)∣1306	CDD:223715	37.402	254	140	6	696	948	91	326	2.85E-54	190	COG0642	BaeS	Signal transduction histidine kinase
LN02_04384∣LN02Chr03:3996220-4000506(-)∣1306	CDD:223855	33.333	123	75	3	1099	1219	4	121	3.28E-26	102	COG0784	CheY	FOG: CheY-like receiver
LN02_04384∣LN02Chr03:3996220-4000506(-)∣1306	CDD:225359	28.916	83	57	1	261	341	1	83	4.89E-10	54.3	COG2770	ResE	FOG: HAMP domain
LN02_04384∣LN02Chr03:3996220-4000506(-)∣1306	CDD:225359	28	75	52	1	445	517	1	75	4.81E-07	45.8	COG2770	ResE	FOG: HAMP domain
LN02_04384∣LN02Chr03:3996220-4000506(-)∣1306	CDD:225359	25.301	83	60	1	353	433	1	83	2.32E-06	43.9	COG2770	ResE	FOG: HAMP domain
LN02_04384∣LN02Chr03:3996220-4000506(-)∣1306	CDD:225359	25.333	75	54	1	537	609	1	75	7.50E-05	39.7	COG2770	ResE	FOG: HAMP domain
LN02_04384∣LN02Chr03:3996220-4000506(-)∣1306	CDD:223910	14.206	359	238	8	283	609	79	399	4.42E-09	57.3	COG0840	Tar	Methyl-accepting chemotaxis protein
LN02_04384∣LN02Chr03:3996220-4000506(-)∣1306	CDD:223910	16.949	177	130	3	560	735	80	240	1.11E-05	46.1	COG0840	Tar	Methyl-accepting chemotaxis protein
LN02_04384∣LN02Chr03:3996220-4000506(-)∣1306	CDD:223910	13.62	279	190	5	127	394	7	245	3.73E-05	44.6	COG0840	Tar	Methyl-accepting chemotaxis protein
LN02_04576∣LN02Chr03:4643957-4644817(-)∣162	CDD:223709	37.5	72	41	1	92	159	6	77	8.38E-07	41.9	COG0636	AtpE	F0F1-type ATP synthase, subunit c/Archaeal/vacuolar-type H+-ATPase, subunit K

LN02_04704 l LN02Chr03:5119370-5120696(-) l 418	CDD:223677	26.946	334	200	12	47	377	1	293	1.30E-37	136	COG0604	Qor	NADPH:quinone reductase and related Zn-dependent oxidoreductases
LN02_04768 l LN02Chr03:5347940-5350042(-) l 671	CDD:223733	25.331	529	295	14	101	621	25	461	1.20E-75	249	COG0661	AarF	Predicted unusual protein kinase
LN02_04832 l LN02Chr03:5568881-5569953(-) l 279	CDD:227560	31.496	254	153	5	21	271	16	251	5.60E-53	170	COG5235	RFA2	Single-stranded DNA-binding replication protein A (RPA), medium (30 kD) subunit
LN02_05088 l LN02Chr04:575682-578204(-) l 581	CDD:223444	35.067	596	314	16	1	577	1	542	2.73E-176	507	COG0367	AsnB	Asparagine synthase (glutamine-hydrolyzing)
LN02_05152 l LN02Chr04:769718-772120(+) l 647	CDD:227401	43.359	655	314	23	1	642	1	611	0	618	COG5069	SAC6	Ca2+-binding actin-bundling protein fimbrin/plastin (EF-Hand superfamily)
LN02_05280 l LN02Chr04:1195845-1197118(-) l 412	CDD:223669	21.56	218	139	5	51	254	21	220	2.67E-08	51.9	COG0596	MhpC	Predicted hydrolases or acyltransferases (alpha/beta hydrolase superfamily)
LN02_05600 l LN02Chr04:2593810-2595703(-) l 443	CDD:223483	29.949	197	127	5	225	413	1	194	9.12E-38	133	COG0406	phoE	Broad specificity phosphatase PhoE and related phosphatases
LN02_05664 l LN02Chr04:2783695-2785484(+) l 546	CDD:224229	22.881	118	81	4	375	489	3	113	7.30E-06	42.9	COG1310	COG1310	Predicted metal-dependent protease of the PAD1/JAB1 superfamily
LN02_05728 l LN02Chr04:3013422-3013966(-) l 153	CDD:319244	70	70	20	1	2	70	1	70	6.91E-26	91	COG5272	UBI4	UBI4; linked to 3D-structure.
LN02_05728 l LN02Chr04:3013422-3013966(-) l 153	CDD:224909	54.167	48	19	2	101	148	3	47	9.47E-17	67.1	COG1998	RPS31	Ribosomal protein S27AE
LN02_06048 l LN02Chr04:4111163-4114611(+) l 1113	CDD:224117	20.513	741	537	8	338	1055	224	935	9.04E-21	95.9	COG1196	Smc	Chromosome segregation ATPases
LN02_06112 l LN02Chr04:4313732-4319116(-) l 1794	CDD:227223	25.856	263	141	9	1358	1612	75	291	1.44E-13	71.5	COG4886	COG4886	Leucine-rich repeat (LRR) protein
LN02_06560 l LN02Chr04:5851371-5854667(-) l 740	CDD:226406	30.38	79	54	1	327	404	767	845	1.75E-04	41.8	COG3889	COG3889	Predicted solute binding protein
LN02_06688 l LN02Chr05:409660-411315(+) l 551	CDD:227719	26.042	192	93	9	173	347	23	182	2.24E-10	59.7	COG5432	RAD18	RING-finger-containing E3 ubiquitin ligase
LN02_07136 l LN02Chr05:2693971-2696423(+) l 748	CDD:223140	27.16	243	129	11	473	713	5	201	1.46E-22	93.5	COG0062	COG0062	Uncharacterized conserved protein
LN02_07264 l LN02Chr05:3137642-3139132(+) l 446	CDD:224105	23.548	310	203	7	157	443	1	299	8.87E-39	139	COG1184	GCD2	Translation initiation factor 2B subunit, eIF-2B alpha/beta/delta family
LN02_07520 l LN02Chr05:4049505-4051158(-) l 423	CDD:223506	30.986	355	214	6	38	388	17	344	4.11E-69	219	COG0429	COG0429	Predicted hydrolase of the alpha/beta-hydrolase fold
LN02_07712 l LN02Chr05:4983513-4984585(-) l 324	CDD:223653	26.16	237	147	4	38	253	9	238	6.61E-33	119	COG0580	GlpF	Glycerol uptake facilitator and related permeases (Major Intrinsic Protein Family)
LN02_07776 l LN02Chr05:5203643-5204723(-) l 205	CDD:224025	30.526	190	112	5	5	178	2	187	5.43E-42	138	COG1100	COG1100	GTPase SAR1 and related small G proteins
LN02_07968 l LN02Chr05:5824623-5825869(-) l 267	CDD:223203	31.624	234	124	10	31	254	1	208	2.84E-47	153	COG0125	Tmk	Thymidylate kinase

LN02_08032∣LN02Chr06:549814-551031(-)∣320	CDD:223959	24.272	206	131	8	32	234	6	189	1.67E-14	69.1	COG1028	FabG	Dehydrogenases with different specificities (related to short-chain alcohol dehydrogenases)
LN02_08480∣LN02Chr06:2751626-2752756(-)∣376	CDD:223200	34.579	214	125	5	162	371	76	278	2.62E-50	167	COG0122	AlkA	3-methyladenine DNA glycosylase/8-oxoguanine DNA glycosylase
LN02_08608∣LN02Chr06:3141106-3143226(+)∣706	CDD:223589	27.178	287	177	9	45	309	2	278	1.82E-42	155	COG0515	SPS1	Serine/threonine protein kinase
LN02_08800∣LN02Chr07:387261-388904(-)∣474	CDD:224931	23.913	92	63	2	366	454	98	185	1.50E-04	39.7	COG2020	STE14	Putative protein-S-isoprenylcysteine methyltransferase
LN02_08864∣LN02Chr07:627625-631453(-)∣995	CDD:225201	20.604	364	249	9	203	540	11	360	1.09E-27	114	COG2319	COG2319	FOG: WD40 repeat
LN02_08864∣LN02Chr07:627625-631453(-)∣995	CDD:225201	20.048	419	273	15	392	792	49	423	4.92E-05	43.9	COG2319	COG2319	FOG: WD40 repeat
LN02_09056∣LN02Chr07:1279590-1284059(-)∣1465	CDD:224054	29.433	282	171	7	851	1115	15	285	8.55E-48	170	COG1131	CcmA	ABC-type multidrug transport system, ATPase component
LN02_09056∣LN02Chr07:1279590-1284059(-)∣1465	CDD:224054	28.448	232	148	5	170	400	21	235	6.50E-43	156	COG1131	CcmA	ABC-type multidrug transport system, ATPase component
LN02_09120∣LN02Chr07:1479014-1482114(-)∣661	CDD:225201	21.134	388	239	15	303	644	78	444	1.79E-19	89	COG2319	COG2319	FOG: WD40 repeat
LN02_09248∣LN02Chr07:1931812-1933362(-)∣457	CDD:227480	43.187	433	218	8	19	447	11	419	6.15E-161	458	COG5151	SSL1	RNA polymerase II transcription initiation/nucleotide excision repair factor TFIIH, subunit SSL1
LN02_00097∣LN02Chr01:476274-477158(-)∣165	CDD:224025	35.507	138	85	3	8	141	2	139	2.70E-33	114	COG1100	COG1100	GTPase SAR1 and related small G proteins
LN02_00161∣LN02Chr01:760140-761669(+)∣475	CDD:223737	21.986	423	284	12	40	453	1	386	1.40E-41	149	COG0665	DadA	Glycine/D-amino acid oxidases (deaminating)
LN02_00353∣LN02Chr01:1876437-1880608(+)∣927	CDD:224149	26.368	201	124	5	152	348	208	388	1.28E-24	104	COG1228	HutI	Imidazolonepropionase and related amidohydrolases
LN02_00417∣LN02Chr01:2063876-2064709(-)∣277	CDD:225735	29.293	198	88	7	53	250	7	152	1.84E-32	114	COG3194	DAL3	Ureidoglycolate hydrolase
LN02_00481∣LN02Chr01:2247656-2249834(-)∣684	CDD:223651	36.464	543	294	16	100	630	29	532	4.67E-141	420	COG0578	GlpA	Glycerol-3-phosphate dehydrogenase
LN02_00545∣LN02Chr01:2431038-2432565(-)∣415	CDD:225862	35.523	411	219	11	12	379	20	427	2.89E-95	290	COG3325	ChiA	Chitinase
LN02_00673∣LN02Chr01:2989377-2990063(-)∣228	CDD:223698	29.126	206	139	4	4	207	1	201	3.52E-36	124	COG0625	Gst	Glutathione S-transferase
LN02_00737∣LN02Chr01:3209116-3209712(+)∣126	CDD:224277	40.179	112	64	2	10	118	5	116	4.19E-29	99.3	COG1358	RPL8A	Ribosomal protein HS6-type (S12/L30/L7a)
LN02_00801∣LN02Chr01:3456823-3457161(-)∣82	CDD:225328	30.882	68	43	2	6	69	3	70	1.30E-10	49.3	COG2608	CopZ	Copper chaperone
LN02_00929∣LN02Chr01:3806099-3806698(+)∣199	CDD:223271	32.086	187	105	8	7	188	4	173	7.32E-34	116	COG0193	Pth	Peptidyl-tRNA hydrolase
LN02_00993∣LN02Chr01:4039146-4040124(-)∣201	CDD:223709	35.897	78	46	1	122	195	2	79	1.89E-06	41.5	COG0636	AtpE	F0F1-type ATP synthase, subunit c/Archaeal/vacuolar-type H+-ATPase, subunit K

LN02_01057 lLN02Chr01:4259022-4262411(+)l996	CDD:224441	20.561	321	190	7	9	293	1	292	9.86E-23	99.6	COG1524	COG1524	Uncharacterized proteins of the AP superfamily
LN02_01249 lLN02Chr01:5052260-5054436(+)l668	CDD:227400	35.821	67	43	0	1	67	81	147	1.92E-06	47.7	COG5068	ARG80	Regulator of arginine metabolism and related MADS box-containing transcription factors
LN02_01313 lLN02Chr01:5231067-5232830(-)l535	CDD:225371	25.688	109	79	2	60	167	50	157	2.42E-11	62.6	COG2814	AraJ	Arabinose efflux permease
LN02_01377 lLN02Chr01:5493207-5495621(-)l770	CDD:227520	17.631	363	223	10	267	615	28	328	1.45E-11	64.3	COG5193	LHP1	La protein, small RNA-binding pol III transcript stabilizing protein and related La-motif-containing proteins involved in translation
LN02_01505 lLN02Chr01:6015210-6016910(-)l445	CDD:223123	49.364	393	187	4	37	427	3	385	2.97E-178	500	COG0045	SucC	Succinyl-CoA synthetase, beta subunit
LN02_01633 lLN02Chr01:6536356-6537393(+)l345	CDD:223677	31.402	328	198	6	22	338	15	326	1.24E-52	174	COG0604	Qor	NADPH:quinone reductase and related Zn-dependent oxidoreductases
LN02_01953 lLN02Chr02:89317-90393(+)l358	CDD:225714	20.71	169	100	6	151	309	83	227	1.05E-07	49.7	COG3173	COG3173	Predicted aminoglycoside phosphotransferase
LN02_02081 lLN02Chr02:555028-556188(-)l366	CDD:226297	21.406	313	209	12	51	344	51	345	3.24E-42	147	COG3774	OCH1	Mannosyltransferase OCH1 and related enzymes
LN02_02145 lLN02Chr02:731682-732198(+)l99	CDD:224869	39.189	74	42	1	1	74	9	79	3.81E-16	64.2	COG1958	LSM1	Small nuclear ribonucleoprotein (snRNP) homolog
LN02_02209 lLN02Chr02:938897-939691(+)l186	CDD:225051	25	76	46	3	21	93	77	144	5.72E-04	36.3	COG2140	COG2140	Thermophilic glucose-6-phosphate isomerase and related metalloenzymes
LN02_02273 lLN02Chr02:1187095-1188213(+)l372	CDD:224117	20.419	191	148	4	151	340	201	388	1.70E-06	47	COG1196	Smc	Chromosome segregation ATPases
LN02_02337 lLN02Chr02:1463806-1464528(-)l156	CDD:227410	52.027	148	70	1	1	147	4	151	3.44E-67	198	COG5078	COG5078	Ubiquitin-protein ligase
LN02_02657 lLN02Chr02:2578524-2580806(+)l649	CDD:227431	40.301	598	326	6	1	594	1	571	0	627	COG5100	NPL4	Nuclear pore protein
LN02_02721 lLN02Chr02:2777632-2778246(-)l143	CDD:227575	46.154	130	64	3	17	141	7	135	7.67E-35	115	COG5250	RPB4	RNA polymerase II, fourth largest subunit
LN02_02849 lLN02Chr02:3205741-3206981(-)l372	CDD:223796	18.777	229	164	7	6	232	18	226	8.84E-06	44.2	COG0724	COG0724	RNA-binding proteins (RRM domain)
LN02_02849 lLN02Chr02:3205741-3206981(-)l372	CDD:223796	22.785	79	57	1	293	367	129	207	7.34E-04	38	COG0724	COG0724	RNA-binding proteins (RRM domain)
LN02_03041 lLN02Chr02:3893833-3895383(-)l516	CDD:224374	29.478	441	289	9	77	516	23	442	7.96E-68	222	COG1457	CodB	Purine-cytosine permease and related proteins
LN02_03233 lLN02Chr02:4579275-4580612(-)l297	CDD:225353	20.874	206	142	7	56	245	11	211	3.14E-15	70.3	COG2755	TesA	Lysophospholipase L1 and related esterases
LN02_03361 lLN02Chr02:4989612-4991352(-)l530	CDD:223330	28.841	371	219	12	19	383	20	351	8.65E-65	211	COG0252	AnsB	L-asparaginase/archaeal Glu-tRNAGln amidotransferase subunit D
LN02_03361 lLN02Chr02:4989612-4991352(-)l530	CDD:223738	31.313	99	62	2	421	513	110	208	2.26E-10	57.9	COG0666	Arp	FOG: Ankyrin repeat
LN02_03745 lLN02Chr03:522448-523530(+)l360	CDD:224966	42.105	342	196	2	13	353	5	345	3.40E-107	315	COG2055	COG2055	Malate/L-lactate dehydrogenases

LN02_03937∣LN02Chr03:1182192-1185066(-)∣869	CDD:227881	30.105	764	469	14	12	759	15	729	4.94E-143	441	COG5594	COG5594	Uncharacterized integral membrane protein
LN02_04001∣LN02Chr03:1382008-1382974(-)∣289	CDD:224772	35.829	187	101	3	22	208	29	196	1.53E-40	137	COG1859	KptA	RNA:NAD 2'-phosphotransferase
LN02_04129∣LN02Chr03:2754497-2759285(+)∣1440	CDD:227830	19.26	1298	825	48	148	1397	251	1373	1.15E-68	251	COG5543	COG5543	Uncharacterized conserved protein
LN02_04321∣LN02Chr03:3785380-3786065(+)∣156	CDD:227410	37.302	126	67	6	1	123	1	117	9.24E-28	98.1	COG5078	COG5078	Ubiquitin-protein ligase
LN02_04385∣LN02Chr03:4005590-4009872(-)∣1375	CDD:227371	23.699	173	115	7	920	1088	1043	1202	3.06E-12	68.6	COG5038	COG5038	Ca2+-dependent lipid-binding protein, contains C2 domain
LN02_04577∣LN02Chr03:4647433-4651318(+)∣1200	CDD:227709	41.484	1092	496	24	203	1180	112	1174	0	841	COG5422	ROM1	RhoGEF, Guanine nucleotide exchange factor for Rho/Rac/Cdc42-like GTPases
LN02_04833∣LN02Chr03:5570803-5574364(+)∣934	CDD:223542	38.059	917	420	21	1	915	2	772	0	817	COG0466	Lon	ATP-dependent Lon protease, bacterial type
LN02_05089∣LN02Chr04:579607-580325(-)∣157	CDD:227410	44.371	151	78	2	3	153	5	149	5.58E-66	195	COG5078	COG5078	Ubiquitin-protein ligase
LN02_05153∣LN02Chr04:772448-774975(-)∣672	CDD:227389	36.293	518	264	9	157	672	59	512	7.36E-126	380	COG5056	ARE1	Acyl-CoA cholesterol acyltransferase
LN02_05281∣LN02Chr04:1197867-1198598(+)∣243	CDD:226357	21.429	56	34	1	18	73	43	88	6.52E-04	36.2	COG3837	COG3837	Uncharacterized conserved protein, contains double-stranded beta-helix domain
LN02_05345∣LN02Chr04:1447852-1452372(-)∣1421	CDD:225094	21.895	950	548	35	312	1243	1	774	8.12E-112	368	COG2183	Tex	Transcriptional accessory protein
LN02_05537∣LN02Chr04:2360872-2365974(+)∣1682	CDD:223164	22.547	1060	544	27	13	1064	6	796	4.41E-155	492	COG0086	RpoC	DNA-directed RNA polymerase, beta' subunit/160 kD subunit
LN02_05537∣LN02Chr04:2360872-2365974(+)∣1682	CDD:223164	27.885	104	69	3	1182	1281	406	507	3.73E-11	65	COG0086	RpoC	DNA-directed RNA polymerase, beta' subunit/160 kD subunit
LN02_05665∣LN02Chr04:2786672-2788431(+)∣517	CDD:223944	39.63	487	269	8	11	495	7	470	2.96E-152	440	COG1012	PutA	NAD-dependent aldehyde dehydrogenases
LN02_05729∣LN02Chr04:3014704-3015483(+)∣117	CDD:227167	63.636	110	35	1	1	110	1	105	1.02E-37	120	COG4830	RPS26B	Ribosomal protein S26
LN02_05857∣LN02Chr04:3425370-3427033(-)∣457	CDD:224733	35.623	393	221	12	17	408	1	362	1.13E-94	288	COG1820	NagA	N-acetylglucosamine-6-phosphate deacetylase
LN02_05921∣LN02Chr04:3627192-3629582(-)∣677	CDD:223336	21.245	273	164	8	14	258	1	250	1.24E-22	96.2	COG0258	Exo	5'-3' exonuclease (including N-terminal domain of PolI)
LN02_05985∣LN02Chr04:3912131-3917292(-)∣1679	CDD:227586	21.327	1402	692	44	283	1658	30	1046	7.65E-97	335	COG5261	IQG1	Protein involved in regulation of cellular morphogenesis/cytokinesis
LN02_06177∣LN02Chr04:4569546-4571266(+)∣476	CDD:225371	19.423	381	223	13	63	389	9	359	4.85E-04	39.1	COG2814	AraJ	Arabinose efflux permease
LN02_06241∣LN02Chr04:4766248-4769076(-)∣841	CDD:223861	22.115	312	151	6	371	671	33	263	3.58E-17	80.1	COG0790	COG0790	FOG: TPR repeat, SEL1 subfamily

LN02_06241|LN02Chr04:4766248-4769076(-)|841	CDD:223861	21.136	317	186	9	165	468	25	290	8.38E-13	67	COG0790	COG0790	FOG: TPR repeat, SEL1 subfamily
LN02_06433|LN02Chr04:5376542-5379267(-)|876	CDD:223855	25.694	144	69	3	673	814	4	111	6.78E-22	89.5	COG0784	CheY	FOG: CheY-like receiver
LN02_06561|LN02Chr04:5860639-5862296(+)|428	CDD:224149	23.009	452	239	19	19	422	9	399	8.95E-23	96.7	COG1228	HutI	Imidazolonepropionase and related amidohydrolases
LN02_06689|LN02Chr05:412244-416177(+)|1137	CDD:227444	29.044	816	566	7	303	1114	120	926	1.54E-166	514	COG5113	UFD2	Ubiquitin fusion degradation protein 2
LN02_06753|LN02Chr05:694950-696512(+)|495	CDD:224693	28.7	223	126	6	260	477	7	201	7.24E-50	166	COG1779	COG1779	C4-type Zn-finger protein
LN02_06753|LN02Chr05:694950-696512(+)|495	CDD:224693	30.994	171	110	4	41	208	3	168	5.75E-27	105	COG1779	COG1779	C4-type Zn-finger protein
LN02_06817|LN02Chr05:901835-903352(+)|407	CDD:227598	32.593	135	87	2	80	211	32	165	1.02E-22	94.8	COG5273	COG5273	Uncharacterized protein containing DHHC-type Zn finger
LN02_06945|LN02Chr05:1751764-1753398(-)|453	CDD:225201	26.712	292	205	6	155	441	167	454	4.51E-39	144	COG2319	COG2319	FOG: WD40 repeat
LN02_07393|LN02Chr05:3606405-3608451(-)|634	CDD:224250	27.329	161	95	8	151	305	444	588	3.78E-06	47	COG1331	COG1331	Highly conserved protein containing a thioredoxin domain
LN02_07457|LN02Chr05:3836806-3848774(+)|3870	CDD:227365	22.019	2625	1464	91	1264	3870	46	2105	0	618	COG5032	TEL1	Phosphatidylinositol kinase and protein kinases of the PI-3 kinase family
LN02_08161|LN02Chr06:1487084-1489411(-)|597	CDD:226406	22.599	177	113	5	320	481	695	862	7.60E-05	42.5	COG3889	COG3889	Predicted solute binding protein
LN02_08289|LN02Chr06:1994026-1995271(+)|384	CDD:223191	47.866	328	168	3	57	384	5	329	3.29E-164	460	COG0113	HemB	Delta-aminolevulinic acid dehydratase
LN02_08481|LN02Chr06:2753382-2753701(+)|87	CDD:224869	44.872	78	38	2	1	74	1	77	6.12E-19	70.8	COG1958	LSM1	Small nuclear ribonucleoprotein (snRNP) homolog
LN02_08545|LN02Chr06:2930339-2931907(+)|458	CDD:223612	31.754	422	237	17	51	451	16	407	1.05E-165	470	COG0538	Icd	Isocitrate dehydrogenases
LN02_08609|LN02Chr06:3143459-3144176(+)|170	CDD:223903	40.26	77	46	0	35	111	167	243	7.95E-21	85.4	COG0833	LysP	Amino acid transporters
LN02_08609|LN02Chr06:3143459-3144176(+)|170	CDD:223903	22.727	88	62	3	72	157	453	536	5.67E-07	45.3	COG0833	LysP	Amino acid transporters
LN02_08673|LN02Chr06:3394697-3397471(-)|87	CDD:223480	39.216	51	21	5	32	76	80	126	5.86E-04	34.1	COG0403	GcvP	Glycine cleavage system protein P (pyridoxal-binding), N-terminal domain
LN02_08865|LN02Chr07:634724-636027(+)|410	CDD:224454	28.493	365	240	6	10	374	7	350	6.48E-80	247	COG1537	PelA	Predicted RNA-binding proteins
LN02_08993|LN02Chr07:1064722-1066314(+)|530	CDD:225201	26.623	308	211	9	227	530	144	440	5.02E-39	145	COG2319	COG2319	FOG: WD40 repeat
LN02_09185|LN02Chr07:1682952-1683474(+)|150	CDD:227573	31.452	124	76	3	1	124	1	115	1.15E-16	68.8	COG5248	TAF19	Transcription initiation factor TFIID, subunit TAF13
LN02_09313|LN02Chr07:2164445-2166408(-)|609	CDD:225043	19.82	555	318	22	4	552	18	451	2.93E-34	132	COG2132	SufI	Putative multicopper oxidases
LN02_00098|LN02Chr01:482220-486181(+)|1183	CDD:225606	27.692	130	93	1	523	651	124	253	1.16E-13	71.1	COG3064	TolA	Membrane protein involved in colicin uptake

LN02_00162|LN02Chr01:762069-763114(+)|259	CDD:224817	44.375	160	85	2	53	212	2	157	2.32E-72	215	COG1905	NuoE	NADH:ubiquinone oxidoreductase 24 kD subunit
LN02_00290|LN02Chr01:1570565-1572518(-)|506	CDD:225371	20.619	388	271	13	50	428	13	372	1.55E-09	56.9	COG2814	AraJ	Arabinose efflux permease
LN02_00482|LN02Chr01:2251456-2252622(-)|339	CDD:223653	39.754	244	132	6	64	304	9	240	3.26E-69	213	COG0580	GlpF	Glycerol uptake facilitator and related permeases (Major Intrinsic Protein Family)
LN02_00674|LN02Chr01:2990982-2992944(-)|606	CDD:225646	24.112	535	322	12	59	572	4	475	7.58E-65	218	COG3104	PTR2	Dipeptide/tripeptide permease
LN02_00866|LN02Chr01:3627724-3629357(-)|526	CDD:223248	22.917	240	136	4	285	518	16	212	1.13E-29	113	COG0170	SEC59	Dolichol kinase
LN02_00930|LN02Chr01:3808666-3810477(+)|470	CDD:223083	46.318	421	212	5	27	445	1	409	5.73E-162	461	COG0004	AmtB	Ammonia permease
LN02_00994|LN02Chr01:4040749-4041310(+)|115	CDD:227452	50	108	49	1	8	110	4	111	1.11E-42	133	COG5123	TOA2	Transcription initiation factor IIA, gamma subunit
LN02_01314|LN02Chr01:5235117-5235795(-)|185	CDD:223611	33.673	98	58	1	23	120	21	111	6.26E-20	78.6	COG0537	Hit	Diadenosine tetraphosphate (Ap4A) hydrolase and other HIT family hydrolases
LN02_01378|LN02Chr01:5501565-5502964(-)|217	CDD:227433	35.172	145	94	0	71	215	56	200	8.68E-43	140	COG5102	SFT2	Membrane protein involved in ER to Golgi transport
LN02_01442|LN02Chr01:5796311-5799803(-)|1047	CDD:225201	16.312	423	284	9	425	841	9	367	5.89E-12	66.3	COG2319	COG2319	FOG: WD40 repeat
LN02_01506|LN02Chr01:6017915-6019905(+)|534	CDD:225035	24.25	400	225	15	118	505	51	384	1.99E-39	145	COG2124	CypX	Cytochrome P450
LN02_01634|LN02Chr01:6537791-6540141(-)|738	CDD:223453	71.468	729	193	7	2	727	14	730	0	1248	COG0376	KatG	Catalase (peroxidase I)
LN02_01698|LN02Chr01:6796419-6797905(+)|272	CDD:225136	24.599	187	90	10	64	205	47	227	5.12E-10	55.3	COG2226	UbiE	Methylase involved in ubiquinone/menaquinone biosynthesis
LN02_01826|LN02Chr01:7150142-7151628(+)|458	CDD:227680	20.144	417	292	11	45	443	121	514	3.95E-45	161	COG5391	COG5391	Phox homology (PX) domain protein
LN02_01890|LN02Chr01:7336297-7338600(+)|712	CDD:225112	18.182	121	94	2	299	417	114	231	1.66E-04	41	COG2202	AtoS	FOG: PAS/PAC domain
LN02_01954|LN02Chr02:92753-95319(-)|786	CDD:224136	22.714	339	241	6	218	551	14	336	1.99E-43	160	COG1215	COG1215	Glycosyltransferases, probably involved in cell wall biogenesis
LN02_02146|LN02Chr02:732577-734239(-)|434	CDD:224868	28.831	385	181	12	2	383	1	295	4.75E-53	177	COG1957	URH1	Inosine-uridine nucleoside N-ribohydrolase
LN02_02210|LN02Chr02:940027-941069(-)|243	CDD:223257	45.249	221	105	8	19	235	58	266	3.34E-81	241	COG0179	MhpD	2-keto-4-pentenoate hydratase/2-oxohepta-3-ene-1,7-dioic acid hydratase (catechol pathway)
LN02_02338|LN02Chr02:1465564-1470647(+)|1376	CDD:225201	21.379	290	213	7	10	296	85	362	6.13E-22	97.9	COG2319	COG2319	FOG: WD40 repeat
LN02_02594|LN02Chr02:2353984-2355197(-)|192	CDD:223175	27.895	190	121	5	1	189	1	175	2.77E-45	144	COG0097	RplF	Ribosomal protein L6P/L9E

LN02_02658|LN02Chr02:2581811-2585762(-)|1171	CDD:223534	55.838	394	171	2	100	491	1	393	0	575	COG0458	CarB	Carbamoylphosphate synthase large subunit (split gene in MJ)
LN02_02658|LN02Chr02:2581811-2585762(-)|1171	CDD:223534	34.243	403	238	9	642	1029	1	391	5.97E-60	208	COG0458	CarB	Carbamoylphosphate synthase large subunit (split gene in MJ)
LN02_02722|LN02Chr02:2778924-2779659(+)|130	CDD:227451	36.522	115	56	3	15	129	1	98	3.89E-22	82.3	COG5122	TRS23	Transport protein particle (TRAPP) complex subunit
LN02_02914|LN02Chr02:3454138-3456522(+)|715	CDD:225201	22.523	222	138	4	22	215	249	464	3.40E-20	91.3	COG2319	COG2319	FOG: WD40 repeat
LN02_03042|LN02Chr02:3896094-3898424(+)|721	CDD:223658	28.5	400	205	10	196	593	43	363	1.09E-61	209	COG0585	COG0585	Uncharacterized conserved protein
LN02_03234|LN02Chr02:4581483-4583602(+)|602	CDD:223216	37.48	611	270	12	6	602	3	515	0	603	COG0138	PurH	AICAR transformylase/IMP cyclohydrolase PurH (only IMP cyclohydrolase domain in Aful)
LN02_03298|LN02Chr02:4780212-4783111(-)|929	CDD:223453	22.137	131	58	2	121	210	156	283	4.83E-06	47.4	COG0376	KatG	Catalase (peroxidase I)
LN02_03362|LN02Chr02:4992294-4994327(+)|532	CDD:223589	26.879	346	209	11	36	372	1	311	9.10E-48	167	COG0515	SPS1	Serine/threonine protein kinase
LN02_03426|LN02Chr02:5169250-5171881(-)|838	CDD:223370	41.791	201	117	0	4	204	5	205	1.16E-81	257	COG0293	FtsJ	23S rRNA methylase
LN02_03490|LN02Chr02:5769361-5770614(-)|417	CDD:223657	39.13	46	27	1	72	117	9	53	8.01E-08	50.2	COG0584	UgpQ	Glycerophosphoryl diester phosphodiesterase
LN02_03618|LN02Chr02:6682651-6690241(-)|2413	CDD:225858	31.677	1127	658	25	13	1130	2	1025	0	649	COG3321	COG3321	Polyketide synthase modules and related proteins
LN02_03618|LN02Chr02:6682651-6690241(-)|2413	CDD:223677	31.636	275	174	7	1749	2018	61	326	1.50E-40	150	COG0604	Qor	NADPH:quinone reductase and related Zn-dependent oxidoreductases
LN02_03618|LN02Chr02:6682651-6690241(-)|2413	CDD:223959	26.897	145	99	6	2042	2183	9	149	3.33E-11	62.9	COG1028	FabG	Dehydrogenases with different specificities (related to short-chain alcohol dehydrogenases)
LN02_04130|LN02Chr03:2759493-2761599(-)|668	CDD:224117	21.127	142	107	3	509	646	294	434	2.21E-06	47.8	COG1196	Smc	Chromosome segregation ATPases
LN02_04258|LN02Chr03:3580500-3581013(-)|120	CDD:227443	42.857	105	57	1	5	109	14	115	2.54E-27	95.1	COG5112	UFD2	U1-like Zn-finger-containing protein
LN02_04322|LN02Chr03:3787106-3789346(+)|664	CDD:227487	21.37	613	410	13	18	627	13	556	3.31E-61	211	COG5158	SEC1	Proteins involved in synaptic transmission and general secretion, Sec1 family
LN02_04386|LN02Chr03:4013267-4017080(+)|1108	CDD:227651	35.714	126	69	4	820	942	3	119	1.14E-29	117	COG5347	COG5347	GTPase-activating protein that regulates ARFs (ADP-ribosylation factors), involved in ARF-mediated vesicular transport
LN02_04642|LN02Chr03:4893555-4896139(-)|779	CDD:227470	43.396	53	29	1	240	292	189	240	6.96E-14	72.3	COG5141	COG5141	PHD zinc finger-containing protein
LN02_04642|LN02Chr03:4893555-4896139(-)|779	CDD:224391	19.372	191	135	5	350	540	18	189	2.29E-11	63.1	COG1474	CDC6	Cdc6-related protein, AAA superfamily ATPase
LN02_04898|LN02Chr03:5899773-5900722(-)|252	CDD:226802	39.648	227	123	5	16	240	3	217	4.25E-74	222	COG4359	COG4359	Uncharacterized conserved protein

LN02_05026 | LN02Chr04 :379504-380826(-) | 440	CDD:227367	19.471	416	188	12	21	435	2	271	1.41E-27	108	COG5034	TNG2	Chromatin remodeling protein, contains PhD zinc finger
LN02_05154 | LN02Chr04 :776146-777520(+) | 222	CDD:223796	27.848	79	55	2	6	83	116	193	3.93E-07	46.9	COG0724	COG0724	RNA-binding proteins (RRM domain)
LN02_05346 | LN02Chr04 :1453219-1454115(+) | 251	CDD:223620	25.806	217	136	7	30	223	5	219	2.37E-19	81	COG0546	Gph	Predicted phosphatases
LN02_05538 | LN02Chr04 :2367273-2367739(-) | 136	CDD:224947	46.237	93	45	1	44	136	1	88	2.30E-31	104	COG2036	HHT1	Histones H3 and H4
LN02_05666 | LN02Chr04 :2789003-2792695(-) | 1139	CDD:225035	25.581	129	88	4	979	1106	267	388	1.45E-05	45.9	COG2124	CypX	Cytochrome P450
LN02_05730 | LN02Chr04 :3016010-3020612(-) | 1502	CDD:224117	24.822	1261	840	18	225	1478	3	1162	0	629	COG1196	Smc	Chromosome segregation ATPases
LN02_05858 | LN02Chr04 :3428549-3434494(+) | 1486	CDD:224389	31.3	377	224	10	33	407	7	350	1.17E-67	231	COG1472	BglX	Beta-glucosidase-related glycosidases
LN02_05986 | LN02Chr04 :3919098-3921394(+) | 696	CDD:224637	46.729	214	103	4	315	528	104	306	7.86E-81	257	COG1723	COG1723	Uncharacterized conserved protein
LN02_06114 | LN02Chr04 :4324911-4325798(-) | 295	CDD:223959	30.182	275	156	8	18	287	7	250	2.43E-47	156	COG1028	FabG	Dehydrogenases with different specificities (related to short-chain alcohol dehydrogenases)
LN02_06306 | LN02Chr04 :4982714-4984761(-) | 531	CDD:223669	17.049	305	195	8	130	431	21	270	1.04E-10	59.6	COG0596	MhpC	Predicted hydrolases or acyltransferases (alpha/beta hydrolase superfamily)
LN02_06370 | LN02Chr04 :5175763-5178427(+) | 791	CDD:223143	54.812	478	156	8	7	480	1	422	0	678	COG0065	LeuC	3-isopropylmalate dehydratase large subunit
LN02_06370 | LN02Chr04 :5175763-5178427(+) | 791	CDD:223144	51.5	200	84	6	555	754	3	189	1.08E-90	280	COG0066	LeuD	3-isopropylmalate dehydratase small subunit
LN02_06434 | LN02Chr04 :5380650-5381617(+) | 265	CDD:226607	38.342	193	92	6	73	262	48	216	1.30E-43	144	COG4122	COG4122	Predicted O-methyltransferase
LN02_06882 | LN02Chr05 :1156279-1157217(+) | 195	CDD:227457	35.294	68	39	4	94	156	69	136	3.55E-06	43	COG5128	COG5128	Transport protein particle (TRAPP) complex subunit
LN02_07138 | LN02Chr05 :2701671-2703127(+) | 468	CDD:227391	48.512	336	158	4	79	404	37	367	1.12E-110	329	COG5058	LAG1	Protein transporter of the TRAM (translocating chain-associating membrane) superfamily, longevity assurance factor
LN02_07330 | LN02Chr05 :3361004-3362567(-) | 452	CDD:224919	35.754	358	201	11	88	432	1	342	1.54E-86	265	COG2008	GLY1	Threonine aldolase
LN02_07394 | LN02Chr05 :3609770-3610427(+) | 170	CDD:224430	42.857	147	83	1	24	170	6	151	2.90E-51	158	COG1513	CynS	Cyanate lyase
LN02_07458 | LN02Chr05 :3850343-3852843(+) | 706	CDD:223442	29.223	592	340	22	84	669	1	519	1.93E-99	313	COG0365	Acs	Acyl-coenzyme A synthetases/AMP-(fatty) acid ligases
LN02_07522 | LN02Chr05 :4054874-4057326(+) | 746	CDD:223589	27.019	322	196	10	384	690	6	303	2.31E-46	167	COG0515	SPS1	Serine/threonine protein kinase
LN02_07650 | LN02Chr05 :4732254-4733865(-) | 327	CDD:223712	44.872	156	76	6	139	285	1	155	9.78E-43	142	COG0639	ApaH	Diadenosine tetraphosphatase and related serine/threonine protein phosphatases

LN02_07842∣LN02Chr05:5473319-5475002(+)∣506	CDD:225180	19.774	354	256	11	68	398	28	376	1.28E-09	57.3	COG2271	UhpC	Sugar phosphate permease
LN02_07906∣LN02Chr05:5650695-5652192(-)∣438	CDD:223737	20.665	421	288	12	1	413	1	383	3.62E-27	109	COG0665	DadA	Glycine/D-amino acid oxidases (deaminating)
LN02_08098∣LN02Chr06:1238553-1239449(+)∣270	CDD:227527	39.004	241	140	4	1	239	1	236	3.23E-68	209	COG5200	LUC7	U1 snRNP component, mediates U1 snRNP association with cap-binding complex
LN02_08162∣LN02Chr06:1491927-1501201(+)∣2215	CDD:226406	24.419	172	100	7	437	590	694	853	4.49E-05	45.6	COG3889	COG3889	Predicted solute binding protein
LN02_08290∣LN02Chr06:1997038-1998680(+)∣527	CDD:224748	19.07	430	248	12	105	517	16	362	3.55E-22	95.6	COG1835	COG1835	Predicted acyltransferases
LN02_08354∣LN02Chr06:2206381-2208812(+)∣783	CDD:223589	26.575	365	184	11	23	371	2	298	2.65E-41	152	COG0515	SPS1	Serine/threonine protein kinase
LN02_08610∣LN02Chr06:3144387-3145782(-)∣413	CDD:223560	36.415	357	193	11	21	369	3	333	1.05E-105	314	COG0484	DnaJ	DnaJ-class molecular chaperone with C-terminal Zn finger domain
LN02_08866∣LN02Chr07:637051-638210(+)∣275	CDD:223729	36.655	281	158	9	6	270	1	277	1.44E-83	249	COG0656	ARA1	Aldo/keto reductases, related to diketogulonate reductase
LN02_08930∣LN02Chr07:842901-844823(-)∣607	CDD:223704	23.103	290	149	8	199	484	34	253	4.01E-40	144	COG0631	PTC1	Serine/threonine protein phosphatase
LN02_09122∣LN02Chr07:1496883-1499946(+)∣849	CDD:223533	19.643	336	222	6	55	388	1	290	2.21E-30	118	COG0457	NrfG	FOG: TPR repeat
LN02_09250∣LN02Chr07:1939365-1940091(+)∣149	CDD:223167	34.568	81	50	1	65	142	1	81	7.67E-21	78.4	COG0089	RplW	Ribosomal protein L23
LN02_00163∣LN02Chr01:763577-765244(-)∣555	CDD:225597	32.653	49	33	0	177	225	81	129	5.68E-04	39.3	COG3055	COG3055	Uncharacterized protein conserved in bacteria
LN02_00483∣LN02Chr01:2257451-2259174(+)∣512	CDD:223628	50	508	241	7	4	510	1	496	0	660	COG0554	GlpK	Glycerol kinase
LN02_00547∣LN02Chr01:2434208-2436584(+)∣771	CDD:223589	35.548	301	152	6	193	473	1	279	3.66E-62	211	COG0515	SPS1	Serine/threonine protein kinase
LN02_00739∣LN02Chr01:3210775-3212188(+)∣410	CDD:225130	27.393	303	160	10	113	410	3	250	1.32E-40	142	COG2220	COG2220	Predicted Zn-dependent hydrolases of the beta-lactamase fold
LN02_00803∣LN02Chr01:3459618-3461018(-)∣466	CDD:223261	28.916	415	246	11	13	404	3	391	4.25E-65	212	COG0183	PaaJ	Acetyl-CoA acetyltransferase
LN02_00867∣LN02Chr01:3629854-3631858(+)∣632	CDD:223587	33.249	397	218	9	21	416	35	385	1.00E-92	293	COG0513	SrmB	Superfamily II DNA and RNA helicases
LN02_01123∣LN02Chr01:4483511-4486973(+)∣852	CDD:226751	32.432	296	182	9	59	345	30	316	4.16E-49	173	COG4301	COG4301	Uncharacterized conserved protein
LN02_01123∣LN02Chr01:4483511-4486973(+)∣852	CDD:224182	23.077	325	181	15	541	849	43	314	3.77E-25	104	COG1262	COG1262	Uncharacterized conserved protein
LN02_01315∣LN02Chr01:5236331-5236849(+)∣143	CDD:227363	59.441	143	57	1	1	143	1	142	3.92E-66	195	COG5030	APS2	Clathrin adaptor complex, small subunit
LN02_01507∣LN02Chr01:6020209-6020619(-)∣136	CDD:223427	44.444	90	39	2	31	118	87	167	7.38E-27	95.6	COG0350	Ada	Methylated DNA-protein cysteine methyltransferase

LN02_01571∣LN02Chr01:6301699-6303045(+)∣448	CDD:224953	46.341	164	87	1	64	227	17	179	1.59E-69	216	COG2042	COG2042	Uncharacterized conserved protein
LN02_01635∣LN02Chr01:6542136-6544365(+)∣633	CDD:223087	30.973	452	275	12	90	533	6	428	4.75E-112	341	COG0008	GlnS	Glutamyl- and glutaminyl-tRNA synthetases
LN02_01699∣LN02Chr01:6800413-6801024(+)∣203	CDD:224526	33.113	151	90	5	50	198	3	144	4.35E-18	74.7	COG1610	COG1610	Uncharacterized conserved protein
LN02_01827∣LN02Chr01:7152497-7155266(-)∣849	CDD:227271	29.412	170	109	5	481	646	2	164	1.26E-32	121	COG4935	COG4935	Regulatory P domain of the subtilisin-like proprotein convertases and other proteases
LN02_01827∣LN02Chr01:7152497-7155266(-)∣849	CDD:224322	29.688	256	152	9	181	421	138	380	4.02E-21	95	COG1404	AprE	Subtilisin-like serine proteases
LN02_01891∣LN02Chr01:7340594-7341884(-)∣360	CDD:226022	33.628	339	204	10	1	336	2	322	1.56E-58	189	COG3491	PcbC	Isopenicillin N synthase and related dioxygenases
LN02_02083∣LN02Chr02:560535-563031(+)∣733	CDD:226256	33.763	699	376	22	5	689	29	654	2.37E-151	452	COG3733	TynA	Cu2+-containing amine oxidase
LN02_02211∣LN02Chr02:941681-944760(-)∣842	CDD:227934	28.832	822	508	18	31	842	19	773	2.95E-132	410	COG5647	COG5647	Cullin, a subunit of E3 ubiquitin ligase
LN02_02403∣LN02Chr02:1715194-1718055(-)∣847	CDD:223796	26.316	152	106	3	320	470	115	261	1.07E-15	75.8	COG0724	COG0724	RNA-binding proteins (RRM domain)
LN02_02403∣LN02Chr02:1715194-1718055(-)∣847	CDD:223796	28.261	92	65	1	707	797	100	191	7.77E-09	55	COG0724	COG0724	RNA-binding proteins (RRM domain)
LN02_02403∣LN02Chr02:1715194-1718055(-)∣847	CDD:223796	17.799	309	222	6	409	701	12	304	1.52E-08	54.2	COG0724	COG0724	RNA-binding proteins (RRM domain)
LN02_02403∣LN02Chr02:1715194-1718055(-)∣847	CDD:223796	24.138	87	61	1	2	83	112	198	2.53E-06	47.2	COG0724	COG0724	RNA-binding proteins (RRM domain)
LN02_02659∣LN02Chr02:2587468-2588284(-)∣190	CDD:226914	37.791	172	101	1	3	174	2	167	1.77E-34	117	COG4539	COG4539	Predicted membrane protein
LN02_02723∣LN02Chr02:2779875-2780792(-)∣284	CDD:227477	44.813	241	117	3	1	241	1	225	6.12E-79	236	COG5148	RPN10	26S proteasome regulatory complex, subunit RPN10/PSMD4
LN02_02851∣LN02Chr02:3210827-3211735(+)∣239	CDD:227555	39.07	215	110	2	24	238	1	194	4.18E-71	213	COG5230	COG5230	Uncharacterized conserved protein
LN02_03043∣LN02Chr02:3899006-3901517(-)∣715	CDD:223589	30.496	282	170	10	422	687	7	278	7.37E-44	159	COG0515	SPS1	Serine/threonine protein kinase
LN02_03491∣LN02Chr02:5771902-5773204(-)∣393	CDD:225862	24.848	330	191	15	75	366	113	423	7.76E-29	114	COG3325	ChiA	Chitinase
LN02_03555∣LN02Chr02:6158074-6161924(-)∣1020	CDD:226406	17.647	187	122	4	609	780	681	850	7.50E-06	46.8	COG3889	COG3889	Predicted solute binding protein
LN02_03555∣LN02Chr02:6158074-6161924(-)∣1020	CDD:226406	22.564	195	118	5	519	699	681	856	2.71E-05	45.2	COG3889	COG3889	Predicted solute binding protein
LN02_03555∣LN02Chr02:6158074-6161924(-)∣1020	CDD:226406	19.643	168	114	5	402	569	710	856	5.81E-04	40.6	COG3889	COG3889	Predicted solute binding protein

LN02_03619∣LN02Chr02:6691575-6699511(+)∣2602	CDD:225858	29.002	1062	678	26	395	1434	3	1010	2.76E-163	532	COG3321	COG3321	Polyketide synthase modules and related proteins
LN02_03619∣LN02Chr02:6691575-6699511(+)∣2602	CDD:225857	27.652	264	155	10	2252	2509	3	236	7.60E-25	106	COG3320	COG3320	Putative dehydrogenase domain of multifunctional non-ribosomal peptide synthetases and related enzymes
LN02_03683∣LN02Chr03:298843-300129(+)∣411	CDD:223589	26.829	123	77	4	134	247	28	146	2.41E-07	49.4	COG0515	SPS1	Serine/threonine protein kinase
LN02_03875∣LN02Chr03:986911-988646(-)∣536	CDD:227404	18.935	338	198	13	131	440	136	425	1.38E-14	73	COG5072	ALK1	Serine/threonine kinase of the haspin family
LN02_03939∣LN02Chr03:1190784-1195665(-)∣1558	CDD:225942	21.814	463	232	18	1092	1549	270	607	2.76E-42	162	COG3408	GDB1	Glycogen debranching enzyme
LN02_03939∣LN02Chr03:1190784-1195665(-)∣1558	CDD:223443	27.368	95	53	6	172	256	28	116	6.51E-04	41.1	COG0366	AmyA	Glycosidases
LN02_04003∣LN02Chr03:1395101-1397572(+)∣823	CDD:225297	22.917	144	90	5	356	498	26	149	1.27E-10	58.2	COG2453	CDC14	Predicted protein-tyrosine phosphatase
LN02_04259∣LN02Chr03:3581869-3584632(+)∣787	CDD:223977	31.473	842	452	25	60	783	23	857	0	768	COG1048	AcnA	Aconitase A
LN02_04323∣LN02Chr03:3790422-3793586(+)∣1032	CDD:224152	21.549	297	191	10	556	838	176	444	6.95E-22	97.1	COG1231	COG1231	Monoamine oxidase
LN02_04323∣LN02Chr03:3790422-3793586(+)∣1032	CDD:224154	23.2	125	68	5	261	384	6	103	3.22E-06	47.8	COG1233	COG1233	Phytoene dehydrogenase and related proteins
LN02_04323∣LN02Chr03:3790422-3793586(+)∣1032	CDD:227935	26	100	60	3	903	996	76	167	7.36E-05	42.2	COG5648	NHP6B	Chromatin-associated proteins containing the HMG domain
LN02_04387∣LN02Chr03:4019018-4021488(+)∣677	CDD:223589	32.381	315	194	6	290	589	2	312	3.49E-66	220	COG0515	SPS1	Serine/threonine protein kinase
LN02_04387∣LN02Chr03:4019018-4021488(+)∣677	CDD:224630	43.038	79	38	3	199	276	91	163	4.73E-12	62.7	COG1716	COG1716	FOG: FHA domain
LN02_04643∣LN02Chr03:4898197-4899856(+)∣436	CDD:224151	30.938	320	166	8	99	415	29	296	9.88E-59	191	COG1230	CzcD	Co/Zn/Cd efflux system component
LN02_04707∣LN02Chr03:5127212-5128237(+)∣341	CDD:224083	30.097	309	166	11	23	307	19	301	6.64E-38	135	COG1161	COG1161	Predicted GTPases
LN02_04771∣LN02Chr03:5360333-5362290(+)∣545	CDD:223192	63.578	464	164	3	79	541	3	462	0	798	COG0114	FumC	Fumarase
LN02_05027∣LN02Chr04:382405-383904(+)∣499	CDD:223201	33.022	321	192	6	74	389	9	311	2.17E-98	297	COG0123	AcuC	Deacetylases, including yeast histone deacetylase and acetoin utilization protein
LN02_05347∣LN02Chr04:1454780-1456668(-)∣512	CDD:223589	29.114	316	187	12	65	356	3	305	2.71E-42	152	COG0515	SPS1	Serine/threonine protein kinase
LN02_05603∣LN02Chr04:2606951-2608891(-)∣555	CDD:224019	23.214	168	106	8	297	462	12	158	1.49E-10	57.7	COG1094	COG1094	Predicted RNA-binding protein (contains KH domains)

LN02_05603丨LN02Chr04 :2606951-2608891(-)丨555	CDD:224019	25.405	185	92	6	195	362	8	163	5.53E-06	44.2	COG1094	COG1094	Predicted RNA-binding protein (contains KH domains)
LN02_05667丨LN02Chr04 :2794976-2797052(-)丨632	CDD:223699	25.843	356	193	13	269	608	79	379	1.27E-50	177	COG0626	MetC	Cystathionine beta-lyases/cystathionine gamma-synthases
LN02_05859丨LN02Chr04 :3435242-3436252(+)丨210	CDD:224931	22.222	144	106	3	46	188	24	162	4.17E-04	37	COG2020	STE14	Putative protein-S-isoprenylcysteine methyltransferase
LN02_05987丨LN02Chr04 :3923833-3925070(-)丨364	CDD:223769	16.61	295	226	5	55	344	10	289	8.29E-05	41	COG0697	RhaT	Permeases of the drug/metabolite transporter (DMT) superfamily
LN02_06051丨LN02Chr04 :4119345-4120745(-)丨466	CDD:227445	31.325	83	54	1	377	459	351	430	1.95E-06	47	COG5114	COG5114	Histone acetyltransferase complex SAGA/ADA, subunit ADA2
LN02_06819丨LN02Chr05 :909620-910389(+)丨201	CDD:224117	26.531	98	72	0	82	179	746	843	5.08E-06	43.5	COG1196	Smc	Chromosome segregation ATPases
LN02_06883丨LN02Chr05 :1157664-1159224(-)丨306	CDD:223117	33.033	333	165	16	2	299	1	310	4.29E-59	188	COG0039	Mdh	Malate/lactate dehydrogenases
LN02_07011丨LN02Chr05 :1950706-1953112(-)丨774	CDD:223796	28.866	97	61	3	282	372	110	204	3.05E-10	59.2	COG0724	COG0724	RNA-binding proteins (RRM domain)
LN02_07011丨LN02Chr05 :1950706-1953112(-)丨774	CDD:223796	29.545	88	55	2	498	579	115	201	2.41E-08	53.4	COG0724	COG0724	RNA-binding proteins (RRM domain)
LN02_07011丨LN02Chr05 :1950706-1953112(-)丨774	CDD:223796	20.856	187	112	5	421	571	115	301	3.73E-07	49.6	COG0724	COG0724	RNA-binding proteins (RRM domain)
LN02_07203丨LN02Chr05 :2910319-2915891(+)丨531	CDD:223589	26.136	264	179	6	53	315	4	252	1.79E-34	131	COG0515	SPS1	Serine/threonine protein kinase
LN02_07331丨LN02Chr05 :3364911-3365714(-)丨183	CDD:224025	28.788	132	90	1	20	147	5	136	1.09E-26	98.1	COG1100	COG1100	GTPase SAR1 and related small G proteins
LN02_07395丨LN02Chr05 :3611041-3611865(+)丨274	CDD:223315	36.245	229	112	10	1	229	2	196	4.44E-48	155	COG0237	CoaE	Dephospho-CoA kinase
LN02_07715丨LN02Chr05 :4993171-4994163(-)丨330	CDD:223959	27.542	236	137	7	26	256	2	208	6.67E-20	84.5	COG1028	FabG	Dehydrogenases with different specificities (related to short-chain alcohol dehydrogenases)
LN02_07779丨LN02Chr05 :5211897-5213676(-)丨567	CDD:227664	33.333	465	286	13	109	565	99	547	5.60E-135	401	COG5371	COG5371	Golgi nucleoside diphosphatase
LN02_07843丨LN02Chr05 :5475569-5476729(+)丨358	CDD:224025	17.16	169	126	5	29	194	8	165	2.80E-06	45	COG1100	COG1100	GTPase SAR1 and related small G proteins
LN02_07907丨LN02Chr05 :5652895-5654238(+)丨415	CDD:226985	23.009	339	214	9	29	336	5	327	2.89E-39	141	COG4638	HcaE	Phenylpropionate dioxygenase and related ring-hydroxylating dioxygenases, large terminal subunit
LN02_08291丨LN02Chr06 :1999178-2001292(-)丨605	CDD:224490	31.183	558	325	16	49	587	16	533	1.45E-96	302	COG1574	COG1574	Predicted metal-dependent hydrolase with the TIM-barrel fold
LN02_08355丨LN02Chr06 :2211370-2213893(+)丨649	CDD:223520	20.755	371	225	17	66	424	5	318	2.35E-19	88.9	COG0443	DnaK	Molecular chaperone
LN02_08547丨LN02Chr06 :2935106-2937473(-)丨703	CDD:227487	24.604	695	404	15	7	699	3	579	3.93E-123	376	COG5158	SEC1	Proteins involved in synaptic transmission and general secretion, Sec1 family

LN02_08611|LN02Chr06:3146433-3149576(+)|1047	CDD:224557	41.597	714	361	15	360	1027	4	707	0	735	COG1643	HrpA	HrpA-like helicases
LN02_08867|LN02Chr07:638772-639581(+)|269	CDD:227665	25.882	85	59	2	176	260	55	135	1.03E-05	43.7	COG5373	COG5373	Predicted membrane protein
LN02_08931|LN02Chr07:847004-847941(-)|224	CDD:227778	26.904	197	133	5	38	223	4	200	3.57E-19	79.5	COG5491	VPS24	Conserved protein implicated in secretion
LN02_09123|LN02Chr07:1500627-1502172(+)|391	CDD:223165	24.092	303	133	10	43	342	1	209	4.17E-54	175	COG0087	RplC	Ribosomal protein L3
LN02_09187|LN02Chr07:1684397-1685793(+)|454	CDD:223769	18.885	323	216	4	75	395	14	292	2.24E-11	61.4	COG0697	RhaT	Permeases of the drug/metabolite transporter (DMT) superfamily
LN02_09251|LN02Chr07:1943044-1944171(+)|346	CDD:225546	26.718	262	174	5	65	318	5	256	8.61E-40	138	COG3000	ERG3	Sterol desaturase
LN02_09315|LN02Chr07:2174119-2175528(+)|469	CDD:225370	21.212	66	47	1	152	217	155	215	1.33E-04	40.7	COG2813	RsmC	16S RNA G1207 methylase RsmC
LN02_00612|LN02Chr01:2692665-2693644(-)|309	CDD:224155	22.222	261	150	14	50	285	31	263	5.56E-08	50.1	COG1234	ElaC	Metal-dependent hydrolases of the beta-lactamase superfamily III
LN02_00676|LN02Chr01:2996647-2998453(-)|520	CDD:225035	19.075	346	210	14	146	487	102	381	7.89E-23	97.9	COG2124	CypX	Cytochrome P450
LN02_00868|LN02Chr01:3633243-3637389(-)|1078	CDD:227432	50.518	1061	502	7	5	1063	13	1052	0	1301	COG5101	CRM1	Importin beta-related nuclear transport receptor
LN02_00932|LN02Chr01:3816039-3818522(-)|751	CDD:225882	32.628	662	401	11	92	744	56	681	4.11E-152	456	COG3345	GalA	Alpha-galactosidase
LN02_00996|LN02Chr01:4043128-4046071(-)|839	CDD:223116	23.81	483	303	10	107	588	23	441	7.12E-55	193	COG0038	EriC	Chloride channel protein EriC
LN02_00996|LN02Chr01:4043128-4046071(-)|839	CDD:223591	19.632	163	79	5	595	753	1	115	1.42E-06	45.2	COG0517	COG0517	FOG: CBS domain
LN02_01060|LN02Chr01:4272507-4274575(+)|665	CDD:227917	31.933	357	170	10	304	659	211	495	1.78E-68	229	COG5630	ARG2	Acetylglutamate synthase
LN02_01124|LN02Chr01:4487428-4488732(-)|338	CDD:224105	29.114	316	202	8	25	334	2	301	7.45E-50	166	COG1184	GCD2	Translation initiation factor 2B subunit, eIF-2B alpha/beta/delta family
LN02_01252|LN02Chr01:5058599-5060931(+)|625	CDD:223981	29.873	472	250	17	5	463	9	412	2.39E-74	245	COG1053	SdhA	Succinate dehydrogenase/fumarate reductase, flavoprotein subunit
LN02_01252|LN02Chr01:5058599-5060931(+)|625	CDD:227599	42.857	84	48	0	540	623	48	131	9.59E-19	81	COG5274	CYB5	Cytochrome b involved in lipid metabolism
LN02_01508|LN02Chr01:6021180-6026003(-)|1579	CDD:227511	20.117	343	210	14	183	512	120	411	4.08E-12	67.6	COG5184	ATS1	Alpha-tubulin suppressor and related RCC1 domain-containing proteins
LN02_01508|LN02Chr01:6021180-6026003(-)|1579	CDD:227511	16.91	343	214	19	379	688	60	364	1.08E-06	49.8	COG5184	ATS1	Alpha-tubulin suppressor and related RCC1 domain-containing proteins
LN02_01572|LN02Chr01:6303274-6306381(-)|982	CDD:223606	46.095	525	261	5	461	981	1	507	0	598	COG0532	InfB	Translation initiation factor 2 (IF-2; GTPase)
LN02_01700|LN02Chr01:6801700-6802276(+)|134	CDD:223329	30.469	128	87	2	1	126	2	129	1.78E-30	103	COG0251	TdcF	Putative translation initiation inhibitor, yjgF family
LN02_01892|LN02Chr01:7343385-7343806(-)|106	CDD:225660	28.155	103	61	5	8	102	29	126	3.94E-08	46.6	COG3118	COG3118	Thioredoxin domain-containing protein

LN02_02020 | LN02Chr02 :348673-350142(-) | 489	CDD:223769	20.225	267	198	4	164	429	35	287	9.46E-06	44.5	COG0697	RhaT	Permeases of the drug/metabolite transporter (DMT) superfamily
LN02_02084 | LN02Chr02 :563389-564402(-) | 277	CDD:223235	35.986	289	154	8	10	277	1	279	1.69E-55	177	COG0157	NadC	Nicotinate-nucleotide pyrophosphorylase
LN02_02212 | LN02Chr02 :945439-946089(+) | 130	CDD:224869	25.676	74	51	1	1	70	6	79	5.08E-13	57.3	COG1958	LSM1	Small nuclear ribonucleoprotein (snRNP) homolog
LN02_02276 | LN02Chr02 :1198075-1199438(-) | 297	CDD:223959	33.068	251	161	5	18	265	3	249	6.16E-44	147	COG1028	FabG	Dehydrogenases with different specificities (related to short-chain alcohol dehydrogenases)
LN02_02660 | LN02Chr02 :2590173-2590859(+) | 138	CDD:224020	25.749	167	93	5	2	137	8	174	3.85E-30	104	COG1095	RPB7	DNA-directed RNA polymerase, subunit E'
LN02_02788 | LN02Chr02 :3002664-3004486(+) | 498	CDD:223619	40.94	149	81	5	353	498	61	205	2.07E-41	144	COG0545	FkpA	FKBP-type peptidyl-prolyl cis-trans isomerases 1
LN02_02852 | LN02Chr02 :3214987-3218401(+) | 944	CDD:223732	23.693	574	389	13	159	726	21	551	1.17E-76	258	COG0659	SUL1	Sulfate permease and related transporters (MFS superfamily)
LN02_02852 | LN02Chr02 :3214987-3218401(+) | 944	CDD:223736	23.448	145	101	3	803	943	1	139	3.24E-14	69.9	COG0664	Crp	cAMP-binding proteins - catabolite gene activator and regulatory subunit of cAMP-dependent protein kinases
LN02_03044 | LN02Chr02 :3903920-3905551(+) | 543	CDD:223587	36.301	438	254	11	112	542	29	448	3.24E-120	361	COG0513	SrmB	Superfamily II DNA and RNA helicases
LN02_03364 | LN02Chr02 :4996692-4997750(+) | 352	CDD:223607	40	355	192	8	5	351	1	342	6.19E-119	344	COG0533	QRI7	Metal-dependent proteases with possible chaperone activity
LN02_03428 | LN02Chr02 :5175614-5176886(-) | 401	CDD:223131	19.608	153	102	4	144	295	67	199	8.91E-09	53.4	COG0053	MMT1	Predicted Co/Zn/Cd cation transporters
LN02_03428 | LN02Chr02 :5175614-5176886(-) | 401	CDD:224151	19.938	321	214	8	82	399	15	295	8.30E-08	50.4	COG1230	CzcD	Co/Zn/Cd efflux system component
LN02_03492 | LN02Chr02 :5773744-5775130(+) | 438	CDD:225201	22.581	279	182	11	71	344	62	311	2.52E-07	49.7	COG2319	COG2319	FOG: WD40 repeat
LN02_03684 | LN02Chr03 :301694-302116(+) | 122	CDD:223589	31.624	117	70	4	3	110	30	145	7.22E-12	57.8	COG0515	SPS1	Serine/threonine protein kinase
LN02_03940 | LN02Chr03 :1197638-1199054(-) | 428	CDD:223318	29.064	406	194	13	15	417	3	317	1.15E-95	287	COG0240	GpsA	Glycerol-3-phosphate dehydrogenase
LN02_04132 | LN02Chr03 :2768473-2770140(-) | 435	CDD:224420	40.831	409	239	1	12	420	6	411	1.04E-173	489	COG1503	eRF1	Peptide chain release factor 1 (eRF1)
LN02_04196 | LN02Chr03 :3309413-3311131(+) | 480	CDD:223208	45.151	299	132	8	53	350	4	271	3.53E-96	288	COG0130	TruB	Pseudouridine synthase
LN02_04260 | LN02Chr03 :3585444-3586500(+) | 264	CDD:223777	20.276	217	153	6	11	213	8	218	0.001	36.7	COG0705	COG0705	Membrane associated serine protease
LN02_04324 | LN02Chr03 :3794133-3795391(-) | 303	CDD:223302	35.764	288	159	9	35	299	1	285	9.51E-73	223	COG0224	AtpG	F0F1-type ATP synthase, gamma subunit
LN02_04452 | LN02Chr03 :4207888-4209925(-) | 648	CDD:224720	14.286	224	174	5	424	645	182	389	6.62E-04	39.8	COG1807	ArnT	4-amino-4-deoxy-L-arabinose transferase and related glycosyltransferases of PMT family
LN02_04516 | LN02Chr03 :4414025-4416083(+) | 574	CDD:224957	47.118	399	191	10	5	400	6	387	3.32E-154	445	COG2046	MET3	ATP sulfurylase (sulfate adenylyltransferase)

LN02_04516｜LN02Chr03:4414025-4416083(+)｜574	CDD:223603	44.792	192	102	3	380	571	8	195	1.54E-46	159	COG0529	CysC	Adenylylsulfate kinase and related kinases
LN02_04580｜LN02Chr03:4660389-4663973(-)｜1133	CDD:227535	38.028	284	165	5	246	520	175	456	4.63E-70	240	COG5210	COG5210	GTPase-activating protein
LN02_04900｜LN02Chr03:5905215-5909152(+)｜1145	CDD:223589	30.508	295	173	7	819	1086	1	290	2.84E-53	188	COG0515	SPS1	Serine/threonine protein kinase
LN02_05092｜LN02Chr04:584387-586401(+)｜614	CDD:223733	33.742	326	194	7	149	468	55	364	1.73E-82	265	COG0661	AarF	Predicted unusual protein kinase
LN02_05156｜LN02Chr04:781501-782658(+)｜184	CDD:224641	44.444	126	65	3	14	139	2	122	6.18E-43	137	COG1727	RPL18A	Ribosomal protein L18E
LN02_05604｜LN02Chr04:2612342-2615945(+)｜1002	CDD:225201	20	390	252	12	6	358	47	413	2.09E-26	110	COG2319	COG2319	FOG: WD40 repeat
LN02_05668｜LN02Chr04:2798991-2801252(+)｜530	CDD:224152	18.962	443	316	13	60	485	32	448	7.10E-27	110	COG1231	COG1231	Monoamine oxidase
LN02_05732｜LN02Chr04:3022938-3023456(-)｜172	CDD:226333	33.735	166	105	1	1	161	25	190	1.42E-37	125	COG3812	COG3812	Uncharacterized protein conserved in bacteria
LN02_05860｜LN02Chr04:3438456-3440373(+)｜528	CDD:224826	36.943	471	222	9	58	526	18	415	1.70E-93	288	COG1914	MntH	Mn2+ and Fe2+ transporters of the NRAMP family
LN02_06180｜LN02Chr04:4582569-4585500(+)｜836	CDD:223589	31.884	276	157	9	562	809	7	279	2.09E-47	170	COG0515	SPS1	Serine/threonine protein kinase
LN02_06308｜LN02Chr04:4986731-4988339(-)｜324	CDD:223254	36.25	320	119	8	5	320	1	239	1.16E-81	244	COG0176	MipB	Transaldolase
LN02_06372｜LN02Chr04:5179835-5180932(-)｜190	CDD:227455	25.862	174	112	5	9	179	1	160	1.48E-41	135	COG5126	FRQ1	Ca2+-binding protein (EF-Hand superfamily)
LN02_06692｜LN02Chr05:436077-440561(-)｜1416	CDD:226978	43.587	803	402	15	629	1405	4	781	0	809	COG4631	XdhB	Xanthine dehydrogenase, molybdopterin-binding subunit B
LN02_06692｜LN02Chr05:436077-440561(-)｜1416	CDD:226977	31.478	575	282	13	29	597	7	475	3.61E-97	318	COG4630	XdhA	Xanthine dehydrogenase, iron-sulfur cluster and FAD-binding subunit A
LN02_06948｜LN02Chr05:1765394-1766922(+)｜348	CDD:223991	36.872	358	207	10	1	348	2	350	6.76E-86	260	COG1063	Tdh	Threonine dehydrogenase and related Zn-dependent dehydrogenases
LN02_07012｜LN02Chr05:1956550-1957869(-)｜194	CDD:224025	31.414	191	124	5	6	189	5	195	6.05E-43	140	COG1100	COG1100	GTPase SAR1 and related small G proteins
LN02_07140｜LN02Chr05:2705730-2707304(-)｜434	CDD:227590	47.135	384	161	3	1	342	114	497	1.08E-133	391	COG5265	ATM1	ABC-type transport system involved in Fe-S cluster assembly, permease and ATPase components
LN02_07268｜LN02Chr05:3149507-3150526(+)｜291	CDD:227428	26.154	195	115	5	5	188	2	178	4.31E-24	94.3	COG5097	MED6	RNA polymerase II transcriptional regulation mediator
LN02_07524｜LN02Chr05:4061073-4061557(-)｜96	CDD:223478	42.593	54	30	1	4	56	3	56	3.23E-14	58.4	COG0401	COG0401	Uncharacterized homolog of Blt101
LN02_07716｜LN02Chr05:4995557-4997432(+)｜568	CDD:224281	37.969	453	251	9	109	559	11	435	5.66E-144	420	COG1362	LAP4	Aspartyl aminopeptidase
LN02_07780｜LN02Chr05:5215014-5217188(-)｜642	CDD:225490	21.158	501	285	17	87	560	66	483	5.71E-29	117	COG2939	COG2939	Carboxypeptidase C (cathepsin A)

LN02_08036|LN02Chr06:571613-572671(-)|352	CDD:223730	35.494	324	179	11	31	349	13	311	2.36E-51	170	COG0657	Aes	Esterase/lipase
LN02_08100|LN02Chr06:1243224-1246504(+)|1023	CDD:225789	29.989	877	515	21	52	907	9	807	7.97E-140	436	COG3250	LacZ	Beta-galactosidase/beta-glucuronidase
LN02_08612|LN02Chr06:3149649-3150538(-)|272	CDD:225670	19.524	210	115	10	55	255	10	174	3.10E-05	41.3	COG3128	PiuC	Uncharacterized iron-regulated protein
LN02_08804|LN02Chr07:400175-403213(-)|871	CDD:223457	45.232	409	195	6	143	543	88	475	1.13E-152	454	COG0380	OtsA	Trehalose-6-phosphate synthase
LN02_08804|LN02Chr07:400175-403213(-)|871	CDD:224789	36.735	294	156	6	558	849	1	266	5.22E-80	256	COG1877	OtsB	Trehalose-6-phosphatase
LN02_09188|LN02Chr07:1686241-1688348(+)|458	CDD:224145	54.566	449	194	5	1	448	3	442	0	639	COG1224	TIP49	DNA helicase TIP49, TBP-interacting protein
LN02_09252|LN02Chr07:1946676-1949500(+)|732	CDD:225371	19.33	388	251	13	195	580	18	345	6.84E-17	80.4	COG2814	AraJ	Arabinose efflux permease
LN02_00037|LN02Chr01:297088-299673(-)|629	CDD:223553	15.407	344	268	5	112	451	1	325	5.28E-04	39.7	COG0477	ProP	Permeases of the major facilitator superfamily
LN02_00101|LN02Chr01:491228-491933(+)|203	CDD:224927	31.606	193	99	4	11	203	2	161	2.88E-33	114	COG2016	COG2016	Predicted RNA-binding protein (contains PUA domain)
LN02_00357|LN02Chr01:1891454-1894187(-)|559	CDD:224864	32.99	485	299	10	37	516	28	491	1.77E-110	336	COG1953	FUI1	Cytosine/uracil/thiamine/allantoin permeases
LN02_00485|LN02Chr01:2263214-2265144(+)|514	CDD:224871	26.355	406	265	10	114	507	5	388	7.15E-57	191	COG1960	CaiA	Acyl-CoA dehydrogenases
LN02_00485|LN02Chr01:2263214-2265144(+)|514	CDD:227599	28.44	109	76	2	2	109	46	153	1.52E-16	74.5	COG5274	CYB5	Cytochrome b involved in lipid metabolism
LN02_00549|LN02Chr01:2438887-2440126(+)|271	CDD:226674	46.124	258	126	5	6	262	1	246	2.11E-96	280	COG4221	COG4221	Short-chain alcohol dehydrogenase of unknown specificity
LN02_00677|LN02Chr01:2999506-3001134(-)|525	CDD:225371	19.036	415	271	8	73	486	37	387	2.21E-15	75	COG2814	AraJ	Arabinose efflux permease
LN02_00869|LN02Chr01:3638815-3639585(+)|232	CDD:223589	21.374	131	86	4	88	203	46	174	1.79E-08	51.3	COG0515	SPS1	Serine/threonine protein kinase
LN02_00997|LN02Chr01:4046856-4047721(+)|187	CDD:227533	29.921	127	81	3	20	140	110	234	3.57E-14	66.2	COG5208	HAP5	CCAAT-binding factor, subunit C
LN02_01061|LN02Chr01:4275428-4276495(+)|287	CDD:227579	27.039	233	139	5	3	220	2	218	3.04E-37	129	COG5254	ARV1	Predicted membrane protein
LN02_01253|LN02Chr01:5064068-5066152(+)|586	CDD:225371	17.69	407	250	12	86	480	50	383	4.96E-07	49.1	COG2814	AraJ	Arabinose efflux permease
LN02_01317|LN02Chr01:5239473-5242828(-)|1016	CDD:223343	21.875	352	233	11	59	393	16	342	2.18E-26	108	COG0265	DegQ	Trypsin-like serine proteases, typically periplasmic, contain C-terminal PDZ domain
LN02_01381|LN02Chr01:5515967-5517264(+)|403	CDD:223584	22.667	300	159	14	79	371	20	253	4.81E-14	69	COG0510	ycfN	Thiamine kinase and related kinases
LN02_01701|LN02Chr01:6802403-6803565(-)|332	CDD:225264	38.095	63	39	0	270	332	95	157	5.14E-04	37.8	COG2391	COG2391	Predicted transporter component

LN02_01957丨LN02Chr02:104796-106373(-)丨525	CDD:225182	26.887	212	140	5	50	251	78	284	2.88E-16	77.5	COG2273	SKN1	Beta-glucanase/Beta-glucan synthetase
LN02_02021丨LN02Chr02:351437-353725(+)丨686	CDD:223808	20.782	409	271	17	127	516	19	393	5.64E-32	127	COG0737	UshA	5'-nucleotidase/2',3'-cyclic phosphodiesterase and related esterases
LN02_02213丨LN02Chr02:946641-947429(-)丨262	CDD:225443	19.679	249	145	12	13	261	87	280	1.91E-17	77	COG2890	HemK	Methylase of polypeptide chain release factors
LN02_02597丨LN02Chr02:2362413-2363586(-)丨348	CDD:223544	29.474	285	174	8	64	340	2	267	9.89E-95	280	COG0468	RecA	RecA/RadA recombinase
LN02_02661丨LN02Chr02:2592010-2593116(+)丨268	CDD:226356	38.281	256	152	3	4	258	5	255	1.52E-69	212	COG3836	HpcH	2,4-dihydroxyhept-2-ene-1,7-dioic acid aldolase
LN02_02725丨LN02Chr02:2782807-2783735(-)丨195	CDD:223102	37.349	83	45	2	101	183	27	102	5.45E-19	75.4	COG0023	SUI1	Translation initiation factor 1 (eIF-1/SUI1) and related proteins
LN02_02789丨LN02Chr02:3005447-3007255(+)丨549	CDD:223589	39.241	158	83	3	323	476	1	149	8.29E-28	112	COG0515	SPS1	Serine/threonine protein kinase
LN02_02917丨LN02Chr02:3461588-3464107(-)丨785	CDD:223120	30.841	321	151	9	377	689	15	272	1.63E-51	179	COG0042	COG0042	tRNA-dihydrouridine synthase
LN02_03237丨LN02Chr02:4589414-4591019(+)丨399	CDD:227916	18.919	296	183	13	105	392	72	318	1.15E-14	71.2	COG5629	COG5629	Predicted metal-binding protein
LN02_03429丨LN02Chr02:5178353-5180548(-)丨606	CDD:223589	23.429	175	110	6	362	519	61	228	1.64E-05	44.3	COG0515	SPS1	Serine/threonine protein kinase
LN02_03493丨LN02Chr02:5776264-5777301(-)丨345	CDD:224371	21.93	228	107	8	117	318	8	190	5.21E-18	81.1	COG1454	EutG	Alcohol dehydrogenase, class IV
LN02_03557丨LN02Chr02:6165315-6166973(-)丨552	CDD:224650	26.359	368	227	10	39	393	8	344	2.24E-54	184	COG1736	DPH2	Diphthamide synthase subunit DPH2
LN02_03621丨LN02Chr02:6720860-6723125(+)丨704	CDD:226256	40.476	672	372	15	13	679	5	653	0	682	COG3733	TynA	Cu2+-containing amine oxidase
LN02_03877丨LN02Chr03:990613-991256(-)丨112	CDD:223090	40.625	96	57	0	14	109	5	100	2.00E-29	99.3	COG0011	COG0011	Uncharacterized conserved protein
LN02_04325丨LN02Chr03:3795926-3799297(+)丨911	CDD:226099	22.113	407	242	16	258	660	19	354	2.95E-69	231	COG3569	COG3569	Topoisomerase IB
LN02_04389丨LN02Chr03:4024903-4025870(-)丨287	CDD:223637	32.275	189	110	5	95	279	3	177	4.34E-44	145	COG0563	Adk	Adenylate kinase and related kinases
LN02_04581丨LN02Chr03:4667568-4669171(+)丨396	CDD:223589	35.082	305	160	8	60	343	1	288	7.70E-57	188	COG0515	SPS1	Serine/threonine protein kinase
LN02_04773丨LN02Chr03:5366253-5367912(+)丨468	CDD:227475	49.02	357	138	8	91	440	18	337	2.17E-107	319	COG5146	PanK	Pantothenate kinase, acetyl-CoA regulated
LN02_04901丨LN02Chr03:5910012-5911459(-)丨448	CDD:224871	41.388	389	218	7	69	448	5	392	6.41E-117	345	COG1960	CaiA	Acyl-CoA dehydrogenases
LN02_05093丨LN02Chr04:587317-589242(-)丨514	CDD:225035	24.888	446	251	15	56	483	14	393	2.30E-32	125	COG2124	CypX	Cytochrome P450
LN02_05157丨LN02Chr04:783062-784574(-)丨298	CDD:223370	36.22	254	111	3	35	288	1	203	5.16E-66	202	COG0293	FtsJ	23S rRNA methylase
LN02_05285丨LN02Chr04:1203395-1204554(-)丨286	CDD:226200	20.213	188	114	8	62	223	79	256	1.19E-06	46.1	COG3675	COG3675	Predicted lipase

LN02_05477lLN02Chr04:1968999-1969781(-)l180	CDD:224117	24.286	140	102	1	45	180	708	847	2.63E-04	37.8	COG1196	Smc	Chromosome segregation ATPases
LN02_05733lLN02Chr04:3024421-3025677(-)l418	CDD:225213	23.49	149	95	7	245	383	178	317	3.10E-05	42.7	COG2334	COG2334	Putative homoserine kinase type II (protein kinase fold)
LN02_05989lLN02Chr04:3932682-3936481(-)l1247	CDD:225127	36.647	865	374	17	296	1159	3	694	0	672	COG2217	ZntA	Cation transport ATPase
LN02_05989lLN02Chr04:3932682-3936481(-)l1247	CDD:225127	32.857	70	45	2	27	95	1	69	3.70E-08	54.5	COG2217	ZntA	Cation transport ATPase
LN02_05989lLN02Chr04:3932682-3936481(-)l1247	CDD:225328	39.706	68	41	0	215	282	4	71	7.11E-14	64.7	COG2608	CopZ	Copper chaperone
LN02_05989lLN02Chr04:3932682-3936481(-)l1247	CDD:225328	32.812	64	43	0	127	190	8	71	3.80E-11	57	COG2608	CopZ	Copper chaperone
LN02_06181lLN02Chr04:4587677-4588771(-)l364	CDD:223959	34.387	253	155	6	115	362	5	251	6.39E-65	203	COG1028	FabG	Dehydrogenases with different specificities (related to short-chain alcohol dehydrogenases)
LN02_06309lLN02Chr04:4989037-4991688(-)l883	CDD:224442	27.922	154	95	5	307	455	32	174	4.48E-19	83.4	COG1525	COG1525	Micrococcal nuclease (thermonuclease) homologs
LN02_06309lLN02Chr04:4989037-4991688(-)l883	CDD:224442	28.333	120	78	6	497	612	54	169	4.54E-08	51.4	COG1525	COG1525	Micrococcal nuclease (thermonuclease) homologs
LN02_06309lLN02Chr04:4989037-4991688(-)l883	CDD:224442	26.429	140	88	6	165	302	40	166	2.30E-06	46	COG1525	COG1525	Micrococcal nuclease (thermonuclease) homologs
LN02_06309lLN02Chr04:4989037-4991688(-)l883	CDD:224442	24.46	139	87	5	9	142	46	171	4.74E-05	42.2	COG1525	COG1525	Micrococcal nuclease (thermonuclease) homologs
LN02_06565lLN02Chr04:5871370-5873083(+)l486	CDD:227170	21.405	299	188	13	71	357	44	307	1.69E-07	50.4	COG4833	COG4833	Predicted glycosyl hydrolase
LN02_06693lLN02Chr05:442158-444018(-)l459	CDD:223513	31.442	423	250	10	40	457	4	391	5.87E-113	335	COG0436	COG0436	Aspartate/tyrosine/aromatic aminotransferase
LN02_06949lLN02Chr05:1766982-1767410(-)l142	CDD:226683	18.954	153	80	9	2	117	576	721	6.74E-04	35.8	COG4230	COG4230	Delta 1-pyrroline-5-carboxylate dehydrogenase
LN02_07141lLN02Chr05:2707378-2711435(-)l518	CDD:227590	37.037	81	49	2	433	511	1	81	1.26E-16	79.3	COG5265	ATM1	ABC-type transport system involved in Fe-S cluster assembly, permease and ATPase components
LN02_07141lLN02Chr05:2707378-2711435(-)l518	CDD:224055	23.171	164	103	4	347	509	7	148	2.04E-10	60.1	COG1132	MdlB	ABC-type multidrug transport system, ATPase and permease components
LN02_07269lLN02Chr05:3150840-3152705(-)l490	CDD:223944	31.081	444	266	8	5	414	34	471	1.10E-99	305	COG1012	PutA	NAD-dependent aldehyde dehydrogenases
LN02_07333lLN02Chr05:3367873-3370664(-)l840	CDD:225514	27.273	253	177	5	392	642	3	250	6.82E-53	181	COG2966	COG2966	Uncharacterized conserved protein
LN02_07333lLN02Chr05:3367873-3370664(-)l840	CDD:226137	22.436	156	109	3	666	821	3	146	4.58E-22	90.8	COG3610	COG3610	Uncharacterized conserved protein
LN02_07397lLN02Chr05:3615158-3617026(-)l622	CDD:225362	35.965	114	65	4	361	467	10	122	3.28E-22	92.5	COG2802	COG2802	Uncharacterized protein, similar to the N-terminal domain of Lon protease
LN02_07397lLN02Chr05:3615158-3617026(-)l622	CDD:227861	33.333	60	35	2	255	312	203	259	5.51E-07	48.4	COG5574	PEX10	RING-finger-containing E3 ubiquitin ligase

LN02_07461∣LN02Chr05:3856841-3858839(-)∣617	CDD:223533	23.348	227	162	4	320	540	62	282	6.35E-07	48.3	COG0457	NrfG	FOG: TPR repeat
LN02_07653∣LN02Chr05:4739478-4741897(-)∣788	CDD:223803	34.516	310	174	9	425	731	1	284	1.68E-84	268	COG0731	COG0731	Fe-S oxidoreductases
LN02_07717∣LN02Chr05:4997942-5001390(-)∣979	CDD:227696	35.735	347	209	8	508	845	36	377	3.35E-77	255	COG5409	COG5409	EXS domain-containing protein
LN02_07717∣LN02Chr05:4997942-5001390(-)∣979	CDD:227695	19.758	248	161	8	200	418	55	293	2.00E-07	51	COG5408	COG5408	SPX domain-containing protein
LN02_07781∣LN02Chr05:5217892-5219714(+)∣579	CDD:223600	35.714	70	42	2	196	263	36	104	7.82E-11	57.2	COG0526	TrxA	Thiol-disulfide isomerase and thioredoxins
LN02_07909∣LN02Chr05:5658884-5661421(+)∣845	CDD:223481	36.207	406	225	7	445	845	3	379	2.80E-92	293	COG0404	GcvT	Glycine cleavage system T protein (aminomethyltransferase)
LN02_07909∣LN02Chr05:5658884-5661421(+)∣845	CDD:223737	27.184	412	255	12	13	410	1	381	1.96E-40	150	COG0665	DadA	Glycine/D-amino acid oxidases (deaminating)
LN02_07973∣LN02Chr05:5840815-5841797(+)∣287	CDD:224025	24	200	138	4	96	281	2	201	1.34E-31	114	COG1100	COG1100	GTPase SAR1 and related small G proteins
LN02_08101∣LN02Chr06:1247311-1251059(+)∣970	CDD:223697	19.876	483	279	17	492	964	16	400	5.25E-42	156	COG0624	ArgE	Acetylornithine deacetylase/Succinyl-diaminopimelate desuccinylase and related deacylases
LN02_08101∣LN02Chr06:1247311-1251059(+)∣970	CDD:225201	21.463	410	225	12	70	474	127	444	4.35E-18	85.5	COG2319	COG2319	FOG: WD40 repeat
LN02_08357∣LN02Chr06:2218446-2220087(-)∣402	CDD:224594	25.641	390	237	13	16	398	47	390	1.25E-35	132	COG1680	AmpC	Beta-lactamase class C and other penicillin binding proteins
LN02_08421∣LN02Chr06:2516903-2517779(-)∣275	CDD:223761	28.302	159	94	4	35	191	20	160	2.63E-13	64.6	COG0689	Rph	RNase PH
LN02_08741∣LN02Chr07:209840-211786(-)∣648	CDD:223951	31.126	453	279	10	22	462	210	641	9.83E-79	260	COG1020	EntF	Non-ribosomal peptide synthetase modules and related proteins
LN02_08741∣LN02Chr07:209840-211786(-)∣648	CDD:223314	28	75	52	2	574	646	1	75	5.61E-04	36.1	COG0236	AcpP	Acyl carrier protein
LN02_08997∣LN02Chr07:1079056-1079897(-)∣204	CDD:223728	34.762	210	127	7	1	204	2	207	1.38E-46	149	COG0655	WrbA	Multimeric flavodoxin WrbA
LN02_09189∣LN02Chr07:1693748-1697839(+)∣1319	CDD:227569	41.026	78	45	1	7	84	7	83	1.93E-15	78.2	COG5244	NIP100	Dynactin complex subunit involved in mitotic spindle partitioning in anaphase B
LN02_09189∣LN02Chr07:1693748-1697839(+)∣1319	CDD:224117	22.756	312	216	4	330	640	221	508	4.02E-14	74.4	COG1196	Smc	Chromosome segregation ATPases
LN02_09189∣LN02Chr07:1693748-1697839(+)∣1319	CDD:224117	17.829	774	525	24	480	1228	216	903	2.91E-07	52	COG1196	Smc	Chromosome segregation ATPases
LN02_00422∣LN02Chr01:2077694-2078914(-)∣357	CDD:223560	42.647	68	37	2	76	141	5	72	3.00E-16	76.1	COG0484	DnaJ	DnaJ-class molecular chaperone with C-terminal Zn finger domain
LN02_00422∣LN02Chr01:2077694-2078914(-)∣357	CDD:227694	24.022	179	107	7	30	191	49	215	1.21E-09	56.5	COG5407	SEC63	Preprotein translocase subunit Sec63

LN02_00486 ǀ LN02Chr01:2265900-2266943(-) ǀ 183	CDD:227414	31.724	145	80	5	14	155	62	190	8.67E-16	69.1	COG5082	AIR1	Arginine methyltransferase-interacting protein, contains RING Zn-finger
LN02_00550 ǀ LN02Chr01:2440932-2442560(-) ǀ 494	CDD:223538	30.675	489	162	7	1	489	2	313	1.13E-106	317	COG0462	PrsA	Phosphoribosylpyrophosphate synthetase
LN02_00678 ǀ LN02Chr01:3003473-3004443(-) ǀ 261	CDD:224480	26.638	229	145	9	23	251	2	207	5.81E-26	98.5	COG1564	THI80	Thiamine pyrophosphokinase
LN02_00742 ǀ LN02Chr01:3217685-3219049(-) ǀ 377	CDD:227566	31.959	194	125	4	77	269	36	223	5.19E-50	165	COG5241	RAD10	Nucleotide excision repair endonuclease NEF1, RAD10 subunit
LN02_00934 ǀ LN02Chr01:3820907-3821357(-) ǀ 106	CDD:223767	34.177	79	51	1	17	95	3	80	5.71E-25	87	COG0695	GrxC	Glutaredoxin and related proteins
LN02_01190 ǀ LN02Chr01:4812002-4817095(+) ǀ 1620	CDD:227544	17.953	1671	1096	60	31	1607	33	1522	9.52E-67	246	COG5219	COG5219	Uncharacterized conserved protein, contains RING Zn-finger
LN02_01638 ǀ LN02Chr01:6548249-6549867(-) ǀ 427	CDD:226297	17.687	294	201	12	101	390	82	338	4.19E-13	67.1	COG3774	OCH1	Mannosyltransferase OCH1 and related enzymes
LN02_01702 ǀ LN02Chr01:6805172-6806372(+) ǀ 360	CDD:223677	27.586	348	200	10	21	352	15	326	7.63E-44	151	COG0604	Qor	NADPH:quinone reductase and related Zn-dependent oxidoreductases
LN02_01766 ǀ LN02Chr01:6961940-6963396(-) ǀ 418	CDD:227408	34.831	356	214	7	54	400	9	355	5.73E-69	220	COG5076	COG5076	Transcription factor involved in chromatin remodeling, contains bromodomain
LN02_01830 ǀ LN02Chr01:7161834-7162616(+) ǀ 260	CDD:224254	31.416	226	120	6	1	219	2	199	1.06E-36	126	COG1335	PncA	Amidases related to nicotinamidase
LN02_01958 ǀ LN02Chr02:108766-109753(-) ǀ 213	CDD:224025	26.794	209	137	4	7	211	1	197	8.75E-42	138	COG1100	COG1100	GTPase SAR1 and related small G proteins
LN02_02086 ǀ LN02Chr02:569477-570973(-) ǀ 498	CDD:226038	22.713	317	197	16	26	313	15	312	1.94E-13	69.4	COG3507	XynB	Beta-xylosidase
LN02_02150 ǀ LN02Chr02:743803-745578(+) ǀ 591	CDD:223777	34.752	141	76	4	350	478	12	148	5.46E-16	74.4	COG0705	COG0705	Membrane associated serine protease
LN02_02598 ǀ LN02Chr02:2364045-2367770(-) ǀ 772	CDD:223149	23.832	214	86	5	561	772	8	146	1.00E-16	74.7	COG0071	IbpA	Molecular chaperone (small heat shock protein)
LN02_02726 ǀ LN02Chr02:2785490-2788282(+) ǀ 930	CDD:225136	23.188	138	69	8	637	760	54	168	3.22E-06	46.5	COG2226	UbiE	Methylase involved in ubiquinone/menaquinone biosynthesis
LN02_02790 ǀ LN02Chr02:3007313-3008229(+) ǀ 285	CDD:223589	18.788	165	130	3	1	164	175	336	2.17E-11	60.5	COG0515	SPS1	Serine/threonine protein kinase
LN02_02854 ǀ LN02Chr02:3226479-3229534(+) ǀ 991	CDD:227392	32.186	494	267	11	214	699	9	442	1.38E-80	270	COG5059	KIP1	Kinesin-like protein
LN02_02982 ǀ LN02Chr02:3675453-3676384(-) ǀ 274	CDD:227366	34.857	175	94	6	1	167	1	163	4.95E-33	117	COG5033	TFG3	Transcription initiation factor IIF, auxiliary subunit
LN02_03238 ǀ LN02Chr02:4591355-4594948(+) ǀ 1145	CDD:223627	28.659	164	109	3	884	1039	696	859	9.90E-14	72.8	COG0553	HepA	Superfamily II DNA/RNA helicases, SNF2 family
LN02_04006 ǀ LN02Chr03:1406249-1409038(-) ǀ 899	CDD:224025	17.978	89	61	3	270	348	7	93	3.28E-04	39.9	COG1100	COG1100	GTPase SAR1 and related small G proteins
LN02_04070 ǀ LN02Chr03:2008640-2009268(+) ǀ 190	CDD:223532	24.862	181	111	5	1	170	11	177	1.86E-27	99.3	COG0456	RimI	Acetyltransferases

LN02_04198∣LN02Chr03:3314571-3318255(-)∣1205	CDD:227411	30.909	385	235	12	30	410	63	420	9.31E-68	238	COG5079	SAC3	Nuclear protein export factor
LN02_04198∣LN02Chr03:3314571-3318255(-)∣1205	CDD:227938	16.8	250	181	7	544	787	150	378	0.001	39.9	COG5651	COG5651	PPE-repeat proteins
LN02_04326∣LN02Chr03:3800380-3801633(-)∣387	CDD:225182	28.378	148	92	6	103	242	114	255	1.22E-12	65.6	COG2273	SKN1	Beta-glucanase/Beta-glucan synthetase
LN02_04582∣LN02Chr03:4669901-4671645(-)∣550	CDD:227698	27.128	188	99	8	281	461	181	337	1.02E-19	89.1	COG5411	COG5411	Phosphatidylinositol 5-phosphate phosphatase
LN02_04646∣LN02Chr03:4907349-4910996(+)∣900	CDD:223959	37.302	252	131	8	311	538	2	250	7.89E-52	179	COG1028	FabG	Dehydrogenases with different specificities (related to short-chain alcohol dehydrogenases)
LN02_04646∣LN02Chr03:4907349-4910996(+)∣900	CDD:223959	32.51	243	134	7	23	245	19	251	5.72E-39	142	COG1028	FabG	Dehydrogenases with different specificities (related to short-chain alcohol dehydrogenases)
LN02_04646∣LN02Chr03:4907349-4910996(+)∣900	CDD:224941	22.481	129	85	3	777	898	22	142	5.92E-14	67.5	COG2030	MaoC	Acyl dehydratase
LN02_04902∣LN02Chr03:5912284-5912960(+)∣184	CDD:223126	56.693	127	48	3	55	179	8	129	1.81E-49	153	COG0048	RpsL	Ribosomal protein S12
LN02_05030∣LN02Chr04:392119-393951(+)∣610	CDD:227381	30.12	83	52	3	235	311	273	355	3.33E-04	40.4	COG5048	COG5048	FOG: Zn-finger
LN02_05670∣LN02Chr04:2803632-2804769(+)∣352	CDD:225921	27.941	272	154	11	58	323	28	263	4.27E-37	132	COG3386	COG3386	Gluconolactonase
LN02_05734∣LN02Chr04:3026228-3028051(+)∣570	CDD:223096	37.182	433	254	8	143	570	16	435	7.53E-149	432	COG0017	AsnS	Aspartyl/asparaginyl-tRNA synthetases
LN02_05926∣LN02Chr04:3638408-3641543(+)∣988	CDD:225201	17.355	242	163	9	34	269	196	406	1.04E-04	42.8	COG2319	COG2319	FOG: WD40 repeat
LN02_06246∣LN02Chr04:4777423-4780229(-)∣881	CDD:224117	21.765	170	123	4	326	495	747	906	6.10E-07	50.5	COG1196	Smc	Chromosome segregation ATPases
LN02_06246∣LN02Chr04:4777423-4780229(-)∣881	CDD:226809	17.06	381	262	9	329	696	143	482	2.26E-05	45	COG4372	COG4372	Uncharacterized protein conserved in bacteria with the myosin-like domain
LN02_06310∣LN02Chr04:4992957-4993838(-)∣198	CDD:223094	29.114	158	85	3	13	169	303	434	1.53E-28	107	COG0015	PurB	Adenylosuccinate lyase
LN02_06438∣LN02Chr04:5391472-5392187(+)∣177	CDD:223532	22.222	108	77	4	72	174	57	162	4.68E-10	53.1	COG0456	RimI	Acetyltransferases
LN02_06502∣LN02Chr04:5660622-5663604(-)∣940	CDD:227540	18.401	538	341	20	25	542	18	477	2.47E-15	77.7	COG5215	KAP95	Karyopherin (importin) beta
LN02_06694∣LN02Chr05:444702-445729(-)∣309	CDD:223589	22.901	262	166	9	47	276	15	272	1.15E-17	79.8	COG0515	SPS1	Serine/threonine protein kinase
LN02_06822∣LN02Chr05:916851-919999(-)∣1014	CDD:223556	22.593	1018	429	30	1	995	16	697	1.56E-128	403	COG0480	FusA	Translation elongation factors (GTPases)
LN02_06886∣LN02Chr05:1163429-1164618(-)∣348	CDD:225546	25.391	256	180	4	86	333	19	271	3.09E-41	142	COG3000	ERG3	Sterol desaturase
LN02_07078∣LN02Chr05:2483206-2491324(-)∣2108	CDD:227409	28.76	379	234	14	1140	1508	192	544	1.14E-52	200	COG5077	COG5077	Ubiquitin carboxyl-terminal hydrolase

LN02_07206|LN02Chr05:2978826-2980316(+)|496	CDD:225201	21.39	374	199	12	73	420	60	364	1.14E-10	60.5	COG2319	COG2319	FOG: WD40 repeat
LN02_07334|LN02Chr05:3372442-3376229(+)|1183	CDD:223163	23.793	1139	655	30	23	1070	11	1027	0	745	COG0085	RpoB	DNA-directed RNA polymerase, beta subunit/140 kD subunit
LN02_07462|LN02Chr05:3859318-3860143(+)|229	CDD:223209	44.954	218	94	3	10	227	4	195	3.60E-96	276	COG0131	HisB	Imidazoleglycerol-phosphate dehydratase
LN02_07654|LN02Chr05:4742508-4745653(-)|898	CDD:223627	28.931	636	301	15	285	884	332	852	2.78E-75	261	COG0553	HepA	Superfamily II DNA/RNA helicases, SNF2 family
LN02_07846|LN02Chr05:5480630-5482320(+)|487	CDD:223778	20.264	227	163	8	165	387	89	301	2.34E-24	100	COG0706	YidC	Preprotein translocase subunit YidC
LN02_07910|LN02Chr05:5662255-5663281(+)|276	CDD:223955	33.333	252	160	6	21	271	13	257	5.82E-46	152	COG1024	CaiD	Enoyl-CoA hydratase/carnithine racemase
LN02_07974|LN02Chr05:5843756-5844365(+)|166	CDD:223583	42.52	127	66	4	38	164	9	128	1.83E-42	135	COG0509	GcvH	Glycine cleavage system H protein (lipoate-binding)
LN02_08038|LN02Chr06:577371-579745(+)|750	CDD:223983	24.832	149	109	2	127	275	57	202	6.60E-15	74.6	COG1055	ArsB	Na+/H+ antiporter NhaD and related arsenite permeases
LN02_08038|LN02Chr06:577371-579745(+)|750	CDD:223983	20.625	160	109	6	578	734	277	421	1.80E-06	48	COG1055	ArsB	Na+/H+ antiporter NhaD and related arsenite permeases
LN02_08870|LN02Chr07:643019-644743(-)|469	CDD:225201	22.5	280	205	8	197	465	119	397	1.24E-17	82.1	COG2319	COG2319	FOG: WD40 repeat
LN02_09126|LN02Chr07:1511787-1513633(+)|503	CDD:223225	37.876	499	226	8	33	488	1	458	1.84E-156	450	COG0147	TrpE	Anthranilate/para-aminobenzoate synthases component I
LN02_09190|LN02Chr07:1698162-1699250(-)|362	CDD:224318	35.227	88	54	2	171	258	4	88	6.92E-11	55.4	COG1400	SEC65	Signal recognition particle 19 kDa protein
LN02_09254|LN02Chr07:1951626-1952329(-)|203	CDD:224876	30.435	115	65	3	85	199	1	100	3.70E-26	94.3	COG1965	CyaY	Protein implicated in iron transport, frataxin homolog
LN02_00167|LN02Chr01:777532-779652(+)|706	CDD:227664	30.753	465	281	14	38	489	5	441	3.91E-72	241	COG5371	COG5371	Golgi nucleoside diphosphatase
LN02_00295|LN02Chr01:1588683-1590442(+)|476	CDD:225671	33.824	272	165	6	63	320	4	274	1.61E-60	197	COG3129	COG3129	Predicted SAM-dependent methyltransferase
LN02_00359|LN02Chr01:1897422-1898681(-)|419	CDD:225451	24.828	290	189	10	99	383	238	503	3.07E-16	77.4	COG2898	COG2898	Uncharacterized conserved protein
LN02_00551|LN02Chr01:2443221-2445123(+)|535	CDD:223552	29.05	179	104	5	14	183	2	166	4.65E-20	87.1	COG0476	ThiF	Dinucleotide-utilizing enzymes involved in molybdopterin and thiamine biosynthesis family 2
LN02_00743|LN02Chr01:3221156-3222725(+)|451	CDD:227284	27.735	393	222	13	44	432	38	372	6.58E-66	213	COG4948	COG4948	L-alanine-DL-glutamate epimerase and related enzymes of enolase superfamily
LN02_00871|LN02Chr01:3642982-3644604(-)|450	CDD:223153	34.473	351	218	6	72	421	4	343	5.64E-103	309	COG0075	COG0075	Serine-pyruvate aminotransferase/archaeal aspartate aminotransferase
LN02_00999|LN02Chr01:4049839-4054704(-)|1570	CDD:223589	22.261	283	188	10	26	287	3	274	1.68E-24	104	COG0515	SPS1	Serine/threonine protein kinase
LN02_00999|LN02Chr01:4049839-4054704(-)|1570	CDD:225201	21.545	246	164	9	1122	1365	187	405	6.86E-10	60.1	COG2319	COG2319	FOG: WD40 repeat

LN02_01127∣LN02Chr01:4492345-4493670(-)∣365	CDD:224232	35.855	304	152	9	52	351	65	329	5.12E-82	250	COG1313	PflX	Uncharacterized Fe-S protein PflX, homolog of pyruvate formate lyase activating proteins
LN02_01191∣LN02Chr01:4817214-4818527(-)∣382	CDD:223589	27.419	310	183	12	85	382	8	287	1.21E-33	126	COG0515	SPS1	Serine/threonine protein kinase
LN02_01255∣LN02Chr01:5067620-5074178(-)∣2080	CDD:225858	34.302	895	531	18	382	1246	6	873	0	613	COG3321	COG3321	Polyketide synthase modules and related proteins
LN02_01255∣LN02Chr01:5067620-5074178(-)∣2080	CDD:225856	23.881	268	168	10	1831	2078	3	254	1.42E-24	102	COG3319	COG3319	Thioesterase domains of type I polyketide synthases or non-ribosomal peptide synthetases
LN02_01255∣LN02Chr01:5067620-5074178(-)∣2080	CDD:223314	30.882	68	46	1	1670	1736	8	75	5.51E-05	40.7	COG0236	AcpP	Acyl carrier protein
LN02_01319∣LN02Chr01:5245547-5248372(-)∣713	CDD:223560	47.059	85	43	2	5	89	4	86	8.17E-25	103	COG0484	DnaJ	DnaJ-class molecular chaperone with C-terminal Zn finger domain
LN02_01447∣LN02Chr01:5854186-5857481(+)∣845	CDD:226406	23.864	176	117	5	349	512	692	862	1.03E-04	42.9	COG3889	COG3889	Predicted solute binding protein
LN02_01511∣LN02Chr01:6032311-6037339(-)∣1556	CDD:223589	28.521	284	174	10	1266	1531	5	277	4.96E-47	171	COG0515	SPS1	Serine/threonine protein kinase
LN02_01639∣LN02Chr01:6550985-6551962(-)∣325	CDD:226297	22.901	262	164	11	78	321	73	314	6.35E-23	94.4	COG3774	OCH1	Mannosyltransferase OCH1 and related enzymes
LN02_01703∣LN02Chr01:6806807-6807769(-)∣320	CDD:224019	37.195	164	101	2	34	195	4	167	2.12E-58	183	COG1094	COG1094	Predicted RNA-binding protein (contains KH domains)
LN02_01831∣LN02Chr01:7163088-7166977(-)∣1246	CDD:224177	43.571	420	222	6	796	1207	1	413	1.51E-159	481	COG1257	HMG1	Hydroxymethylglutaryl-CoA reductase
LN02_02151∣LN02Chr02:747658-749394(+)∣578	CDD:223915	26.552	290	161	10	88	375	1	240	6.14E-47	162	COG0846	SIR2	NAD-dependent protein deacetylases, SIR2 family
LN02_02215∣LN02Chr02:952282-953633(-)∣367	CDD:225714	31.661	319	190	11	31	349	30	320	1.65E-54	179	COG3173	COG3173	Predicted aminoglycoside phosphotransferase
LN02_02279∣LN02Chr02:1204999-1209191(+)∣1356	CDD:223589	33.214	280	164	9	1050	1314	7	278	8.14E-57	199	COG0515	SPS1	Serine/threonine protein kinase
LN02_02471∣LN02Chr02:1936917-1938604(-)∣508	CDD:224100	34.962	266	157	4	96	358	7	259	5.73E-74	232	COG1179	COG1179	Dinucleotide-utilizing enzymes involved in molybdopterin and thiamine biosynthesis family 1
LN02_02599∣LN02Chr02:2372651-2373508(-)∣263	CDD:223711	30	230	130	6	30	257	20	220	1.32E-42	142	COG0638	PRE1	20S proteasome, alpha and beta subunits
LN02_02663∣LN02Chr02:2594193-2595352(+)∣327	CDD:227422	23	100	66	3	219	312	174	268	4.60E-06	44.7	COG5091	SGT1	Suppressor of G2 allele of skp1 and related proteins
LN02_02727∣LN02Chr02:2788636-2790021(+)∣364	CDD:223407	24.603	252	174	6	95	339	2	244	3.21E-30	114	COG0330	HflC	Membrane protease subunits, stomatin/prohibitin homologs
LN02_02791∣LN02Chr02:3008495-3010124(-)∣496	CDD:226098	16.822	321	157	14	57	358	26	255	2.93E-06	45.9	COG3568	ElsH	Metal-dependent hydrolase
LN02_02919∣LN02Chr02:3467286-3468839(-)∣495	CDD:224117	21.6	125	97	1	168	291	676	800	6.84E-06	45.9	COG1196	Smc	Chromosome segregation ATPases

LN02_02983	LN02Chr02:3677183-3678142(+)	290	CDD:223774	22.222	90	60	1	6	95	1	80	3.77E-09	53.4	COG0702	COG0702	Predicted nucleoside-diphosphate-sugar epimerases
LN02_03047	LN02Chr02:3911830-3913044(+)	404	CDD:223431	28.512	242	143	6	52	287	13	230	1.04E-46	159	COG0354	COG0354	Predicted aminomethyltransferase related to GcvT

LN02_03367	LN02Chr02:5005685-5006689(+)	215	CDD:227455	27.225	191	88	4	16	202	12	155	1.64E-33	115	COG5126	FRQ1	Ca2+-binding protein (EF-Hand superfamily)
LN02_03495	LN02Chr02:5781483-5783421(-)	599	CDD:225142	28.773	497	290	7	65	560	3	436	1.22E-87	276	COG2233	UraA	Xanthine/uracil permeases
LN02_03687	LN02Chr03:314166-314925(-)	234	CDD:224584	24.812	133	93	4	63	188	29	161	1.46E-12	61.5	COG1670	RimL	Acetyltransferases, including N-acetylases of ribosomal proteins
LN02_03815	LN02Chr03:823751-824337(+)	131	CDD:223171	45.038	131	59	3	1	129	1	120	1.58E-42	133	COG0093	RplN	Ribosomal protein L14
LN02_03943	LN02Chr03:1205842-1206979(+)	250	CDD:227489	23.558	208	129	8	29	228	383	568	1.01E-12	64.2	COG5160	ULP1	Protease, Ulp1 family
LN02_04071	LN02Chr03:2009689-2010322(+)	173	CDD:224704	34.94	166	95	4	1	155	2	165	2.63E-46	147	COG1791	COG1791	Uncharacterized conserved protein, contains double-stranded beta-helix domain
LN02_04135	LN02Chr03:2777337-2778361(+)	254	CDD:223168	41.176	255	135	6	1	253	34	275	8.47E-86	254	COG0090	RplB	Ribosomal protein L2
LN02_04263	LN02Chr03:3591358-3593116(+)	413	CDD:223774	24.211	285	199	5	56	339	5	273	6.85E-25	100	COG0702	COG0702	Predicted nucleoside-diphosphate-sugar epimerases
LN02_04391	LN02Chr03:4028945-4029967(-)	340	CDD:224007	21.407	327	190	11	1	315	3	274	1.83E-14	69.5	COG1082	IolE	Sugar phosphate isomerases/epimerases
LN02_04455	LN02Chr03:4214579-4216076(+)	451	CDD:225180	24.26	169	115	5	93	251	60	225	6.50E-08	51.5	COG2271	UhpC	Sugar phosphate permease
LN02_04455	LN02Chr03:4214579-4216076(+)	451	CDD:223553	17.134	321	262	2	59	375	1	321	5.14E-07	48.5	COG0477	ProP	Permeases of the major facilitator superfamily
LN02_04519	LN02Chr03:4424424-4425127(+)	114	CDD:223102	31.858	113	65	4	1	112	3	104	9.51E-21	77.3	COG0023	SUI1	Translation initiation factor 1 (eIF-1/SUI1) and related proteins
LN02_04839	LN02Chr03:5644107-5647154(-)	939	CDD:226406	22.093	172	115	7	30	195	694	852	1.33E-05	46	COG3889	COG3889	Predicted solute binding protein
LN02_05095	LN02Chr04:596691-598269(+)	375	CDD:225136	35	40	26	0	113	152	46	85	3.75E-06	44.6	COG2226	UbiE	Methylase involved in ubiquinone/menaquinone biosynthesis
LN02_05159	LN02Chr04:790157-791855(-)	316	CDD:225201	33.762	311	183	10	6	310	146	439	1.40E-39	142	COG2319	COG2319	FOG: WD40 repeat
LN02_05351	LN02Chr04:1466004-1467138(+)	329	CDD:227884	23.669	169	104	5	50	206	69	224	5.59E-14	69.1	COG5597	COG5597	Alpha-N-acetylglucosamine transferase
LN02_05543	LN02Chr04:2374288-2379097(-)	1580	CDD:223206	40.69	435	233	12	400	831	10	422	2.79E-144	447	COG0128	AroA	5-enolpyruvylshikimate-3-phosphate synthase
LN02_05543	LN02Chr04:2374288-2379097(-)	1580	CDD:223414	40.896	357	182	6	35	390	27	355	2.96E-120	379	COG0337	AroB	3-dehydroquinate synthetase
LN02_05543	LN02Chr04:2374288-2379097(-)	1580	CDD:223247	30.612	294	187	6	1280	1570	3	282	6.20E-70	233	COG0169	AroE	Shikimate 5-dehydrogenase

LN02_05543∣LN02Chr04: 2374288-2379097(-)∣1580	CDD:223782	29.004	231	154	8	1054	1281	8	231	4.79E-44	157	COG0710	AroD	3-dehydroquinate dehydratase
LN02_05543∣LN02Chr04: 2374288-2379097(-)∣1580	CDD:223775	34.641	153	88	4	860	1007	3	148	6.41E-42	148	COG0703	AroK	Shikimate kinase
LN02_05735∣LN02Chr04: 3030423-3031818(+)∣387	CDD:224322	34.317	271	141	11	113	351	116	381	3.27E-24	101	COG1404	AprE	Subtilisin-like serine proteases
LN02_05927∣LN02Chr04: 3642595-3645524(+)∣909	CDD:224418	24.659	880	498	23	32	898	27	754	5.17E-124	390	COG1501	COG1501	Alpha-glucosidases, family 31 of glycosyl hydrolases
LN02_06247∣LN02Chr04: 4781865-4782721(-)∣254	CDD:227536	39.827	231	99	3	24	254	7	197	1.76E-77	230	COG5211	SSU72	RNA polymerase II-interacting protein involved in transcription start site selection
LN02_06311∣LN02Chr04: 4994465-4995843(-)∣269	CDD:223094	31.304	230	147	4	41	269	1	220	2.07E-60	194	COG0015	PurB	Adenylosuccinate lyase
LN02_06375∣LN02Chr04: 5191736-5193520(+)∣567	CDD:225371	20.875	297	223	6	56	346	5	295	7.79E-13	67.3	COG2814	AraJ	Arabinose efflux permease
LN02_06567∣LN02Chr04: 5879218-5879734(-)∣128	CDD:226219	40	115	54	4	6	119	3	103	2.49E-30	102	COG3695	COG3695	Predicted methylated DNA-protein cysteine methyltransferase
LN02_06759∣LN02Chr05: 713617-715666(-)∣497	CDD:223944	50.418	478	229	5	19	494	1	472	0	590	COG1012	PutA	NAD-dependent aldehyde dehydrogenases
LN02_06887∣LN02Chr05: 1166622-1168394(+)∣492	CDD:225371	21.053	133	102	2	83	215	58	187	4.73E-04	39.5	COG2814	AraJ	Arabinose efflux permease
LN02_07015∣LN02Chr05: 1964250-1967434(+)∣909	CDD:223116	22.379	496	301	11	201	695	22	434	3.42E-54	192	COG0038	EriC	Chloride channel protein EriC
LN02_07143∣LN02Chr05: 2724051-2726967(-)∣820	CDD:223540	51.009	545	214	8	237	779	1	494	0	551	COG0464	SpoVK	ATPases of the AAA+ class
LN02_07207∣LN02Chr05: 2981250-2983841(+)∣834	CDD:223769	23.49	149	98	3	72	217	38	173	2.12E-05	44.1	COG0697	RhaT	Permeases of the drug/metabolite transporter (DMT) superfamily
LN02_07335∣LN02Chr05: 3376804-3377363(-)∣130	CDD:223470	33.333	108	53	1	31	119	1	108	7.17E-21	78.5	COG0393	COG0393	Uncharacterized conserved protein
LN02_07463∣LN02Chr05: 3860528-3861964(-)∣446	CDD:224283	47.454	432	198	9	16	446	1	404	0	511	COG1364	ArgJ	N-acetylglutamate synthase (N-acetylornithine aminotransferase)
LN02_07655∣LN02Chr05: 4746484-4747300(-)∣220	CDD:227840	21.875	160	113	5	23	180	6	155	7.14E-08	48	COG5553	COG5553	Predicted metal-dependent enzyme of the double-stranded beta helix superfamily
LN02_07783∣LN02Chr05: 5224825-5227872(-)∣988	CDD:226406	26.087	115	72	3	326	433	739	847	6.47E-04	40.6	COG3889	COG3889	Predicted solute binding protein
LN02_07975∣LN02Chr05: 5844886-5845863(-)∣325	CDD:223259	46.372	317	156	5	5	320	3	306	2.65E-122	350	COG0181	HemC	Porphobilinogen deaminase
LN02_08103∣LN02Chr06: 1256686-1260290(+)∣1099	CDD:225201	30.114	352	195	12	701	1036	137	453	4.48E-40	152	COG2319	COG2319	FOG: WD40 repeat
LN02_08231∣LN02Chr06: 1830956-1832841(+)∣529	CDD:223884	17.506	417	308	8	125	516	8	413	1.70E-31	123	COG0814	SdaC	Amino acid permeases
LN02_08359∣LN02Chr06: 2223692-2226879(-)∣978	CDD:227408	26.812	138	90	3	540	671	107	239	3.37E-11	63.3	COG5076	COG5076	Transcription factor involved in chromatin remodeling, contains bromodomain

LN02_08359∣LN02Chr06: 2223692-2226879(-)∣978	CDD:227408	24.699	166	111	6	282	437	90	251	6.53E-06	46.3	COG5076	COG5076	Transcription factor involved in chromatin remodeling, contains bromodomain
LN02_08423∣LN02Chr06: 2520260-2523129(-)∣911	CDD:227382	41.026	897	451	17	1	870	1	846	0	854	COG5049	XRN1	5'-3' exonuclease
LN02_08871∣LN02Chr07: 645333-645944(+)∣178	CDD:225736	29.358	109	65	4	78	176	66	172	7.63E-09	49.7	COG3195	COG3195	Uncharacterized protein conserved in bacteria
LN02_09255∣LN02Chr07: 1952884-1953527(+)∣188	CDD:227363	37.427	171	87	3	1	171	1	151	1.88E-50	157	COG5030	APS2	Clathrin adaptor complex, small subunit
LN02_09319∣LN02Chr07: 2194845-2197192(+)∣752	CDD:224287	18.433	217	156	8	408	618	349	550	1.63E-11	64.4	COG1368	MdoB	Phosphoglycerol transferase and related proteins, alkaline phosphatase superfamily
LN02_00104∣LN02Chr01: 496817-497973(-)∣350	CDD:225313	44.848	330	163	8	18	345	7	319	5.58E-112	326	COG2515	Acd	1-aminocyclopropane-1-carboxylate deaminase
LN02_00488∣LN02Chr01: 2271857-2274683(+)∣857	CDD:227820	23.575	386	238	16	391	762	53	395	7.99E-27	111	COG5533	UBP5	Ubiquitin C-terminal hydrolase
LN02_00488∣LN02Chr01: 2271857-2274683(+)∣857	CDD:225711	22.667	150	112	4	203	350	236	383	9.28E-05	43	COG3170	FimV	Tfp pilus assembly protein FimV
LN02_00616∣LN02Chr01: 2721892-2723900(-)∣525	CDD:226406	23.596	89	66	2	111	199	767	853	1.69E-04	41.4	COG3889	COG3889	Predicted solute binding protein
LN02_00744∣LN02Chr01: 3223387-3224814(+)∣427	CDD:227829	20.053	374	247	11	64	427	76	407	7.72E-19	85.2	COG5542	COG5542	Predicted integral membrane protein
LN02_00936∣LN02Chr01: 3824613-3825820(+)∣202	CDD:223180	37.857	140	70	3	4	131	12	146	6.02E-43	138	COG0102	RplM	Ribosomal protein L13
LN02_01192∣LN02Chr01: 4819458-4822360(+)∣898	CDD:227441	45.62	879	458	10	23	894	12	877	0	1006	COG5110	RPN1	26S proteasome regulatory complex component
LN02_01384∣LN02Chr01: 5532086-5537832(-)∣1891	CDD:223589	21.609	435	167	9	693	1112	1	276	9.61E-31	123	COG0515	SPS1	Serine/threonine protein kinase
LN02_01384∣LN02Chr01: 5532086-5537832(-)∣1891	CDD:223855	27.82	133	87	4	1483	1609	1	130	1.09E-19	84.1	COG0784	CheY	FOG: CheY-like receiver
LN02_01512∣LN02Chr01: 6039920-6042232(-)∣770	CDD:225344	24.468	94	68	1	35	125	44	137	7.89E-05	42.9	COG2730	BglC	Endoglucanase
LN02_01640∣LN02Chr01: 6555167-6556284(+)∣304	CDD:225031	23.913	138	91	4	60	191	5	134	4.31E-17	75.9	COG2120	COG2120	Uncharacterized proteins, LmbE homologs
LN02_01704∣LN02Chr01: 6808174-6808748(+)∣157	CDD:223158	45.89	146	69	3	11	156	4	139	2.21E-50	155	COG0080	RplK	Ribosomal protein L11
LN02_01832∣LN02Chr01: 7169496-7170925(-)∣382	CDD:226022	33.133	332	193	7	12	338	6	313	3.65E-87	264	COG3491	PcbC	Isopenicillin N synthase and related dioxygenases
LN02_01960∣LN02Chr02: 113525-114122(-)∣113	CDD:223277	44.262	61	34	0	53	113	1	61	4.16E-15	61.5	COG0199	RpsN	Ribosomal protein S14
LN02_02152∣LN02Chr02: 750030-753586(+)∣1138	CDD:223327	31.37	883	510	20	227	1103	6	798	0	567	COG0249	MutS	Mismatch repair ATPase (MutS family)
LN02_02216∣LN02Chr02: 955166-955852(-)∣228	CDD:227549	30.952	168	109	4	24	191	83	243	7.08E-21	84.9	COG5224	HAP2	CCAAT-binding factor, subunit B

LN02_02280∣LN02Chr02: 1210092-1210859(+)∣165	CDD:2233 72	45.113	133	68	1	32	164	7	134	1.56E-52	160	COG02 95	Cdd	Cytidine deaminase
LN02_02344∣LN02Chr02: 1485853-1486258(+)∣113	CDD:2274 20	38.144	97	59	1	17	113	15	110	1.57E-24	87.2	COG50 88	SOH1	Rad5p-binding protein
LN02_02408∣LN02Chr02: 1728569-1730534(-)∣591	CDD:2235 89	28.107	338	171	12	205	520	3	290	1.99E-39	146	COG05 15	SPS1	Serine/threonine protein kinase
LN02_02536∣LN02Chr02: 2151687-2152964(+)∣329	CDD:2234 89	22.65	234	160	9	106	329	12	234	2.00E-25	99.4	COG04 12	COG041 2	Dienelactone hydrolase and related enzymes
LN02_02600∣LN02Chr02: 2373935-2379941(+)∣1941	CDD:2273 65	24.824	568	338	21	1458	1941	1539	2101	9.60E-73	267	COG50 32	TEL1	Phosphatidylinositol kinase and protein kinases of the PI-3 kinase family
LN02_02728∣LN02Chr02: 2790939-2791422(+)∣86	CDD:3192 44	53.03	66	31	0	18	83	9	74	1.85E-19	72.1	COG52 72	UBI4	UBI4; linked to 3D-structure.
LN02_02856∣LN02Chr02: 3235176-3236777(+)∣533	CDD:2277 01	26.733	404	243	9	124	524	31	384	8.57E-52	178	COG54 14	COG541 4	TATA-binding protein-associated factor
LN02_03048∣LN02Chr02: 3914723-3915550(+)∣275	CDD:2235 60	43.939	66	32	2	21	83	5	68	8.62E-13	64.6	COG04 84	DnaJ	DnaJ-class molecular chaperone with C-terminal Zn finger domain
LN02_03048∣LN02Chr02: 3914723-3915550(+)∣275	CDD:2251 24	20.339	177	135	2	20	190	6	182	9.04E-06	42.9	COG22 14	CbpA	DnaJ-class molecular chaperone
LN02_03112∣LN02Chr02: 4126231-4128200(+)∣367	CDD:2240 85	55.858	367	160	2	1	367	1	365	0	539	COG11 63	DRG	Predicted GTPase
LN02_03560∣LN02Chr02: 6179495-6180916(-)∣473	CDD:2264 06	23.377	154	93	6	210	350	694	835	3.98E-04	39.8	COG38 89	COG388 9	Predicted solute binding protein
LN02_03752∣LN02Chr03: 534789-535911(+)∣339	CDD:2268 03	22.188	329	183	12	12	320	11	286	3.18E-31	116	COG43 60	APA2	ATP adenylyltransferase (5',5'''-P-1,P-4-tetraphosphate phosphorylase II)
LN02_03816∣LN02Chr03: 824976-826085(-)∣130	CDD:2245 10	34.821	112	67	2	22	129	1	110	4.18E-27	94.4	COG15 94	RPB9	DNA-directed RNA polymerase, subunit M/Transcription elongation factor TFIIS
LN02_03880∣LN02Chr03: 996904-1002584(-)∣1876	CDD:2236 27	37.391	575	297	18	1269	1811	323	866	1.07E-116	389	COG05 53	HepA	Superfamily II DNA/RNA helicases, SNF2 family
LN02_04008∣LN02Chr03: 1412436-1415128(+)∣765	CDD:2275 35	24.891	229	124	6	115	340	257	440	1.82E-17	82.9	COG52 10	COG521 0	GTPase-activating protein
LN02_04072∣LN02Chr03: 2011395-2012299(+)∣231	CDD:2231 13	46	200	107	1	30	229	9	207	5.20E-91	264	COG00 35	Upp	Uracil phosphoribosyltransferase
LN02_04136∣LN02Chr03: 2779385-2780949(+)∣349	CDD:2235 98	22.801	307	215	8	37	340	11	298	5.72E-42	145	COG05 24	RbsK	Sugar kinases, ribokinase family
LN02_04200∣LN02Chr03: 3321210-3323240(+)∣649	CDD:2240 83	28.045	353	188	7	159	506	9	300	1.29E-58	197	COG11 61	COG116 1	Predicted GTPases
LN02_04264∣LN02Chr03: 3593583-3596046(+)∣803	CDD:2236 27	33.818	550	323	15	208	732	333	866	1.56E-112	360	COG05 53	HepA	Superfamily II DNA/RNA helicases, SNF2 family
LN02_04328∣LN02Chr03: 3813515-3815479(+)∣536	CDD:2273 81	43.077	65	34	2	413	475	32	95	2.51E-07	50.1	COG50 48	COG504 8	FOG: Zn-finger
LN02_04392∣LN02Chr03: 4031207-4033811(-)∣650	CDD:2239 81	53.184	581	242	10	61	633	4	562	0	662	COG10 53	SdhA	Succinate dehydrogenase/fumarate reductase, flavoprotein subunit
LN02_04456∣LN02Chr03: 4216596-4218051(-)∣468	CDD:2235 29	18.28	279	207	7	44	316	82	345	5.56E-09	54.7	COG04 52	Dfp	Phosphopantothenoylcysteine synthetase/decarboxylase

LN02_04584|LN02Chr03: 4678938-4682455(-)|772	CDD:2274 00	16.461	243	190	3	69	304	163	399	6.65E-05	43.1	COG50 68	ARG80	Regulator of arginine metabolism and related MADS box-containing transcription factors
LN02_04648|LN02Chr03: 4914775-4915722(-)|295	CDD:2266 91	38.258	264	143	8	4	262	20	268	1.42E-65	205	COG42 40	COG424 0	Predicted kinase
LN02_04904|LN02Chr03: 5913550-5914997(-)|415	CDD:2273 53	56.386	321	140	0	91	411	79	399	7.11E-178	499	COG50 20	KTR1	Mannosyltransferase
LN02_04968|LN02Chr04: 107209-112083(-)|1462	CDD:2264 06	21.547	181	116	6	390	554	692	862	3.82E-04	41.8	COG38 89	COG388 9	Predicted solute binding protein
LN02_05032|LN02Chr04: 397234-398920(+)|458	CDD:2237 21	39.796	294	157	4	109	400	1	276	1.70E-97	291	COG06 48	Nfo	Endonuclease IV
LN02_05352|LN02Chr04: 1468228-1469748(+)|506	CDD:2260 34	28.169	142	81	7	36	175	14	136	8.06E-06	44.7	COG35 03	COG350 3	Predicted membrane protein
LN02_05416|LN02Chr04: 1678342-1679833(+)|445	CDD:2279 28	44.928	69	32	2	186	248	154	222	7.68E-12	64.1	COG56 41	GAT1	GATA Zn-finger-containing transcription factor
LN02_05416|LN02Chr04: 1678342-1679833(+)|445	CDD:2279 28	25.676	222	132	8	34	248	162	357	4.57E-11	61.4	COG56 41	GAT1	GATA Zn-finger-containing transcription factor
LN02_05672|LN02Chr04: 2815276-2816540(+)|279	CDD:2239 59	35.055	271	150	6	12	278	2	250	1.16E-52	169	COG10 28	FabG	Dehydrogenases with different specificities (related to short-chain alcohol dehydrogenases)
LN02_05800|LN02Chr04: 3237565-3238562(+)|251	CDD:2237 11	43.049	223	123	3	3	224	1	220	2.09E-77	231	COG06 38	PRE1	20S proteasome, alpha and beta subunits
LN02_05928|LN02Chr04: 3646397-3648566(+)|533	CDD:2239 03	45.347	505	268	6	35	532	38	541	0	569	COG08 33	LysP	Amino acid transporters
LN02_05992|LN02Chr04: 3942323-3942977(+)|123	CDD:2250 08	43.011	93	48	2	15	107	2	89	4.06E-20	75.5	COG20 97	RPL31A	Ribosomal protein L31E
LN02_06312|LN02Chr04: 4996886-4997884(-)|154	CDD:2249 43	33.121	157	91	5	4	151	28	179	1.20E-40	131	COG20 32	SodC	Cu/Zn superoxide dismutase
LN02_06440|LN02Chr04: 5394358-5395375(-)|260	CDD:2239 59	38.077	260	143	7	6	257	1	250	2.66E-56	178	COG10 28	FabG	Dehydrogenases with different specificities (related to short-chain alcohol dehydrogenases)
LN02_06568|LN02Chr04: 5880952-5886419(-)|1606	CDD:2273 92	18.69	519	243	12	79	469	37	504	2.05E-18	87.9	COG50 59	KIP1	Kinesin-like protein
LN02_06568|LN02Chr04: 5880952-5886419(-)|1606	CDD:2241 17	16.821	862	604	20	828	1600	173	1010	3.25E-14	75.1	COG11 96	Smc	Chromosome segregation ATPases
LN02_06568|LN02Chr04: 5880952-5886419(-)|1606	CDD:2241 17	18.98	843	578	17	356	1169	236	1002	1.08E-09	60.5	COG11 96	Smc	Chromosome segregation ATPases
LN02_06632|LN02Chr05: 177345-179127(-)|563	CDD:2275 32	18.2	500	318	16	90	540	284	741	2.86E-12	66.2	COG52 07	UBP14	Isopeptidase T
LN02_06632|LN02Chr05: 177345-179127(-)|563	CDD:3192 44	19.355	62	50	0	6	67	3	64	1.19E-05	40.5	COG52 72	UBI4	UBI4; linked to 3D-structure.
LN02_06824|LN02Chr05: 932154-933307(+)|331	CDD:2236 62	19.608	153	97	6	53	186	9	154	1.09E-06	44.9	COG05 89	UspA	Universal stress protein UspA and related nucleotide-binding proteins
LN02_06888|LN02Chr05: 1168483-1171054(-)|386	CDD:2234 95	33.762	311	152	11	108	384	52	342	2.34E-74	232	COG04 18	PyrC	Dihydroorotase

LN02_06952|LN02Chr05: 1773841-1776208(+)|743	CDD:2240 55	35.69	580	311	9	170	703	4	567	4.98E- 138	415	COG11 32	MdlB	ABC-type multidrug transport system, ATPase and permease components
LN02_07016|LN02Chr05: 1967725-1970723(-)|856	CDD:2247 06	29.965	574	268	19	266	836	1	443	3.91E-96	305	COG17 93	CDC9	ATP-dependent DNA ligase
LN02_07080|LN02Chr05: 2495916-2497913(-)|665	CDD:2274 47	28.391	634	415	12	38	661	49	653	1.28E- 104	329	COG51 17	NOC3	Protein involved in the nuclear export of pre-ribosomes
LN02_07144|LN02Chr05: 2728325-2729512(-)|365	CDD:2265 73	25.824	364	156	13	1	357	1	257	2.30E-41	143	COG40 88	COG408 8	Predicted nucleotide kinase
LN02_07208|LN02Chr05: 2984741-2985597(+)|270	CDD:2244 41	21.212	165	99	6	125	269	24	177	9.60E-13	64.5	COG15 24	COG152 4	Uncharacterized proteins of the AP superfamily
LN02_07336|LN02Chr05: 3378547-3379092(-)|113	CDD:2238 92	49.206	126	45	3	5	113	26	149	1.18E-37	121	COG08 22	IscU	NifU homolog involved in Fe-S cluster formation
LN02_07400|LN02Chr05: 3622813-3624894(-)|624	CDD:2240 54	28.929	280	175	8	25	300	2	261	1.27E-53	182	COG11 31	CcmA	ABC-type multidrug transport system, ATPase component
LN02_07464|LN02Chr05: 3864563-3866056(+)|461	CDD:2241 05	25.484	310	212	6	157	458	1	299	9.45E-47	160	COG11 84	GCD2	Translation initiation factor 2B subunit, eIF-2B alpha/beta/delta family
LN02_07528|LN02Chr05: 4075949-4076827(+)|236	CDD:2273 87	47.748	111	57	1	88	198	72	181	2.20E-49	157	COG50 54	ERV1	Mitochondrial sulfhydryl oxidase involved in the biogenesis of cytosolic Fe/S proteins
LN02_07592|LN02Chr05: 4411895-4412657(+)|220	CDD:2274 72	29.534	193	126	4	7	198	6	189	9.68E-40	132	COG51 43	SNC1	Synaptobrevin/VAMP-like protein
LN02_07784|LN02Chr05: 5233313-5234796(+)|462	CDD:2235 97	32.734	278	169	6	4	276	1	265	4.87E-48	165	COG05 23	COG052 3	Putative GTPases (G3E family)
LN02_07848|LN02Chr05: 5487191-5488525(-)|402	CDD:2235 87	41.24	371	212	4	28	392	29	399	1.07E- 145	421	COG05 13	SrmB	Superfamily II DNA and RNA helicases
LN02_08488|LN02Chr06: 2773626-2776916(-)|1032	CDD:2235 89	26.211	351	217	10	649	975	2	334	7.05E-41	152	COG05 15	SPS1	Serine/threonine protein kinase
LN02_08616|LN02Chr06: 3168230-3169796(-)|462	CDD:2271 70	24.868	189	118	8	175	354	133	306	9.45E-08	50.8	COG48 33	COG483 3	Predicted glycosyl hydrolase
LN02_08744|LN02Chr07: 219589-221592(-)|497	CDD:2238 09	21.854	151	107	3	51	194	39	185	3.83E-04	39.6	COG07 38	FucP	Fucose permease
LN02_08872|LN02Chr07: 646905-648192(+)|393	CDD:2248 57	19.938	321	190	12	74	384	24	287	8.12E-07	47.3	COG19 46	TesB	Acyl-CoA thioesterase
LN02_08936|LN02Chr07: 869359-870161(-)|131	CDD:2246 31	45.69	116	61	1	12	127	19	132	9.18E-47	144	COG17 17	RPL32	Ribosomal protein L32E
LN02_09000|LN02Chr07: 1084947-1086780(+)|553	CDD:2234 66	35.294	357	215	9	109	455	1	351	9.29E-79	249	COG03 89	DinP	Nucleotidyltransferase/DNA polymerase involved in DNA repair
LN02_09128|LN02Chr07: 1517077-1518952(+)|557	CDD:2234 66	22.753	356	190	11	16	366	2	277	2.43E-31	122	COG03 89	DinP	Nucleotidyltransferase/DNA polymerase involved in DNA repair
LN02_09192|LN02Chr07: 1704062-1705174(-)|370	CDD:2233 61	21.637	342	161	12	26	365	3	239	5.15E-23	93.5	COG02 84	PyrF	Orotidine-5'-phosphate decarboxylase
LN02_09320|LN02Chr07: 2197856-2199610(-)|553	CDD:2251 86	22.378	572	342	21	19	540	4	523	5.31E-38	144	COG23 03	BetA	Choline dehydrogenase and related flavoproteins
LN02_00069|LN02Chr01: 385375-388176(+)|913	CDD:2275 35	27.095	358	181	6	461	808	139	426	1.57E-48	177	COG52 10	COG521 0	GTPase-activating protein

LN02_00581|LN02Chr01: 2609150-2610037(+)|295	CDD:2251 37	38.189	254	141	5	42	295	6	243	5.04E-78	234	COG22 27	UbiG	2-polyprenyl-3-methyl-5-hydroxy-6-metoxy-1,4-benzoquinol methylase
LN02_00709|LN02Chr01: 3109042-3110094(-)|321	CDD:2245 50	44.322	273	141	5	43	315	1	262	4.42E-121	345	COG16 35	THI4	Ribulose 1,5-bisphosphate synthetase, converts PRPP to RuBP, flavoprotein
LN02_00965|LN02Chr01: 3948893-3950488(+)|422	CDD:2232 82	17.127	181	119	5	68	248	6	155	3.84E-15	71.9	COG02 04	PlsC	1-acyl-sn-glycerol-3-phosphate acyltransferase
LN02_01029|LN02Chr01: 4153211-4154452(-)|413	CDD:2237 30	30.035	283	171	8	100	380	52	309	1.64E-43	151	COG06 57	Aes	Esterase/lipase
LN02_01093|LN02Chr01: 4401960-4405592(+)|1210	CDD:2245 57	38.83	752	396	18	496	1191	2	745	0	774	COG16 43	HrpA	HrpA-like helicases
LN02_01093|LN02Chr01: 4401960-4405592(+)|1210	CDD:2241 06	44.444	81	40	4	239	318	616	692	3.95E-15	77.2	COG11 85	Pnp	Polyribonucleotide nucleotidyltransferase (polynucleotide phosphorylase)
LN02_01093|LN02Chr01: 4401960-4405592(+)|1210	CDD:2241 06	18.727	267	165	10	236	467	298	547	1.90E-13	71.8	COG11 85	Pnp	Polyribonucleotide nucleotidyltransferase (polynucleotide phosphorylase)
LN02_01221|LN02Chr01: 4923407-4924621(+)|404	CDD:2271 70	32.057	209	118	9	142	344	88	278	1.72E-20	89.3	COG48 33	COG483 3	Predicted glycosyl hydrolase
LN02_01285|LN02Chr01: 5151387-5152563(+)|335	CDD:2242 29	24.818	137	92	3	49	177	1	134	1.89E-25	96.4	COG13 10	COG131 0	Predicted metal-dependent protease of the PAD1/JAB1 superfamily
LN02_01413|LN02Chr01: 5652810-5655179(+)|701	CDD:2264 06	21.134	194	116	7	251	432	694	862	2.55E-05	44.5	COG38 89	COG388 9	Predicted solute binding protein
LN02_01669|LN02Chr01: 6694627-6695449(+)|238	CDD:2274 72	21.705	129	96	2	80	208	66	189	8.91E-15	67.5	COG51 43	SNC1	Synaptobrevin/VAMP-like protein
LN02_01733|LN02Chr01: 6881278-6882777(+)|499	CDD:2235 18	38.559	472	211	11	29	493	179	578	1.61E-148	434	COG04 41	ThrS	Threonyl-tRNA synthetase
LN02_01925|LN02Chr01: 7424336-7425756(-)|455	CDD:2256 60	27.731	119	76	4	10	126	6	116	1.24E-09	56.2	COG31 18	COG311 8	Thioredoxin domain-containing protein
LN02_02053|LN02Chr02: 472511-474940(+)|789	CDD:2246 30	32.184	87	43	4	205	290	90	161	2.86E-13	66.1	COG17 16	COG171 6	FOG: FHA domain
LN02_02053|LN02Chr02: 472511-474940(+)|789	CDD:2241 17	17.442	172	124	3	498	669	235	388	5.83E-08	53.6	COG11 96	Smc	Chromosome segregation ATPases
LN02_02117|LN02Chr02: 661583-662750(-)|267	CDD:2233 13	25.751	233	131	7	25	251	1	197	8.74E-31	111	COG02 35	AraD	Ribulose-5-phosphate 4-epimerase and related epimerases and aldolases
LN02_02245|LN02Chr02: 1053830-1055397(+)|488	CDD:2237 27	30.357	448	245	10	39	485	3	384	1.06E-79	250	COG06 54	UbiH	2-polyprenyl-6-methoxyphenol hydroxylase and related FAD-dependent oxidoreductases
LN02_02373|LN02Chr02: 1590313-1596265(-)|1853	CDD:2237 38	32.941	85	49	2	515	591	86	170	5.12E-10	59.1	COG06 66	Arp	FOG: Ankyrin repeat
LN02_02373|LN02Chr02: 1590313-1596265(-)|1853	CDD:2237 38	30.488	82	52	2	1422	1498	104	185	1.33E-05	45.6	COG06 66	Arp	FOG: Ankyrin repeat
LN02_02373|LN02Chr02: 1590313-1596265(-)|1853	CDD:2237 38	23.333	180	99	5	1450	1628	63	204	2.19E-05	44.8	COG06 66	Arp	FOG: Ankyrin repeat
LN02_02501|LN02Chr02: 2038566-2039072(+)|168	CDD:2245 06	33.645	107	59	5	2	108	98	192	4.66E-11	56.2	COG15 90	COG159 0	Uncharacterized conserved protein

LN02_02565|LN02Chr02:2256701-2258524(-)|519	CDD:224136	18.072	415	304	11	18	418	5	397	5.65E-12	64.6	COG1215	COG1215	Glycosyltransferases, probably involved in cell wall biogenesis
LN02_02629|LN02Chr02:2481624-2482775(-)|269	CDD:227479	29.167	96	68	0	49	144	8	103	2.80E-08	48.8	COG5150	COG5150	Class 2 transcription repressor NC2, beta subunit (Dr1)
LN02_02693|LN02Chr02:2698797-2700451(-)|502	CDD:223600	27.174	92	66	1	375	465	31	122	2.50E-08	49.9	COG0526	TrxA	Thiol-disulfide isomerase and thioredoxins
LN02_02693|LN02Chr02:2698797-2700451(-)|502	CDD:223600	25	96	68	3	24	116	15	109	6.60E-07	45.7	COG0526	TrxA	Thiol-disulfide isomerase and thioredoxins
LN02_02821|LN02Chr02:3128271-3131771(+)|1146	CDD:223466	27.968	379	237	8	361	735	3	349	4.33E-58	201	COG0389	DinP	Nucleotidyltransferase/DNA polymerase involved in DNA repair
LN02_02885|LN02Chr02:3322649-3326818(-)|1362	CDD:223715	26.897	290	144	8	859	1147	105	327	8.29E-36	136	COG0642	BaeS	Signal transduction histidine kinase
LN02_02885|LN02Chr02:3322649-3326818(-)|1362	CDD:223855	36.296	135	68	4	1212	1344	8	126	1.14E-28	109	COG0784	CheY	FOG: CheY-like receiver
LN02_02885|LN02Chr02:3322649-3326818(-)|1362	CDD:225112	22.297	148	111	2	704	851	86	229	5.78E-08	52.2	COG2202	AtoS	FOG: PAS/PAC domain
LN02_03077|LN02Chr02:4018832-4020876(-)|567	CDD:223395	30.853	551	330	16	29	563	12	527	9.12E-115	348	COG0318	CaiC	Acyl-CoA synthetases (AMP-forming)/AMP-acid ligases II
LN02_03333|LN02Chr02:4901897-4904292(+)|706	CDD:227455	25	168	79	7	112	273	25	151	8.10E-12	61.2	COG5126	FRQ1	Ca2+-binding protein (EF-Hand superfamily)
LN02_03717|LN02Chr03:398482-399537(-)|351	CDD:225505	34.104	346	207	11	7	348	16	344	3.03E-71	223	COG2957	COG2957	Peptidylarginine deiminase and related enzymes
LN02_03781|LN02Chr03:733309-736204(-)|390	CDD:223182	39.906	426	218	7	1	389	1	425	9.11E-155	440	COG0104	PurA	Adenylosuccinate synthase
LN02_04037|LN02Chr03:1549162-1551057(-)|563	CDD:224637	36.105	421	173	7	138	557	1	326	4.34E-111	332	COG1723	COG1723	Uncharacterized conserved protein
LN02_04101|LN02Chr03:2366362-2370275(+)|1244	CDD:223533	15.748	254	206	3	908	1159	19	266	2.19E-06	47.9	COG0457	NrfG	FOG: TPR repeat
LN02_04165|LN02Chr03:2927576-2932759(+)|1302	CDD:227367	25.781	128	70	10	793	902	147	267	4.99E-06	46.9	COG5034	TNG2	Chromatin remodeling protein, contains PhD zinc finger
LN02_04229|LN02Chr03:3445891-3447053(-)|361	CDD:226952	29.134	254	157	7	100	333	40	290	3.41E-71	222	COG4586	COG4586	ABC-type uncharacterized transport system, ATPase component
LN02_04293|LN02Chr03:3691329-3693140(-)|525	CDD:225201	17.661	419	301	13	13	424	62	443	6.95E-05	42.4	COG2319	COG2319	FOG: WD40 repeat
LN02_04677|LN02Chr03:5037918-5042821(-)|1588	CDD:223550	18.411	1309	591	36	222	1524	51	888	8.65E-113	377	COG0474	MgtA	Cation transport ATPase
LN02_04805|LN02Chr03:5466221-5467774(-)|482	CDD:223669	17.778	360	194	7	111	469	22	280	1.28E-16	77.4	COG0596	MhpC	Predicted hydrolases or acyltransferases (alpha/beta hydrolase superfamily)
LN02_04869|LN02Chr03:5824988-5826575(-)|449	CDD:226407	28.704	432	205	14	8	438	4	333	3.41E-49	168	COG3890	ERG8	Phosphomevalonate kinase
LN02_05189|LN02Chr04:904893-906662(-)|589	CDD:224536	27.481	524	286	29	24	524	23	475	1.73E-55	192	COG1621	SacC	Beta-fructosidases (levanase/invertase)
LN02_05637|LN02Chr04:2708759-2710735(-)|548	CDD:223587	37.659	393	237	5	26	410	6	398	1.89E-137	405	COG0513	SrmB	Superfamily II DNA and RNA helicases

LN02_05701∣LN02Chr04:2926743-2927991(-)∣379	CDD:223743	16.456	237	165	3	42	278	6	209	8.08E-07	46.7	COG0671	PgpB	Membrane-associated phospholipid phosphatase
LN02_05893∣LN02Chr04:3537541-3538938(-)∣376	CDD:227356	46.4	375	188	3	1	368	73	441	0	515	COG5023	COG5023	Tubulin
LN02_05957∣LN02Chr04:3820080-3821050(+)∣200	CDD:224644	21.538	130	92	2	41	170	9	128	1.62E-06	43.1	COG1730	GIM5	Predicted prefoldin, molecular chaperone implicated in de novo protein folding
LN02_06213∣LN02Chr04:4681970-4682837(-)∣261	CDD:225998	23.164	177	113	6	15	186	1	159	1.44E-14	66.7	COG3467	COG3467	Predicted flavin-nucleotide-binding protein
LN02_06597∣LN02Chr05:56605-58330(-)∣498	CDD:223553	16.185	346	275	5	38	383	4	334	7.02E-04	38.9	COG0477	ProP	Permeases of the major facilitator superfamily
LN02_06661∣LN02Chr05:289939-291758(+)∣526	CDD:225035	27.23	213	110	6	310	522	241	408	1.15E-15	75.9	COG2124	CypX	Cytochrome P450
LN02_06789∣LN02Chr05:819586-820742(-)∣343	CDD:223536	27.247	356	198	9	9	338	6	326	5.46E-73	226	COG0460	ThrA	Homoserine dehydrogenase
LN02_06981∣LN02Chr05:1853166-1854038(+)∣237	CDD:223796	22.628	137	99	2	26	155	80	216	1.21E-12	63	COG0724	COG0724	RNA-binding proteins (RRM domain)
LN02_07429∣LN02Chr05:3764712-3765804(+)∣268	CDD:223555	51.931	233	107	4	34	266	1	228	3.08E-117	333	COG0479	FrdB	Succinate dehydrogenase/fumarate reductase, Fe-S protein subunit
LN02_07493∣LN02Chr05:3960352-3963703(+)∣1058	CDD:223957	20.422	1043	701	38	10	998	3	970	3.08E-122	395	COG1026	COG1026	Predicted Zn-dependent peptidases, insulinase-like
LN02_07557∣LN02Chr05:4146889-4149866(+)∣973	CDD:224418	31.761	721	418	18	172	888	82	732	1.38E-172	519	COG1501	COG1501	Alpha-glucosidases, family 31 of glycosyl hydrolases
LN02_07749∣LN02Chr05:5075595-5076545(-)∣316	CDD:223406	29.87	308	199	7	10	315	7	299	5.12E-58	186	COG0329	DapA	Dihydrodipicolinate synthase/N-acetylneuraminate lyase
LN02_07813∣LN02Chr05:5369219-5369872(+)∣117	CDD:224869	28.916	83	48	3	34	116	8	79	2.48E-14	60.4	COG1958	LSM1	Small nuclear ribonucleoprotein (snRNP) homolog
LN02_07877∣LN02Chr05:5570887-5572011(+)∣347	CDD:227352	21.818	55	43	0	222	276	313	367	5.86E-04	38.5	COG5019	CDC3	Septin family protein
LN02_08133∣LN02Chr06:1367931-1370842(+)∣869	CDD:223327	28.371	712	462	16	94	799	120	789	4.62E-129	405	COG0249	MutS	Mismatch repair ATPase (MutS family)
LN02_08197∣LN02Chr06:1744187-1745020(-)∣146	CDD:225108	23.256	86	62	1	43	128	32	113	1.37E-06	42	COG2198	ArcB	FOG: HPt domain
LN02_08389∣LN02Chr06:2430829-2432100(-)∣397	CDD:227518	27.778	216	152	3	4	215	1	216	1.43E-43	154	COG5191	COG5191	Uncharacterized conserved protein, contains HAT (Half-A-TPR) repeat
LN02_08453∣LN02Chr06:2661782-2663754(-)∣632	CDD:224117	24.315	292	193	7	73	358	224	493	4.26E-12	66.3	COG1196	Smc	Chromosome segregation ATPases
LN02_08645∣LN02Chr06:3275547-3282700(-)∣2259	CDD:225858	35.098	1020	607	19	9	1016	3	979	0	704	COG3321	COG3321	Polyketide synthase modules and related proteins
LN02_08645∣LN02Chr06:3275547-3282700(-)∣2259	CDD:223677	33.333	330	202	9	1544	1862	3	325	1.03E-59	206	COG0604	Qor	NADPH:quinone reductase and related Zn-dependent oxidoreductases
LN02_08645∣LN02Chr06:3275547-3282700(-)∣2259	CDD:223959	26.891	238	162	6	1888	2114	9	245	7.28E-21	91.4	COG1028	FabG	Dehydrogenases with different specificities (related to short-chain alcohol dehydrogenases)

LN02_09157|LN02Chr07:1595692-1597246(-)|337	CDD:223135	59.581	334	130	5	2	332	1	332	0	507	COG0057	GapA	Glyceraldehyde-3-phosphate dehydrogenase/erythrose-4-phosphate dehydrogenase
LN02_00041|LN02Chr01:306300-309736(+)|1008	CDD:223154	21.063	489	223	17	99	577	69	404	5.64E-24	103	COG0076	GadB	Glutamate decarboxylase and related PLP-dependent proteins
LN02_00809|LN02Chr01:3476763-3477634(-)|227	CDD:223447	14.754	183	136	5	50	224	121	291	2.99E-04	38.4	COG0370	FeoB	Fe2+ transport system protein B
LN02_00873|LN02Chr01:3648607-3651284(+)|770	CDD:224557	40.816	735	376	17	58	750	5	722	0	773	COG1643	HrpA	HrpA-like helicases
LN02_01001|LN02Chr01:4059898-4060743(-)|281	CDD:227464	20.608	296	161	13	6	281	4	245	9.54E-10	54.7	COG5135	COG5135	Uncharacterized conserved protein
LN02_01065|LN02Chr01:4282556-4284742(-)|510	CDD:225201	20.155	258	178	7	16	268	81	315	1.18E-18	85.5	COG2319	COG2319	FOG: WD40 repeat
LN02_01193|LN02Chr01:4822807-4826237(-)|1099	CDD:224216	17.737	327	202	12	772	1055	312	614	7.46E-05	43.5	COG1297	COG1297	Predicted membrane protein
LN02_01321|LN02Chr01:5251664-5254242(-)|487	CDD:227888	36.842	152	84	4	332	473	19	168	7.39E-29	109	COG5601	CDC36	General negative regulator of transcription subunit
LN02_01321|LN02Chr01:5251664-5254242(-)|487	CDD:227952	18.837	361	235	15	121	460	202	525	6.24E-12	64.7	COG5665	NOT5	CCR4-NOT transcriptional regulation complex, NOT5 subunit
LN02_01513|LN02Chr01:6043189-6046982(-)|777	CDD:223553	19.424	139	110	1	236	372	45	183	1.50E-04	41.6	COG0477	ProP	Permeases of the major facilitator superfamily
LN02_01641|LN02Chr01:6557529-6558633(-)|316	CDD:225182	22.01	209	125	11	73	269	59	241	4.28E-06	44.8	COG2273	SKN1	Beta-glucanase/Beta-glucan synthetase
LN02_01833|LN02Chr01:7172574-7173620(-)|286	CDD:225498	43.713	167	79	4	113	278	4	156	3.03E-59	183	COG2947	COG2947	Uncharacterized conserved protein
LN02_01897|LN02Chr01:7358515-7360368(+)|617	CDD:225371	27.632	152	110	0	100	251	38	189	3.52E-09	56.1	COG2814	AraJ	Arabinose efflux permease
LN02_01897|LN02Chr01:7358515-7360368(+)|617	CDD:223553	21.359	309	233	4	105	407	34	338	2.05E-05	43.9	COG0477	ProP	Permeases of the major facilitator superfamily
LN02_01961|LN02Chr02:114874-116190(-)|367	CDD:227402	52.273	308	137	3	51	358	9	306	3.26E-109	319	COG5070	VRG4	Nucleotide-sugar transporter
LN02_02089|LN02Chr02:578333-579964(+)|543	CDD:225179	17.094	468	319	17	47	499	13	426	7.06E-19	86.2	COG2270	COG2270	Permeases of the major facilitator superfamily
LN02_02345|LN02Chr02:1486639-1487111(-)|110	CDD:224510	32.759	116	69	4	1	110	1	113	3.18E-25	88.7	COG1594	RPB9	DNA-directed RNA polymerase, subunit M/Transcription elongation factor TFIIS
LN02_02409|LN02Chr02:1736475-1738331(+)|562	CDD:223751	23.353	167	108	2	49	215	5	151	1.13E-18	83.9	COG0679	COG0679	Predicted permeases
LN02_02601|LN02Chr02:2380349-2382616(-)|599	CDD:223131	22.695	282	205	8	308	583	14	288	2.09E-30	118	COG0053	MMT1	Predicted Co/Zn/Cd cation transporters
LN02_02665|LN02Chr02:2598169-2600336(+)|646	CDD:225179	18.895	561	313	9	72	618	6	438	1.16E-53	187	COG2270	COG2270	Permeases of the major facilitator superfamily
LN02_02793|LN02Chr02:3013720-3015333(-)|465	CDD:227507	29.333	75	48	2	271	345	529	598	2.53E-04	40.5	COG5180	PBP1	Protein interacting with poly(A)-binding protein
LN02_02793|LN02Chr02:3013720-3015333(-)|465	CDD:224768	25.424	59	44	0	395	453	490	548	2.95E-04	40	COG1855	COG1855	ATPase (PilT family)

LN02_02921 I LN02Chr02: 3474962-3476539(-) I 339	CDD:223589	26.3	327	192	7	31	337	1	298	2.71E-38	137	COG0515	SPS1	Serine/threonine protein kinase
LN02_03049 I LN02Chr02: 3915687-3916817(-) I 376	CDD:225070	23.019	265	137	9	117	374	86	290	1.20E-14	70.9	COG2159	COG2159	Predicted metal-dependent hydrolase of the TIM-barrel fold
LN02_03113 I LN02Chr02: 4129823-4134490(-) I 1494	CDD:223738	44.928	69	38	0	385	453	135	203	6.87E-12	64.1	COG0666	Arp	FOG: Ankyrin repeat
LN02_03113 I LN02Chr02: 4129823-4134490(-) I 1494	CDD:223738	22.222	171	108	5	341	488	2	170	3.10E-07	50.2	COG0666	Arp	FOG: Ankyrin repeat
LN02_03369 I LN02Chr02: 5016419-5017421(-) I 310	CDD:223181	46.457	127	68	0	184	310	4	130	1.58E-48	155	COG0103	RpsI	Ribosomal protein S9
LN02_03817 I LN02Chr03: 828713-829698(+) I 205	CDD:223831	21.667	120	92	2	87	204	139	258	1.35E-12	62.4	COG0760	SurA	Parvulin-like peptidyl-prolyl isomerase
LN02_03881 I LN02Chr03: 1005512-1009361(+) I 1202	CDD:224117	26.062	1201	841	17	1	1192	1	1163	1.58E-165	520	COG1196	Smc	Chromosome segregation ATPases
LN02_04073 I LN02Chr03: 2012470-2012968(-) I 100	CDD:227225	41.463	82	47	1	1	82	1	81	1.15E-29	99.5	COG4888	COG4888	Uncharacterized Zn ribbon-containing protein
LN02_04201 I LN02Chr03: 3327174-3330314(-) I 911	CDD:224136	16.25	320	224	9	528	835	138	425	8.60E-11	62.3	COG1215	COG1215	Glycosyltransferases, probably involved in cell wall biogenesis
LN02_04329 I LN02Chr03: 3817870-3821987(-) I 1217	CDD:225857	44.194	310	157	6	787	1088	4	305	3.86E-92	298	COG3320	COG3320	Putative dehydrogenase domain of multifunctional non-ribosomal peptide synthetases and related enzymes
LN02_04329 I LN02Chr03: 3817870-3821987(-) I 1217	CDD:223951	28.15	508	264	10	23	523	229	642	1.70E-84	286	COG1020	EntF	Non-ribosomal peptide synthetase modules and related proteins
LN02_04393 I LN02Chr03: 4034705-4038124(-) I 951	CDD:227680	36.538	52	29	1	225	276	157	204	2.06E-04	41.7	COG5391	COG5391	Phox homology (PX) domain protein
LN02_04521 I LN02Chr03: 4427982-4428978(+) I 292	CDD:223669	27.273	286	200	6	7	289	1	281	4.05E-35	125	COG0596	MhpC	Predicted hydrolases or acyltransferases (alpha/beta hydrolase superfamily)
LN02_05033 I LN02Chr04: 399508-403237(-) I 1188	CDD:223589	23.03	165	99	4	838	1002	1	137	7.51E-11	62.5	COG0515	SPS1	Serine/threonine protein kinase
LN02_05161 I LN02Chr04: 817894-820909(+) I 972	CDD:224254	25.328	229	132	8	66	286	5	202	1.35E-22	94.2	COG1335	PncA	Amidases related to nicotinamidase
LN02_05161 I LN02Chr04: 817894-820909(+) I 972	CDD:225687	20.952	210	127	9	496	699	18	194	4.95E-12	62.8	COG3145	AlkB	Alkylated DNA repair protein
LN02_05353 I LN02Chr04: 1470629-1473265(+) I 878	CDD:227583	24.635	479	218	13	163	633	44	387	1.28E-37	145	COG5258	GTPBP1	GTPase
LN02_05481 I LN02Chr04: 2013347-2016274(+) I 670	CDD:226406	20.482	83	66	0	19	101	753	835	5.95E-04	39.8	COG3889	COG3889	Predicted solute binding protein
LN02_05673 I LN02Chr04: 2816659-2818109(-) I 366	CDD:223980	39.007	282	155	6	65	336	50	324	6.91E-84	255	COG1052	LdhA	Lactate dehydrogenase and related dehydrogenases
LN02_05801 I LN02Chr04: 3238968-3240100(-) I 301	CDD:223743	26.282	156	101	2	104	259	77	218	2.29E-09	53.6	COG0671	PgpB	Membrane-associated phospholipid phosphatase
LN02_05865 I LN02Chr04: 3459253-3460172(+) I 246	CDD:225355	31.507	219	136	7	19	232	6	215	1.82E-35	123	COG2761	FrnE	Predicted dithiol-disulfide isomerase involved in polyketide biosynthesis
LN02_05993 I LN02Chr04: 3943688-3944412(-) I 180	CDD:227410	56.738	141	61	0	30	170	3	143	1.25E-68	203	COG5078	COG5078	Ubiquitin-protein ligase

LN02_06057∣LN02Chr04:4137915-4142003(+)∣1292	CDD:224055	32.526	578	363	9	62	637	12	564	2.19E-121	386	COG1132	MdlB	ABC-type multidrug transport system, ATPase and permease components
LN02_06057∣LN02Chr04:4137915-4142003(+)∣1292	CDD:224055	31.399	586	370	7	704	1289	14	567	3.14E-121	385	COG1132	MdlB	ABC-type multidrug transport system, ATPase and permease components
LN02_06121∣LN02Chr04:4341402-4344772(+)∣1097	CDD:226947	40	1015	547	21	104	1097	68	1041	0	908	COG4581	COG4581	Superfamily II RNA helicase
LN02_06185∣LN02Chr04:4603985-4605798(+)∣498	CDD:225035	22.102	371	233	15	112	477	73	392	4.82E-44	157	COG2124	CypX	Cytochrome P450
LN02_06249∣LN02Chr04:4787997-4789187(+)∣327	CDD:223617	32.273	220	126	9	80	297	9	207	4.50E-37	130	COG0543	UbiB	2-polyprenylphenol hydroxylase and related flavodoxin oxidoreductases
LN02_06313∣LN02Chr04:4998978-5000487(+)∣479	CDD:223608	18.619	333	237	7	105	430	105	410	3.42E-12	65.3	COG0534	NorM	Na+-driven multidrug efflux pump
LN02_06441∣LN02Chr04:5396014-5398315(-)∣713	CDD:223589	28.428	299	167	11	428	698	1	280	1.61E-45	164	COG0515	SPS1	Serine/threonine protein kinase
LN02_06889∣LN02Chr05:1173410-1175524(+)∣576	CDD:225201	33.981	309	151	11	296	572	153	440	1.74E-45	164	COG2319	COG2319	FOG: WD40 repeat
LN02_06953∣LN02Chr05:1777317-1778316(+)∣309	CDD:225176	29.861	288	176	10	20	297	26	297	3.01E-43	147	COG2267	PldB	Lysophospholipase
LN02_07017∣LN02Chr05:1971291-1972968(-)∣378	CDD:223997	35.294	357	186	9	53	369	1	352	1.35E-103	307	COG1071	AcoA	Pyruvate/2-oxoglutarate dehydrogenase complex, dehydrogenase (E1) component, eukaryotic type, alpha subunit
LN02_07081∣LN02Chr05:2498234-2499369(+)∣333	CDD:225047	26.316	171	70	8	181	299	1	167	9.60E-15	69	COG2136	IMP4	Predicted exosome subunit/U3 small nucleolar ribonucleoprotein (snoRNP) component, contains IMP4 domain
LN02_07209∣LN02Chr05:2985693-2987952(+)∣708	CDD:225661	24.828	145	100	4	5	142	266	408	2.27E-07	50.9	COG3119	AslA	Arylsulfatase A and related enzymes
LN02_07529∣LN02Chr05:4077388-4078417(+)∣230	CDD:223711	30.457	197	131	3	33	226	33	226	1.23E-51	164	COG0638	PRE1	20S proteasome, alpha and beta subunits
LN02_07785∣LN02Chr05:5235655-5239794(-)∣1289	CDD:223605	20.302	463	328	11	11	454	1	441	5.64E-39	149	COG0531	PotE	Amino acid transporters
LN02_07849∣LN02Chr05:5488879-5490457(+)∣504	CDD:227515	28.101	516	313	9	1	504	1	470	3.60E-113	340	COG5188	PRP9	Splicing factor 3a, subunit 3
LN02_07977∣LN02Chr05:5849760-5853518(+)∣1083	CDD:224201	45.263	475	229	6	622	1079	3	463	2.40E-160	479	COG1282	PntB	NAD/NADP transhydrogenase beta subunit
LN02_07977∣LN02Chr05:5849760-5853518(+)∣1083	CDD:225826	54.247	365	158	4	79	443	1	356	2.04E-147	442	COG3288	PntA	NAD/NADP transhydrogenase alpha subunit
LN02_07977∣LN02Chr05:5849760-5853518(+)∣1083	CDD:225826	25	124	82	2	479	591	77	200	7.29E-15	74.3	COG3288	PntA	NAD/NADP transhydrogenase alpha subunit
LN02_08041∣LN02Chr06:589715-593822(+)∣1346	CDD:224054	33.175	211	122	6	90	294	16	213	5.47E-46	164	COG1131	CcmA	ABC-type multidrug transport system, ATPase component
LN02_08041∣LN02Chr06:589715-593822(+)∣1346	CDD:224054	28.92	287	182	9	706	988	4	272	6.30E-44	159	COG1131	CcmA	ABC-type multidrug transport system, ATPase component
LN02_08105∣LN02Chr06:1265888-1267015(-)∣375	CDD:224363	33.446	296	156	8	51	343	30	287	3.40E-45	154	COG1446	COG1446	Asparaginase

LN02_08233 | LN02Chr06: 1835147-1836499(-) | 195	CDD:224025	31.472	197	129	4	1	191	1	197	8.27E-50	158	COG1100	COG1100	GTPase SAR1 and related small G proteins
LN02_08745 | LN02Chr07: 222251-223357(-) | 325	CDD:223406	28.231	294	199	7	13	305	5	287	4.74E-64	202	COG0329	DapA	Dihydrodipicolinate synthase/N-acetylneuraminate lyase
LN02_08937 | LN02Chr07: 870673-871521(+) | 144	CDD:223181	42.446	139	66	4	7	144	5	130	1.29E-48	149	COG0103	RpsI	Ribosomal protein S9
LN02_09001 | LN02Chr07: 1087447-1088343(-) | 177	CDD:223619	59.341	91	33	3	38	127	117	204	5.95E-37	124	COG0545	FkpA	FKBP-type peptidyl-prolyl cis-trans isomerases 1
LN02_09065 | LN02Chr07: 1310977-1312313(+) | 394	CDD:223693	20.769	390	239	11	6	391	4	327	9.89E-45	154	COG0620	MetE	Methionine synthase II (cobalamin-independent)
LN02_09257 | LN02Chr07: 1954983-1958508(+) | 1126	CDD:223569	22.396	960	532	32	31	974	8	770	5.60E-180	544	COG0495	LeuS	Leucyl-tRNA synthetase
LN02_00106 | LN02Chr01: 499866-500352(+) | 114	CDD:224510	28.448	116	76	3	2	113	1	113	3.62E-18	70.9	COG1594	RPB9	DNA-directed RNA polymerase, subunit M/Transcription elongation factor TFIIS
LN02_00298 | LN02Chr01: 1593584-1594417(-) | 277	CDD:223698	29.362	235	129	11	29	250	1	211	4.42E-33	117	COG0625	Gst	Glutathione S-transferase
LN02_00426 | LN02Chr01: 2089783-2090549(-) | 199	CDD:224986	51.515	66	32	0	1	66	1	66	2.57E-24	88.2	COG2075	RPL24A	Ribosomal protein L24E
LN02_00554 | LN02Chr01: 2454382-2457608(-) | 1043	CDD:223627	22.811	811	337	20	199	997	331	864	4.97E-60	218	COG0553	HepA	Superfamily II DNA/RNA helicases, SNF2 family
LN02_00746 | LN02Chr01: 3227394-3231306(-) | 1278	CDD:223964	19.535	215	151	6	552	765	527	720	1.27E-09	59.6	COG1033	COG1033	Predicted exporters of the RND superfamily
LN02_00746 | LN02Chr01: 3227394-3231306(-) | 1278	CDD:223964	22.105	190	121	8	1053	1237	181	348	1.12E-04	43.4	COG1033	COG1033	Predicted exporters of the RND superfamily
LN02_01066 | LN02Chr01: 4285611-4286692(+) | 300	CDD:227412	33.333	135	83	3	161	293	91	220	2.43E-27	103	COG5080	YIP1	Rab GTPase interacting factor, Golgi membrane protein
LN02_01194 | LN02Chr01: 4826847-4827683(+) | 217	CDD:223290	25.728	206	113	10	9	198	5	186	3.73E-26	97.4	COG0212	COG0212	5-formyltetrahydrofolate cyclo-ligase
LN02_01258 | LN02Chr01: 5078367-5080291(-) | 574	CDD:225371	16.08	398	269	8	69	422	6	382	1.62E-16	78.8	COG2814	AraJ	Arabinose efflux permease
LN02_01322 | LN02Chr01: 5254655-5256193(-) | 375	CDD:223597	31.921	354	197	10	30	373	1	320	5.67E-70	219	COG0523	COG0523	Putative GTPases (G3E family)
LN02_01386 | LN02Chr01: 5539403-5540468(-) | 305	CDD:223282	27.612	134	85	4	42	175	28	149	2.38E-17	77.3	COG0204	PlsC	1-acyl-sn-glycerol-3-phosphate acyltransferase
LN02_01450 | LN02Chr01: 5871243-5873606(-) | 705	CDD:223953	29.954	651	408	19	48	693	2	609	1.64E-145	435	COG1022	FAA1	Long-chain acyl-CoA synthetases (AMP-forming)
LN02_01514 | LN02Chr01: 6048861-6053209(+) | 1426	CDD:223533	17.544	285	209	5	445	725	5	267	1.10E-06	49.1	COG0457	NrfG	FOG: TPR repeat
LN02_01578 | LN02Chr01: 6321869-6323608(-) | 520	CDD:224968	56.744	215	90	3	306	519	8	220	3.06E-98	293	COG2057	AtoA	Acyl CoA:acetate/3-ketoacid CoA transferase, beta subunit
LN02_01578 | LN02Chr01: 6321869-6323608(-) | 520	CDD:224702	44.351	239	114	2	38	276	1	220	1.76E-86	263	COG1788	AtoD	Acyl CoA:acetate/3-ketoacid CoA transferase, alpha subunit
LN02_01578 | LN02Chr01: 6321869-6323608(-) | 520	CDD:227016	24.767	537	305	24	44	519	7	505	1.10E-32	128	COG4670	COG4670	Acyl CoA:acetate/3-ketoacid CoA transferase

LN02_01706|LN02Chr01:6811497-6814197(+)|773	CDD:223589	29.956	227	143	8	389	604	3	224	1.47E-29	118	COG0515	SPS1	Serine/threonine protein kinase
LN02_01834|LN02Chr01:7175603-7176846(+)|341	CDD:225201	21.143	350	230	10	16	337	66	397	1.12E-17	80.9	COG2319	COG2319	FOG: WD40 repeat
LN02_01898|LN02Chr01:7361615-7363211(+)|443	CDD:223727	25.135	370	232	11	19	378	16	350	2.77E-33	126	COG0654	UbiH	2-polyprenyl-6-methoxyphenol hydroxylase and related FAD-dependent oxidoreductases
LN02_02026|LN02Chr02:365279-366706(-)|441	CDD:225201	19.723	289	195	7	35	315	187	446	9.42E-14	69.7	COG2319	COG2319	FOG: WD40 repeat
LN02_02090|LN02Chr02:580226-581532(+)|403	CDD:223497	34	50	30	2	40	87	2	50	1.76E-05	43.7	COG0420	SbcD	DNA repair exonuclease
LN02_02154|LN02Chr02:757954-758995(-)|329	CDD:227362	44.728	313	168	4	13	323	26	335	3.99E-114	331	COG5029	CAL1	Prenyltransferase, beta subunit
LN02_02282|LN02Chr02:1214851-1216547(-)|512	CDD:225201	37.013	308	167	10	184	483	151	439	1.85E-52	182	COG2319	COG2319	FOG: WD40 repeat
LN02_02410|LN02Chr02:1738975-1739970(-)|230	CDD:225998	29.688	64	38	1	31	94	20	76	1.00E-04	38.5	COG3467	COG3467	Predicted flavin-nucleotide-binding protein
LN02_02538|LN02Chr02:2157530-2158104(-)|160	CDD:223725	47.205	161	75	5	4	157	1	158	8.99E-67	197	COG0652	PpiB	Peptidyl-prolyl cis-trans isomerase (rotamase) - cyclophilin family
LN02_02730|LN02Chr02:2794182-2795507(+)|376	CDD:227421	23.676	321	167	7	24	344	11	253	1.50E-38	137	COG5090	TFG2	Transcription initiation factor IIF, small subunit (RAP30)
LN02_02986|LN02Chr02:3682669-3683740(-)|309	CDD:223959	36.905	252	155	4	58	306	1	251	2.00E-73	223	COG1028	FabG	Dehydrogenases with different specificities (related to short-chain alcohol dehydrogenases)
LN02_03050|LN02Chr02:3918117-3919658(+)|334	CDD:226022	36.283	339	168	7	14	331	6	317	8.25E-81	246	COG3491	PcbC	Isopenicillin N synthase and related dioxygenases
LN02_03242|LN02Chr02:4605782-4607719(-)|589	CDD:223326	20.796	452	302	17	27	468	4	409	4.38E-36	138	COG0248	GppA	Exopolyphosphatase
LN02_03306|LN02Chr02:4815960-4816757(+)|233	CDD:223743	24.375	160	97	2	40	192	79	221	1.54E-06	44.8	COG0671	PgpB	Membrane-associated phospholipid phosphatase
LN02_03370|LN02Chr02:5017717-5019387(+)|412	CDD:223698	24.607	191	133	5	12	196	11	196	1.59E-26	102	COG0625	Gst	Glutathione S-transferase
LN02_03498|LN02Chr02:5789479-5793074(+)|1153	CDD:227446	36.812	1054	520	15	4	1053	3	914	0	867	COG5116	RPN2	26S proteasome regulatory complex component
LN02_03818|LN02Chr03:830355-832230(-)|576	CDD:224265	25.397	189	114	4	357	543	57	220	8.49E-17	76.8	COG1346	LrgB	Putative effector of murein hydrolase
LN02_03946|LN02Chr03:1209371-1211005(+)|478	CDD:223552	43.75	272	133	3	58	328	2	254	1.89E-87	265	COG0476	ThiF	Dinucleotide-utilizing enzymes involved in molybdopterin and thiamine biosynthesis family 2
LN02_03946|LN02Chr03:1209371-1211005(+)|478	CDD:223680	36.842	114	57	5	357	469	2	101	4.76E-15	68.4	COG0607	PspE	Rhodanese-related sulfurtransferase
LN02_04202|LN02Chr03:3334194-3336198(+)|466	CDD:225182	27.869	122	74	3	220	341	104	211	2.33E-15	74.4	COG2273	SKN1	Beta-glucanase/Beta-glucan synthetase
LN02_04330|LN02Chr03:3824510-3827008(+)|832	CDD:225013	31.399	293	129	8	10	302	3	223	1.33E-49	171	COG2102	COG2102	Predicted ATPases of PP-loop superfamily

LN02_04330|LN02Chr03: 3824510-3827008(+)|832	CDD:223329	29.577	71	50	0	383	453	46	116	4.56E-11	58.4	COG0251	TdcF	Putative translation initiation inhibitor, yjgF family
LN02_04330|LN02Chr03: 3824510-3827008(+)|832	CDD:223329	34.884	86	31	5	486	563	12	80	4.49E-04	38	COG0251	TdcF	Putative translation initiation inhibitor, yjgF family
LN02_04522|LN02Chr03: 4430989-4433212(+)|561	CDD:223796	18.593	199	153	4	216	411	84	276	6.30E-12	63.8	COG0724	COG0724	RNA-binding proteins (RRM domain)
LN02_04522|LN02Chr03: 4430989-4433212(+)|561	CDD:223796	30.233	86	52	3	77	158	115	196	2.67E-05	43.4	COG0724	COG0724	RNA-binding proteins (RRM domain)
LN02_04650|LN02Chr03: 4923261-4926536(+)|886	CDD:223136	44.39	811	371	17	79	879	9	749	0	897	COG0058	GlgP	Glucan phosphorylase
LN02_04714|LN02Chr03: 5152212-5153665(-)|348	CDD:223959	32.453	265	161	7	86	346	1	251	5.47E-56	180	COG1028	FabG	Dehydrogenases with different specificities (related to short-chain alcohol dehydrogenases)
LN02_04778|LN02Chr03: 5373881-5375805(+)|613	CDD:223861	28.244	262	172	5	288	539	17	272	5.72E-29	113	COG0790	COG0790	FOG: TPR repeat, SEL1 subfamily
LN02_05034|LN02Chr04: 403831-405970(-)|503	CDD:223226	56.264	439	168	10	67	500	3	422	0	606	COG0148	Eno	Enolase
LN02_05098|LN02Chr04: 603017-604291(-)|391	CDD:225941	37.464	347	189	7	8	352	5	325	2.43E-95	285	COG3407	MVD1	Mevalonate pyrophosphate decarboxylase
LN02_05162|LN02Chr04: 821745-823671(-)|514	CDD:223190	43.432	472	203	7	41	511	5	413	0	582	COG0112	GlyA	Glycine/serine hydroxymethyltransferase
LN02_05290|LN02Chr04: 1215815-1217764(+)|556	CDD:223605	22.366	465	341	7	46	510	17	461	1.90E-38	144	COG0531	PotE	Amino acid transporters
LN02_05482|LN02Chr04: 2029603-2032287(-)|818	CDD:226406	23.757	181	100	7	185	339	694	862	2.07E-04	41.8	COG3889	COG3889	Predicted solute binding protein
LN02_05546|LN02Chr04: 2391299-2394186(+)|895	CDD:224162	38.744	653	329	9	35	676	15	607	0	607	COG1241	MCM2	Predicted ATPase involved in replication control, Cdc46/Mcm family
LN02_05610|LN02Chr04: 2626097-2627316(+)|354	CDD:223669	25.641	117	76	3	90	204	16	123	3.91E-07	48.1	COG0596	MhpC	Predicted hydrolases or acyltransferases (alpha/beta hydrolase superfamily)
LN02_05738|LN02Chr04: 3037557-3039099(-)|478	CDD:225779	22.162	370	222	10	77	442	14	321	9.36E-27	107	COG3239	DesA	Fatty acid desaturase
LN02_05866|LN02Chr04: 3460966-3461871(-)|301	CDD:227501	27.907	301	194	7	1	297	1	282	2.19E-48	160	COG5174	TFA2	Transcription initiation factor IIE, beta subunit
LN02_05994|LN02Chr04: 3945279-3947338(+)|532	CDD:223589	23.393	389	265	9	15	387	3	374	1.81E-46	164	COG0515	SPS1	Serine/threonine protein kinase
LN02_06058|LN02Chr04: 4142531-4146532(-)|1333	CDD:223951	34.945	455	255	10	8	430	197	642	4.66E-73	254	COG1020	EntF	Non-ribosomal peptide synthetase modules and related proteins
LN02_06186|LN02Chr04: 4606761-4608192(-)|403	CDD:225482	56.164	219	95	1	1	218	6	224	5.08E-86	258	COG2930	COG2930	Uncharacterized conserved protein
LN02_06250|LN02Chr04: 4789530-4792143(-)|785	CDD:225143	23.967	242	164	7	389	627	198	422	1.19E-09	58	COG2234	Iap	Predicted aminopeptidases
LN02_06314|LN02Chr04: 5002461-5004295(+)|557	CDD:226582	19.94	331	207	14	95	416	72	353	1.71E-10	60.2	COG4097	COG4097	Predicted ferric reductase
LN02_06442|LN02Chr04: 5399799-5401102(+)|384	CDD:223991	32.011	378	221	14	7	377	2	350	1.24E-67	214	COG1063	Tdh	Threonine dehydrogenase and related Zn-dependent dehydrogenases

LN02_06634∣LN02Chr05:181314-183567(-)∣629	CDD:223796	20.588	102	68	3	268	368	107	196	1.37E-05	44.6	COG0724	COG0724	RNA-binding proteins (RRM domain)
LN02_06698∣LN02Chr05:456891-459629(+)∣826	CDD:227873	28.829	111	65	3	153	260	7	106	2.47E-07	47.2	COG5586	COG5586	Uncharacterized conserved protein
LN02_06698∣LN02Chr05:456891-459629(+)∣826	CDD:223901	32.911	79	38	6	100	164	6	83	6.42E-04	36.9	COG0831	UreA	Urea amidohydrolase (urease) gamma subunit
LN02_06762∣LN02Chr05:722632-725691(+)∣1019	CDD:225201	19.512	369	257	11	409	775	78	408	1.81E-17	83.6	COG2319	COG2319	FOG: WD40 repeat
LN02_06762∣LN02Chr05:722632-725691(+)∣1019	CDD:225201	16.514	436	303	15	156	584	69	450	3.58E-13	70.1	COG2319	COG2319	FOG: WD40 repeat
LN02_06954∣LN02Chr05:1779201-1780661(+)∣486	CDD:223131	26.829	287	189	10	196	471	12	288	1.03E-28	112	COG0053	MMT1	Predicted Co/Zn/Cd cation transporters
LN02_07018∣LN02Chr05:1973714-1975387(-)∣557	CDD:225421	20.833	312	206	12	199	505	99	374	5.30E-21	92.2	COG2866	COG2866	Predicted carboxypeptidase
LN02_07082∣LN02Chr05:2500919-2502234(+)∣299	CDD:224013	37.815	238	105	4	88	284	1	236	3.50E-74	228	COG1088	RfbB	dTDP-D-glucose 4,6-dehydratase
LN02_07146∣LN02Chr05:2732356-2733256(-)∣234	CDD:225396	37.037	54	33	1	95	148	96	148	9.66E-06	41.9	COG2840	COG2840	Uncharacterized protein conserved in bacteria
LN02_07210∣LN02Chr05:2988698-2989423(-)∣160	CDD:226300	23.558	208	94	8	1	157	79	272	1.62E-10	54.8	COG3777	COG3777	Uncharacterized conserved protein
LN02_07338∣LN02Chr05:3380683-3383629(+)∣853	CDD:224423	24.579	297	161	9	578	821	330	616	7.19E-33	132	COG1506	DAP2	Dipeptidyl aminopeptidases/acylaminoacyl-peptidases
LN02_07658∣LN02Chr05:4752752-4753997(+)∣306	CDD:225546	25.49	255	169	5	34	285	1	237	5.26E-41	141	COG3000	ERG3	Sterol desaturase
LN02_07786∣LN02Chr05:5243005-5245353(+)∣753	CDD:223884	20.948	401	291	9	358	734	14	412	4.25E-59	203	COG0814	SdaC	Amino acid permeases
LN02_07850∣LN02Chr05:5490874-5491885(+)∣308	CDD:225383	31.081	74	44	3	9	82	2	68	1.86E-09	51.2	COG2827	COG2827	Predicted endonuclease containing a URI domain
LN02_07978∣LN02Chr05:5853925-5854622(-)∣136	CDD:226284	34.444	90	52	4	29	116	10	94	2.26E-09	49.2	COG3761	COG3761	NADH:ubiquinone oxidoreductase 17.2 kD subunit
LN02_08234∣LN02Chr06:1837792-1839047(+)∣368	CDD:223276	29.231	65	39	2	119	178	9	71	1.41E-04	37.7	COG0198	RplX	Ribosomal protein L24
LN02_08490∣LN02Chr06:2782579-2783859(-)∣426	CDD:223220	24.373	279	196	6	130	398	24	297	4.49E-47	161	COG0142	IspA	Geranylgeranyl pyrophosphate synthase
LN02_08810∣LN02Chr07:419609-421927(-)∣731	CDD:227381	43.077	65	34	1	149	210	31	95	6.93E-06	46.2	COG5048	COG5048	FOG: Zn-finger
LN02_09002∣LN02Chr07:1089569-1092436(-)∣589	CDD:224779	53.592	515	230	8	74	587	20	526	0	747	COG1866	PckA	Phosphoenolpyruvate carboxykinase (ATP)
LN02_09194∣LN02Chr07:1709377-1710720(+)∣353	CDD:223632	31.034	116	71	3	58	166	15	128	7.75E-22	89	COG0558	PgsA	Phosphatidylglycerophosphate synthase
LN02_09322∣LN02Chr07:2202661-2204244(-)∣510	CDD:225371	24.265	136	99	2	69	204	51	182	1.54E-07	50.3	COG2814	AraJ	Arabinose efflux permease
LN02_09322∣LN02Chr07:2202661-2204244(-)∣510	CDD:223553	17.123	292	227	2	69	358	42	320	2.10E-06	46.6	COG0477	ProP	Permeases of the major facilitator superfamily

LN02_00299|LN02Chr01:1595689-1597423(+)|520	CDD:223510	21.429	126	79	5	337	457	393	503	4.55E-04	39.6	COG0433	COG0433	HerA helicase

LN02_00491|LN02Chr01:2281833-2282816(+)|260	CDD:224146	35.484	124	72	2	144	259	33	156	1.90E-32	113	COG1225	Bcp	Peroxiredoxin
LN02_00811|LN02Chr01:3479490-3481198(+)|518	CDD:225459	38.393	448	248	12	8	454	6	426	1.59E-126	374	COG2907	COG2907	Predicted NAD/FAD-binding protein
LN02_00875|LN02Chr01:3657828-3659579(-)|515	CDD:227187	22.705	207	139	9	233	423	114	315	4.76E-15	73.7	COG4850	COG4850	Uncharacterized conserved protein
LN02_01003|LN02Chr01:4069317-4070672(-)|451	CDD:223162	19.343	274	112	10	183	446	81	255	1.80E-18	81.9	COG0084	TatD	Mg-dependent DNase
LN02_01067|LN02Chr01:4288057-4289773(+)|484	CDD:227499	25	128	65	4	308	433	55	153	7.29E-06	43.9	COG5171	YRB1	Ran GTPase-activating protein (Ran-binding protein)
LN02_01259|LN02Chr01:5082541-5083435(+)|273	CDD:226275	30.088	113	66	5	161	265	153	260	6.61E-08	49.3	COG3752	COG3752	Steroid 5-alpha reductase family enzyme
LN02_01707|LN02Chr01:6815018-6816132(+)|144	CDD:223312	51.579	95	46	0	50	144	2	96	3.91E-40	127	COG0234	GroS	Co-chaperonin GroES (HSP10)
LN02_02411|LN02Chr02:1740745-1743214(+)|736	CDD:227111	45.39	705	318	12	32	730	2	645	0	661	COG4770	COG4770	Acetyl/propionyl-CoA carboxylase, alpha subunit
LN02_02475|LN02Chr02:1945792-1948963(-)|874	CDD:227540	38.178	867	522	10	1	865	1	855	0	764	COG5215	KAP95	Karyopherin (importin) beta
LN02_02731|LN02Chr02:2796002-2796946(+)|314	CDD:223634	23.105	277	126	16	26	291	9	209	3.60E-04	38	COG0560	SerB	Phosphoserine phosphatase
LN02_02859|LN02Chr02:3240548-3242374(-)|528	CDD:225035	21.739	460	284	12	72	527	23	410	2.92E-55	188	COG2124	CypX	Cytochrome P450
LN02_02987|LN02Chr02:3685477-3685834(+)|74	CDD:226252	38.983	59	32	1	15	73	8	62	5.53E-04	32.1	COG3729	GsiB	General stress protein
LN02_03051|LN02Chr02:3920225-3921634(+)|442	CDD:225714	25	108	63	6	158	254	123	223	0.001	38.2	COG3173	COG3173	Predicted aminoglycoside phosphotransferase
LN02_03499|LN02Chr02:5794074-5796119(-)|681	CDD:224952	28.054	221	123	8	260	475	64	253	1.38E-26	106	COG2041	COG2041	Sulfite oxidase and related enzymes
LN02_03499|LN02Chr02:5794074-5796119(-)|681	CDD:227599	27.711	83	58	2	133	215	45	125	3.43E-08	50.6	COG5274	CYB5	Cytochrome b involved in lipid metabolism
LN02_03563|LN02Chr02:6218912-6221263(-)|655	CDD:225805	28.205	78	49	2	455	525	114	191	1.19E-04	41.5	COG3266	DamX	Uncharacterized protein conserved in bacteria
LN02_04011|LN02Chr03:1440891-1441673(-)|260	CDD:223440	34.387	253	143	7	7	253	2	237	6.67E-64	197	COG0363	NagB	6-phosphogluconolactonase/Glucosamine-6-phosphate isomerase/deaminase
LN02_04139|LN02Chr03:2787069-2787581(-)|149	CDD:227461	55.034	149	64	3	1	149	1	146	5.56E-63	187	COG5132	BUD31	Cell cycle control protein, G10 family
LN02_04267|LN02Chr03:3601067-3601924(-)|285	CDD:223565	25.862	232	138	5	13	212	6	235	2.11E-25	98.7	COG0491	GloB	Zn-dependent hydrolases, including glyoxylases
LN02_04331|LN02Chr03:3827581-3828853(+)|311	CDD:225060	30.435	115	66	3	190	304	17	117	4.54E-13	62.1	COG2149	COG2149	Predicted membrane protein

LN02_04523ǀLN02Chr03:4434100-4436658(+)ǀ522	CDD:223664	22.814	263	188	7	13	260	5	267	3.11E-14	71.9	COG0591	PutP	Na+/proline symporter
LN02_04587ǀLN02Chr03:4702003-4705554(+)ǀ1084	CDD:223589	27.707	314	179	9	271	569	1	281	3.50E-48	173	COG0515	SPS1	Serine/threonine protein kinase
LN02_04651ǀLN02Chr03:4931393-4933074(+)ǀ327	CDD:223589	21.875	320	180	8	20	290	2	300	1.61E-29	113	COG0515	SPS1	Serine/threonine protein kinase
LN02_04715ǀLN02Chr03:5155787-5157215(-)ǀ441	CDD:224871	43.99	391	210	7	58	440	3	392	8.30E-115	339	COG1960	CaiA	Acyl-CoA dehydrogenases
LN02_04779ǀLN02Chr03:5376773-5377493(-)ǀ187	CDD:225660	30.263	76	43	3	66	140	41	107	2.10E-06	43.9	COG3118	COG3118	Thioredoxin domain-containing protein
LN02_04971ǀLN02Chr04:178250-180400(+)ǀ643	CDD:226406	22.527	182	110	8	372	538	694	859	1.40E-04	41.8	COG3889	COG3889	Predicted solute binding protein
LN02_05035ǀLN02Chr04:406831-408890(-)ǀ392	CDD:223215	40.16	376	201	9	23	384	36	401	2.39E-139	400	COG0137	ArgG	Argininosuccinate synthase
LN02_05099ǀLN02Chr04:605963-606849(-)ǀ162	CDD:223709	44.737	76	38	1	85	156	3	78	6.45E-11	52.6	COG0636	AtpE	F0F1-type ATP synthase, subunit c/Archaeal/vacuolar-type H+-ATPase, subunit K
LN02_05227ǀLN02Chr04:1033055-1034403(+)ǀ354	CDD:225201	34.951	309	183	11	56	354	59	359	7.56E-40	144	COG2319	COG2319	FOG: WD40 repeat
LN02_05355ǀLN02Chr04:1474471-1475975(+)ǀ367	CDD:223484	42.857	364	195	6	4	366	1	352	4.88E-139	396	COG0407	HemE	Uroporphyrinogen-III decarboxylase
LN02_05547ǀLN02Chr04:2394593-2396052(-)ǀ442	CDD:224117	16.667	114	83	2	182	293	749	852	2.25E-04	40.5	COG1196	Smc	Chromosome segregation ATPases
LN02_05611ǀLN02Chr04:2627580-2628719(-)ǀ328	CDD:227640	36.242	149	93	1	47	193	44	192	1.02E-29	112	COG5333	CCL1	Cdk activating kinase (CAK)/RNA polymerase II transcription initiation/nucleotide excision repair factor TFIIH/TFIIK, cyclin H subunit
LN02_05675ǀLN02Chr04:2822572-2824289(-)ǀ554	CDD:223553	18.625	349	268	5	67	412	3	338	2.64E-06	46.6	COG0477	ProP	Permeases of the major facilitator superfamily
LN02_05803ǀLN02Chr04:3242845-3244530(-)ǀ522	CDD:224105	23.009	339	203	13	177	510	13	298	7.58E-19	84.3	COG1184	GCD2	Translation initiation factor 2B subunit, eIF-2B alpha/beta/delta family
LN02_05803ǀLN02Chr04:3242845-3244530(-)ǀ522	CDD:223568	21.739	115	85	2	48	162	43	152	1.12E-05	42.9	COG0494	MutT	NTP pyrophosphohydrolases including oxidative damage repair enzymes
LN02_05867ǀLN02Chr04:3462812-3463691(-)ǀ141	CDD:227599	41.772	79	46	0	3	81	48	126	7.37E-27	95.7	COG5274	CYB5	Cytochrome b involved in lipid metabolism
LN02_05995ǀLN02Chr04:3947455-3947971(-)ǀ96	CDD:227521	48.913	92	40	3	1	89	1	88	5.23E-28	94.5	COG5194	APC11	Component of SCF ubiquitin ligase and anaphase-promoting complex
LN02_06123ǀLN02Chr04:4346503-4346622(-)ǀ39	CDD:223589	27.027	37	27	0	1	37	243	279	6.67E-05	34.3	COG0515	SPS1	Serine/threonine protein kinase
LN02_06251ǀLN02Chr04:4793842-4796086(+)ǀ557	CDD:225371	24.699	166	119	3	6	170	2	162	1.75E-12	66.1	COG2814	AraJ	Arabinose efflux permease
LN02_06251ǀLN02Chr04:4793842-4796086(+)ǀ557	CDD:225121	19.78	182	128	4	324	490	256	434	4.31E-05	43	COG2211	MelB	Na+/melibiose symporter and related transporters

LN02_06315|LN02Chr04:5005130-5009576(-)|1381	CDD:223715	38.261	230	127	5	804	1032	113	328	1.32E-63	217	COG0642	BaeS	Signal transduction histidine kinase
LN02_06315|LN02Chr04:5005130-5009576(-)|1381	CDD:223855	34.307	137	73	4	1107	1237	2	127	2.58E-29	111	COG0784	CheY	FOG: CheY-like receiver
LN02_06315|LN02Chr04:5005130-5009576(-)|1381	CDD:225112	24.18	244	165	6	391	631	1	227	1.18E-16	78	COG2202	AtoS	FOG: PAS/PAC domain
LN02_06315|LN02Chr04:5005130-5009576(-)|1381	CDD:225112	21.429	252	177	6	529	779	1	232	2.62E-11	62.2	COG2202	AtoS	FOG: PAS/PAC domain
LN02_06379|LN02Chr04:5207050-5209502(+)|764	CDD:226119	24.268	239	129	15	45	269	36	236	8.20E-09	54.3	COG3591	COG3591	V8-like Glu-specific endopeptidase
LN02_06443|LN02Chr04:5401440-5402849(-)|309	CDD:223729	38.188	309	153	8	5	308	5	280	2.66E-101	295	COG0656	ARA1	Aldo/keto reductases, related to diketogulonate reductase
LN02_06507|LN02Chr04:5688443-5689394(-)|258	CDD:223959	30.224	268	131	7	13	243	1	249	9.72E-35	122	COG1028	FabG	Dehydrogenases with different specificities (related to short-chain alcohol dehydrogenases)
LN02_06827|LN02Chr05:939100-941334(-)|564	CDD:223796	21.495	214	157	3	66	277	101	305	4.82E-11	61.1	COG0724	COG0724	RNA-binding proteins (RRM domain)
LN02_06891|LN02Chr05:1177668-1180158(+)|575	CDD:223884	17.886	123	100	1	247	368	4	126	1.21E-14	73.1	COG0814	SdaC	Amino acid permeases
LN02_06955|LN02Chr05:1781918-1783743(-)|547	CDD:227352	24.651	215	129	10	117	323	10	199	1.91E-08	53.5	COG5019	CDC3	Septin family protein
LN02_07083|LN02Chr05:2505378-2506670(+)|370	CDD:225136	22.549	204	124	8	107	287	35	227	5.68E-12	61.9	COG2226	UbiE	Methylase involved in ubiquinone/menaquinone biosynthesis
LN02_07211|LN02Chr05:2990909-2993330(+)|792	CDD:224418	28.06	531	321	19	119	616	202	704	2.16E-104	335	COG1501	COG1501	Alpha-glucosidases, family 31 of glycosyl hydrolases
LN02_07659|LN02Chr05:4754528-4755204(-)|174	CDD:225065	37.5	80	50	0	63	142	19	98	9.61E-27	94.7	COG2154	COG2154	Pterin-4a-carbinolamine dehydratase
LN02_07787|LN02Chr05:5247583-5248870(+)|408	CDD:223617	26.087	92	66	1	140	231	61	150	1.08E-11	61.7	COG0543	UbiB	2-polyprenylphenol hydroxylase and related flavodoxin oxidoreductases
LN02_07851|LN02Chr05:5493415-5499005(+)|1749	CDD:223098	28.395	81	51	5	1026	1104	66	141	3.82E-04	41.9	COG0019	LysA	Diaminopimelate decarboxylase
LN02_07851|LN02Chr05:5493415-5499005(+)|1749	CDD:227367	17.844	269	192	10	1111	1371	20	267	5.89E-04	40.7	COG5034	TNG2	Chromatin remodeling protein, contains PhD zinc finger
LN02_07979|LN02Chr05:5854853-5856575(+)|525	CDD:224419	19.651	458	283	19	68	519	57	435	5.18E-33	128	COG1502	Cls	Phosphatidylserine/phosphatidylglycerophosphate/cardiolipin synthases and related enzymes
LN02_08235|LN02Chr06:1839752-1840768(+)|272	CDD:223455	47.692	195	90	7	51	242	12	197	8.85E-71	214	COG0378	HypB	Ni2+-binding GTPase involved in regulation of expression and maturation of urease and hydrogenase
LN02_08427|LN02Chr06:2576849-2580026(+)|963	CDD:224117	19.196	224	169	4	679	896	269	486	4.87E-10	60.5	COG1196	Smc	Chromosome segregation ATPases
LN02_08619|LN02Chr06:3177850-3179727(+)|509	CDD:223443	18.493	438	278	19	28	449	28	402	1.63E-16	78.9	COG0366	AmyA	Glycosidases

LN02_09003	LN02Chr07 :1096894-1099199(-)	713	CDD:227581	44.316	431	229	6	279	707	5	426	0	520	COG5256	TEF1	Translation elongation factor EF-1alpha (GTPase)
LN02_09067	LN02Chr07 :1314369-1315758(+)	348	CDD:227385	34.409	93	61	0	5	97	58	150	3.06E-15	70.2	COG5052	YOP1	Protein involved in membrane traffic
LN02_09195	LN02Chr07 :1711428-1713487(-)	537	CDD:225043	22.922	397	249	11	44	436	107	450	4.86E-62	207	COG2132	SufI	Putative multicopper oxidases
LN02_09259	LN02Chr07 :1963871-1964421(-)	87	CDD:227541	53.846	65	30	0	7	71	1	65	8.41E-28	93	COG5216	COG5216	Uncharacterized conserved protein
LN02_00236	LN02Chr01 :1424692-1425883(+)	377	CDD:225606	33.654	104	62	3	170	273	60	156	6.77E-05	41.5	COG3064	TolA	Membrane protein involved in colicin uptake
LN02_00620	LN02Chr01 :2737037-2741332(+)	1348	CDD:226406	23.757	181	100	7	267	421	694	862	2.26E-04	42.5	COG3889	COG3889	Predicted solute binding protein
LN02_00684	LN02Chr01 :3032461-3033415(+)	289	CDD:225182	20.522	268	159	15	28	273	17	252	2.60E-09	54.4	COG2273	SKN1	Beta-glucanase/Beta-glucan synthetase
LN02_00812	LN02Chr01 :3482010-3483092(+)	203	CDD:225716	48.677	189	88	1	5	193	3	182	7.82E-84	244	COG3175	COX11	Cytochrome oxidase assembly factor
LN02_01260	LN02Chr01 :5084405-5086030(-)	513	CDD:225136	20.982	224	122	13	304	497	27	225	1.66E-04	40.3	COG2226	UbiE	Methylase involved in ubiquinone/menaquinone biosynthesis
LN02_01836	LN02Chr01 :7178552-7179875(+)	368	CDD:226022	19.907	216	103	10	133	342	123	274	2.13E-08	52	COG3491	PcbC	Isopenicillin N synthase and related dioxygenases
LN02_01964	LN02Chr02 :127943-129557(-)	472	CDD:223449	26.161	409	269	10	63	470	9	385	1.05E-96	294	COG0372	GltA	Citrate synthase
LN02_02156	LN02Chr02 :765052-765651(-)	199	CDD:227550	44.937	158	75	4	10	158	1	155	4.01E-40	132	COG5225	RRS1	Uncharacterized protein involved in ribosome biogenesis
LN02_02220	LN02Chr02 :971553-972288(-)	151	CDD:223262	25	104	62	1	44	147	1	88	5.45E-28	96.5	COG0184	RpsO	Ribosomal protein S15P/S13E
LN02_02284	LN02Chr02 :1223314-1224464(+)	339	CDD:223268	26.48	321	173	13	11	314	1	275	7.29E-50	165	COG0190	FolD	5,10-methylene-tetrahydrofolate dehydrogenase/Methenyl tetrahydrofolate cyclohydrolase
LN02_02348	LN02Chr02 :1491497-1492699(-)	349	CDD:225002	23.626	182	107	6	105	286	15	164	2.18E-17	77	COG2091	Sfp	Phosphopantetheinyl transferase
LN02_02604	LN02Chr02 :2392251-2392976(-)	198	CDD:223132	37.989	179	76	2	12	190	9	152	2.03E-56	172	COG0054	RibH	Riboflavin synthase beta-chain
LN02_02668	LN02Chr02 :2609629-2610977(+)	376	CDD:223528	18.302	377	226	13	8	371	3	310	5.14E-23	95.4	COG0451	WcaG	Nucleoside-diphosphate-sugar epimerases
LN02_02732	LN02Chr02 :2797331-2799947(-)	677	CDD:225922	27.011	659	405	21	20	667	12	605	1.12E-119	367	COG3387	SGA1	Glucoamylase and related glycosyl hydrolases
LN02_02796	LN02Chr02 :3024051-3024728(+)	193	CDD:223827	43.609	133	72	2	50	179	9	141	1.12E-38	127	COG0756	Dut	dUTPase
LN02_02924	LN02Chr02 :3480274-3481806(+)	476	CDD:224117	19.328	119	89	2	44	155	279	397	4.32E-04	39.7	COG1196	Smc	Chromosome segregation ATPases
LN02_02988	LN02Chr02 :3686689-3687867(+)	392	CDD:227578	23.757	181	95	8	107	268	372	528	1.88E-09	56.1	COG5253	MSS4	Phosphatidylinositol-4-phosphate 5-kinase

LN02_03052∣LN02Chr02:3921969-3922964(+)∣218	CDD:227525	33.166	199	132	1	14	212	5	202	9.98E-44	143	COG5198	Ptpl	Protein tyrosine phosphatase-like protein (contains Pro instead of catalytic Arg)
LN02_03180∣LN02Chr02:4412518-4412959(+)∣86	CDD:224869	38.028	71	40	1	17	83	8	78	7.50E-19	70.8	COG1958	LSM1	Small nuclear ribonucleoprotein (snRNP) homolog
LN02_03564∣LN02Chr02:6227660-6228961(+)∣433	CDD:223727	24.808	391	249	14	18	403	4	354	1.08E-24	102	COG0654	UbiH	2-polyprenyl-6-methoxyphenol hydroxylase and related FAD-dependent oxidoreductases
LN02_03628∣LN02Chr03:47315-49342(-)∣641	CDD:224136	16.257	529	327	17	119	630	4	433	3.02E-13	69.2	COG1215	COG1215	Glycosyltransferases, probably involved in cell wall biogenesis
LN02_03692∣LN02Chr03:325611-329104(-)∣1137	CDD:223715	24.863	366	158	6	534	898	87	336	6.66E-38	142	COG0642	BaeS	Signal transduction histidine kinase
LN02_03692∣LN02Chr03:325611-329104(-)∣1137	CDD:223855	35.772	123	72	4	1006	1126	6	123	6.86E-29	109	COG0784	CheY	FOG: CheY-like receiver
LN02_03884∣LN02Chr03:1012567-1014663(-)∣648	CDD:227470	33.333	45	28	1	147	189	196	240	5.52E-07	49.6	COG5141	COG5141	PHD zinc finger-containing protein
LN02_03884∣LN02Chr03:1012567-1014663(-)∣648	CDD:227568	46.154	26	14	0	73	98	318	343	3.11E-05	43.8	COG5243	HRD1	HRD ubiquitin ligase complex, ER membrane component
LN02_04332∣LN02Chr03:3829719-3832201(+)∣688	CDD:224037	31.732	583	314	16	147	686	218	759	3.04E-95	308	COG1112	COG1112	Superfamily I DNA and RNA helicases and helicase subunits
LN02_04716∣LN02Chr03:5158015-5160053(+)∣572	CDD:227136	41.575	546	281	9	45	572	1	526	0	623	COG4799	COG4799	Acetyl-CoA carboxylase, carboxyltransferase component (subunits alpha and beta)
LN02_04780∣LN02Chr03:5378624-5381257(+)∣833	CDD:225371	24.752	202	138	6	158	353	17	210	1.91E-14	73	COG2814	AraJ	Arabinose efflux permease
LN02_04844∣LN02Chr03:5675718-5678819(-)∣890	CDD:226406	22.051	195	115	7	248	431	694	862	1.26E-04	42.5	COG3889	COG3889	Predicted solute binding protein
LN02_05036∣LN02Chr04:409733-410650(-)∣257	CDD:224019	37.64	178	110	1	79	256	8	184	3.97E-70	211	COG1094	COG1094	Predicted RNA-binding protein (contains KH domains)
LN02_05100∣LN02Chr04:607945-610926(+)∣945	CDD:227637	30.989	455	235	9	230	673	116	502	1.16E-80	270	COG5329	COG5329	Phosphoinositide polyphosphatase (Sac family)
LN02_05164∣LN02Chr04:824874-826274(-)∣247	CDD:225058	51.007	149	73	0	60	208	1	149	2.73E-54	169	COG2147	RPL19A	Ribosomal protein L19E
LN02_05292∣LN02Chr04:1223760-1226621(+)∣953	CDD:223809	22.65	468	289	11	425	886	20	420	3.50E-37	142	COG0738	FucP	Fucose permease
LN02_05420∣LN02Chr04:1690648-1692730(+)∣598	CDD:223757	38.225	293	172	6	6	295	5	291	8.80E-91	279	COG0685	MetF	5,10-methylenetetrahydrofolate reductase
LN02_05612∣LN02Chr04:2629553-2632229(+)∣510	CDD:227455	18.571	140	102	5	25	155	19	155	4.85E-10	55.4	COG5126	FRQ1	Ca2+-binding protein (EF-Hand superfamily)
LN02_05740∣LN02Chr04:3043881-3046157(-)∣712	CDD:227535	25.869	259	168	9	417	668	186	427	2.31E-21	95.3	COG5210	COG5210	GTPase-activating protein
LN02_05996∣LN02Chr04:3948758-3949853(+)∣337	CDD:225450	35.254	295	164	10	33	326	9	277	1.83E-83	251	COG2897	SseA	Rhodanese-related sulfurtransferase
LN02_06188∣LN02Chr04:4610291-4612130(-)∣585	CDD:227651	39.231	130	72	2	21	144	12	140	5.05E-45	159	COG5347	COG5347	GTPase-activating protein that regulates ARFs (ADP-ribosylation factors),

														involved in ARF-mediated vesicular transport
LN02_06636|LN02Chr05:188104-190652(-)|795	CDD:225491	30.323	155	88	6	459	599	329	477	1.89E-15	76.8	COG2940	COG2940	Proteins containing SET domain
LN02_06700|LN02Chr05:464752-465258(+)|168	CDD:223195	32.374	139	70	8	13	148	10	127	3.68E-25	91.9	COG0117	RibD	Pyrimidine deaminase
LN02_06764|LN02Chr05:730437-731997(-)|479	CDD:223515	23.75	400	269	7	7	392	2	379	3.20E-49	170	COG0438	RfaG	Glycosyltransferase
LN02_06892|LN02Chr05:1180862-1182709(-)|615	CDD:223589	29.766	299	180	7	18	293	2	293	8.76E-49	172	COG0515	SPS1	Serine/threonine protein kinase
LN02_06956|LN02Chr05:1784597-1785657(+)|326	CDD:223589	30.284	317	189	8	9	312	1	298	3.73E-58	189	COG0515	SPS1	Serine/threonine protein kinase
LN02_07340|LN02Chr05:3391547-3392515(-)|277	CDD:223669	22.124	226	165	5	29	250	19	237	2.48E-14	68.5	COG0596	MhpC	Predicted hydrolases or acyltransferases (alpha/beta hydrolase superfamily)
LN02_07404|LN02Chr05:3637065-3640308(+)|1022	CDD:223589	24.653	288	186	7	53	324	4	276	9.91E-34	131	COG0515	SPS1	Serine/threonine protein kinase
LN02_07468|LN02Chr05:3876435-3877455(+)|315	CDD:227428	25.359	209	123	6	5	212	2	178	1.26E-25	98.9	COG5097	MED6	RNA polymerase II transcriptional regulation mediator
LN02_07532|LN02Chr05:4088089-4089652(-)|452	CDD:224919	35.754	358	201	11	88	432	1	342	1.54E-86	265	COG2008	GLY1	Threonine aldolase
LN02_07788|LN02Chr05:5249117-5253263(-)|1301	CDD:223550	18.565	1185	637	32	132	1295	9	886	8.61E-141	449	COG0474	MgtA	Cation transport ATPase
LN02_07852|LN02Chr05:5500148-5501210(-)|300	CDD:223379	57.778	180	76	0	117	296	14	193	5.53E-104	299	COG0302	FolE	GTP cyclohydrolase I
LN02_07980|LN02Chr05:5856876-5857937(-)|316	CDD:223166	33.816	207	111	5	90	293	29	212	1.01E-35	125	COG0088	RplD	Ribosomal protein L4
LN02_08236|LN02Chr06:1841102-1843698(-)|838	CDD:227504	32.555	814	495	14	8	815	3	768	0	602	COG5177	COG5177	Uncharacterized conserved protein
LN02_08364|LN02Chr06:2249789-2254527(+)|1539	CDD:227623	34.783	115	75	0	772	886	311	425	9.46E-28	119	COG5307	COG5307	SEC7 domain proteins
LN02_08428|LN02Chr06:2582257-2584724(-)|626	CDD:224216	19.623	637	417	18	3	625	68	623	6.43E-54	191	COG1297	COG1297	Predicted membrane protein
LN02_08876|LN02Chr07:653860-655689(+)|502	CDD:227353	41.388	389	156	5	116	502	77	395	6.03E-147	424	COG5020	KTR1	Mannosyltransferase
LN02_08940|LN02Chr07:897950-900625(-)|555	CDD:223587	38.386	409	245	4	109	515	2	405	8.08E-151	439	COG0513	SrmB	Superfamily II DNA and RNA helicases
LN02_09132|LN02Chr07:1532884-1535115(-)|486	CDD:224106	23.188	138	76	5	192	327	515	624	2.37E-04	40.6	COG1185	Pnp	Polyribonucleotide nucleotidyltransferase (polynucleotide phosphorylase)
LN02_09196|LN02Chr07:1715972-1717344(+)|361	CDD:223744	26.23	366	235	10	1	351	1	346	3.19E-51	172	COG0672	FTR1	High-affinity Fe2+/Pb2+ permease

LN02_09260∣LN02Chr07:1965034-1967506(+)∣736	CDD:226646	23.862	637	403	14	95	706	22	601	2.33E-98	313	COG4178	COG4178	ABC-type uncharacterized transport system, permease and ATPase components
LN02_00045∣LN02Chr01:315250-316722(+)∣490	CDD:224983	35.104	433	265	6	1	430	1	420	3.32E-103	313	COG2072	TrkA	Predicted flavoprotein involved in K+ transport
LN02_00109∣LN02Chr01:505174-507023(+)∣561	CDD:225371	19.328	238	173	5	62	295	16	238	4.16E-07	49.1	COG2814	AraJ	Arabinose efflux permease
LN02_00237∣LN02Chr01:1427039-1429345(+)∣750	CDD:225201	21.111	360	257	11	397	749	58	397	1.09E-18	86.7	COG2319	COG2319	FOG: WD40 repeat
LN02_00621∣LN02Chr01:2742171-2743945(-)∣513	CDD:226406	27.059	85	56	2	296	379	768	847	0.001	39.1	COG3889	COG3889	Predicted solute binding protein
LN02_00685∣LN02Chr01:3033873-3035123(-)∣319	CDD:223894	24.286	70	40	2	161	228	20	78	9.62E-06	41.8	COG0824	FcbC	Predicted thioesterase
LN02_00749∣LN02Chr01:3235944-3237620(-)∣487	CDD:224806	54.588	425	185	5	38	460	6	424	0	651	COG1894	NuoF	NADH:ubiquinone oxidoreductase, NADH-binding (51 kD) subunit
LN02_00813∣LN02Chr01:3483696-3484250(+)∣136	CDD:223276	28.205	78	47	2	46	114	1	78	1.32E-11	54.6	COG0198	RplX	Ribosomal protein L24
LN02_00941∣LN02Chr01:3847325-3848785(+)∣486	CDD:224419	19.762	420	213	13	63	462	75	390	3.13E-15	74.5	COG1502	Cls	Phosphatidylserine/phosphatidylglycerophosphate/cardiolipin synthases and related enzymes
LN02_01197∣LN02Chr01:4840915-4842003(-)∣210	CDD:224025	31.953	169	91	3	23	167	18	186	5.73E-30	107	COG1100	COG1100	GTPase SAR1 and related small G proteins
LN02_01261∣LN02Chr01:5087152-5089790(+)∣710	CDD:225043	18.121	596	323	18	56	644	5	442	9.87E-29	117	COG2132	SufI	Putative multicopper oxidases
LN02_01325∣LN02Chr01:5269239-5273070(-)∣1180	CDD:224117	24.535	1182	835	22	1	1164	1	1143	0	577	COG1196	Smc	Chromosome segregation ATPases
LN02_01453∣LN02Chr01:5878480-5880252(+)∣436	CDD:225428	65.734	429	144	3	1	429	1	426	0	709	COG2873	MET17	O-acetylhomoserine sulfhydrylase
LN02_01517∣LN02Chr01:6055681-6056786(-)∣302	CDD:223404	30.502	259	154	7	22	277	6	241	7.58E-54	173	COG0327	COG0327	Uncharacterized conserved protein
LN02_01645∣LN02Chr01:6571031-6571441(+)∣136	CDD:223243	32.593	135	89	1	1	135	309	441	7.87E-27	100	COG0165	ArgH	Argininosuccinate lyase
LN02_01709∣LN02Chr01:6819570-6820671(+)∣203	CDD:224025	33.508	191	109	3	11	183	6	196	5.36E-45	146	COG1100	COG1100	GTPase SAR1 and related small G proteins
LN02_01773∣LN02Chr01:6984367-6985265(+)∣255	CDD:227386	44.037	218	99	3	38	255	23	217	2.72E-73	220	COG5053	CDC33	Translation initiation factor 4E (eIF-4E)
LN02_01837∣LN02Chr01:7180129-7181797(-)∣487	CDD:223505	20.37	324	179	10	163	476	11	265	6.03E-09	53.9	COG0428	COG0428	Predicted divalent heavy-metal cations transporter
LN02_01965∣LN02Chr02:130567-132512(+)∣601	CDD:225134	35.835	533	233	8	73	600	5	433	2.22E-172	493	COG2224	AceA	Isocitrate lyase
LN02_02093∣LN02Chr02:594083-596122(-)∣558	CDD:224666	44.231	52	29	0	182	233	14	65	1.50E-13	68.6	COG1752	RssA	Predicted esterase of the alpha-beta hydrolase superfamily
LN02_02221∣LN02Chr02:973096-974975(-)∣559	CDD:223796	20.354	226	163	4	284	502	43	258	2.02E-12	65.4	COG0724	COG0724	RNA-binding proteins (RRM domain)

LN02_02221 | LN02Chr02 :973096-974975(-) | 559	CDD:223796	28.358	67	41	2	484	548	130	191	2.91E-04	39.9	COG0724	COG0724	RNA-binding proteins (RRM domain)
LN02_02221 | LN02Chr02 :973096-974975(-) | 559	CDD:223796	21.678	143	104	3	162	303	48	183	3.24E-04	39.9	COG0724	COG0724	RNA-binding proteins (RRM domain)
LN02_02349 | LN02Chr02 :1493312-1496038(-) | 761	CDD:223520	27.928	222	122	11	193	413	2	186	3.33E-10	60	COG0443	DnaK	Molecular chaperone
LN02_02477 | LN02Chr02 :1951966-1953821(-) | 575	CDD:223197	29.952	414	247	8	4	413	24	398	1.97E-116	348	COG0119	LeuA	Isopropylmalate/homocitrate/citramalate synthases
LN02_02541 | LN02Chr02 :2162346-2164271(-) | 553	CDD:227359	37.397	484	255	12	1	483	15	451	1.08E-125	374	COG5026	COG5026	Hexokinase
LN02_02605 | LN02Chr02 :2393767-2396505(+) | 912	CDD:223400	25	444	282	12	2	434	22	425	8.75E-60	212	COG0323	MutL	DNA mismatch repair enzyme (predicted ATPase)
LN02_02733 | LN02Chr02 :2802500-2804441(+) | 596	CDD:223769	15.814	215	161	5	313	523	89	287	1.12E-10	59.9	COG0697	RhaT	Permeases of the drug/metabolite transporter (DMT) superfamily
LN02_02797 | LN02Chr02 :3025104-3026541(-) | 347	CDD:225149	34.948	289	150	8	6	292	1	253	1.76E-72	223	COG2240	PdxK	Pyridoxal/pyridoxine/pyridoxamine kinase
LN02_03117 | LN02Chr02 :4144908-4148313(-) | 1058	CDD:224117	21.032	252	174	5	576	821	259	491	3.65E-09	57.8	COG1196	Smc	Chromosome segregation ATPases
LN02_03181 | LN02Chr02 :4413072-4414335(-) | 290	CDD:225136	32.773	119	71	3	45	158	50	164	1.72E-17	76.9	COG2226	UbiE	Methylase involved in ubiquinone/menaquinone biosynthesis
LN02_03373 | LN02Chr02 :5022852-5024996(-) | 714	CDD:223733	29.062	437	297	8	277	704	8	440	3.45E-87	280	COG0661	AarF	Predicted unusual protein kinase
LN02_03501 | LN02Chr02 :5801570-5802313(+) | 158	CDD:223734	24.706	85	58	1	50	134	34	112	3.31E-11	54.7	COG0662	{ManC	Mannose-6-phosphate isomerase
LN02_03629 | LN02Chr03 :50700-52004(-) | 415	CDD:223798	29.358	218	131	8	116	327	66	266	9.66E-32	118	COG0726	CDA1	Predicted xylanase/chitin deacetylase
LN02_03693 | LN02Chr03 :331873-332833(-) | 266	CDD:226146	25.463	216	134	9	43	252	17	211	4.56E-11	58.1	COG3619	COG3619	Predicted membrane protein
LN02_03757 | LN02Chr03 :569064-572065(-) | 963	CDD:226406	24.545	110	72	4	277	376	739	847	2.28E-04	42.2	COG3889	COG3889	Predicted solute binding protein
LN02_04013 | LN02Chr03 :1446474-1448848(-) | 713	CDD:227526	37.313	134	79	4	10	142	1	130	1.56E-33	123	COG5199	SCP1	Calponin
LN02_04141 | LN02Chr03 :2788784-2789434(-) | 156	CDD:224675	40.449	89	47	2	56	139	6	93	2.36E-28	98.2	COG1761	RPB11	DNA-directed RNA polymerase, subunit L
LN02_04205 | LN02Chr03 :3350327-3352743(-) | 664	CDD:223358	29.658	526	273	19	132	655	1	431	6.05E-116	351	COG0281	SfcA	Malic enzyme
LN02_04269 | LN02Chr03 :3604616-3607679(-) | 898	CDD:227408	27.347	245	176	1	239	481	124	368	1.54E-47	171	COG5076	COG5076	Transcription factor involved in chromatin remodeling, contains bromodomain
LN02_04269 | LN02Chr03 :3604616-3607679(-) | 898	CDD:227408	28.421	95	68	0	64	158	159	253	7.00E-10	58.7	COG5076	COG5076	Transcription factor involved in chromatin remodeling, contains bromodomain
LN02_04461 | LN02Chr03 :4227909-4228750(+) | 252	CDD:223959	36.965	257	145	5	4	250	1	250	5.37E-62	192	COG1028	FabG	Dehydrogenases with different specificities (related to short-chain alcohol dehydrogenases)

LN02_04589 ǀ LN02Chr03 :4708652-4711372(+) ǀ 821	CDD:223464	34.951	206	127	3	171	375	12	211	1.90E-49	175	COG0387	ChaA	Ca2+/H+ antiporter
LN02_04589 ǀ LN02Chr03 :4708652-4711372(+) ǀ 821	CDD:223464	41.358	162	95	0	650	811	207	368	7.65E-49	174	COG0387	ChaA	Ca2+/H+ antiporter
LN02_04653 ǀ LN02Chr03 :4938075-4940727(-) ǀ 770	CDD:227934	34.197	772	477	11	16	770	16	773	0	595	COG5647	COG5647	Cullin, a subunit of E3 ubiquitin ligase
LN02_05229 ǀ LN02Chr04 :1035548-1036864(+) ǀ 438	CDD:223513	21.795	390	243	14	53	427	48	390	4.04E-30	117	COG0436	COG0436	Aspartate/tyrosine/aromatic aminotransferase
LN02_05293 ǀ LN02Chr04 :1227667-1229557(-) ǀ 556	CDD:223617	21.014	138	89	8	293	423	26	150	6.52E-07	47.8	COG0543	UbiB	2-polyprenylphenol hydroxylase and related flavodoxin oxidoreductases
LN02_05421 ǀ LN02Chr04 :1694091-1696741(-) ǀ 725	CDD:223796	16.216	148	116	3	325	465	105	251	9.94E-08	51.5	COG0724	COG0724	RNA-binding proteins (RRM domain)
LN02_05421 ǀ LN02Chr04 :1694091-1696741(-) ǀ 725	CDD:225606	33.028	109	65	4	447	553	90	192	1.06E-05	45.3	COG3064	TolA	Membrane protein involved in colicin uptake
LN02_05485 ǀ LN02Chr04 :2068963-2070012(+) ǀ 349	CDD:225546	28.75	160	94	6	170	326	91	233	1.72E-16	75.6	COG3000	ERG3	Sterol desaturase
LN02_05549 ǀ LN02Chr04 :2398657-2400133(-) ǀ 414	CDD:223540	35.088	342	211	4	5	343	136	469	2.50E-75	240	COG0464	SpoVK	ATPases of the AAA+ class
LN02_05741 ǀ LN02Chr04 :3049007-3050910(-) ǀ 529	CDD:223535	37.044	521	305	14	20	528	11	520	1.39E-150	438	COG0459	GroL	Chaperonin GroEL (HSP60 family)
LN02_05869 ǀ LN02Chr04 :3467376-3468674(-) ǀ 400	CDD:223101	48.936	329	160	5	75	400	1	324	4.60E-149	422	COG0022	AcoB	Pyruvate/2-oxoglutarate dehydrogenase complex, dehydrogenase (E1) component, eukaryotic type, beta subunit
LN02_05997 ǀ LN02Chr04 :3950659-3951990(+) ǀ 255	CDD:223176	38.693	199	104	6	48	246	1	181	9.30E-52	163	COG0098	RpsE	Ribosomal protein S5
LN02_06189 ǀ LN02Chr04 :4612541-4613298(+) ǀ 211	CDD:223307	43.796	137	74	3	72	207	4	138	1.96E-68	203	COG0229	COG0229	Conserved domain frequently associated with peptide methionine sulfoxide reductase
LN02_06317 ǀ LN02Chr04 :5013775-5015794(-) ǀ 560	CDD:223232	21.933	538	331	15	40	555	3	473	8.99E-64	213	COG0154	GatA	Asp-tRNAAsn/Glu-tRNAGln amidotransferase A subunit and related amidases
LN02_06445 ǀ LN02Chr04 :5412022-5412852(+) ǀ 242	CDD:227635	33.663	101	64	1	145	242	175	275	2.55E-12	62.2	COG5325	COG5325	t-SNARE complex subunit, syntaxin
LN02_06765 ǀ LN02Chr05 :732507-735272(+) ǀ 921	CDD:223587	23.024	582	278	16	342	921	22	435	3.10E-58	205	COG0513	SrmB	Superfamily II DNA and RNA helicases
LN02_06893 ǀ LN02Chr05 :1186333-1187548(+) ǀ 174	CDD:226682	37.306	193	86	7	12	173	7	195	9.96E-45	144	COG4229	COG4229	Predicted enolase-phosphatase
LN02_07469 ǀ LN02Chr05 :3877769-3879636(-) ǀ 517	CDD:223944	31.532	444	291	8	5	441	34	471	2.64E-108	328	COG1012	PutA	NAD-dependent aldehyde dehydrogenases
LN02_07597 ǀ LN02Chr05 :4420841-4425383(-) ǀ 1352	CDD:223671	23.383	201	107	9	1080	1263	150	320	1.13E-10	61.6	COG0598	CorA	Mg2+ and Co2+ transporters
LN02_07661 ǀ LN02Chr05 :4759151-4761746(-) ǀ 750	CDD:227602	28.808	302	185	12	54	345	8	289	3.02E-36	139	COG5277	COG5277	Actin and related proteins

LN02_07661 ǀ LN02Chr05 :4759151-4761746(-) ǀ 750	CDD:227602	30.719	153	79	3	618	743	290	442	1.25E-22	98.6	COG5277	COG5277	Actin and related proteins
LN02_07661 ǀ LN02Chr05 :4759151-4761746(-) ǀ 750	CDD:224300	23	100	69	3	296	393	6	99	2.70E-04	38.4	COG1382	GimC	Prefoldin, chaperonin cofactor
LN02_07853 ǀ LN02Chr05 :5502308-5503428(+) ǀ 347	CDD:227576	27.703	148	62	3	163	310	78	180	6.17E-17	75.5	COG5251	TAF40	Transcription initiation factor TFIID, subunit TAF11
LN02_08173 ǀ LN02Chr06 :1653857-1655140(+) ǀ 427	CDD:224732	25.747	435	283	13	1	427	2	404	1.04E-32	124	COG1819	COG1819	Glycosyl transferases, related to UDP-glucuronosyltransferase
LN02_08237 ǀ LN02Chr06 :1844162-1845987(+) ǀ 553	CDD:225201	20	240	178	6	136	361	69	308	5.30E-13	68.2	COG2319	COG2319	FOG: WD40 repeat
LN02_08365 ǀ LN02Chr06 :2255360-2256620(-) ǀ 352	CDD:225182	24.757	206	122	12	49	236	63	253	5.76E-08	50.9	COG2273	SKN1	Beta-glucanase/Beta-glucan synthetase
LN02_08429 ǀ LN02Chr06 :2585322-2585816(+) ǀ 145	CDD:223126	37.5	136	72	4	9	144	1	123	6.18E-51	155	COG0048	RpsL	Ribosomal protein S12
LN02_08621 ǀ LN02Chr06 :3182000-3183805(+) ǀ 601	CDD:227270	20.947	549	323	25	41	546	29	509	1.82E-16	80.2	COG4934	COG4934	Predicted protease
LN02_08685 ǀ LN02Chr06 :3524605-3527367(+) ǀ 920	CDD:226406	23.353	167	109	7	227	387	694	847	6.41E-05	43.7	COG3889	COG3889	Predicted solute binding protein
LN02_08877 ǀ LN02Chr07 :656750-657840(+) ǀ 296	CDD:223278	38.06	134	73	3	43	171	3	131	6.36E-27	100	COG0200	RplO	Ribosomal protein L15
LN02_08941 ǀ LN02Chr07 :901672-902933(-) ǀ 367	CDD:224172	28.021	389	181	12	7	363	82	403	4.72E-64	206	COG1252	Ndh	NADH dehydrogenase, FAD-containing subunit
LN02_09005 ǀ LN02Chr07 :1103965-1105121(+) ǀ 338	CDD:227374	36.111	288	102	4	3	280	18	233	1.38E-72	222	COG5041	SKB2	Casein kinase II, beta subunit
LN02_09133 ǀ LN02Chr07 :1536598-1537639(+) ǀ 320	CDD:223517	32.87	216	89	2	80	295	1	160	8.01E-53	168	COG0440	IlvH	Acetolactate synthase, small (regulatory) subunit
LN02_09325 ǀ LN02Chr07 :2213714-2215260(+) ǀ 494	CDD:223884	20.652	276	205	8	97	363	26	296	1.56E-08	53.4	COG0814	SdaC	Amino acid permeases
LN02_00046 ǀ LN02Chr01 :316841-317544(-) ǀ 214	CDD:226037	28.926	121	67	6	6	123	23	127	1.81E-11	57.9	COG3506	COG3506	Uncharacterized conserved protein
LN02_00174 ǀ LN02Chr01 :821442-823721(+) ǀ 542	CDD:226406	22.881	118	71	3	336	433	739	856	1.75E-04	41.4	COG3889	COG3889	Predicted solute binding protein
LN02_00366 ǀ LN02Chr01 :1911809-1913857(-) ǀ 551	CDD:223112	43.738	503	242	11	1	500	4	468	0	544	COG0034	PurF	Glutamine phosphoribosylpyrophosphate amidotransferase
LN02_00430 ǀ LN02Chr01 :2096793-2098991(+) ǀ 613	CDD:223385	22.605	668	422	21	24	613	26	676	3.00E-87	287	COG0308	PepN	Aminopeptidase N
LN02_00494 ǀ LN02Chr01 :2285677-2287614(+) ǀ 645	CDD:223221	38.772	570	321	15	14	570	4	558	0	630	COG0143	MetG	Methionyl-tRNA synthetase
LN02_00686 ǀ LN02Chr01 :3035742-3037996(-) ǀ 637	CDD:225371	27.273	154	111	1	135	287	36	189	3.88E-12	65.3	COG2814	AraJ	Arabinose efflux permease
LN02_00750 ǀ LN02Chr01 :3238054-3238638(+) ǀ 194	CDD:223735	21.97	132	92	3	22	153	13	133	4.56E-08	48	COG0663	PaaY	Carbonic anhydrases/acetyltransferases, isoleucine patch superfamily
LN02_01070 ǀ LN02Chr01 :4296684-4299787(-) ǀ 895	CDD:227881	30.796	867	498	14	45	882	34	827	2.71E-176	528	COG5594	COG5594	Uncharacterized integral membrane protein

LN02_01198 ǀ LN02Chr01 :4848186-4849142(-) ǀ 169	CDD:2239 86	32.558	129	59	6	15	133	3	113	3.68E-32	112	COG10 58	CinA	Predicted nucleotide-utilizing enzyme related to molybdopterin-biosynthesis enzyme MoeA
LN02_01262 ǀ LN02Chr01 :5090711-5092824(+) ǀ 620	CDD:2246 10	23.098	368	250	8	266	620	78	425	2.19E-48	172	COG16 96	DltB	Predicted membrane protein involved in D-alanine export
LN02_01390 ǀ LN02Chr01 :5555607-5556459(+) ǀ 257	CDD:2233 55	54.545	88	39	1	149	235	3	90	1.55E-49	155	COG02 78	COG027 8	Glutaredoxin-related protein
LN02_01390 ǀ LN02Chr01 :5555607-5556459(+) ǀ 257	CDD:2256 60	23.077	143	93	6	2	139	23	153	4.03E-07	47	COG31 18	COG311 8	Thioredoxin domain-containing protein
LN02_01518 ǀ LN02Chr01 :6057349-6057796(+) ǀ 104	CDD:2233 49	34.483	87	49	2	4	82	3	89	4.91E-22	79.7	COG02 71	BolA	Stress-induced morphogen (activity unknown)
LN02_01646 ǀ LN02Chr01 :6571592-6575492(-) ǀ 455	CDD:2232 43	42.667	75	43	0	1	75	10	84	2.38E-15	75	COG01 65	ArgH	Argininosuccinate lyase
LN02_01966 ǀ LN02Chr02 :133014-134051(-) ǀ 280	CDD:2239 59	33.077	260	160	8	2	256	1	251	2.80E-58	184	COG10 28	FabG	Dehydrogenases with different specificities (related to short-chain alcohol dehydrogenases)
LN02_02158 ǀ LN02Chr02 :768234-771022(-) ǀ 811	CDD:2241 62	36.921	734	382	15	77	798	18	682	0	638	COG12 41	MCM2	Predicted ATPase involved in replication control, Cdc46/Mcm family
LN02_02222 ǀ LN02Chr02 :975653-976984(+) ǀ 406	CDD:2232 00	22.176	239	150	5	87	325	63	265	2.06E-33	123	COG01 22	AlkA	3-methyladenine DNA glycosylase/8-oxoguanine DNA glycosylase
LN02_02734 ǀ LN02Chr02 :2805220-2806377(-) ǀ 385	CDD:2237 96	18.812	202	154	3	161	362	42	233	3.51E-16	75.8	COG07 24	COG072 4	RNA-binding proteins (RRM domain)
LN02_02798 ǀ LN02Chr02 :3027729-3028850(-) ǀ 262	CDD:2236 65	22.097	267	189	8	6	258	62	323	3.46E-26	102	COG05 92	DnaN	DNA polymerase sliding clamp subunit (PCNA homolog)
LN02_02862 ǀ LN02Chr02 :3248801-3249984(-) ǀ 261	CDD:2273 74	48.908	229	114	2	2	227	9	237	1.67E-97	282	COG50 41	SKB2	Casein kinase II, beta subunit
LN02_02926 ǀ LN02Chr02 :3486689-3491156(-) ǀ 1446	CDD:2241 17	24.828	145	101	3	1090	1234	319	455	3.48E-04	42	COG11 96	Smc	Chromosome segregation ATPases
LN02_03054 ǀ LN02Chr02 :3925806-3930323(-) ǀ 1450	CDD:2236 27	31.461	712	423	20	379	1041	158	853	7.69E-128	416	COG05 53	HepA	Superfamily II DNA/RNA helicases, SNF2 family
LN02_03054 ǀ LN02Chr02 :3925806-3930323(-) ǀ 1450	CDD:2274 08	23.846	260	170	5	1160	1408	25	267	1.38E-22	98.7	COG50 76	COG507 6	Transcription factor involved in chromatin remodeling, contains bromodomain
LN02_03118 ǀ LN02Chr02 :4149083-4149682(+) ǀ 199	CDD:3192 44	31.429	70	46	2	125	193	1	69	8.16E-06	39.7	COG52 72	UBI4	UBI4; linked to 3D-structure.
LN02_03182 ǀ LN02Chr02 :4417110-4418147(-) ǀ 251	CDD:2231 14	42.213	244	109	4	6	246	5	219	1.15E-93	271	COG00 36	Rpe	Pentose-5-phosphate-3-epimerase
LN02_03310 ǀ LN02Chr02 :4823850-4825975(-) ǀ 610	CDD:2231 39	32.967	273	135	2	326	598	56	280	2.50E-67	218	COG00 61	nadF	NAD kinase
LN02_03566 ǀ LN02Chr02 :6230782-6234710(+) ǀ 1223	CDD:2233 27	35.329	852	497	18	334	1179	1	804	0	715	COG02 49	MutS	Mismatch repair ATPase (MutS family)
LN02_03630 ǀ LN02Chr03 :53184-54500(+) ǀ 438	CDD:2237 49	31.646	395	230	9	51	414	10	395	6.72E-83	258	COG06 77	WecC	UDP-N-acetyl-D-mannosaminuronate dehydrogenase

LN02_03886|LN02Chr03:1017423-1018196(-)|92	CDD:224908	51.765	85	41	0	4	88	3	87	1.80E-30	100	COG1997	RPL43A	Ribosomal protein L37AE/L43A
LN02_03950|LN02Chr03:1218774-1219926(+)|317	CDD:225979	32.487	197	116	8	22	213	1	185	3.30E-21	89.9	COG3448	COG3448	CBS-domain-containing membrane protein
LN02_04462|LN02Chr03:4229856-4231629(+)|499	CDD:223944	42.616	474	259	6	19	490	2	464	3.85E-168	480	COG1012	PutA	NAD-dependent aldehyde dehydrogenases
LN02_04910|LN02Chr03:5931315-5934641(+)|952	CDD:227928	35.878	131	57	5	663	779	158	275	2.38E-11	64.1	COG5641	GAT1	GATA Zn-finger-containing transcription factor
LN02_05102|LN02Chr04:613210-615789(+)|684	CDD:223100	52.096	668	306	7	4	664	3	663	0	1048	COG0021	TktA	Transketolase
LN02_05166|LN02Chr04:836197-841119(+)|1477	CDD:224557	31.085	756	421	19	633	1372	8	679	8.85E-171	531	COG1643	HrpA	HrpA-like helicases
LN02_05422|LN02Chr04:1697706-1700377(-)|823	CDD:223556	29.34	818	432	27	2	808	14	696	0	632	COG0480	FusA	Translation elongation factors (GTPases)
LN02_05678|LN02Chr04:2835347-2836476(+)|352	CDD:227640	26.042	192	125	5	34	218	23	204	2.15E-24	98.3	COG5333	CCL1	Cdk activating kinase (CAK)/RNA polymerase II transcription initiation/nucleotide excision repair factor TFIIH/TFIIK, cyclin H subunit
LN02_05742|LN02Chr04:3051445-3053928(+)|746	CDD:227657	32.323	594	344	15	169	743	3	557	9.01E-144	430	COG5354	COG5354	Uncharacterized protein, contains Trp-Asp (WD) repeat
LN02_05806|LN02Chr04:3251137-3253183(-)|632	CDD:226017	32.77	473	245	18	167	631	6	413	8.21E-113	342	COG3486	IucD	Lysine/ornithine N-monooxygenase
LN02_05870|LN02Chr04:3469459-3471608(+)|696	CDD:225344	28.297	417	245	18	276	677	12	389	2.60E-52	183	COG2730	BglC	Endoglucanase
LN02_05998|LN02Chr04:3953723-3955013(-)|402	CDD:227357	28.571	252	168	4	49	290	178	427	2.30E-43	154	COG5024	COG5024	Cyclin
LN02_06190|LN02Chr04:4614394-4616113(+)|419	CDD:223261	35.556	405	230	13	31	419	1	390	1.52E-90	276	COG0183	PaaJ	Acetyl-CoA acetyltransferase
LN02_06318|LN02Chr04:5016897-5018544(+)|453	CDD:223959	20	185	120	4	110	292	32	190	8.74E-05	41	COG1028	FabG	Dehydrogenases with different specificities (related to short-chain alcohol dehydrogenases)
LN02_06446|LN02Chr04:5414111-5417165(+)|688	CDD:223774	30.667	75	47	2	521	595	6	75	8.24E-04	38.7	COG0702	COG0702	Predicted nucleoside-diphosphate-sugar epimerases
LN02_06574|LN02Chr04:5901186-5902587(-)|350	CDD:223594	27.019	359	209	16	6	333	2	338	1.14E-49	168	COG0520	csdA	Selenocysteine lyase/Cysteine desulfurase
LN02_06638|LN02Chr05:196055-197461(+)|401	CDD:227598	24.528	106	68	4	112	215	79	174	7.30E-20	86.3	COG5273	COG5273	Uncharacterized protein containing DHHC-type Zn finger
LN02_07022|LN02Chr05:1982324-1983348(-)|149	CDD:227455	58.042	143	59	1	5	147	14	155	8.09E-56	169	COG5126	FRQ1	Ca2+-binding protein (EF-Hand superfamily)
LN02_07214|LN02Chr05:3003415-3007801(-)|1387	CDD:223540	32.164	513	275	15	726	1217	3	463	6.14E-88	292	COG0464	SpoVK	ATPases of the AAA+ class
LN02_07342|LN02Chr05:3396862-3397392(+)|176	CDD:227232	61.818	55	20	1	49	102	8	62	1.51E-16	67.6	COG4895	COG4895	Uncharacterized conserved protein
LN02_07534|LN02Chr05:4092001-4092804(-)|183	CDD:224025	28.788	132	90	1	20	147	5	136	1.09E-26	98.1	COG1100	COG1100	GTPase SAR1 and related small G proteins

LN02_07918｜LN02Chr05:5683464-5684692(+)｜330	CDD:225130	18.893	307	179	11	27	317	1	253	2.89E-06	44.8	COG2220	COG2220	Predicted Zn-dependent hydrolases of the beta-lactamase fold
LN02_08174｜LN02Chr06:1655971-1658167(-)｜702	CDD:227590	56.225	498	216	1	177	674	1	496	0	709	COG5265	ATM1	ABC-type transport system involved in Fe-S cluster assembly, permease and ATPase components
LN02_08238｜LN02Chr06:1846362-1847150(-)｜227	CDD:224901	52.066	121	57	1	107	227	3	122	5.30E-50	156	COG1990	pth2	Peptidyl-tRNA hydrolase
LN02_08302｜LN02Chr06:2054274-2056867(-)｜840	CDD:226406	26.733	101	65	3	260	352	739	838	4.06E-04	41	COG3889	COG3889	Predicted solute binding protein
LN02_08430｜LN02Chr06:2586086-2587309(-)｜407	CDD:223459	29.139	302	200	3	92	393	2	289	2.12E-61	197	COG0382	UbiA	4-hydroxybenzoate polyprenyltransferase and related prenyltransferases
LN02_08814｜LN02Chr07:435319-435785(-)｜146	CDD:226314	25.926	135	81	7	9	134	7	131	1.29E-13	60.9	COG3791	COG3791	Uncharacterized conserved protein
LN02_08878｜LN02Chr07:662695-665125(-)｜659	CDD:223207	42.351	621	268	12	53	657	25	571	0	594	COG0129	IlvD	Dihydroxyacid dehydratase/phosphogluconate dehydratase
LN02_08942｜LN02Chr07:906976-909531(-)｜514	CDD:223428	42.294	279	138	3	7	285	5	260	7.97E-96	288	COG0351	ThiD	Hydroxymethylpyrimidine/phosphomethylpyrimidine kinase
LN02_08942｜LN02Chr07:906976-909531(-)｜514	CDD:223889	36.279	215	132	3	297	510	3	213	1.24E-64	206	COG0819	TenA	Putative transcription activator
LN02_09070｜LN02Chr07:1322766-1326103(-)｜989	CDD:227427	25.328	229	142	8	99	325	72	273	2.87E-09	57.8	COG5096	COG5096	Vesicle coat complex, various subunits
LN02_09134｜LN02Chr07:1537856-1539034(-)｜352	CDD:223156	44.753	324	159	6	22	343	1	306	1.16E-126	363	COG0078	ArgF	Ornithine carbamoyltransferase
LN02_09198｜LN02Chr07:1720369-1722341(+)｜633	CDD:223704	32.022	178	83	8	444	606	105	259	2.51E-19	85.1	COG0631	PTC1	Serine/threonine protein phosphatase
LN02_09326｜LN02Chr07:2216491-2218056(+)｜495	CDD:224786	26.531	98	54	4	64	160	23	103	1.66E-04	41.3	COG1874	LacA	Beta-galactosidase
LN02_00047｜LN02Chr01:318697-320985(+)｜762	CDD:224323	25.088	283	181	9	67	328	2	274	3.90E-45	160	COG1405	SUA7	Transcription initiation factor TFIIIB, Brf1 subunit/Transcription initiation factor TFIIB
LN02_00111｜LN02Chr01:508347-510862(-)｜701	CDD:223107	45.061	577	288	7	100	676	2	549	0	666	COG0028	IlvB	Thiamine pyrophosphate-requiring enzymes
LN02_00239｜LN02Chr01:1432825-1434434(-)｜462	CDD:224143	47.281	423	202	1	40	462	3	404	0	549	COG1222	RPT1	ATP-dependent 26S proteasome regulatory subunit
LN02_00303｜LN02Chr01:1605698-1607415(+)｜538	CDD:223605	20.996	462	332	10	42	490	8	449	3.93E-26	108	COG0531	PotE	Amino acid transporters
LN02_00431｜LN02Chr01:2099225-2099655(-)｜117	CDD:226033	31.373	102	63	3	10	104	6	107	4.59E-13	58.2	COG3502	COG3502	Uncharacterized protein conserved in bacteria
LN02_00495｜LN02Chr01:2288349-2289136(+)｜226	CDD:223532	27.848	158	106	3	1	154	12	165	6.11E-28	101	COG0456	RimI	Acetyltransferases
LN02_00751｜LN02Chr01:3238733-3240475(-)｜497	CDD:224371	36.554	383	200	7	79	461	10	349	7.64E-102	307	COG1454	EutG	Alcohol dehydrogenase, class IV

LN02_00879	LN02Chr01 :3671101-3671916(+)	162	CDD:223725	47.436	156	68	6	15	162	8	157	1.92E-62	186	COG0652	PpiB	Peptidyl-prolyl cis-trans isomerase (rotamase) - cyclophilin family
LN02_01007	LN02Chr01 :4076457-4078240(-)	491	CDD:227352	48.429	382	182	3	96	476	1	368	4.10E-157	448	COG5019	CDC3	Septin family protein
LN02_01071	LN02Chr01 :4309167-4311869(-)	835	CDD:226406	23.958	96	71	2	63	157	758	852	5.66E-04	40.6	COG3889	COG3889	Predicted solute binding protein
LN02_01135	LN02Chr01 :4513642-4514288(-)	146	CDD:224301	59.459	74	30	0	1	74	1	74	6.19E-33	108	COG1383	RPS17A	Ribosomal protein S17E
LN02_01519	LN02Chr01 :6058291-6060926(-)	750	CDD:227427	30.621	676	418	15	17	655	19	680	8.47E-151	455	COG5096	COG5096	Vesicle coat complex, various subunits
LN02_01711	LN02Chr01 :6823400-6825921(+)	766	CDD:223540	32.381	525	293	11	249	761	20	494	5.88E-92	294	COG0464	SpoVK	ATPases of the AAA+ class
LN02_01775	LN02Chr01 :6986131-6988117(-)	590	CDD:223395	31.516	587	312	13	11	582	16	527	3.49E-103	319	COG0318	CaiC	Acyl-CoA synthetases (AMP-forming)/AMP-acid ligases II
LN02_01967	LN02Chr02 :135454-137288(+)	481	CDD:223252	31.557	469	256	16	29	480	23	443	2.23E-110	331	COG0174	GlnA	Glutamine synthetase
LN02_02159	LN02Chr02 :771698-775455(+)	1222	CDD:223540	32.022	534	281	14	530	1062	12	464	2.12E-90	298	COG0464	SpoVK	ATPases of the AAA+ class
LN02_02223	LN02Chr02 :977354-981001(+)	1215	CDD:227508	49.128	975	459	7	240	1212	29	968	0	1230	COG5181	HSH155	U2 snRNP spliceosome subunit
LN02_02287	LN02Chr02 :1230541-1231561(-)	308	CDD:224025	23.853	218	147	5	1	209	3	210	1.83E-22	90.4	COG1100	COG1100	GTPase SAR1 and related small G proteins
LN02_02479	LN02Chr02 :1959524-1961979(-)	784	CDD:223283	41.019	373	183	6	4	374	2	339	1.08E-128	385	COG0205	PfkA	6-phosphofructokinase
LN02_02479	LN02Chr02 :1959524-1961979(-)	784	CDD:223283	29.08	337	202	10	397	727	3	308	3.29E-40	148	COG0205	PfkA	6-phosphofructokinase
LN02_02607	LN02Chr02 :2403699-2405933(-)	744	CDD:223916	24.138	174	91	11	357	505	17	174	1.31E-11	62.6	COG0847	DnaQ	DNA polymerase III, epsilon subunit and related 3'-5' exonucleases
LN02_02735	LN02Chr02 :2808111-2810392(+)	531	CDD:223439	60.95	484	177	4	41	523	1	473	0	804	COG0362	Gnd	6-phosphogluconate dehydrogenase
LN02_02863	LN02Chr02 :3250665-3254200(-)	1100	CDD:225201	17.907	430	298	15	143	562	47	431	1.33E-04	42.8	COG2319	COG2319	FOG: WD40 repeat
LN02_02927	LN02Chr02 :3491339-3492418(+)	192	CDD:223264	39.08	87	52	1	97	183	1	86	2.65E-31	106	COG0186	RpsQ	Ribosomal protein S17
LN02_03055	LN02Chr02 :3931736-3933487(-)	583	CDD:225201	17.906	363	250	12	90	428	53	391	3.21E-14	72	COG2319	COG2319	FOG: WD40 repeat
LN02_03375	LN02Chr02 :5028159-5030059(+)	475	CDD:223712	32.026	153	96	4	284	429	3	154	6.18E-23	92	COG0639	ApaH	Diadenosine tetraphosphatase and related serine/threonine protein phosphatases
LN02_03375	LN02Chr02 :5028159-5030059(+)	475	CDD:223533	28.571	105	74	1	2	105	164	268	4.62E-08	51.4	COG0457	NrfG	FOG: TPR repeat
LN02_03439	LN02Chr02 :5302583-5304511(+)	642	CDD:225805	25.455	110	73	3	362	466	33	138	4.18E-05	43	COG3266	DamX	Uncharacterized protein conserved in bacteria
LN02_03567	LN02Chr02 :6235001-6236471(-)	323	CDD:223254	29.719	249	136	10	5	250	1	213	4.83E-36	127	COG0176	MipB	Transaldolase

LN02_03631 ǀ LN02Chr03 :55042-57526(-) ǀ 732	CDD:227598	30.841	214	136	4	376	585	33	238	4.65E-35	132	COG5273	COG5273	Uncharacterized protein containing DHHC-type Zn finger
LN02_03631 ǀ LN02Chr03 :55042-57526(-) ǀ 732	CDD:223738	31.724	145	86	4	91	225	68	209	2.06E-17	79.5	COG0666	Arp	FOG: Ankyrin repeat
LN02_03631 ǀ LN02Chr03 :55042-57526(-) ǀ 732	CDD:223738	25.466	161	112	2	160	312	70	230	2.70E-13	67.2	COG0666	Arp	FOG: Ankyrin repeat
LN02_03695 ǀ LN02Chr03 :335916-337196(+) ǀ 342	CDD:225311	36.538	260	158	5	45	304	4	256	3.14E-58	187	COG2513	PrpB	PEP phosphonomutase and related enzymes
LN02_03823 ǀ LN02Chr03 :848652-854580(-) ǀ 1855	CDD:223395	20.55	691	351	21	291	970	7	510	1.92E-32	131	COG0318	CaiC	Acyl-CoA synthetases (AMP-forming)/AMP-acid ligases II
LN02_03823 ǀ LN02Chr03 :848652-854580(-) ǀ 1855	CDD:223395	17.215	517	319	16	1063	1559	13	440	3.80E-20	93.3	COG0318	CaiC	Acyl-CoA synthetases (AMP-forming)/AMP-acid ligases II
LN02_03887 ǀ LN02Chr03 :1019058-1020708(+) ǀ 423	CDD:225807	26.179	424	245	13	6	413	3	374	4.94E-51	173	COG3268	COG3268	Uncharacterized conserved protein
LN02_04207 ǀ LN02Chr03 :3365442-3366851(+) ǀ 469	CDD:224983	20.833	432	268	18	11	426	8	381	4.53E-33	127	COG2072	TrkA	Predicted flavoprotein involved in K+ transport
LN02_04271 ǀ LN02Chr03 :3608882-3611175(-) ǀ 689	CDD:223085	24.538	379	254	11	246	615	12	367	1.49E-64	216	COG0006	PepP	Xaa-Pro aminopeptidase
LN02_04335 ǀ LN02Chr03 :3840276-3842801(-) ǀ 841	CDD:223416	29.491	668	396	19	156	810	59	664	4.72E-139	425	COG0339	Dcp	Zn-dependent oligopeptidases
LN02_04463 ǀ LN02Chr03 :4241832-4243434(+) ǀ 451	CDD:225371	26.126	111	81	1	88	197	51	161	1.06E-09	56.9	COG2814	AraJ	Arabinose efflux permease
LN02_04591 ǀ LN02Chr03 :4716195-4720175(-) ǀ 1307	CDD:223496	21.652	799	549	22	3	777	2	747	1.96E-31	130	COG0419	SbcC	ATPase involved in DNA repair
LN02_04591 ǀ LN02Chr03 :4716195-4720175(-) ǀ 1307	CDD:224117	16.724	293	218	4	845	1126	169	446	2.00E-07	52.4	COG1196	Smc	Chromosome segregation ATPases
LN02_04591 ǀ LN02Chr03 :4716195-4720175(-) ǀ 1307	CDD:227321	28.09	89	51	4	1191	1278	447	523	3.77E-07	51.5	COG4988	CydD	ABC-type transport system involved in cytochrome bd biosynthesis, ATPase and permease components
LN02_04591 ǀ LN02Chr03 :4716195-4720175(-) ǀ 1307	CDD:227278	15.417	240	174	5	720	948	50	271	3.90E-05	44.7	COG4942	COG4942	Membrane-bound metallopeptidase
LN02_04719 ǀ LN02Chr03 :5169860-5170767(-) ǀ 148	CDD:225793	35.135	111	62	3	36	146	3	103	3.88E-31	105	COG3254	COG3254	Uncharacterized conserved protein
LN02_04911 ǀ LN02Chr03 :5936318-5939006(-) ǀ 696	CDD:223589	22.559	297	172	10	367	608	1	294	2.65E-21	93.7	COG0515	SPS1	Serine/threonine protein kinase
LN02_05039 ǀ LN02Chr04 :417024-419803(+) ǀ 630	CDD:223097	32.524	618	354	18	34	630	2	577	1.43E-159	467	COG0018	ArgS	Arginyl-tRNA synthetase
LN02_05167 ǀ LN02Chr04 :841626-844859(-) ǀ 1077	CDD:223796	25	172	122	3	693	857	25	196	2.05E-18	84.6	COG0724	COG0724	RNA-binding proteins (RRM domain)
LN02_05167 ǀ LN02Chr04 :841626-844859(-) ǀ 1077	CDD:223796	18.595	242	158	5	565	771	69	306	3.19E-13	68.8	COG0724	COG0724	RNA-binding proteins (RRM domain)
LN02_05167 ǀ LN02Chr04 :841626-844859(-) ǀ 1077	CDD:227438	20.307	261	154	12	44	285	108	333	6.87E-04	40.4	COG5107	RNA14	Pre-mRNA 3'-end processing (cleavage and polyadenylation) factor
LN02_05359 ǀ LN02Chr04 :1481864-1483286(+) ǀ 318	CDD:223118	38.535	314	162	5	10	318	2	289	4.25E-101	296	COG0040	HisG	ATP phosphoribosyltransferase

LN02_05615 | LN02Chr04 :2638085-2640254(+) | 685	CDD:2231 15	21.39	374	183	10	43	409	24	293	2.51E-27	109	COG00 37	MesJ	tRNA(Ile)-lysidine synthase MesJ
LN02_05743 | LN02Chr04 :3057255-3061187(+) | 1265	CDD:2235 89	25.325	308	177	10	837	1127	7	278	5.03E-35	135	COG05 15	SPS1	Serine/threonine protein kinase
LN02_05807 | LN02Chr04 :3257406-3259710(+) | 599	CDD:2239 51	31.858	452	275	9	3	444	214	642	1.78E-78	258	COG10 20	EntF	Non-ribosomal peptide synthetase modules and related proteins
LN02_06127 | LN02Chr04 :4360059-4362149(+) | 451	CDD:2246 62	23.058	399	291	8	3	397	2	388	2.29E-84	261	COG17 48	LYS9	Saccharopine dehydrogenase and related proteins
LN02_06191 | LN02Chr04 :4616622-4618247(-) | 466	CDD:2252 80	20.507	473	199	19	18	464	7	328	5.88E-35	130	COG24 23	COG242 3	Predicted ornithine cyclodeaminase, mu-crystallin homolog
LN02_06319 | LN02Chr04 :5018976-5019821(-) | 281	CDD:2274 16	31.771	192	103	8	19	191	53	235	3.44E-34	122	COG50 84	YTH1	Cleavage and polyadenylation specificity factor (CPSF) Clipper subunit and related makorin family Zn-finger proteins
LN02_06575 | LN02Chr04 :5902881-5904560(+) | 456	CDD:2249 83	26.718	262	127	8	3	257	5	208	5.21E-28	112	COG20 72	TrkA	Predicted flavoprotein involved in K+ transport
LN02_06703 | LN02Chr05 :472079-473681(-) | 438	CDD:2236 69	24.427	131	88	3	71	201	37	156	9.45E-05	41.2	COG05 96	MhpC	Predicted hydrolases or acyltransferases (alpha/beta hydrolase superfamily)
LN02_06767 | LN02Chr05 :738406-739983(+) | 525	CDD:2233 54	25.49	204	140	3	6	200	39	239	1.36E-13	69.9	COG02 77	GlcD	FAD/FMN-containing dehydrogenases
LN02_07215 | LN02Chr05 :3008448-3011279(+) | 718	CDD:2236 69	17.593	216	155	7	328	541	23	217	7.61E-05	42.3	COG05 96	MhpC	Predicted hydrolases or acyltransferases (alpha/beta hydrolase superfamily)
LN02_07279 | LN02Chr05 :3178138-3178867(-) | 214	CDD:2231 59	29.947	187	120	8	13	194	45	225	4.46E-15	68.4	COG00 81	RplA	Ribosomal protein L1
LN02_07599 | LN02Chr05 :4430238-4431135(-) | 272	CDD:2232 89	35.87	92	49	3	46	134	2	86	6.34E-24	89.6	COG02 11	RpmA	Ribosomal protein L27
LN02_07663 | LN02Chr05 :4763243-4763833(+) | 196	CDD:2275 22	47.059	68	36	0	129	196	51	118	2.53E-21	82	COG51 95	COG519 5	Uncharacterized conserved protein
LN02_07855 | LN02Chr05 :5506072-5507331(-) | 355	CDD:2238 69	44.126	349	184	2	7	355	3	340	3.67E-107	315	COG07 98	ACR3	Arsenite efflux pump ACR3 and related permeases
LN02_08239 | LN02Chr06 :1847521-1848439(+) | 254	CDD:2236 45	21.569	204	121	9	7	206	6	174	3.01E-23	91.2	COG05 72	Udk	Uridine kinase
LN02_08367 | LN02Chr06 :2259752-2261668(-) | 638	CDD:2237 38	27.273	143	83	4	245	381	58	185	5.64E-05	42.1	COG06 66	Arp	FOG: Ankyrin repeat
LN02_08751 | LN02Chr07 :242465-244915(-) | 791	CDD:2260 67	29.235	797	456	30	30	789	37	762	3.47E-128	398	COG35 37	COG353 7	Putative alpha-1,2-mannosidase
LN02_09071 | LN02Chr07 :1326816-1327992(+) | 352	CDD:2251 36	24.348	115	64	4	87	197	54	149	7.64E-09	52.7	COG22 26	UbiE	Methylase involved in ubiquinone/menaquinone biosynthesis
LN02_09327 | LN02Chr07 :2220623-2221783(+) | 386	CDD:2235 89	26.168	107	74	2	197	302	75	177	4.46E-07	48.6	COG05 15	SPS1	Serine/threonine protein kinase
LN02_00240 | LN02Chr01 :1434848-1435901(+) | 332	CDD:2233 22	17.671	249	118	6	74	322	7	168	1.50E-05	41.8	COG02 44	RplJ	Ribosomal protein L10
LN02_00944 | LN02Chr01 :3853863-3855242(-) | 459	CDD:2277 21	22.066	213	124	9	160	352	203	393	2.03E-12	66	COG54 34	PGU1	Endopygalactorunase

LN02_01008|LN02Chr01:4083117-4084461(-)|421	CDD:223353	36.95	341	187	8	51	386	2	319	8.56E-117	341	COG0276	HemH	Protoheme ferro-lyase (ferrochelatase)
LN02_01200|LN02Chr01:4851103-4851717(-)|204	CDD:223563	31.977	172	107	4	34	199	10	177	2.67E-41	137	COG0489	Mrp	ATPases involved in chromosome partitioning
LN02_01520|LN02Chr01:6061922-6064645(-)|872	CDD:224706	26.108	609	281	19	237	843	2	443	6.39E-76	251	COG1793	CDC9	ATP-dependent DNA ligase
LN02_01648|LN02Chr01:6578287-6579111(-)|274	CDD:225136	37.313	134	78	2	23	156	37	164	7.83E-31	112	COG2226	UbiE	Methylase involved in ubiquinone/menaquinone biosynthesis
LN02_01776|LN02Chr01:6991327-6991842(+)|117	CDD:223349	44.681	94	45	2	6	99	4	90	4.20E-25	88.2	COG0271	BolA	Stress-induced morphogen (activity unknown)
LN02_01840|LN02Chr01:7187472-7188101(+)|209	CDD:224565	18.935	169	102	6	26	190	90	227	8.07E-09	51	COG1651	DsbG	Protein-disulfide isomerase
LN02_01968|LN02Chr02:137597-138740(-)|333	CDD:223298	28.205	117	54	7	93	185	52	162	6.15E-04	37.7	COG0220	COG0220	Predicted S-adenosylmethionine-dependent methyltransferase
LN02_02160|LN02Chr02:775621-776871(-)|416	CDD:224139	24.127	315	159	11	74	380	4	246	4.49E-25	100	COG1218	CysQ	3'-Phosphoadenosine 5'-phosphosulfate (PAPS) 3'-phosphatase
LN02_02224|LN02Chr02:990138-991493(+)|357	CDD:227381	26.667	105	73	2	244	344	245	349	2.44E-05	42.8	COG5048	COG5048	FOG: Zn-finger
LN02_02288|LN02Chr02:1233212-1234595(+)|394	CDD:223270	61.008	377	143	2	15	390	4	377	0	598	COG0192	MetK	S-adenosylmethionine synthetase
LN02_02352|LN02Chr02:1503364-1505624(-)|524	CDD:226734	43.8	500	241	14	28	518	4	472	4.23E-167	478	COG4284	COG4284	UDP-glucose pyrophosphorylase
LN02_02416|LN02Chr02:1749578-1751830(+)|546	CDD:223520	21.483	391	240	20	4	366	5	356	2.75E-21	94.3	COG0443	DnaK	Molecular chaperone
LN02_02544|LN02Chr02:2171512-2175252(-)|1025	CDD:224786	27.976	168	106	7	45	202	1	163	3.11E-17	83.6	COG1874	LacA	Beta-galactosidase
LN02_02672|LN02Chr02:2619761-2622450(+)|768	CDD:227820	23.611	288	194	9	210	487	141	412	1.31E-35	137	COG5533	UBP5	Ubiquitin C-terminal hydrolase
LN02_02864|LN02Chr02:3256155-3258048(+)|551	CDD:224089	31.011	445	234	11	105	549	87	458	1.27E-88	278	COG1167	ARO8	Transcriptional regulators containing a DNA-binding HTH domain and an aminotransferase domain (MocR family) and their eukaryotic orthologs
LN02_03056|LN02Chr02:3934436-3936225(-)|489	CDD:225035	24.331	411	249	13	59	458	25	384	1.23E-46	164	COG2124	CypX	Cytochrome P450
LN02_03184|LN02Chr02:4421520-4422959(+)|419	CDD:223699	31.235	429	234	12	14	419	6	396	2.48E-93	283	COG0626	MetC	Cystathionine beta-lyases/cystathionine gamma-synthases
LN02_03312|LN02Chr02:4828295-4830049(+)|257	CDD:223598	20.5	200	136	9	8	201	6	188	4.30E-04	37.9	COG0524	RbsK	Sugar kinases, ribokinase family
LN02_03568|LN02Chr02:6236871-6238457(+)|528	CDD:223187	32.927	328	189	5	160	485	3	301	1.02E-59	197	COG0109	CyoE	Polyprenyltransferase (cytochrome oxidase assembly factor)
LN02_03632|LN02Chr03:58232-59089(+)|285	CDD:225138	24.855	173	86	7	48	210	12	150	3.27E-11	58.2	COG2229	COG2229	Predicted GTPase
LN02_03696|LN02Chr03:341729-343174(+)|481	CDD:223594	24.943	437	264	14	34	464	24	402	2.84E-59	197	COG0520	csdA	Selenocysteine lyase/Cysteine desulfurase

LN02_03952∣LN02Chr03:1226288-1229147(+)∣900	CDD:225514	30.952	252	167	5	396	645	4	250	1.67E-59	200	COG2966	COG2966	Uncharacterized conserved protein
LN02_03952∣LN02Chr03:1226288-1229147(+)∣900	CDD:226137	17.13	216	109	4	674	889	8	153	3.28E-15	71.2	COG3610	COG3610	Uncharacterized conserved protein
LN02_04016∣LN02Chr03:1453749-1455707(-)∣570	CDD:223553	22.378	286	214	6	82	360	1	285	2.37E-08	53.2	COG0477	ProP	Permeases of the major facilitator superfamily
LN02_04208∣LN02Chr03:3367855-3369271(-)∣352	CDD:223166	21.569	255	159	6	4	258	1	214	2.00E-32	117	COG0088	RplD	Ribosomal protein L4
LN02_04272∣LN02Chr03:3611560-3612283(+)∣165	CDD:223158	35.211	142	88	3	7	146	2	141	6.24E-40	129	COG0080	RplK	Ribosomal protein L11
LN02_04464∣LN02Chr03:4245858-4247750(+)∣474	CDD:227581	58.15	454	163	4	3	456	2	428	0	672	COG5256	TEF1	Translation elongation factor EF-1alpha (GTPase)
LN02_04528∣LN02Chr03:4444654-4446928(+)∣623	CDD:223780	24.107	336	164	12	4	323	1	261	3.40E-46	161	COG0708	XthA	Exonuclease III
LN02_04592∣LN02Chr03:4723052-4724752(+)∣507	CDD:225112	33.333	93	60	1	153	243	109	201	1.24E-05	43.7	COG2202	AtoS	FOG: PAS/PAC domain
LN02_04656∣LN02Chr03:4947736-4948404(-)∣163	CDD:223807	31.081	148	71	4	16	160	5	124	1.94E-25	91.5	COG0736	AcpS	Phosphopantetheinyl transferase (holo-ACP synthase)
LN02_04784∣LN02Chr03:5401782-5404658(+)∣764	CDD:224805	23.127	307	206	12	32	331	24	307	1.05E-28	114	COG1893	ApbA	Ketopantoate reductase
LN02_04976∣LN02Chr04:230008-231117(+)∣332	CDD:223589	22.624	221	138	7	61	251	1	218	3.21E-15	72.9	COG0515	SPS1	Serine/threonine protein kinase
LN02_05168∣LN02Chr04:847449-850157(+)∣902	CDD:223738	27.922	154	101	3	347	491	51	203	8.30E-08	51.4	COG0666	Arp	FOG: Ankyrin repeat
LN02_05168∣LN02Chr04:847449-850157(+)∣902	CDD:223738	29.078	141	77	4	482	619	54	174	1.52E-07	50.6	COG0666	Arp	FOG: Ankyrin repeat
LN02_05168∣LN02Chr04:847449-850157(+)∣902	CDD:223738	24.286	140	79	3	663	797	84	201	2.60E-06	46.7	COG0666	Arp	FOG: Ankyrin repeat
LN02_05168∣LN02Chr04:847449-850157(+)∣902	CDD:223738	24.699	166	92	3	552	710	66	205	6.55E-05	42.5	COG0666	Arp	FOG: Ankyrin repeat
LN02_05168∣LN02Chr04:847449-850157(+)∣902	CDD:223738	24.183	153	81	3	750	898	80	201	5.92E-04	39.4	COG0666	Arp	FOG: Ankyrin repeat
LN02_05552∣LN02Chr04:2425993-2428220(+)∣702	CDD:227584	33.672	591	282	16	111	693	32	520	1.42E-135	406	COG5259	RSC8	RSC chromatin remodeling complex subunit RSC8
LN02_05744∣LN02Chr04:3061472-3064008(-)∣797	CDD:227561	34.902	255	158	2	85	339	59	305	5.57E-83	271	COG5236	COG5236	Uncharacterized conserved protein, contains RING Zn-finger
LN02_05808∣LN02Chr04:3268634-3272100(+)∣1134	CDD:223951	17.391	207	160	5	367	564	1	205	1.14E-10	62.7	COG1020	EntF	Non-ribosomal peptide synthetase modules and related proteins
LN02_05808∣LN02Chr04:3268634-3272100(+)∣1134	CDD:225967	30	60	42	0	50	109	2	61	8.70E-05	38.9	COG3433	COG3433	Aryl carrier domain
LN02_06000∣LN02Chr04:3974075-3976106(+)∣604	CDD:224151	33.974	156	95	2	362	517	146	293	2.63E-37	137	COG1230	CzcD	Co/Zn/Cd efflux system component

LN02_06000	LN02Chr04 :3974075-3976106(+)	604	CDD:2241 51	42.063	126	72	1	10	135	21	145	6.18E-37	136	COG12 30	CzcD	Co/Zn/Cd efflux system component
LN02_06128	LN02Chr04 :4363808-4364796(+)	275	CDD:2252 40	31.757	148	93	5	77	216	44	191	2.63E-19	81.7	COG23 65	COG236 5	Protein tyrosine/serine phosphatase
LN02_06256	LN02Chr04 :4809518-4811762(+)	614	CDD:2235 20	48.355	608	274	10	9	614	7	576	0	601	COG04 43	DnaK	Molecular chaperone
LN02_06320	LN02Chr04 :5020543-5022103(-)	381	CDD:2254 43	35.897	39	25	0	140	178	106	144	5.63E-06	44.6	COG28 90	HemK	Methylase of polypeptide chain release factors
LN02_06320	LN02Chr04 :5020543-5022103(-)	381	CDD:2251 36	28.972	107	53	6	145	236	52	150	3.72E-05	41.9	COG22 26	UbiE	Methylase involved in ubiquinone/menaquinone biosynthesis
LN02_06320	LN02Chr04 :5020543-5022103(-)	381	CDD:2235 74	19.068	236	180	3	99	324	3	237	1.57E-04	39.9	COG05 00	SmtA	SAM-dependent methyltransferases
LN02_06384	LN02Chr04 :5216734-5218627(+)	563	CDD:2230 96	30.417	503	251	19	78	563	15	435	1.01E-96	298	COG00 17	AsnS	Aspartyl/asparaginyl-tRNA synthetases
LN02_06576	LN02Chr04 :5904932-5906636(+)	510	CDD:2249 83	25	284	132	10	1	279	3	210	1.69E-23	100	COG20 72	TrkA	Predicted flavoprotein involved in K+ transport
LN02_06768	LN02Chr05 :741531-743321(-)	459	CDD:2259 59	29.778	450	240	8	8	456	1	375	1.03E-122	359	COG34 25	PksG	3-hydroxy-3-methylglutaryl CoA synthase
LN02_06896	LN02Chr05 :1191579-1192778(-)	399	CDD:2236 71	31.461	89	59	1	299	387	31	117	2.07E-14	70.4	COG05 98	CorA	Mg2+ and Co2+ transporters
LN02_07216	LN02Chr05 :3012834-3013954(+)	266	CDD:2233 95	38.567	293	148	6	2	263	243	534	2.80E-73	230	COG03 18	CaiC	Acyl-CoA synthetases (AMP-forming)/AMP-acid ligases II
LN02_07280	LN02Chr05 :3180082-3181392(+)	436	CDD:2230 87	24.941	421	260	13	1	405	92	472	1.19E-74	238	COG00 08	GlnS	Glutamyl- and glutaminyl-tRNA synthetases
LN02_07408	LN02Chr05 :3705638-3707128(+)	496	CDD:2252 01	21.39	374	199	12	73	420	60	364	1.14E-10	60.5	COG23 19	COG231 9	FOG: WD40 repeat
LN02_07536	LN02Chr05 :4094966-4097758(-)	840	CDD:2255 14	27.273	253	177	5	392	642	3	250	6.82E-53	181	COG29 66	COG296 6	Uncharacterized conserved protein
LN02_07536	LN02Chr05 :4094966-4097758(-)	840	CDD:2261 37	22.436	156	109	3	666	821	3	146	4.58E-22	90.8	COG36 10	COG361 0	Uncharacterized conserved protein
LN02_07664	LN02Chr05 :4764913-4765811(+)	138	CDD:2274 50	43.22	118	67	0	3	120	1	118	1.09E-26	94.1	COG51 20	GOT1	Membrane protein involved in Golgi transport
LN02_07728	LN02Chr05 :5021570-5022552(+)	293	CDD:2237 87	20	285	188	13	3	266	33	298	2.71E-13	66.4	COG07 15	TauA	ABC-type nitrate/sulfonate/bicarbonate transport systems, periplasmic components
LN02_07856	LN02Chr05 :5508144-5509125(+)	298	CDD:2235 70	25.909	220	123	11	18	234	2	184	1.86E-07	48.4	COG04 96	SurE	Predicted acid phosphatase
LN02_08112	LN02Chr06 :1285429-1287594(+)	711	CDD:2268 01	32.203	59	29	3	623	670	28	86	2.09E-05	41.3	COG43 57	COG435 7	Zinc finger domain containing protein (CHY type)
LN02_08240	LN02Chr06 :1848576-1853340(-)	1464	CDD:2240 36	23.279	494	319	18	78	551	13	466	4.34E-41	157	COG11 11	MPH1	ERCC4-like helicases
LN02_08240	LN02Chr06 :1848576-1853340(-)	1464	CDD:2236 44	23.664	262	132	8	1155	1409	8	208	1.74E-16	77.7	COG05 71	Rnc	dsRNA-specific ribonuclease
LN02_08240	LN02Chr06 :1848576-1853340(-)	1464	CDD:2236 44	29.586	169	85	6	970	1129	3	146	1.03E-13	69.6	COG05 71	Rnc	dsRNA-specific ribonuclease

LN02_08944∣LN02Chr07:917253-918330(+)∣296	CDD:226174	38.408	289	170	4	9	295	15	297	1.20E-85	256	COG3648	COG3648	Uricase (urate oxidase)
LN02_09328∣LN02Chr07:2223292-2224558(+)∣356	CDD:223730	24.303	251	165	8	111	356	81	311	5.77E-26	103	COG0657	Aes	Esterase/lipase
LN02_00241∣LN02Chr01:1436891-1438500(+)∣472	CDD:225139	40.143	279	139	6	168	446	19	269	2.31E-92	279	COG2230	Cfa	Cyclopropane fatty acid synthase and related methyltransferases
LN02_00305∣LN02Chr01:1609400-1610943(+)∣494	CDD:225490	37.622	513	269	18	2	483	4	496	1.24E-135	398	COG2939	COG2939	Carboxypeptidase C (cathepsin A)
LN02_00433∣LN02Chr01:2101586-2106939(+)∣1741	CDD:227392	31.67	581	320	19	8	576	23	538	6.10E-85	287	COG5059	KIP1	Kinesin-like protein
LN02_00433∣LN02Chr01:2101586-2106939(+)∣1741	CDD:224117	20.346	231	161	7	666	882	647	868	7.77E-06	47.8	COG1196	Smc	Chromosome segregation ATPases
LN02_00497∣LN02Chr01:2293331-2294847(-)∣451	CDD:224105	23.678	397	186	4	27	422	5	285	6.52E-33	123	COG1184	GCD2	Translation initiation factor 2B subunit, eIF-2B alpha/beta/delta family
LN02_00561∣LN02Chr01:2471748-2478671(+)∣2186	CDD:227434	21.641	2181	1464	53	39	2182	8	1980	0	684	COG5103	CDC39	Cell division control protein, negative regulator of transcription
LN02_01009∣LN02Chr01:4084830-4086889(+)∣644	CDD:224192	27.551	98	63	4	327	422	55	146	2.76E-05	43.5	COG1273	COG1273	Ku-homolog
LN02_01137∣LN02Chr01:4516501-4518022(+)∣432	CDD:223420	46.978	364	184	3	42	404	5	360	1.76E-170	479	COG0343	Tgt	Queuine/archaeosine tRNA-ribosyltransferase
LN02_01201∣LN02Chr01:4853114-4854141(+)∣322	CDD:224331	25.784	287	176	8	15	299	18	269	2.03E-10	58	COG1413	COG1413	FOG: HEAT repeat
LN02_01457∣LN02Chr01:5889018-5889776(+)∣252	CDD:224025	27.397	219	113	4	8	213	4	189	3.73E-35	122	COG1100	COG1100	GTPase SAR1 and related small G proteins
LN02_01649∣LN02Chr01:6580108-6583830(-)∣1131	CDD:224471	20.762	814	473	34	109	846	9	726	2.19E-55	204	COG1554	ATH1	Trehalose and maltose hydrolases (possible phosphorylases)
LN02_01713∣LN02Chr01:6833779-6834609(+)∣276	CDD:225714	21.547	181	116	7	53	227	67	227	4.31E-10	56.3	COG3173	COG3173	Predicted aminoglycoside phosphotransferase
LN02_01841∣LN02Chr01:7190239-7191614(+)∣410	CDD:225491	23.669	169	89	9	121	283	342	476	6.45E-05	41.7	COG2940	COG2940	Proteins containing SET domain
LN02_02097∣LN02Chr02:610806-612748(+)∣621	CDD:224117	17.557	131	102	3	230	356	238	366	2.37E-06	47.8	COG1196	Smc	Chromosome segregation ATPases
LN02_02161∣LN02Chr02:777601-782767(-)∣1642	CDD:223627	30.252	714	406	20	284	916	143	845	8.75E-99	336	COG0553	HepA	Superfamily II DNA/RNA helicases, SNF2 family
LN02_02481∣LN02Chr02:1966253-1969697(-)∣1090	CDD:223627	30.769	403	214	14	282	655	294	660	4.11E-51	192	COG0553	HepA	Superfamily II DNA/RNA helicases, SNF2 family
LN02_02481∣LN02Chr02:1966253-1969697(-)∣1090	CDD:223627	42.262	168	93	2	925	1090	701	866	9.04E-29	121	COG0553	HepA	Superfamily II DNA/RNA helicases, SNF2 family
LN02_02481∣LN02Chr02:1966253-1969697(-)∣1090	CDD:227861	23.596	89	51	3	667	750	189	265	5.23E-05	43.3	COG5574	PEX10	RING-finger-containing E3 ubiquitin ligase
LN02_02545∣LN02Chr02:2178947-2179822(+)∣291	CDD:226406	24.427	131	91	2	36	162	723	849	5.72E-04	38.3	COG3889	COG3889	Predicted solute binding protein

LN02_02609∣LN02Chr02:2408922-2410178(-)∣418	CDD:223699	45.547	393	200	6	21	402	6	395	9.42E-167	470	COG0626	MetC	Cystathionine beta-lyases/cystathionine gamma-synthases
LN02_02737∣LN02Chr02:2814674-2817634(-)∣931	CDD:225862	32.071	396	222	12	540	895	39	427	1.12E-74	249	COG3325	ChiA	Chitinase
LN02_02737∣LN02Chr02:2814674-2817634(-)∣931	CDD:227596	16.321	386	280	14	75	453	3909	4258	1.64E-08	55.8	COG5271	MDN1	AAA ATPase containing von Willebrand factor type A (vWA) domain
LN02_02865∣LN02Chr02:3258632-3260471(-)∣534	CDD:225035	24.81	395	215	12	135	526	90	405	2.43E-39	145	COG2124	CypX	Cytochrome P450
LN02_03121∣LN02Chr02:4154011-4154610(+)∣143	CDD:226057	59.574	94	33	1	35	128	5	93	2.56E-45	140	COG3526	COG3526	Uncharacterized protein conserved in bacteria
LN02_03185∣LN02Chr02:4423643-4424458(+)∣271	CDD:223798	29.858	211	133	6	71	271	61	266	6.74E-28	105	COG0726	CDA1	Predicted xylanase/chitin deacetylase
LN02_03377∣LN02Chr02:5031129-5031644(+)∣121	CDD:227530	46.667	105	54	2	14	118	10	112	2.28E-37	120	COG5204	SPT4	Transcription elongation factor SPT4
LN02_03505∣LN02Chr02:5809905-5811432(+)∣475	CDD:225496	25.581	129	71	4	103	230	31	135	1.38E-06	46.2	COG2945	COG2945	Predicted hydrolase of the alpha/beta superfamily
LN02_03633∣LN02Chr03:59417-61019(-)∣516	CDD:223110	51.935	310	135	5	17	325	4	300	1.07E-124	364	COG0031	CysK	Cysteine synthase
LN02_03633∣LN02Chr03:59417-61019(-)∣516	CDD:226147	25.333	150	76	4	361	509	66	180	3.73E-09	53.6	COG3620	COG3620	Predicted transcriptional regulator with C-terminal CBS domains
LN02_04145∣LN02Chr03:2812279-2813739(-)∣486	CDD:223589	25.175	286	195	8	207	479	5	284	7.20E-38	139	COG0515	SPS1	Serine/threonine protein kinase
LN02_04337∣LN02Chr03:3844986-3846367(-)∣419	CDD:223120	26.901	342	198	10	24	347	7	314	3.58E-51	172	COG0042	COG0042	tRNA-dihydrouridine synthase
LN02_04657∣LN02Chr03:4949755-4952645(+)∣940	CDD:223248	30.12	166	91	6	703	864	9	153	5.87E-15	72	COG0170	SEC59	Dolichol kinase
LN02_05169∣LN02Chr04:853644-854763(+)∣304	CDD:226406	20.946	148	104	3	94	241	738	872	2.62E-04	39.5	COG3889	COG3889	Predicted solute binding protein
LN02_05233∣LN02Chr04:1046070-1047775(-)∣469	CDD:227170	23.022	278	176	11	149	407	93	351	2.21E-06	46.5	COG4833	COG4833	Predicted glycosyl hydrolase
LN02_05617∣LN02Chr04:2643492-2645494(-)∣483	CDD:225180	24.581	179	124	3	45	220	38	208	4.41E-06	45.7	COG2271	UhpC	Sugar phosphate permease
LN02_05681∣LN02Chr04:2846876-2849666(+)∣608	CDD:226406	20.958	167	116	4	286	452	701	851	4.20E-04	40.2	COG3889	COG3889	Predicted solute binding protein
LN02_05745∣LN02Chr04:3065258-3066415(-)∣268	CDD:227818	22.134	253	137	5	12	264	6	198	2.69E-24	94.8	COG5531	COG5531	SWIB-domain-containing proteins implicated in chromatin remodeling
LN02_05809∣LN02Chr04:3272684-3274320(-)∣534	CDD:225186	31.295	556	316	20	10	522	4	536	1.43E-101	314	COG2303	BetA	Choline dehydrogenase and related flavoproteins
LN02_05873∣LN02Chr04:3479748-3480438(-)∣197	CDD:223274	31.788	151	71	4	22	172	186	304	6.58E-30	109	COG0196	RibF	FAD synthase
LN02_06001∣LN02Chr04:3977562-3978338(-)∣206	CDD:225816	37.374	99	61	1	36	134	1	98	7.82E-30	103	COG3277	GAR1	RNA-binding protein involved in rRNA processing
LN02_06705∣LN02Chr05:475413-477777(-)∣619	CDD:225371	23.497	183	139	1	83	264	13	195	7.89E-14	70.7	COG2814	AraJ	Arabinose efflux permease

LN02_07281	LN02Chr05:3182485-3184070(+)	453	CDD:224493	30.811	370	176	11	7	374	3	294	2.11E-65	209	COG1577	ERG12	Mevalonate kinase
LN02_07345	LN02Chr05:3404855-3407447(-)	745	CDD:223293	29.022	634	272	20	3	635	1	457	1.13E-128	388	COG0215	CysS	Cysteinyl-tRNA synthetase
LN02_07409	LN02Chr05:3708062-3710653(+)	834	CDD:223769	23.49	149	98	3	72	217	38	173	2.12E-05	44.1	COG0697	RhaT	Permeases of the drug/metabolite transporter (DMT) superfamily
LN02_07729	LN02Chr05:5023559-5025301(+)	449	CDD:223769	17.284	243	178	3	178	419	57	277	2.29E-07	49.1	COG0697	RhaT	Permeases of the drug/metabolite transporter (DMT) superfamily
LN02_08241	LN02Chr06:1854638-1855857(+)	329	CDD:227511	23.958	192	132	5	137	323	178	360	4.40E-12	63.7	COG5184	ATS1	Alpha-tubulin suppressor and related RCC1 domain-containing proteins
LN02_08369	LN02Chr06:2263852-2267975(+)	1295	CDD:227532	32.877	73	45	3	1133	1202	668	739	1.23E-07	53.1	COG5207	UBP14	Isopeptidase T
LN02_08369	LN02Chr06:2263852-2267975(+)	1295	CDD:227409	56.667	30	13	0	604	633	194	223	1.27E-05	46.8	COG5077	COG5077	Ubiquitin carboxyl-terminal hydrolase
LN02_08433	LN02Chr06:2595757-2597065(-)	325	CDD:223089	31.077	325	189	11	4	325	11	303	1.88E-66	208	COG0010	SpeB	Arginase/agmatinase/formimionoglutamate hydrolase, arginase family
LN02_08881	LN02Chr07:677047-679451(+)	616	CDD:223587	27.092	502	306	7	33	531	30	474	1.71E-98	307	COG0513	SrmB	Superfamily II DNA and RNA helicases
LN02_08945	LN02Chr07:918825-919472(-)	179	CDD:223180	48.182	110	57	0	39	148	25	134	1.92E-45	143	COG0102	RplM	Ribosomal protein L13
LN02_09201	LN02Chr07:1732824-1734026(+)	376	CDD:225546	20.541	185	128	6	190	369	65	235	1.35E-19	84.8	COG3000	ERG3	Sterol desaturase
LN02_09201	LN02Chr07:1732824-1734026(+)	376	CDD:227599	23.153	203	59	4	8	210	51	156	1.98E-16	73.3	COG5274	CYB5	Cytochrome b involved in lipid metabolism
LN02_09265	LN02Chr07:1980000-1981499(-)	499	CDD:223738	28.736	87	56	1	199	279	139	225	1.66E-05	43.3	COG0666	Arp	FOG: Ankyrin repeat
LN02_09329	LN02Chr07:2224845-2226589(-)	509	CDD:225139	36.042	283	171	7	192	473	10	283	1.15E-63	206	COG2230	Cfa	Cyclopropane fatty acid synthase and related methyltransferases
LN02_00242	LN02Chr01:1438588-1439667(-)	338	CDD:223916	23.392	171	104	8	123	275	14	175	4.15E-08	50.6	COG0847	DnaQ	DNA polymerase III, epsilon subunit and related 3'-5' exonucleases
LN02_00434	LN02Chr01:2111084-2112611(+)	356	CDD:225136	34.211	38	25	0	112	149	48	85	1.12E-04	39.9	COG2226	UbiE	Methylase involved in ubiquinone/menaquinone biosynthesis
LN02_00498	LN02Chr01:2295913-2297494(+)	495	CDD:227517	41.451	193	96	4	296	486	204	381	4.15E-49	170	COG5190	FCP1	TFIIF-interacting CTD phosphatases, including NLI-interacting factor
LN02_00626	LN02Chr01:2780541-2781570(-)	268	CDD:225220	37.313	134	81	2	131	263	1	132	6.73E-46	147	COG2343	COG2343	Uncharacterized protein conserved in bacteria
LN02_00946	LN02Chr01:3870080-3872315(+)	581	CDD:223232	22.018	545	314	17	57	569	1	466	1.56E-53	186	COG0154	GatA	Asp-tRNAAsn/Glu-tRNAGln amidotransferase A subunit and related amidases
LN02_01074	LN02Chr01:4323855-4326870(+)	861	CDD:225201	22.539	386	274	14	6	373	58	436	3.27E-39	149	COG2319	COG2319	FOG: WD40 repeat

LN02_01138lLN02Chr01: 4519076-4522746(+)l1198	CDD:223201	29.758	289	149	10	310	596	82	318	4.20E-50	178	COG0123	AcuC	Deacetylases, including yeast histone deacetylase and acetoin utilization protein
LN02_01266lLN02Chr01: 5099042-5099884(-)l280	CDD:223698	21.399	243	135	9	27	268	8	195	5.28E-08	49.4	COG0625	Gst	Glutathione S-transferase
LN02_01330lLN02Chr01: 5325212-5327210(+)l643	CDD:223587	35.309	405	216	13	76	477	29	390	2.99E-97	305	COG0513	SrmB	Superfamily II DNA and RNA helicases
LN02_01394lLN02Chr01: 5566604-5569299(-)l795	CDD:223771	23.597	695	379	13	98	791	1	544	2.68E-78	259	COG0699	COG0699	Predicted GTPases (dynamin-related)
LN02_01458lLN02Chr01: 5890448-5892921(-)l744	CDD:224839	44.139	691	365	8	60	744	22	697	0	702	COG1928	PMT1	Dolichyl-phosphate-mannose--protein O-mannosyl transferase
LN02_01650lLN02Chr01: 6584629-6586482(-)l617	CDD:226056	27.669	459	263	15	134	568	206	619	1.34E-50	183	COG3525	Chb	N-acetyl-beta-hexosaminidase
LN02_01842lLN02Chr01: 7192165-7193453(+)l355	CDD:223730	28.829	222	137	7	109	325	81	286	3.99E-26	103	COG0657	Aes	Esterase/lipase
LN02_02034lLN02Chr02: 387987-389652(+)l461	CDD:226700	18.974	195	124	8	166	353	2	169	1.15E-04	41.3	COG4249	COG4249	Uncharacterized protein containing caspase domain
LN02_02098lLN02Chr02: 614556-616581(-)l367	CDD:223861	20	170	100	5	179	345	85	221	5.39E-17	77.4	COG0790	COG0790	FOG: TPR repeat, SEL1 subfamily
LN02_02162lLN02Chr02: 784576-786709(+)l608	CDD:224166	56.951	597	251	3	4	600	1	591	0	1000	COG1245	COG1245	Predicted ATPase, RNase L inhibitor (RLI) homolog
LN02_02226lLN02Chr02: 1002348-1003195(-)l262	CDD:224442	28.962	183	94	8	81	259	38	188	3.31E-20	82.6	COG1525	COG1525	Micrococcal nuclease (thermonuclease) homologs
LN02_02354lLN02Chr02: 1509327-1510363(-)l299	CDD:224456	21.311	122	78	3	172	284	4	116	1.69E-09	51.8	COG1539	FolB	Dihydroneopterin aldolase
LN02_02546lLN02Chr02: 2183332-2189454(+)l2009	CDD:224117	18.883	376	251	13	1289	1655	155	485	8.08E-18	87.5	COG1196	Smc	Chromosome segregation ATPases
LN02_02546lLN02Chr02: 2183332-2189454(+)l2009	CDD:224117	18.384	359	237	9	1490	1831	172	491	4.96E-06	48.6	COG1196	Smc	Chromosome segregation ATPases
LN02_02546lLN02Chr02: 2183332-2189454(+)l2009	CDD:224117	17.914	374	266	10	1073	1446	679	1011	4.34E-04	42.4	COG1196	Smc	Chromosome segregation ATPases
LN02_02738lLN02Chr02: 2819636-2822188(-)l825	CDD:223594	23.195	457	259	21	23	460	19	402	4.89E-29	117	COG0520	csdA	Selenocysteine lyase/Cysteine desulfurase
LN02_02738lLN02Chr02: 2819636-2822188(-)l825	CDD:225758	25.559	313	160	13	487	795	3	246	5.77E-24	99.4	COG3217	COG3217	Uncharacterized Fe-S protein
LN02_02802lLN02Chr02: 3040257-3056915(-)l5465	CDD:224495	26.056	142	96	5	5003	5142	12	146	3.68E-05	45.8	COG1579	COG1579	Zn-ribbon protein, possibly nucleic acid-binding
LN02_02930lLN02Chr02: 3497865-3498938(+)l357	CDD:226314	23.77	122	83	4	11	132	4	115	3.06E-11	57.4	COG3791	COG3791	Uncharacterized conserved protein
LN02_02930lLN02Chr02: 3497865-3498938(+)l357	CDD:226314	24.427	131	68	4	175	305	2	101	6.55E-05	39.3	COG3791	COG3791	Uncharacterized conserved protein
LN02_03122lLN02Chr02: 4155654-4157132(-)l324	CDD:223712	37.662	154	86	7	130	274	3	155	2.72E-37	128	COG0639	ApaH	Diadenosine tetraphosphatase and related serine/threonine protein phosphatases

LN02_03506|LN02Chr02: 5812065-5813900(+)|567	CDD:223944	47.791	498	230	7	69	564	1	470	0	517	COG1012	PutA	NAD-dependent aldehyde dehydrogenases
LN02_03634|LN02Chr03: 63141-64811(+)|489	CDD:225143	29.179	329	209	10	135	449	90	408	8.42E-44	157	COG2234	Iap	Predicted aminopeptidases
LN02_03954|LN02Chr03: 1235175-1236996(-)|503	CDD:225180	20.93	215	157	6	29	242	10	212	7.93E-11	61.1	COG2271	UhpC	Sugar phosphate permease
LN02_04018|LN02Chr03: 1458998-1460949(-)|582	CDD:223222	21.226	424	240	14	94	502	10	354	8.80E-49	170	COG0144	Sun	tRNA and rRNA cytosine-C5-methylases
LN02_04146|LN02Chr03: 2819868-2822423(-)|698	CDD:223589	25.464	377	208	11	83	431	2	333	1.13E-43	159	COG0515	SPS1	Serine/threonine protein kinase
LN02_04274|LN02Chr03: 3614022-3614759(+)|170	CDD:227528	58.282	163	60	3	9	170	3	158	9.53E-66	195	COG5201	SKP1	SCF ubiquitin ligase, SKP1 component
LN02_04594|LN02Chr03: 4728207-4730402(-)|605	CDD:223232	29.098	488	289	18	109	569	8	465	2.04E-73	240	COG0154	GatA	Asp-tRNAAsn/Glu-tRNAGln amidotransferase A subunit and related amidases
LN02_04658|LN02Chr03: 4953013-4954903(-)|590	CDD:227862	25.815	368	242	8	225	589	193	532	1.17E-55	194	COG5575	ORC2	Origin recognition complex, subunit 2
LN02_04786|LN02Chr03: 5407529-5408041(-)|170	CDD:224934	34.667	75	39	1	69	143	31	95	1.13E-13	60.9	COG2023	RPR2	RNase P subunit RPR2
LN02_05106|LN02Chr04: 627330-630315(+)|904	CDD:227445	34.091	44	27	1	394	435	8	51	4.34E-06	47	COG5114	COG5114	Histone acetyltransferase complex SAGA/ADA, subunit ADA2
LN02_05170|LN02Chr04: 855409-856660(-)|323	CDD:223169	23.485	132	73	3	157	288	17	120	4.82E-09	50.7	COG0091	RplV	Ribosomal protein L22
LN02_05426|LN02Chr04: 1721892-1724192(-)|472	CDD:223906	36.55	342	202	7	109	447	2	331	1.78E-103	309	COG0836	{ManC	Mannose-1-phosphate guanylyltransferase
LN02_05554|LN02Chr04: 2433224-2435357(+)|686	CDD:225297	21.111	90	63	1	580	669	94	175	9.34E-08	49.8	COG2453	CDC14	Predicted protein-tyrosine phosphatase
LN02_05682|LN02Chr04: 2855857-2857756(+)|557	CDD:225371	26.966	89	64	1	66	154	54	141	2.05E-09	56.5	COG2814	AraJ	Arabinose efflux permease
LN02_05810|LN02Chr04: 3276678-3278222(+)|514	CDD:223944	42.017	476	254	8	29	502	15	470	4.35E-152	440	COG1012	PutA	NAD-dependent aldehyde dehydrogenases
LN02_06002|LN02Chr04: 3979022-3980116(+)|336	CDD:224274	28.07	342	174	10	1	334	1	278	7.88E-54	175	COG1355	COG1355	Predicted dioxygenase
LN02_06322|LN02Chr04: 5026337-5028181(+)|614	CDD:223560	47.059	68	29	2	7	72	5	67	1.36E-15	75.7	COG0484	DnaJ	DnaJ-class molecular chaperone with C-terminal Zn finger domain
LN02_06450|LN02Chr04: 5423191-5425072(+)|515	CDD:225035	20.358	447	284	17	56	494	29	411	1.45E-28	114	COG2124	CypX	Cytochrome P450
LN02_06770|LN02Chr05: 747298-749199(-)|633	CDD:223540	24.675	231	115	6	286	484	268	471	1.47E-17	82.9	COG0464	SpoVK	ATPases of the AAA+ class
LN02_06834|LN02Chr05: 981023-984587(-)|1094	CDD:225857	26.894	264	172	12	702	959	3	251	8.48E-29	116	COG3320	COG3320	Putative dehydrogenase domain of multifunctional non-ribosomal peptide synthetases and related enzymes
LN02_06834|LN02Chr05: 981023-984587(-)|1094	CDD:223395	19.883	513	355	23	59	543	43	527	1.20E-22	100	COG0318	CaiC	Acyl-CoA synthetases (AMP-forming)/AMP-acid ligases II

LN02_06962∣LN02Chr05: 1802025-1803988(+)∣569	CDD:223725	43.871	155	81	4	319	470	1	152	5.47E-56	182	COG0652	PpiB	Peptidyl-prolyl cis-trans isomerase (rotamase) - cyclophilin family
LN02_07090∣LN02Chr05: 2540096-2541553(-)∣485	CDD:223616	46.944	409	193	5	42	448	395	781	3.11E-152	450	COG0542	clpA	ATP-binding subunits of Clp protease and DnaK/DnaJ chaperones
LN02_07154∣LN02Chr05: 2751133-2752008(-)∣246	CDD:223213	28.448	232	93	8	59	244	3	207	8.64E-24	92.3	COG0135	TrpF	Phosphoribosylanthranilate isomerase
LN02_07154∣LN02Chr05: 2751133-2752008(-)∣246	CDD:223212	48	25	13	0	19	43	229	253	7.20E-04	36.8	COG0134	TrpC	Indole-3-glycerol phosphate synthase
LN02_07346∣LN02Chr05: 3408097-3409026(+)∣309	CDD:225317	27.523	218	97	5	54	270	24	181	2.04E-39	136	COG2519	GCD14	tRNA(1-methyladenosine) methyltransferase and related methyltransferases
LN02_07410∣LN02Chr05: 3711552-3714767(+)∣1071	CDD:224441	20.833	288	165	9	125	366	24	294	1.50E-23	102	COG1524	COG1524	Uncharacterized proteins of the AP superfamily
LN02_07410∣LN02Chr05: 3711552-3714767(+)∣1071	CDD:225661	24.828	145	100	4	323	460	266	408	8.53E-07	49.8	COG3119	AslA	Arylsulfatase A and related enzymes
LN02_07538∣LN02Chr05: 4099537-4103329(+)∣1234	CDD:223163	23.826	1150	692	34	23	1121	11	1027	0	766	COG0085	RpoB	DNA-directed RNA polymerase, beta subunit/140 kD subunit
LN02_07986∣LN02Chr05: 5873562-5874588(+)∣322	CDD:223738	25.714	210	119	5	113	320	26	200	1.79E-06	45.2	COG0666	Arp	FOG: Ankyrin repeat
LN02_08178∣LN02Chr06: 1686923-1688730(-)∣579	CDD:225035	25.786	159	89	4	414	571	280	410	2.13E-09	56.7	COG2124	CypX	Cytochrome P450
LN02_08242∣LN02Chr06: 1855933-1857062(-)∣277	CDD:223711	37.41	278	125	7	3	277	1	232	1.30E-72	219	COG0638	PRE1	20S proteasome, alpha and beta subunits
LN02_08370∣LN02Chr06: 2269955-2270791(-)∣278	CDD:223560	32.432	74	38	3	62	133	7	70	1.97E-10	57.6	COG0484	DnaJ	DnaJ-class molecular chaperone with C-terminal Zn finger domain
LN02_08626∣LN02Chr06: 3198168-3199552(-)∣281	CDD:226735	23.502	217	131	8	19	215	4	205	7.55E-12	60.9	COG4285	COG4285	Uncharacterized conserved protein
LN02_08690∣LN02Chr06: 3553307-3555152(+)∣563	CDD:225371	20.321	187	148	1	62	247	13	199	7.01E-12	64.2	COG2814	AraJ	Arabinose efflux permease
LN02_08882∣LN02Chr07: 681857-683466(+)∣470	CDD:225182	26.02	196	116	7	128	315	117	291	2.78E-24	100	COG2273	SKN1	Beta-glucanase/Beta-glucan synthetase
LN02_08946∣LN02Chr07: 924985-926905(+)∣507	CDD:223533	24	125	85	2	4	123	165	284	7.04E-06	44.8	COG0457	NrfG	FOG: TPR repeat
LN02_09202∣LN02Chr07: 1734729-1736683(+)∣570	CDD:223796	24.378	201	145	2	167	366	4	198	6.92E-17	78.8	COG0724	COG0724	RNA-binding proteins (RRM domain)
LN02_00070∣LN02Chr01: 389529-389915(-)∣128	CDD:227410	45.238	84	43	2	35	118	72	152	1.43E-23	86.5	COG5078	COG5078	Ubiquitin-protein ligase
LN02_00326∣LN02Chr01: 1783730-1786165(-)∣658	CDD:223808	27.413	518	331	18	16	507	10	508	5.92E-62	212	COG0737	UshA	5'-nucleotidase/2',3'-cyclic phosphodiesterase and related esterases
LN02_00518∣LN02Chr01: 2353169-2355207(-)∣576	CDD:223903	51.462	513	233	7	63	573	41	539	0	594	COG0833	LysP	Amino acid transporters
LN02_00582∣LN02Chr01: 2610963-2613081(+)∣668	CDD:225490	23.158	475	257	23	76	523	87	480	1.93E-30	122	COG2939	COG2939	Carboxypeptidase C (cathepsin A)
LN02_00838∣LN02Chr01: 3549511-3550890(+)∣404	CDD:227577	33.784	296	140	6	1	289	1	247	8.94E-55	180	COG5252	COG5252	Uncharacterized conserved protein, contains CCCH-type Zn-finger protein

LN02_00966∣LN02Chr01:3952392-3954312(+)∣502	CDD:225180	23.239	284	192	8	98	371	68	335	1.86E-09	56.5	COG2271	UhpC	Sugar phosphate permease
LN02_01030∣LN02Chr01:4156631-4159387(+)∣918	CDD:223096	35.189	449	253	14	156	588	7	433	7.70E-122	374	COG0017	AsnS	Aspartyl/asparaginyl-tRNA synthetases
LN02_01030∣LN02Chr01:4156631-4159387(+)∣918	CDD:225451	20.846	331	212	18	602	901	202	513	1.32E-07	52.3	COG2898	COG2898	Uncharacterized conserved protein
LN02_01222∣LN02Chr01:4926015-4929891(+)∣1239	CDD:227559	23.364	535	284	16	55	584	27	440	5.76E-20	93.5	COG5234	CIN1	Beta-tubulin folding cofactor D
LN02_01286∣LN02Chr01:5153900-5154814(+)∣280	CDD:223637	24.051	237	94	4	8	239	2	157	5.32E-34	118	COG0563	Adk	Adenylate kinase and related kinases
LN02_01478∣LN02Chr01:5945788-5952477(+)∣2110	CDD:223147	56.855	496	200	5	761	1253	1	485	0	746	COG0069	GltB	Glutamate synthase domain 2
LN02_01478∣LN02Chr01:5945788-5952477(+)∣2110	CDD:223567	40.987	466	253	9	1624	2083	1	450	3.32E-168	520	COG0493	GltD	NADPH-dependent glutamate synthase beta chain and related oxidoreductases
LN02_01478∣LN02Chr01:5945788-5952477(+)∣2110	CDD:223145	47.222	396	183	5	40	435	1	370	2.83E-166	511	COG0067	GltB	Glutamate synthase domain 1
LN02_01478∣LN02Chr01:5945788-5952477(+)∣2110	CDD:223148	54.138	290	124	4	1261	1549	3	284	7.37E-127	398	COG0070	GltB	Glutamate synthase domain 3
LN02_01670∣LN02Chr01:6696751-6699121(-)∣762	CDD:224117	16.477	352	257	7	99	422	659	1001	5.15E-11	63.2	COG1196	Smc	Chromosome segregation ATPases
LN02_01670∣LN02Chr01:6696751-6699121(-)∣762	CDD:224117	17.481	389	297	9	285	668	157	526	1.89E-05	45.1	COG1196	Smc	Chromosome segregation ATPases
LN02_01798∣LN02Chr01:7060161-7061099(-)∣259	CDD:223959	25.283	265	170	6	1	253	1	249	4.92E-38	131	COG1028	FabG	Dehydrogenases with different specificities (related to short-chain alcohol dehydrogenases)
LN02_01862∣LN02Chr01:7245203-7246767(+)∣409	CDD:223999	21.393	201	126	6	144	339	88	261	3.07E-08	51.6	COG1073	COG1073	Hydrolases of the alpha/beta superfamily
LN02_01926∣LN02Chr01:7426314-7427545(+)∣359	CDD:225987	22.4	125	84	3	194	305	120	244	4.78E-04	38.6	COG3456	COG3456	Predicted component of the type VI protein secretion system, contains a FHA domain
LN02_02182∣LN02Chr02:837556-843480(-)∣1866	CDD:227546	19.114	1873	1192	59	40	1856	11	1616	3.88E-124	427	COG5221	DOP1	Dopey and related predicted leucine zipper transcription factors
LN02_02246∣LN02Chr02:1058219-1059058(-)∣279	CDD:224727	20.635	252	151	9	10	259	11	215	4.31E-09	52.8	COG1814	COG1814	Uncharacterized membrane protein
LN02_02374∣LN02Chr02:1626338-1631380(+)∣1663	CDD:223588	39.107	560	306	14	779	1331	3	534	5.68E-178	544	COG0514	RecQ	Superfamily II DNA helicase
LN02_02438∣LN02Chr02:1820114-1820709(+)∣166	CDD:223391	33.813	139	90	2	16	153	5	142	8.54E-36	118	COG0314	MoaE	Molybdopterin converting factor, large subunit
LN02_02694∣LN02Chr02:2703155-2704481(-)∣415	CDD:224814	40.318	377	189	9	32	401	1	348	2.81E-128	371	COG1902	NemA	NADH:flavin oxidoreductases, Old Yellow Enzyme family
LN02_02886∣LN02Chr02:3331475-3334474(+)∣948	CDD:225689	15.574	122	84	4	60	179	53	157	3.18E-05	43.3	COG3147	DedD	Uncharacterized protein conserved in bacteria
LN02_02950∣LN02Chr02:3565266-3566750(+)∣469	CDD:223796	22.167	203	136	2	1	181	104	306	2.22E-19	85.8	COG0724	COG0724	RNA-binding proteins (RRM domain)

LN02_03142|LN02Chr02: 4285454-4287300(+)|558	CDD:2231 20	32.283	254	147	7	34	283	8	240	2.24E-55	186	COG00 42	COG004 2	tRNA-dihydrouridine synthase
LN02_03206|LN02Chr02: 4490752-4494075(+)|1012	CDD:2235 89	31.126	302	187	9	71	354	2	300	2.43E-51	183	COG05 15	SPS1	Serine/threonine protein kinase
LN02_03398|LN02Chr02: 5093938-5095250(-)|400	CDD:2235 89	26.316	323	202	9	63	373	1	299	3.55E-47	162	COG05 15	SPS1	Serine/threonine protein kinase
LN02_03654|LN02Chr03: 140136-141197(+)|213	CDD:2242 12	28.205	78	50	3	16	92	452	524	1.95E-04	38.5	COG12 93	COG129 3	Predicted RNA-binding protein homologous to eukaryotic snRNP
LN02_03718|LN02Chr03: 401436-402593(-)|346	CDD:2239 92	37.069	348	186	9	4	334	1	332	4.92E-97	288	COG10 64	AdhP	Zn-dependent alcohol dehydrogenases
LN02_03782|LN02Chr03: 738589-740201(-)|444	CDD:2277 21	22.154	325	185	11	69	348	97	398	1.73E-08	53.6	COG54 34	PGU1	Endopygalactorunase
LN02_03846|LN02Chr03: 919764-920589(+)|226	CDD:2231 88	44.531	128	69	2	84	210	53	179	6.98E-40	133	COG01 10	WbbJ	Acetyltransferase (isoleucine patch superfamily)
LN02_04038|LN02Chr03: 1553030-1557425(+)|1353	CDD:2235 50	23.412	1055	580	25	232	1276	47	883	0	561	COG04 74	MgtA	Cation transport ATPase
LN02_04102|LN02Chr03: 2370927-2373059(-)|648	CDD:2273 58	27.874	348	178	10	107	383	71	416	4.00E-36	140	COG50 25	COG502 5	Transcription factor of the Forkhead/HNF3 family
LN02_04166|LN02Chr03: 2934540-2938325(-)|1176	CDD:2274 30	21.355	679	483	18	289	938	120	776	5.86E-77	268	COG50 99	COG509 9	RNA-binding protein of the Puf family, translational repressor
LN02_04294|LN02Chr03: 3694681-3696918(+)|644	CDD:2254 49	40.379	317	173	7	56	370	2	304	2.03E-100	306	COG28 96	MoaA	Molybdenum cofactor biosynthesis enzyme
LN02_04294|LN02Chr03: 3694681-3696918(+)|644	CDD:2233 92	55.128	156	68	2	489	644	4	157	3.15E-66	211	COG03 15	MoaC	Molybdenum cofactor biosynthesis enzyme
LN02_04358|LN02Chr03: 3903656-3905285(-)|397	CDD:2235 87	44.737	380	204	4	15	388	20	399	3.03E-140	406	COG05 13	SrmB	Superfamily II DNA and RNA helicases
LN02_04614|LN02Chr03: 4807487-4809315(+)|312	CDD:2279 35	22.581	155	100	4	118	264	58	200	7.19E-12	61	COG56 48	NHP6B	Chromatin-associated proteins containing the HMG domain
LN02_04678|LN02Chr03: 5045691-5047717(-)|624	CDD:2278 63	46.552	58	31	0	76	133	59	116	6.93E-20	84	COG55 76	COG557 6	Homeodomain-containing transcription factor
LN02_04678|LN02Chr03: 5045691-5047717(-)|624	CDD:2261 93	22.917	144	87	5	328	466	33	157	7.16E-05	42.2	COG36 67	PcoB	Uncharacterized protein involved in copper resistance
LN02_04742|LN02Chr03: 5240894-5241814(-)|229	CDD:2243 08	30.622	209	129	2	12	220	1	193	5.23E-28	102	COG13 90	NtpE	Archaeal/vacuolar-type H+-ATPase subunit E
LN02_04998|LN02Chr04: 306170-307898(+)|427	CDD:2273 68	49.215	382	171	7	21	402	12	370	1.53E-158	449	COG50 35	CDC50	Cell cycle control protein
LN02_05062|LN02Chr04: 491722-493849(+)|621	CDD:2256 05	27.742	155	107	2	416	570	49	198	2.01E-12	64.8	COG30 63	PilF	Tfp pilus assembly protein PilF
LN02_05062|LN02Chr04: 491722-493849(+)|621	CDD:2271 24	22.989	174	112	7	336	497	101	264	3.37E-04	39.8	COG47 85	NlpI	Lipoprotein NlpI, contains TPR repeats
LN02_05190|LN02Chr04: 908597-916770(-)|2682	CDD:2239 51	23.134	670	443	16	353	998	19	640	7.12E-83	284	COG10 20	EntF	Non-ribosomal peptide synthetase modules and related proteins
LN02_05190|LN02Chr04: 908597-916770(-)|2682	CDD:2239 51	20	185	141	3	1926	2103	12	196	2.23E-12	69.6	COG10 20	EntF	Non-ribosomal peptide synthetase modules and related proteins

LN02_05190|LN02Chr04: 908597-916770(-)|2682	CDD:223395	20.438	137	93	4	2450	2586	403	523	3.57E-04	42.8	COG0318	CaiC	Acyl-CoA synthetases (AMP-forming)/AMP-acid ligases II
LN02_05254|LN02Chr04: 1116737-1117439(-)|161	CDD:223894	28.777	139	91	4	16	151	4	137	1.53E-23	86.9	COG0824	FcbC	Predicted thioesterase
LN02_05318|LN02Chr04: 1354449-1358230(-)|1114	CDD:223760	31.66	259	147	8	788	1038	3	239	1.87E-60	204	COG0688	Psd	Phosphatidylserine decarboxylase
LN02_05318|LN02Chr04: 1354449-1358230(-)|1114	CDD:227371	27.344	128	80	5	45	168	1042	1160	8.35E-08	53.6	COG5038	COG5038	Ca2+-dependent lipid-binding protein, contains C2 domain
LN02_05318|LN02Chr04: 1354449-1358230(-)|1114	CDD:227371	26.606	109	68	5	271	378	1038	1135	1.74E-06	49.4	COG5038	COG5038	Ca2+-dependent lipid-binding protein, contains C2 domain
LN02_05638|LN02Chr04: 2712412-2714176(-)|544	CDD:224871	33.744	406	237	12	140	535	5	388	1.42E-89	278	COG1960	CaiA	Acyl-CoA dehydrogenases
LN02_05638|LN02Chr04: 2712412-2714176(-)|544	CDD:227599	38.095	84	51	1	1	84	48	130	8.76E-17	75.2	COG5274	CYB5	Cytochrome b involved in lipid metabolism
LN02_05894|LN02Chr04: 3548451-3550390(+)|465	CDD:225180	22.663	353	239	11	75	411	49	383	1.19E-07	50.7	COG2271	UhpC	Sugar phosphate permease
LN02_06022|LN02Chr04: 4028319-4031147(-)|774	CDD:223589	21.429	252	167	9	47	272	9	255	4.08E-12	65.5	COG0515	SPS1	Serine/threonine protein kinase
LN02_06150|LN02Chr04: 4427665-4430164(-)|596	CDD:225779	19.205	302	201	9	262	555	38	304	1.99E-22	96	COG3239	DesA	Fatty acid desaturase
LN02_06470|LN02Chr04: 5486503-5488513(-)|546	CDD:225129	24.658	365	228	11	78	438	1	322	1.04E-41	151	COG2219	PRI2	Eukaryotic-type DNA primase, large subunit
LN02_06982|LN02Chr05: 1857106-1860315(+)|876	CDD:223589	23.418	316	192	11	343	643	2	282	6.01E-30	119	COG0515	SPS1	Serine/threonine protein kinase
LN02_07174|LN02Chr05: 2810408-2813301(+)|844	CDD:225491	23.776	286	205	5	234	506	1	286	5.07E-17	81.8	COG2940	COG2940	Proteins containing SET domain
LN02_07174|LN02Chr05: 2810408-2813301(+)|844	CDD:225491	27.941	204	124	7	98	279	278	480	1.89E-15	76.8	COG2940	COG2940	Proteins containing SET domain
LN02_07238|LN02Chr05: 3055372-3055966(+)|158	CDD:227410	43.21	162	71	2	1	154	1	149	1.34E-63	189	COG5078	COG5078	Ubiquitin-protein ligase
LN02_07302|LN02Chr05: 3270896-3273885(+)|917	CDD:224389	27.926	376	218	10	127	491	55	388	8.86E-58	201	COG1472	BglX	Beta-glucosidase-related glycosidases
LN02_07430|LN02Chr05: 3766519-3767955(+)|446	CDD:223704	21.429	210	140	7	129	323	16	215	9.48E-13	65.1	COG0631	PTC1	Serine/threonine protein phosphatase
LN02_07494|LN02Chr05: 3964091-3965789(-)|458	CDD:223575	24.555	281	183	9	161	431	33	294	8.80E-26	103	COG0501	HtpX	Zn-dependent protease with chaperone function
LN02_07558|LN02Chr05: 4151839-4154430(+)|863	CDD:223740	19.095	199	140	7	519	715	112	291	6.54E-13	67.6	COG0668	MscS	Small-conductance mechanosensitive channel
LN02_07686|LN02Chr05: 4840931-4842216(-)|402	CDD:227531	64.982	277	92	2	31	307	26	297	3.25E-146	417	COG5206	GPI8	Glycosylphosphatidylinositol transamidase (GPIT), subunit GPI8
LN02_07750|LN02Chr05: 5077694-5079116(+)|423	CDD:223745	20.096	418	247	14	4	414	4	341	1.16E-23	98.4	COG0673	MviM	Predicted dehydrogenases and related proteins
LN02_07814|LN02Chr05: 5370866-5372750(-)|589	CDD:225251	40.597	335	179	7	4	337	2	317	1.74E-72	232	COG2376	DAK1	Dihydroxyacetone kinase

LN02_07878丨LN02Chr05: 5573022-5574595(-)丨453	CDD:2239 91	29.397	398	224	13	40	433	2	346	1.26E-78	245	COG10 63	Tdh	Threonine dehydrogenase and related Zn-dependent dehydrogenases
LN02_08262丨LN02Chr06: 1922561-1923630(+)丨318	CDD:2278 84	25.116	215	107	8	91	291	148	322	7.64E-14	68.4	COG55 97	COG559 7	Alpha-N-acetylglucosamine transferase
LN02_08390丨LN02Chr06: 2433400-2436761(+)丨957	CDD:2260 98	25.157	159	103	8	687	833	15	169	1.29E-08	53.9	COG35 68	ElsH	Metal-dependent hydrolase
LN02_08454丨LN02Chr06: 2664958-2665644(-)丨110	CDD:2248 23	51.02	98	48	0	8	105	1	98	5.35E-47	143	COG19 11	RPL30	Ribosomal protein L30E
LN02_08518丨LN02Chr06: 2854026-2856969(+)丨924	CDD:2241 36	16.857	350	236	10	348	695	115	411	2.48E-26	110	COG12 15	COG121 5	Glycosyltransferases, probably involved in cell wall biogenesis
LN02_08582丨LN02Chr06: 3074209-3076340(-)丨702	CDD:2233 62	30.556	468	242	23	1	434	1	419	2.39E-72	238	COG02 85	FolC	Folylpolyglutamate synthase
LN02_08646丨LN02Chr06: 3287015-3290950(+)丨1259	CDD:2237 15	24.606	317	147	6	578	894	110	334	1.30E-28	115	COG06 42	BaeS	Signal transduction histidine kinase
LN02_08646丨LN02Chr06: 3287015-3290950(+)丨1259	CDD:2238 55	32.203	118	73	4	1133	1249	4	115	4.18E-22	90.3	COG07 84	CheY	FOG: CheY-like receiver
LN02_08646丨LN02Chr06: 3287015-3290950(+)丨1259	CDD:2251 13	23.037	191	125	4	34	220	3	175	9.48E-09	53.4	COG22 03	FhlA	FOG: GAF domain
LN02_08710丨LN02Chr06: 3650904-3652402(+)丨481	CDD:2237 25	35.593	177	92	7	2	177	1	156	4.07E-43	146	COG06 52	PpiB	Peptidyl-prolyl cis-trans isomerase (rotamase) - cyclophilin family
LN02_08710丨LN02Chr06: 3650904-3652402(+)丨481	CDD:2237 96	25.517	145	108	0	209	353	74	218	4.37E-18	81.9	COG07 24	COG072 4	RNA-binding proteins (RRM domain)
LN02_08774丨LN02Chr07: 312759-314670(+)丨527	CDD:2243 74	24.948	477	294	11	75	527	6	442	3.00E-44	159	COG14 57	CodB	Purine-cytosine permease and related proteins
LN02_08966丨LN02Chr07: 988521-989365(-)丨172	CDD:2240 83	30.435	115	76	1	58	172	1	111	1.33E-27	102	COG11 61	COG116 1	Predicted GTPases
LN02_09286丨LN02Chr07: 2033892-2034933(+)丨303	CDD:2270 56	21.805	133	67	6	145	258	4	118	8.33E-04	37.3	COG47 12	COG471 2	Uncharacterized protein conserved in bacteria
LN02_00243丨LN02Chr01: 1439979-1440966(+)丨270	CDD:2273 60	26.941	219	95	4	31	249	9	162	2.96E-31	116	COG50 27	SAS2	Histone acetyltransferase (MYST family)
LN02_00563丨LN02Chr01: 2485540-2486824(-)丨386	CDD:2251 36	25.926	162	72	5	152	312	42	156	1.55E-05	43	COG22 26	UbiE	Methylase involved in ubiquinone/menaquinone biosynthesis
LN02_00627丨LN02Chr01: 2785656-2787095(+)丨413	CDD:2235 76	48.943	331	161	6	76	404	6	330	1.86E-148	422	COG05 02	BioB	Biotin synthase and related enzymes
LN02_00755丨LN02Chr01: 3252264-3259292(-)丨1988	CDD:2273 54	27.016	496	318	10	1502	1987	411	872	3.75E-92	317	COG50 21	HUL4	Ubiquitin-protein ligase
LN02_00755丨LN02Chr01: 3252264-3259292(-)丨1988	CDD:2273 96	25.366	205	143	6	294	491	71	272	1.21E-06	50.3	COG50 64	SRP1	Karyopherin (importin) alpha
LN02_01139丨LN02Chr01: 4523109-4524269(-)丨290	CDD:2243 05	17.293	266	172	10	1	255	1	229	5.34E-24	94.7	COG13 87	HIS2	Histidinol phosphatase and related hydrolases of the PHP family
LN02_01203丨LN02Chr01: 4863223-4865110(+)丨601	CDD:2234 64	33.01	412	226	5	179	589	6	368	1.65E-96	296	COG03 87	ChaA	Ca2+/H+ antiporter
LN02_01267丨LN02Chr01: 5101537-5103546(+)丨565	CDD:2233 95	22.202	545	347	22	17	527	17	518	3.55E-56	194	COG03 18	CaiC	Acyl-CoA synthetases (AMP-forming)/AMP-acid ligases II

LN02_01331∣LN02Chr01: 5327797-5329119(-)∣351	CDD:225201	24.567	289	179	9	58	343	105	357	1.82E-24	100	COG2319	COG2319	FOG: WD40 repeat
LN02_01459∣LN02Chr01: 5894914-5896936(+)∣650	CDD:226801	34.615	52	29	2	258	304	15	66	1.92E-05	41.3	COG4357	COG4357	Zinc finger domain containing protein (CHY type)
LN02_01459∣LN02Chr01: 5894914-5896936(+)∣650	CDD:227568	22.34	94	60	2	349	433	256	345	2.41E-04	41.1	COG5243	HRD1	HRD ubiquitin ligase complex, ER membrane component
LN02_01523∣LN02Chr01: 6099599-6101577(-)∣546	CDD:223535	32.264	530	333	15	8	527	1	514	5.13E-144	422	COG0459	GroL	Chaperonin GroEL (HSP60 family)
LN02_01843∣LN02Chr01: 7194345-7197700(+)∣1048	CDD:227392	36.044	541	297	10	55	585	16	517	4.48E-137	422	COG5059	KIP1	Kinesin-like protein
LN02_01971∣LN02Chr02: 151967-153581(-)∣493	CDD:227488	21.875	224	167	5	199	415	164	386	9.68E-16	76.1	COG5159	RPN6	26S proteasome regulatory complex component
LN02_02035∣LN02Chr02: 390470-392719(-)∣726	CDD:225882	33.631	672	397	10	64	726	56	687	1.33E-156	467	COG3345	GalA	Alpha-galactosidase
LN02_02099∣LN02Chr02: 617673-619322(-)∣418	CDD:227416	22.609	115	79	5	232	337	106	219	4.25E-06	45.2	COG5084	YTH1	Cleavage and polyadenylation specificity factor (CPSF) Clipper subunit and related makorin family Zn-finger proteins
LN02_02163∣LN02Chr02: 787574-790513(-)∣857	CDD:227435	25.258	582	382	10	8	575	13	555	8.77E-95	307	COG5104	PRP40	Splicing factor
LN02_02547∣LN02Chr02: 2190660-2192827(-)∣661	CDD:223626	34.036	332	184	7	334	659	38	340	1.92E-87	274	COG0552	FtsY	Signal recognition particle GTPase
LN02_02611∣LN02Chr02: 2411343-2414406(+)∣579	CDD:223578	46.655	568	252	14	1	557	1	528	0	729	COG0504	PyrG	CTP synthase (UTP-ammonia lyase)
LN02_02675∣LN02Chr02: 2627485-2634952(-)∣2194	CDD:227223	27.097	310	220	5	769	1075	44	350	6.84E-26	109	COG4886	COG4886	Leucine-rich repeat (LRR) protein
LN02_02675∣LN02Chr02: 2627485-2634952(-)∣2194	CDD:227223	32.402	179	107	9	1253	1428	117	284	1.27E-09	59.6	COG4886	COG4886	Leucine-rich repeat (LRR) protein
LN02_02675∣LN02Chr02: 2627485-2634952(-)∣2194	CDD:223704	22.344	273	174	9	1472	1743	25	260	2.91E-25	104	COG0631	PTC1	Serine/threonine protein phosphatase
LN02_02675∣LN02Chr02: 2627485-2634952(-)∣2194	CDD:225025	22.311	251	132	5	1779	2025	32	223	5.44E-20	88.4	COG2114	CyaA	Adenylate cyclase, family 3 (some proteins contain HAMP domain)
LN02_02803∣LN02Chr02: 3059085-3060125(-)∣346	CDD:225035	26.518	313	169	12	8	312	35	294	1.04E-22	95.2	COG2124	CypX	Cytochrome P450
LN02_02867∣LN02Chr02: 3265423-3267648(+)∣644	CDD:224153	24.855	519	304	21	104	612	2	444	2.54E-54	189	COG1232	HemY	Protoporphyrinogen oxidase
LN02_02931∣LN02Chr02: 3499475-3501282(+)∣495	CDD:223362	28.484	488	275	16	13	491	1	423	7.26E-82	257	COG0285	FolC	Folylpolyglutamate synthase
LN02_03315∣LN02Chr02: 4845999-4847636(+)∣289	CDD:223565	26.562	256	150	5	33	263	10	252	5.66E-37	129	COG0491	GloB	Zn-dependent hydrolases, including glyoxylases
LN02_03379∣LN02Chr02: 5033488-5034409(+)∣266	CDD:227463	22.17	212	129	6	9	207	3	191	1.25E-19	82.8	COG5134	COG5134	Uncharacterized conserved protein
LN02_03507∣LN02Chr02: 5814392-5816071(-)∣559	CDD:223605	18.919	444	336	7	63	504	36	457	3.24E-34	132	COG0531	PotE	Amino acid transporters
LN02_03699∣LN02Chr03: 352415-353530(-)∣252	CDD:223477	22.794	136	77	4	85	211	49	165	0.001	36.5	COG0400	COG0400	Predicted esterase

LN02_03827|LN02Chr03: 864033-866591(-)|852	CDD:2241 55	28.239	301	185	9	554	832	1	292	3.55E-51	178	COG12 34	ElaC	Metal-dependent hydrolases of the beta-lactamase superfamily III
LN02_04147|LN02Chr03: 2823581-2824159(+)|144	CDD:2274 51	19.863	146	102	5	1	143	1	134	3.98E-05	38.3	COG51 22	TRS23	Transport protein particle (TRAPP) complex subunit
LN02_04211|LN02Chr03: 3379045-3380508(+)|444	CDD:2231 28	65.829	398	129	3	47	444	4	394	0	650	COG00 50	TufB	GTPases - translation elongation factors
LN02_04275|LN02Chr03: 3615689-3616765(-)|67	CDD:2250 78	58.824	51	21	0	17	67	1	51	7.06E-13	54	COG21 67	RPL39	Ribosomal protein L39E
LN02_04659|LN02Chr03: 4955298-4956120(+)|212	CDD:2250 90	26.446	121	71	5	52	171	28	131	2.13E-05	40.4	COG21 79	COG217 9	Predicted hydrolase of the HAD superfamily
LN02_04787|LN02Chr03: 5408605-5410888(+)|434	CDD:2235 87	37.5	400	242	5	25	420	4	399	1.96E-134	393	COG05 13	SrmB	Superfamily II DNA and RNA helicases
LN02_04979|LN02Chr04: 242391-243434(-)|347	CDD:2267 36	39.222	334	168	10	15	347	7	306	6.91E-99	292	COG42 86	COG428 6	Uncharacterized conserved protein related to MYG1 family
LN02_05043|LN02Chr04: 438446-441483(+)|892	CDD:2241 17	23.256	344	240	6	296	635	672	995	1.86E-09	58.6	COG11 96	Smc	Chromosome segregation ATPases
LN02_05107|LN02Chr04: 631522-635342(+)|1255	CDD:2274 99	21.134	194	128	5	1074	1255	26	206	7.28E-10	57.3	COG51 71	YRB1	Ran GTPase-activating protein (Ran-binding protein)
LN02_05107|LN02Chr04: 631522-635342(+)|1255	CDD:2279 38	18.859	403	288	4	674	1075	121	485	1.53E-04	42.6	COG56 51	COG565 1	PPE-repeat proteins
LN02_05235|LN02Chr04: 1051998-1053985(-)|576	CDD:2275 11	32.251	431	258	10	138	566	63	461	1.50E-81	260	COG51 84	ATS1	Alpha-tubulin suppressor and related RCC1 domain-containing proteins
LN02_05363|LN02Chr04: 1498942-1501834(+)|376	CDD:2233 27	35.06	251	142	7	73	316	521	757	3.05E-63	213	COG02 49	MutS	Mismatch repair ATPase (MutS family)
LN02_05491|LN02Chr04: 2089905-2091748(+)|493	CDD:2249 32	37.246	443	189	7	22	455	5	367	1.79E-133	388	COG20 21	MET2	Homoserine acetyltransferase
LN02_05555|LN02Chr04: 2436221-2438397(-)|569	CDD:2252 01	18.182	297	202	7	110	394	149	416	9.94E-06	45.1	COG23 19	COG231 9	FOG: WD40 repeat
LN02_05619|LN02Chr04: 2650759-2652640(-)|570	CDD:2265 82	18.991	337	212	14	125	451	68	353	1.92E-10	60.2	COG40 97	COG409 7	Predicted ferric reductase
LN02_06003|LN02Chr04: 3981165-3982981(+)|415	CDD:2274 06	33.476	233	154	1	96	328	26	257	3.23E-49	165	COG50 74	COG507 4	t-SNARE complex subunit, syntaxin
LN02_06195|LN02Chr04: 4628162-4633201(+)|1589	CDD:2240 37	29.224	438	243	7	1083	1503	367	754	5.84E-63	227	COG11 12	COG111 2	Superfamily I DNA and RNA helicases and helicase subunits
LN02_06195|LN02Chr04: 4628162-4633201(+)|1589	CDD:2243 85	18.301	153	89	7	673	808	47	180	2.67E-04	40.9	COG14 68	COG146 8	CRISPR-associated protein Cas4 (RecB family exonuclease)
LN02_06259|LN02Chr04: 4819721-4825216(-)|1763	CDD:2231 64	32.426	882	493	26	18	892	6	791	0	744	COG00 86	RpoC	DNA-directed RNA polymerase, beta' subunit/160 kD subunit
LN02_06259|LN02Chr04: 4819721-4825216(-)|1763	CDD:2231 64	23.664	393	227	15	1077	1463	413	738	9.58E-28	118	COG00 86	RpoC	DNA-directed RNA polymerase, beta' subunit/160 kD subunit
LN02_06323|LN02Chr04: 5029504-5031143(-)|518	CDD:2231 22	34.205	459	241	11	40	497	26	424	2.58E-106	321	COG00 44	PyrC	Dihydroorotase and related cyclic amidohydrolases
LN02_06387|LN02Chr04: 5228880-5230802(+)|490	CDD:2251 80	21.667	300	210	10	71	360	49	333	7.17E-11	61.1	COG22 71	UhpC	Sugar phosphate permease

LN02_06451∣LN02Chr04: 5427498-5429684(+)∣728	CDD:224117	20.904	177	127	2	128	304	715	878	8.49E-12	65.9	COG1196	Smc	Chromosome segregation ATPases
LN02_06643∣LN02Chr05: 208596-215424(+)∣1481	CDD:227721	40.426	47	24	2	707	750	79	124	3.94E-05	44.8	COG5434	PGU1	Endopygalactorunase
LN02_06835∣LN02Chr05: 986300-987930(+)∣398	CDD:225779	17.269	249	165	8	96	324	70	297	6.18E-07	47.9	COG3239	DesA	Fatty acid desaturase
LN02_06899∣LN02Chr05: 1206792-1207330(-)∣111	CDD:227521	56.18	89	37	1	23	111	1	87	4.42E-42	130	COG5194	APC11	Component of SCF ubiquitin ligase and anaphase-promoting complex
LN02_07091∣LN02Chr05: 2541759-2542874(-)∣371	CDD:223616	49.3	357	157	5	7	362	2	335	2.62E-144	425	COG0542	clpA	ATP-binding subunits of Clp protease and DnaK/DnaJ chaperones
LN02_07155∣LN02Chr05: 2752037-2752327(-)∣96	CDD:223212	60.227	88	35	0	7	94	110	197	7.71E-46	145	COG0134	TrpC	Indole-3-glycerol phosphate synthase
LN02_07283∣LN02Chr05: 3185166-3185914(-)∣214	CDD:227457	39.378	193	111	4	19	211	22	208	9.07E-54	168	COG5128	COG5128	Transport protein particle (TRAPP) complex subunit
LN02_07411∣LN02Chr05: 3715517-3716242(-)∣160	CDD:226300	23.558	208	94	8	1	157	79	272	1.62E-10	54.8	COG3777	COG3777	Uncharacterized conserved protein
LN02_07539∣LN02Chr05: 4103905-4104464(-)∣130	CDD:223470	33.333	108	53	1	31	119	1	108	7.17E-21	78.5	COG0393	COG0393	Uncharacterized conserved protein
LN02_07603∣LN02Chr05: 4436339-4439849(-)∣1091	CDD:223550	34.452	1013	529	21	28	1028	28	917	0	776	COG0474	MgtA	Cation transport ATPase
LN02_07667∣LN02Chr05: 4773558-4774617(-)∣197	CDD:227472	35.204	196	120	2	3	197	1	190	3.78E-52	163	COG5143	SNC1	Synaptobrevin/VAMP-like protein
LN02_07731∣LN02Chr05: 5030522-5032910(-)∣739	CDD:225201	16.972	436	298	11	280	700	11	397	1.21E-10	61.3	COG2319	COG2319	FOG: WD40 repeat
LN02_08243∣LN02Chr06: 1857689-1859158(+)∣436	CDD:227510	35.714	56	27	3	102	157	25	71	3.65E-06	46.5	COG5183	SSM4	Protein involved in mRNA turnover and stability
LN02_08435∣LN02Chr06: 2604441-2606702(+)∣626	CDD:227911	23.746	299	187	8	343	625	221	494	8.09E-24	102	COG5624	TAF61	Transcription initiation factor TFIID, subunit TAF12 (also component of histone acetyltransferase SAGA)
LN02_08563∣LN02Chr06: 3024539-3026432(+)∣452	CDD:227270	29	100	50	5	348	444	429	510	4.53E-05	42.8	COG4934	COG4934	Predicted protease
LN02_08883∣LN02Chr07: 685420-686600(+)∣386	CDD:225333	30.03	333	189	16	42	357	18	323	5.79E-41	145	COG2706	COG2706	3-carboxymuconate cyclase
LN02_08947∣LN02Chr07: 927537-929646(-)∣605	CDD:223087	42.378	328	173	7	85	407	10	326	1.02E-111	340	COG0008	GlnS	Glutamyl- and glutaminyl-tRNA synthetases
LN02_09203∣LN02Chr07: 1738376-1740951(+)∣733	CDD:224117	16.418	134	107	2	65	198	763	891	1.00E-04	42.8	COG1196	Smc	Chromosome segregation ATPases
LN02_09267∣LN02Chr07: 1995748-1996563(+)∣271	CDD:225064	24.545	110	56	5	154	263	71	153	1.00E-07	47.3	COG2153	ElaA	Predicted acyltransferase
LN02_00116∣LN02Chr01: 532901-534589(-)∣531	CDD:223231	29.899	495	225	18	30	516	9	389	3.64E-77	245	COG0153	GalK	Galactokinase
LN02_00244∣LN02Chr01: 1442579-1443495(-)∣208	CDD:224025	38.674	181	93	4	13	175	4	184	8.39E-46	148	COG1100	COG1100	GTPase SAR1 and related small G proteins
LN02_00436∣LN02Chr01: 2113042-2114474(+)∣377	CDD:227122	32.99	194	116	5	151	338	73	258	1.04E-30	119	COG4783	COG4783	Putative Zn-dependent protease, contains TPR repeats

LN02_00500|LN02Chr01:2301116-2301997(-)|244	CDD:224277	37.255	102	55	1	87	188	2	94	1.51E-20	81.2	COG1358	RPL8A	Ribosomal protein HS6-type (S12/L30/L7a)
LN02_00564|LN02Chr01:2487278-2488962(+)|518	CDD:224653	33.333	114	64	6	254	362	18	124	1.32E-11	60.8	COG1739	COG1739	Uncharacterized conserved protein
LN02_00628|LN02Chr01:2787224-2788755(-)|458	CDD:223234	33.793	435	222	11	6	421	1	388	2.60E-99	300	COG0156	BioF	7-keto-8-aminopelargonate synthetase and related enzymes
LN02_00756|LN02Chr01:3260784-3262227(-)|451	CDD:223997	40.659	364	205	6	69	428	2	358	1.40E-125	366	COG1071	AcoA	Pyruvate/2-oxoglutarate dehydrogenase complex, dehydrogenase (E1) component, eukaryotic type, alpha subunit
LN02_00884|LN02Chr01:3680669-3680932(-)|87	CDD:224754	50.909	55	27	0	4	58	1	55	1.68E-13	56.4	COG1841	RpmD	Ribosomal protein L30/L7E
LN02_00948|LN02Chr01:3873894-3874787(+)|209	CDD:223678	50.49	204	86	6	5	198	4	202	3.39E-82	240	COG0605	SodA	Superoxide dismutase
LN02_01012|LN02Chr01:4093525-4094299(+)|142	CDD:224869	37.838	74	43	2	1	71	6	79	3.39E-16	65.8	COG1958	LSM1	Small nuclear ribonucleoprotein (snRNP) homolog
LN02_01332|LN02Chr01:5329749-5331296(+)|447	CDD:225687	26.087	115	62	6	248	361	102	194	3.92E-10	56.3	COG3145	AlkB	Alkylated DNA repair protein
LN02_01652|LN02Chr01:6588983-6589585(-)|175	CDD:225218	22.561	164	102	10	2	157	53	199	2.08E-07	46	COG2340	COG2340	Uncharacterized protein with SCP/PR1 domains
LN02_01780|LN02Chr01:7006490-7008925(+)|811	CDD:227891	33.602	497	263	8	253	749	94	523	1.51E-110	344	COG5604	COG5604	Uncharacterized conserved protein
LN02_02036|LN02Chr02:393955-395083(+)|344	CDD:225041	41.983	343	187	6	2	343	7	338	1.15E-116	338	COG2130	COG2130	Putative NADP-dependent oxidoreductases
LN02_02228|LN02Chr02:1005114-1008071(+)|961	CDD:225201	31.132	318	189	11	416	724	144	440	1.89E-35	138	COG2319	COG2319	FOG: WD40 repeat
LN02_02228|LN02Chr02:1005114-1008071(+)|961	CDD:225201	19.646	565	322	15	91	645	8	450	1.81E-24	105	COG2319	COG2319	FOG: WD40 repeat
LN02_02228|LN02Chr02:1005114-1008071(+)|961	CDD:225201	32.653	98	62	2	42	139	348	441	6.29E-09	56.3	COG2319	COG2319	FOG: WD40 repeat
LN02_02292|LN02Chr02:1247600-1248702(+)|310	CDD:224941	28.369	141	94	5	170	304	20	159	2.50E-13	64	COG2030	MaoC	Acyl dehydratase
LN02_02484|LN02Chr02:1978166-1980574(-)|778	CDD:224441	25.532	376	250	9	248	604	41	405	1.50E-52	186	COG1524	COG1524	Uncharacterized proteins of the AP superfamily
LN02_02548|LN02Chr02:2193562-2194809(+)|325	CDD:227524	33.758	314	177	6	9	322	2	284	1.62E-81	246	COG5197	COG5197	Predicted membrane protein
LN02_02932|LN02Chr02:3501465-3503036(-)|523	CDD:223362	30.958	449	243	14	84	518	27	422	6.54E-92	284	COG0285	FolC	Folylpolyglutamate synthase
LN02_03124|LN02Chr02:4161734-4165913(-)|1305	CDD:227470	30.444	496	259	11	443	937	131	541	2.81E-105	345	COG5141	COG5141	PHD zinc finger-containing protein
LN02_03316|LN02Chr02:4848359-4849154(+)|189	CDD:227455	22.517	151	104	3	14	164	1	138	9.12E-10	52.3	COG5126	FRQ1	Ca2+-binding protein (EF-Hand superfamily)
LN02_03572|LN02Chr02:6247694-6249744(+)|608	CDD:223383	35.088	171	100	2	433	603	164	323	2.63E-33	127	COG0306	PitA	Phosphate/sulphate permeases

LN02_03572|LN02Chr02: 6247694-6249744(+)|608	CDD:2233 83	32.184	174	110	3	8	181	2	167	3.85E-28	112	COG03 06	PitA	Phosphate/sulphate permeases
LN02_03636|LN02Chr03: 75729-76712(-)|246	CDD:2243 06	26.866	67	40	1	114	171	34	100	3.17E-06	42.6	COG13 88	LytE	FOG: LysM repeat
LN02_03700|LN02Chr03: 355439-356137(+)|232	CDD:2258 58	22.277	202	118	9	34	218	475	654	1.20E-09	54.8	COG33 21	COG332 1	Polyketide synthase modules and related proteins
LN02_03828|LN02Chr03: 868287-870016(+)|503	CDD:2275 37	21.996	491	218	7	14	503	30	356	7.78E-34	128	COG52 12	PDE1	Low-affinity cAMP phosphodiesterase
LN02_04212|LN02Chr03: 3381486-3381878(-)|57	CDD:2232 77	41.176	51	25	2	7	54	10	58	1.14E-09	45.7	COG01 99	RpsN	Ribosomal protein S14
LN02_04276|LN02Chr03: 3616924-3617817(+)|180	CDD:2240 25	26.316	190	117	4	14	180	2	191	2.02E-29	105	COG11 00	COG110 0	GTPase SAR1 and related small G proteins
LN02_04532|LN02Chr03: 4463116-4467323(-)|1051	CDD:2241 17	18.466	352	247	6	311	644	691	1020	1.13E-13	72.4	COG11 96	Smc	Chromosome segregation ATPases
LN02_04596|LN02Chr03: 4734841-4736408(-)|456	CDD:2274 40	25.651	269	167	4	208	454	137	394	1.05E-47	166	COG51 09	COG510 9	Uncharacterized conserved protein, contains RING Zn-finger
LN02_04660|LN02Chr03: 4957263-4957786(+)|135	CDD:2278 83	40.876	137	74	1	1	130	52	188	1.03E-22	85.7	COG55 96	TIM22	Mitochondrial import inner membrane translocase, subunit TIM22
LN02_05044|LN02Chr04: 441658-442982(-)|237	CDD:2237 38	23.113	212	154	4	5	208	2	212	3.86E-17	74.9	COG06 66	Arp	FOG: Ankyrin repeat
LN02_05108|LN02Chr04: 636262-638091(+)|481	CDD:2273 56	48.956	431	209	6	35	464	15	435	0	602	COG50 23	COG502 3	Tubulin
LN02_05236|LN02Chr04: 1055774-1056305(+)|130	CDD:2231 23	36.17	141	65	4	6	130	256	387	3.98E-24	92.3	COG00 45	SucC	Succinyl-CoA synthetase, beta subunit
LN02_05428|LN02Chr04: 1726207-1729674(+)|1082	CDD:2274 89	26.007	273	105	5	533	805	369	544	6.77E-29	120	COG51 60	ULP1	Protease, Ulp1 family
LN02_05492|LN02Chr04: 2096351-2098876(+)|713	CDD:2253 71	28.723	94	61	2	122	215	57	144	4.16E-06	46.5	COG28 14	AraJ	Arabinose efflux permease
LN02_05556|LN02Chr04: 2439057-2441494(+)|657	CDD:2241 20	22.459	610	418	14	1	583	73	654	4.64E-105	330	COG11 99	DinG	Rad3-related DNA helicases
LN02_06132|LN02Chr04: 4376180-4377049(+)|192	CDD:2273 97	43.094	181	100	2	6	185	3	181	2.27E-62	188	COG50 65	PHO88	Protein involved in inorganic phosphate transport
LN02_06260|LN02Chr04: 4826124-4827603(-)|470	CDD:2252 01	21.345	342	220	11	24	358	155	454	4.61E-14	70.9	COG23 19	COG231 9	FOG: WD40 repeat
LN02_06324|LN02Chr04: 5031977-5033438(-)|452	CDD:2232 20	34.492	374	189	6	82	452	2	322	2.21E-94	285	COG01 42	IspA	Geranylgeranyl pyrophosphate synthase
LN02_06388|LN02Chr04: 5231430-5232680(-)|416	CDD:2237 27	24.257	404	268	11	6	395	1	380	8.26E-41	146	COG06 54	UbiH	2-polyprenyl-6-methoxyphenol hydroxylase and related FAD-dependent oxidoreductases
LN02_06452|LN02Chr04: 5430984-5433999(-)|915	CDD:2248 39	40.172	697	379	12	58	751	38	699	0	624	COG19 28	PMT1	Dolichyl-phosphate-mannose--protein O-mannosyl transferase
LN02_06516|LN02Chr04: 5708498-5709217(+)|210	CDD:2260 90	31.937	191	125	2	7	197	2	187	6.94E-49	155	COG35 60	FMR2	Predicted oxidoreductase related to nitroreductase
LN02_06708|LN02Chr05: 485368-487875(-)|790	CDD:2237 30	36.25	160	97	1	138	292	72	231	2.30E-35	133	COG06 57	Aes	Esterase/lipase

LN02_06964∣LN02Chr05: 1807209-1808458(-)∣391	CDD:225182	21.488	242	136	12	122	339	104	315	8.84E-09	53.6	COG2273	SKN1	Beta-glucanase/Beta-glucan synthetase
LN02_07348∣LN02Chr05: 3410257-3410749(+)∣113	CDD:224869	24.658	73	51	2	7	75	7	79	1.44E-13	58.1	COG1958	LSM1	Small nuclear ribonucleoprotein (snRNP) homolog
LN02_07412∣LN02Chr05: 3717729-3720152(+)∣807	CDD:224418	28.06	531	321	19	134	631	202	704	7.11E-103	332	COG1501	COG1501	Alpha-glucosidases, family 31 of glycosyl hydrolases
LN02_07540∣LN02Chr05: 4105590-4106196(-)∣131	CDD:223892	50.4	125	41	3	5	110	26	148	9.11E-39	125	COG0822	IscU	NifU homolog involved in Fe-S cluster formation
LN02_07604∣LN02Chr05: 4444541-4446573(+)∣627	CDD:226744	37.126	334	174	6	197	527	25	325	5.06E-97	298	COG4294	Uve	UV damage repair endonuclease
LN02_07668∣LN02Chr05: 4775830-4781855(+)∣1956	CDD:224125	29.951	818	512	16	242	1054	4	765	0	615	COG1204	COG1204	Superfamily II helicase
LN02_07668∣LN02Chr05: 4775830-4781855(+)∣1956	CDD:224125	31.199	609	364	18	1122	1717	24	590	7.97E-120	395	COG1204	COG1204	Superfamily II helicase
LN02_07732∣LN02Chr05: 5033873-5035366(+)∣379	CDD:223199	24.841	314	170	10	1	310	1	252	1.13E-41	144	COG0121	COG0121	Predicted glutamine amidotransferase
LN02_07796∣LN02Chr05: 5274296-5276999(+)∣814	CDD:225942	20.339	236	157	9	523	755	358	565	6.23E-10	59.8	COG3408	GDB1	Glycogen debranching enzyme
LN02_07860∣LN02Chr05: 5517293-5518354(+)∣353	CDD:224981	30.595	353	208	8	7	343	3	334	4.40E-65	206	COG2070	COG2070	Dioxygenases related to 2-nitropropane dioxygenase
LN02_08308∣LN02Chr06: 2071155-2072401(+)∣302	CDD:224517	39.735	151	85	3	146	296	7	151	1.12E-45	149	COG1601	GCD7	Translation initiation factor 2, beta subunit (eIF-2beta)/eIF-5 N-terminal domain
LN02_08628∣LN02Chr06: 3206042-3209944(+)∣1300	CDD:223951	35.828	441	242	10	8	418	213	642	4.39E-73	254	COG1020	EntF	Non-ribosomal peptide synthetase modules and related proteins
LN02_08628∣LN02Chr06: 3206042-3209944(+)∣1300	CDD:223188	18.792	149	102	5	666	799	28	172	1.78E-04	41	COG0110	WbbJ	Acetyltransferase (isoleucine patch superfamily)
LN02_08692∣LN02Chr06: 3559458-3561403(-)∣600	CDD:225043	20.613	587	306	21	2	580	9	443	1.18E-42	156	COG2132	SufI	Putative multicopper oxidases
LN02_08820∣LN02Chr07: 451175-452916(+)∣511	CDD:225181	30.29	449	239	20	73	500	88	483	1.86E-62	209	COG2272	PnbA	Carboxylesterase type B
LN02_08884∣LN02Chr07: 687962-689330(+)∣330	CDD:223729	37.864	309	152	8	6	310	5	277	2.40E-89	266	COG0656	ARA1	Aldo/keto reductases, related to diketogulonate reductase
LN02_08948∣LN02Chr07: 930410-931881(+)∣418	CDD:223560	39.017	346	188	11	11	348	5	335	1.74E-107	319	COG0484	DnaJ	DnaJ-class molecular chaperone with C-terminal Zn finger domain
LN02_09076∣LN02Chr07: 1344177-1347223(+)∣841	CDD:224157	32.985	479	268	10	25	502	1	427	1.03E-126	384	COG1236	YSH1	Predicted exonuclease of the beta-lactamase fold involved in RNA processing
LN02_09204∣LN02Chr07: 1741807-1743920(-)∣461	CDD:223513	28.732	355	221	14	115	461	61	391	5.71E-58	193	COG0436	COG0436	Aspartate/tyrosine/aromatic aminotransferase
LN02_09268∣LN02Chr07: 1997021-1997987(-)∣213	CDD:223127	42.038	157	82	3	57	213	1	148	1.06E-62	189	COG0049	RpsG	Ribosomal protein S7
LN02_00053∣LN02Chr01: 337790-339587(+)∣529	CDD:224374	26.039	457	313	10	74	529	10	442	6.88E-44	158	COG1457	CodB	Purine-cytosine permease and related proteins

LN02_00373|LN02Chr01: 1938354-1939849(+)|378	CDD:2233 01	31.268	355	176	14	37	378	1	300	1.92E-51	171	COG02 23	Fmt	Methionyl-tRNA formyltransferase
LN02_00437|LN02Chr01: 2114907-2115232(+)|84	CDD:2275 99	36.782	87	50	3	1	83	48	133	6.72E-17	67.9	COG52 74	CYB5	Cytochrome b involved in lipid metabolism
LN02_00565|LN02Chr01: 2490096-2491445(+)|326	CDD:2235 89	27.864	323	176	12	3	310	1	281	2.82E-56	184	COG05 15	SPS1	Serine/threonine protein kinase
LN02_00629|LN02Chr01: 2789745-2792586(+)|814	CDD:2232 39	23.698	557	298	16	266	811	1	441	7.75E-54	190	COG01 61	BioA	Adenosylmethionine-8-amino-7-oxononanoate aminotransferase
LN02_00629|LN02Chr01: 2789745-2792586(+)|814	CDD:2232 10	26.374	182	95	6	18	184	7	164	9.25E-17	77.3	COG01 32	BioD	Dethiobiotin synthetase
LN02_00693|LN02Chr01: 3065535-3067389(+)|568	CDD:2234 82	37.269	542	294	13	37	559	17	531	1.40E-148	435	COG04 05	Ggt	Gamma-glutamyltransferase
LN02_00757|LN02Chr01: 3263620-3264654(+)|317	CDD:2251 36	41.085	258	131	7	60	316	1	238	1.11E-94	277	COG22 26	UbiE	Methylase involved in ubiquinone/menaquinone biosynthesis
LN02_00885|LN02Chr01: 3681916-3684080(-)|660	CDD:2231 52	29.245	318	193	7	39	352	4	293	5.07E-87	271	COG00 74	SucD	Succinyl-CoA synthetase, alpha subunit
LN02_00885|LN02Chr01: 3681916-3684080(-)|660	CDD:2234 49	20.717	251	163	9	401	638	150	377	2.39E-39	146	COG03 72	GltA	Citrate synthase
LN02_01013|LN02Chr01: 4094974-4100714(-)|1859	CDD:2273 15	34.942	1036	446	17	125	1138	37	866	0	964	COG49 82	COG498 2	3-oxoacyl-[acyl-carrier protein].
LN02_01013|LN02Chr01: 4094974-4100714(-)|1859	CDD:2233 81	24.795	488	237	11	1155	1625	33	407	6.58E-75	253	COG03 04	FabB	3-oxoacyl-(acyl-carrier-protein) synthase
LN02_01013|LN02Chr01: 4094974-4100714(-)|1859	CDD:2238 07	43.333	120	63	2	1742	1857	5	123	1.79E-37	134	COG07 36	AcpS	Phosphopantetheinyl transferase (holo-ACP synthase)
LN02_01013|LN02Chr01: 4094974-4100714(-)|1859	CDD:2234 08	43.103	58	32	1	4	61	234	290	6.06E-14	71.9	COG03 31	FabD	(acyl-carrier-protein) S-malonyltransferase
LN02_01077|LN02Chr01: 4332440-4337234(+)|1532	CDD:2240 36	22.318	578	353	20	90	636	16	528	1.47E-49	182	COG11 11	MPH1	ERCC4-like helicases
LN02_01077|LN02Chr01: 4332440-4337234(+)|1532	CDD:2236 44	29.379	177	90	5	1247	1422	13	155	3.26E-29	114	COG05 71	Rnc	dsRNA-specific ribonuclease
LN02_01077|LN02Chr01: 4332440-4337234(+)|1532	CDD:2236 44	29.333	150	74	5	1051	1199	21	139	5.77E-12	64.2	COG05 71	Rnc	dsRNA-specific ribonuclease
LN02_01269|LN02Chr01: 5106771-5108532(+)|518	CDD:2261 99	26.282	312	166	9	19	318	31	290	2.18E-40	148	COG36 73	COG367 3	Uncharacterized conserved protein
LN02_01333|LN02Chr01: 5331523-5332449(+)|308	CDD:2248 96	27.917	240	144	7	65	301	3	216	3.37E-35	124	COG19 85	RibD	Pyrimidine reductase, riboflavin biosynthesis
LN02_01397|LN02Chr01: 5579293-5581316(-)|561	CDD:2253 71	22.102	371	233	9	73	442	7	322	8.90E-21	91.5	COG28 14	AraJ	Arabinose efflux permease
LN02_01525|LN02Chr01: 6108006-6110433(-)|783	CDD:2273 81	39.506	81	47	1	451	529	384	464	3.97E-04	40.4	COG50 48	COG504 8	FOG: Zn-finger
LN02_01717|LN02Chr01: 6845023-6846518(+)|421	CDD:2235 39	18.487	238	146	4	115	352	4	193	4.78E-18	81.3	COG04 63	WcaA	Glycosyltransferases involved in cell wall biogenesis

LN02_01781|LN02Chr01:7009372-7012482(-)|1008	CDD:224117	21.452	303	213	4	305	604	198	478	4.83E-15	77.1	COG1196	Smc	Chromosome segregation ATPases
LN02_01973|LN02Chr02:159956-160718(-)|204	CDD:223568	24	150	108	4	58	203	13	160	6.31E-12	58.7	COG0494	MutT	NTP pyrophosphohydrolases including oxidative damage repair enzymes
LN02_02229|LN02Chr02:1008489-1009081(+)|121	CDD:224869	31.884	69	45	1	30	96	11	79	2.07E-18	70.8	COG1958	LSM1	Small nuclear ribonucleoprotein (snRNP) homolog
LN02_02293|LN02Chr02:1249047-1250921(+)|579	CDD:227569	32.031	128	76	5	7	132	6	124	2.68E-11	63.2	COG5244	NIP100	Dynactin complex subunit involved in mitotic spindle partitioning in anaphase B
LN02_02293|LN02Chr02:1249047-1250921(+)|579	CDD:227223	19.758	248	171	12	158	399	116	341	6.20E-08	51.9	COG4886	COG4886	Leucine-rich repeat (LRR) protein
LN02_02357|LN02Chr02:1526085-1527834(+)|517	CDD:223083	42.857	420	228	4	32	450	1	409	4.52E-157	450	COG0004	AmtB	Ammonia permease
LN02_02485|LN02Chr02:1981241-1983061(-)|530	CDD:227170	21.505	93	70	2	168	260	136	225	2.12E-05	43.8	COG4833	COG4833	Predicted glycosyl hydrolase
LN02_02613|LN02Chr02:2417485-2418537(-)|317	CDD:227437	40.575	313	180	3	1	312	1	308	1.07E-102	301	COG5106	RPF2	Uncharacterized conserved protein
LN02_02805|LN02Chr02:3064947-3067173(-)|594	CDD:227568	31.579	76	47	2	331	402	275	349	9.13E-10	58.1	COG5243	HRD1	HRD ubiquitin ligase complex, ER membrane component
LN02_02869|LN02Chr02:3273240-3275058(-)|496	CDD:223441	44.191	482	245	9	1	469	2	472	0	626	COG0364	Zwf	Glucose-6-phosphate 1-dehydrogenase
LN02_02933|LN02Chr02:3503695-3505911(-)|664	CDD:226676	28.019	207	131	6	453	656	228	419	4.00E-12	65.8	COG4223	COG4223	Uncharacterized protein conserved in bacteria
LN02_02933|LN02Chr02:3503695-3505911(-)|664	CDD:224495	23.649	148	100	6	334	473	32	174	8.36E-05	41.6	COG1579	COG1579	Zn-ribbon protein, possibly nucleic acid-binding
LN02_03061|LN02Chr02:3947277-3949642(-)|700	CDD:227381	27.083	144	96	3	557	693	35	176	1.03E-06	48.5	COG5048	COG5048	FOG: Zn-finger
LN02_03125|LN02Chr02:4168622-4171250(-)|853	CDD:227223	32.836	134	86	4	429	560	101	232	5.05E-13	68.8	COG4886	COG4886	Leucine-rich repeat (LRR) protein
LN02_03189|LN02Chr02:4430789-4432530(-)|551	CDD:225201	29.018	224	147	5	280	502	154	366	7.72E-27	110	COG2319	COG2319	FOG: WD40 repeat
LN02_03317|LN02Chr02:4849690-4850684(-)|261	CDD:227386	24.02	204	137	7	35	234	28	217	1.48E-19	81.5	COG5053	CDC33	Translation initiation factor 4E (eIF-4E)
LN02_03957|LN02Chr03:1244229-1244757(-)|153	CDD:224745	27.885	104	72	2	17	120	20	120	2.11E-18	73.5	COG1832	COG1832	Predicted CoA-binding protein
LN02_04213|LN02Chr03:3382464-3383670(+)|372	CDD:225660	41.111	90	47	3	20	106	23	109	4.04E-15	72.4	COG3118	COG3118	Thioredoxin domain-containing protein
LN02_04213|LN02Chr03:3382464-3383670(+)|372	CDD:225660	23.226	155	101	6	123	270	1	144	1.19E-05	43.5	COG3118	COG3118	Thioredoxin domain-containing protein
LN02_04533|LN02Chr03:4468758-4469473(-)|160	CDD:225050	38.614	101	58	1	3	103	2	98	4.94E-32	107	COG2139	RPL21A	Ribosomal protein L21E
LN02_04597|LN02Chr03:4737440-4740190(+)|888	CDD:224117	25.688	109	79	1	760	866	235	343	2.19E-04	42	COG1196	Smc	Chromosome segregation ATPases
LN02_04853|LN02Chr03:5782710-5785179(+)|732	CDD:223400	29.368	538	351	14	28	562	3	514	2.73E-135	410	COG0323	MutL	DNA mismatch repair enzyme (predicted ATPase)

LN02_04917|LN02Chr03: 5954913-5958107(-)|535	CDD:224964	64.407	59	19	1	479	535	9	67	3.97E-24	92.9	COG2053	RPS28A	Ribosomal protein S28E/S33
LN02_05045|LN02Chr04: 443247-444974(+)|497	CDD:225201	32.268	313	193	9	174	476	139	442	8.70E-48	169	COG2319	COG2319	FOG: WD40 repeat
LN02_05237|LN02Chr04: 1056731-1057538(+)|217	CDD:223188	35	180	108	5	34	209	2	176	4.42E-37	125	COG0110	WbbJ	Acetyltransferase (isoleucine patch superfamily)
LN02_05429|LN02Chr04: 1730404-1732227(-)|607	CDD:225201	22.727	264	172	6	247	493	194	442	7.97E-22	95.9	COG2319	COG2319	FOG: WD40 repeat
LN02_05621|LN02Chr04: 2655141-2656235(-)|147	CDD:227410	56.757	148	63	1	1	147	5	152	5.42E-81	232	COG5078	COG5078	Ubiquitin-protein ligase
LN02_05877|LN02Chr04: 3488237-3489277(+)|346	CDD:223985	46.154	26	14	0	48	73	2	27	4.10E-04	38	COG1057	NadD	Nicotinic acid mononucleotide adenylyltransferase
LN02_06005|LN02Chr04: 3991293-3992365(-)|309	CDD:224327	29.412	85	55	3	25	104	29	113	3.30E-07	48	COG1409	Icc	Predicted phosphohydrolases
LN02_06133|LN02Chr04: 4377818-4379185(+)|384	CDD:227474	38.235	272	129	8	116	384	57	292	2.25E-67	212	COG5145	RAD14	DNA excision repair protein
LN02_06261|LN02Chr04: 4828068-4829487(+)|291	CDD:226508	38.628	277	141	6	2	277	1	249	1.74E-83	248	COG4021	COG4021	Uncharacterized conserved protein
LN02_06645|LN02Chr05: 221798-223865(+)|579	CDD:223796	29.63	81	53	2	146	224	115	193	4.00E-08	52.3	COG0724	COG0724	RNA-binding proteins (RRM domain)
LN02_06773|LN02Chr05: 761106-762467(+)|314	CDD:225201	21.835	316	202	10	26	311	69	369	8.69E-30	115	COG2319	COG2319	FOG: WD40 repeat
LN02_06901|LN02Chr05: 1211129-1212052(-)|255	CDD:224154	18.352	267	181	15	2	242	17	272	1.75E-07	48.6	COG1233	COG1233	Phytoene dehydrogenase and related proteins
LN02_06965|LN02Chr05: 1810227-1812939(-)|809	CDD:227381	40.58	69	39	1	15	81	19	87	3.53E-06	47.4	COG5048	COG5048	FOG: Zn-finger
LN02_07285|LN02Chr05: 3188963-3192356(-)|969	CDD:227698	33.762	311	162	10	610	914	61	333	2.46E-56	199	COG5411	COG5411	Phosphatidylinositol 5-phosphate phosphatase
LN02_07285|LN02Chr05: 3188963-3192356(-)|969	CDD:225201	17.819	376	265	14	254	615	73	418	3.50E-05	44.3	COG2319	COG2319	FOG: WD40 repeat
LN02_07669|LN02Chr05: 4782701-4783763(-)|309	CDD:224025	37.762	143	77	4	5	138	5	144	4.56E-32	116	COG1100	COG1100	GTPase SAR1 and related small G proteins
LN02_07861|LN02Chr05: 5519029-5521086(+)|587	CDD:223587	33.638	437	269	10	113	545	31	450	2.13E-125	376	COG0513	SrmB	Superfamily II DNA and RNA helicases
LN02_08117|LN02Chr06: 1308731-1311162(+)|693	CDD:223953	30.49	633	399	10	68	688	2	605	3.17E-167	490	COG1022	FAA1	Long-chain acyl-CoA synthetases (AMP-forming)
LN02_08437|LN02Chr06: 2609708-2611755(+)|581	CDD:227887	23.37	184	121	6	292	462	186	362	3.60E-05	43.2	COG5600	COG5600	Transcription-associated recombination protein
LN02_08693|LN02Chr06: 3563781-3564422(-)|171	CDD:223483	26.744	86	48	5	16	93	1	79	3.79E-04	36.7	COG0406	phoE	Broad specificity phosphatase PhoE and related phosphatases
LN02_08757|LN02Chr07: 262569-263323(+)|217	CDD:227311	29.469	207	121	7	1	197	1	192	1.66E-29	109	COG4977	COG4977	Transcriptional regulator containing an amidase domain and an AraC-type DNA-binding HTH domain
LN02_08821|LN02Chr07: 453188-454843(-)|420	CDD:225139	36.585	287	143	6	134	402	9	274	5.05E-82	251	COG2230	Cfa	Cyclopropane fatty acid synthase and related methyltransferases

LN02_08885 | LN02Chr07: 689704-693309(-) | 1178	CDD:2236 27	25.036	691	310	14	511	1178	359	864	2.29E-58	214	COG05 53	HepA	Superfamily II DNA/RNA helicases, SNF2 family
LN02_09013 | LN02Chr07: 1133127-1137914(+) | 1514	CDD:2267 02	27.251	844	482	28	282	1101	12	747	3.28E-101	339	COG42 51	COG425 1	Bacteriophytochrome (light-regulated signal transduction histidine kinase)
LN02_09013 | LN02Chr07: 1133127-1137914(+) | 1514	CDD:2238 55	26.923	130	80	1	1320	1449	1	115	3.07E-22	91.1	COG07 84	CheY	FOG: CheY-like receiver
LN02_09077 | LN02Chr07: 1347546-1350466(-) | 862	CDD:2241 88	21.612	819	467	18	33	848	6	652	2.00E-109	347	COG12 69	NtpI	Archaeal/vacuolar-type H+-ATPase subunit I
LN02_09269 | LN02Chr07: 1998343-1998880(+) | 85	CDD:2272 38	45	80	44	0	2	81	25	104	5.02E-23	81.9	COG49 01	COG490 1	Ribosomal protein S25
LN02_00054 | LN02Chr01: 340688-342055(+) | 455	CDD:2234 79	31.279	438	265	13	20	453	2	407	3.31E-72	231	COG04 02	SsnA	Cytosine deaminase and related metal-dependent hydrolases
LN02_00182 | LN02Chr01: 860344-861788(+) | 455	CDD:2247 55	22.222	180	116	4	244	399	46	225	3.90E-04	38.8	COG18 42	PspA	Phage shock protein A (IM30), suppresses sigma54-dependent transcription
LN02_00246 | LN02Chr01: 1445728-1447844(-) | 686	CDD:2241 72	27.599	529	250	17	159	681	2	403	1.57E-93	293	COG12 52	Ndh	NADH dehydrogenase, FAD-containing subunit
LN02_00438 | LN02Chr01: 2116189-2117991(+) | 520	CDD:2231 54	23.529	442	270	15	76	513	61	438	2.93E-39	145	COG00 76	GadB	Glutamate decarboxylase and related PLP-dependent proteins
LN02_00502 | LN02Chr01: 2303951-2304901(+) | 280	CDD:2234 07	27.2	250	165	6	20	262	12	251	1.55E-32	118	COG03 30	HflC	Membrane protease subunits, stomatin/prohibitin homologs
LN02_00630 | LN02Chr01: 2793057-2795599(-) | 701	CDD:2243 27	14.706	272	185	10	227	486	36	272	8.38E-06	45.3	COG14 09	Icc	Predicted phosphohydrolases
LN02_00694 | LN02Chr01: 3067940-3069598(+) | 552	CDD:2253 71	24.074	162	123	0	110	271	17	178	2.53E-08	53	COG28 14	AraJ	Arabinose efflux permease
LN02_01014 | LN02Chr01: 4104682-4110987(+) | 2101	CDD:2273 14	37.778	810	398	18	573	1374	6	717	0	830	COG49 81	COG498 1	Enoyl reductase domain of yeast-type FAS1
LN02_01014 | LN02Chr01: 4104682-4110987(+) | 2101	CDD:2234 08	25.781	384	159	11	1689	2063	1	267	2.97E-55	192	COG03 31	FabD	(acyl-carrier-protein) S-malonyltransferase
LN02_01014 | LN02Chr01: 4104682-4110987(+) | 2101	CDD:2234 08	21.658	374	206	17	172	540	7	298	2.95E-35	135	COG03 31	FabD	(acyl-carrier-protein) S-malonyltransferase
LN02_01014 | LN02Chr01: 4104682-4110987(+) | 2101	CDD:2249 41	26.016	123	76	4	1575	1687	35	152	6.90E-14	68.6	COG20 30	MaoC	Acyl dehydratase
LN02_01206 | LN02Chr01: 4884325-4887152(-) | 882	CDD:2235 87	34.545	440	282	4	86	519	31	470	1.80E-122	377	COG05 13	SrmB	Superfamily II DNA and RNA helicases
LN02_01334 | LN02Chr01: 5332879-5333832(+) | 317	CDD:2235 98	36.308	325	173	9	4	316	1	303	1.35E-61	195	COG05 24	RbsK	Sugar kinases, ribokinase family
LN02_01398 | LN02Chr01: 5583486-5585076(-) | 486	CDD:2230 95	32.534	292	120	11	83	364	111	335	2.10E-59	195	COG00 16	PheS	Phenylalanyl-tRNA synthetase alpha subunit
LN02_01398 | LN02Chr01: 5583486-5585076(-) | 486	CDD:2231 50	25.893	112	69	2	376	486	552	650	5.30E-21	93.1	COG00 72	PheT	Phenylalanyl-tRNA synthetase beta subunit
LN02_01526 | LN02Chr01: 6112019-6114205(+) | 670	CDD:2254 57	24.342	152	110	4	55	203	114	263	6.48E-17	81.7	COG29 05	COG290 5	Predicted signal-transduction protein containing cAMP-binding and CBS domains

LN02_01526 l LN02Chr01: 6112019-6114205(+) l 670	CDD:2254 57	32.773	119	62	6	266	374	158	268	2.71E-06	47.4	COG29 05	COG290 5	Predicted signal-transduction protein containing cAMP-binding and CBS domains
LN02_01526 l LN02Chr01: 6112019-6114205(+) l 670	CDD:2235 91	24.684	158	75	5	155	309	1	117	1.55E-08	50.5	COG05 17	COG051 7	FOG: CBS domain
LN02_01718 l LN02Chr01: 6847105-6848643(-) l 512	CDD:2237 27	23.41	393	260	13	46	434	15	370	6.84E-32	123	COG06 54	UbiH	2-polyprenyl-6-methoxyphenol hydroxylase and related FAD-dependent oxidoreductases
LN02_02166 l LN02Chr02: 797622-799359(-) l 545	CDD:2278 63	32.051	78	51	1	63	140	57	132	1.98E-13	65.2	COG55 76	COG557 6	Homeodomain-containing transcription factor
LN02_02230 l LN02Chr02: 1009392-1011923(-) l 843	CDD:2241 57	20.219	183	98	8	399	558	50	207	1.68E-08	54.7	COG12 36	YSH1	Predicted exonuclease of the beta-lactamase fold involved in RNA processing
LN02_02294 l LN02Chr02: 1252606-1254735(-) l 689	CDD:2252 01	21.902	347	208	14	310	656	158	441	5.79E-20	90.5	COG23 19	COG231 9	FOG: WD40 repeat
LN02_02422 l LN02Chr02: 1772306-1773577(+) l 378	CDD:2233 98	32.159	227	100	9	110	334	17	191	2.75E-34	123	COG03 21	LipB	Lipoate-protein ligase B
LN02_02486 l LN02Chr02: 1984367-1984947(+) l 106	CDD:2274 72	40	60	36	0	26	85	103	162	2.51E-26	93.6	COG51 43	SNC1	Synaptobrevin/VAMP-like protein
LN02_02614 l LN02Chr02: 2419143-2421214(+) l 656	CDD:2235 33	22.344	273	196	5	315	583	7	267	6.61E-12	63.7	COG04 57	NrfG	FOG: TPR repeat
LN02_02870 l LN02Chr02: 3276916-3277742(+) l 109	CDD:2252 95	56.731	104	38	1	6	109	4	100	5.04E-43	133	COG24 51	COG245 1	Ribosomal protein L35AE/L33A
LN02_02934 l LN02Chr02: 3506992-3507979(-) l 220	CDD:2240 25	38.122	181	97	5	19	185	5	184	4.37E-44	144	COG11 00	COG110 0	GTPase SAR1 and related small G proteins
LN02_02998 l LN02Chr02: 3710667-3714933(-) l 1344	CDD:2240 55	29.152	566	373	11	766	1321	18	565	5.20E-101	331	COG11 32	MdlB	ABC-type multidrug transport system, ATPase and permease components
LN02_02998 l LN02Chr02: 3710667-3714933(-) l 1344	CDD:2240 55	21.401	514	324	10	213	703	109	565	3.59E-49	181	COG11 32	MdlB	ABC-type multidrug transport system, ATPase and permease components
LN02_03062 l LN02Chr02: 3958719-3961298(-) l 623	CDD:2252 01	21.104	308	213	7	27	324	93	380	4.15E-26	109	COG23 19	COG231 9	FOG: WD40 repeat
LN02_03190 l LN02Chr02: 4432915-4435857(+) l 980	CDD:2245 57	40.197	709	366	15	273	928	37	740	0	738	COG16 43	HrpA	HrpA-like helicases
LN02_03446 l LN02Chr02: 5388798-5393927(+) l 1584	CDD:2273 55	35.507	1518	842	30	4	1500	5	1406	0	1229	COG50 22	COG502 2	Myosin heavy chain
LN02_03638 l LN02Chr03: 79804-83337(+) l 1090	CDD:2258 62	27.098	417	225	14	71	424	37	437	1.95E-54	193	COG33 25	ChiA	Chitinase
LN02_03958 l LN02Chr03: 1245331-1246360(+) l 303	CDD:2251 36	34.653	101	59	4	41	135	54	153	2.47E-16	73.8	COG22 26	UbiE	Methylase involved in ubiquinone/menaquinone biosynthesis
LN02_04214 l LN02Chr03: 3385309-3387882(+) l 857	CDD:2278 27	13.768	276	167	9	595	856	155	373	2.03E-15	75.8	COG55 40	COG554 0	RING-finger-containing ubiquitin ligase
LN02_04342 l LN02Chr03: 3865006-3865536(+) l 176	CDD:2274 59	46.154	143	77	0	34	176	27	169	1.79E-48	152	COG51 30	YIP3	Prenylated rab acceptor 1 and related proteins
LN02_04470 l LN02Chr03: 4258754-4260322(-) l 522	CDD:2237 96	29.134	127	83	3	319	441	105	228	2.14E-07	49.6	COG07 24	COG072 4	RNA-binding proteins (RRM domain)

LN02_04534|LN02Chr03: 4469864-4470618(+)|191	CDD:223596	27.219	169	108	4	3	170	5	159	3.57E-35	120	COG0522	RpsD	Ribosomal protein S4 and related proteins
LN02_04726|LN02Chr03: 5194550-5195401(-)|283	CDD:224117	24.837	153	109	1	105	257	717	863	1.22E-06	46.6	COG1196	Smc	Chromosome segregation ATPases
LN02_04790|LN02Chr03: 5419499-5421414(+)|546	CDD:223671	18.182	198	125	7	289	481	155	320	4.17E-11	61.2	COG0598	CorA	Mg2+ and Co2+ transporters
LN02_05046|LN02Chr04: 445173-447701(-)|727	CDD:224391	30.675	326	197	6	318	642	13	310	1.23E-57	197	COG1474	CDC6	Cdc6-related protein, AAA superfamily ATPase
LN02_05174|LN02Chr04: 860136-861601(-)|422	CDD:223720	24.051	316	161	13	67	379	10	249	3.29E-32	120	COG0647	NagD	Predicted sugar phosphatases of the HAD superfamily
LN02_05238|LN02Chr04: 1058230-1062949(-)|1520	CDD:224054	33.649	211	130	5	898	1105	18	221	3.98E-48	171	COG1131	CcmA	ABC-type multidrug transport system, ATPase component
LN02_05238|LN02Chr04: 1058230-1062949(-)|1520	CDD:224054	22.997	287	204	6	194	478	15	286	2.17E-39	145	COG1131	CcmA	ABC-type multidrug transport system, ATPase component
LN02_05366|LN02Chr04: 1507034-1508913(+)|479	CDD:227498	60.042	478	169	6	5	479	2	460	0	624	COG5170	CDC55	Serine/threonine protein phosphatase 2A, regulatory subunit
LN02_05430|LN02Chr04: 1735655-1736767(+)|237	CDD:223711	28.866	194	136	1	39	232	34	225	9.69E-45	147	COG0638	PRE1	20S proteasome, alpha and beta subunits
LN02_05494|LN02Chr04: 2103128-2104106(-)|267	CDD:224808	30	190	124	2	52	238	6	189	4.46E-39	132	COG1896	COG1896	Predicted hydrolases of HD superfamily
LN02_05622|LN02Chr04: 2657861-2659348(-)|427	CDD:224871	34.86	393	239	9	46	426	5	392	2.55E-92	281	COG1960	CaiA	Acyl-CoA dehydrogenases
LN02_05750|LN02Chr04: 3081947-3083782(+)|565	CDD:223354	17.304	497	335	16	88	551	6	459	3.30E-11	62.6	COG0277	GlcD	FAD/FMN-containing dehydrogenases
LN02_05814|LN02Chr04: 3290039-3291565(+)|508	CDD:223544	30.729	192	107	4	120	311	20	185	3.57E-31	118	COG0468	RecA	RecA/RadA recombinase
LN02_06134|LN02Chr04: 4380128-4382815(+)|838	CDD:225201	22.811	434	247	11	406	798	61	447	4.18E-21	94.4	COG2319	COG2319	FOG: WD40 repeat
LN02_06134|LN02Chr04: 4380128-4382815(+)|838	CDD:224117	19.101	89	72	0	41	129	381	469	6.74E-04	40.5	COG1196	Smc	Chromosome segregation ATPases
LN02_06198|LN02Chr04: 4638601-4639933(-)|410	CDD:223193	33.019	318	174	9	87	402	4	284	3.18E-75	233	COG0115	IlvE	Branched-chain amino acid aminotransferase/4-amino-4-deoxychorismate lyase
LN02_06390|LN02Chr04: 5236824-5238127(+)|384	CDD:223101	49.693	326	158	3	59	383	3	323	5.08E-172	480	COG0022	AcoB	Pyruvate/2-oxoglutarate dehydrogenase complex, dehydrogenase (E1) component, eukaryotic type, beta subunit
LN02_06454|LN02Chr04: 5439159-5440268(+)|229	CDD:227680	31.469	143	69	4	109	222	121	263	2.25E-23	94.9	COG5391	COG5391	Phox homology (PX) domain protein
LN02_06582|LN02Chr04: 5918825-5919916(-)|363	CDD:225136	34.545	55	31	3	93	145	40	91	4.28E-06	44.6	COG2226	UbiE	Methylase involved in ubiquinone/menaquinone biosynthesis
LN02_06582|LN02Chr04: 5918825-5919916(-)|363	CDD:223574	20.497	161	117	5	79	239	21	170	1.61E-04	39.9	COG0500	SmtA	SAM-dependent methyltransferases
LN02_06646|LN02Chr05: 226255-227431(+)|321	CDD:225483	32.53	83	56	0	233	315	141	223	4.78E-05	41.9	COG2931	COG2931	RTX toxins and related Ca2+-binding proteins

LN02_06774|LN02Chr05: 763339-767767(-)|903	CDD:225044	22.857	455	248	19	6	424	1	388	8.29E-22	95.9	COG2133	COG2133	Glucose/sorbosone dehydrogenases
LN02_07094|LN02Chr05: 2553444-2555971(+)|736	CDD:223589	32.663	199	116	6	543	736	1	186	1.42E-33	130	COG0515	SPS1	Serine/threonine protein kinase
LN02_07222|LN02Chr05: 3022312-3023204(-)|183	CDD:224020	24.339	189	121	3	1	180	1	176	9.03E-29	102	COG1095	RPB7	DNA-directed RNA polymerase, subunit E'
LN02_07350|LN02Chr05: 3413713-3414736(+)|320	CDD:226000	25.758	132	72	3	47	156	53	180	2.19E-04	39.5	COG3469	COG3469	Chitinase
LN02_07478|LN02Chr05: 3905089-3906054(-)|300	CDD:223159	29.048	210	134	9	80	280	22	225	5.78E-16	72.6	COG0081	RplA	Ribosomal protein L1
LN02_07542|LN02Chr05: 4107786-4110750(+)|917	CDD:224423	21.277	564	370	19	345	892	108	613	1.40E-53	194	COG1506	DAP2	Dipeptidyl aminopeptidases/acylaminoacyl-peptidases
LN02_07798|LN02Chr05: 5279471-5281488(-)|588	CDD:223561	32.563	519	281	14	70	588	5	454	5.05E-135	398	COG0486	ThdF	Predicted GTPase
LN02_07862|LN02Chr05: 5521857-5526798(-)|1456	CDD:224557	34.765	722	349	17	382	1085	28	645	3.73E-179	552	COG1643	HrpA	HrpA-like helicases
LN02_07926|LN02Chr05: 5706862-5708352(+)|470	CDD:223202	33.619	467	260	18	8	466	4	428	3.03E-129	378	COG0124	HisS	Histidyl-tRNA synthetase
LN02_08118|LN02Chr06: 1312246-1315392(-)|1018	CDD:227820	23.41	393	241	12	635	1017	73	415	2.62E-30	122	COG5533	UBP5	Ubiquitin C-terminal hydrolase
LN02_08118|LN02Chr06: 1312246-1315392(-)|1018	CDD:223680	20.896	134	68	7	353	486	6	101	1.01E-04	39.9	COG0607	PspE	Rhodanese-related sulfurtransferase
LN02_08246|LN02Chr06: 1863592-1866417(-)|874	CDD:223533	21.304	230	151	4	645	874	57	256	1.52E-04	41.8	COG0457	NrfG	FOG: TPR repeat
LN02_08246|LN02Chr06: 1863592-1866417(-)|874	CDD:223082	50	40	20	0	282	321	9	48	4.73E-04	40	COG0003	ArsA	Predicted ATPase involved in chromosome partitioning
LN02_08310|LN02Chr06: 2078640-2080601(+)|653	CDD:224402	24.131	489	220	12	5	490	3	343	1.81E-45	162	COG1485	COG1485	Predicted ATPase
LN02_08438|LN02Chr06: 2614071-2616245(+)|672	CDD:227360	43.046	151	73	2	335	485	195	332	1.91E-52	183	COG5027	SAS2	Histone acetyltransferase (MYST family)
LN02_08758|LN02Chr07: 264797-266449(+)|550	CDD:225371	20.94	234	159	8	231	452	139	358	4.49E-06	46.1	COG2814	AraJ	Arabinose efflux permease
LN02_08886|LN02Chr07: 693743-694920(+)|363	CDD:224478	22.519	262	170	6	29	286	5	237	4.40E-06	44.7	COG1562	ERG9	Phytoene/squalene synthetase
LN02_09014|LN02Chr07: 1139522-1144477(-)|1651	CDD:224054	37.849	251	147	6	1267	1510	5	253	1.34E-66	224	COG1131	CcmA	ABC-type multidrug transport system, ATPase component
LN02_09014|LN02Chr07: 1139522-1144477(-)|1651	CDD:224054	32.653	294	173	5	489	768	11	293	6.04E-66	222	COG1131	CcmA	ABC-type multidrug transport system, ATPase component
LN02_09142|LN02Chr07: 1554224-1557929(-)|1192	CDD:227354	33.787	441	252	9	762	1191	460	871	1.62E-99	334	COG5021	HUL4	Ubiquitin-protein ligase
LN02_00055|LN02Chr01: 345515-346462(-)|252	CDD:223377	20	140	91	6	1	129	6	135	2.80E-06	44.2	COG0300	DltE	Short-chain dehydrogenases of various substrate specificities
LN02_00183|LN02Chr01: 862154-863476(-)|423	CDD:227719	25.594	379	223	8	5	359	4	347	9.52E-55	183	COG5432	RAD18	RING-finger-containing E3 ubiquitin ligase

LN02_00247∣LN02Chr01: 1449581-1451442(-)∣452	CDD:223645	41.346	208	116	2	25	230	10	213	1.32E-72	225	COG0572	Udk	Uridine kinase
LN02_00247∣LN02Chr01: 1449581-1451442(-)∣452	CDD:223113	30.27	185	125	4	253	436	4	185	5.88E-36	129	COG0035	Upp	Uracil phosphoribosyltransferase
LN02_00311∣LN02Chr01: 1703915-1706371(-)∣690	CDD:226406	21.176	85	66	1	241	324	746	830	2.24E-04	41.4	COG3889	COG3889	Predicted solute binding protein
LN02_00567∣LN02Chr01: 2501552-2504980(-)∣1142	CDD:225127	32.123	909	400	20	205	1111	1	694	0	561	COG2217	ZntA	Cation transport ATPase
LN02_00567∣LN02Chr01: 2501552-2504980(-)∣1142	CDD:225328	23.529	68	49	2	9	76	3	67	2.51E-04	37.7	COG2608	CopZ	Copper chaperone
LN02_00695∣LN02Chr01: 3070777-3074983(+)∣1313	CDD:223550	19.825	1145	638	28	140	1248	15	915	6.19E-138	442	COG0474	MgtA	Cation transport ATPase
LN02_00887∣LN02Chr01: 3687289-3689010(+)∣489	CDD:223123	24.634	410	239	15	70	473	39	384	1.47E-67	219	COG0045	SucC	Succinyl-CoA synthetase, beta subunit
LN02_01015∣LN02Chr01: 4112396-4113986(+)∣482	CDD:223479	25.855	468	284	9	12	478	2	407	4.26E-61	203	COG0402	SsnA	Cytosine deaminase and related metal-dependent hydrolases
LN02_01079∣LN02Chr01: 4342317-4345816(-)∣992	CDD:223951	27.312	465	294	10	1	439	196	642	1.43E-73	252	COG1020	EntF	Non-ribosomal peptide synthetase modules and related proteins
LN02_01271∣LN02Chr01: 5111627-5112881(-)∣285	CDD:223985	27.027	222	137	6	36	257	1	197	9.62E-45	147	COG1057	NadD	Nicotinic acid mononucleotide adenylyltransferase
LN02_01527∣LN02Chr01: 6114607-6116192(+)∣485	CDD:223130	30.837	227	138	2	145	352	1	227	2.31E-54	180	COG0052	RpsB	Ribosomal protein S2
LN02_01847∣LN02Chr01: 7206113-7207755(+)∣463	CDD:224478	21.07	299	210	7	33	329	5	279	2.52E-48	164	COG1562	ERG9	Phytoene/squalene synthetase
LN02_02423∣LN02Chr02: 1774946-1777166(+)∣683	CDD:223664	23.871	465	329	8	16	474	1	446	3.07E-56	196	COG0591	PutP	Na+/proline symporter
LN02_02487∣LN02Chr02: 1986876-1988525(+)∣549	CDD:223622	36.364	264	156	6	94	345	2	265	6.32E-71	225	COG0548	ArgB	Acetylglutamate kinase
LN02_02679∣LN02Chr02: 2647040-2648086(+)∣286	CDD:223257	46.491	228	108	4	56	281	51	266	4.09E-90	266	COG0179	MhpD	2-keto-4-pentenoate hydratase/2-oxohepta-3-ene-1,7-dioic acid hydratase (catechol pathway)
LN02_02935∣LN02Chr02: 3509671-3511227(-)∣394	CDD:223589	25.895	363	222	8	35	377	2	337	6.36E-53	178	COG0515	SPS1	Serine/threonine protein kinase
LN02_02999∣LN02Chr02: 3718237-3719026(+)∣208	CDD:224021	29.703	202	113	7	9	207	7	182	4.78E-29	104	COG1096	COG1096	Predicted RNA-binding protein (consists of S1 domain and a Zn-ribbon domain)
LN02_03127∣LN02Chr02: 4181631-4183187(+)∣441	CDD:223582	32.673	202	98	4	33	234	1	164	4.35E-30	117	COG0508	AceF	Pyruvate/2-oxoglutarate dehydrogenase complex, dihydrolipoamide acyltransferase (E2) component, and related enzymes
LN02_03191∣LN02Chr02: 4437701-4440238(-)∣589	CDD:227438	17.222	180	124	5	23	188	34	202	4.33E-08	53.1	COG5107	RNA14	Pre-mRNA 3'-end processing (cleavage and polyadenylation) factor
LN02_03319∣LN02Chr02: 4854237-4856138(-)∣599	CDD:227392	36.232	414	232	8	95	506	49	432	1.22E-91	290	COG5059	KIP1	Kinesin-like protein
LN02_03383∣LN02Chr02: 5045119-5048065(+)∣783	CDD:224389	28.175	252	156	7	83	316	67	311	9.80E-37	139	COG1472	BglX	Beta-glucosidase-related glycosidases

LN02_03447 | LN02Chr02: 5394598-5395325(+) | 177	CDD:2248 47	37.195	164	100	2	5	168	1	161	4.76E-56	172	COG19 36	COG193 6	Predicted nucleotide kinase (related to CMP and AMP kinases)
LN02_03639 | LN02Chr03: 88716-90272(+) | 471	CDD:2233 54	18.062	454	316	15	48	471	32	459	1.44E-27	111	COG02 77	GlcD	FAD/FMN-containing dehydrogenases
LN02_03959 | LN02Chr03: 1247454-1248117(+) | 147	CDD:2274 55	33.775	151	94	3	1	147	9	157	6.69E-34	113	COG51 26	FRQ1	Ca2+-binding protein (EF-Hand superfamily)
LN02_04151 | LN02Chr03: 2846801-2849080(-) | 759	CDD:2273 58	48.913	92	47	0	194	285	81	172	1.77E-22	99.5	COG50 25	COG502 5	Transcription factor of the Forkhead/HNF3 family
LN02_04279 | LN02Chr03: 3628071-3630640(-) | 722	CDD:2251 86	22.547	581	320	22	204	714	15	535	2.33E-25	107	COG23 03	BetA	Choline dehydrogenase and related flavoproteins
LN02_04471 | LN02Chr03: 4266806-4267873(+) | 355	CDD:2239 59	25.746	268	163	10	29	284	3	246	1.17E-29	111	COG10 28	FabG	Dehydrogenases with different specificities (related to short-chain alcohol dehydrogenases)
LN02_04983 | LN02Chr04: 271206-274661(+) | 1061	CDD:2242 08	17.01	388	249	13	650	1003	2	350	6.19E-04	40.5	COG12 89	COG128 9	Predicted membrane protein
LN02_05047 | LN02Chr04: 448282-448698(+) | 138	CDD:2259 49	27.586	116	67	4	4	118	22	121	6.16E-04	34.7	COG34 15	COG341 5	Transposase and inactivated derivatives
LN02_05303 | LN02Chr04: 1298463-1299524(+) | 313	CDD:2240 01	23.607	305	167	13	49	310	55	336	6.85E-11	59.4	COG10 75	LipA	Predicted acetyltransferases and hydrolases with the alpha/beta hydrolase fold
LN02_05367 | LN02Chr04: 1509951-1511804(+) | 511	CDD:2278 18	22.989	174	110	6	232	397	60	217	1.90E-16	75.9	COG55 31	COG553 1	SWIB-domain-containing proteins implicated in chromatin remodeling
LN02_05431 | LN02Chr04: 1738334-1740480(+) | 675	CDD:2235 87	40.662	423	224	7	160	574	5	408	1.98E-152	448	COG05 13	SrmB	Superfamily II DNA and RNA helicases
LN02_05559 | LN02Chr04: 2452538-2455849(-) | 1103	CDD:2274 89	22.581	217	133	9	880	1086	370	561	5.74E-19	89.2	COG51 60	ULP1	Protease, Ulp1 family
LN02_05623 | LN02Chr04: 2663988-2667096(+) | 962	CDD:2275 78	39.806	309	162	6	303	605	244	534	5.82E-105	336	COG52 53	MSS4	Phosphatidylinositol-4-phosphate 5-kinase
LN02_05687 | LN02Chr04: 2880050-2882119(+) | 618	CDD:2231 05	35.751	386	207	13	18	400	14	361	2.79E-103	315	COG00 26	PurK	Phosphoribosylaminoimidazole carboxylase (NCAIR synthetase)
LN02_05687 | LN02Chr04: 2880050-2882119(+) | 618	CDD:2231 19	55.625	160	71	0	446	605	2	161	3.50E-68	216	COG00 41	PurE	Phosphoribosylcarboxyaminoimidazole (NCAIR) mutase
LN02_05815 | LN02Chr04: 3291966-3295771(-) | 1157	CDD:2231 63	29.498	1156	683	31	26	1153	9	1060	0	1040	COG00 85	RpoB	DNA-directed RNA polymerase, beta subunit/140 kD subunit
LN02_05943 | LN02Chr04: 3761268-3767049(+) | 987	CDD:2264 06	24.194	124	74	3	280	383	739	862	1.98E-04	42.2	COG38 89	COG388 9	Predicted solute binding protein
LN02_06007 | LN02Chr04: 4001367-4003185(+) | 475	CDD:2237 60	24.885	217	116	9	243	451	43	220	1.17E-19	85	COG06 88	Psd	Phosphatidylserine decarboxylase
LN02_06135 | LN02Chr04: 4383638-4386395(-) | 877	CDD:2234 00	19.037	872	432	35	1	844	1	626	4.47E-32	130	COG03 23	MutL	DNA mismatch repair enzyme (predicted ATPase)
LN02_06199 | LN02Chr04: 4641030-4644027(+) | 946	CDD:2235 89	16.374	342	236	7	77	413	40	336	1.81E-07	51.3	COG05 15	SPS1	Serine/threonine protein kinase
LN02_06519 | LN02Chr04: 5720397-5720843(+) | 114	CDD:2275 87	61.682	107	39	2	7	112	26	131	2.04E-49	150	COG52 62	HTA1	Histone H2A

LN02_06583∣LN02Chr04: 5920913-5921797(+)∣294	CDD:223698	17.992	239	132	7	46	277	9	190	2.90E-10	56	COG0625	Gst	Glutathione S-transferase
LN02_06647∣LN02Chr05: 228122-229354(+)∣410	CDD:224594	25.128	390	239	15	8	396	45	382	3.19E-27	108	COG1680	AmpC	Beta-lactamase class C and other penicillin binding proteins
LN02_06775∣LN02Chr05: 769779-771789(+)∣533	CDD:223605	20.69	464	334	10	56	503	12	457	2.15E-25	106	COG0531	PotE	Amino acid transporters
LN02_06839∣LN02Chr05: 993575-995369(-)∣522	CDD:225035	24.309	362	205	8	131	492	98	390	5.83E-44	157	COG2124	CypX	Cytochrome P450
LN02_06903∣LN02Chr05: 1219597-1221947(-)∣757	CDD:223229	51.748	429	197	6	4	428	1	423	0	588	COG0151	PurD	Phosphoribosylamine-glycine ligase
LN02_06903∣LN02Chr05: 1219597-1221947(-)∣757	CDD:223228	51.335	337	134	4	436	744	4	338	8.30E-148	433	COG0150	PurM	Phosphoribosylaminoimidazole (AIR) synthetase
LN02_06967∣LN02Chr05: 1821254-1822928(-)∣445	CDD:224481	25.648	347	159	12	94	432	64	319	9.44E-36	132	COG1565	COG1565	Uncharacterized conserved protein
LN02_07415∣LN02Chr05: 3730241-3734627(-)∣1387	CDD:223540	32.164	513	275	15	726	1217	3	463	6.14E-88	292	COG0464	SpoVK	ATPases of the AAA+ class
LN02_07479∣LN02Chr05: 3906405-3908355(+)∣631	CDD:223087	28.713	505	302	15	113	600	9	472	4.46E-115	349	COG0008	GlnS	Glutamyl- and glutaminyl-tRNA synthetases
LN02_07671∣LN02Chr05: 4788259-4789028(+)∣153	CDD:224881	31.818	88	59	1	27	114	2	88	1.18E-16	68.9	COG1970	MscL	Large-conductance mechanosensitive channel
LN02_07799∣LN02Chr05: 5282029-5283020(+)∣260	CDD:223711	38.618	246	135	6	4	245	1	234	5.30E-65	199	COG0638	PRE1	20S proteasome, alpha and beta subunits
LN02_08183∣LN02Chr06: 1700428-1703225(-)∣907	CDD:227476	23.504	234	153	6	453	667	206	432	3.02E-17	82.9	COG5147	REB1	Myb superfamily proteins, including transcription factors and mRNA splicing factors
LN02_08439∣LN02Chr06: 2616738-2617963(-)∣336	CDD:225491	25.123	203	126	6	136	335	300	479	3.06E-21	91	COG2940	COG2940	Proteins containing SET domain

LN02_08887∣LN02Chr07: 695198-695750(+)∣118	CDD:223437	25	96	67	2	1	95	1	92	8.92E-12	54.6	COG0360	RpsF	Ribosomal protein S6
LN02_08951∣LN02Chr07: 937260-939123(-)∣442	CDD:223364	26.596	282	197	5	12	287	1	278	1.16E-59	193	COG0287	TyrA	Prephenate dehydrogenase
LN02_09143∣LN02Chr07: 1559396-1561080(-)∣347	CDD:223991	32.011	353	222	10	8	346	2	350	2.85E-77	238	COG1063	Tdh	Threonine dehydrogenase and related Zn-dependent dehydrogenases
LN02_00056∣LN02Chr01: 350246-351679(+)∣477	CDD:227673	28.294	463	149	6	18	475	7	291	1.94E-57	189	COG5383	COG5383	Uncharacterized protein conserved in bacteria
LN02_00504∣LN02Chr01: 2307858-2308556(-)∣232	CDD:224931	21.145	227	136	7	8	232	1	186	3.13E-11	57.4	COG2020	STE14	Putative protein-S-isoprenylcysteine methyltransferase
LN02_00568∣LN02Chr01: 2506857-2510090(+)∣1077	CDD:223446	37.592	407	229	10	674	1077	203	587	4.00E-115	365	COG0369	CysJ	Sulfite reductase, alpha subunit (flavoprotein)
LN02_00568∣LN02Chr01: 2506857-2510090(+)∣1077	CDD:223746	23.333	210	130	6	199	383	136	339	4.55E-15	75	COG0674	PorA	Pyruvate:ferredoxin oxidoreductase and related 2-oxoacid:ferredoxin oxidoreductases, alpha subunit

LN02_00568|LN02Chr01:2506857-2510090(+)|1077	CDD:223946	25.743	101	72	2	463	563	33	130	5.33E-04	39.7	COG1014	PorG	Pyruvate:ferredoxin oxidoreductase and related 2-oxoacid:ferredoxin oxidoreductases, gamma subunit
LN02_00632|LN02Chr01:2802432-2804133(+)|516	CDD:225035	25.737	373	199	10	115	487	86	380	2.09E-44	158	COG2124	CypX	Cytochrome P450
LN02_00696|LN02Chr01:3075676-3076967(-)|335	CDD:223117	36.615	325	180	12	20	331	2	313	2.88E-86	259	COG0039	Mdh	Malate/lactate dehydrogenases
LN02_00760|LN02Chr01:3273941-3274864(-)|285	CDD:223921	49.693	163	78	2	85	246	17	176	2.15E-66	202	COG0852	NuoC	NADH:ubiquinone oxidoreductase 27 kD subunit
LN02_01208|LN02Chr01:4887629-4888462(+)|216	CDD:225124	21.29	155	118	2	38	188	6	160	3.15E-06	43.7	COG2214	CbpA	DnaJ-class molecular chaperone
LN02_01336|LN02Chr01:5336419-5337765(-)|347	CDD:227410	29.167	72	49	2	26	95	10	81	6.52E-10	54.2	COG5078	COG5078	Ubiquitin-protein ligase
LN02_01400|LN02Chr01:5587815-5589102(-)|346	CDD:223280	16.725	287	177	10	26	258	17	295	2.41E-35	128	COG0202	RpoA	DNA-directed RNA polymerase, alpha subunit/40 kD subunit
LN02_01464|LN02Chr01:5904518-5905441(+)|307	CDD:223165	47.465	217	110	3	53	266	3	218	7.05E-82	244	COG0087	RplC	Ribosomal protein L3
LN02_01528|LN02Chr01:6117378-6119159(+)|593	CDD:224168	22.152	158	95	8	84	233	32	169	3.48E-06	44.6	COG1247	COG1247	Sortase and related acyltransferases
LN02_01656|LN02Chr01:6601630-6603338(+)|488	CDD:225862	28.608	395	240	14	90	447	39	428	2.89E-57	193	COG3325	ChiA	Chitinase
LN02_01784|LN02Chr01:7023595-7026393(+)|932	CDD:223736	23.239	142	104	3	10	148	3	142	4.63E-19	84.1	COG0664	Crp	cAMP-binding proteins - catabolite gene activator and regulatory subunit of cAMP-dependent protein kinases
LN02_01784|LN02Chr01:7023595-7026393(+)|932	CDD:223736	24.286	140	95	3	209	348	1	129	8.07E-15	71.4	COG0664	Crp	cAMP-binding proteins - catabolite gene activator and regulatory subunit of cAMP-dependent protein kinases
LN02_01848|LN02Chr01:7209388-7211002(-)|464	CDD:224322	36.919	344	180	12	143	455	74	411	3.48E-35	134	COG1404	AprE	Subtilisin-like serine proteases
LN02_02104|LN02Chr02:626283-629741(+)|1075	CDD:223550	33.497	1021	526	21	64	1064	30	917	0	773	COG0474	MgtA	Cation transport ATPase
LN02_02232|LN02Chr02:1014805-1017276(+)|823	CDD:223589	26.421	299	190	9	43	321	2	290	6.08E-40	149	COG0515	SPS1	Serine/threonine protein kinase
LN02_02296|LN02Chr02:1256883-1259414(+)|749	CDD:224206	22.672	741	516	19	18	720	16	737	4.17E-114	360	COG1287	COG1287	Uncharacterized membrane protein, required for N-linked glycosylation
LN02_02680|LN02Chr02:2649268-2650936(+)|402	CDD:223120	26.606	218	137	6	33	248	16	212	9.77E-33	122	COG0042	COG0042	tRNA-dihydrouridine synthase
LN02_02744|LN02Chr02:2833777-2834991(-)|404	CDD:223507	26.724	348	201	14	19	362	20	317	6.42E-43	150	COG0430	RCL1	RNA 3'-terminal phosphate cyclase
LN02_02808|LN02Chr02:3075002-3077599(-)|865	CDD:227934	20.976	205	152	4	534	736	448	644	1.65E-12	68.3	COG5647	COG5647	Cullin, a subunit of E3 ubiquitin ligase
LN02_03064|LN02Chr02:3969284-3970342(-)|258	CDD:223184	27.344	256	169	6	1	254	1	241	4.30E-58	182	COG0106	HisA	Phosphoribosylformimino-5-aminoimidazole carboxamide ribonucleotide (ProFAR) isomerase

LN02_03192∣LN02Chr02: 4442401-4446629(+)∣1309	CDD:227637	35.177	651	324	17	21	670	3	556	8.16E-152	467	COG5329	COG5329	Phosphoinositide polyphosphatase (Sac family)
LN02_03192∣LN02Chr02: 4442401-4446629(+)∣1309	CDD:227698	43.575	358	189	6	618	969	3	353	1.32E-114	364	COG5411	COG5411	Phosphatidylinositol 5-phosphate phosphatase
LN02_03256∣LN02Chr02: 4646657-4648348(+)∣521	CDD:223796	26.087	230	160	3	239	463	82	306	1.04E-22	95.8	COG0724	COG0724	RNA-binding proteins (RRM domain)
LN02_03448∣LN02Chr02: 5395911-5396809(+)∣183	CDD:223205	42.105	190	96	6	5	181	4	192	3.87E-59	180	COG0127	COG0127	Xanthosine triphosphate pyrophosphatase
LN02_03512∣LN02Chr02: 5832059-5833619(-)∣441	CDD:224013	41.018	334	186	7	43	372	1	327	8.81E-123	358	COG1088	RfbB	dTDP-D-glucose 4,6-dehydratase
LN02_03704∣LN02Chr03: 362909-364254(+)∣435	CDD:227884	26.263	99	70	2	189	287	119	214	1.08E-04	41	COG5597	COG5597	Alpha-N-acetylglucosamine transferase
LN02_03960∣LN02Chr03: 1249065-1251277(+)∣701	CDD:223416	30.633	679	414	16	39	701	45	682	1.22E-167	494	COG0339	Dcp	Zn-dependent oligopeptidases
LN02_04024∣LN02Chr03: 1480713-1484793(-)∣1336	CDD:224055	33.107	589	370	6	745	1333	3	567	4.08E-130	410	COG1132	MdlB	ABC-type multidrug transport system, ATPase and permease components
LN02_04024∣LN02Chr03: 1480713-1484793(-)∣1336	CDD:224055	31.687	587	372	5	85	671	7	564	1.04E-123	392	COG1132	MdlB	ABC-type multidrug transport system, ATPase and permease components
LN02_04344∣LN02Chr03: 3868047-3868671(+)∣169	CDD:227410	45.985	137	69	4	9	145	2	133	1.29E-44	141	COG5078	COG5078	Ubiquitin-protein ligase
LN02_04472∣LN02Chr03: 4268941-4270108(-)∣219	CDD:224025	24.731	186	122	4	25	192	18	203	1.06E-24	94.3	COG1100	COG1100	GTPase SAR1 and related small G proteins
LN02_04536∣LN02Chr03: 4473216-4477829(+)∣1452	CDD:223494	28.892	713	414	23	612	1316	156	783	3.45E-134	431	COG0417	PolB	DNA polymerase elongation subunit (family B)
LN02_04600∣LN02Chr03: 4744752-4746283(-)∣476	CDD:223944	39.066	471	274	5	9	473	3	466	3.94E-144	418	COG1012	PutA	NAD-dependent aldehyde dehydrogenases
LN02_04728∣LN02Chr03: 5200394-5202973(+)∣829	CDD:227827	28.736	87	46	2	633	719	301	371	3.73E-11	62.7	COG5540	COG5540	RING-finger-containing ubiquitin ligase
LN02_04792∣LN02Chr03: 5422994-5425980(-)∣967	CDD:224859	31.765	255	145	9	713	959	1	234	3.59E-47	166	COG1948	MUS81	ERCC4-type nuclease
LN02_04856∣LN02Chr03: 5791799-5795040(-)∣961	CDD:224044	24.055	291	142	7	353	643	29	240	3.01E-37	137	COG1119	ModF	ABC-type molybdenum transport system, ATPase component/photorepair protein PhrA
LN02_04856∣LN02Chr03: 5791799-5795040(-)∣961	CDD:224044	28.632	234	123	8	44	270	51	247	4.25E-20	88.1	COG1119	ModF	ABC-type molybdenum transport system, ATPase component/photorepair protein PhrA
LN02_05176∣LN02Chr04: 864857-865738(-)∣293	CDD:223959	34.191	272	139	7	20	288	16	250	5.96E-43	144	COG1028	FabG	Dehydrogenases with different specificities (related to short-chain alcohol dehydrogenases)
LN02_05240∣LN02Chr04: 1072637-1074292(+)∣551	CDD:225371	22.321	112	87	0	67	178	43	154	4.97E-05	42.6	COG2814	AraJ	Arabinose efflux permease
LN02_05432∣LN02Chr04: 1742943-1743865(+)∣267	CDD:223663	30.286	175	93	7	32	204	4	151	1.40E-23	90.8	COG0590	CumB	Cytosine/adenosine deaminases
LN02_05496∣LN02Chr04: 2106680-2107292(-)∣146	CDD:227436	30.682	88	53	5	6	89	240	323	2.88E-06	42.7	COG5105	MIH1	Mitotic inducer, protein phosphatase

LN02_05560	LN02Chr04: 2457100-2459257(-)	671	CDD:223727	23.529	374	258	12	45	414	6	355	1.77E-46	166	COG0654	UbiH	2-polyprenyl-6-methoxyphenol hydroxylase and related FAD-dependent oxidoreductases
LN02_05752	LN02Chr04: 3086399-3087163(+)	214	CDD:223589	24.793	121	82	5	89	206	14	128	5.31E-10	55.1	COG0515	SPS1	Serine/threonine protein kinase
LN02_06008	LN02Chr04: 4003441-4004625(-)	254	CDD:223698	33.01	206	125	7	9	210	4	200	1.45E-51	164	COG0625	Gst	Glutathione S-transferase
LN02_06072	LN02Chr04: 4179952-4181551(+)	455	CDD:225180	20	420	309	14	13	417	33	440	1.81E-05	43.8	COG2271	UhpC	Sugar phosphate permease
LN02_06200	LN02Chr04: 4645065-4646797(-)	520	CDD:223915	28.105	306	144	9	167	458	2	245	2.28E-64	207	COG0846	SIR2	NAD-dependent protein deacetylases, SIR2 family
LN02_06328	LN02Chr04: 5045932-5046889(+)	259	CDD:223738	23.529	187	127	7	26	203	31	210	2.86E-10	56	COG0666	Arp	FOG: Ankyrin repeat
LN02_06392	LN02Chr04: 5238780-5242688(+)	1277	CDD:226947	38.072	1006	530	17	292	1277	109	1041	0	847	COG4581	COG4581	Superfamily II RNA helicase
LN02_06456	LN02Chr04: 5445933-5447309(+)	391	CDD:223464	28.108	370	246	4	22	390	15	365	1.34E-61	200	COG0387	ChaA	Ca2+/H+ antiporter
LN02_06712	LN02Chr05: 497029-499179(+)	595	CDD:226406	24.719	89	65	2	251	339	767	853	7.82E-04	39.5	COG3889	COG3889	Predicted solute binding protein
LN02_06840	LN02Chr05: 997643-999586(+)	601	CDD:225646	22.139	533	351	12	60	577	4	487	9.24E-86	273	COG3104	PTR2	Dipeptide/tripeptide permease
LN02_06904	LN02Chr05: 1223701-1225434(+)	577	CDD:227370	55.422	83	36	1	8	90	3	84	3.04E-33	124	COG5037	TOS9	Gluconate transport-inducing protein
LN02_06968	LN02Chr05: 1823705-1825324(+)	467	CDD:223589	26.488	336	204	11	108	430	1	306	4.39E-38	140	COG0515	SPS1	Serine/threonine protein kinase
LN02_07096	LN02Chr05: 2563076-2563477(-)	133	CDD:224920	27.642	123	87	2	9	131	3	123	1.25E-07	44.6	COG2009	SdhC	Succinate dehydrogenase/fumarate reductase, cytochrome b subunit
LN02_07160	LN02Chr05: 2772027-2773643(+)	440	CDD:227325	35.952	420	235	9	27	433	3	401	2.15E-164	466	COG4992	ArgD	Ornithine/acetylornithine aminotransferase
LN02_07416	LN02Chr05: 3735275-3738106(+)	718	CDD:223669	17.593	216	155	7	328	541	23	217	7.61E-05	42.3	COG0596	MhpC	Predicted hydrolases or acyltransferases (alpha/beta hydrolase superfamily)
LN02_07480	LN02Chr05: 3909373-3911037(+)	480	CDD:224493	30.811	370	176	11	34	401	3	294	1.47E-65	211	COG1577	ERG12	Mevalonate kinase
LN02_07544	LN02Chr05: 4118620-4119651(-)	298	CDD:223669	22.059	272	199	6	29	296	19	281	7.62E-21	87.4	COG0596	MhpC	Predicted hydrolases or acyltransferases (alpha/beta hydrolase superfamily)
LN02_07800	LN02Chr05: 5283321-5285712(-)	721	CDD:224162	40.773	699	374	14	30	713	4	677	0	629	COG1241	MCM2	Predicted ATPase involved in replication control, Cdc46/Mcm family
LN02_08120	LN02Chr06: 1321051-1322038(+)	284	CDD:223483	20.27	148	110	4	1	147	1	141	1.37E-10	56.7	COG0406	phoE	Broad specificity phosphatase PhoE and related phosphatases
LN02_08312	LN02Chr06: 2081841-2083287(-)	443	CDD:223408	31.844	358	192	10	74	430	2	308	2.03E-77	240	COG0331	FabD	(acyl-carrier-protein) S-malonyltransferase
LN02_08824	LN02Chr07: 460860-461720(+)	256	CDD:223698	26.613	124	78	5	73	192	52	166	3.74E-07	46.7	COG0625	Gst	Glutathione S-transferase
LN02_08952	LN02Chr07: 939473-940567(+)	364	CDD:227511	29.157	415	227	17	1	364	67	465	7.26E-41	146	COG5184	ATS1	Alpha-tubulin suppressor and related RCC1 domain-containing proteins

LN02_09080|LN02Chr07: 1358258-1360731(+)|701	CDD:227367	25.521	192	119	4	502	692	103	271	2.76E-25	103	COG5034	TNG2	Chromatin remodeling protein, contains PhD zinc finger
LN02_09080|LN02Chr07: 1358258-1360731(+)|701	CDD:226198	20	130	74	5	95	218	3	108	8.67E-04	38.1	COG3672	COG3672	Predicted transglutaminase-like cysteine proteinase
LN02_00185|LN02Chr01: 866005-866745(-)|246	CDD:223738	30.37	135	87	3	21	154	80	208	1.41E-07	47.9	COG0666	Arp	FOG: Ankyrin repeat
LN02_00505|LN02Chr01: 2309441-2311021(+)|448	CDD:223477	23.308	133	82	6	1	119	3	129	3.21E-05	41.9	COG0400	COG0400	Predicted esterase
LN02_00569|LN02Chr01: 2513826-2515402(-)|428	CDD:223737	18.632	424	288	14	1	409	4	385	1.47E-21	92.7	COG0665	DadA	Glycine/D-amino acid oxidases (deaminating)
LN02_00633|LN02Chr01: 2804448-2807098(-)|809	CDD:223738	34.066	182	106	5	7	181	13	187	1.19E-16	77.2	COG0666	Arp	FOG: Ankyrin repeat
LN02_00633|LN02Chr01: 2804448-2807098(-)|809	CDD:223738	39.759	83	42	2	291	365	109	191	6.76E-11	60.2	COG0666	Arp	FOG: Ankyrin repeat
LN02_00633|LN02Chr01: 2804448-2807098(-)|809	CDD:224322	22.973	296	159	11	484	753	144	396	6.42E-14	71.9	COG1404	AprE	Subtilisin-like serine proteases
LN02_00761|LN02Chr01: 3275286-3281168(+)|1262	CDD:227502	40.594	202	80	4	9	179	11	203	1.16E-53	192	COG5175	MOT2	Transcriptional repressor
LN02_00825|LN02Chr01: 3519029-3524156(+)|1568	CDD:225201	19.403	335	237	12	36	355	77	393	7.28E-07	50.5	COG2319	COG2319	FOG: WD40 repeat
LN02_01209|LN02Chr01: 4889038-4890778(-)|532	CDD:223560	43.041	388	183	9	73	458	2	353	5.25E-129	378	COG0484	DnaJ	DnaJ-class molecular chaperone with C-terminal Zn finger domain
LN02_01401|LN02Chr01: 5589527-5590867(+)|446	CDD:225201	28.754	313	205	11	59	360	58	363	4.12E-33	127	COG2319	COG2319	FOG: WD40 repeat
LN02_01529|LN02Chr01: 6119908-6121921(-)|556	CDD:223535	34.774	532	330	11	14	536	1	524	5.86E-163	471	COG0459	GroL	Chaperonin GroEL (HSP60 family)
LN02_01721|LN02Chr01: 6855329-6857075(+)|414	CDD:223739	28.485	330	212	6	16	337	1	314	1.60E-91	276	COG0667	Tas	Predicted oxidoreductases (related to aryl-alcohol dehydrogenases)
LN02_01785|LN02Chr01: 7027245-7028411(+)|349	CDD:223742	23.789	227	156	7	125	348	21	233	1.48E-14	69.3	COG0670	COG0670	Integral membrane protein, interacts with FtsH
LN02_01977|LN02Chr02: 186970-187605(+)|211	CDD:223698	30.516	213	133	6	3	211	1	202	2.89E-34	118	COG0625	Gst	Glutathione S-transferase
LN02_02169|LN02Chr02: 809670-810639(-)|238	CDD:223710	25.822	213	149	4	16	221	2	212	3.69E-35	122	COG0637	COG0637	Predicted phosphatase/phosphohexomutase
LN02_02361|LN02Chr02: 1548109-1549839(+)|576	CDD:223589	29.167	72	49	2	280	350	76	146	6.93E-04	39	COG0515	SPS1	Serine/threonine protein kinase
LN02_02489|LN02Chr02: 1996567-1998459(+)|542	CDD:227423	41.355	428	237	7	125	542	28	451	4.07E-144	420	COG5092	NMT1	N-myristoyl transferase
LN02_02553|LN02Chr02: 2208373-2216055(+)|2375	CDD:225858	33.399	1021	581	26	1	1000	3	945	0	602	COG3321	COG3321	Polyketide synthase modules and related proteins
LN02_02553|LN02Chr02: 2208373-2216055(+)|2375	CDD:223677	28.664	307	188	11	1663	1952	20	312	6.59E-28	113	COG0604	Qor	NADPH:quinone reductase and related Zn-dependent oxidoreductases
LN02_02553|LN02Chr02: 2208373-2216055(+)|2375	CDD:223959	23.429	175	124	5	2005	2172	19	190	1.05E-05	46.3	COG1028	FabG	Dehydrogenases with different specificities (related to short-chain alcohol dehydrogenases)

LN02_02873 | LN02Chr02: 3286117-3289328(+) | 1044	CDD:223627	31.271	582	336	14	469	1027	326	866	2.16E-92	311	COG0553	HepA	Superfamily II DNA/RNA helicases, SNF2 family
LN02_02937 | LN02Chr02: 3521767-3523867(-) | 547	CDD:223483	26.667	195	119	5	264	451	1	178	1.13E-21	90.6	COG0406	phoE	Broad specificity phosphatase PhoE and related phosphatases
LN02_03001 | LN02Chr02: 3722228-3722959(+) | 243	CDD:225288	28.889	90	59	2	154	241	428	514	1.06E-06	46.2	COG2433	COG2433	Uncharacterized conserved protein
LN02_03065 | LN02Chr02: 3970871-3972930(+) | 522	CDD:225056	40.551	254	147	2	227	480	3	252	2.56E-82	254	COG2145	ThiM	Hydroxyethylthiazole kinase, sugar kinase family
LN02_03065 | LN02Chr02: 3970871-3972930(+) | 522	CDD:223429	48.066	181	86	3	6	186	4	176	7.78E-54	178	COG0352	ThiE	Thiamine monophosphate synthase
LN02_03129 | LN02Chr02: 4188375-4189899(+) | 429	CDD:223381	47.897	428	200	8	1	426	2	408	5.47E-160	454	COG0304	FabB	3-oxoacyl-(acyl-carrier-protein) synthase
LN02_03321 | LN02Chr02: 4858897-4859866(-) | 283	CDD:223611	25.397	63	41	3	74	135	17	74	6.77E-06	42	COG0537	Hit	Diadenosine tetraphosphate (Ap4A) hydrolase and other HIT family hydrolases
LN02_03385 | LN02Chr02: 5049042-5050730(-) | 508	CDD:223950	26.977	215	86	5	236	450	1	144	6.15E-21	86.6	COG1019	COG1019	Predicted nucleotidyltransferase
LN02_03513 | LN02Chr02: 5834678-5835706(+) | 294	CDD:223528	20.847	307	199	9	7	277	2	300	5.87E-18	79.6	COG0451	WcaG	Nucleoside-diphosphate-sugar epimerases
LN02_04153 | LN02Chr03: 2854287-2858879(-) | 1488	CDD:224117	16.149	644	429	15	627	1194	304	912	5.80E-07	51.3	COG1196	Smc	Chromosome segregation ATPases
LN02_04217 | LN02Chr03: 3395237-3396987(+) | 526	CDD:223777	27.273	187	125	5	241	420	27	209	2.05E-21	90.2	COG0705	COG0705	Membrane associated serine protease
LN02_04281 | LN02Chr03: 3660347-3663922(-) | 1069	CDD:223956	36.373	932	554	14	26	955	16	910	0	777	COG1025	Ptr	Secreted/periplasmic Zn-dependent peptidases, insulinase-like
LN02_04345 | LN02Chr03: 3869599-3870826(-) | 374	CDD:224137	19.318	176	112	6	200	373	74	221	7.84E-06	44	COG1216	COG1216	Predicted glycosyltransferases
LN02_04665 | LN02Chr03: 4966897-4968937(+) | 620	CDD:226070	21.204	382	229	21	207	541	92	448	2.19E-18	85.6	COG3540	PhoD	Phosphodiesterase/alkaline phosphatase D
LN02_04729 | LN02Chr03: 5203596-5205063(-) | 371	CDD:223214	35.733	375	169	13	16	363	2	331	2.50E-97	290	COG0136	Asd	Aspartate-semialdehyde dehydrogenase
LN02_04921 | LN02Chr03: 5971023-5972969(-) | 570	CDD:223520	28.274	481	294	10	12	488	1	434	6.99E-85	272	COG0443	DnaK	Molecular chaperone
LN02_05049 | LN02Chr04: 451289-452133(-) | 152	CDD:224644	23.022	139	105	1	12	150	5	141	1.68E-18	73.9	COG1730	GIM5	Predicted prefoldin, molecular chaperone implicated in de novo protein folding
LN02_05177 | LN02Chr04: 866588-868127(-) | 422	CDD:227284	31.232	349	206	10	68	397	33	366	1.03E-69	222	COG4948	COG4948	L-alanine-DL-glutamate epimerase and related enzymes of enolase superfamily
LN02_05241 | LN02Chr04: 1076194-1078226(+) | 621	CDD:225186	27.524	614	365	16	6	615	4	541	6.65E-70	233	COG2303	BetA	Choline dehydrogenase and related flavoproteins
LN02_05369 | LN02Chr04: 1514388-1517800(+) | 1071	CDD:223138	34.294	1006	561	25	1	994	16	933	0	1017	COG0060	IleS	Isoleucyl-tRNA synthetase
LN02_05497 | LN02Chr04: 2108134-2109798(-) | 520	CDD:227445	35.433	508	245	12	14	518	2	429	1.03E-127	377	COG5114	COG5114	Histone acetyltransferase complex SAGA/ADA, subunit ADA2

LN02_05689 | LN02Chr04: 2887148-2890204(-) | 1018	CDD:224117	14.798	223	175	3	636	848	739	956	1.45E-09	59	COG1196	Smc	Chromosome segregation ATPases
LN02_05817 | LN02Chr04: 3300221-3304417(+) | 1017	CDD:227430	36.76	321	196	3	597	910	437	757	1.83E-86	292	COG5099	COG5099	RNA-binding protein of the Puf family, translational repressor
LN02_05945 | LN02Chr04: 3781735-3784267(-) | 745	CDD:224260	25.854	205	130	8	287	489	69	253	6.52E-26	107	COG1341	COG1341	Predicted GTPase or GTP-binding protein
LN02_06201 | LN02Chr04: 4647993-4652341(-) | 1390	CDD:223550	29.874	954	567	19	271	1215	51	911	0	617	COG0474	MgtA	Cation transport ATPase
LN02_06457 | LN02Chr04: 5447352-5448916(-) | 442	CDD:223464	23.136	389	264	8	53	429	3	368	4.87E-33	125	COG0387	ChaA	Ca2+/H+ antiporter
LN02_06777 | LN02Chr05: 779320-782489(+) | 956	CDD:223589	28.261	322	190	8	49	343	1	308	9.88E-50	178	COG0515	SPS1	Serine/threonine protein kinase
LN02_06841 | LN02Chr05: 1000560-1001473(-) | 244	CDD:223253	25.862	232	133	5	32	231	9	233	2.54E-29	108	COG0175	CysH	3'-phosphoadenosine 5'-phosphosulfate sulfotransferase (PAPS reductase)/FAD synthetase and related enzymes
LN02_07097 | LN02Chr05: 2564274-2565231(-) | 273	CDD:223760	24.231	260	162	9	7	263	12	239	2.74E-46	152	COG0688	Psd	Phosphatidylserine decarboxylase
LN02_07225 | LN02Chr05: 3029178-3029621(+) | 112	CDD:224969	42.857	112	61	2	1	112	1	109	8.40E-12	54.3	COG2058	RPP1A	Ribosomal protein L12E/L44/L45/RPP1/RPP2
LN02_07289 | LN02Chr05: 3207866-3209731(+) | 569	CDD:227598	28.804	184	119	3	58	240	63	235	8.32E-36	133	COG5273	COG5273	Uncharacterized protein containing DHHC-type Zn finger
LN02_07353 | LN02Chr05: 3417814-3418676(-) | 154	CDD:223183	56.818	132	57	0	5	136	3	134	5.16E-80	230	COG0105	Ndk	Nucleoside diphosphate kinase
LN02_07417 | LN02Chr05: 3738628-3740782(+) | 548	CDD:223395	36.26	524	320	12	26	545	21	534	8.78E-140	412	COG0318	CaiC	Acyl-CoA synthetases (AMP-forming)/AMP-acid ligases II
LN02_07673 | LN02Chr05: 4795050-4796895(+) | 502	CDD:227170	21.324	272	163	12	69	330	44	274	2.07E-07	50	COG4833	COG4833	Predicted glycosyl hydrolase
LN02_07865 | LN02Chr05: 5533797-5536082(-) | 496	CDD:223605	21.397	458	336	8	8	465	16	449	8.64E-24	101	COG0531	PotE	Amino acid transporters
LN02_07929 | LN02Chr05: 5712601-5714288(-) | 472	CDD:225180	19.876	322	225	15	28	336	31	332	2.87E-08	52.7	COG2271	UhpC	Sugar phosphate permease
LN02_08313 | LN02Chr06: 2083836-2085711(-) | 553	CDD:227828	20.988	81	64	0	58	138	70	150	7.60E-05	40.7	COG5541	RET3	Vesicle coat complex COPI, zeta subunit
LN02_08441 | LN02Chr06: 2621078-2624158(-) | 957	CDD:227427	23.84	927	528	24	8	931	6	757	5.92E-141	436	COG5096	COG5096	Vesicle coat complex, various subunits
LN02_08633 | LN02Chr06: 3222288-3224189(-) | 613	CDD:223589	23.776	286	177	11	334	591	2	274	3.23E-18	84	COG0515	SPS1	Serine/threonine protein kinase
LN02_09081 | LN02Chr07: 1361479-1362747(-) | 358	CDD:223099	28.713	303	138	4	37	339	15	239	2.11E-71	220	COG0020	UppS	Undecaprenyl pyrophosphate synthase
LN02_09145 | LN02Chr07: 1563681-1564414(-) | 170	CDD:227378	55.882	102	45	0	1	102	1	102	1.19E-53	163	COG5045	COG5045	Ribosomal protein S10E
LN02_09273 | LN02Chr07: 2006308-2007239(-) | 259	CDD:227229	40.816	49	25	3	100	147	5	50	6.07E-05	37.9	COG4892	COG4892	Predicted heme/steroid binding protein
LN02_00058 | LN02Chr01: 353868-355431(+) | 491	CDD:223587	41.589	428	242	7	67	488	28	453	1.43E-143	419	COG0513	SrmB	Superfamily II DNA and RNA helicases

LN02_00186∣LN02Chr01:868184-871027(+)∣890	CDD:227637	32.252	493	282	15	91	572	62	513	9.18E-124	383	COG5329	COG5329	Phosphoinositide polyphosphatase (Sac family)
LN02_00314∣LN02Chr01:1756956-1758707(+)∣512	CDD:225180	18.103	232	182	5	127	356	90	315	1.48E-08	53.8	COG2271	UhpC	Sugar phosphate permease
LN02_00378∣LN02Chr01:1952068-1952985(-)∣243	CDD:226607	39.894	188	93	4	55	240	47	216	9.52E-43	141	COG4122	COG4122	Predicted O-methyltransferase
LN02_00442∣LN02Chr01:2126276-2128844(-)∣826	CDD:223977	28.904	858	479	28	67	820	23	853	0	660	COG1048	AcnA	Aconitase A
LN02_00634∣LN02Chr01:2807668-2808540(+)∣290	CDD:227002	23.358	137	97	5	57	188	63	196	1.48E-05	43.3	COG4655	COG4655	Predicted membrane protein
LN02_00890∣LN02Chr01:3694349-3696746(+)∣667	CDD:226735	39.259	270	133	7	6	267	1	247	3.95E-68	220	COG4285	COG4285	Uncharacterized conserved protein
LN02_00890∣LN02Chr01:3694349-3696746(+)∣667	CDD:223417	29.478	268	147	12	403	659	2	238	1.06E-34	128	COG0340	BirA	Biotin-(acetyl-CoA carboxylase) ligase
LN02_00954∣LN02Chr01:3915037-3915985(-)∣298	CDD:225714	21.264	174	110	6	87	250	71	227	3.20E-07	47.8	COG3173	COG3173	Predicted aminoglycoside phosphotransferase
LN02_01146∣LN02Chr01:4540527-4541624(-)∣331	CDD:225856	28.788	132	85	3	23	150	2	128	4.09E-12	62.5	COG3319	COG3319	Thioesterase domains of type I polyketide synthases or non-ribosomal peptide synthetases
LN02_01210∣LN02Chr01:4894129-4894777(-)∣166	CDD:224961	28.431	102	69	3	52	153	33	130	4.94E-12	57.4	COG2050	PaaI	HGG motif-containing thioesterase, possibly involved in aromatic compounds catabolism
LN02_01402∣LN02Chr01:5592177-5596537(-)∣1394	CDD:223738	21.905	210	146	4	873	1068	2	207	1.16E-09	57.5	COG0666	Arp	FOG: Ankyrin repeat
LN02_01658∣LN02Chr01:6604424-6605746(-)∣378	CDD:225086	38.721	297	158	9	88	373	2	285	1.40E-62	199	COG2175	TauD	Probable taurine catabolism dioxygenase
LN02_01722∣LN02Chr01:6857547-6858962(-)∣354	CDD:225201	23.41	346	209	12	11	318	103	430	3.07E-18	82.8	COG2319	COG2319	FOG: WD40 repeat
LN02_01786∣LN02Chr01:7028902-7029590(-)∣201	CDD:223303	44.91	167	88	2	34	199	7	170	5.09E-79	230	COG0225	MsrA	Peptide methionine sulfoxide reductase
LN02_01850∣LN02Chr01:7217030-7217602(+)∣190	CDD:224897	38.202	178	100	4	9	186	3	170	2.08E-52	163	COG1986	COG1986	Inosine/xanthosine triphosphatase
LN02_01914∣LN02Chr01:7400427-7401876(+)∣388	CDD:225344	19.595	148	101	6	50	180	84	230	3.03E-09	55.2	COG2730	BglC	Endoglucanase
LN02_01978∣LN02Chr02:187709-189118(-)∣469	CDD:224732	22.422	446	291	14	25	461	1	400	3.15E-34	129	COG1819	COG1819	Glycosyl transferases, related to UDP-glucuronosyltransferase
LN02_02106∣LN02Chr02:634072-635397(+)∣441	CDD:223088	46.296	216	104	4	30	245	4	207	4.80E-78	238	COG0009	SUA5	Putative translation factor (SUA5)
LN02_02170∣LN02Chr02:811650-812757(+)∣275	CDD:224950	27.753	227	134	9	22	242	3	205	1.15E-17	76.6	COG2039	Pcp	Pyrrolidone-carboxylate peptidase (N-terminal pyroglutamyl peptidase)
LN02_02234∣LN02Chr02:1021266-1022830(+)∣426	CDD:223552	37.908	153	83	3	40	190	32	174	8.27E-47	158	COG0476	ThiF	Dinucleotide-utilizing enzymes involved in molybdopterin and thiamine biosynthesis family 2
LN02_02298∣LN02Chr02:1263549-1267384(-)∣1212	CDD:223589	24.39	369	236	10	293	645	1	342	1.65E-47	172	COG0515	SPS1	Serine/threonine protein kinase

LN02_02362∣LN02Chr02: 1552608-1553333(-)∣116	CDD:2237 96	36.275	102	62	1	13	111	115	216	6.07E-17	71.5	COG07 24	COG072 4	RNA-binding proteins (RRM domain)
LN02_02426∣LN02Chr02: 1779728-1781308(+)∣526	CDD:2233 54	21.154	468	313	16	32	481	21	450	3.60E-41	151	COG02 77	GlcD	FAD/FMN-containing dehydrogenases
LN02_02490∣LN02Chr02: 2000006-2001135(+)∣308	CDD:2237 96	27	100	73	0	64	163	98	197	4.30E-12	62.7	COG07 24	COG072 4	RNA-binding proteins (RRM domain)
LN02_02618∣LN02Chr02: 2436616-2438994(-)∣771	CDD:2264 06	22.807	171	107	5	224	384	692	847	3.59E-04	41	COG38 89	COG388 9	Predicted solute binding protein
LN02_02682∣LN02Chr02: 2654660-2655827(+)∣327	CDD:2230 99	37.615	109	67	1	220	327	136	244	4.20E-25	98.5	COG00 20	UppS	Undecaprenyl pyrophosphate synthase
LN02_03002∣LN02Chr02: 3723617-3725209(-)∣485	CDD:2232 08	25.926	189	75	3	8	196	15	138	3.98E-28	110	COG01 30	TruB	Pseudouridine synthase
LN02_03066∣LN02Chr02: 3973483-3975731(-)∣570	CDD:2257 11	25.962	104	64	3	45	137	289	390	1.00E-04	42.2	COG31 70	FimV	Tfp pilus assembly protein FimV
LN02_03194∣LN02Chr02: 4451710-4453173(-)∣275	CDD:2273 73	65.683	271	89	1	1	271	1	267	2.11E-136	383	COG50 40	BMH1	14-3-3 family protein
LN02_03322∣LN02Chr02: 4860244-4862597(-)∣737	CDD:2245 57	31.777	664	353	20	2	644	98	682	2.32E-139	427	COG16 43	HrpA	HrpA-like helicases
LN02_03450∣LN02Chr02: 5400140-5403468(+)∣1068	CDD:2274 93	28.254	630	392	25	395	1004	5	594	2.90E-122	385	COG51 64	SPT5	Transcription elongation factor
LN02_03450∣LN02Chr02: 5400140-5403468(+)∣1068	CDD:2233 28	15.287	157	107	7	230	362	2	156	3.11E-04	40	COG02 50	NusG	Transcription antiterminator
LN02_03642∣LN02Chr03: 95236-95871(-)∣211	CDD:2231 49	23.737	198	87	5	14	211	11	144	1.50E-17	73.2	COG00 71	IbpA	Molecular chaperone (small heat shock protein)
LN02_04026∣LN02Chr03: 1490126-1492889(-)∣709	CDD:2241 17	16.438	365	283	4	135	485	657	1013	1.07E-11	65.5	COG11 96	Smc	Chromosome segregation ATPases
LN02_04026∣LN02Chr03: 1490126-1492889(-)∣709	CDD:2241 17	17.949	273	215	3	358	630	669	932	2.96E-10	60.5	COG11 96	Smc	Chromosome segregation ATPases
LN02_04026∣LN02Chr03: 1490126-1492889(-)∣709	CDD:2274 81	25.333	75	45	3	628	695	163	233	3.14E-05	43.1	COG51 52	COG515 2	Uncharacterized conserved protein, contains RING and CCCH-type Zn-fingers
LN02_04346∣LN02Chr03: 3871848-3873391(+)∣305	CDD:2237 96	40.741	81	47	1	80	159	108	188	1.91E-10	57.6	COG07 24	COG072 4	RNA-binding proteins (RRM domain)
LN02_04410∣LN02Chr03: 4093301-4096259(+)∣941	CDD:2273 67	50.704	71	30	3	856	926	206	271	3.55E-23	97.3	COG50 34	TNG2	Chromatin remodeling protein, contains PhD zinc finger
LN02_04538∣LN02Chr03: 4484506-4489723(-)∣1710	CDD:2241 17	16.727	275	183	3	180	451	705	936	5.55E-12	67.8	COG11 96	Smc	Chromosome segregation ATPases
LN02_04538∣LN02Chr03: 4484506-4489723(-)∣1710	CDD:2274 30	20.106	189	141	3	933	1114	148	333	8.75E-04	40.9	COG50 99	COG509 9	RNA-binding protein of the Puf family, translational repressor
LN02_04922∣LN02Chr03: 5973350-5974170(+)∣245	CDD:2240 22	27.6	250	149	10	1	244	4	227	1.59E-31	113	COG10 97	RRP4	RNA-binding protein Rrp4 and related proteins (contain S1 domain and KH domain)
LN02_04986∣LN02Chr04: 279805-282213(+)∣726	CDD:2235 89	34.643	280	152	7	74	325	2	278	1.11E-60	206	COG05 15	SPS1	Serine/threonine protein kinase

LN02_05050 | LN02Chr04: 452519-454661(-) | 609	CDD:2235 19	33.652	523	272	17	71	585	26	481	5.13E-113	344	COG04 42	ProS	Prolyl-tRNA synthetase
LN02_05498 | LN02Chr04: 2110704-2112036(+) | 383	CDD:2240 10	42.857	371	174	8	10	379	2	335	1.30E-115	337	COG10 85	GalT	Galactose-1-phosphate uridylyltransferase
LN02_05626 | LN02Chr04: 2670872-2673902(-) | 981	CDD:2241 57	20.93	430	231	14	9	432	7	333	1.96E-25	107	COG12 36	YSH1	Predicted exonuclease of the beta-lactamase fold involved in RNA processing
LN02_05818 | LN02Chr04: 3306472-3309083(-) | 489	CDD:2245 47	52.217	203	87	2	287	489	2	194	7.64E-83	251	COG16 32	RPL15A	Ribosomal protein L15E
LN02_06138 | LN02Chr04: 4393260-4394222(+) | 320	CDD:2239 85	25	120	71	5	73	187	5	110	8.98E-05	40	COG10 57	NadD	Nicotinic acid mononucleotide adenylyltransferase
LN02_06266 | LN02Chr04: 4843626-4844920(-) | 377	CDD:2237 64	51.542	227	105	3	77	303	1	222	6.01E-114	328	COG06 92	Ung	Uracil DNA glycosylase
LN02_06458 | LN02Chr04: 5449239-5450704(+) | 408	CDD:2234 64	32.877	365	223	5	46	397	13	368	1.28E-52	177	COG03 87	ChaA	Ca2+/H+ antiporter
LN02_06778 | LN02Chr05: 783873-785317(+) | 393	CDD:2275 34	63.251	283	104	0	98	380	30	312	9.37E-146	413	COG52 09	RCD1	Uncharacterized protein involved in cell differentiation/sexual development
LN02_06842 | LN02Chr05: 1002623-1003636(-) | 297	CDD:2260 40	30.822	292	172	11	7	283	10	286	1.34E-42	146	COG35 09	LpqC	Poly(3-hydroxybutyrate) depolymerase
LN02_07354 | LN02Chr05: 3419731-3422740(+) | 965	CDD:2244 18	31.761	721	418	18	153	869	82	732	1.63E-172	519	COG15 01	COG150 1	Alpha-glucosidases, family 31 of glycosyl hydrolases
LN02_07482 | LN02Chr05: 3912136-3912885(-) | 214	CDD:2274 57	39.378	193	111	4	19	211	22	208	9.07E-54	168	COG51 28	COG512 8	Transport protein particle (TRAPP) complex subunit
LN02_07546 | LN02Chr05: 4123947-4124609(+) | 220	CDD:2272 32	61.818	55	20	1	67	120	8	62	4.98E-17	69.9	COG48 95	COG489 5	Uncharacterized conserved protein
LN02_07674 | LN02Chr05: 4797514-4798849(-) | 396	CDD:2231 57	36.269	386	213	11	10	395	2	354	1.60E-98	294	COG00 79	HisC	Histidinol-phosphate/aromatic aminotransferase and cobyric acid decarboxylase
LN02_07802 | LN02Chr05: 5290972-5292238(-) | 347	CDD:2232 20	23.032	343	238	10	7	347	4	322	1.08E-41	145	COG01 42	IspA	Geranylgeranyl pyrophosphate synthase
LN02_07866 | LN02Chr05: 5536739-5538604(+) | 485	CDD:2241 52	20.264	454	309	12	38	466	9	434	3.60E-29	116	COG12 31	COG123 1	Monoamine oxidase
LN02_07866 | LN02Chr05: 5536739-5538604(+) | 485	CDD:2240 70	33.735	83	46	3	1	74	80	162	8.78E-07	48.2	COG11 48	HdrA	Heterodisulfide reductase, subunit A and related polyferredoxins
LN02_08250 | LN02Chr06: 1873657-1874526(-) | 289	CDD:2235 89	19.424	139	102	4	114	251	34	163	2.57E-08	51.3	COG05 15	SPS1	Serine/threonine protein kinase
LN02_08378 | LN02Chr06: 2392487-2393410(+) | 270	CDD:2245 00	39.216	204	107	5	58	260	18	205	9.51E-39	132	COG15 84	COG158 4	Predicted membrane protein
LN02_08570 | LN02Chr06: 3044669-3046492(+) | 527	CDD:2251 80	20.093	214	158	4	21	233	9	210	1.12E-12	66.9	COG22 71	UhpC	Sugar phosphate permease
LN02_08570 | LN02Chr06: 3044669-3046492(+) | 527	CDD:2253 71	17.964	334	223	9	78	409	43	327	3.60E-05	43	COG28 14	AraJ	Arabinose efflux permease
LN02_08762 | LN02Chr07: 272482-273391(+) | 256	CDD:2253 16	36.323	223	107	7	36	253	11	203	1.53E-50	162	COG25 18	Pcm	Protein-L-isoaspartate carboxylmethyltransferase

LN02_08826|LN02Chr07: 463933-466644(-)|651	CDD:2241 97	56.716	67	29	0	584	650	1	67	6.18E-34	120	COG12 78	CspC	Cold shock proteins
LN02_08890|LN02Chr07: 699379-701930(+)|814	CDD:2233 50	27.273	55	38	1	138	190	600	654	5.23E-04	40.3	COG02 72	Lig	NAD-dependent DNA ligase (contains BRCT domain type II)
LN02_08954|LN02Chr07: 946635-947342(+)|116	CDD:2250 85	54.839	93	42	0	9	101	1	93	1.65E-32	107	COG21 74	RPL34A	Ribosomal protein L34E
LN02_09210|LN02Chr07: 1754712-1756093(-)|396	CDD:2231 62	27.895	380	146	14	16	392	2	256	2.09E-55	180	COG00 84	TatD	Mg-dependent DNase
LN02_09274|LN02Chr07: 2007899-2010020(+)|672	CDD:2244 95	17.788	208	164	3	42	242	4	211	1.72E-05	43.9	COG15 79	COG157 9	Zn-ribbon protein, possibly nucleic acid-binding
LN02_00123|LN02Chr01: 546526-549747(-)|911	CDD:2240 55	33.505	582	357	8	249	821	4	564	1.74E-139	424	COG11 32	MdlB	ABC-type multidrug transport system, ATPase and permease components
LN02_00315|LN02Chr01: 1759096-1759797(+)|183	CDD:2241 68	23.313	163	91	5	4	158	1	137	2.22E-06	42.7	COG12 47	COG124 7	Sortase and related acyltransferases
LN02_00763|LN02Chr01: 3285352-3286609(+)|398	CDD:2237 30	19.286	280	197	8	1	274	30	286	2.70E-06	45.7	COG06 57	Aes	Esterase/lipase
LN02_00827|LN02Chr01: 3526588-3528414(-)|608	CDD:2231 79	24.468	376	153	10	69	444	2	246	4.68E-54	182	COG01 01	TruA	Pseudouridylate synthase
LN02_01083|LN02Chr01: 4362876-4365011(-)|711	CDD:2279 25	34.513	678	326	15	16	689	12	575	2.55E-95	305	COG56 38	COG563 8	Uncharacterized conserved protein
LN02_01147|LN02Chr01: 4543025-4544702(+)|498	CDD:2276 40	19.524	210	145	5	33	238	18	207	7.31E-17	78.2	COG53 33	CCL1	Cdk activating kinase (CAK)/RNA polymerase II transcription initiation/nucleotide excision repair factor TFIIH/TFIIK, cyclin H subunit
LN02_01275|LN02Chr01: 5124345-5125936(-)|499	CDD:2234 49	41.337	404	211	6	56	458	12	390	3.36E-137	398	COG03 72	GltA	Citrate synthase
LN02_01595|LN02Chr01: 6411439-6415480(+)|1295	CDD:2241 17	19.732	897	590	19	239	1107	169	963	3.50E-26	114	COG11 96	Smc	Chromosome segregation ATPases
LN02_01723|LN02Chr01: 6859623-6860767(+)|315	CDD:2240 18	30.657	274	183	3	6	278	1	268	4.57E-95	279	COG10 93	SUI2	Translation initiation factor 2, alpha subunit (eIF-2alpha)
LN02_02107|LN02Chr02: 635678-636426(-)|196	CDD:2278 28	38.021	192	112	4	5	196	3	187	3.57E-53	165	COG55 41	RET3	Vesicle coat complex COPI, zeta subunit
LN02_02235|LN02Chr02: 1023237-1024458(-)|381	CDD:2232 80	23.39	295	200	5	71	359	21	295	2.44E-53	176	COG02 02	RpoA	DNA-directed RNA polymerase, alpha subunit/40 kD subunit
LN02_02363|LN02Chr02: 1555126-1555776(+)|216	CDD:2232 00	30.769	52	31	2	13	64	144	190	5.83E-04	37	COG01 22	AlkA	3-methyladenine DNA glycosylase/8-oxoguanine DNA glycosylase
LN02_02427|LN02Chr02: 1782144-1785744(-)|787	CDD:2251 77	15.455	220	165	6	376	581	204	416	7.14E-04	39.8	COG22 68	COG226 8	Uncharacterized protein conserved in bacteria
LN02_02491|LN02Chr02: 2002033-2003468(-)|400	CDD:2252 14	33.071	127	72	5	188	309	63	181	1.05E-14	69	COG23 35	COG233 5	Secreted and surface protein containing fasciclin-like repeats
LN02_02491|LN02Chr02: 2002033-2003468(-)|400	CDD:2252 14	23.387	124	78	2	53	176	79	185	2.67E-07	47.8	COG23 35	COG233 5	Secreted and surface protein containing fasciclin-like repeats
LN02_02555|LN02Chr02: 2223784-2225366(-)|465	CDD:2251 36	20.192	208	118	9	203	384	40	225	2.47E-08	51.5	COG22 26	UbiE	Methylase involved in ubiquinone/menaquinone biosynthesis

LN02_02683∣LN02Chr02: 2655988-2657161(-)∣335	CDD:2256 87	31.469	143	75	7	146	280	67	194	7.10E-21	85.9	COG31 45	AlkB	Alkylated DNA repair protein
LN02_02747∣LN02Chr02: 2840184-2842623(-)∣693	CDD:2244 23	23.699	692	446	24	1	685	2	618	2.14E-82	270	COG15 06	DAP2	Dipeptidyl aminopeptidases/acylaminoacyl-peptidases
LN02_02939∣LN02Chr02: 3527711-3530649(-)∣847	CDD:2275 32	19.154	449	287	17	129	543	305	711	1.17E-06	49.3	COG52 07	UBP14	Isopeptidase T
LN02_03067∣LN02Chr02: 3977894-3979850(-)∣615	CDD:2232 34	40.541	407	220	8	130	533	1	388	8.17E-147	427	COG01 56	BioF	7-keto-8-aminopelargonate synthetase and related enzymes
LN02_03131∣LN02Chr02: 4191883-4192668(-)∣207	CDD:2233 40	33.121	157	84	5	6	161	2	138	7.29E-37	123	COG02 62	FolA	Dihydrofolate reductase
LN02_03195∣LN02Chr02: 4455480-4456742(-)∣420	CDD:2262 00	19.872	156	111	4	183	325	135	289	2.81E-11	61.5	COG36 75	COG367 5	Predicted lipase
LN02_03451∣LN02Chr02: 5404693-5406507(-)∣380	CDD:2251 39	27.986	293	193	8	85	372	3	282	1.76E-63	201	COG22 30	Cfa	Cyclopropane fatty acid synthase and related methyltransferases
LN02_03515∣LN02Chr02: 5842674-5843941(+)∣401	CDD:2241 17	24.675	77	58	0	27	103	768	844	3.92E-05	42.8	COG11 96	Smc	Chromosome segregation ATPases
LN02_04027∣LN02Chr03: 1493628-1495466(+)∣612	CDD:2274 48	35.26	173	105	3	401	573	314	479	5.60E-33	129	COG51 18	BDP1	Transcription initiation factor TFIIIB, Bdp1 subunit
LN02_04219∣LN02Chr03: 3401916-3405448(+)∣1107	CDD:2234 94	32.394	852	479	22	126	970	11	772	0	596	COG04 17	PolB	DNA polymerase elongation subunit (family B)
LN02_04475∣LN02Chr03: 4278959-4279469(+)∣112	CDD:2258 60	41.346	104	57	2	7	110	6	105	5.69E-26	90.5	COG33 23	COG332 3	Uncharacterized protein conserved in bacteria
LN02_04539∣LN02Chr03: 4491059-4492190(+)∣346	CDD:2274 81	32.524	206	108	3	113	318	80	254	6.31E-52	170	COG51 52	COG515 2	Uncharacterized conserved protein, contains RING and CCCH-type Zn-fingers
LN02_04667∣LN02Chr03: 4970912-4971613(-)∣194	CDD:2237 06	40	100	55	4	84	182	4	99	1.01E-26	95.2	COG06 33	Fdx	Ferredoxin
LN02_04795∣LN02Chr03: 5431522-5432429(-)∣156	CDD:2234 38	37.5	72	44	1	29	99	4	75	1.03E-23	85.3	COG03 61	InfA	Translation initiation factor 1 (IF-1)
LN02_04859∣LN02Chr03: 5801666-5804755(-)∣785	CDD:2274 27	34.432	607	350	16	96	662	22	620	5.70E-138	423	COG50 96	COG509 6	Vesicle coat complex, various subunits
LN02_04923∣LN02Chr03: 5976714-5979734(-)∣308	CDD:2233 48	35.714	70	41	1	220	289	2	67	1.10E-10	58.6	COG02 70	Dcm	Site-specific DNA methylase
LN02_04987∣LN02Chr04: 282942-285048(-)∣545	CDD:2251 34	35.66	530	235	7	20	544	5	433	0	523	COG22 24	AceA	Isocitrate lyase
LN02_05051∣LN02Chr04: 454906-458102(+)∣987	CDD:2278 80	36.105	842	464	19	172	980	19	819	1.10E-168	511	COG55 93	COG559 3	Nucleic-acid-binding protein possibly involved in ribosomal biogenesis
LN02_05307∣LN02Chr04: 1319060-1320466(+)∣442	CDD:2237 32	18.317	404	268	13	37	412	35	404	4.04E-05	42.6	COG06 59	SUL1	Sulfate permease and related transporters (MFS superfamily)
LN02_05435∣LN02Chr04: 1753248-1753834(+)∣163	CDD:2256 60	32.292	96	61	2	26	119	16	109	1.31E-17	75.1	COG31 18	COG311 8	Thioredoxin domain-containing protein

LN02_05563∣LN02Chr04: 2464622-2466010(+)∣462	CDD:2252 01	25.356	351	218	11	21	354	116	439	2.48E-25	105	COG23 19	COG231 9	FOG: WD40 repeat
LN02_05691∣LN02Chr04: 2893972-2895433(-)∣422	CDD:2251 36	27.869	122	65	8	100	211	49	157	2.50E-05	42.3	COG22 26	UbiE	Methylase involved in ubiquinone/menaquinone biosynthesis
LN02_05819∣LN02Chr04: 3309937-3312760(-)∣894	CDD:2246 88	31.959	97	64	1	621	715	51	147	2.48E-12	65.1	COG17 74	COG177 4	Uncharacterized homolog of PSP1
LN02_05819∣LN02Chr04: 3309937-3312760(-)∣894	CDD:2246 88	18.807	218	127	6	499	713	77	247	6.80E-12	64	COG17 74	COG177 4	Uncharacterized homolog of PSP1
LN02_06139∣LN02Chr04: 4394558-4396537(+)∣558	CDD:2241 29	17.681	526	259	21	9	531	3	357	4.71E-15	73.9	COG12 08	GCD1	Nucleoside-diphosphate-sugar pyrophosphorylase involved in lipopolysaccharide biosynthesis/translation initiation factor 2B, gamma/epsilon subunits (eIF-2Bgamma/eIF-2Bepsilon)
LN02_06203∣LN02Chr04: 4654933-4657505(-)∣767	CDD:2235 70	25.545	321	164	15	1	316	1	251	4.30E-34	128	COG04 96	SurE	Predicted acid phosphatase
LN02_06395∣LN02Chr04: 5248055-5249761(+)∣462	CDD:2243 74	23.971	413	280	10	65	462	49	442	2.39E-31	122	COG14 57	CodB	Purine-cytosine permease and related proteins
LN02_06587∣LN02Chr05: 18993-20513(-)∣506	CDD:2235 89	27.358	106	64	3	317	413	121	222	3.60E-05	42.8	COG05 15	SPS1	Serine/threonine protein kinase
LN02_06843∣LN02Chr05: 1006149-1008678(+)∣778	CDD:2241 17	19.087	241	168	6	207	440	223	443	5.10E-05	43.9	COG11 96	Smc	Chromosome segregation ATPases
LN02_06907∣LN02Chr05: 1229403-1233912(+)∣898	CDD:2235 22	37.297	555	240	10	443	894	72	621	4.88E-172	510	COG04 45	GidA	Flavin-dependent tRNA uridine 5-carboxymethylaminomethyl modification enzyme GidA
LN02_06907∣LN02Chr05: 1229403-1233912(+)∣898	CDD:2274 87	31.579	418	242	11	46	431	1	406	1.43E-85	282	COG51 58	SEC1	Proteins involved in synaptic transmission and general secretion, Sec1 family
LN02_06971∣LN02Chr05: 1830250-1832849(-)∣839	CDD:2235 68	20.8	125	91	5	108	224	9	133	1.73E-10	57.6	COG04 94	MutT	NTP pyrophosphohydrolases including oxidative damage repair enzymes
LN02_07163∣LN02Chr05: 2779335-2781694(+)∣697	CDD:2235 89	25.765	392	220	10	23	393	2	343	2.24E-44	161	COG05 15	SPS1	Serine/threonine protein kinase
LN02_07355∣LN02Chr05: 3425394-3427079(+)∣561	CDD:2237 40	19.095	199	140	7	280	476	112	291	4.99E-12	64.2	COG06 68	MscS	Small-conductance mechanosensitive channel
LN02_07931∣LN02Chr05: 5718687-5720701(+)∣545	CDD:2232 32	29.4	500	291	13	65	539	1	463	3.84E-69	227	COG01 54	GatA	Asp-tRNAAsn/Glu-tRNAGln amidotransferase A subunit and related amidases
LN02_08123∣LN02Chr06: 1333979-1335911(-)∣496	CDD:2273 62	30.073	409	202	10	86	492	16	342	3.90E-82	255	COG50 29	CAL1	Prenyltransferase, beta subunit
LN02_08251∣LN02Chr06: 1876667-1878171(+)∣465	CDD:2232 43	54.148	458	208	2	9	465	2	458	0	637	COG01 65	ArgH	Argininosuccinate lyase
LN02_08315∣LN02Chr06: 2087240-2088406(+)∣340	CDD:2240 22	24.686	239	144	7	60	285	9	224	2.80E-42	144	COG10 97	RRP4	RNA-binding protein Rrp4 and related proteins (contain S1 domain and KH domain)

LN02_08635∣LN02Chr06: 3227488-3230103(-)∣871	CDD:2256 61	21.064	451	246	21	438	820	31	439	9.79E-13	68.3	COG31 19	AslA	Arylsulfatase A and related enzymes
LN02_08891∣LN02Chr07: 702799-705754(-)∣937	CDD:2241 17	20.238	84	67	0	42	125	831	914	1.24E-05	46.2	COG11 96	Smc	Chromosome segregation ATPases
LN02_08955∣LN02Chr07: 948045-948934(+)∣233	CDD:2262 60	32.258	62	40	1	161	222	68	127	8.68E-05	38.2	COG37 37	COG373 7	Uncharacterized conserved protein
LN02_09083∣LN02Chr07: 1372079-1372956(-)∣206	CDD:2237 84	33.898	177	102	2	38	203	6	178	4.00E-37	124	COG07 12	AtpH	F0F1-type ATP synthase, delta subunit (mitochondrial oligomycin sensitivity protein)
LN02_09211∣LN02Chr07: 1757060-1759994(+)∣897	CDD:2232 87	33.697	733	382	14	39	754	1	646	0	621	COG02 09	NrdA	Ribonucleotide reductase, alpha subunit
LN02_09275∣LN02Chr07: 2013058-2014347(-)∣384	CDD:2251 65	31.624	234	119	8	36	253	5	213	3.57E-34	128	COG22 56	MGS1	ATPase related to the helicase subunit of the Holliday junction resolvase
LN02_00252∣LN02Chr01: 1462251-1465252(-)∣953	CDD:2241 62	34.89	728	345	10	83	799	2	611	0	632	COG12 41	MCM2	Predicted ATPase involved in replication control, Cdc46/Mcm family
LN02_00380∣LN02Chr01: 1956811-1957683(-)∣183	CDD:2274 13	54.375	160	72	1	19	178	14	172	1.11E-69	207	COG50 81	COG508 1	Predicted membrane protein
LN02_00444∣LN02Chr01: 2134377-2135882(+)∣381	CDD:2239 90	61.892	370	137	3	9	378	1	366	0	567	COG10 62	AdhC	Zn-dependent alcohol dehydrogenases, class III
LN02_00636∣LN02Chr01: 2812811-2814321(-)∣462	CDD:2233 19	23.316	193	110	10	79	252	1	174	3.28E-16	73.5	COG02 41	HisB	Histidinol phosphatase and related phosphatases
LN02_00636∣LN02Chr01: 2812811-2814321(-)∣462	CDD:2269 86	23.577	123	76	3	305	411	4	124	9.64E-12	60.6	COG46 39	COG463 9	Predicted kinase
LN02_00828∣LN02Chr01: 3530594-3531462(+)∣204	CDD:2240 25	23.494	166	106	5	21	169	4	165	8.90E-14	64.6	COG11 00	COG110 0	GTPase SAR1 and related small G proteins
LN02_01148∣LN02Chr01: 4544852-4546814(-)∣597	CDD:2252 97	43.182	44	22	3	167	209	107	148	1.42E-07	49	COG24 53	CDC14	Predicted protein-tyrosine phosphatase
LN02_01276∣LN02Chr01: 5127344-5128742(-)∣387	CDD:2278 84	20	135	89	6	46	167	71	199	4.71E-05	42.2	COG55 97	COG559 7	Alpha-N-acetylglucosamine transferase
LN02_01340∣LN02Chr01: 5353960-5356126(+)∣567	CDD:2253 71	19.632	163	125	1	19	181	5	161	4.05E-12	64.9	COG28 14	AraJ	Arabinose efflux permease
LN02_01404∣LN02Chr01: 5604168-5606174(-)∣573	CDD:2231 04	22.098	448	293	9	13	452	1	400	8.56E-51	177	COG00 25	NhaP	NhaP-type Na+/H+ and K+/H+ antiporters
LN02_01724∣LN02Chr01: 6861444-6862072(+)∣181	CDD:2237 53	25.581	129	85	3	40	168	29	146	3.17E-09	50.8	COG06 81	LepB	Signal peptidase I
LN02_02044∣LN02Chr02: 444834-445883(+)∣349	CDD:2249 51	27.405	343	186	17	2	336	9	296	7.71E-40	139	COG20 40	MHT1	Homocysteine/selenocysteine methylase (S-methylmethionine-dependent)
LN02_02108∣LN02Chr02: 636940-640204(+)∣1015	CDD:2241 62	38.952	706	343	10	255	954	15	638	0	695	COG12 41	MCM2	Predicted ATPase involved in replication control, Cdc46/Mcm family
LN02_02236∣LN02Chr02: 1025957-1030588(-)∣1482	CDD:2279 38	16.384	177	144	2	416	589	203	378	8.21E-04	40.7	COG56 51	COG565 1	PPE-repeat proteins
LN02_02364∣LN02Chr02: 1557758-1558965(+)∣301	CDD:2251 68	23.308	133	78	4	187	301	14	140	1.01E-08	50.4	COG22 59	COG225 9	Predicted membrane protein
LN02_02684∣LN02Chr02: 2657742-2659627(+)∣464	CDD:2253 18	27.64	322	170	7	118	437	68	328	9.84E-58	191	COG25 20	COG252 0	Predicted methyltransferase

LN02_02812 ǀ LN02Chr02: 3094022-3096095(-) ǀ 542	CDD:225121	29.268	82	56	2	27	107	22	102	2.37E-04	40.7	COG2211	MelB	Na+/melibiose symporter and related transporters
LN02_02876 ǀ LN02Chr02: 3295351-3297776(-) ǀ 764	CDD:223861	24.907	269	190	4	432	691	7	272	3.46E-32	124	COG0790	COG0790	FOG: TPR repeat, SEL1 subfamily
LN02_02940 ǀ LN02Chr02: 3531993-3533224(+) ǀ 358	CDD:223172	33.929	168	102	4	188	352	16	177	3.90E-36	126	COG0094	RplE	Ribosomal protein L5
LN02_03068 ǀ LN02Chr02: 3981028-3986433(-) ǀ 1746	CDD:223627	27.256	554	352	15	859	1393	333	854	5.92E-68	245	COG0553	HepA	Superfamily II DNA/RNA helicases, SNF2 family
LN02_03132 ǀ LN02Chr02: 4193181-4194427(-) ǀ 235	CDD:226431	22.488	209	135	8	6	212	10	193	5.50E-07	45.6	COG3917	NahD	2-hydroxychromene-2-carboxylate isomerase
LN02_03196 ǀ LN02Chr02: 4458094-4459255(-) ǀ 342	CDD:223285	42.633	319	128	6	24	342	5	268	9.47E-119	341	COG0207	ThyA	Thymidylate synthase
LN02_03260 ǀ LN02Chr02: 4658568-4659618(+) ǀ 245	CDD:224104	28.696	230	122	5	41	244	2	215	2.94E-42	141	COG1183	PssA	Phosphatidylserine synthase
LN02_03324 ǀ LN02Chr02: 4865607-4867636(-) ǀ 551	CDD:227396	61.765	544	187	7	1	542	1	525	0	752	COG5064	SRP1	Karyopherin (importin) alpha
LN02_03644 ǀ LN02Chr03: 103671-107378(+) ǀ 861	CDD:226406	22.424	165	105	6	213	365	694	847	1.51E-04	42.5	COG3889	COG3889	Predicted solute binding protein
LN02_03772 ǀ LN02Chr03: 683987-686356(-) ǀ 735	CDD:226406	19.205	151	105	3	38	171	702	852	5.55E-05	43.7	COG3889	COG3889	Predicted solute binding protein
LN02_03900 ǀ LN02Chr03: 1075184-1076901(+) ǀ 533	CDD:224281	45.359	474	205	9	52	522	13	435	0	542	COG1362	LAP4	Aspartyl aminopeptidase
LN02_04028 ǀ LN02Chr03: 1496280-1498066(-) ǀ 392	CDD:319244	32.308	65	44	0	10	74	10	74	2.41E-14	64.8	COG5272	UBI4	UBI4; linked to 3D-structure.
LN02_04348 ǀ LN02Chr03: 3876580-3880109(+) ǀ 1036	CDD:223599	43.483	959	451	17	79	1032	5	877	0	1068	COG0525	ValS	Valyl-tRNA synthetase
LN02_04476 ǀ LN02Chr03: 4279929-4283007(-) ǀ 918	CDD:227565	38.52	919	535	13	1	914	1	894	0	809	COG5240	SEC21	Vesicle coat complex COPI, gamma subunit
LN02_05052 ǀ LN02Chr04: 458585-459693(+) ǀ 339	CDD:224961	23.148	108	77	4	217	323	31	133	1.42E-08	50.1	COG2050	PaaI	HGG motif-containing thioesterase, possibly involved in aromatic compounds catabolism
LN02_05116 ǀ LN02Chr04: 666418-669455(-) ǀ 817	CDD:224389	28.399	331	176	14	114	419	57	351	4.61E-49	175	COG1472	BglX	Beta-glucosidase-related glycosidases
LN02_05244 ǀ LN02Chr04: 1082139-1084753(-) ǀ 848	CDD:224162	39.91	669	346	9	168	827	1	622	0	684	COG1241	MCM2	Predicted ATPase involved in replication control, Cdc46/Mcm family
LN02_05500 ǀ LN02Chr04: 2113763-2114862(+) ǀ 346	CDD:223377	36.735	196	119	2	67	262	5	195	9.55E-63	198	COG0300	DltE	Short-chain dehydrogenases of various substrate specificities
LN02_05564 ǀ LN02Chr04: 2466458-2469675(-) ǀ 1049	CDD:223641	46.817	974	444	16	68	1041	7	906	0	1291	COG0567	SucA	2-oxoglutarate dehydrogenase complex, dehydrogenase (E1) component, and related enzymes
LN02_05628 ǀ LN02Chr04: 2674834-2676227(+) ǀ 422	CDD:223727	21.374	393	240	12	4	384	1	336	7.22E-28	110	COG0654	UbiH	2-polyprenyl-6-methoxyphenol hydroxylase and related FAD-dependent oxidoreductases
LN02_05692 ǀ LN02Chr04: 2900435-2902673(+) ǀ 706	CDD:227564	30.352	369	215	10	322	680	30	366	1.29E-64	216	COG5239	CCR4	mRNA deadenylase, exonuclease subunit and related nucleases

LN02_05692|LN02Chr04: 2900435-2902673(+)|706	CDD:2272 23	23.005	213	157	3	68	274	10	221	3.22E-14	71.9	COG48 86	COG488 6	Leucine-rich repeat (LRR) protein
LN02_05884|LN02Chr04: 3511089-3511979(-)|229	CDD:2236 78	52.217	203	89	4	32	229	1	200	1.35E-85	249	COG06 05	SodA	Superoxide dismutase
LN02_05948|LN02Chr04: 3793176-3794685(+)|417	CDD:2237 43	15.344	189	145	3	130	309	33	215	7.98E-04	37.8	COG06 71	PgpB	Membrane-associated phospholipid phosphatase
LN02_06076|LN02Chr04: 4186146-4190110(-)|1216	CDD:2237 15	24.834	302	152	7	546	847	108	334	1.60E-31	124	COG06 42	BaeS	Signal transduction histidine kinase
LN02_06076|LN02Chr04: 4186146-4190110(-)|1216	CDD:2238 55	31.092	119	75	3	1088	1205	3	115	2.29E-21	88.4	COG07 84	CheY	FOG: CheY-like receiver
LN02_06140|LN02Chr04: 4397198-4399731(+)|810	CDD:2235 87	27.758	562	321	11	196	752	3	484	8.33E-104	327	COG05 13	SrmB	Superfamily II DNA and RNA helicases
LN02_06396|LN02Chr04: 5250066-5252212(-)|609	CDD:2251 86	32.101	595	336	18	19	608	7	538	9.33E-99	308	COG23 03	BetA	Choline dehydrogenase and related flavoproteins
LN02_06588|LN02Chr05: 22399-26701(-)|1410	CDD:2258 62	27.228	404	221	11	157	503	39	426	1.56E-53	191	COG33 25	ChiA	Chitinase
LN02_06908|LN02Chr05: 1233949-1235332(-)|322	CDD:2236 32	26.984	189	118	4	99	283	11	183	3.12E-27	102	COG05 58	PgsA	Phosphatidylglycerophosphate synthase
LN02_07100|LN02Chr05: 2571263-2572416(-)|335	CDD:2239 59	25.439	228	136	6	18	240	2	200	2.19E-28	107	COG10 28	FabG	Dehydrogenases with different specificities (related to short-chain alcohol dehydrogenases)
LN02_07484|LN02Chr05: 3915932-3919721(-)|1212	CDD:2276 98	33.762	311	162	10	853	1157	61	333	9.26E-54	192	COG54 11	COG541 1	Phosphatidylinositol 5-phosphate phosphatase
LN02_07484|LN02Chr05: 3915932-3919721(-)|1212	CDD:2252 01	17.819	376	265	14	497	858	73	418	1.63E-04	42.4	COG23 19	COG231 9	FOG: WD40 repeat
LN02_07676|LN02Chr05: 4803182-4809163(+)|1895	CDD:2235 34	55.584	394	170	3	451	840	1	393	0	571	COG04 58	CarB	Carbamoylphosphate synthase large subunit (split gene in MJ)
LN02_07676|LN02Chr05: 4803182-4809163(+)|1895	CDD:2235 34	33.505	388	237	7	992	1360	1	386	2.34E-63	219	COG04 58	CarB	Carbamoylphosphate synthase large subunit (split gene in MJ)
LN02_07676|LN02Chr05: 4803182-4809163(+)|1895	CDD:2235 79	45.244	389	181	5	24	412	6	362	1.26E-169	517	COG05 05	CarA	Carbamoylphosphate synthase small subunit
LN02_07676|LN02Chr05: 4803182-4809163(+)|1895	CDD:2236 14	45.307	309	163	5	1590	1893	5	312	3.88E-122	384	COG05 40	PyrB	Aspartate carbamoyltransferase, catalytic chain
LN02_07676|LN02Chr05: 4803182-4809163(+)|1895	CDD:2232 16	31.667	60	31	2	1369	1421	4	60	1.39E-04	43.4	COG01 38	PurH	AICAR transformylase/IMP cyclohydrolase PurH (only IMP cyclohydrolase domain in Aful)
LN02_08060|LN02Chr06: 818426-822135(-)|705	CDD:2264 06	26.364	110	66	4	538	633	739	847	2.09E-04	41.4	COG38 89	COG388 9	Predicted solute binding protein
LN02_08316|LN02Chr06: 2088997-2090615(-)|487	CDD:2239 44	25.854	410	289	9	19	420	36	438	9.10E-75	240	COG10 12	PutA	NAD-dependent aldehyde dehydrogenases
LN02_08380|LN02Chr06: 2395248-2397510(-)|536	CDD:2230 95	29.07	430	199	13	107	526	1	334	3.20E-108	323	COG00 16	PheS	Phenylalanyl-tRNA synthetase alpha subunit
LN02_08508|LN02Chr06: 2821375-2822473(-)|261	CDD:2239 59	35.686	255	145	8	10	254	4	249	5.74E-47	154	COG10 28	FabG	Dehydrogenases with different specificities (related to short-chain alcohol dehydrogenases)

LN02_08572｜LN02Chr06: 3052757-3053450(+)｜142	CDD:2252 25	33.333	87	50	2	40	126	3	81	2.61E-09	48.5	COG23 50	COG235 0	Uncharacterized protein conserved in bacteria
LN02_08700｜LN02Chr06: 3628799-3629497(+)｜232	CDD:2259 87	39.394	66	33	3	50	114	14	73	7.64E-07	46.3	COG34 56	COG345 6	Predicted component of the type VI protein secretion system, contains a FHA domain
LN02_08956｜LN02Chr07: 950655-955400(+)｜703	CDD:2246 99	41.009	456	238	10	206	659	54	480	2.24E-150	443	COG17 85	PhoA	Alkaline phosphatase
LN02_08956｜LN02Chr07: 950655-955400(+)｜703	CDD:2257 83	37.5	40	25	0	26	65	328	367	5.02E-06	46.6	COG32 43	PhaC	Poly(3-hydroxyalkanoate) synthetase
LN02_09276｜LN02Chr07: 2014884-2016938(-)｜563	CDD:2237 43	23.618	199	138	4	43	234	15	206	5.34E-14	68.7	COG06 71	PgpB	Membrane-associated phospholipid phosphatase
LN02_00135｜LN02Chr01: 599002-601296(-)｜509	CDD:2264 06	22.093	172	105	7	220	374	694	853	5.16E-04	39.8	COG38 89	COG388 9	Predicted solute binding protein
LN02_00583｜LN02Chr01: 2613614-2614989(-)｜412	CDD:2279 14	19.231	156	96	5	215	369	131	257	3.52E-11	60.4	COG56 27	MMS21	DNA repair protein MMS21
LN02_00775｜LN02Chr01: 3359861-3363629(+)｜711	CDD:2264 06	23.392	171	103	7	435	594	694	847	9.02E-05	42.9	COG38 89	COG388 9	Predicted solute binding protein
LN02_00967｜LN02Chr01: 3955586-3957281(-)｜491	CDD:2253 71	22.905	179	133	2	71	248	39	213	1.47E-06	47.2	COG28 14	AraJ	Arabinose efflux permease
LN02_01031｜LN02Chr01: 4160546-4162385(-)｜540	CDD:2278 27	41.071	56	32	1	293	347	319	374	1.42E-11	63.1	COG55 40	COG554 0	RING-finger-containing ubiquitin ligase
LN02_01031｜LN02Chr01: 4160546-4162385(-)｜540	CDD:2275 68	28.571	126	61	4	245	356	246	356	2.31E-08	53.4	COG52 43	HRD1	HRD ubiquitin ligase complex, ER membrane component
LN02_01287｜LN02Chr01: 5156095-5157407(-)｜352	CDD:2275 23	38.35	206	120	4	5	208	2	202	6.31E-42	143	COG51 96	ERD2	ER lumen protein retaining receptor
LN02_01479｜LN02Chr01: 5953262-5954307(+)｜307	CDD:2235 60	41.176	85	48	1	16	100	5	87	2.17E-22	93.1	COG04 84	DnaJ	DnaJ-class molecular chaperone with C-terminal Zn finger domain
LN02_01543｜LN02Chr01: 6209543-6212191(+)｜788	CDD:2264 66	51.88	798	343	6	8	770	1	792	0	1029	COG39 57	COG395 7	Phosphoketolase
LN02_01607｜LN02Chr01: 6462234-6465213(+)｜932	CDD:2254 82	28.729	181	125	4	164	342	45	223	1.07E-18	83.4	COG29 30	COG293 0	Uncharacterized conserved protein
LN02_01735｜LN02Chr01: 6885859-6886749(-)｜279	CDD:2235 01	40.976	205	97	8	51	255	3	183	1.83E-57	180	COG04 24	Maf	Nucleotide-binding protein implicated in inhibition of septum formation
LN02_01799｜LN02Chr01: 7061774-7063058(+)｜348	CDD:2240 25	39.259	135	77	2	26	159	1	131	3.41E-33	120	COG11 00	COG110 0	GTPase SAR1 and related small G proteins
LN02_01863｜LN02Chr01: 7247468-7249511(+)｜557	CDD:2274 23	46.12	451	231	8	114	557	6	451	8.54E-176	501	COG50 92	NMT1	N-myristoyl transferase
LN02_01927｜LN02Chr01: 7428234-7429877(-)｜526	CDD:2275 17	35.465	172	110	1	220	390	213	384	2.69E-37	139	COG51 90	FCP1	TFIIF-interacting CTD phosphatases, including NLI-interacting factor
LN02_02247｜LN02Chr02: 1061236-1062659(-)｜442	CDD:2260 42	17.901	162	106	5	159	307	228	375	2.82E-05	43.2	COG35 11	PlcC	Phospholipase C
LN02_02311｜LN02Chr02: 1324090-1328379(+)｜1429	CDD:2264 06	22.652	181	112	7	479	643	694	862	1.08E-04	43.7	COG38 89	COG388 9	Predicted solute binding protein
LN02_02631｜LN02Chr02: 2491100-2491834(+)｜244	CDD:2275 71	33.186	226	141	3	1	226	1	216	1.15E-79	236	COG52 46	PRP11	Splicing factor 3a, subunit 2

LN02_02695|LN02Chr02: 2705582-2706968(-)|360	CDD:2234 53	26.513	347	163	9	82	336	68	414	6.07E-65	216	COG03 76	KatG	Catalase (peroxidase I)
LN02_03079|LN02Chr02: 4025870-4028836(-)|928	CDD:2232 46	24.619	394	263	9	423	809	125	491	1.55E-64	222	COG01 68	TrkG	Trk-type K+ transport systems, membrane components
LN02_03207|LN02Chr02: 4495457-4499273(-)|1234	CDD:2241 36	21.992	241	146	7	869	1099	138	346	1.30E-18	87.3	COG12 15	COG121 5	Glycosyltransferases, probably involved in cell wall biogenesis
LN02_03783|LN02Chr03: 743206-744501(+)|394	CDD:2277 21	24.29	317	169	13	37	298	97	397	1.21E-15	75.6	COG54 34	PGU1	Endopygalactorunase
LN02_03847|LN02Chr03: 921100-923387(+)|618	CDD:2252 01	31.307	329	198	14	95	398	114	439	4.95E-39	146	COG23 19	COG231 9	FOG: WD40 repeat
LN02_03975|LN02Chr03: 1306896-1309794(+)|687	CDD:2243 59	22.667	150	87	6	103	225	97	244	5.73E-16	76.6	COG14 42	RfaJ	Lipopolysaccharide biosynthesis proteins, LPS:glycosyltransferases
LN02_03975|LN02Chr03: 1306896-1309794(+)|687	CDD:2278 84	25	172	118	7	2	162	61	232	1.77E-09	57.2	COG55 97	COG559 7	Alpha-N-acetylglucosamine transferase
LN02_04167|LN02Chr03: 2948302-2950371(-)|663	CDD:2235 00	41.853	626	283	9	21	644	12	558	0	724	COG04 23	GRS1	Glycyl-tRNA synthetase (class II)
LN02_04551|LN02Chr03: 4549301-4552211(+)|648	CDD:2251 61	35.759	481	237	9	87	566	3	412	8.14E-112	340	COG22 52	COG225 2	Xanthine/uracil/vitamin C permease
LN02_04615|LN02Chr03: 4810464-4813688(-)|842	CDD:2238 75	66.491	570	185	3	276	842	2	568	0	1060	COG08 04	UreC	Urea amidohydrolase (urease) alpha subunit
LN02_04615|LN02Chr03: 4810464-4813688(-)|842	CDD:2239 01	54	100	45	1	1	100	1	99	2.31E-43	149	COG08 31	UreA	Urea amidohydrolase (urease) gamma subunit
LN02_04615|LN02Chr03: 4810464-4813688(-)|842	CDD:2239 02	56.79	81	35	0	138	218	3	83	5.70E-38	134	COG08 32	UreB	Urea amidohydrolase (urease) beta subunit
LN02_04871|LN02Chr03: 5833520-5835757(+)|745	CDD:2235 87	26.362	569	331	11	144	712	28	508	1.50E-91	293	COG05 13	SrmB	Superfamily II DNA and RNA helicases
LN02_05063|LN02Chr04: 495123-496456(-)|336	CDD:2274 06	27.419	248	171	2	73	316	22	264	3.33E-40	140	COG50 74	COG507 4	t-SNARE complex subunit, syntaxin
LN02_05255|LN02Chr04: 1118072-1120713(-)|837	CDD:2243 89	30.168	358	218	10	26	367	48	389	4.07E-68	228	COG14 72	BglX	Beta-glucosidase-related glycosidases
LN02_05383|LN02Chr04: 1560364-1563220(+)|866	CDD:2243 22	28.417	278	168	8	69	330	61	323	8.36E-16	78.1	COG14 04	AprE	Subtilisin-like serine proteases
LN02_05575|LN02Chr04: 2504297-2506023(+)|505	CDD:2243 91	21.395	215	128	7	19	224	18	200	3.52E-05	43.1	COG14 74	CDC6	Cdc6-related protein, AAA superfamily ATPase
LN02_05639|LN02Chr04: 2715393-2716745(-)|394	CDD:2233 36	28.406	345	200	15	18	350	1	310	4.82E-60	194	COG02 58	Exo	5'-3' exonuclease (including N-terminal domain of PolI)
LN02_05767|LN02Chr04: 3124079-3124705(+)|208	CDD:2263 89	24.812	133	86	5	29	160	9	128	7.40E-13	60.5	COG38 71	COG387 1	Uncharacterized stress protein (general stress protein 26)
LN02_05831|LN02Chr04: 3349695-3351277(+)|457	CDD:2236 85	24.533	428	288	16	45	451	25	438	1.14E-22	97.1	COG06 12	PqqL	Predicted Zn-dependent peptidases
LN02_06151|LN02Chr04: 4434205-4435725(+)|376	CDD:2278 74	24.786	234	152	7	112	341	10	223	2.52E-25	99.6	COG55 87	COG558 7	Uncharacterized conserved protein
LN02_06407|LN02Chr04: 5290917-5293113(+)|579	CDD:2251 61	36.498	474	229	11	16	488	6	408	6.48E-116	348	COG22 52	COG225 2	Xanthine/uracil/vitamin C permease

LN02_06855|LN02Chr05: 1043671-1045681(+)|446	CDD:223085	22.454	432	224	10	51	438	19	383	2.21E-45	159	COG0006	PepP	Xaa-Pro aminopeptidase
LN02_06919|LN02Chr05: 1377803-1381474(+)|502	CDD:223699	32.105	190	125	4	308	495	208	395	2.55E-49	171	COG0626	MetC	Cystathionine beta-lyases/cystathionine gamma-synthases
LN02_07111|LN02Chr05: 2596951-2598371(+)|397	CDD:223730	21.239	339	214	11	82	396	2	311	1.95E-24	99.6	COG0657	Aes	Esterase/lipase
LN02_07367|LN02Chr05: 3462315-3464790(-)|665	CDD:226739	35.484	465	267	14	17	480	25	457	1.36E-91	289	COG4289	COG4289	Uncharacterized protein conserved in bacteria
LN02_07495|LN02Chr05: 3966883-3968557(-)|450	CDD:224117	16.308	325	248	4	39	360	661	964	4.97E-08	52.4	COG1196	Smc	Chromosome segregation ATPases
LN02_07815|LN02Chr05: 5373841-5375445(-)|462	CDD:227170	21.402	271	161	15	63	320	44	275	7.72E-09	54.2	COG4833	COG4833	Predicted glycosyl hydrolase
LN02_07943|LN02Chr05: 5750815-5752107(-)|385	CDD:224322	35.636	275	141	9	105	348	111	380	4.02E-27	109	COG1404	AprE	Subtilisin-like serine proteases
LN02_08071|LN02Chr06: 1023111-1024340(+)|409	CDD:223296	32.62	187	99	4	147	328	1	165	2.25E-34	123	COG0218	COG0218	Predicted GTPase
LN02_08391|LN02Chr06: 2437795-2439664(+)|528	CDD:225035	20.628	446	274	12	53	488	16	391	2.00E-37	139	COG2124	CypX	Cytochrome P450
LN02_08455|LN02Chr06: 2665998-2666944(+)|219	CDD:223275	30.573	157	88	5	11	167	1	136	1.81E-46	148	COG0197	RplP	Ribosomal protein L16/L10E
LN02_08519|LN02Chr06: 2859899-2861645(+)|388	CDD:223137	37.54	309	175	8	65	368	5	300	3.06E-115	336	COG0059	IlvC	Ketol-acid reductoisomerase
LN02_08839|LN02Chr07: 502462-505074(-)|870	CDD:225661	22.034	236	169	7	592	820	210	437	3.15E-12	66.7	COG3119	AslA	Arylsulfatase A and related enzymes
LN02_08839|LN02Chr07: 502462-505074(-)|870	CDD:225105	19.417	309	165	16	421	706	237	484	2.02E-05	45.1	COG2194	COG2194	Predicted membrane-associated, metal-dependent hydrolase
LN02_08903|LN02Chr07: 750165-750561(+)|91	CDD:224869	43.243	74	40	1	14	87	8	79	7.16E-20	73.5	COG1958	LSM1	Small nuclear ribonucleoprotein (snRNP) homolog
LN02_09159|LN02Chr07: 1601818-1603101(+)|380	CDD:227469	41.791	335	167	7	30	363	20	327	1.04E-102	304	COG5140	UFD1	Ubiquitin fusion-degradation protein
LN02_09223|LN02Chr07: 1794417-1798552(-)|1217	CDD:224117	21.466	191	150	0	489	679	694	884	1.69E-15	79	COG1196	Smc	Chromosome segregation ATPases
LN02_00061|LN02Chr01: 367209-368483(+)|390	CDD:224025	20.853	211	148	6	15	218	6	204	6.73E-24	95.8	COG1100	COG1100	GTPase SAR1 and related small G proteins
LN02_00125|LN02Chr01: 556947-557645(+)|183	CDD:227229	33.824	68	43	2	61	127	2	68	2.22E-04	35.6	COG4892	COG4892	Predicted heme/steroid binding protein
LN02_00317|LN02Chr01: 1762592-1765176(+)|806	CDD:223738	22.927	205	115	6	377	560	4	186	2.49E-08	52.5	COG0666	Arp	FOG: Ankyrin repeat
LN02_00381|LN02Chr01: 1961841-1963008(+)|267	CDD:223985	30.734	218	127	6	31	248	1	194	3.62E-44	145	COG1057	NadD	Nicotinic acid mononucleotide adenylyltransferase
LN02_00445|LN02Chr01: 2136967-2138229(+)|390	CDD:223255	32.547	212	130	3	145	356	10	208	1.66E-60	192	COG0177	Nth	Predicted EndoIII-related endonuclease
LN02_00509|LN02Chr01: 2317187-2319225(+)|550	CDD:225371	23.226	155	98	5	78	226	51	190	8.43E-04	38.7	COG2814	AraJ	Arabinose efflux permease

LN02_00701∣LN02Chr01: 3091638-3093386(+)∣582	CDD:227270	25.483	259	134	14	333	569	326	547	3.71E-17	82.5	COG4934	COG4934	Predicted protease
LN02_00765∣LN02Chr01: 3290021-3291525(-)∣475	CDD:227390	38.506	348	168	9	27	372	8	311	1.13E-97	295	COG5057	LAG1	Phosphotyrosyl phosphatase activator
LN02_00893∣LN02Chr01: 3701708-3702787(-)∣212	CDD:226798	57.522	113	48	0	15	127	1	113	1.28E-35	119	COG4352	RPL13	Ribosomal protein L13E
LN02_01405∣LN02Chr01: 5608509-5609843(-)∣382	CDD:223405	35.333	150	85	5	234	380	4	144	1.20E-35	125	COG0328	RnhA	Ribonuclease HI
LN02_01405∣LN02Chr01: 5608509-5609843(-)∣382	CDD:225878	43.75	48	27	0	79	126	6	53	1.24E-10	58	COG3341	COG3341	Predicted double-stranded RNA/RNA-DNA hybrid binding protein
LN02_01405∣LN02Chr01: 5608509-5609843(-)∣382	CDD:225878	48.889	45	23	0	138	182	7	51	4.02E-10	56.5	COG3341	COG3341	Predicted double-stranded RNA/RNA-DNA hybrid binding protein
LN02_01853∣LN02Chr01: 7222483-7224172(-)∣458	CDD:227829	19.565	138	108	3	105	240	99	235	7.10E-04	38.5	COG5542	COG5542	Predicted integral membrane protein
LN02_02109∣LN02Chr02: 640846-643446(-)∣783	CDD:227369	49.32	515	228	7	1	504	1	493	0	648	COG5036	COG5036	SPX domain-containing protein involved in vacuolar polyphosphate accumulation
LN02_02109∣LN02Chr02: 640846-643446(-)∣783	CDD:227589	44.144	111	59	1	665	775	13	120	4.92E-37	132	COG5264	VTC1	Vacuolar transporter chaperone
LN02_02173∣LN02Chr02: 818881-820527(+)∣548	CDD:223232	28.305	537	277	17	34	530	3	471	4.62E-80	256	COG0154	GatA	Asp-tRNAAsn/Glu-tRNAGln amidotransferase A subunit and related amidases
LN02_02237∣LN02Chr02: 1031406-1032356(+)∣271	CDD:223711	33.175	211	138	1	4	211	5	215	2.30E-63	196	COG0638	PRE1	20S proteasome, alpha and beta subunits
LN02_02429∣LN02Chr02: 1789709-1790569(-)∣286	CDD:224655	28.315	279	156	11	10	284	3	241	1.54E-43	147	COG1741	COG1741	Pirin-related protein
LN02_03005∣LN02Chr02: 3733591-3735988(+)∣710	CDD:227637	30.303	594	321	15	19	605	17	524	5.05E-127	386	COG5329	COG5329	Phosphoinositide polyphosphatase (Sac family)
LN02_03133∣LN02Chr02: 4216522-4221534(-)∣1619	CDD:223540	43.554	287	135	4	543	822	220	486	1.54E-75	257	COG0464	SpoVK	ATPases of the AAA+ class
LN02_03197∣LN02Chr02: 4459798-4460584(+)∣105	CDD:227384	67.021	94	31	0	8	101	4	97	9.99E-28	94.6	COG5051	RPL36A	Ribosomal protein L36E
LN02_03837∣LN02Chr03: 898261-899501(-)∣343	CDD:223959	28.631	241	155	6	34	270	1	228	6.29E-36	128	COG1028	FabG	Dehydrogenases with different specificities (related to short-chain alcohol dehydrogenases)
LN02_04093∣LN02Chr03: 2290963-2296307(-)∣820	CDD:226406	29.293	99	65	1	605	698	764	862	2.40E-04	41.8	COG3889	COG3889	Predicted solute binding protein
LN02_04157∣LN02Chr03: 2896699-2898068(+)∣313	CDD:223617	31.381	239	139	8	69	307	10	223	2.37E-44	149	COG0543	UbiB	2-polyprenylphenol hydroxylase and related flavodoxin oxidoreductases
LN02_04285∣LN02Chr03: 3670675-3671289(+)∣165	CDD:226826	27.273	55	34	1	91	145	10	58	3.70E-07	42.5	COG4391	COG4391	Uncharacterized protein conserved in bacteria
LN02_04349∣LN02Chr03: 3880402-3882609(-)∣735	CDD:223587	36.087	460	254	8	255	708	1	426	8.40E-127	384	COG0513	SrmB	Superfamily II DNA and RNA helicases
LN02_04477∣LN02Chr03: 4283720-4284977(+)∣375	CDD:227677	26.78	295	167	15	75	366	11	259	2.26E-32	119	COG5387	COG5387	Chaperone required for the assembly of the mitochondrial F1-ATPase

LN02_04797|LN02Chr03: 5434580-5435985(+)|420	CDD:227596	29.032	93	48	4	325	417	4012	4086	1.79E-04	41.1	COG5271	MDN1	AAA ATPase containing von Willebrand factor type A (vWA) domain
LN02_04925|LN02Chr03: 5980865-5987312(+)|2069	CDD:224037	29.711	589	351	17	1268	1850	226	757	2.48E-84	292	COG1112	COG1112	Superfamily I DNA and RNA helicases and helicase subunits
LN02_04989|LN02Chr04: 286424-288839(-)|739	CDD:224541	33.853	641	337	13	71	710	4	558	0	542	COG1626	TreA	Neutral trehalase
LN02_05053|LN02Chr04: 460013-461028(+)|246	CDD:223603	59	200	73	2	43	241	2	193	2.23E-106	303	COG0529	CysC	Adenylylsulfate kinase and related kinases
LN02_05181|LN02Chr04: 879870-881885(+)|671	CDD:227861	26.984	63	32	1	195	257	218	266	1.93E-07	49.9	COG5574	PEX10	RING-finger-containing E3 ubiquitin ligase
LN02_05309|LN02Chr04: 1322630-1326379(+)|1249	CDD:227358	24.812	133	82	4	719	842	295	418	5.60E-08	54	COG5025	COG5025	Transcription factor of the Forkhead/HNF3 family
LN02_05501|LN02Chr04: 2115524-2117301(+)|541	CDD:223202	31.743	482	265	17	69	540	1	428	2.47E-125	371	COG0124	HisS	Histidyl-tRNA synthetase
LN02_05629|LN02Chr04: 2676992-2678302(-)|436	CDD:227370	34.066	182	82	2	1	170	4	159	6.13E-42	146	COG5037	TOS9	Gluconate transport-inducing protein
LN02_05757|LN02Chr04: 3094381-3095892(-)|421	CDD:227362	27.39	387	205	8	7	393	25	335	3.92E-39	141	COG5029	CAL1	Prenyltransferase, beta subunit
LN02_05821|LN02Chr04: 3318462-3319429(-)|299	CDD:223766	50	80	38	1	198	275	7	86	1.09E-30	108	COG0694	COG0694	Thioredoxin-like proteins and domains
LN02_05885|LN02Chr04: 3512624-3513230(+)|178	CDD:227467	31.395	172	109	3	1	171	1	164	2.11E-39	129	COG5138	COG5138	Uncharacterized conserved protein
LN02_05949|LN02Chr04: 3796294-3797736(-)|424	CDD:223589	29.31	290	178	9	19	287	2	285	2.92E-57	190	COG0515	SPS1	Serine/threonine protein kinase
LN02_06013|LN02Chr04: 4012887-4014780(+)|549	CDD:223605	22.075	453	330	6	56	508	4	433	1.86E-36	138	COG0531	PotE	Amino acid transporters
LN02_06141|LN02Chr04: 4400442-4402981(-)|683	CDD:224157	24.46	139	94	4	10	143	32	164	1.29E-13	70.5	COG1236	YSH1	Predicted exonuclease of the beta-lactamase fold involved in RNA processing
LN02_06397|LN02Chr04: 5256206-5257498(+)|368	CDD:225288	30	70	48	1	106	174	395	464	3.23E-05	42.8	COG2433	COG2433	Uncharacterized conserved protein
LN02_06461|LN02Chr04: 5455600-5458217(-)|854	CDD:223252	29.724	434	250	12	431	834	16	424	4.67E-60	208	COG0174	GlnA	Glutamine synthetase
LN02_06461|LN02Chr04: 5455600-5458217(-)|854	CDD:225070	18.593	199	123	8	197	372	106	288	3.64E-07	49.7	COG2159	COG2159	Predicted metal-dependent hydrolase of the TIM-barrel fold
LN02_06589|LN02Chr05: 27937-32873(+)|1596	CDD:227721	47.059	51	18	1	261	311	84	125	9.22E-07	50.2	COG5434	PGU1	Endopygalactorunase
LN02_06589|LN02Chr05: 27937-32873(+)|1596	CDD:227721	42.222	45	25	1	622	665	80	124	9.98E-05	43.6	COG5434	PGU1	Endopygalactorunase
LN02_06845|LN02Chr05: 1013922-1014911(-)|314	CDD:223483	24.852	169	97	9	128	284	11	161	5.86E-14	66.7	COG0406	phoE	Broad specificity phosphatase PhoE and related phosphatases
LN02_06973|LN02Chr05: 1836157-1837631(+)|416	CDD:223286	33.133	332	203	6	78	392	14	343	8.52E-112	329	COG0208	NrdF	Ribonucleotide reductase, beta subunit
LN02_07293|LN02Chr05: 3233350-3236701(+)|1005	CDD:223957	19.529	1019	656	37	10	937	3	948	1.61E-108	357	COG1026	COG1026	Predicted Zn-dependent peptidases, insulinase-like

LN02_07357|LN02Chr05: 3435367-3436778(-)|335	CDD:2231 60	42.818	369	153	7	1	327	2	354	1.10E-138	395	COG00 82	AroC	Chorismate synthase
LN02_07549|LN02Chr05: 4132002-4134597(-)|802	CDD:2232 93	30.272	588	247	13	3	589	1	426	6.45E-141	421	COG02 15	CysS	Cysteinyl-tRNA synthetase
LN02_07677|LN02Chr05: 4809728-4811026(+)|408	CDD:2233 37	42.52	254	96	7	156	408	10	214	4.32E-75	230	COG02 59	PdxH	Pyridoxamine-phosphate oxidase
LN02_07741|LN02Chr05: 5060651-5061255(-)|169	CDD:2234 63	60.123	163	62	1	5	167	3	162	8.25E-93	264	COG03 86	BtuE	Glutathione peroxidase
LN02_07805|LN02Chr05: 5320160-5322516(-)|693	CDD:2235 33	19.331	269	208	4	253	512	1	269	5.50E-08	52.2	COG04 57	NrfG	FOG: TPR repeat
LN02_08253|LN02Chr06: 1884656-1885863(+)|367	CDD:2261 45	21.585	366	196	15	3	367	4	279	7.27E-23	94	COG36 18	COG361 8	Predicted metal-dependent hydrolase of the TIM-barrel fold
LN02_08381|LN02Chr06: 2398030-2399895(-)|621	CDD:2231 42	35.115	524	284	17	98	615	6	479	6.77E-150	438	COG00 64	GatB	Asp-tRNAAsn/Glu-tRNAGln amidotransferase B subunit (PET112 homolog)
LN02_08445|LN02Chr06: 2636660-2638955(+)|454	CDD:2237 04	24.573	293	175	8	19	304	6	259	1.46E-55	182	COG06 31	PTC1	Serine/threonine protein phosphatase
LN02_08509|LN02Chr06: 2823327-2824372(+)|287	CDD:2235 74	25	128	75	3	79	204	48	156	6.32E-07	46.8	COG05 00	SmtA	SAM-dependent methyltransferases
LN02_08957|LN02Chr07: 957267-960188(-)|453	CDD:2238 61	25.98	204	140	4	202	401	31	227	3.56E-16	75.9	COG07 90	COG079 0	FOG: TPR repeat, SEL1 subfamily
LN02_09021|LN02Chr07: 1161585-1163856(+)|675	CDD:2237 33	24.769	541	287	9	89	627	13	435	5.78E-88	281	COG06 61	AarF	Predicted unusual protein kinase
LN02_09149|LN02Chr07: 1567555-1568069(-)|152	CDD:2237 96	35.366	82	50	1	8	89	115	193	5.26E-17	73.1	COG07 24	COG072 4	RNA-binding proteins (RRM domain)
LN02_09277|LN02Chr07: 2017908-2019251(+)|274	CDD:2239 59	34.866	261	149	8	22	272	2	251	3.63E-51	165	COG10 28	FabG	Dehydrogenases with different specificities (related to short-chain alcohol dehydrogenases)
LN02_00062|LN02Chr01: 368742-369064(-)|70	CDD:2249 07	40	45	26	1	27	70	5	49	6.09E-10	46.6	COG19 96	RPC10	DNA-directed RNA polymerase, subunit RPC10 (contains C4-type Zn-finger)
LN02_00126|LN02Chr01: 558248-561041(-)|908	CDD:2251 43	26.842	190	91	9	525	691	186	350	1.81E-09	58	COG22 34	Iap	Predicted aminopeptidases
LN02_00190|LN02Chr01: 881420-882511(+)|254	CDD:2243 12	37.85	214	122	4	7	213	2	211	2.67E-61	189	COG13 94	NtpD	Archaeal/vacuolar-type H+-ATPase subunit D
LN02_00254|LN02Chr01: 1467232-1469150(+)|566	CDD:2260 68	38.889	468	243	9	91	555	2	429	3.03E-159	459	COG35 38	COG353 8	Uncharacterized conserved protein
LN02_00318|LN02Chr01: 1766078-1768279(-)|641	CDD:2244 95	24.051	79	42	2	564	631	61	132	8.73E-04	38.5	COG15 79	COG157 9	Zn-ribbon protein, possibly nucleic acid-binding
LN02_00382|LN02Chr01: 1965673-1967808(+)|711	CDD:2274 14	23.78	164	110	4	334	490	20	175	2.87E-17	77.6	COG50 82	AIR1	Arginine methyltransferase-interacting protein, contains RING Zn-finger
LN02_00446|LN02Chr01: 2139377-2139669(-)|72	CDD:2261 10	34.328	67	41	2	1	64	88	154	7.96E-11	51.4	COG35 82	COG358 2	Predicted nucleic acid binding protein containing the AN1-type Zn-finger
LN02_00510|LN02Chr01: 2319887-2321362(-)|491	CDD:2237 69	22.083	240	154	4	223	461	80	287	4.50E-10	57.6	COG06 97	RhaT	Permeases of the drug/metabolite transporter (DMT) superfamily

LN02_00574	LN02Chr01: 2532665-2533928(-)	358	CDD:226751	27.815	302	196	10	33	330	26	309	2.05E-32	120	COG4301	COG4301	Uncharacterized conserved protein
LN02_00702	LN02Chr01: 3094633-3096062(+)	452	CDD:223594	23.602	322	216	11	46	357	35	336	2.18E-34	130	COG0520	csdA	Selenocysteine lyase/Cysteine desulfurase
LN02_00830	LN02Chr01: 3533452-3534874(+)	435	CDD:223538	38.106	433	147	7	1	432	1	313	8.48E-131	376	COG0462	PrsA	Phosphoribosylpyrophosphate synthetase
LN02_01022	LN02Chr01: 4132661-4134477(-)	534	CDD:223596	48.333	60	31	0	149	208	85	144	9.05E-20	84.7	COG0522	RpsD	Ribosomal protein S4 and related proteins
LN02_01022	LN02Chr01: 4132661-4134477(-)	534	CDD:225177	15.612	237	176	7	222	448	210	432	6.92E-04	39	COG2268	COG2268	Uncharacterized protein conserved in bacteria
LN02_01086	LN02Chr01: 4373144-4376495(-)	1021	CDD:223550	34.867	978	520	20	55	1007	32	917	0	739	COG0474	MgtA	Cation transport ATPase
LN02_01150	LN02Chr01: 4551071-4552803(-)	476	CDD:225201	29.117	419	252	12	64	474	16	397	4.09E-50	174	COG2319	COG2319	FOG: WD40 repeat
LN02_01278	LN02Chr01: 5132125-5133772(+)	503	CDD:226363	32.051	468	238	11	32	498	12	400	9.67E-107	321	COG3844	COG3844	Kynureninase
LN02_01406	LN02Chr01: 5611157-5612547(-)	430	CDD:223663	25.316	79	48	2	342	418	74	143	1.07E-09	53.9	COG0590	CumB	Cytosine/adenosine deaminases
LN02_01470	LN02Chr01: 5924916-5928229(-)	997	CDD:227663	16.816	446	210	11	604	992	398	739	7.19E-14	72.7	COG5369	COG5369	Uncharacterized conserved protein
LN02_01854	LN02Chr01: 7224466-7225445(+)	260	CDD:225089	23.944	142	90	4	29	169	5	129	1.96E-11	58.9	COG2178	COG2178	Predicted RNA-binding protein of the translin family
LN02_01918	LN02Chr01: 7408051-7408743(+)	230	CDD:226237	28.774	212	141	4	9	217	8	212	3.90E-12	60.5	COG3714	COG3714	Predicted membrane protein
LN02_02174	LN02Chr02: 820840-821964(-)	281	CDD:225174	22.989	87	58	4	76	157	273	355	1.51E-05	42.7	COG2265	TrmA	SAM-dependent methyltransferases related to tRNA (uracil-5-)-methyltransferase
LN02_02238	LN02Chr02: 1033239-1035637(-)	746	CDD:223861	19.76	167	103	4	543	703	80	221	2.40E-15	74.3	COG0790	COG0790	FOG: TPR repeat, SEL1 subfamily
LN02_02366	LN02Chr02: 1563019-1565123(-)	642	CDD:225297	30.159	189	116	4	211	398	1	174	2.65E-35	128	COG2453	CDC14	Predicted protein-tyrosine phosphatase
LN02_02494	LN02Chr02: 2008379-2009915(+)	482	CDD:225182	24.638	138	86	5	244	379	104	225	1.48E-11	62.9	COG2273	SKN1	Beta-glucanase/Beta-glucan synthetase
LN02_02558	LN02Chr02: 2234826-2238169(-)	1066	CDD:224212	23.547	688	378	20	1	673	1	555	9.23E-73	249	COG1293	COG1293	Predicted RNA-binding protein homologous to eukaryotic snRNP
LN02_02622	LN02Chr02: 2453233-2454153(-)	269	CDD:223767	33.333	84	52	3	162	245	1	80	1.91E-15	67	COG0695	GrxC	Glutaredoxin and related proteins
LN02_02686	LN02Chr02: 2673588-2676939(-)	1063	CDD:223631	22.061	553	283	20	533	1046	256	699	2.82E-29	122	COG0557	VacB	Exoribonuclease R
LN02_02878	LN02Chr02: 3302234-3303409(+)	391	CDD:223149	29.487	156	91	5	236	391	10	146	3.38E-24	94.4	COG0071	IbpA	Molecular chaperone (small heat shock protein)
LN02_02942	LN02Chr02: 3536699-3538499(+)	375	CDD:227602	47.63	443	164	11	1	375	2	444	5.53E-175	491	COG5277	COG5277	Actin and related proteins
LN02_03070	LN02Chr02: 3989899-3990545(-)	138	CDD:223702	27.451	102	65	3	31	125	5	104	1.55E-11	55.9	COG0629	Ssb	Single-stranded DNA-binding protein

LN02_03326 | LN02Chr02: 4871278-4875044(-) | 1178	CDD:227824	25.527	427	283	10	81	495	29	432	4.21E-51	190	COG5537	IRR1	Cohesin
LN02_03710 | LN02Chr03: 380796-383211(+) | 589	CDD:224322	27.734	256	146	10	174	403	135	377	9.68E-14	70.7	COG1404	AprE	Subtilisin-like serine proteases
LN02_04030 | LN02Chr03: 1505910-1507847(+) | 618	CDD:223492	27.605	547	301	16	6	547	4	460	1.35E-70	232	COG0415	PhrB	Deoxyribodipyrimidine photolyase
LN02_04094 | LN02Chr03: 2314758-2315336(+) | 149	CDD:224947	30.337	89	51	3	44	132	9	86	4.74E-09	47.7	COG2036	HHT1	Histones H3 and H4
LN02_04158 | LN02Chr03: 2898673-2902340(-) | 1194	CDD:223533	24.852	169	115	2	748	912	111	271	8.65E-06	46	COG0457	NrfG	FOG: TPR repeat
LN02_04158 | LN02Chr03: 2898673-2902340(-) | 1194	CDD:226809	21.788	179	109	4	853	1031	122	269	5.06E-05	44.2	COG4372	COG4372	Uncharacterized protein conserved in bacteria with the myosin-like domain
LN02_04222 | LN02Chr03: 3413120-3414451(+) | 405	CDD:224717	36.446	332	192	6	1	318	2	328	1.31E-91	278	COG1804	CaiB	Predicted acyl-CoA transferases/carnitine dehydratase
LN02_04286 | LN02Chr03: 3672281-3673209(+) | 202	CDD:223509	40.278	144	78	4	57	199	1	137	1.48E-57	175	COG0432	COG0432	Uncharacterized conserved protein
LN02_04478 | LN02Chr03: 4286285-4287020(+) | 218	CDD:223730	21.717	198	111	6	37	210	74	251	9.64E-07	45.3	COG0657	Aes	Esterase/lipase
LN02_04798 | LN02Chr03: 5436221-5436544(-) | 107	CDD:223377	27.778	108	59	6	5	107	141	234	7.88E-06	39.9	COG0300	DltE	Short-chain dehydrogenases of various substrate specificities
LN02_04862 | LN02Chr03: 5810031-5811701(+) | 437	CDD:224365	49.875	399	193	4	35	433	5	396	0	559	COG1448	TyrB	Aspartate/tyrosine/aromatic aminotransferase
LN02_05054 | LN02Chr04: 461327-462841(-) | 393	CDD:227456	30	360	247	2	3	362	4	358	4.12E-91	276	COG5127	COG5127	Vacuolar H+-ATPase V1 sector, subunit C
LN02_05118 | LN02Chr04: 677691-679436(+) | 291	CDD:223498	42.756	283	160	1	9	291	1	281	6.67E-121	345	COG0421	SpeE	Spermidine synthase
LN02_05182 | LN02Chr04: 882664-885018(+) | 784	CDD:227674	28.864	537	263	15	241	766	123	551	1.02E-65	226	COG5384	Mpp10	U3 small nucleolar ribonucleoprotein component
LN02_05246 | LN02Chr04: 1091164-1093208(-) | 619	CDD:226148	41.558	77	31	4	18	94	10	72	1.41E-07	51.1	COG3621	COG3621	Patatin
LN02_05374 | LN02Chr04: 1529767-1531387(+) | 445	CDD:223971	23.99	396	243	12	1	396	1	338	1.11E-46	161	COG1041	COG1041	Predicted DNA modification methylase
LN02_05502 | LN02Chr04: 2118021-2122497(+) | 1424	CDD:227624	27.742	1258	801	25	108	1327	18	1205	0	645	COG5308	NUP170	Nuclear pore complex subunit
LN02_05566 | LN02Chr04: 2473280-2476297(-) | 1005	CDD:223540	43.636	220	116	5	693	907	239	455	5.28E-59	208	COG0464	SpoVK	ATPases of the AAA+ class
LN02_05694 | LN02Chr04: 2905787-2907220(-) | 477	CDD:224149	28.193	415	264	14	58	462	13	403	2.99E-69	223	COG1228	HutI	Imidazolonepropionase and related amidohydrolases
LN02_05950 | LN02Chr04: 3798914-3799761(-) | 214	CDD:224107	22.959	196	111	8	37	199	50	238	2.41E-13	63.8	COG1186	PrfB	Protein chain release factor B
LN02_06078 | LN02Chr04: 4193852-4195260(-) | 446	CDD:227594	40.885	384	217	6	60	442	1	375	6.59E-91	278	COG5269	ZUO1	Ribosome-associated chaperone zuotin
LN02_06142 | LN02Chr04: 4403924-4406087(-) | 646	CDD:225748	32.323	297	167	8	65	349	44	318	8.60E-42	151	COG3207	DIT1	Pyoverdine/dityrosine biosynthesis protein

LN02_06142∣LN02Chr04:4403924-4406087(-)∣646	CDD:225086	22.989	174	112	6	488	646	111	277	7.49E-13	66.6	COG2175	TauD	Probable taurine catabolism dioxygenase
LN02_06462∣LN02Chr04:5458983-5459825(+)∣280	CDD:225796	64.045	267	90	3	10	276	3	263	1.56E-164	454	COG3257	GlxB	Uncharacterized protein, possibly involved in glyoxylate utilization
LN02_06782∣LN02Chr05:804049-806696(+)∣524	CDD:226275	24.299	214	124	6	317	523	84	266	1.10E-09	56.3	COG3752	COG3752	Steroid 5-alpha reductase family enzyme
LN02_06846∣LN02Chr05:1015320-1016820(+)∣458	CDD:224805	24.506	253	143	11	175	423	71	279	9.91E-17	77.7	COG1893	ApbA	Ketopantoate reductase
LN02_07102∣LN02Chr05:2575383-2580259(+)∣711	CDD:224399	23.795	332	172	14	247	564	30	294	4.57E-35	132	COG1482	ManA	Phosphomannose isomerase
LN02_07230∣LN02Chr05:3037859-3038946(+)∣268	CDD:223555	51.931	233	107	4	34	266	1	228	3.08E-117	333	COG0479	FrdB	Succinate dehydrogenase/fumarate reductase, Fe-S protein subunit
LN02_07294∣LN02Chr05:3239877-3241551(-)∣450	CDD:224117	16.308	325	248	4	39	360	661	964	4.97E-08	52.4	COG1196	Smc	Chromosome segregation ATPases
LN02_07422∣LN02Chr05:3749152-3750047(-)∣183	CDD:224020	24.339	189	121	3	1	180	1	176	9.03E-29	102	COG1095	RPB7	DNA-directed RNA polymerase, subunit E'
LN02_07550∣LN02Chr05:4135247-4136728(+)∣493	CDD:225317	26.78	295	135	8	54	347	24	238	5.83E-51	171	COG2519	GCD14	tRNA(1-methyladenosine) methyltransferase and related methyltransferases
LN02_07742∣LN02Chr05:5062102-5063589(+)∣421	CDD:223737	21.934	424	259	17	13	419	13	381	1.69E-18	83.8	COG0665	DadA	Glycine/D-amino acid oxidases (deaminating)
LN02_07934∣LN02Chr05:5727213-5728970(-)∣528	CDD:223615	40.265	452	261	6	2	448	1	448	1.02E-174	497	COG0541	Ffh	Signal recognition particle GTPase
LN02_08126∣LN02Chr06:1346562-1347479(+)∣305	CDD:319244	70.27	74	21	1	78	150	1	74	3.18E-22	85.6	COG5272	UBI4	UBI4; linked to 3D-structure.
LN02_08126∣LN02Chr06:1346562-1347479(+)∣305	CDD:319244	70.27	74	21	1	2	74	1	74	4.87E-22	84.8	COG5272	UBI4	UBI4; linked to 3D-structure.
LN02_08126∣LN02Chr06:1346562-1347479(+)∣305	CDD:319244	70.27	74	21	1	154	226	1	74	4.87E-22	84.8	COG5272	UBI4	UBI4; linked to 3D-structure.
LN02_08126∣LN02Chr06:1346562-1347479(+)∣305	CDD:319244	70.27	74	21	1	230	302	1	74	4.87E-22	84.8	COG5272	UBI4	UBI4; linked to 3D-structure.
LN02_08254∣LN02Chr06:1886536-1888797(-)∣753	CDD:224117	19.231	286	194	4	345	630	237	485	1.86E-05	45.1	COG1196	Smc	Chromosome segregation ATPases
LN02_08318∣LN02Chr06:2093615-2094394(+)∣209	CDD:227350	42.188	64	33	1	102	161	68	131	1.07E-11	57.9	COG5017	COG5017	Uncharacterized conserved protein
LN02_08510∣LN02Chr06:2824552-2825502(-)∣316	CDD:227826	17.957	323	234	10	3	313	2	305	9.14E-16	73.4	COG5539	COG5539	Predicted cysteine protease (OTU family)
LN02_08574∣LN02Chr06:3057306-3058148(+)∣109	CDD:224969	39.64	111	57	3	6	109	2	109	4.59E-11	52.4	COG2058	RPP1A	Ribosomal protein L12E/L44/L45/RPP1/RPP2
LN02_08638∣LN02Chr06:3237750-3238342(-)∣180	CDD:223532	21.472	163	101	5	6	163	14	154	5.16E-07	44.6	COG0456	RimI	Acetyltransferases
LN02_08766∣LN02Chr07:283607-284539(+)∣310	CDD:225462	29.577	71	46	2	7	76	3	70	4.53E-04	37.7	COG2910	COG2910	Putative NADH-flavin reductase
LN02_08830∣LN02Chr07:476821-478290(-)∣489	CDD:223317	31.897	116	70	4	365	473	4	117	2.00E-14	66.8	COG0239	CrcB	Integral membrane protein possibly involved in chromosome condensation

LN02_08830|LN02Chr07: 476821-478290(-)|489	CDD:223317	24.324	37	28	0	240	276	66	102	3.27E-05	40.6	COG0239	CrcB	Integral membrane protein possibly involved in chromosome condensation
LN02_09150|LN02Chr07: 1568635-1572550(+)|1260	CDD:225201	24.919	309	204	10	32	332	72	360	1.23E-27	115	COG2319	COG2319	FOG: WD40 repeat
LN02_09150|LN02Chr07: 1568635-1572550(+)|1260	CDD:227665	27.885	104	66	3	753	848	18	120	2.52E-04	42.1	COG5373	COG5373	Predicted membrane protein
LN02_09278|LN02Chr07: 2019305-2022099(-)|727	CDD:223552	40.994	161	93	2	119	278	29	188	5.14E-49	170	COG0476	ThiF	Dinucleotide-utilizing enzymes involved in molybdopterin and thiamine biosynthesis family 2
LN02_00063|LN02Chr01: 369673-370653(-)|296	CDD:227923	27.437	277	173	14	27	288	6	269	3.33E-36	128	COG5636	COG5636	Uncharacterized conserved protein, contains Zn-ribbon-like motif
LN02_00191|LN02Chr01: 882625-883481(-)|245	CDD:223796	28.713	101	65	2	6	103	111	207	2.46E-11	59.6	COG0724	COG0724	RNA-binding proteins (RRM domain)
LN02_00319|LN02Chr01: 1769086-1770064(+)|257	CDD:224562	33.495	206	132	2	13	218	3	203	9.24E-56	175	COG1648	CysG	Siroheme synthase (precorrin-2 oxidase/ferrochelatase domain)
LN02_00447|LN02Chr01: 2143760-2145916(+)|645	CDD:224871	18.931	449	267	13	83	520	30	392	4.43E-34	131	COG1960	CaiA	Acyl-CoA dehydrogenases
LN02_00511|LN02Chr01: 2322478-2324269(-)|541	CDD:223690	23.593	462	265	12	57	512	28	407	3.53E-59	199	COG0617	PcnB	tRNA nucleotidyltransferase/poly(A) polymerase
LN02_00767|LN02Chr01: 3295423-3299172(+)|1120	CDD:224117	19.804	818	576	21	243	1027	167	937	8.48E-23	102	COG1196	Smc	Chromosome segregation ATPases
LN02_00831|LN02Chr01: 3536430-3537242(-)|229	CDD:227778	29.518	166	105	5	74	229	41	204	1.20E-07	47.5	COG5491	VPS24	Conserved protein implicated in secretion
LN02_01023|LN02Chr01: 4134908-4137966(+)|974	CDD:225165	28.358	67	35	3	268	321	29	95	6.50E-06	46.5	COG2256	MGS1	ATPase related to the helicase subunit of the Holliday junction resolvase
LN02_01087|LN02Chr01: 4378624-4380424(-)|451	CDD:227910	28.372	430	285	7	21	441	4	419	1.94E-91	281	COG5623	CLP1	Predicted GTPase subunit of the pre-mRNA cleavage complex
LN02_01215|LN02Chr01: 4901611-4902789(-)|324	CDD:224327	29.487	78	39	3	58	119	1	78	7.68E-08	50	COG1409	Icc	Predicted phosphohydrolases
LN02_01407|LN02Chr01: 5627664-5629839(+)|655	CDD:223483	23.707	232	136	6	402	629	2	196	1.98E-24	98.7	COG0406	phoE	Broad specificity phosphatase PhoE and related phosphatases
LN02_01599|LN02Chr01: 6426405-6426968(-)|140	CDD:226370	37.838	37	18	2	45	76	309	345	6.37E-04	35.4	COG3852	NtrB	Signal transduction histidine kinase, nitrogen specific
LN02_01727|LN02Chr01: 6867342-6868469(+)|375	CDD:226022	31.212	330	202	5	36	364	4	309	1.27E-74	231	COG3491	PcbC	Isopenicillin N synthase and related dioxygenases
LN02_01855|LN02Chr01: 7226550-7229061(+)|787	CDD:227507	19.895	191	129	7	146	324	425	603	6.08E-04	40.1	COG5180	PBP1	Protein interacting with poly(A)-binding protein
LN02_01919|LN02Chr01: 7410151-7411081(-)|266	CDD:223959	32.946	258	158	9	15	264	1	251	1.85E-53	171	COG1028	FabG	Dehydrogenases with different specificities (related to short-chain alcohol dehydrogenases)
LN02_02111|LN02Chr02: 646298-648556(-)|492	CDD:227359	48.117	478	233	8	6	480	1	466	0	538	COG5026	COG5026	Hexokinase
LN02_02175|LN02Chr02: 822490-823763(+)|335	CDD:227558	19.876	322	203	10	27	306	67	375	1.73E-34	127	COG5233	GRH1	Peripheral Golgi membrane protein

LN02_02495 ǀ LN02Chr02: 2018584-2021427(+) ǀ 893	CDD:2241 36	19.366	284	204	8	508	787	138	400	1.45E-13	70.7	COG12 15	COG121 5	Glycosyltransferases, probably involved in cell wall biogenesis
LN02_02687 ǀ LN02Chr02: 2680767-2682647(+) ǀ 565	CDD:2276 25	31.418	261	166	7	301	556	47	299	2.76E-47	164	COG53 09	COG530 9	Exo-beta-1,3-glucanase
LN02_02751 ǀ LN02Chr02: 2849389-2850253(+) ǀ 229	CDD:2238 64	27.778	72	46	3	140	209	115	182	1.04E-04	39.6	COG07 93	Prc	Periplasmic protease
LN02_02943 ǀ LN02Chr02: 3539009-3539812(-) ǀ 208	CDD:2241 07	31.356	118	68	2	9	124	72	178	1.59E-24	93.8	COG11 86	PrfB	Protein chain release factor B
LN02_03007 ǀ LN02Chr02: 3741728-3744788(-) ǀ 959	CDD:2231 38	34.995	1003	519	30	6	952	4	929	0	853	COG00 60	IleS	Isoleucyl-tRNA synthetase
LN02_03199 ǀ LN02Chr02: 4464222-4464916(+) ǀ 191	CDD:2237 11	31.383	188	123	4	1	185	36	220	1.93E-46	149	COG06 38	PRE1	20S proteasome, alpha and beta subunits
LN02_03327 ǀ LN02Chr02: 4875631-4876623(+) ǀ 330	CDD:2238 99	31.088	193	119	5	35	227	29	207	1.33E-38	135	COG08 29	UreH	Urease accessory protein UreH
LN02_03519 ǀ LN02Chr02: 5859250-5860194(-) ǀ 314	CDD:2262 97	25.676	222	122	10	65	267	82	279	4.93E-18	80.6	COG37 74	OCH1	Mannosyltransferase OCH1 and related enzymes
LN02_03647 ǀ LN02Chr03: 112443-114722(-) ǀ 569	CDD:2264 06	24.348	115	73	4	337	443	739	847	3.44E-04	40.6	COG38 89	COG388 9	Predicted solute binding protein
LN02_03711 ǀ LN02Chr03: 383806-385477(+) ǀ 491	CDD:2243 22	22.093	344	194	13	147	483	130	406	1.63E-05	44.2	COG14 04	AprE	Subtilisin-like serine proteases
LN02_03775 ǀ LN02Chr03: 719973-723303(+) ǀ 877	CDD:2243 89	27.481	393	210	12	86	458	48	385	2.89E-49	176	COG14 72	BglX	Beta-glucosidase-related glycosidases
LN02_03903 ǀ LN02Chr03: 1084980-1086056(+) ǀ 358	CDD:2250 86	19.598	199	121	9	166	337	100	286	3.95E-05	41.6	COG21 75	TauD	Probable taurine catabolism dioxygenase
LN02_03967 ǀ LN02Chr03: 1271941-1273356(+) ǀ 389	CDD:2251 43	21	300	206	13	107	389	124	409	1.91E-25	104	COG22 34	Iap	Predicted aminopeptidases
LN02_04031 ǀ LN02Chr03: 1508020-1509115(-) ǀ 339	CDD:2271 22	27.451	102	70	2	160	260	303	401	4.60E-05	42	COG47 83	COG478 3	Putative Zn-dependent protease, contains TPR repeats
LN02_04159 ǀ LN02Chr03: 2905118-2907425(+) ǀ 622	CDD:2276 80	15.706	503	279	11	117	617	163	522	1.09E-12	67.5	COG53 91	COG539 1	Phox homology (PX) domain protein
LN02_04223 ǀ LN02Chr03: 3415156-3417608(-) ǀ 699	CDD:2237 71	26.254	598	383	10	106	697	1	546	1.95E-87	282	COG06 99	COG069 9	Predicted GTPases (dynamin-related)
LN02_04351 ǀ LN02Chr03: 3884220-3884813(+) ǀ 197	CDD:2235 60	29.897	97	45	4	13	109	7	80	1.16E-10	56.9	COG04 84	DnaJ	DnaJ-class molecular chaperone with C-terminal Zn finger domain
LN02_04415 ǀ LN02Chr03: 4103272-4104424(+) ǀ 306	CDD:2237 20	36.77	291	155	6	17	306	3	265	8.78E-77	232	COG06 47	NagD	Predicted sugar phosphatases of the HAD superfamily
LN02_04607 ǀ LN02Chr03: 4782206-4782981(-) ǀ 221	CDD:2235 77	40.361	166	95	1	44	209	3	164	4.14E-53	166	COG05 03	Apt	Adenine/guanine phosphoribosyltransferases and related PRPP-binding proteins
LN02_04671 ǀ LN02Chr03: 5014019-5016220(-) ǀ 733	CDD:2278 20	18.72	422	214	16	143	543	64	377	2.11E-14	72.7	COG55 33	UBP5	Ubiquitin C-terminal hydrolase
LN02_05119 ǀ LN02Chr04: 679837-680784(-) ǀ 315	CDD:2234 65	36.14	285	163	7	23	303	1	270	6.91E-60	190	COG03 88	COG038 8	Predicted amidohydrolase
LN02_05375 ǀ LN02Chr04: 1531706-1532970(-) ǀ 357	CDD:2274 05	34.156	243	144	8	115	342	17	258	1.97E-50	166	COG50 73	VID24	Vacuolar import and degradation protein

LN02_05503|LN02Chr04:2122798-2123790(-)|318	CDD:226750	28.079	203	131	6	4	200	2	195	4.75E-25	97	COG4300	CadD	Predicted permease, cadmium resistance protein
LN02_05631|LN02Chr04:2687813-2689618(-)|552	CDD:223096	35.938	512	231	16	42	545	6	428	2.44E-142	415	COG0017	AsnS	Aspartyl/asparaginyl-tRNA synthetases
LN02_05695|LN02Chr04:2907841-2909670(-)|576	CDD:224164	49.814	538	245	11	32	567	1	515	0	746	COG1243	ELP3	Histone acetyltransferase
LN02_05759|LN02Chr04:3097604-3100835(-)|900	CDD:223589	32.857	280	159	7	38	288	1	280	5.60E-47	169	COG0515	SPS1	Serine/threonine protein kinase
LN02_05951|LN02Chr04:3800796-3803752(+)|866	CDD:223796	17.259	197	153	3	429	616	110	305	3.49E-15	74.6	COG0724	COG0724	RNA-binding proteins (RRM domain)
LN02_06143|LN02Chr04:4408904-4411098(+)|528	CDD:223727	24.932	365	240	13	13	360	2	349	1.11E-54	186	COG0654	UbiH	2-polyprenyl-6-methoxyphenol hydroxylase and related FAD-dependent oxidoreductases
LN02_06335|LN02Chr04:5072054-5074931(+)|894	CDD:223457	43.146	445	224	9	188	625	64	486	5.98E-164	484	COG0380	OtsA	Trehalose-6-phosphate synthase
LN02_06335|LN02Chr04:5072054-5074931(+)|894	CDD:224789	30.038	263	166	6	628	890	2	246	1.03E-44	159	COG1877	OtsB	Trehalose-6-phosphatase
LN02_06463|LN02Chr04:5460875-5463059(-)|627	CDD:227270	21.212	594	378	25	47	620	48	571	2.85E-20	92.5	COG4934	COG4934	Predicted protease
LN02_06527|LN02Chr04:5738989-5739996(-)|335	CDD:223528	25.153	326	204	10	6	310	3	309	8.55E-34	123	COG0451	WcaG	Nucleoside-diphosphate-sugar epimerases
LN02_06719|LN02Chr05:573629-576748(-)|769	CDD:226406	21.186	118	82	3	170	282	751	862	2.11E-04	41.8	COG3889	COG3889	Predicted solute binding protein
LN02_06783|LN02Chr05:806833-808039(-)|339	CDD:223236	47.706	327	166	5	14	339	1	323	1.97E-142	403	COG0158	Fbp	Fructose-1,6-bisphosphatase
LN02_06911|LN02Chr05:1241341-1242884(+)|479	CDD:225741	47.761	469	208	7	6	469	5	441	0	558	COG3200	AroG	3-deoxy-D-arabino-heptulosonate 7-phosphate (DAHP) synthase
LN02_06975|LN02Chr05:1839492-1840879(-)|390	CDD:225201	20.619	388	258	10	1	380	4	349	2.30E-18	83.6	COG2319	COG2319	FOG: WD40 repeat
LN02_07167|LN02Chr05:2787854-2788859(+)|240	CDD:223955	22.692	260	148	8	1	223	4	247	6.21E-12	60.5	COG1024	CaiD	Enoyl-CoA hydratase/carnithine racemase
LN02_07231|LN02Chr05:3039660-3041098(+)|446	CDD:223704	21.429	210	140	7	129	323	16	215	9.48E-13	65.1	COG0631	PTC1	Serine/threonine protein phosphatase
LN02_07295|LN02Chr05:3242614-3245631(+)|920	CDD:223957	35.268	913	510	14	24	874	1	894	0	669	COG1026	COG1026	Predicted Zn-dependent peptidases, insulinase-like
LN02_07359|LN02Chr05:3440568-3443430(+)|803	CDD:227535	25.538	325	204	6	408	729	186	475	2.57E-52	187	COG5210	COG5210	GTPase-activating protein
LN02_07551|LN02Chr05:4137408-4137900(+)|113	CDD:224869	24.658	73	51	2	7	75	7	79	1.44E-13	58.1	COG1958	LSM1	Small nuclear ribonucleoprotein (snRNP) homolog
LN02_07807|LN02Chr05:5329899-5331228(-)|326	CDD:224016	27.044	318	188	11	10	326	4	278	2.26E-44	150	COG1091	RfbD	dTDP-4-dehydrorhamnose reductase
LN02_07871|LN02Chr05:5555873-5557025(+)|321	CDD:226217	31.657	338	197	10	7	317	7	337	1.17E-72	225	COG3693	XynA	Beta-1,4-xylanase
LN02_08127|LN02Chr06:1349036-1351213(+)|688	CDD:227532	45.161	62	29	2	398	454	163	224	7.47E-13	68.9	COG5207	UBP14	Isopeptidase T

LN02_08127|LN02Chr06:1349036-1351213(+)|688	CDD:227827	34.884	43	25	1	342	381	326	368	1.96E-06	47.7	COG5540	COG5540	RING-finger-containing ubiquitin ligase
LN02_08319|LN02Chr06:2094720-2096660(-)|610	CDD:225201	23.022	417	300	10	23	431	29	432	4.26E-28	114	COG2319	COG2319	FOG: WD40 repeat
LN02_08511|LN02Chr06:2826689-2830945(-)|1317	CDD:224117	18.137	204	159	3	21	220	648	847	9.28E-09	57	COG1196	Smc	Chromosome segregation ATPases
LN02_08703|LN02Chr06:3635893-3637113(+)|384	CDD:223878	39.695	262	89	6	118	379	1	193	1.88E-72	221	COG0807	RibA	GTP cyclohydrolase II

LN02_08831|LN02Chr07:478655-479512(-)|229	CDD:223113	38.636	220	119	7	6	224	3	207	2.44E-59	183	COG0035	Upp	Uracil phosphoribosyltransferase
LN02_08959|LN02Chr07:962994-966937(-)|1218	CDD:225201	25.538	325	203	8	8	327	148	438	4.24E-38	146	COG2319	COG2319	FOG: WD40 repeat
LN02_09087|LN02Chr07:1385738-1386685(+)|264	CDD:224025	32.787	183	116	5	53	229	3	184	2.04E-43	144	COG1100	COG1100	GTPase SAR1 and related small G proteins
LN02_00064|LN02Chr01:371619-372095(+)|158	CDD:223611	33.333	132	81	1	9	140	14	138	1.78E-27	97.1	COG0537	Hit	Diadenosine tetraphosphate (Ap4A) hydrolase and other HIT family hydrolases
LN02_00256|LN02Chr01:1471590-1476583(+)|1542	CDD:223233	40.177	565	281	12	965	1528	2	510	0	580	COG0155	CysI	Sulfite reductase, beta subunit (hemoprotein)
LN02_00256|LN02Chr01:1471590-1476583(+)|1542	CDD:223945	24.268	239	138	9	562	780	73	288	2.70E-34	131	COG1013	PorB	Pyruvate:ferredoxin oxidoreductase and related 2-oxoacid:ferredoxin oxidoreductases, beta subunit
LN02_00256|LN02Chr01:1471590-1476583(+)|1542	CDD:223446	27.309	249	153	6	800	1031	47	284	9.18E-31	126	COG0369	CysJ	Sulfite reductase, alpha subunit (flavoprotein)
LN02_00256|LN02Chr01:1471590-1476583(+)|1542	CDD:223746	20.282	355	212	9	99	443	70	363	1.54E-22	98.5	COG0674	PorA	Pyruvate:ferredoxin oxidoreductase and related 2-oxoacid:ferredoxin oxidoreductases, alpha subunit
LN02_00384|LN02Chr01:1971902-1973302(-)|466	CDD:223796	16.889	225	178	3	167	385	50	271	3.08E-10	58.4	COG0724	COG0724	RNA-binding proteins (RRM domain)
LN02_00384|LN02Chr01:1971902-1973302(-)|466	CDD:223796	15.354	254	181	5	27	278	49	270	1.55E-09	56.1	COG0724	COG0724	RNA-binding proteins (RRM domain)
LN02_00512|LN02Chr01:2324971-2328066(+)|1009	CDD:227585	24.627	268	121	9	711	976	159	347	8.32E-23	100	COG5260	TRF4	DNA polymerase sigma
LN02_00512|LN02Chr01:2324971-2328066(+)|1009	CDD:227585	20.863	139	92	3	206	344	41	161	7.47E-09	56.3	COG5260	TRF4	DNA polymerase sigma
LN02_00896|LN02Chr01:3708030-3709487(+)|377	CDD:223794	63.112	347	126	1	12	358	6	350	0	568	COG0722	AroG	3-deoxy-D-arabino-heptulosonate 7-phosphate (DAHP) synthase
LN02_01024|LN02Chr01:4138272-4140114(-)|494	CDD:224223	35.262	363	205	5	112	459	1	348	4.87E-108	323	COG1304	idi	Isopentenyl diphosphate isomerase (BS_ypgA, MTH48 and related proteins)
LN02_01024|LN02Chr01:4138272-4140114(-)|494	CDD:227599	48.333	60	31	0	2	61	52	111	1.72E-15	71.4	COG5274	CYB5	Cytochrome b involved in lipid metabolism
LN02_01344|LN02Chr01:5371716-5373087(+)|412	CDD:223652	29.765	383	245	12	37	410	8	375	1.68E-85	264	COG0579	COG0579	Predicted dehydrogenase

LN02_01856∣LN02Chr01:7230062-7230871(+)∣269	CDD:224727	20.179	223	162	5	47	264	14	225	4.02E-15	69.7	COG1814	COG1814	Uncharacterized membrane protein
LN02_01984∣LN02Chr02:200776-202127(-)∣378	CDD:226022	27.485	342	219	7	29	365	5	322	5.93E-71	222	COG3491	PcbC	Isopenicillin N synthase and related dioxygenases
LN02_02048∣LN02Chr02:460335-461422(+)∣250	CDD:223959	31.95	241	146	7	1	231	3	235	2.59E-36	126	COG1028	FabG	Dehydrogenases with different specificities (related to short-chain alcohol dehydrogenases)
LN02_02240∣LN02Chr02:1041628-1042898(+)∣303	CDD:223796	38.889	90	55	0	1	90	109	198	1.33E-22	92.7	COG0724	COG0724	RNA-binding proteins (RRM domain)
LN02_02496∣LN02Chr02:2022575-2025993(-)∣1110	CDD:223562	26.923	624	254	15	479	1015	4	512	1.01E-112	358	COG0488	Uup	ATPase components of ABC transporters with duplicated ATPase domains
LN02_02560∣LN02Chr02:2243069-2244856(+)∣505	CDD:226107	45.618	445	223	8	64	505	15	443	7.52E-169	481	COG3579	PepC	Aminopeptidase C
LN02_02688∣LN02Chr02:2685184-2687511(-)∣729	CDD:225201	22.761	268	193	6	399	659	123	383	3.18E-11	63.2	COG2319	COG2319	FOG: WD40 repeat
LN02_02752∣LN02Chr02:2851443-2852507(+)∣216	CDD:224025	29.101	189	113	4	7	174	4	192	6.38E-40	133	COG1100	COG1100	GTPase SAR1 and related small G proteins
LN02_03200∣LN02Chr02:4465432-4468792(-)∣1000	CDD:223550	35.318	974	532	19	12	978	33	915	0	765	COG0474	MgtA	Cation transport ATPase
LN02_03264∣LN02Chr02:4683133-4683819(+)∣186	CDD:223532	33.566	143	86	4	37	172	36	176	1.74E-23	88.9	COG0456	RimI	Acetyltransferases
LN02_03392∣LN02Chr02:5069296-5074053(+)∣1500	CDD:223627	24.297	391	229	14	324	687	359	709	1.41E-17	85.9	COG0553	HepA	Superfamily II DNA/RNA helicases, SNF2 family
LN02_03392∣LN02Chr02:5069296-5074053(+)∣1500	CDD:223627	33.884	121	69	3	1287	1398	713	831	9.58E-12	66.7	COG0553	HepA	Superfamily II DNA/RNA helicases, SNF2 family
LN02_03392∣LN02Chr02:5069296-5074053(+)∣1500	CDD:227719	34.043	47	31	0	1157	1203	28	74	1.45E-06	49.3	COG5432	RAD18	RING-finger-containing E3 ubiquitin ligase
LN02_03520∣LN02Chr02:5861638-5862817(+)∣362	CDD:223453	26.519	181	79	9	124	258	99	271	1.90E-07	49.7	COG0376	KatG	Catalase (peroxidase I)
LN02_03648∣LN02Chr03:121027-125433(-)∣1468	CDD:224055	25.287	522	356	10	961	1462	43	550	3.33E-69	241	COG1132	MdlB	ABC-type multidrug transport system, ATPase and permease components
LN02_03648∣LN02Chr03:121027-125433(-)∣1468	CDD:224055	22.069	580	371	17	288	842	16	539	3.54E-34	136	COG1132	MdlB	ABC-type multidrug transport system, ATPase and permease components
LN02_03776∣LN02Chr03:723683-724658(-)∣153	CDD:226211	25.472	106	63	3	21	120	4	99	4.07E-04	35.5	COG3686	COG3686	Predicted membrane protein
LN02_03968∣LN02Chr03:1274562-1275516(-)∣260	CDD:227424	26.432	227	114	7	1	223	1	178	2.90E-29	106	COG5093	COG5093	Uncharacterized conserved protein
LN02_04160∣LN02Chr03:2908118-2909381(+)∣394	CDD:227551	35.264	397	232	9	4	394	3	380	8.13E-106	315	COG5226	CEG1	mRNA capping enzyme, guanylyltransferase (alpha) subunit
LN02_04224∣LN02Chr03:3421244-3423177(+)∣583	CDD:225201	27.451	306	201	10	286	582	144	437	5.80E-27	111	COG2319	COG2319	FOG: WD40 repeat
LN02_04352∣LN02Chr03:3885543-3886732(-)∣267	CDD:227373	75.581	258	63	0	4	261	6	263	4.71E-149	414	COG5040	BMH1	14-3-3 family protein
LN02_04416∣LN02Chr03:4104785-4106048(-)∣358	CDD:226275	22.837	289	190	12	48	330	10	271	1.01E-34	125	COG3752	COG3752	Steroid 5-alpha reductase family enzyme

LN02_04544∣LN02Chr03:4509982-4512201(+)∣739	CDD:223589	24.183	306	200	8	379	681	3	279	1.55E-37	141	COG0515	SPS1	Serine/threonine protein kinase
LN02_04672∣LN02Chr03:5016885-5018745(+)∣427	CDD:225086	22.632	190	123	9	223	397	90	270	3.33E-06	45.4	COG2175	TauD	Probable taurine catabolism dioxygenase
LN02_04864∣LN02Chr03:5814005-5815708(-)∣513	CDD:224416	26.943	386	241	10	17	392	1	355	6.92E-83	258	COG1499	NMD3	NMD protein affecting ribosome stability and mRNA decay
LN02_04992∣LN02Chr04:294895-296547(-)∣531	CDD:223697	22.479	476	299	13	56	528	1	409	5.91E-66	216	COG0624	ArgE	Acetylornithine deacetylase/Succinyl-diaminopimelate desuccinylase and related deacylases
LN02_05120∣LN02Chr04:681237-682047(-)∣201	CDD:226295	31.405	121	72	4	41	159	15	126	4.13E-09	50.5	COG3772	COG3772	Phage-related lysozyme (muraminidase)
LN02_05184∣LN02Chr04:889804-892068(+)∣754	CDD:223782	27.309	249	159	10	176	421	1	230	3.04E-29	113	COG0710	AroD	3-dehydroquinate dehydratase
LN02_05248∣LN02Chr04:1101205-1104212(-)∣938	CDD:224418	25.674	853	471	18	31	873	27	726	1.50E-126	398	COG1501	COG1501	Alpha-glucosidases, family 31 of glycosyl hydrolases
LN02_05376∣LN02Chr04:1534686-1536101(+)∣374	CDD:227402	19.835	242	169	5	123	361	90	309	1.13E-04	40.6	COG5070	VRG4	Nucleotide-sugar transporter
LN02_05568∣LN02Chr04:2479808-2481770(-)∣507	CDD:223133	73.191	470	106	5	48	502	4	468	0	870	COG0055	AtpD	F0F1-type ATP synthase, beta subunit
LN02_05632∣LN02Chr04:2692380-2693426(+)∣348	CDD:226745	26.203	187	131	4	101	284	98	280	7.72E-11	59.2	COG4295	COG4295	Uncharacterized protein conserved in bacteria
LN02_05696∣LN02Chr04:2910330-2911649(+)∣332	CDD:223152	62.799	293	105	2	38	330	5	293	1.57E-147	414	COG0074	SucD	Succinyl-CoA synthetase, alpha subunit
LN02_05760∣LN02Chr04:3102712-3107126(-)∣1447	CDD:223627	27.239	804	344	17	499	1283	272	853	1.66E-97	331	COG0553	HepA	Superfamily II DNA/RNA helicases, SNF2 family
LN02_05760∣LN02Chr04:3102712-3107126(-)∣1447	CDD:225177	21.472	163	106	6	53	204	221	372	6.41E-04	41	COG2268	COG2268	Uncharacterized protein conserved in bacteria
LN02_05952∣LN02Chr04:3804392-3806320(-)∣598	CDD:223617	18.43	293	138	11	297	581	14	213	2.01E-09	55.5	COG0543	UbiB	2-polyprenylphenol hydroxylase and related flavodoxin oxidoreductases
LN02_05952∣LN02Chr04:3804392-3806320(-)∣598	CDD:226582	21.159	397	230	20	117	475	10	361	8.66E-04	39	COG4097	COG4097	Predicted ferric reductase
LN02_06080∣LN02Chr04:4196302-4197480(-)∣374	CDD:223796	17.04	223	174	3	120	336	7	224	8.30E-04	38	COG0724	COG0724	RNA-binding proteins (RRM domain)
LN02_06336∣LN02Chr04:5075762-5077062(+)∣392	CDD:223193	27.046	281	174	6	39	315	5	258	1.12E-44	153	COG0115	IlvE	Branched-chain amino acid aminotransferase/4-amino-4-deoxychorismate lyase
LN02_06400∣LN02Chr04:5261845-5265762(-)∣1114	CDD:223738	27.094	203	118	6	396	576	41	235	2.89E-16	76.8	COG0666	Arp	FOG: Ankyrin repeat
LN02_06400∣LN02Chr04:5261845-5265762(-)∣1114	CDD:223738	33.333	81	52	1	993	1071	119	199	2.99E-05	43.7	COG0666	Arp	FOG: Ankyrin repeat
LN02_06400∣LN02Chr04:5261845-5265762(-)∣1114	CDD:223738	24.528	159	109	4	915	1063	1	158	4.00E-05	43.3	COG0666	Arp	FOG: Ankyrin repeat
LN02_06400∣LN02Chr04:5261845-5265762(-)∣1114	CDD:223589	18.63	365	207	9	685	1007	41	357	3.02E-11	63.6	COG0515	SPS1	Serine/threonine protein kinase

LN02_06464|LN02Chr04:5465238-5466209(+)|323	CDD:223236	28.613	346	182	15	1	314	4	316	1.50E-47	160	COG0158	Fbp	Fructose-1,6-bisphosphatase
LN02_06912|LN02Chr05:1245218-1246927(-)|519	CDD:225371	25.85	147	100	5	71	215	51	190	2.79E-04	40.3	COG2814	AraJ	Arabinose efflux permease
LN02_07104|LN02Chr05:2584634-2585252(+)|173	CDD:223796	25.217	115	86	0	43	157	108	222	9.62E-23	89.2	COG0724	COG0724	RNA-binding proteins (RRM domain)
LN02_07296|LN02Chr05:3249755-3251193(+)|446	CDD:223098	27.047	403	251	10	43	425	7	386	5.96E-88	270	COG0019	LysA	Diaminopimelate decarboxylase
LN02_07872|LN02Chr05:5557324-5558871(-)|456	CDD:227170	23.404	235	146	9	143	359	93	311	2.26E-09	55.8	COG4833	COG4833	Predicted glycosyl hydrolase
LN02_07936|LN02Chr05:5730855-5732447(+)|530	CDD:223354	22.973	222	150	6	41	256	32	238	1.09E-18	85.7	COG0277	GlcD	FAD/FMN-containing dehydrogenases
LN02_08576|LN02Chr06:3061625-3062867(+)|291	CDD:227398	30.18	222	143	3	1	213	1	219	9.75E-33	118	COG5066	SCS2	VAMP-associated protein involved in inositol metabolism
LN02_08640|LN02Chr06:3266537-3268602(+)|577	CDD:223903	49.225	516	245	7	63	576	41	541	0	608	COG0833	LysP	Amino acid transporters
LN02_08832|LN02Chr07:480122-483215(-)|983	CDD:224706	23.142	471	273	22	214	657	34	442	9.16E-40	150	COG1793	CDC9	ATP-dependent DNA ligase
LN02_09024|LN02Chr07:1170692-1171836(-)|306	CDD:223563	42.857	231	116	7	35	261	47	265	5.73E-75	228	COG0489	Mrp	ATPases involved in chromosome partitioning
LN02_00264|LN02Chr01:1495081-1500741(-)|1859	CDD:227623	25.287	1218	689	33	316	1508	1	1022	0	575	COG5307	COG5307	SEC7 domain proteins
LN02_00456|LN02Chr01:2169724-2171178(-)|484	CDD:225201	30.196	255	128	8	60	310	108	316	1.64E-21	94	COG2319	COG2319	FOG: WD40 repeat
LN02_00584|LN02Chr01:2617968-2618809(+)|202	CDD:225112	24.074	108	70	1	95	202	130	225	4.57E-05	39.8	COG2202	AtoS	FOG: PAS/PAC domain
LN02_00648|LN02Chr01:2859323-2862598(-)|1025	CDD:227709	25.954	262	164	9	235	471	485	741	2.06E-14	74.9	COG5422	ROM1	RhoGEF, Guanine nucleotide exchange factor for Rho/Rac/Cdc42-like GTPases
LN02_00840|LN02Chr01:3555696-3557759(-)|536	CDD:223572	27.29	513	267	14	19	528	2	411	2.52E-127	375	COG0498	ThrC	Threonine synthase
LN02_00904|LN02Chr01:3725128-3726495(+)|455	CDD:225035	23.414	457	288	17	1	449	1	403	3.02E-39	143	COG2124	CypX	Cytochrome P450
LN02_00968|LN02Chr01:3958955-3960591(-)|485	CDD:226143	26.477	457	228	18	17	468	6	359	1.13E-45	160	COG3616	COG3616	Predicted amino acid aldolase or racemase
LN02_01096|LN02Chr01:4415675-4417657(+)|518	CDD:225035	24.67	227	118	9	294	515	233	411	1.03E-19	88.3	COG2124	CypX	Cytochrome P450
LN02_01224|LN02Chr01:4936017-4936963(-)|232	CDD:224923	48.75	80	41	0	153	232	1	80	1.44E-35	118	COG2012	RPB5	DNA-directed RNA polymerase, subunit H, RpoH/RPB5
LN02_01352|LN02Chr01:5415956-5416939(+)|245	CDD:223711	42.017	238	127	4	8	242	3	232	1.42E-82	244	COG0638	PRE1	20S proteasome, alpha and beta subunits
LN02_01480|LN02Chr01:5955017-5956957(-)|646	CDD:225174	26.052	499	280	17	138	620	6	431	3.30E-85	271	COG2265	TrmA	SAM-dependent methyltransferases related to tRNA (uracil-5-)-methyltransferase
LN02_01544|LN02Chr01:6213050-6214701(-)|468	CDD:225371	18.414	353	265	9	116	458	48	387	7.92E-15	73	COG2814	AraJ	Arabinose efflux permease

LN02_01608|LN02Chr01:6466373-6468741(+)|756	CDD:227602	23.952	167	95	8	83	232	74	225	1.94E-07	50.9	COG5277	COG5277	Actin and related proteins
LN02_01736|LN02Chr01:6887683-6890079(+)|688	CDD:227408	22.523	111	75	2	288	397	147	247	1.67E-08	54	COG5076	COG5076	Transcription factor involved in chromatin remodeling, contains bromodomain
LN02_01736|LN02Chr01:6887683-6890079(+)|688	CDD:227408	22.628	137	100	1	2	138	112	242	2.47E-06	47.1	COG5076	COG5076	Transcription factor involved in chromatin remodeling, contains bromodomain
LN02_01800|LN02Chr01:7063956-7066595(+)|853	CDD:225789	21.637	684	436	26	4	653	10	627	1.16E-86	290	COG3250	LacZ	Beta-galactosidase/beta-glucuronidase
LN02_01864|LN02Chr01:7249704-7251085(-)|388	CDD:223669	22.321	336	199	6	47	381	7	281	8.77E-14	68.1	COG0596	MhpC	Predicted hydrolases or acyltransferases (alpha/beta hydrolase superfamily)
LN02_02504|LN02Chr02:2045649-2047475(+)|608	CDD:223949	25.373	134	73	4	459	592	111	217	0.001	38.4	COG1018	Hmp	Flavodoxin reductases (ferredoxin-NADPH reductases) family 1
LN02_02568|LN02Chr02:2275191-2277430(+)|624	CDD:223354	35.332	467	278	10	160	612	1	457	1.26E-113	345	COG0277	GlcD	FAD/FMN-containing dehydrogenases
LN02_02632|LN02Chr02:2492406-2494661(-)|659	CDD:223589	28.793	323	169	8	279	584	1	279	2.02E-46	166	COG0515	SPS1	Serine/threonine protein kinase
LN02_02760|LN02Chr02:2876198-2877535(-)|348	CDD:223141	30.599	317	172	9	21	335	14	284	2.32E-61	195	COG0063	COG0063	Predicted sugar kinase
LN02_02952|LN02Chr02:3572854-3576678(-)|1246	CDD:227604	25.052	479	230	14	780	1242	156	521	2.46E-40	154	COG5279	CYK3	Uncharacterized protein involved in cytokinesis, contains TGc (transglutaminase/protease-like) domain
LN02_03080|LN02Chr02:4032110-4034605(-)|831	CDD:223989	30.425	447	272	13	319	743	13	442	9.51E-105	327	COG1061	SSL2	DNA or RNA helicases of superfamily II
LN02_03144|LN02Chr02:4291505-4291924(-)|139	CDD:225218	31.373	102	59	6	22	115	77	175	6.74E-14	63	COG2340	COG2340	Uncharacterized protein with SCP/PR1 domains
LN02_03400|LN02Chr02:5097972-5100800(-)|820	CDD:227492	23.77	122	83	3	471	587	474	590	7.03E-04	40.1	COG5163	NOP7	Protein required for biogenesis of the 60S ribosomal subunit
LN02_03400|LN02Chr02:5097972-5100800(-)|820	CDD:224117	15.917	289	214	8	317	591	691	964	7.52E-04	40.1	COG1196	Smc	Chromosome segregation ATPases
LN02_03720|LN02Chr03:425672-427891(+)|699	CDD:225201	30.055	366	235	12	337	682	34	398	6.77E-41	153	COG2319	COG2319	FOG: WD40 repeat
LN02_03784|LN02Chr03:745029-746394(+)|371	CDD:227022	25	312	175	14	40	321	96	378	1.31E-17	80.8	COG4677	PemB	Pectin methylesterase
LN02_03848|LN02Chr03:924039-925106(-)|355	CDD:224777	30.916	262	158	7	60	316	29	272	1.03E-73	227	COG1864	NUC1	DNA/RNA endonuclease G, NUC1
LN02_03976|LN02Chr03:1310468-1313218(-)|843	CDD:227568	25.612	449	258	11	4	441	1	384	1.42E-47	174	COG5243	HRD1	HRD ubiquitin ligase complex, ER membrane component
LN02_04168|LN02Chr03:2951456-2952239(+)|185	CDD:224293	39.56	182	101	4	1	179	1	176	2.50E-55	170	COG1374	NIP7	Protein involved in ribosomal biogenesis, contains PUA domain
LN02_04232|LN02Chr03:3452323-3454194(-)|497	CDD:225035	24.773	440	254	18	32	459	6	380	5.44E-36	135	COG2124	CypX	Cytochrome P450
LN02_04424|LN02Chr03:4139345-4142164(+)|841	CDD:227430	25.283	795	498	24	1	756	23	760	1.07E-82	278	COG5099	COG5099	RNA-binding protein of the Puf family, translational repressor

LN02_04488 | LN02Chr03 :4319390-4321642(-) | 719	CDD:2234 92	24.869	571	305	21	8	573	5	456	3.15E-77	253	COG04 15	PhrB	Deoxyribodipyrimidine photolyase
LN02_04808 | LN02Chr03 :5481345-5482301(+) | 318	CDD:2251 82	22.383	277	168	15	28	297	20	256	4.69E-08	50.9	COG22 73	SKN1	Beta-glucanase/Beta-glucan synthetase
LN02_05000 | LN02Chr04 :309081-312477(-) | 1032	CDD:2276 93	43.151	1022	551	12	1	1017	1	997	0	927	COG54 06	COG540 6	Nucleosome binding factor SPN, SPT16 subunit
LN02_05064 | LN02Chr04 :498021-498988(-) | 230	CDD:2250 47	21.827	197	75	5	85	208	1	191	5.67E-11	57	COG21 36	IMP4	Predicted exosome subunit/U3 small nucleolar ribonucleoprotein (snoRNP) component, contains IMP4 domain
LN02_05128 | LN02Chr04 :696655-697909(-) | 319	CDD:2252 01	28.205	312	196	10	4	305	79	372	2.16E-39	141	COG23 19	COG231 9	FOG: WD40 repeat
LN02_05320 | LN02Chr04 :1362526-1364244(+) | 438	CDD:2236 88	36.641	131	80	3	150	280	1	128	2.66E-45	151	COG06 15	TagD	Cytidylyltransferase
LN02_05576 | LN02Chr04 :2506756-2508337(-) | 496	CDD:2237 69	15.951	326	225	5	113	436	6	284	1.77E-08	52.9	COG06 97	RhaT	Permeases of the drug/metabolite transporter (DMT) superfamily
LN02_05832 | LN02Chr04 :3353065-3355787(+) | 854	CDD:2275 07	29.073	313	184	9	2	280	90	398	2.28E-26	112	COG51 80	PBP1	Protein interacting with poly(A)-binding protein
LN02_05960 | LN02Chr04 :3830319-3831835(-) | 486	CDD:2278 86	33.441	311	172	10	117	425	16	293	2.48E-53	179	COG55 99	PTP2	Protein tyrosine phosphatase
LN02_06024 | LN02Chr04 :4033697-4035628(-) | 643	CDD:2244 19	23.684	152	92	4	439	589	276	404	5.19E-11	62.2	COG15 02	Cls	Phosphatidylserine/phosphatidylglycero phosphate/cardiolipin synthases and related enzymes
LN02_06152 | LN02Chr04 :4437076-4439245(-) | 605	CDD:2252 49	25.891	645	366	24	20	604	198	790	1.96E-57	203	COG23 74	COG237 4	Predicted extracellular nuclease
LN02_06408 | LN02Chr04 :5293578-5294921(-) | 377	CDD:2260 22	20.772	337	203	14	5	333	4	284	5.45E-11	60.1	COG34 91	PcbC	Isopenicillin N synthase and related dioxygenases
LN02_06536 | LN02Chr04 :5774777-5778940(+) | 1123	CDD:2234 97	23.237	241	124	5	388	627	1	181	4.68E-32	126	COG04 20	SbcD	DNA repair exonuclease
LN02_07112 | LN02Chr05 :2601081-2602004(+) | 307	CDD:2275 72	44.706	85	47	0	190	274	21	105	1.86E-29	105	COG52 47	BUR6	Class 2 transcription repressor NC2, alpha subunit (DRAP1 homolog)
LN02_07304 | LN02Chr05 :3276269-3278530(-) | 670	CDD:2232 21	36.833	562	297	9	50	583	2	533	1.32E-170	496	COG01 43	MetG	Methionyl-tRNA synthetase
LN02_07496 | LN02Chr05 :3969619-3972639(+) | 1006	CDD:2239 57	37.257	977	575	15	24	983	1	956	0	800	COG10 26	COG102 6	Predicted Zn-dependent peptidases, insulinase-like
LN02_07688 | LN02Chr05 :4846704-4847680(-) | 287	CDD:2247 11	47.312	279	126	5	1	277	1	260	1.56E-108	312	COG17 98	DPH5	Diphthamide biosynthesis methyltransferase
LN02_07752 | LN02Chr05 :5081606-5082620(-) | 252	CDD:2237 10	27.35	234	147	7	3	236	1	211	1.73E-26	99.8	COG06 37	COG063 7	Predicted phosphatase/phosphohexomutase
LN02_07944 | LN02Chr05 :5758589-5760033(+) | 447	CDD:2267 42	19.435	283	216	5	37	316	16	289	1.14E-09	56.7	COG42 92	COG429 2	Predicted membrane protein
LN02_08200 | LN02Chr06 :1753233-1757542(+) | 1313	CDD:2276 10	32.811	1277	763	31	1	1248	1	1211	0	847	COG52 90	COG529 0	IkappaB kinase complex, IKAP component
LN02_08264 | LN02Chr06 :1932578-1934660(+) | 548	CDD:2241 52	21.206	514	296	24	67	542	6	448	1.44E-18	85.2	COG12 31	COG123 1	Monoamine oxidase

LN02_08328|LN02Chr06:2120370-2121659(+)|390	CDD:223865	38.922	167	93	5	99	264	26	184	7.35E-35	124	COG0794	GutQ	Predicted sugar phosphate isomerase involved in capsule formation
LN02_08392|LN02Chr06:2440512-2442535(+)|578	CDD:227430	12.955	247	197	7	203	449	161	389	7.57E-04	39.3	COG5099	COG5099	RNA-binding protein of the Puf family, translational repressor
LN02_08520|LN02Chr06:2862171-2863459(+)|367	CDD:224092	35.457	361	186	10	9	365	6	323	8.69E-90	271	COG1171	IlvA	Threonine dehydratase
LN02_08584|LN02Chr06:3082267-3087499(+)|1461	CDD:223589	34.13	293	164	8	24	290	1	290	4.65E-52	185	COG0515	SPS1	Serine/threonine protein kinase
LN02_08712|LN02Chr06:3659717-3661185(+)|447	CDD:224399	22.049	449	197	12	4	437	2	312	4.14E-68	216	COG1482	ManA	Phosphomannose isomerase
LN02_08840|LN02Chr07:511067-512812(-)|581	CDD:226470	40.463	561	310	8	1	557	1	541	0	559	COG3961	COG3961	Pyruvate decarboxylase and related thiamine pyrophosphate-requiring enzymes
LN02_08904|LN02Chr07:751313-753544(+)|641	CDD:227583	36.884	507	292	7	100	604	43	523	7.39E-123	371	COG5258	GTPBP1	GTPase
LN02_09224|LN02Chr07:1799822-1801392(+)|500	CDD:224169	37.5	448	261	11	56	499	21	453	3.82E-145	421	COG1249	Lpd	Pyruvate/2-oxoglutarate dehydrogenase complex, dihydrolipoamide dehydrogenase (E3) component, and related enzymes
LN02_09288|LN02Chr07:2038769-2039566(+)|265	CDD:226168	32.53	83	44	4	167	240	65	144	1.66E-04	38.8	COG3642	COG3642	Mn2+-dependent serine/threonine protein kinase
LN02_00265|LN02Chr01:1502535-1504010(+)|459	CDD:223582	38.724	439	217	8	36	459	2	403	7.62E-123	360	COG0508	AceF	Pyruvate/2-oxoglutarate dehydrogenase complex, dihydrolipoamide acyltransferase (E2) component, and related enzymes
LN02_00329|LN02Chr01:1792706-1794563(-)|544	CDD:223515	27.182	401	261	8	54	452	1	372	5.06E-51	176	COG0438	RfaG	Glycosyltransferase
LN02_00521|LN02Chr01:2366360-2367742(-)|410	CDD:227187	26.923	208	128	8	170	360	111	311	3.24E-25	103	COG4850	COG4850	Uncharacterized conserved protein
LN02_00585|LN02Chr01:2619282-2620788(-)|376	CDD:223344	26.948	308	176	8	1	294	1	273	2.92E-42	146	COG0266	Nei	Formamidopyrimidine-DNA glycosylase
LN02_00649|LN02Chr01:2868048-2871175(+)|1005	CDD:225201	28.679	265	174	8	346	595	63	327	1.04E-26	112	COG2319	COG2319	FOG: WD40 repeat
LN02_00713|LN02Chr01:3134220-3135212(+)|285	CDD:223365	41.885	191	108	2	72	259	5	195	5.94E-73	220	COG0288	CynT	Carbonic anhydrase
LN02_00969|LN02Chr01:3962240-3964618(-)|709	CDD:227585	33.115	305	182	6	375	669	56	348	2.57E-53	188	COG5260	TRF4	DNA polymerase sigma
LN02_01033|LN02Chr01:4165601-4166653(+)|350	CDD:223644	24.354	271	136	11	57	321	27	234	1.27E-20	86.5	COG0571	Rnc	dsRNA-specific ribonuclease
LN02_01225|LN02Chr01:4938134-4939992(+)|490	CDD:224117	16.129	217	174	4	69	280	697	910	4.10E-08	52.8	COG1196	Smc	Chromosome segregation ATPases
LN02_01545|LN02Chr01:6216496-6217925(-)|391	CDD:224924	25.434	173	113	6	63	226	7	172	3.85E-10	56.5	COG2013	COG2013	Uncharacterized conserved protein
LN02_01609|LN02Chr01:6469686-6471948(-)|660	CDD:223608	27.891	441	311	5	213	648	9	447	2.51E-86	275	COG0534	NorM	Na+-driven multidrug efflux pump

LN02_01673|LN02Chr01:6706731-6708131(+)|447	CDD:223554	37.612	335	177	6	1	334	1	304	2.67E-109	322	COG0478	COG0478	RIO-like serine/threonine protein kinase fused to N-terminal HTH domain
LN02_02121|LN02Chr02:672674-674432(+)|549	CDD:223944	37.866	478	282	7	57	531	4	469	1.12E-140	412	COG1012	PutA	NAD-dependent aldehyde dehydrogenases
LN02_02441|LN02Chr02:1824536-1827124(-)|862	CDD:226582	21.858	366	212	20	211	546	44	365	1.31E-14	74.1	COG4097	COG4097	Predicted ferric reductase
LN02_02505|LN02Chr02:2048237-2050249(-)|627	CDD:223086	37.063	286	135	4	305	590	2	242	1.27E-70	225	COG0007	CysG	Uroporphyrinogen-III methylase
LN02_02505|LN02Chr02:2048237-2050249(-)|627	CDD:224562	30.233	129	70	4	63	190	14	123	7.42E-10	56.2	COG1648	CysG	Siroheme synthase (precorrin-2 oxidase/ferrochelatase domain)
LN02_02761|LN02Chr02:2878329-2880785(-)|552	CDD:223134	65.354	508	168	1	44	551	2	501	0	888	COG0056	AtpA	F0F1-type ATP synthase, alpha subunit
LN02_02953|LN02Chr02:3578908-3581165(+)|657	CDD:223589	29.132	357	218	10	9	335	1	352	9.87E-52	180	COG0515	SPS1	Serine/threonine protein kinase
LN02_03017|LN02Chr02:3768362-3770368(+)|668	CDD:227492	37.27	652	331	8	3	654	2	575	0	539	COG5163	NOP7	Protein required for biogenesis of the 60S ribosomal subunit
LN02_03209|LN02Chr02:4502228-4503905(+)|442	CDD:224717	46.667	405	200	5	38	441	7	396	5.66E-164	464	COG1804	CaiB	Predicted acyl-CoA transferases/carnitine dehydratase
LN02_03273|LN02Chr02:4706961-4708395(+)|355	CDD:223589	23.41	346	227	10	23	355	2	322	6.51E-42	147	COG0515	SPS1	Serine/threonine protein kinase
LN02_03401|LN02Chr02:5102683-5104004(+)|289	CDD:225240	30.172	116	72	2	179	285	134	249	1.22E-08	51.7	COG2365	COG2365	Protein tyrosine/serine phosphatase
LN02_03593|LN02Chr02:6454074-6457241(+)|1002	CDD:226406	26.957	115	70	4	134	240	739	847	5.60E-05	44.1	COG3889	COG3889	Predicted solute binding protein
LN02_03657|LN02Chr03:146155-148969(-)|865	CDD:224322	28.713	202	116	7	150	339	138	323	6.86E-12	65.7	COG1404	AprE	Subtilisin-like serine proteases
LN02_03785|LN02Chr03:746654-747757(-)|367	CDD:225546	29.064	203	130	5	142	344	45	233	2.19E-17	78.3	COG3000	ERG3	Sterol desaturase
LN02_03977|LN02Chr03:1314251-1315062(-)|251	CDD:223129	37.179	78	49	0	91	168	8	85	1.06E-19	78.8	COG0051	RpsJ	Ribosomal protein S10
LN02_04041|LN02Chr03:1571406-1572731(-)|387	CDD:223991	35.18	361	212	9	29	380	2	349	6.07E-97	290	COG1063	Tdh	Threonine dehydrogenase and related Zn-dependent dehydrogenases
LN02_04169|LN02Chr03:2952702-2954844(-)|482	CDD:223341	39.815	432	188	9	34	462	1	363	8.13E-155	442	COG0263	ProB	Glutamate 5-kinase
LN02_04233|LN02Chr03:3455473-3457568(+)|552	CDD:225748	30	340	192	8	188	526	21	315	4.73E-63	207	COG3207	DIT1	Pyoverdine/dityrosine biosynthesis protein
LN02_04361|LN02Chr03:3913397-3914683(+)|386	CDD:223475	23.497	183	128	4	121	301	51	223	1.93E-14	68.9	COG0398	COG0398	Uncharacterized conserved protein
LN02_04489|LN02Chr03:4324458-4326568(+)|564	CDD:224990	39.583	480	259	12	78	556	4	453	8.50E-150	435	COG2079	PrpD	Uncharacterized protein involved in propionate catabolism
LN02_04617|LN02Chr03:4818850-4821691(+)|845	CDD:227670	25.915	355	228	13	433	779	41	368	1.44E-26	110	COG5379	BtaA	S-adenosylmethionine:diacylglycerol 3-amino-3-carboxypropyl transferase
LN02_04617|LN02Chr03:4818850-4821691(+)|845	CDD:225136	24.444	135	69	4	131	264	55	157	3.19E-08	52.3	COG2226	UbiE	Methylase involved in ubiquinone/menaquinone biosynthesis

LN02_04745|LN02Chr03:5252296-5253387(+)|224	CDD:227400	32.683	205	94	5	56	222	83	281	3.23E-29	110	COG5068	ARG80	Regulator of arginine metabolism and related MADS box-containing transcription factors
LN02_05001|LN02Chr04:313558-314299(+)|193	CDD:225039	24.859	177	122	6	9	179	2	173	7.51E-14	63.7	COG2128	COG2128	Uncharacterized conserved protein
LN02_05065|LN02Chr04:499725-503160(+)|1115	CDD:224037	25.29	775	540	13	128	887	11	761	6.19E-121	387	COG1112	COG1112	Superfamily I DNA and RNA helicases and helicase subunits
LN02_05129|LN02Chr04:698669-700734(-)|602	CDD:224630	29.293	99	57	4	20	118	71	156	1.67E-08	51.9	COG1716	COG1716	FOG: FHA domain
LN02_05257|LN02Chr04:1136800-1138374(-)|524	CDD:223638	30.466	279	137	6	130	400	34	263	8.41E-68	217	COG0564	RluA	Pseudouridylate synthases, 23S RNA-specific
LN02_05577|LN02Chr04:2509719-2511594(+)|593	CDD:227509	31.298	393	223	10	5	396	13	359	1.74E-83	265	COG5182	CUS1	Splicing factor 3b, subunit 2
LN02_05641|LN02Chr04:2727305-2728663(+)|396	CDD:224825	35.556	45	27	1	179	223	83	125	6.13E-04	37.4	COG1913	COG1913	Predicted Zn-dependent proteases
LN02_05705|LN02Chr04:2936604-2938708(+)|617	CDD:223562	37.616	537	313	10	84	610	3	527	0	524	COG0488	Uup	ATPase components of ABC transporters with duplicated ATPase domains
LN02_05961|LN02Chr04:3833431-3835275(-)|614	CDD:227511	19.361	501	266	22	100	557	36	441	1.36E-18	86	COG5184	ATS1	Alpha-tubulin suppressor and related RCC1 domain-containing proteins
LN02_06025|LN02Chr04:4036253-4038016(-)|550	CDD:224983	17.647	238	152	9	175	400	78	283	5.30E-06	45.9	COG2072	TrkA	Predicted flavoprotein involved in K+ transport
LN02_06025|LN02Chr04:4036253-4038016(-)|550	CDD:223717	63.636	22	8	0	9	30	4	25	1.22E-05	44.8	COG0644	FixC	Dehydrogenases (flavoproteins)
LN02_06025|LN02Chr04:4036253-4038016(-)|550	CDD:223523	27.536	69	41	2	11	76	1	63	1.00E-04	41.8	COG0446	HcaD	Uncharacterized NAD(FAD)-dependent dehydrogenases
LN02_06281|LN02Chr04:4900161-4901612(-)|483	CDD:224169	35.169	472	281	8	4	473	5	453	8.23E-137	399	COG1249	Lpd	Pyruvate/2-oxoglutarate dehydrogenase complex, dihydrolipoamide dehydrogenase (E3) component, and related enzymes
LN02_06473|LN02Chr04:5497260-5499548(+)|720	CDD:223589	26.996	263	136	7	378	628	7	225	1.04E-30	121	COG0515	SPS1	Serine/threonine protein kinase
LN02_06601|LN02Chr05:90564-91528(+)|287	CDD:223377	27.626	257	161	10	33	282	22	260	9.65E-36	126	COG0300	DltE	Short-chain dehydrogenases of various substrate specificities
LN02_06793|LN02Chr05:828202-835532(-)|1934	CDD:223370	28.049	246	82	5	1681	1881	9	204	1.34E-40	146	COG0293	FtsJ	23S rRNA methylase
LN02_06921|LN02Chr05:1383890-1385405(+)|451	CDD:223560	55.556	63	27	1	2	64	6	67	4.70E-24	100	COG0484	DnaJ	DnaJ-class molecular chaperone with C-terminal Zn finger domain
LN02_06921|LN02Chr05:1383890-1385405(+)|451	CDD:227594	24.26	169	85	5	12	179	58	184	2.63E-07	49.3	COG5269	ZUO1	Ribosome-associated chaperone zuotin
LN02_06985|LN02Chr05:1869457-1874202(+)|1407	CDD:223494	29.961	761	429	24	441	1175	106	788	3.81E-130	419	COG0417	PolB	DNA polymerase elongation subunit (family B)
LN02_07113|LN02Chr05:2603265-2606661(+)|976	CDD:227365	21.809	807	517	29	231	976	1347	2100	2.74E-75	268	COG5032	TEL1	Phosphatidylinositol kinase and protein kinases of the PI-3 kinase family

LN02_07177 | LN02Chr05 :2824013-2825534(-) | 477	CDD:2235 52	31.214	173	113	3	99	268	1	170	2.59E-38	137	COG04 76	ThiF	Dinucleotide-utilizing enzymes involved in molybdopterin and thiamine biosynthesis family 2
LN02_07497 | LN02Chr05 :3976764-3978202(+) | 446	CDD:2230 98	27.047	403	251	10	43	425	7	386	5.96E-88	270	COG00 19	LysA	Diaminopimelate decarboxylase
LN02_07561 | LN02Chr05 :4162534-4163947(-) | 394	CDD:2231 60	46.891	386	172	5	1	386	2	354	9.30E-170	476	COG00 82	AroC	Chorismate synthase
LN02_07753 | LN02Chr05 :5084050-5085446(-) | 375	CDD:2235 60	25.576	434	175	9	2	374	1	347	1.15E-63	205	COG04 84	DnaJ	DnaJ-class molecular chaperone with C-terminal Zn finger domain
LN02_08137 | LN02Chr06 :1388662-1389749(+) | 328	CDD:2232 47	28.276	290	186	11	29	315	10	280	3.68E-51	168	COG01 69	AroE	Shikimate 5-dehydrogenase
LN02_08265 | LN02Chr06 :1934928-1935612(-) | 188	CDD:2237 35	27.711	166	100	3	1	166	2	147	4.88E-16	69.5	COG06 63	PaaY	Carbonic anhydrases/acetyltransferases, isoleucine patch superfamily
LN02_08329 | LN02Chr06 :2126164-2127948(+) | 455	CDD:2273 69	19.745	157	97	3	1	157	1	128	2.62E-09	56	COG50 36	COG503 6	SPX domain-containing protein involved in vacuolar polyphosphate accumulation
LN02_08393 | LN02Chr06 :2443022-2443612(-) | 116	CDD:2231 29	41.748	103	57	2	15	115	2	103	8.71E-33	108	COG00 51	RpsJ	Ribosomal protein S10
LN02_08777 | LN02Chr07 :319021-321940(-) | 888	CDD:2241 20	16.569	851	470	23	15	850	6	631	5.49E-61	216	COG11 99	DinG	Rad3-related DNA helicases
LN02_08969 | LN02Chr07 :997810-998754(-) | 255	CDD:2231 74	21.25	160	90	5	96	254	7	131	7.78E-17	71.8	COG00 96	RpsH	Ribosomal protein S8
LN02_09033 | LN02Chr07 :1197052-1198594(+) | 468	CDD:2260 39	52.273	440	190	8	10	449	1	420	8.44E-167	474	COG35 08	HmgA	Homogentisate 1,2-dioxygenase
LN02_00330 | LN02Chr01 :1797574-1799739(+) | 629	CDD:2239 36	36.889	450	213	12	37	477	3	390	2.23E-98	304	COG10 04	Ugd	Predicted UDP-glucose 6-dehydrogenase
LN02_00394 | LN02Chr01 :2002583-2004316(-) | 449	CDD:2249 24	35.455	220	129	6	235	445	8	223	1.94E-51	170	COG20 13	COG201 3	Uncharacterized conserved protein
LN02_00714 | LN02Chr01 :3135903-3139418(-) | 1129	CDD:2235 50	34.087	1062	518	22	7	1054	24	917	0	749	COG04 74	MgtA	Cation transport ATPase
LN02_00842 | LN02Chr01 :3560618-3563057(-) | 766	CDD:2236 93	57.402	331	138	1	431	761	1	328	8.34E-156	453	COG06 20	MetE	Methionine synthase II (cobalamin-independent)
LN02_00842 | LN02Chr01 :3560618-3563057(-) | 766	CDD:2236 93	22.222	351	209	16	9	342	13	316	9.12E-11	61.2	COG06 20	MetE	Methionine synthase II (cobalamin-independent)
LN02_01098 | LN02Chr01 :4419491-4423269(-) | 1041	CDD:2236 27	18.489	887	498	20	134	999	173	855	1.27E-39	156	COG05 53	HepA	Superfamily II DNA/RNA helicases, SNF2 family
LN02_01290 | LN02Chr01 :5161827-5162909(+) | 360	CDD:2275 45	27.829	327	211	5	26	349	9	313	1.89E-51	170	COG52 20	TFB3	Cdk activating kinase (CAK)/RNA polymerase II transcription initiation/nucleotide excision repair factor TFIIH, subunit TFB3
LN02_01482 | LN02Chr01 :5957900-5960409(+) | 572	CDD:2234 82	38.122	543	302	14	34	563	10	531	7.60E-155	452	COG04 05	Ggt	Gamma-glutamyltransferase
LN02_01546 | LN02Chr01 :6218468-6220659(+) | 674	CDD:2273 92	35.312	337	194	9	29	354	31	354	2.40E-63	217	COG50 59	KIP1	Kinesin-like protein
LN02_01674 | LN02Chr01 :6708576-6710510(-) | 539	CDD:2237 96	21.393	201	131	4	225	398	97	297	2.71E-12	65	COG07 24	COG072 4	RNA-binding proteins (RRM domain)

LN02_01866|LN02Chr01:7253549-7254824(-)|380	CDD:225143	20.408	245	134	13	117	355	211	400	3.58E-06	45.7	COG2234	Iap	Predicted aminopeptidases
LN02_02122|LN02Chr02:675379-677647(-)|666	CDD:223727	22.067	358	245	11	59	406	4	337	3.38E-45	162	COG0654	UbiH	2-polyprenyl-6-methoxyphenol hydroxylase and related FAD-dependent oxidoreductases
LN02_02378|LN02Chr02:1639128-1640068(+)|235	CDD:224584	23.711	97	74	0	111	207	71	167	2.92E-10	55	COG1670	RimL	Acetyltransferases, including N-acetylases of ribosomal proteins
LN02_02442|LN02Chr02:1829313-1830329(+)|338	CDD:225034	29.078	282	145	7	36	317	24	250	5.17E-34	123	COG2123	COG2123	RNase PH-related exoribonuclease
LN02_02506|LN02Chr02:2050964-2053543(-)|859	CDD:227746	29.114	395	215	16	340	730	76	409	4.93E-48	175	COG5459	COG5459	Predicted rRNA methylase
LN02_02762|LN02Chr02:2881828-2882473(-)|173	CDD:223172	38.854	157	86	3	8	154	24	180	4.64E-58	177	COG0094	RplE	Ribosomal protein L5
LN02_02826|LN02Chr02:3139698-3140396(-)|187	CDD:226007	35.669	157	93	2	19	175	9	157	1.49E-28	101	COG3476	COG3476	Tryptophan-rich sensory protein (mitochondrial benzodiazepine receptor homolog)
LN02_02890|LN02Chr02:3364524-3369464(-)|1585	CDD:226406	26.415	106	66	4	479	574	739	842	2.27E-04	42.9	COG3889	COG3889	Predicted solute binding protein
LN02_02954|LN02Chr02:3582612-3586756(+)|1231	CDD:224117	18.807	218	153	4	996	1212	178	372	1.12E-08	56.6	COG1196	Smc	Chromosome segregation ATPases
LN02_03082|LN02Chr02:4035708-4036767(-)|268	CDD:223711	42.918	233	124	5	3	230	1	229	3.41E-62	193	COG0638	PRE1	20S proteasome, alpha and beta subunits
LN02_03274|LN02Chr02:4710077-4711063(+)|277	CDD:227471	32.444	225	119	3	54	276	19	212	1.44E-57	180	COG5142	OXR1	Oxidation resistance protein
LN02_03338|LN02Chr02:4912012-4915047(-)|970	CDD:223569	41.371	875	386	25	58	915	8	772	0	813	COG0495	LeuS	Leucyl-tRNA synthetase
LN02_03402|LN02Chr02:5104082-5105432(-)|364	CDD:223574	19.444	252	194	4	110	359	11	255	4.27E-10	56.8	COG0500	SmtA	SAM-dependent methyltransferases
LN02_03722|LN02Chr03:435482-437716(+)|744	CDD:227721	41.176	51	21	2	31	81	84	125	4.70E-05	43.6	COG5434	PGU1	Endopygalactorunase
LN02_03786|LN02Chr03:748531-749601(+)|356	CDD:223377	27.652	264	166	6	99	347	6	259	2.61E-41	142	COG0300	DltE	Short-chain dehydrogenases of various substrate specificities
LN02_03850|LN02Chr03:929331-931190(+)|549	CDD:223505	30.588	170	105	3	377	541	101	262	2.77E-25	102	COG0428	COG0428	Predicted divalent heavy-metal cations transporter
LN02_04042|LN02Chr03:1575989-1579683(+)|1208	CDD:223715	24.08	299	158	9	715	1010	93	325	6.84E-37	139	COG0642	BaeS	Signal transduction histidine kinase
LN02_04042|LN02Chr03:1575989-1579683(+)|1208	CDD:223855	29.655	145	84	4	1066	1208	2	130	4.80E-28	107	COG0784	CheY	FOG: CheY-like receiver
LN02_04042|LN02Chr03:1575989-1579683(+)|1208	CDD:225112	25.532	141	98	4	580	720	99	232	1.22E-06	48.3	COG2202	AtoS	FOG: PAS/PAC domain
LN02_04234|LN02Chr03:3457805-3459790(-)|550	CDD:225179	17.936	407	279	13	102	496	50	413	4.38E-09	55.8	COG2270	COG2270	Permeases of the major facilitator superfamily
LN02_04298|LN02Chr03:3708606-3710388(-)|547	CDD:225490	42.237	438	223	14	133	543	56	490	5.78E-152	442	COG2939	COG2939	Carboxypeptidase C (cathepsin A)

LN02_04554∣LN02Chr03:4557405-4559215(+)∣540	CDD:223760	17.91	402	159	10	137	532	3	239	1.10E-40	144	COG0688	Psd	Phosphatidylserine decarboxylase
LN02_04682∣LN02Chr03:5059488-5064719(-)∣1698	CDD:224055	29.63	567	349	9	1118	1675	41	566	1.38E-105	347	COG1132	MdlB	ABC-type multidrug transport system, ATPase and permease components
LN02_04682∣LN02Chr03:5059488-5064719(-)∣1698	CDD:224055	25.847	472	326	9	478	946	104	554	3.14E-57	206	COG1132	MdlB	ABC-type multidrug transport system, ATPase and permease components
LN02_04810∣LN02Chr03:5485147-5487096(+)∣649	CDD:224780	31.693	508	210	12	22	525	6	380	3.46E-110	334	COG1867	TRM1	N2,N2-dimethylguanosine tRNA methyltransferase
LN02_04874∣LN02Chr03:5838511-5841198(+)∣827	CDD:223556	32.468	770	421	17	51	819	8	679	2.43E-170	506	COG0480	FusA	Translation elongation factors (GTPases)
LN02_05322∣LN02Chr04:1370222-1373668(+)∣992	CDD:227381	19.313	233	163	7	1	227	148	361	7.54E-04	40.1	COG5048	COG5048	FOG: Zn-finger
LN02_05386∣LN02Chr04:1574057-1578818(+)∣1497	CDD:224666	33	300	183	6	1182	1472	2	292	7.38E-72	240	COG1752	RssA	Predicted esterase of the alpha-beta hydrolase superfamily
LN02_05386∣LN02Chr04:1574057-1578818(+)∣1497	CDD:223736	31.707	123	83	1	819	940	11	133	1.78E-17	79.9	COG0664	Crp	cAMP-binding proteins - catabolite gene activator and regulatory subunit of cAMP-dependent protein kinases
LN02_05386∣LN02Chr04:1574057-1578818(+)∣1497	CDD:223736	20.93	172	93	3	661	824	1	137	3.77E-09	55.6	COG0664	Crp	cAMP-binding proteins - catabolite gene activator and regulatory subunit of cAMP-dependent protein kinases
LN02_06026∣LN02Chr04:4039091-4039465(-)∣70	CDD:225292	32.812	64	43	0	3	66	2	65	5.09E-18	67.4	COG2443	Sss1	Preprotein translocase subunit Sss1
LN02_06218∣LN02Chr04:4702372-4704767(+)∣747	CDD:223562	37.77	556	306	9	194	737	3	530	0	554	COG0488	Uup	ATPase components of ABC transporters with duplicated ATPase domains
LN02_06282∣LN02Chr04:4902123-4905599(-)∣1085	CDD:224361	30.619	921	462	19	20	937	1	747	0	646	COG1444	COG1444	Predicted P-loop ATPase fused to an acetyltransferase
LN02_06602∣LN02Chr05:93276-95239(-)∣634	CDD:224983	27.865	384	235	12	214	583	9	364	1.11E-59	203	COG2072	TrkA	Predicted flavoprotein involved in K+ transport
LN02_06730∣LN02Chr05:619316-620318(+)∣306	CDD:225616	25.758	66	46	1	58	123	10	72	5.86E-05	38	COG3074	COG3074	Uncharacterized protein conserved in bacteria

LN02_06794∣LN02Chr05:836584-838095(-)∣323	CDD:223538	54.747	316	140	3	5	319	1	314	9.39e-161	448	COG0462	PrsA	Phosphoribosylpyrophosphate synthetase
LN02_07242∣LN02Chr05:3069491-3071464(-)∣577	CDD:225181	30.040	506	299	17	27	529	8	461	1.81e-69	229	COG2272	PnbA	Carboxylesterase type B
LN02_07306∣LN02Chr05:3280198-3281958(+)∣457	CDD:227823	32.922	243	125	7	202	429	20	239	9.00e-45	156	COG5536	BET4	Protein prenyltransferase, alpha subunit
LN02_07434∣LN02Chr05:3776334-3777683(+)∣449	CDD:223573	58.998	439	160	3	4	441	1	420	0.0	685	COG0499	SAM1	S-adenosylhomocysteine hydrolase

LN02_07498|LN02 Chr05:3979131-3980494(+)|230	CDD:225003	34.091	88	57	1	143	230	2	88	3.02e-27	97.4	COG2092	EFB1	Translation elongation factor EF-1beta
LN02_07754|LN02 Chr05:5097592-5101287(-)|1204	CDD:226406	20.856	187	117	8	247	420	694	862	4.72e-04	41.4	COG3889	COG3889	Predicted solute binding protein
LN02_07882|LN02 Chr05:5587173-5588328(-)|309	CDD:223796	30.526	95	62	3	8	100	108	200	1.11e-09	55.3	COG0724	COG0724	RNA-binding proteins (RRM domain)
LN02_07882|LN02 Chr05:5587173-5588328(-)|309	CDD:223796	24.590	122	80	5	165	283	111	223	5.46e-07	47.2	COG0724	COG0724	RNA-binding proteins (RRM domain)
LN02_07946|LN02 Chr05:5763443-5766004(-)|853	CDD:224418	28.927	643	378	23	137	776	143	709	1.97e-119	377	COG1501	COG1501	Alpha-glucosidases, family 31 of glycosyl hydrolases
LN02_08266|LN02 Chr06:1936191-1937783(-)|530	CDD:225201	27.636	275	186	8	42	307	155	425	7.37e-27	110	COG2319	COG2319	FOG: WD40 repeat
LN02_08522|LN02 Chr06:2864687-2865584(-)|263	CDD:225371	22.330	103	80	0	147	249	43	145	2.36e-08	51.1	COG2814	AraJ	Arabinose efflux permease
LN02_08650|LN02 Chr06:3298458-3300223(+)|442	CDD:223727	381	24.934	237	14	1	369	2	345	3.22e-38	139	COG0654	UbiH	2-polyprenyl-6-methoxyphenol hydroxylase and related FAD-dependent oxidoreductases
LN02_08714|LN02 Chr06:3665145-3665921(+)|186	CDD:224025	29.545	132	88	2	13	139	1	132	2.47e-30	107	COG1100	COG1100	GTPase SAR1 and related small G proteins
LN02_08778|LN02 Chr07:322288-323206(+)|149	CDD:223278	25.316	158	98	8	4	149	3	152	7.80e-20	77.7	COG0200	RplO	Ribosomal protein L15
LN02_08906|LN02 Chr07:760858-761559(-)|233	CDD:223568	28.378	148	99	3	59	204	11	153	4.42e-11	56.8	COG0494	MutT	NTP pyrophosphohydrolases including oxidative damage repair enzymes
LN02_08970|LN02 Chr07:999006-1000756(-)|497	CDD:223727	24.235	392	240	12	8	396	6	343	2.07e-41	149	COG0654	UbiH	2-polyprenyl-6-methoxyphenol hydroxylase and related FAD-dependent oxidoreductases

Table S7. The deprotonated peak [M-H]$^-$ (*m*/*z*) in HRESIMS spectra and molecular formula of the sorbicillinoids isolated from albino strain LN02.

**Compd. No.**	**RT (min) in HPLC**	**Srobicillinoid**	**[M-H]$^-$ (*m*/*z*)**	**Formula**
**1**	20.0	Trichotetronine	495.2151	$C_{28}H_{31}O_8$
**2**	19.6	Demethyltrichodimerol	481.1883	$C_{27}H_{29}O_8$
*	18.7	Unidentified	495.2084	$C_{28}H_{31}O_8$
**3**	17.7	Dihydrotrichodimer ether A	495.2041	$C_{28}H_{31}O_8$
**4**	17.3	Bisorbicillinol	495.2143	$C_{28}H_{31}O_8$
*	15.9	Unidentified	529.2152	$C_{28}H_{33}O_{10}$

Note: Compounds were detected after 30 days of growth in PDA medium at 28 °C. The mass-to-charge ratio (*m*/*z*) of the protonated metabolites, their empirical formulas and retention time (RT, min) were indicated. (*) indicated the unknown sorbicillinoid.

Table S8. ^{1}H NMR and ^{13}C NMR data of the sorbicillinoids isolated from albino strain LN02.

Position	Trichotetronine (1, CD_3OD)		Demethyltrichodimerol (2, CD_3OD)		Dihydrotrichodimer ether A (3, CD_3COCD_3)		Bisorbicillinol (4, CD_3OD)	
	δ_C	δ_H mult. (*J* in Hz)	δ_C	δ_H mult. (*J* in Hz)	δ_C	δ_H mult. (*J* in Hz)	δ_C	δ_H mult. (*J* in Hz)
1	63.5		48.4	3.64 d (12.6)	58.9	3.11 s	nd	3.65 d (1.9)
2	197.5		105.2		79.4		110.4	
3	110.0		201.2		105.3		198.0	
4	43.4	3.35 s	61.6		60.5		nd	
5	75.8		105.8		201.9		209.2	
6	210.1		82.0		105.6		75.3	
7	52.8	3.29 s	59.5	3.09 s	175.6		48.3	3.40 d (2.0)
8	43.9	3.31 s	105.0		120.6	6.50 d (14.8)	69.1	
9	169.7		198.9		142.9	7.32 dd (14.8, 11.0)	nd	
10	119.5	6.21-6.43 m	61.9	3.55 d (12.6)	132.0	6.41 ddd (14.8, 10.8, 1.2)	111.7	
11	143.9	7.31 dd (14.6, 11.0)	104.4		139.8	6.26 dq (15.2, 7.2)	nd	
12	131.6	6.21-6.43 m	80.1		18.9	1.88 d (6.6)	nd	
13	140.8	6.21-6.43 m	nd		22.2	1.34 s	169.7	
14	18.9	1.90 d (6.3)	120.3	6.18-6.42 m	19.6	1.31 s	120.5	
15	202.3		144.6	7.30 dd (14.6, 10.8)	54.0	3.19 s	142.8	7.24 d (15.4, 10.4)
16	128.4	6.21-6.43 m	132.2	6.18-6.42 m	79.2		132.5	6.15-6.40 m
17	147.8	7.20 dd (10.3, 15.2)	140.6	1.89 d (2.72)	105.0		139.8	6.15-6.40 m
18	132.3	6.21-6.43 m	18.9		56.4		18.9	1.88 d (6.1)
19	145.1	6.21-6.43 m	176.8		173.5		199.1	
20	19.1	1.90 d (6.3)	120.1	6.18-6.42 m	109.6		125.8	6.40 d (14.7)
21	84.4		144.0	7.30 dd (14.6, 10.8)	189.0		146.6	7.21 dd (14.7, 10.6)

22	179.2		132.3	6.18-6.42 m	41.6	2.28 dd (16.7, 3.2)	131.6	6.08 m
23	97.7		141.2		81.3	4.32 ddd (13.2, 6.8, 3.2)	143.7	5.95 m
24	176.8		19.0	1.89 d (2.72)	128.8	5.57 ddd (15.2, 6.8, 1.6)	19.0	1.87 d (5.7)
25					131.5	5.75 ddq(*J* = 15.2, 1.2, 6.4)		
26					17.9	1.66 dd (6.4, 0.8)		
27					21.6	1.24 s		
28					19.4	1.41 s		
1-CH_3	24.2	1.00 s	/	/			/	/
4-CH_3	/	/	20.1	1.37 s			8.8	1.41 s
5-CH_3	11.3	1.18 s	/	/			/	/
6-CH_3	/	/	21.1	1.36 s			25.2	1.13 s
10-CH_3	/	/	/	/			10.7	1.60 s
12-CH_3	/	/	21.0	1.36 (s)			nd	1.20 s
21-CH_3	23.5	1.42 s	/	/			/	/
23-CH_3	6.5	1.52 s	/	/			/	/

Note: ^{1}H NMR spectra were measured at 400 MHz, and ^{13}C NMR spectra at 100 MHz. Nd: Not detected. The carbon atoms were numbered which were shown in Figure S3.

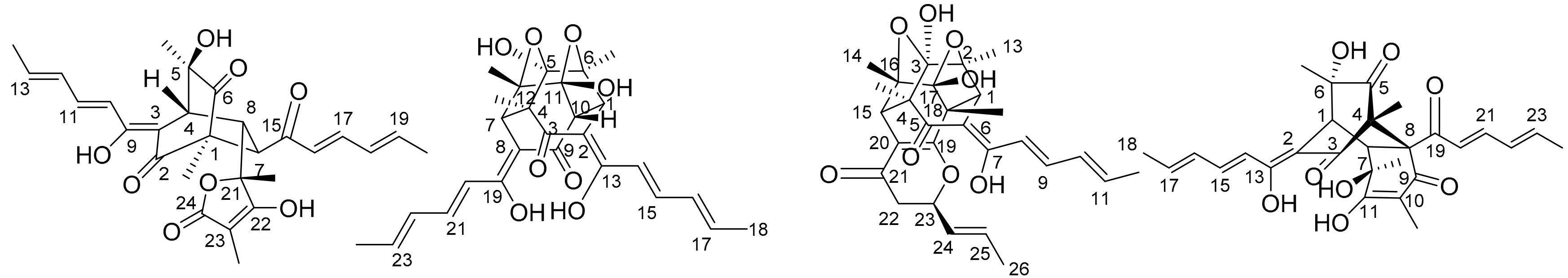

Trichotetronine (**1**) Demethyltrichodimerol (**2**) Dihydrotrichodimer ether A (**3**) Bisorbicillinol (**4**)

Figure S3. Structures of four identified sorbicillinoids (**1**–**4**) with carbon atoms numbered in strain LN02.

Table S9. Sequences of the cloned gene *uvpks1* from *V. virens* (*U. virens*) P1, *uvpks1* promoter from *V. virens* (*U. virens*) P1, and *uvpks1* promoter from *V. virens* (*U. virens*) LN02.

>*uvpks1* cDNA *Ustilaginoidea virens* P1

ATGGCGAACGTGTTCCAAATTGCCGTCTTCGGCGATCTGTCTGTTCCGTACCACTCGGAGCT
CCGCAGACTCTTCTCTGAAAAGAGAGACTATGTCTTGGCTACCCTCTTCACCAAGTCGTATT
ACGCCGTCAAGTCGGAGATTTCTCGACTGCCCCCCAGCCAGAGGGCCCAGTTCCCATTTTC
GTCCAACATTGAGGAGCTGTTGAACGCTGACAAGGAATCGACCACGTCCAACTATGCGCT
GGACAGCTTCTTTTTCTGCCTCTGTCAGATATCGTCCTTTGTCTCCCACCTTAATCGATCCGG
GACGTCGTATCCCCGTGCATCTTCTTCTTGCCTGGCGAGCCGATGCATCGGCCTGCTCGCTG
CCGTGGCCATCAGCTGCAGCGAGAACGTGTATGACTTGGTCTCGATAGCCCCCGAGGTTGT
CGCTTTGGCCTTTAGGGTCGGGCTCCTGGTGCAGGGCAAAACCAAATCCGTCACCCTCAGC
TCCGGCAATGGCGCATCGTGCTCGACCGTGATAGCTGGCCTTGACGAGCCTGCCGCGTCGC
AGCTGCTCAACGCCTACTTCGACAACAAGGGCGCACCCGCCCTCAGCCGAGTCTACGTCA
GCGCCGTGGGCAGCGGCACAATCACGCTCTCCGGCCCTCCTGCCCAGTTGAAGGAGTTCTT
GTCCCACCACAGCGACCTCAAGGCCGGAAAGATCCAGGTTGGCGGCCTGTTCCACTCGCC
GAGCCTTTACACCGACGCCGATGTGTCGGGTCTGGTCGCCTCGGCGACTGCGCACCTGCGG
GGCAGAGTCGCCCGCATCCCCGTCATCTTGAACGGGCACGAGAAGCAGCAGGAACTCGTG
GGTGGGGAGACGTGCCAGCACCTCCTCGAGGTTGTCGTCTCCGACATTCTCCGCCACCAGA
TGAGGTGGGATCTTGCGGCCGAAAGAGTCATCCGCGCCATCAGACGCTCGGGCTGCTCGG
CTGTCGAGCTCCTCCCCTTCGTGGCGGGAAGCGTCGAGGGCCTGTCGTCTGTCCTTCGCGCC
ACCATGGGCATCGACCGCGTGGACGTGCCCAACACGGCTGGCGTGGACTCTTCCTCGCGC
GGCAGCGGCCATGCCGACGCCGAGAAGCAGCCCGCCAGGTCCAAGATTGCCATCATTGGC
TTCTCCGGCCGGTATCCCGAGGCCGAGACAATGAGGAGTTCTGGGAGCTCCTGGCCGCAG
GACTCGACGTCCACAGGGAGATTCCCAAGGAACGATTCGACCCCTACCTGTACTTTGACCC
GACCTGCAAAAAGAAGAACACCAGCGGCGTCACAAAGGGCTGTTTCGTCCGCAACCCCG
ACCTCTTCGACTCGAGGTTCTTCAGCATGTCGCCCAGAGAGGCGGACCAGGCGGATCCCGC
CCAGAGGTTTGCCTTGATGACGGCATACGAGGCCATGGAGATGGCGGGATTCGTGCCGGA
CTCGACGCCGTCCTCGCAGAGGAGTCGCGTGGGCGTCTTTTACGGCACCGCCAGCGACGA
CTACCGCGAGATCAACGCCGCGCAAAACGTCGACACGTACTTCGTCCCCGGCGGGAGCCG
CGCCTTCCTGCCGGCCCGCATCAACTACCACTTCCGATTCAGCGGGCCGTCGTTCGACGTC
GACACGGCGTGCTCGTCCGGCCTCGCTGCCGTCCACATCGCCTGCAACTCGCTGTGGGCCG
AGGACTGCGACGTCGCCATCGCGGGAGGCACAAACATCCTCACCAACCCCGACAACTGGG
CCGGCCTGGACCGGGCGCACTTCCTGTCGCGCACCGGCAACTGCAACACCTTTGACGACG
CCGCCGACGGCTACTGCAGGTCCGACACTGTCGCCACAGTCATCTTGAAGAGGCTCGAGG
ATGCTCTGCTGGATGGGGATCCGGTGTTCGGAACCATCTTGGGAGCCTACACCAACCATTC
CGCCGAAGCCGTGTCCATGACCCGGCCGCACTCGGGTGCCCAGAGGGCCATTTGCACCGC
ATCCTCCGATCCTCCAACGTCGACTGCTCCGAGGTCAGCTACGTGGAGATGCACGGAACG
GGAACCCAGCACGGAGACGCTACCGAAATGGACTCTGTCCTGAGCGTCTTTGCGCCCGAC
ACCACGTCGCGCAAGAGCCCTCTGTTCATCGGTTCGGTCAAGGCCAACGTCGGGCATGCCG
AGTCGGCCGCTGGCATCTCGTCGCTGGTCAAGGTCCTTCTCATGATGCAGAAGAACGCCAC
CCGCGCCACGTCGGCATCAAGACGAAGCTCAACAGGAACTTCCCCAAGGATCTTGTCCAG
CGAAACGTCCACATCTCCCTGGAGAACAGGTCGTGGCCACGGCCCGACCCCAGGGTCGTC
CCGCACGGCAGGAGGGTCTTTATCAACAACTTTGGCGCCGCCGGCGGCAACTCGTCCGTCC
TGGTCGAGGACGCCCCCGTCAGGCCGGCGCCGGAGCGAGACGATGCCTCGTGGCCCGTCC
ACGCCGTGGCCGTCTCGGCAAAGACGCAGAACTCCTTCAAGGAAAACATCCGAGCGCTGA
TAGCGTATCTGGAGACGCGCCCCACGTCTCTCTCGGCTCACTGTCCTACACCACCACCGCC
CGCCGCATTCACTACAGCTACCGCACGGCGGTGGTCGGCTCGTCGGTTGATGAGATCCGGA
ATGCGCTGCATGACGTGGCAGCCAGAGAGAAGCACCTGTCCACGGCGGGTGGTGGACCTC
CCATCGGCTTCAGCTTCACCGGCCAGGGCTCTCAGTACCTGGGCATGGGCAAGAAGCTGCT

CTCCTTGCCGCAGTTCGAGTCGCTCCTCGCGGGCCTCGACGGCATCGTTCGCTTGCAGGGCT
TCCCGTCCATCCTGGACGTGGTGAGCGGCAAGGCCGAGACGCCGATTGAGGACATGAGCC
CCGTCAAGGTGCAGTTGGCGATAGCCTGCCTGGAGATGGCCCTGGGGAAGTTCTGGATCGC
TCTCGGCGTCGTCCCGCAGATTGTCGTCGGCCACAGCCTCGGCGAGTACGCGGCGCTGAAC
ATTGCCGGCGTGCTCTCCGACGCGGACACGATCCACCTCGTGGGGACCAGAGCTCGCCTCT
TGGAGAAGGCCTGCTCGATGGGCAGCCACTCCATGCTGCCGTCAAGGCATCGGCGGCCGA
GGCGTCGTCGCTGCGCTCGTCGTCTCACCCCGACCTGGACATTGCCTGCATCAACGGACCC
GAGGACACCGTGGTCGCTGGCTCCAACAGCCAGATCGAGGCCTTCAAGGACCTCCTCAAC
GGCAGGTCCGTCAAGTCCACCCAGGTCAAGGTCCAGTTCGCCTTCCACTCGGCCCAGGTCG
AGCCCATGCTCGAGGCTTTCCGACAAGCCTGCGGCGCAGTCGTCGTCAACGAGCCCAGCA
TCCCCGTCATCTCGCCGCTGCTGGGCCGCGTCATGAAGAGCGCCTCGGACATTGGCCGGT
CGGCGACTACCTGGCCCGTCACTCCGCGAAACCGTAAACTTTTGCGAAGGGGTTCTGTCAG
CCAGGAACTCGGGACTCATCCCCGACAAGATGATGTGGGTGGAAGTAGGACCTCACCCCA
TCTGCAGCAACATGCTTAGTCGACCCTGGGGTCCTCGACACAGACGATCCCCAGTCTGCGG
CGCGGAGAGGATGACTGCAAGATCTTTACTCCCGCGCTGGCGAAGCTCTACGACAGCGGA
CTGGCCATCAACTGGGGCGAGTACCACGCCGGCGCGCAGCAGACCAAGCAGGTGCTTCTC
CTGCCCTCGTACCGGTGGGAGCTGAAGAGCCACTGGATCCCGTACACGAACGACTGGTGC
CTGACCAAGGGCGACGCGCCAGCACCCCAGCTGCTGGCGCTGCCGGAGGCGGCGGCGGC
GGCGGCGGCGGCCGAGAGGAGACTCTTCACCACGTCTGTCCAGTACATCACGGCGGAGAG
TTACGGTGCCCAGGAGGCTTCCATGACGGCACGCACGGACGTGCAGCACCCCGACTTCAG
GGAGGTGCTGCTGGCGCACCAGGTCAACGGCCGGCCCGTCTGCTCCTCGGCAGTGTACGC
CGACATGGCCTACACCATGTTCTCGCGAATGCTGGAAAAGTCGTCGGTGCCCTTTGACAAG
TCGGACCTGGGGATCGAAGTGGCCGACATGGCCGCAGACAAGTCGCTCATCCTCAACGAC
GACCCGTCACCGCAGATGCTGGAGCTGAAGGCCAGCGTCAACTGGTCGACCAGGCAGGG
GTCCTTCTCCATGTCGAGCATCTCGTCAGCCGACGGGAAGCCGACGGCCAAACACGCAAA
GTGCTCGGGCTTCTTCACCGACAAGAGCCGGTGGAAGTCGGAATGGAAGCGACGCGATTT
CCTCGTCAAGTCGAGAATCCAGGAACTCCGCAGCTCGGTCCATGACGACTCGGGCTCCGTG
CACATGATCAAGACGGGCATGTTTTACAAGCTCTTCACCGCGCTCGTGGATTACCGCGATT
CCTTCAAGGGGTGCCGCGAGCTCGTCATGCGCTCGGCCGACCTGGAGTCGACCGCCAAGG
TCAGGTTCAACACGCCGGCGGGGACGGCGGACAAGTGGAAGCTGCCGCCGCACTGGCTCG
ACAGCTTGGGCCAAATCACGGGCTTCACGATGAACGGCAACGACGAAGTCGACTCCAAGA
ACCAGGTCTACATCAACCACGGGTGGGACAACATGAAGATTTGCGGCGTCTTGTCCGACC
AGACGACGTACAACACCTACCTCAAGATGCAGCCCAAGACAAGGGCTCGTACTGCGGCG
ACGTGTACATTTTCAACCAGGACATGGACGAGGTGGTTGCCGTGTACGAGGGCGTGACGTT
TGCTGCCGTGCAGAGAAAGGTGCTTGATCTGGTGCTTCCCAAGCCCAAGGCAGCGGCGCA
GTCAGGAGCAGCGGCTGCGGCTGCGGCTGCGGCTCCATCACAACGGCAGCAGCAGCAGC
AGCAGCAGCAGCAGCAGCAACCAGCACAGCCCGTTGCTGCCATCAGGAATCCGGAATGG
ACGACATGCCGCCGACCCTGGTTCCGTCGGAAAAGAAGGACGTGCCATCAGAGAAGCTCA
AGGTCATCATCGCGGAGGAAGTCGGGGCGTCCATTTCCGACGTGCAGGACGACGCGGAGC
TCGCTCCCTTGGGGGTGGATTCGCTGCTTGCGCTCACAATCTCGGATCGCATGCTCGAGGA
GCTGGGCCTGCGGGTCGACTCGAGCGCCTTCATCTCGTGCATACGGTTGCCGAGCTGGTGC
GGCACATACTGGGGTCGTCGACGCCGTCGTCCGACTCTGGCCCGGCAACGCCGTCCATCAC
GCCGCTGCAGGAGCCTGACTTTGGGACGTCTGCCCTGTCCGAGAGGATCGAGAGCGCATTC
GCGTCGGTGCAAGTCGAGTCGGACCGATGTTCGGACACGACGCAGTATGGAGACGAAAA
GGCGGACGCGGTGACCAAGTTCGCCAGTATCAAACCCCTGGAAGCAGTTGAGATCCCGCC
TGCGACCTCGGTCCTGCTGCAAGGCAACCCGCGGACTTGCACCAGGAAGGTATGGCTGTTC
CCCGACGGATCCGGCTCGGCTGCGTCGTACATGCCGCTGCCAGACGTGGATCCCGCCAAG
GTGGCCATCTACGGGCTCAGCAGCCCCTTCATCAAGCACACGGCCACGGCCAAGCCGTGC
CAGTTTGGCGAGATGACGGCGGCCTACGTGGCCGAGATGCGCCGTCGCCAGCCGTCCGGC
CCGTACTCGGTGGGAGGCTGGTCGGCCGGCGGGCTCTGCGCCTACGACGCCGCGCAGAGG
CTGGTGGCCGACGGGGAGACGGTCGACGCGCTGATCCTGATCGACAGCCCCAACCCCATC
GGGCTCAAGGAGCTGCCGCCGCGGCTGTACAACGAGCTGTCGAGACTCAACGTGTTCGGG

GCCGAGCCGGGGGCCAAGGTGCCGGAATGGCTGGTGCCGCACTTCAAGCTGTTTGCCGAC
ATCCTGGTGACGTGCAAGCTGCGTCCGTGGCAGGCGGCCAAGCCTCTGCCCGCGTGGGCG
CTCTGGGCGAGGAACGGCGTGGACGAGAACCAGACGATTGAGCGCTGGCCGAGCGACCC
GGAAAACATGACCTGGCTGCTCAACCGCCGCACGCAGGCCATTCTGGGGTGCAACGGGTG
GGACGAGCTGCTGGGCAAGAAGAACATCACCGTGGGCGTGGTCGAGGGAGCCCATCACTT
TAGTATGCTGAAGCAGCCGGCCGTGCCGCAGGTGAGCGATTTCCTGCGCACCATAATGGA
GTCTACCGGCGCGGGTATCTAA

>*uvpks1* promoter *Ustilaginoidea virens* P1

TACGGCGAAAGATGTCGAAACGAGTCCCGATTTGGGTTGGGCGTTTGGGGGGGGGGGGG
GGGAAGAAAGAAGAAAATTATTACACCAAGTCAGAGCAAAGAGGGGGGCCCGGGGGGG
GGGGGGGGGGGGGGGGGGGCGTTGAGGCCAAAAAGCTCAGTGTCCCAGAGAAAAGACGC
CAGCCAGTCCTGCGTCAAGACGTTTTGAGAGACAACTGGTTTGGGAAGCTTTGAGAGAAA
GTGAAGGGGGATTACGAAACAGCAGAGGAGGGAGATTTGGAGAAGATTGCGCGGCCTTTT
TGGGCTATTATGGGCGAGGCGGGAGATGGTACGTAAAGTACCTCTACTAATACGTGACGA
GATGTCGTCGAGACGGATCATAATGCTCTCATGGAAAACGCATTGCCGGCTCAAAATAGG
CCTCGTGTTTCACATTGGGCGACTCCGGGCAGCCTCTTACTCTCACACCATCACGAAGCAA
CATAAGACATCCACTCGACCCGGTTGGTCCAAGACAAGAAAGAGGAGAGAAAAAAAGCA
TACTTCTTACTAAGACACGGGGGCTTGGCTTCGTGCGTTGCAGTTGCAGTTGCAGTTGCAGT
TGCAGTTGCAGTGTCGAGAGATTGCCATCCCTGGACGGATCCAGAACAGAGATATGGAGA
CGATGGCTTTTTTCGGCCAAGAAGGCGTGTCTGGCGGCTGGGAGGATGAGGTGATACGCG
GCGCAACTAGTTGGCCCGCGTGAGCAGGGCGAGAGAGGGAGGACGTCGGGACCTGGTCG
CCGCCGCCGCCGCCGCCGCCGCCGCCGCCGCCGCCGCCGTCGTCGTGGTCGTGGTCGT

>*uvpks1* promoter *Ustilaginoidea virens* LN02

TACGGCGAAAGATGTCGAAACGAGTCCCGATTTGGGTTGGGCGTTTGGGGGGGGGGGGG
GGGAAGAAAGAAGAAAATTATTACACCAAGTCAGAGCAAAGAGGGGGGCCCGGGGGGG
GGGGGGGGGGGGGGGGGGGCGTTGAGGCCAAAAAGCTCAGTGTCCCAGAGAAAAGACGC
CAGTCCTGCGTCAAGACGTTTTGAGAGACAACTGGTTTGGGAAGCTTTGAGAGAAAGTGA
AGGGGGATTACGAAACAGCAGAGGAGGGAGATTTGGAGAAGATTGCGCGGCCTTTTTGGG
CTATTATGGGCGAGGCGGGAGATGGTACGTAAAGTACCTCTACTAATACGTGACGAGATG
TCGTCGAGACGGATCATAATGCTCTCATGGAAAACGCATTGCCGGCTCAAAATAGGCCTC
GTGTTTCACATTGGGCGACTCCGGGCAGCCTCTTACTCTCACACCATCACGAAGCAACATA
AGACATCCACTCGACCCGGTTGGTCCAAGACAAGAAAGAGGAGAGAAAAAAAGCATACT
TCTTACTAAGACACGGGGGCTTGGCTTCGTGCGTTGCAGTTGCAGTTGCAGTTGCAGTTGCA
GTTGCAGTGTCGAGAGATTGCCATCCCTGGACGGATCCAGAACAGAGATATGGAGACGAT
GGCTTTTTTCGGCCAAGAAGGCGTGTCTGGCGGCTGGGAGGATGAGGTGATACGCGGCGC
AACTAGTTGGCCCGCGTGAGCAGGGCGAGAGAGGGAGGACGTCGGGACCTGGTCGCCGC
CGCCGCCGCCGCCGCCGCCGCCGCCGCCGCCGCCGTCGTCGTGGTCGTGGTCGT

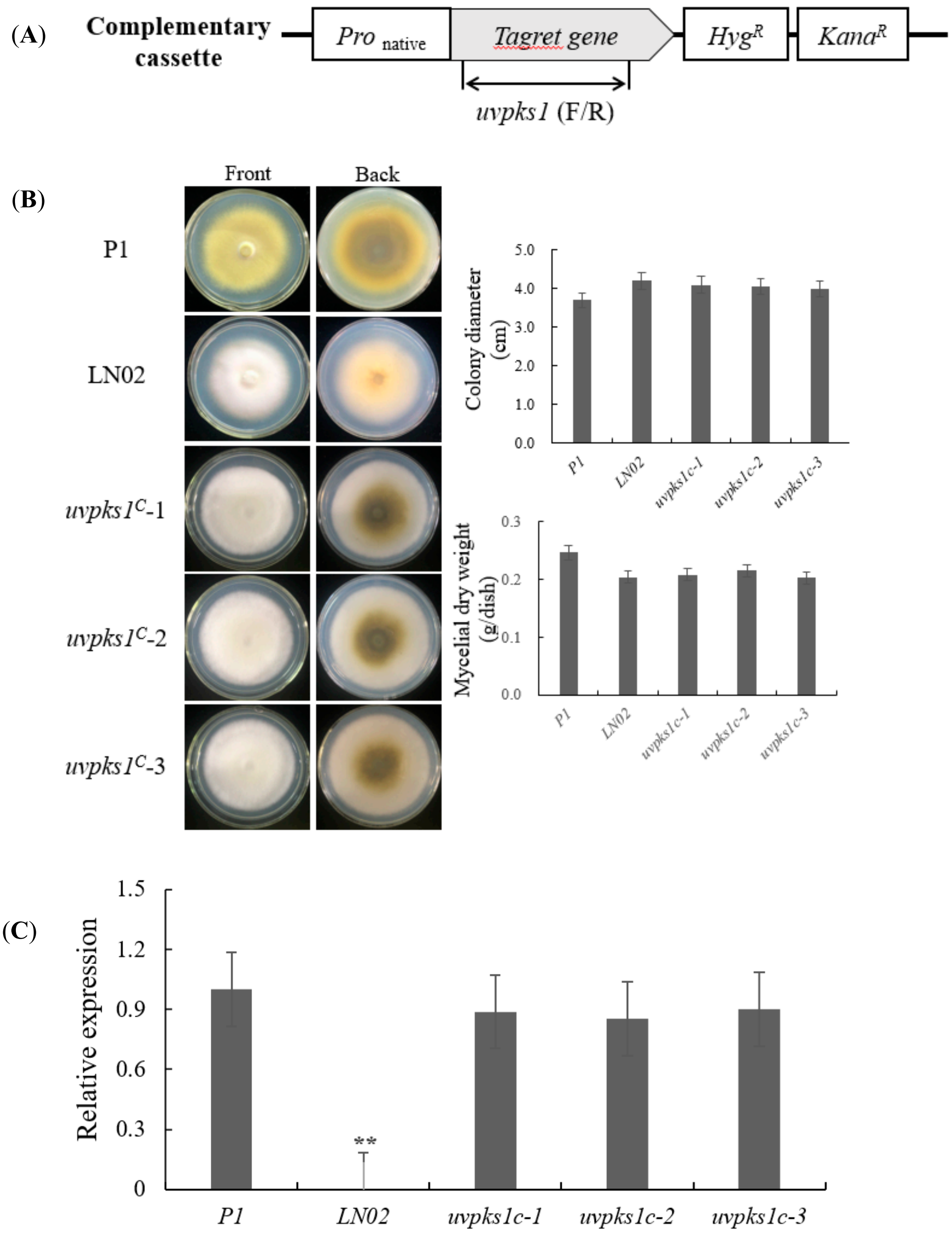

Figure S4. Complementation of normal *uvpks1* in albino strain LN02 restored to normal phenotypes.

(**A**) Schematic illustration of complementation of normal *uvpks1* in strain LN02; (**B**) Colony morphologies observed after 21 days of culture on PSA; (**C**) Expression analysis of the *uspks1* in strains P1, LN02 and complementary *uvpks1*^{C}-1. β-actin and α-tubulin were used as the controls. Data were given as means ± SD from three independent biological replicates and were compared by Student's *t*-test (** *P* < 0.01).

Table S10. The deprotonated peak [M-H]$^-$ (*m*/*z*) in HRESIMS spectra and molecular formula of the ustilaginoidins isolated from the in the complementary strain *uvpks1*^{C}-1 of albino strain LN02.

Compd. No.	RT (min) in HPLC	Ustilaginoidin	[M-H]$^-$ (*m*/*z*)	Formula
**5**	17.7	Ustilaginoidin E	531.1247	$C_{29}H_{23}O_{10}$
**6**	18.7	Ustilaginoidin K	541.1115	$C_{30}H_{21}O_{10}$
**7**	19.6	Ustilaginoidin D	545.1441	$C_{30}H_{25}O_{10}$
**8**	19.9	Isochaetochromin B_2	545.1394	$C_{30}H_{25}O_{10}$

Note: Compounds were detected after 30 days of growth in PDA medium at 28°C. The mass-to-charge ratio (*m*/*z*) of the protonated metabolites, their empirical formulas and retention time (RT, min) were indicated.

Table S11. ^{1}H and ^{13}C NMR data of the ustilaginoidins isolated from the complementary mutant *uvpks1*^{C}-1 of albino strain LN02.

Position	Ustilaginoidin E (5, CD_3COCD_3)		Ustilaginoidin K (6, CD_3COCD_3)		Ustilaginoidin D (7, CD_3COCD_3)		Isochaetochromin B_2 (8, CD_3COCD_3)	
	δ_C	δ_H mult. (*J* in Hz)	δ_C	δ_H mult. (*J* in Hz)	δ_C	δ_H mult. (*J* in Hz)	δ_C	δ_H mult. (*J* in Hz)
2, 2′	74.2	4.22 dq (11.0, 6.2)	166.2		79.1	4.21 dq (11.0, 6.2)	79.0	4.22 dq (10.9, 6.2)
	79.0	4.53 ddq (11.8, 3.1, 6.2)	166.2		79.1	4.21 dq (11.0, 6.2)	76.3	4.60 dq (3.2, 6.5)
3, 3′	43.5	2.76 dd (17.5, 3.1)	113.3		46.8	2.79 dq (10.8, 7.0)	46.7	Overlap
	46.7	2.84 dd (17.5, 11.8)	113.3		46.8	2.79 dq (10.8, 7.0)	44.9	2.78 dq (3.2, 7.1)
4, 4′	199.6		184.0		201.9		203.5	
	201.8		184.0		201.9		201.8	
4a, 4a′	102.9		102.4		102.4		102.3	
	102.3		102.4		102.4		101.7	
5, 5′	164.4		nd		165.6		166.2	
	165.7		nd		165.6		165.4	
5a, 5a′	106.6		105.9		106.6		106.5	
	106.4		105.9		106.6		106.4	
6, 6′	160.8		159.5		160.9		160.8	
	161.8		159.5		160.9		160.8	
7, 7′	100.9	6.46 s	100.8	6.50 s	101.0	6.45 s	100.1	6.46 s
	100.9	6.46 s	100.8	6.50 s	101.0	6.45 s	100.1	6.46 s
8, 8′	161.7		160.1		161.8		161.9	
	161.8		160.1		161.8		161.7	
9, 9′	105.6		106.5		105.7		105.7	
	105.6		106.5		105.7		105.7	

9a, 9a′	143.2		141.1		143.3		143.2	
	143.2		141.1		143.3		143.2	
10, 10′	99.9	5.92 s	99.3	6.34 s	100.1	5.92 s	99.9	5.92 s
	100.1	5.93 s	99.3	6.34 s	100.1	5.92 s	99.9	5.92 s
10a, 10a′	156.5		152.7		156.6		156.5	
	156.7		152.7		156.6		156.2	
2-CH3	19.8	1.38 d (6.2)	18.6	2.23 s	19.9	1.40 d (6.2)	19.8	1.40 d (6.2)
2′-CH3	20.9	1.40 d (6.2)	18.6	2.23 s	19.9	1.40 d (6.2)	16.6	1.29 d (6.5)
3-CH3			8.9	2.02 s	10.2	1.22 d (6.9)	10.0	1.22 d (7.0)
3′-CH3	10.0	1.22 d (6.9)	8.9	2.02 s	10.2	1.22 d (6.9)	9.8	1.17 d (7.3)
5-OH		15.51 s		15.51 s		15.57 s		15.57 s
5′-OH		15.57 s		15.51 s		15.57 s		15.53 s
6-OH		9.63 s		9.63 s		9.63 s		9.65 s
6′-OH		9.65 s		9.63 s		9.63 s		9.64 s
8-OH		8.47 s		8.47 s		8.48 s		8.49 s
8′-OH		8.47 s		8.47 s		8.48 s		8.46 s

Note: ^{1}H NMR spectra were measured at 400 MHz, and ^{13}C NMR spectra at 100 MHz. Nd: Not detected. The carbon atoms were numbered which were shown in Figure S5.

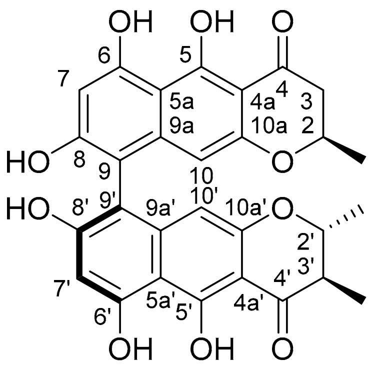

Ustilaginoidin E (**5**)

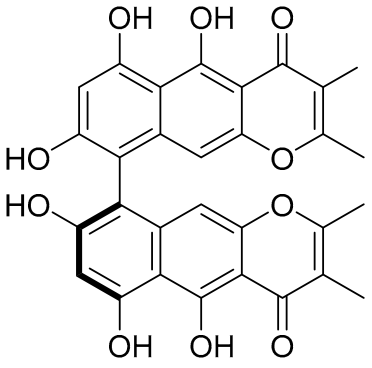

Ustilaginoidin K (**6**)

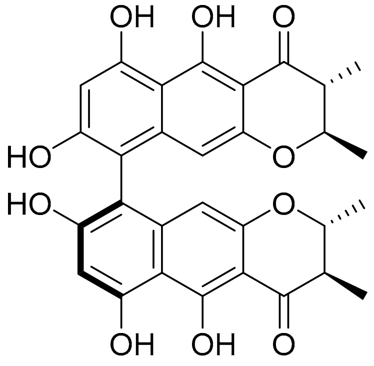

Ustilaginoidin D (**7**)

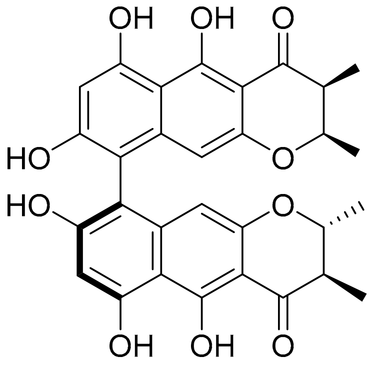

Isochaetochromin B2 (**8**)

Figure S5. Structures of four identified ustilaginoidins (**5**–**8**) with the carbon atoms numbered in the complementary mutant *uvpks1*^C-1 of strain LN02.

Table S12. Putative number of secondary metabolite BGCs in six RFS fungal strains by antiSMASH.

BGC	BGC number of RFS Fungal Strain					
	**UV8b** [14]	**IPU010** [15]	**UV_Gvt** [16]	**UV-FJ-1** [17]	**JS60-2** [18]	**LN02** This study
T1PKS	14	6	6	4	5	5
NRPS	17	6	8	6	4	4
NRPS-like	0	4	4	7	5	4
NAPAA	0	2	2	0	2	2
Terpenoid	6	4	4	4	5	4
GDS	6	0	0	0	0	0
TC	6	0	0	0	0	0
Total	49	22	24	21	21	19

Note: T1PKS: type I polyketide synthase; NRPS: nonribosomal peptide synthetase; NRPS-like: NRPS-like fragment; NAPAA: non-alpha poly-amino acids like e-polylysin; Terpenoid: biosynthesis of terpenoids; GDS: geranylgeranyl diphosphate synthases; TC: terpenoid cyclases.

Table S13. The primers used in this study.

Primer	Oligonucleotide Sequence (5′-3′)	Description
*Uvpks1*F-2	ATGGCGAACGTGTTCCAAATTG	The primers were used to amplified the sequences of *uvpks1*.
*Uvpks1*R-4	GGCGTTGATCTCGCGGTAGTC	
*Uvpks1*F-5	TCGACTCGAGGTTCTTCAGCA	
*Uvpks1*R-5	CAGGGTCGACCTAAGCATGTTG	
*Uvpks1*F-1	ATGAAGAGCGCCTCGGACAT	
Uvpks1-R	TTAGATACCCGCGCCGGTAGA	
Uvpks1-pro-P1-F	ACGACCACGACCACGACGA	The primers were used to amplified the sequences of the promoter of *uvpks1* in P1 strain.
Uvpks1-pro-P1-R	TACGGCGAAAGATGTCGAAACGAG	
Uvpks1-pro-LN02-F	ACGACCACGACCACGACGA	The primers were used to amplified the sequences of the promoter of *uvpks1* in LN02 strain.
Uvpks1-pro-LN02-R	TACGGCGAAAGATGTCGAAACGAG	
Uvpks1-pCBHT-F	aatgatggatccccgggtaccccACGACCACGACCACGACGA	The primers were used to constructed the complementary vectors.
Uvpks1-pCBHT-R	agaaatcgcaacctcgaattcTTAGATACCCGCGCCGGTAGA	
β-actin-F	CCGTGAGAAGATGACCCAGA	The primers were used as the control in RT-PCR.
β-actin-R	GGCGAAACCCTCGTAGATGG	
α-tubulin-F	GGCGTTTACAATGGCACTTC	
α-tubulin-R	CGGAACAGTTGACCAAAGG	
RT-*uvpks1*-F	ACTCCATTATGGTGCGCAGG	The primers were used for *uvpks-1* gene in RT-PCR.
RT-*uvpks1*-R	ACCCGGAAAACATGACCTGG	
